# Identification of hub driving genes and regulators of lung adenocarcinoma based on the gene Co-expression network

**DOI:** 10.1042/BSR20200295

**Published:** 2020-03-31

**Authors:** Zihao Xu, Zilong Wu, Jiatang Xu, Jingtao Zhang, Bentong Yu

**Affiliations:** 1 Department of Thoracic Surgery, The First Affiliated Hospital of Nanchang University, Nanchang, Jiangxi, 330006, China; 2 School of Public Health, Nanchang University, Nanchang, Jiangxi, 330006, China; 3 The First Clinical Medical College, Nanchang University, Nanchang, Jiangxi, 330006, China; 4 The Second Clinical Medical College, Nanchang University, Nanchang, Jiangxi, 330006, China

**Keywords:** CeRNA, Co-expressed network, Hub driving gene, LUAD

## Abstract

Lung adenocarcinoma (LUAD) remains the leading cause of cancer-related deaths worldwide. Increasing evidence suggests that circular RNAs (circRNAs) and long non-coding RNAs (lncRNAs) can regulate target gene expression and participate in tumor genesis and progression. However, hub driving genes and regulators playing a potential role in LUAD progression have not been fully elucidated yet. Based on data from The Cancer Genome Atlas database, 2837 differentially expressed genes, 741 DE-regulators were screened by comparing cancer tissues with paracancerous tissues. Then, 651 hub driving genes were selected by the topological relation of the protein–protein interaction network. Also, the target genes of DE-regulators were identified. Moreover, a key gene set containing 65 genes was obtained from the hub driving genes and target genes intersection. Subsequently, 183 hub regulators were selected based on the analysis of node degree in the ceRNA network. Next, a comprehensive analysis of the subgroups and Wnt, mTOR, and MAPK signaling pathways was conducted to understand enrichment of the subgroups. Survival analysis and a receiver operating characteristic curve analysis were further used to screen for the key genes and regulators. Furthermore, we verified key molecules based on external database, LRRK2, PECAM1, EPAS1, LDB2, and HOXA11-AS showed good results. LRRK2 was further identified as promising biomarker associated with CNV alteration and various immune cells’ infiltration levels in LUAD. Overall, the present study provided a novel perspective and insight into hub driving genes and regulators in LUAD, suggesting that the identified signature could serve as an independent prognostic biomarker.

## Introduction

In both sexes, lung cancer is one of the most common malignancies and the first leading cause of cancer-related death worldwide [1]. Despite advances in cancer therapy, the 5-year survival rate of lung cancer is only 19% [2]. Notably, 70% of lung cancer patients have locally advanced or metastatic disease at the time of diagnosis, which leaves a small window for early detection or treatment [3]. LUAD, as the most common type of lung cancer, causes a great deal of concern. Therefore, it is necessary to identify molecular markers associated with patient survival that may contribute to the development of gene-targeted therapeutic approaches.

Numerous studies demonstrate that dysregulated non-coding RNAs are essential for the initiation and progression of lung cancer. LncRNAs are non-coding RNAs ranging in length from 200 nucleotides to 100 kb, which hold substantial promise as novel biomarkers and therapeutic targets for cancer [4]. Meanwhile, circRNAs are closed loop structures without a 5′ cap or a 3′ Poly A tail. Similarly, circRNAs are also regarded as a potential molecular marker that may serve in the diagnosis and treatment of the disease, as it may play an essential role in the initiation and progression of tumors [5]. Recently, Salmena et al. proposed that mRNAs, lncRNAs, and circRNAs may regulate the expression of each other by targeting miRNAs [6]. Therefore, investigating the hub driving regulators (circRNAs, lncRNAs) is helpful to further explore the regulatory network in the progression of LUAD.

**Figure 1:**
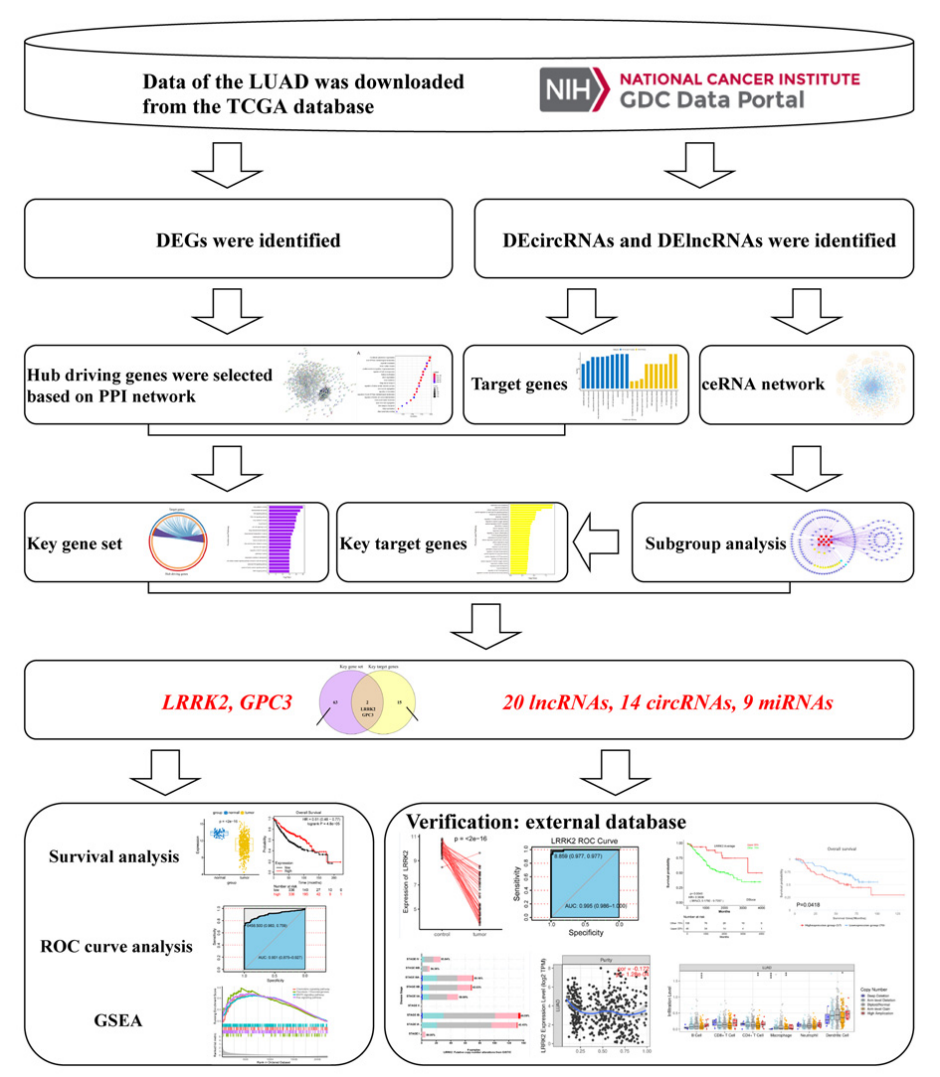
Flowchart of identifying hub driving genes and regulators

TCGA is a publicly available dataset that provides many types of genomic data [7]. For instance, Liu et al. identified a circRNA signature as a potential noninvasive biomarker for LUAD diagnosis based on TCGA-LUAD data [8]. In our study, we focused on combining the PPI network and the subgroup analysis based on the ceRNA network to explore the regulatory relationship between hub genes and regulators. In total, our research aims to systematically incorporate genes, lncRNAs, circRNAs, and miRNAs to build a co-expression network, and combine the results of subgroup analysis with survival analysis to narrow the range of valuable genes and regulators. Furthermore, external validation was used for verification and further exploration of the hub gene, Leucine-rich repeat kinase-2 (LRRK2) (Figure 1).

## Methods and materials

### Data source and pre-processing

The foundation data of LUAD were obtained from the TCGA data portal (https://tcga-data.nci.nih.gov/tcga/), including transcriptome profiling and clinical information of the patients. Specifically, the present study contained 585 patients with LUAD and 515 RNA-sequencing (RNA-seq) data. GENECODE (https://www.gencodegenes.org/) was used to annotate RNAs in the original transcriptome profiling, and a total of 16,906 genes, 187 lncRNAs, and 9810 miRNAs were annotated. The external dataset used for validation was from the Gene Expression Omnibus (GEO) database, searched for paired sample items and GSE18842 contained 44 tumor samples and 44 paired non-tumor samples was selected.

### Differentially expression analysis

By judging whether there were biological replicates, the transcriptome data from TCGA was subjected to normalization with the method of DESeq in the DESeq2 R package [9] and the method of the trimmed mean of *M*-values (TMM) in the edgeR R package [10]. In addition, to avoid a low abundance impact on the next procedure, RNAs with an FPKM value of <1 were excluded. After that, differential expression analysis of items with biological replicates was performed using the DESeq2 package and differential expression analysis of non-biologically replicated items using the edgeR package. Meanwhile, the false discovery rate (FDR) [11] adjusted *P* < 0.05 and | logFC | > 0 were used as criteria to distinguish between cancer and normal groups. A heatmap was plotted using the pheatmap R package [12].

### Functional enrichment analysis of gene ontology and KEGG pathways

Kyoto Encyclopedia of Genes and Genomes (KEGG) pathway analyses and Gene Ontology (GO) functional enrichment analyses were performed using the clusterProfiler R package [13]. A *P*-value of less than 0.05 was considered to represent a statistically significant enrichment of DEGs in pathways. Similarly, functional categories with *P* < 0.05 were considered significant.

### PPI network construction and analysis

Based on the DEGs identified, the Search Tool for the Retrieval of Interacting Genes (STRING, https://string-db.org/) database [14] was used to construct a protein–protein interaction (PPI) network. Visualization was performed using Cytoscape software [15].

### Construction of the ceRNA Network

According to the hypothesis of ceRNA, it is crucial to match the DEGs and DE-regulators. Thus, based on the results of GO and KEGG analysis, pairs of lncRNA–miRNA were established using the StarBase database (http://starbase.sysu.edu.cn/) [16], pairs of circRNA–miRNA were built using the primary data supplied by TargetScan (http://www.targetscan.org/vert_72/) [17] and miRanda (http://www.microrna.org/microrna/home.do) [18].

### Identification of a prognostic signature

Based on the key genes, miRNAs, circRNAs, and lncRNAs in the ceRNA network, the ggpubr R package and Kaplan–Meier database (http://kmplot.com/analysis/) [19] were used to assess the expression of these genes and the prognostic value of overall survival for LUAD patients, and prognosis-related genes and regulators were identified using the cut-off of *P* < 0.05. Then, ROC curve analysis and the area under the ROC curve (AUC) performed using the pROC R package [20] were used to assess the diagnostic value of the above genes and regulators.

### Gene Set Enrichment Analysis (GSEA)

First, low and high LRRK2 groups were divided based on the median expression level of LRRK2 in LUAD. Then, the limma R package [21] was used to perform differential expression analysis between low and high LRRK2 groups. Next, GSEA was performed based on the ordered list of all genes according to the logFC value using the clusterProfiler R package. Gene set permutations were performed 1000 times for each analysis. *P*-value < 0.05 was set as a significant enrichment criterion.

### Verification based on external databases

To improve the reliability of genes and regulators as independent prognostic signatures, GSE18842 was used to verify expression levels between paired samples, and the ggplot2 R package (https://ggplot2.tidyverse.org/) was used to perform the boxplot, the pROC R package was used to perform the ROC curve analysis. Further, the OSluca (http://bioinfo.henu.edu.cn/LUCA/LUCAList.jsp) database was used to verify the survival results of the prognosis-related genes based on several datasets from GEO. Moreover, lnCAR [22] was used to assess the reliability of lncRNAs. Mutation and copy number variation (CNV) in LUAD were analyzed using the cBioPortal tool [23]. TIMER database [24] was analyzed to search for a correlation of LRRK2 expression with tumor purity and the abundance of immune infiltrates in LUAD.

## Results

### Identification of DEGs and DE-regulators

Principal component analysis (PCA) was used to assess differences between groups and sample replicates within the group. The PCA result of the transcriptome data is shown in Supplementary Figure S1A, tumor tissues and normal tissues were divided into two clusters. Simultaneously, DEGs were analyzed using the DESeq2 and edgeR packages. Using *P* < 0.05 and |logFC| ≥ 0 as the cut-off criteria, 2837 DEGs (1757 up-regulated and 1080 down-regulated; Supplementary Table S1), 723 DEcircRNAs (615 up-regulated and 108 down-regulated; Supplementary Table S2), and 18 DElncRNAs (8 up-regulated and 10 down-regulated; Supplementary Table S3) were identified by comparing LUAD and normal adjunct tissues. Next, the pheatmap R package was used to perform DEG cluster analysis and for generating a heat map with gene expression level value log10 (FPKM+1) (Supplementary Figure S1B).

### KEGG pathway enrichment analysis of DEGs and protein–protein network construction

To gain insight into the underlying biological processes and pathways associated with DEGs, we performed the KEGG pathway analysis. KEGG pathway analysis revealed that DEGs in LUAD were related to many essential pathways in cancer pathogenesis, such as the MAPK pathway, ErbB pathway, calcium signaling pathway, Wnt pathway, and mTOR pathway (Supplementary Figure S2 and Table S4). Moreover, based on the DEGs, the protein–protein network was also constructed using the STRING database (Supplementary Figure S3).

### Identification of hub driving genes and functional pathway enrichment analyses

The top DEGs were further screened following the topological relation of the network. The size of the node in the interaction network graph was proportional to the degree of the node. That is, the more edges connected to the nodes, the higher the degree, the larger the nodes, and these nodes may be at the core of the network. Thus, degree > 3 was set as the criteria, 651 hub driving genes were screened and identified according to the degree of each node in the PPI network (Supplementary Table S5). We then used the clusterProfiler R package to perform KEGG pathways and GO enrichment analyses of the identified hub driving genes (Supplementary Table S6). In GO functional enrichment analysis, the hub driving genes that were significantly enriched included regulation of mitotic cell cycle phase transition, small GTPase mediated signal transduction, regulation of cell morphogenesis, and post-transcriptional regulation of gene expression (Supplementary Figure S4A). In the KEGG analysis, hub driving genes were significantly enriched in the well-known cancer-related pathways, such as mTOR, MAPK, ErbB signaling pathways, cell cycle, and non-small cell lung cancer (Supplementary Figure S4B).

### Construction of the ceRNA Network base on miRNA, lncRNA and circRNA interaction

In order to systematically explore the effect of dynamic changes in ceRNA on LUAD-related genes, the ceRNA network comprising lncRNAs, circRNAs, miRNAs, and genes was constructed. First, using TargetScan and miRanda to predict the interaction of circRNA–miRNA, 644359 potential lncRNA–miRNA pairs were formed. Similarly, 11255 potential lncRNA–miRNA pairs were matched based on StarBase. Next, the potential interaction of mRNA and lncRNA, mRNA and lncRNA were predicted based on known circRNA–miRNA and lncRNA–miRNA pairs.

In total, and based on the previous results of KEGG and GO analyses of hub driving genes and the aforementioned predictions, the ceRNA network was then constructed and it contained 502 genes, 323 miRNAs, 1009 circRNAs and 393 lncRNAs (Supplementary Figure S5).

### Identification of target genes of DE-regulators and functional pathway enrichment analyses

In order to further elucidate the possible biological functions of DE-regulators, target genes of DE-regulators (Supplementary Tables S2 and S3) were aggregated into a target set (Supplementary Table S7). The target set was then analyzed for function and pathway enrichment to observe the functions and pathways regulated by the DE-regulators. The result was as follows (Supplementary Figure S6 and Table S8), these target genes are related to many crucial pathways in cancer pathogenesis, such as Wnt, PI3K–Akt and other KEGG signaling pathways. Not only that, they also participated in many biological processes, such as lung epithelial cell differentiation.

### Relationship of target genes and hub driving genes

**Figure 2:**
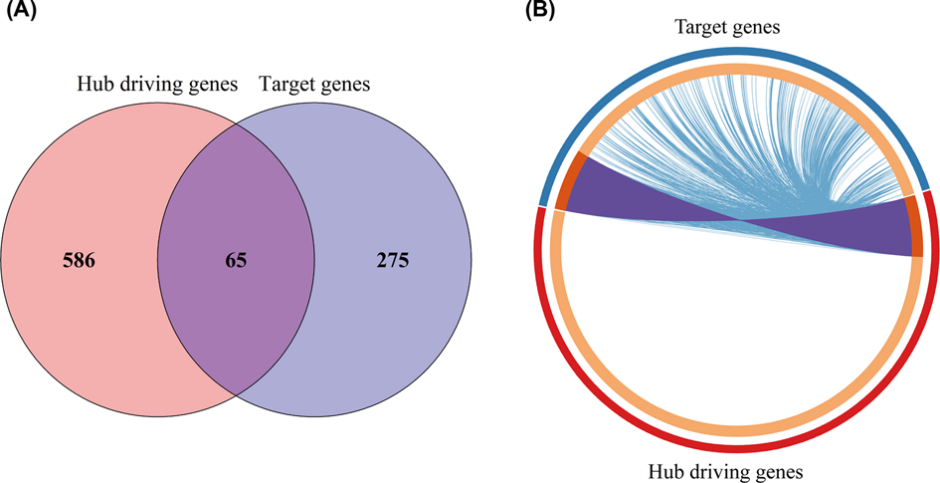
Relationship of hub driving genes and target genes (**A**) Intersection of the two. (**B**) Circos diagram: light orange represents genes that are unique in the two lists. The same genes shared by the two gene lists are linked by the purple line, and the blue line connects different genes to the same biological processes (BP).

The target genes of DE-regulators (Supplementary Table S7) were mapped to hub driving genes to obtain the intersection of the two (Figure 2A), and the 65 genes obtained were called the key gene set. As shown in the Circos diagram (Figure 2B), each arc represented the identity of each gene list externally. Moreover, internally, each arc represented a list of genes, where each gene was a dot on the arc. Dark orange represented genes that were common to both lists, which were called the key gene set (Table 1).

**Table 1:** Key gene set (common genes in hub driving genes and target genes)

Type	Symbols
Key genes	HPSE2, KIF26B, SKA3, NCAPH, KCNT2, NCKAP5, NTRK2, GPC5, TEK, DACH1, C10orf67, STXBP6, PTPRQ, ZNF385B, LAMP3, NEIL3, CD36, PRKG2, TNXB, TOP2A, CALCRL, DNAH9, LRRK2, RGS22, ITGA8, GRIK4, STRA6, KIF15, FAT3, NTNG1, BUB1B, RSPO2, SCN7A, RGS9, TMEM132C, ACOXL, TGFBR3, HHIP, SH3GL3, MGC27382, LPL, ZBTB16, KIF4A, GPC3, CENPI, TMEM232, ABI3BP, PCDH15, CDHR3, SLIT2, TRHDE, C1orf87, RXFP1, ADAMTSL3, VWA3A, KCNN4, KHDRBS2, POLQ, NCAPG, ST8SIA6, BMPER, CNKSR2, CNTN6, LRRC36, AFF3

Furthermore, light orange represented genes that were unique to both lists. The same genes shared by the two gene lists were linked by purple lines. The blue lines connected different genes to the same biological process, which indicated the amount of functional overlap between hub driving genes and the target set. Consequently, the greater the number of purple links, and the longer the dark orange arc, the more overlap there was between the hub driving genes and the target set.

### Identification of hub regulators through degree analysis of nodes

**Figure 3:**
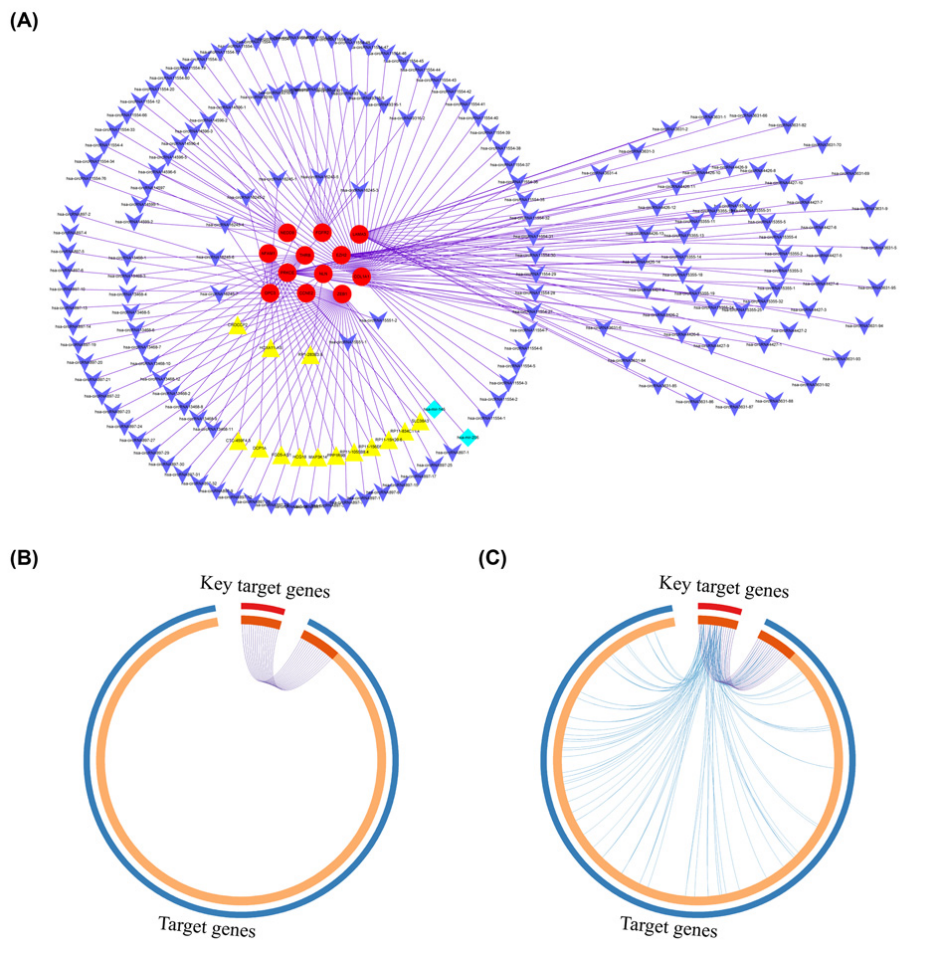
Construction of subgroup based on the degree of nodes in ceRNA network and relationship of key target genes and target genes (**A**) A total of 14 lncRNAs, 169 circRNAs, 2 miRNAs, and 12 genes are involved in the co-expression network. LncRNAs, circRNAs, miRNAs and mRNAs are represented by yellow, blue, cyan, and red. (**B**) Circos diagram: light orange represents genes that are unique in the two lists. The same genes shared by the two gene lists are linked by the purple line (**C**) Circos diagram: the blue line connects different genes to the same biological processes (BP).

Based on the ceRNA network and the results of the functional analysis of hub driving genes, the key genes that enriched in the Wnt, mTOR, and MAPK signaling pathways were picked as subgroup analysis because these signaling pathways were more closely related to cancer and more typical. Then, the criteria for mRNA filtration included logFC in mRNA expression > 1, *P*-value < 0.05, and the presence of reverse expression levels with miRNA. The subgroup contained 12 genes, 2 miRNAs, 169 circRNAs, and 14 lncRNAs (Figure 3A; Supplementary Table S9). Therefore, 183 hub regulators (169 circRNAa, 14 lncRNAs) were obtained. Through the co-expression network, we could see that both lncRNA and circRNA were involved in the formation of LUAD, and many circRNAs or genes were regulated by lncRNA and miRNA. In order to narrow down the range of genes of interest, a list of genes interacting with hub regulators was selected from the miRNA–lncRNA, miRNA–mRNA, and circRNA–miRNA interaction pairs, and a total of 17 target genes were obtained (Table 2), which were called key target genes (Figure 3B). The regulation of key target genes and target genes are shown in Figure 3C.

**Table 2:** Key target genes (target genes of hub regulators)

Symbol	logFC	AveExpr	*P*.Value	adj.P.Val	Degree
LRRK2	2.274270189	6.950077312	1.09E-27	1.38E-26	12
GPC3	2.132786284	5.698064171	3.31E-34	6.48E-33	8
NTRK3	1.778785344	2.133012079	5.42E-27	6.50E-26	18
FGFR2	1.683420265	5.953655331	5.49E-28	7.05E-27	18
FBLN5	1.478761588	6.781600047	1.18E-37	2.89E-36	8
LAMA3	1.399436258	6.889725061	7.06E-13	3.08E-12	96
PRKCE	1.398837057	5.770301235	6.59E-73	2.08E-70	28
NEDD9	1.329462751	7.86094489	1.53E-32	2.69E-31	10
SPI1	1.203072595	6.635743515	3.08E-28	4.04E-27	6
THRB	1.118872349	5.046001248	3.84E-16	2.14E-15	13
ZEB1	1.061051693	6.10035385	4.95E-36	1.10E-34	33
NFAM1	1.041363166	5.700256395	5.27E-20	3.88E-19	14
EPB41L3	1.039222467	6.153891686	4.96E-21	3.92E-20	4
NLN	-1.024693865	5.921883949	7.95E-44	2.89E-42	6
CCNE2	-1.434359893	4.794999243	9.48E-32	1.58E-30	3
EZH2	-1.808877538	5.550975326	1.84E-64	3.03E-62	33
COL1A1	-1.956268976	10.34462342	5.65E-35	1.17E-33	81

### Functional and pathway analyses of the key gene set and key target genes

**Figure 4:**
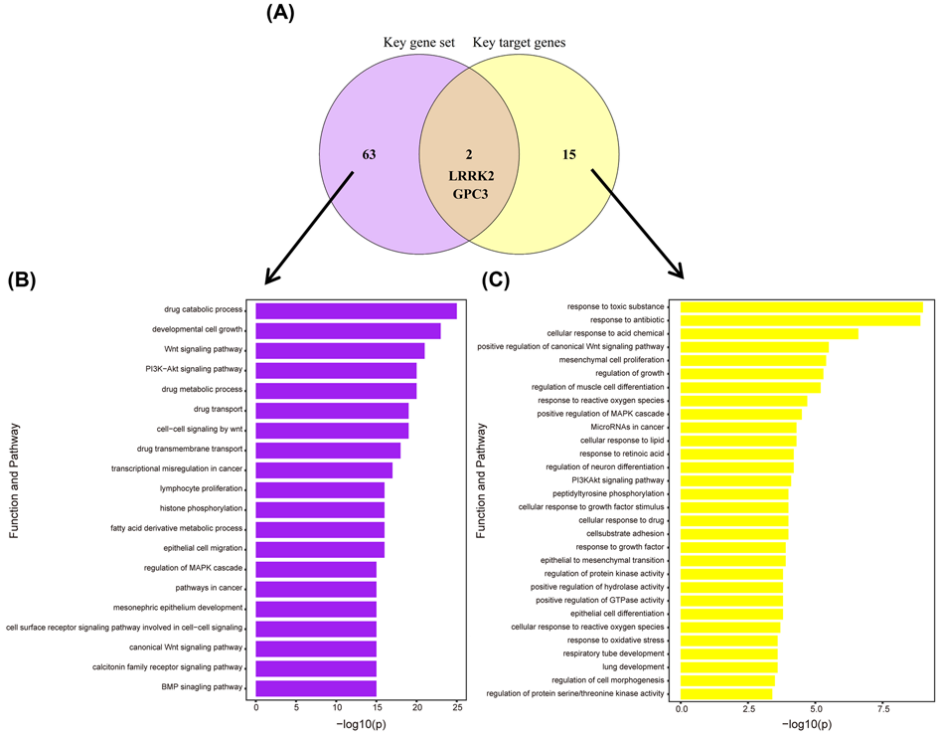
Intersection of key gene set and key target genes, and the functional pathway enrichment analysis of the two (**A**) Venn diagram. (**B**) Functional pathway enrichment of key gene set: significantly enriched GO terms and KEGG pathways (*P*-value < 0.05). (**C**) Functional pathway enrichment of key target genes: selected 30 significantly enriched GO terms and KEGG pathways (*P*-value < 0.05).

First, LRRK2 and GPC3 were obtained by taking the intersection of the key gene set and key target genes (Figure 4A). In order to confirm the reliability of the intersection genes, we performed KEGG pathway and GO analyses of the key gene set (Supplementary Table S10). As shown in Figure 4B, key gene set was not only involved in critical cancer-related pathways, such as the Wnt signaling pathway and the PI3K–Akt signaling pathway, as well as in many biological processes, including cancer transcription disorders, and histone phosphorylation. Meanwhile, the key target genes were similarly enriched in the PI3K–Akt signaling pathway, Wnt signaling pathway and some lung-related GO terms (Figure 4C and Supplementary Table S11).

### Survival analysis and ROC curve analysis

**Figure 5:**
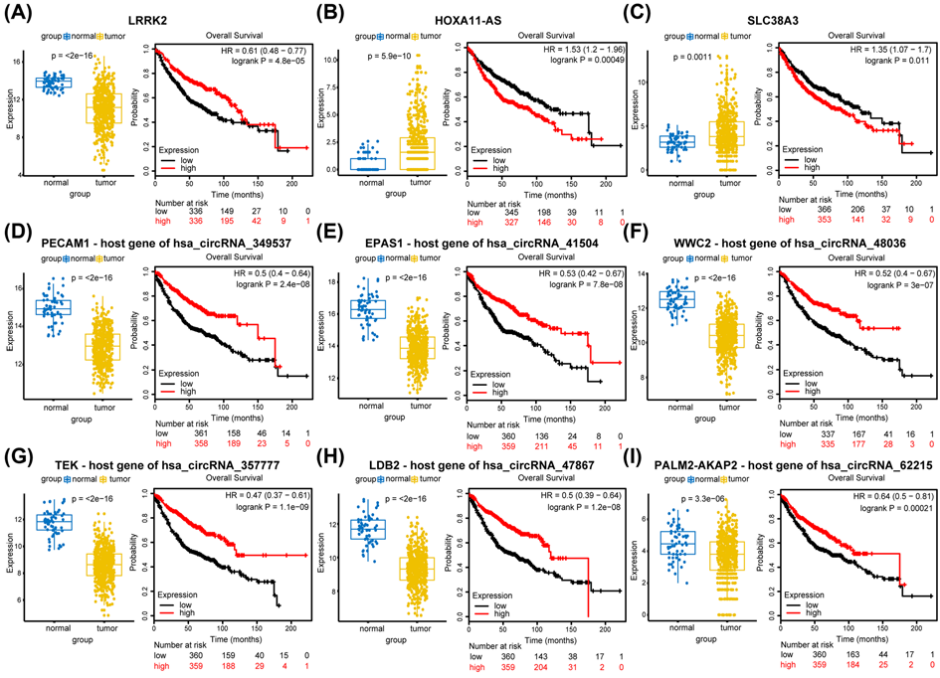
The boxplot of expression level and Kaplan–Meier curves for 1 gene, 2 lncRNAs, and 6 host genes of circRNAs associated with overall survival in LUAD (**A**) LRRK2 (**B**) HOXA11-AS (**C**) SLC38A3 (**D**) PECAM1- host gene of hsa_circRNA_349537 (**E**) EPAS1 – host gene of hsa_circRNA_41504 (**F**) WWC2 – host gene of hsa_circRNA_48036 (**G**) TEK – host gene of hsa_circRNA_357777 (**H**) LDB2 – host gene of hsa_circRNA_47867 (**I**) PALM2-AKAP2 – host gene of hsa_circRNA_62215.

First, two genes were selected by taking the intersection of the key gene set and the key target genes. Then, by combining the ceRNA network, the results of the subgroup analysis and Wnt, mTOR, MAPK signaling pathways enrichment, 14 circRNAs, 20 lncRNAs, and 9 miRNAs were obtained for survival analysis verification (Table 3). Further, the Kaplan–Meier database was used to assess the prognostic value of overall survival for LUAD patients, and screening was performed at *P* < 0.05, analysis of overall survival revealed that RNAs with prognostic values in LUAD were: one gene, two lncRNAs, and six host genes of circRNAs (Figure 5 and Table 4).

**Table 3:** Selected four types of RNAs

Type	Symbols
mRNA	LRRK2, GPC3
lncRNA	HCG18, RP1-283E3.8, RP11-508N12.4, RP11-206L10.11, HOXA11-AS, RP11-977G19.5, CROCCP2, SENP3-EIF4A1, RP11-15H20.6, MAP3K14, RP11-1055B8.4, RP11-834C11.4, RP11-156E6.1, DCP1A, CTC-459F4.3, RP11-186B7.4, PPP1R9B, XXbac-BPG32J3.20, SLC38A3, FGD5-AS1
miRNA	hsa-mir-200a-3p, hsa-mir-200c-3p, hsa-mir-146a-5p, hsa-miR-219a-5p, hsa-miR-218-5p, hsa-mir-205-5p, hsa-mir-145-5p, hsa-mir-223-3p, hsa-mir-138-5p
circRNA	hsa_circRNA_120801, hsa_circRNA_349537, hsa_circRNA_346334, hsa_circRNA_345029, hsa_circRNA_41504, hsa_circRNA_343770, hsa_circRNA_127614, hsa_circRNA_357665, hsa_circRNA_48036, hsa_circRNA_357777, hsa_circRNA_47867, hsa_circRNA_62215, hsa_circRNA_62213, hsa_circRNA_357547

**Table 4:** Host genes of circRNAs

circRNA	Host gene	chr	Strand
hsa_circRNA_349537	PECAM1	chr17:64341633-64352463	-
hsa_circRNA_41504	EPAS1	chr2:46360637-46361090	+
hsa_circRNA_48036	WWC2	chr4:183193598-183209025	+
hsa_circRNA_357777	TEK	chr9:27180239-27192623	+
hsa_circRNA_47867	LDB2	chr4:16585921-16759260	-
hsa_circRNA_62215	PALM2-AKAP2	chr9:110136126-110138539	+

As the analysis of the differential expression and the KM curve showed, HOXA11-AS and SLC38A3 presented high expression and poor prognosis in LUAD progression, which indicated that lncRNA HOXA11-AS and SLC38A3 might promote the process of LUAD, leading to a significant reduction in survival time. Conversely, LRRK2, the host genes of hsa_circRNA_349537, hsa_circRNA_41504, hsa_circRNA_48036, hsa_circRNA_357777, hsa_circRNA_47867 and hsa_circRNA_62215, respectively, showed high expression and good prognosis. This suggests a potential protective role against cancer pathogenicity. Finally, ROC curve analysis was used to investigate the diagnostic value of the above genes and regulators in distinguishing LUAD patients from normal controls. Notably, the host genes of circRNAs hsa_circRNA_349537, hsa_circRNA_41504, hsa_circRNA_48036, hsa_circRNA_357777, and hsa_circRNA_47867 provided good diagnostic values for LUAD patients with AUC > 0.9 (Supplementary Figure S7).

### Verification based on external database and further exploration of LRRK2

**Figure 6:**
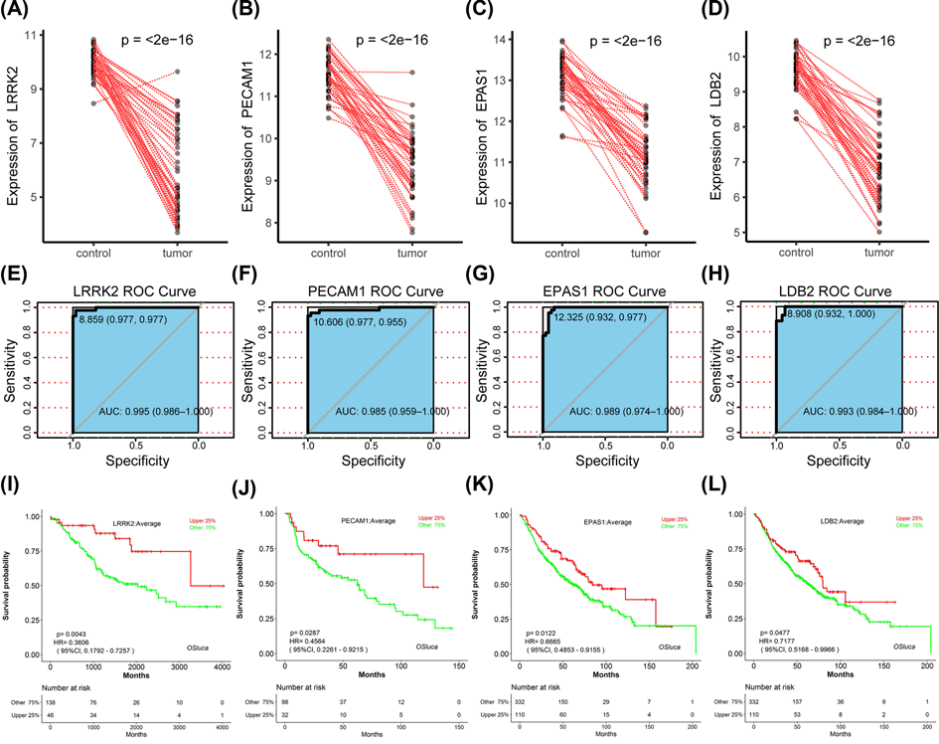
Verification of genes and the host genes of circRNAs based on external database (**A**–**D**) The expression level of LRRK2, PECAM1, EPAS1, and LDB2 in GSE18842. (**E–H**) ROC curve analysis in GSE18842. LRRK2: AUC 0.995, PECAM1: AUC 0.985, EPAS1: AUC 0.989, LDB2: AUC 0.993. (**I**–**L**) Survival analysis based on GEO database using OSluca: LRRK2-GSE41271, PECAM1-GSE4573, EPAS1-GSE68465, LDB2-GSE68465.

**Figure 7:**
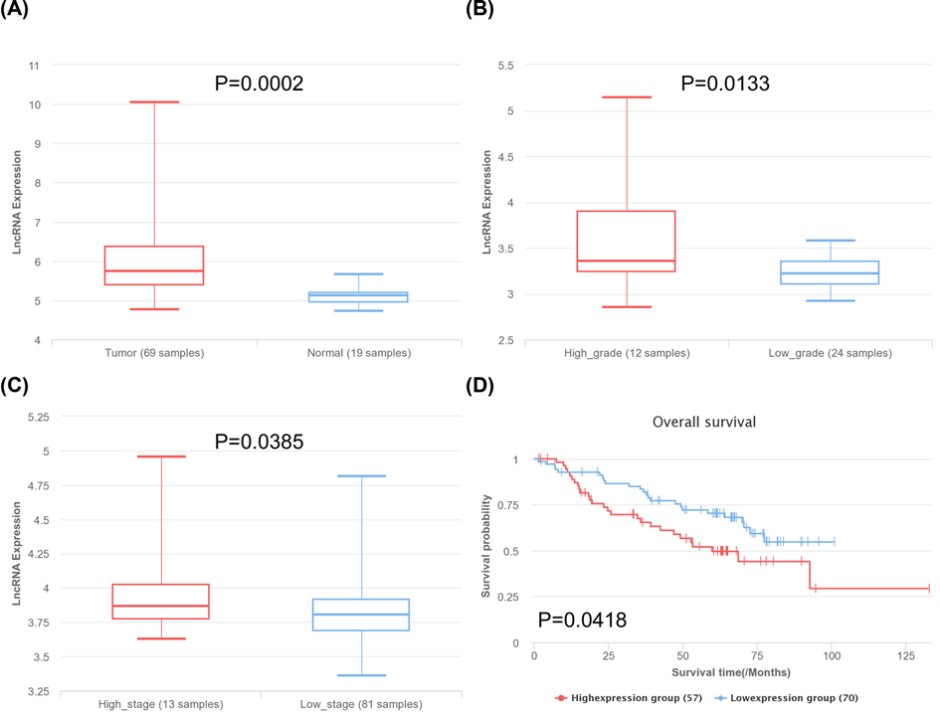
Verification of lncRNAs based on lnCAR database (**A**) Differential expression of HOXA11-AS between normal and tumor in LUAD. (**B**) Differential expression of HOXA11-AS between high grade and low grade in LUAD. (**C**) Differential expression of HOXA11-AS between high stage and low stage in LUAD. (**D**) Survival analysis of HOXA11-AS.

Base on the prognosis-related genes and the host genes of circRNAs, we specifically selected the paired samples item GSE18842 for verification. In 88 paired samples, LRRK2, PECAM1, EPAS1, and LDB2 showed significant low expression in tumor samples, which was consistent with previous results (Figure 6A–D). Meanwhile, they showed good diagnostic value with AUC > 0.9 in ROC curve analysis (Figure 6E–H). Furthermore, they also showed high expression and good prognosis in GSE41271, GSE4573, GSE68465 datasets (Figure 6I–L). As for the lncRNA, HOXA11-AS showed significant high in tumor group, high grade group, and high stage group (Figure 7A–C), presenting high expression and poor prognosis (Figure 7D).

**Figure 8:**
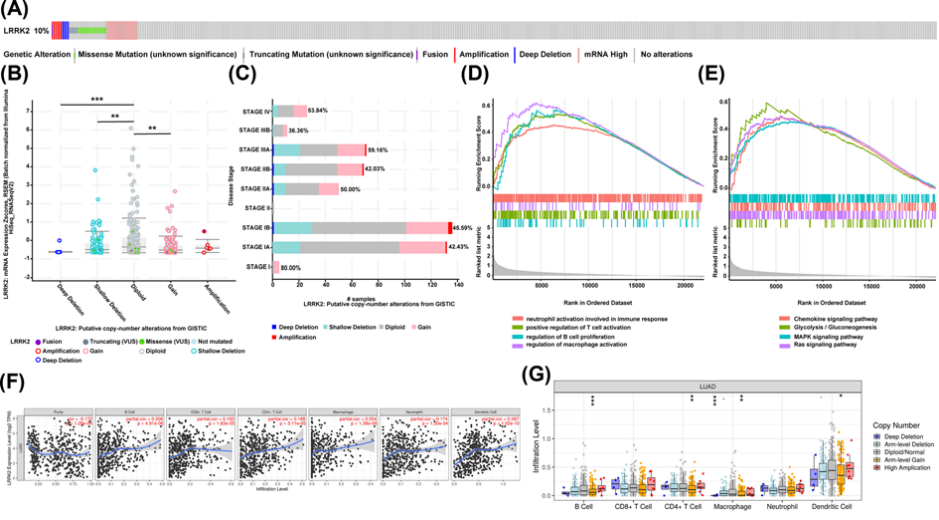
Further exploration of LRRK2 (**A**) OncoPrint of LRRK2 alterations in LUAD cohort. The different types of genetic alterations are highlighted in different colors. (**B**) LRRK2 expression in different LRRK2 CNV groups. **, *P*<0.01, ***, *P*<0.001 (**C**) Distribution of LRRK2 CNV frequency in different stage subgroups. The percentage number on the right of the bar indicates the ratio of patients with LRRK2 deep deletion, shallow deletion, and gain in all this subgroup patients. (**D** and** E**) Significantly enriched GO terms and KEGG pathways of LRRK2 by GSEA (*P*-value < 0.05). (**F**) LRRK2 expression is significantly related to tumor purity and has significant positive correlations with infiltrating levels of B cell, CD8+ T cells, CD4+ T cells, macrophages, neutrophils, and dendritic cells in LUAD. (**G**) LRRK2 CNV affects the infiltrating levels of B cells, CD4+ T CELL, macrophages, and dendritic cells in LUAD. *, *P*<0.05, **, *P*<0.01, ***, *P*<0.001.

Then, we looked to investigate LRRK2 for mutations and immune immersion. We used the OncoPrint in cBioPortal to determine the types and frequency of LRRK2 alterations in LUAD. LRRK2 was altered in 49 of 503 (10%) LUAD patients (Figure 8A). In addition, compared with the diploid group, deep deletion, shallow deletion, and gain group had lower LRRK2 expression levels (Figure 8B). Then, the frequency distribution of LRRK2 CNV patients in different stage groups was presented in Figure 8C, indicating the high occurrence of LRRK2 CNV alteration in LUAD. Next, as shown in Figure 8D,E, several interesting pathways were enriched, such as the chemokine signaling pathway, glycolysis/gluconeogenesis, MAPK signaling pathway, and the Ras signaling pathway. Notably, GSEA results showed immune-related GO terms including neutrophil activation that is involved in immune responses, positive regulation of T-cell activation, regulation of B-cell proliferation, and regulation of macrophage activation. Therefore, we investigated whether LRRK2 expression was correlated with immune infiltration levels in LUAD using TIMER database. The results showed that LRRK2 expression had significant correlations with tumor purity (*r* = −0.172, *P* = 1.28E-04) and significant correlations with various immune cells infiltration levels (Figure 8F). Mainly, LRRK2 CNV had significant correlations with infiltrating levels of B cells, CD4+ T cells, macrophages, and dendritic cells (Figure 8G).

## Discussion

Abnormal expression of non-coding RNAs has been widely observed in various diseases, and studies have shown that non-coding RNAs are essential biological functional regulators in cancer progression [25–27]. However, only a few studies systematically investigate the role and relationship of hub driving genes and regulators by co-expression network analysis. Furthermore, the introduction of the ceRNA hypothesis makes the interaction between lncRNA, circRNA, miRNA, and mRNA more reliable and necessary.

In the present study, we identified hub-driving genes, lncRNAs and circRNAs from the TCGA database. First, we identified several DEGs in cancer-related signaling pathways (Wnt, mTOR, MAPK signaling pathways) in the KEGG results, and based on these signaling pathways we screened the relevant molecules in the ceRNA network to build subgroups. Numerous re-searches indicated that Wnt signaling substantially impacted non-small cell lung cancer (NSCLC) tumorigenesis, prognosis, and resistance to therapy, with loss of Wnt signaling inhibitors by promoter hypermethylation appearing to be particularly crucial [28]. Moreover, it hasalso proved that MAPK signal amplification indeed promoted the rapid progression of established adenomas to malignant adenocarcinomas [29]. Interestingly, cell senescence in COPD has been linked to mTOR activation as well as in cancer [30].

The key gene set contained 65 genes and was the intersection of hub driving genes and target genes of DE-regulators. At the same time, KEGG and GO enrichment analyses also showed that key genes were not only involved in critical cancer-related pathways, such as Wnt signaling pathways and PI3K–Akt signaling pathways [31,32], they were also involved in many biological processes, such as cancer transcription disorders and histone phosphorylation. Through the continuous screening of the key gene set, some special cancer genes were identified. Nonetheless, even among the unselected genes, there were some well-known cancer-related genes, including TOP2A, as its interacting proteins, and its modified abnormal alterations may play an important role in CIN in human cancers [33]. Moreover, it has been reported that the high expression of NCAPH in tumor tissues of patients with colon cancer had a significantly better prognosis and survival rate than patients with low expression [34]. As for the intersection of the key gene set and key target genes, it is noteworthy that although GPC3 had not been used as the final independent prognostic indicator, it also confirmed the GPC3 over-expression in a variety of cancers [35]. LRRK2 was amplified and overexpressed in papillary renal and thyroid carcinomas in the study [36]. Moreover, they also confirmed that the down-regulation of LRRK2 in cultured tumor cells compromises MET activation and selectively reduces downstream MET signaling to mTOR and STAT3. Similarly, LRRK2 also showed anti-carcinogenic activities in the study by Jiang et al. [37], which coincided with our analysis in LUAD. With the further exploration of LRRK2, it is noteworthy that LRRK2 CNV alteration showed high incidence and LRRK2 was correlated with various immune cells’ infiltration levels, which provided multiple levels of evidence for the importance of LRRK2 in LUAD and also made LRRK2 more reliable as an independent prognostic signature.

Among the lncRNAs, although HCG18 had not been selected, it was found that lncRNA HCG18 suppressed the bladder cancer progression by cooperating with NOTCH1 and miR-34c-5p [38]. Over-expression of lncRNA SLC38A3 may promote LUAD progression leading to poor prognosis according to our study, which was similar with the study of Wang et al. in NSCLC [39]. Wang et al. confirmed that SLC38A3 activated PDK1/AKT signaling and promoted metastasis of NSCLC through regulating glutamine and histidine transport. As for our concerned lncRNA, HOXA11-AS also predicted poor prognosis in NSCLC, which could promote cell epithelial–mesenchymal transition by inhibiting miR-200b expression in NSCLC [40]. HOXA11-AS, which is a well-known marker in cancer pathogenesis further supported the selection of hub driving regulators [41]. Moreover, HOXA11-AS was significantly high in tumor group, high grade group, and high stage group, presenting high expression and poor prognosis, which indicated that HOXA11-AS might be associated with tumor metastasis.

In previous research, the role and mechanism of selected circRNAs had not been proven, however, it was through the research and verification of their host genes, that it was found that they may exist as protective factors in LUAD, which opened avenues for clinical research. Among the host genes in our study, some had previously been identified by cancer-related institutes. For instance, PECAM1 was suggested as an independent prognostic factor in clear cell renal cell carcinoma [42]. Meanwhile, the method of studying circRNAs’ host gene had been used by many studies. Zhou et al. revealed the regulatory mechanism circ-ENO1 on its host gene ENO1 and its function in glycolysis and tumor progression, and identified circ-ENO1 as a treatment target in LUAD [43]. CircITGA7 has been shown to inhibit the proliferation and metastasis of colorectal cancer cells by inhibiting the Ras signaling pathway and promoting the transcription of the host gene ITGA7 [44]. At the same time, with further research into circRNA in cancer, the potential function of these markers may be discovered. For example, it had been reported that hsa_circ_100395 regulated lung cancer cell proliferation, migration and invasion through modulating the miR-1228/TCF21 pathway [45]. Nan et al. revealed that circNOL10 could affect mitochondrial function through regulating the humanin polypeptide family and subsequently affect multiple signaling pathways, ultimately inhibiting lung cancer development [46].

Our findings provided new insights into a better understanding of the ceRNA network associated with non-coding RNAs in LUAD and potential biomarkers for diagnosis and prognosis. Meanwhile, through the screening of the degree of nodes in the PPI network, subgroup analysis, the key pathways and functions and several external databases were fully taken into account; consequently reliable results were obtained, which provided a new way of thinking for the exploration of prognosis indicators. However, our study is based on bioinformatic analysis, so further experiments are needed to confirm the conclusions made here.

## Conclusion

The present study focused on a ceRNA network to provide a novel perspective and insight into LUAD and suggested that the signature of LRRK2, HOXA11-AS, and three host genes (PECAM1-hsa_circRNA_349537, EPAS1-hsa_circRNA_41504, LDB2-hsa_circRNA_47867) could serve as independent prognostic biomarkers in LUAD.

## Supplementary Material

Supplementary Figures S1-S7 and Tables S1-S11

## Data Availability

The datasets analyzed in this study are available in The Cancer Genome Atlas (TCGA) and Gene Expression Omnibus (GEO).

## Abbreviations

AUC: area under the ROC curve
ceRNA: competing endogenous RNA
circRNA: circular RNA
DEcircRNA: differentially expressed circular RNA
DEG: differentially expressed genes
DElncRNA: differentially expressed long non-coding RNA
GEO: Gene Expression Omnibus
GO: Gene ontology
GSEA: Gene set enrichment analysis
KEGG: Kyoto encyclopedia of genes and genomes
lncRNA: long non-coding RNA
LUAD: lung adenocarcinoma
miRNA: micro RNA
NSCLC: non-small cell lung cancer
PPI: protein–protein interaction
ROC: receiver operating characteristic
TCGA: The Cancer Genome Atlas
