# Supplementary material for: Identification of hub driving genes and regulators of lung adenocarcinoma based on the gene Co-expression network
Source: Biosci Rep. 2020 Mar 31;40(4):BSR20200295. doi: 10.1042/BSR20200295 (PMC7108999; doi:10.1042/BSR20200295)
Supplement: Supplementary Figures S1-S7 and Tables S1-S11 [file BSR-2020-0295_supp.pdf]

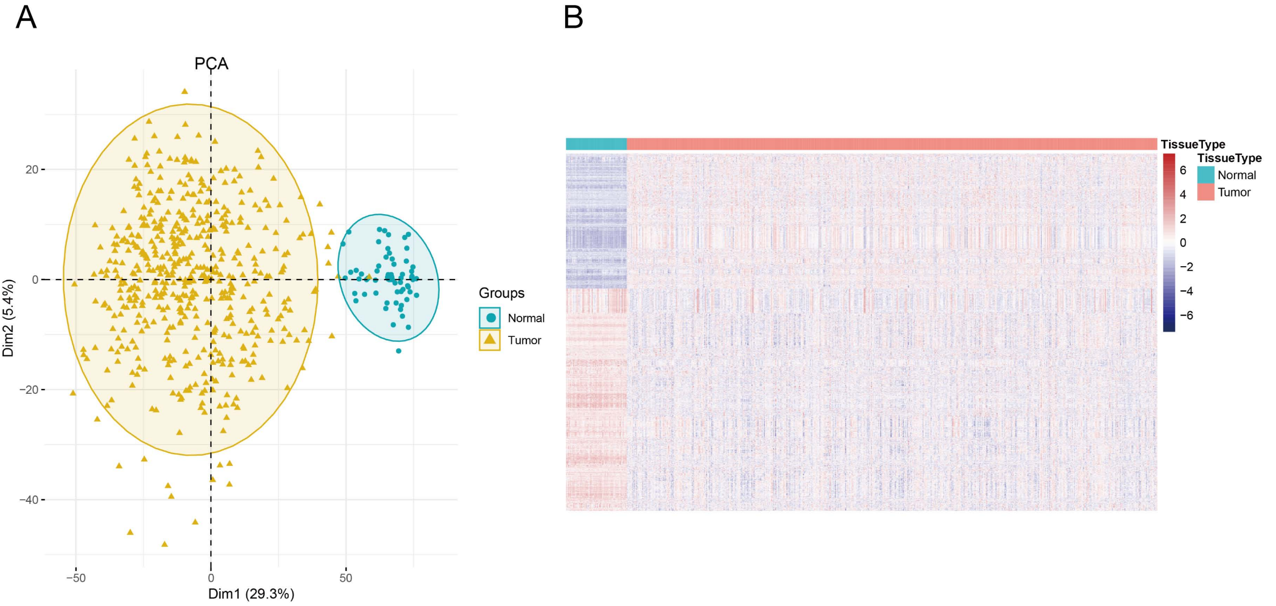
A
B
PCA
Dim1 (29.3%)
Dim2 (5.4%)
Groups
Normal
Tumor
TissueType
TissueType
Normal
Tumor

**Figure S1: PCA (A) and heatmap (B) of DEGs.**

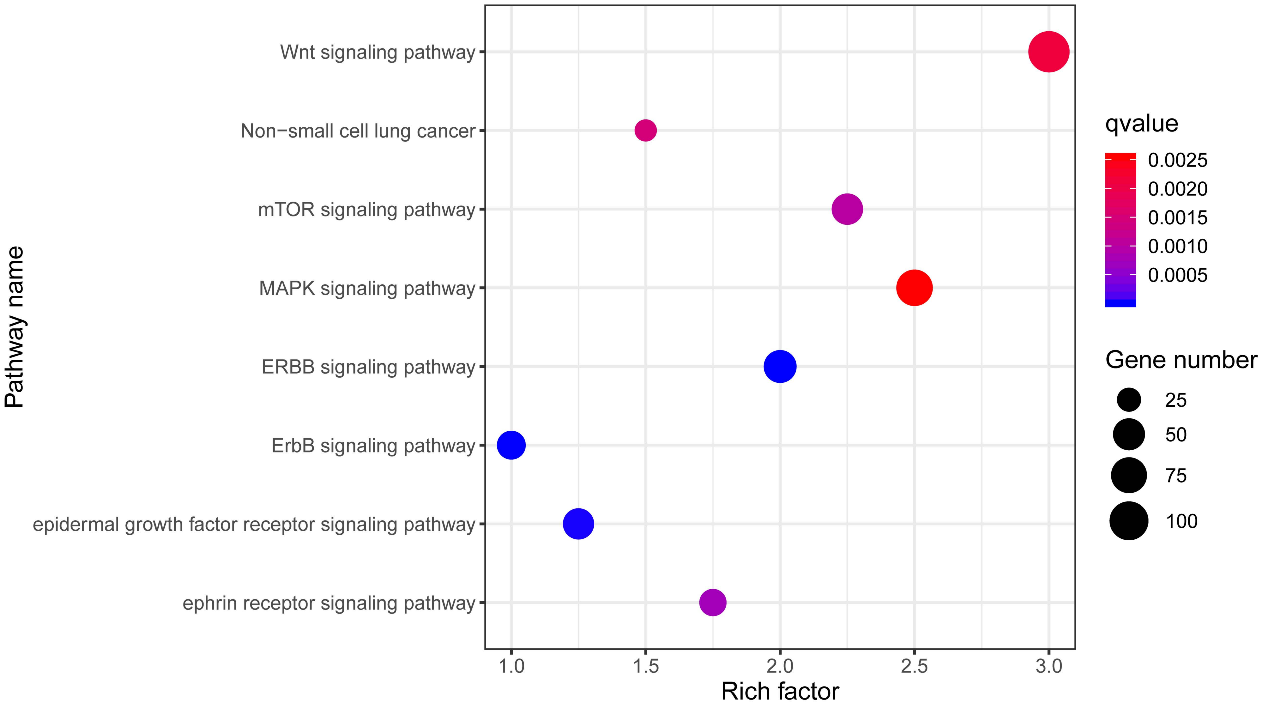
Wnt signaling pathway
Non−small cell lung cancer
mTOR signaling pathway
MAPK signaling pathway
ERBB signaling pathway
ErbB signaling pathway
epidermal growth factor receptor signaling pathway
ephrin receptor signaling pathway
Pathway name
1.0
1.5
2.0
2.5
3.0
Rich factor
qvalue
0.0025
0.0020
0.0015
0.0010
0.0005
Gene number
25
50
75
100

**Figure S2：KEGG pathway enrichment analysis of 2837 DEGs.**Eight significantly KEGG pathways (q-value < 0.05).

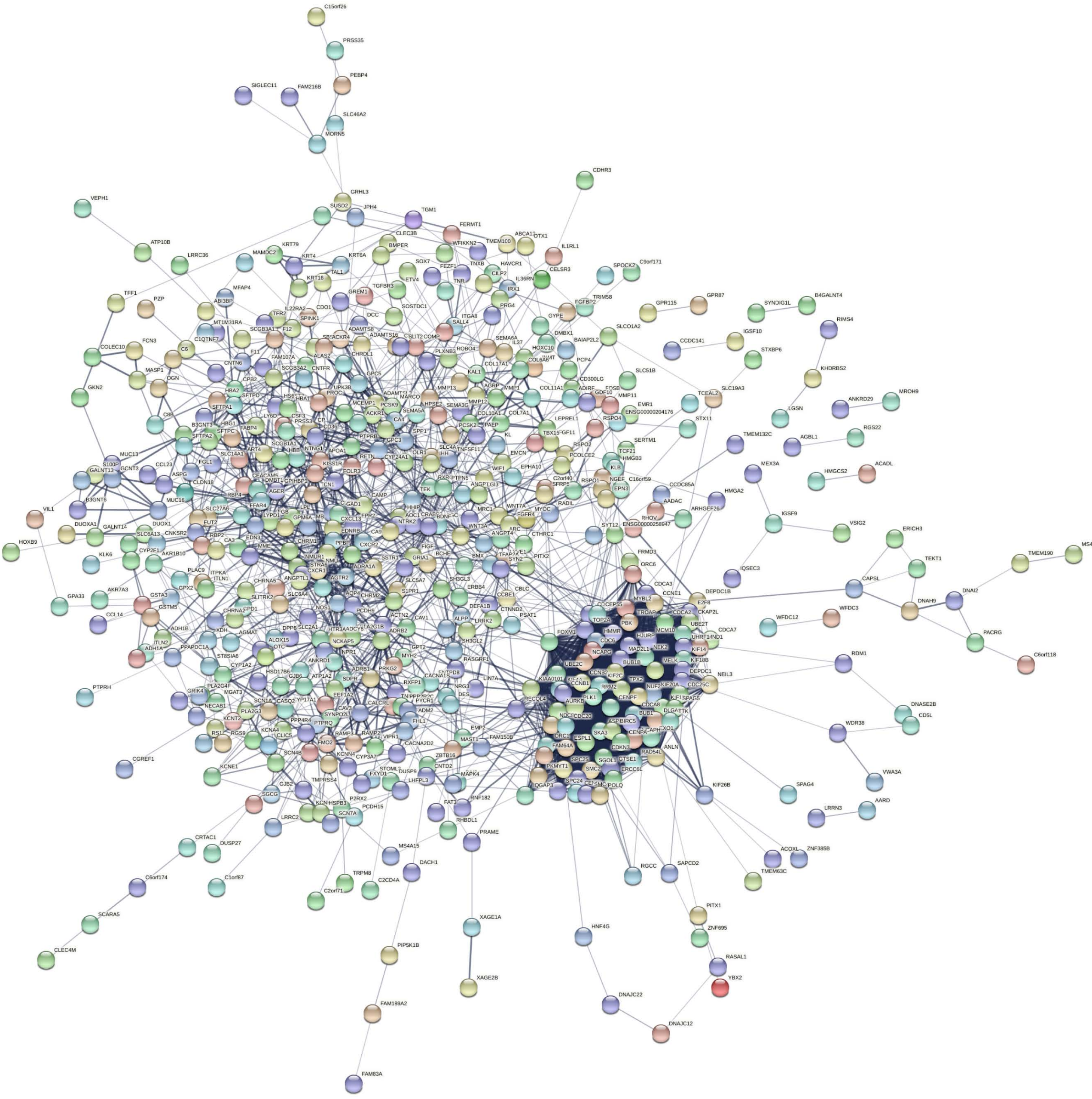

C15orf26
PRSS35
PEBP4
SIGLEC11
FAM216B
SLC46A2
MORN5
GRHL3
SUSD2
JPH4
TGM1
FERMT1
CDHR3
VEPH1
ATP10B
LRRC36
KRT79
MAMDC2
KRT4
KRT6A
CLEC3B
BMPER
WFIKKN2
TMEM100
ABCA12
OTX1
IL1RL1
SOX7
FEZF1
TNXB
HAVCR1
ETV4
TNR
CILP2
IL36RN
CELSR3
SPOCK2
C9orf171
KRT16
TAL1
TGFBR3
GREM1
IRX1
TFF1
MFAP4
PZP
ABI3BP
CDO1
DCC
SOSTDC1
PRG4
TRIM58
FGFBP2
GPR115
GPR87
SYNDIG1L
B4GALNT4
IL22RA2
TFR2
SPINK1
SALL4
ITGA8
GYPE
DMBX1
SLCO1A2
RIMS4
IGSF10
CCDC141
MT1M31RA
SCGB3A1
F12
ADAMTS8
FCN3
COLEC10
C1QTNF7
C6
F11
ACKR4
ADAMTS16
SLIT2
COMP
SEMA6A
IL37
HOXC10
BAIAP2L2
STXBP6
KHDRBS2
CNTN6
FAM107A
ALAS2
CHRDL1
PLXNB3
ROBO4
COL6A6
PCP4
SLC51B
MASP1
OGN
SCGB3A2
CNTFR
GPC5
COL17A1
KAL1
CD300LG
GKN2
CPB2
HBA2
SFTPD
UPK3B
ADAMTSL3
MMP13
AGRP
MMP1
COL11A1
ADIRF
FOSB
GDF10
MMP11
TCEAL2
SLC19A3
ANKRD29
MROH9
LGSN
HBA1
PROC
MCEMP1
PCSK9
MARCO
HPSE2
SEMA3G
MMP12
EMR1
ENSG00000204176
SFTPA1
C8B
LY6D
CSF3
SEMA5A
ACKR1
CA4
COL10A1
COL7A1
LEPREL1
STX11
B3GNT3
HBG1
FABP4
PRSS3
CP
CD36
SPP1
PCSK2
PAEP
RSPO4
TMEM132C
AGBL1
RGS22
SFTPA2
SFTPC
PTPRB
GPC3
TBX15
GFI1
SERTM1
SLC14A1
ART4
SCGB1A1
HBB
NTNG1
APOA1
CYP24A1
OLR1
SLC4A
IHH
TNFSF11
KL
RSPO2
TCF21
HMGB3
MEX3A
HMGCS2
ACADL
MUC13
S100P
GALNT13
GCNT3
CCL23
FGL1
KISS1R
RETN
FOLR3
EMCN
EPHA10
PCOLCE2
KLB
C16orf59
CCDC85A
HMGA2
ASPG
CLDN18
CEACAM5
DMBT1
GPIHBP1
TCN1
TEK
RXRG
PTPN5
ANGPTL7
LGI3
C2orf40
SFRP5
RSPO1
NGEF
EPN3
B3GNT6
MUC16
AGER
CAMP
AADAC
VIL1
RBP4
SLC27A6
FFAR4
FGB
LYPD1
LPL
GAD1
HHIP
WNT7A
MYOC
RADIL
RHOV
ARHGEF26
IGSF9
DUOXA1
GALNT14
DUOX1
FUT2
CNKSR2
GPM6A
MB
CXCL13
FPR2
NTRK2
AOC1
CRABP
BDNF
MRC1
FGFR4
SYT12
ENSG00000258947
VSIG2
ERICH3
HOXB9
SLC6A13
RBP2
CA3
EDN3
EDNRB
CA9
WNT3A
ANGPT4
CTHRC1
KLK6
CYP2F1
AKR1B10
MME
CHRM1
PPBP
CXCR2
FIGF
BCHE
BMX
TFAP2A
SYN2
PITX2
FRMD3
TEKT1
MS4A
TMEM190
PLAC9
ITPKA
ITLN1
NMUR1
NMU
STRA6
CXCR1
SSTR1
ADRA1A
GRIA1
SH3GL3
ORC6
GPA33
GPX2
AKR7A3
CHRNA5
ANGPTL1
AGTR2
SLC5A7
ERBB4
CBLC
CDCA3
CAPSL
GSTA3
SLITRK2
SLC6A4
AQP4
CHRM2
S1PR1
DEFA1B
CCBE1
CCNE1
DEPDC1B
E2F8
DNAH9
DNAI2
GSTM5
CHRNA2
CTNND2
PSAT1
CDCEP55
MYBL2
TROAP
CDCA2
CKAP2L
CCL14
NOS1
PCDH9
ACTN2
CAV1
ALPP
LRRK2
TOP2A
PBK
UBE2T
WFDC12
WFDC3
ADH1B
XDH
AGMAT
DPP6
SLC2A1
HTR3A
ADCY8
PLA2G1B
ADRB2
HMMR
HJURP
MCM10
UHRF1
CDCA7
PACRG
ITLN2
PPAPDC1A
ALOX15
OTC
NCKAP5
NPR1
GPT2
SH3GL2
FOXM1
CDC6
MAD2L1
NEK2
KIF14
ADH1A
ST8SIA6
CYP1A2
ANKRD1
ADRB1
PRKG2
MYH2
RASGRF1
NCAPG
BUB1B
MELK
KIF18B
RDM1
C6orf118
HSD17B6
GJB6
ATP1A2
SDPR
CACNA1S
ENTPD8
UBE2C
KIF4A
CCNB2
KIF2C
TPX2
DEPDC1
NEIL3
PTPRH
GRIK4
PLA2G4F
MGAT3
EEF1A2
RXFP1
PPP2R2C
PYCR1
NRG3
DES
LIN7A
KIAA0101
CCNB1
RRM2
KIF20A
CDC25C
SCN1A
CASQ2
CYP17A1
RECQL4
AURKB
PLK1
CENPF
NUF2
SPAG5
TTK
NECAB1
PLA2G3
SYNPO2L
CAV3
CALCRL
ADM2
CDCA8
DLGAP5
CDC20
BUB1
BIRC5
KCNT2
PPP4R4
PTPRQ
FHL1
NDC80
ASPM
NCAPH
EXO1
DNASE2B
CD5L
RGS9
KCNA4
CLIC5
RAMP3
RAMP2
EMP2
MAST1
FAM150B
ORC1
ESPL1
SKA3
CENPA
WDR38
FMO2
VIPR1
CACNA2D2
FAM64A
CDKN3
RAD54L
ANLN
CGREF1
SCN4B
KCNN4
CYP3A7
ZBTB16
SPC25
SGOL1
GTSE1
KCNE1
TMPRSS4
FXYD1
STOML3
DUSP9
CNTD2
MAPK4
PKMYT1
SMC2
ERCC6L
LHFPL3
SPC24
POLQ
IQGAP3
KIF26B
SPAG4
VWA3A
GJB2
SGCG
P2RX2
HSPB3
RNF182
FAT3
LRRN3
AARD
SCN7A
PCDH15
LRRC2
RHBDL1
PRAME
CRTAC1
DUSP27
MS4A15
ACOXL
ZNF385B
TMEM63C
RGCC
SAPCD2
C1orf87
TRPM8
C2CD4A
DACH1
C6orf174
C2orf71
PITX1
ZNF695
SCARA5
HNF4G
XAGE1A
RASAL1
YBX2
CLEC4M
PIP5K1B
XAGE2B
DNAJC22
FAM189A2
DNAJC12
FAM83A

**Figure S3: PPI network based on DEGs.**

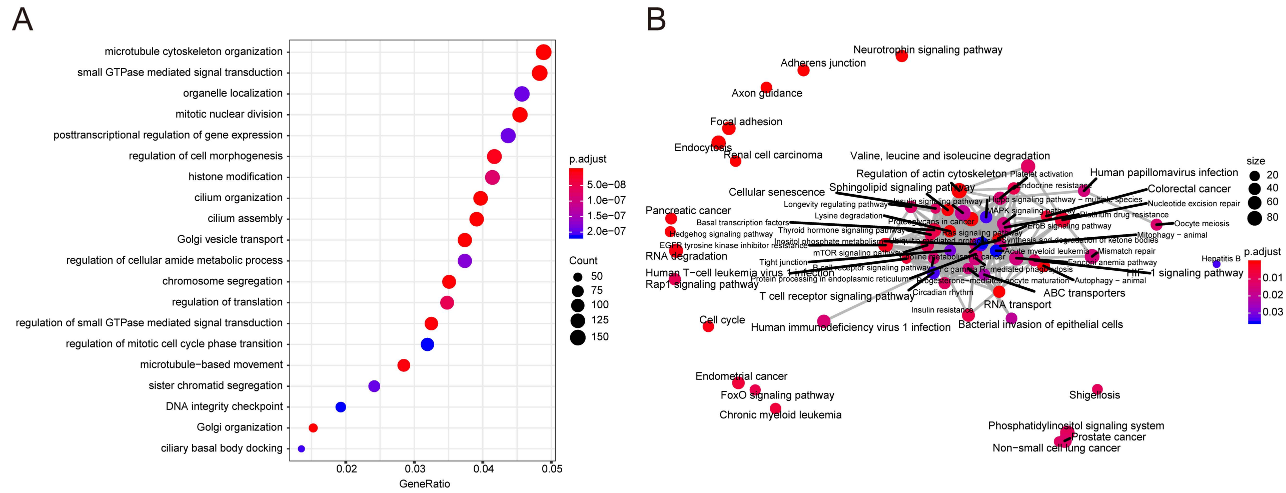

A
microtubule cytoskeleton organization
small GTPase mediated signal transduction
organelle localization
mitotic nuclear division
posttranscriptional regulation of gene expression
regulation of cell morphogenesis
histone modification
cilium organization
cilium assembly
Golgi vesicle transport
regulation of cellular amide metabolic process
chromosome segregation
regulation of translation
regulation of small GTPase mediated signal transduction
regulation of mitotic cell cycle phase transition
microtubule−based movement
sister chromatid segregation
DNA integrity checkpoint
Golgi organization
ciliary basal body docking
0.02
0.03
0.04
0.05
GeneRatio
p.adjust
5.0e−08
1.0e−07
1.5e−07
2.0e−07
Count
50
75
100
125
150
B
Neurotrophin signaling pathway
Adherens junction
Axon guidance
Focal adhesion
Endocytosis
Renal cell carcinoma
Valine, leucine and isoleucine degradation
Regulation of actin cytoskeleton
Platelet activation
Human papillomavirus infection
Endocrine resistance
Colorectal cancer
Sphingolipid signaling pathway
Cellular senescence
Insulin signaling pathway
Hippo signaling pathway − multiple species
Nucleotide excision repair
Longevity regulating pathway
MAPK signaling pathway
Platinum drug resistance
Pancreatic cancer
Lysine degradation
Proteoglycans in cancer
ErbB signaling pathway
Oocyte meiosis
Basal transcription factors
Thyroid hormone signaling pathway
Ras signaling pathway
Hedgehog signaling pathway
Mitophagy − animal
Inositol phosphate metabolism
Ubiquitin mediated proteolysis
Synthesis and degradation of ketone bodies
EGFR tyrosine kinase inhibitor resistance
mTOR signaling pathway
Acute myeloid leukemia
Mismatch repair
RNA degradation
Choline metabolism in cancer
Tight junction
Fanconi anemia pathway
Hepatitis B
B cell receptor signaling pathway
Fc gamma R−mediated phagocytosis
Human T−cell leukemia virus 1 infection
HIF−1 signaling pathway
Protein processing in endoplasmic reticulum
Progesterone−mediated oocyte maturation
Autophagy − animal
Rap1 signaling pathway
Circadian rhythm
ABC transporters
T cell receptor signaling pathway
Insulin resistance
RNA transport
Cell cycle
Human immunodeficiency virus 1 infection
Bacterial invasion of epithelial cells
Endometrial cancer
FoxO signaling pathway
Chronic myeloid leukemia
Shigellosis
Phosphatidylinositol signaling system
Prostate cancer
Non−small cell lung cancer
size
20
40
60
80
p.adjust
0.01
0.02
0.03

**Figure S4: Functional and pathway analysis of 651 hub driving genes identified by PPI network** (A) GO enrichment of 651 hub driving genes: selected 20 significantly enriched GO terms (p-value < 0.05). (B) KEGG pathway enrichment of 651 hub driving genes: selected 65 significantly enriched KEGG pathways (p-value < 0.05).

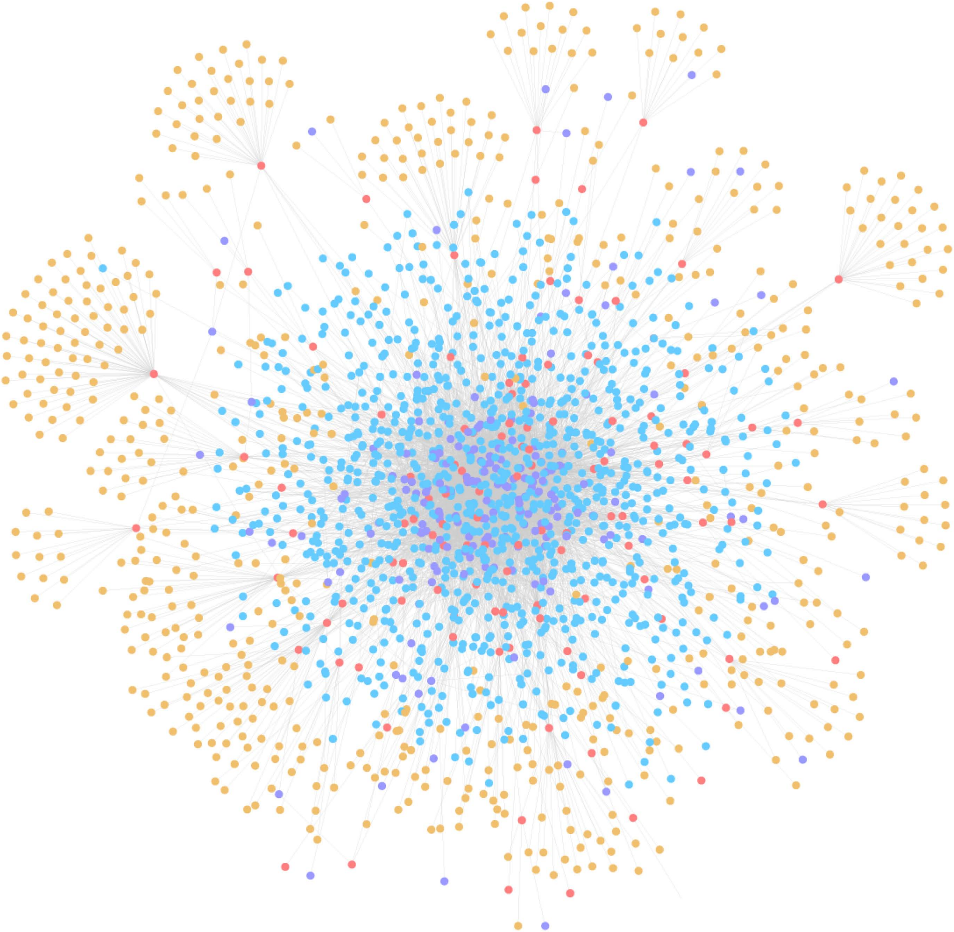

**FigureS5: Construction of the ceRNA network based on gene, miRNA, lncRNA and circRNA interaction.** A total of 393 lncRNAs, 1009 circRNAs, 323 miRNAs, and 502 genes are involved in the co-expression network. LncRNAs, circRNAs, miRNAs and genes are represented by red, yellow, purple, and blue.

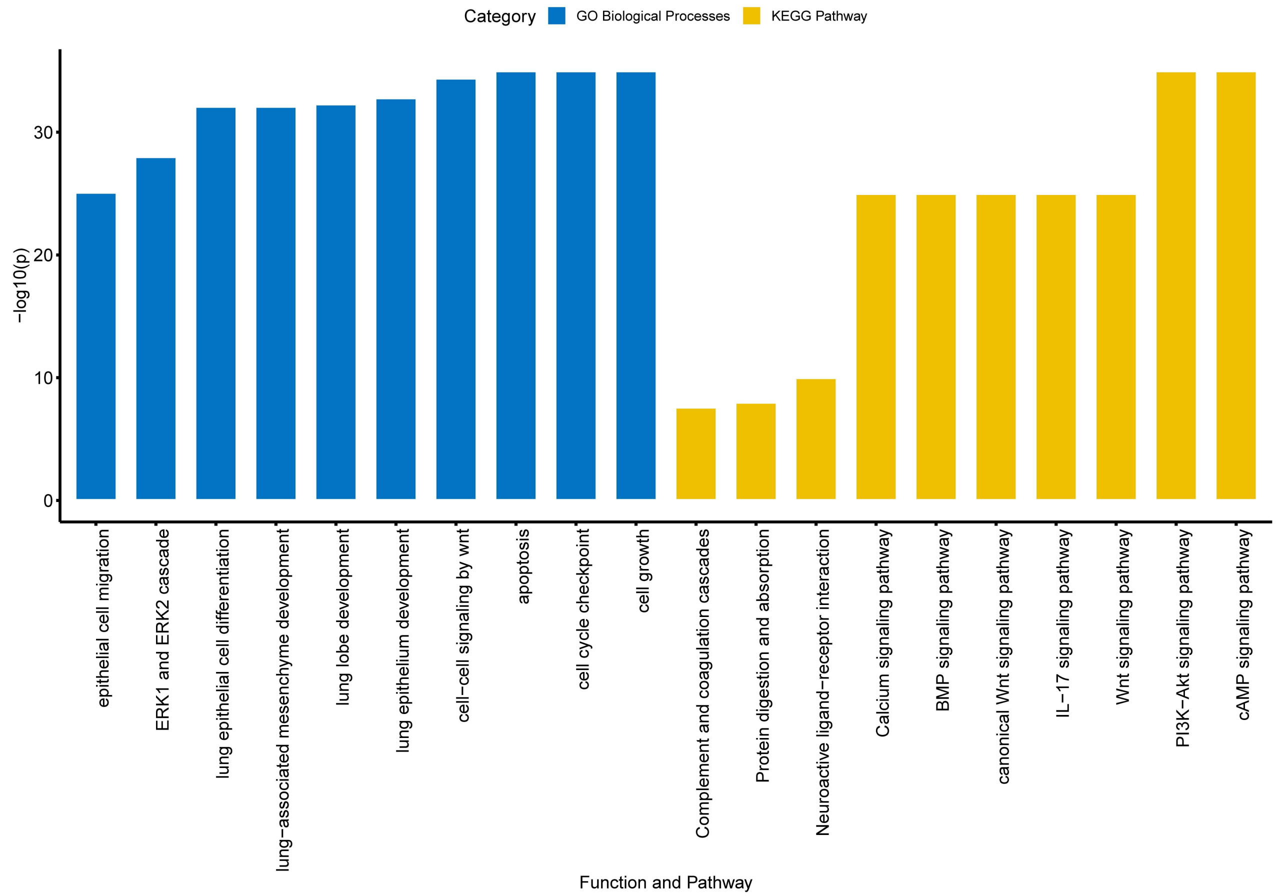

Category
GO Biological Processes
KEGG Pathway
-log10(p)
0
10
20
30
epithelial cell migration
ERK1 and ERK2 cascade
lung epithelial cell differentiation
lung−associated mesenchyme development
lung lobe development
lung epithelium development
cell−cell signaling by wnt
apoptosis
cell cycle checkpoint
cell growth
Complement and coagulation cascades
Protein digestion and absorption
Neuroactive ligand−receptor interaction
Calcium signaling pathway
BMP signaling pathway
canonical Wnt signaling pathway
IL−17 signaling pathway
Wnt signaling pathway
PI3K−Akt signaling pathway
cAMP signaling pathway
Function and Pathway

**Figure S6: Functional and pathway analysis of 340 target genes.** GO and KEGG enrichment of 340 target genes: selected 10 significantly enriched GO terms and 10 significantly enriched KEGG pathways (p-value < 0.05).

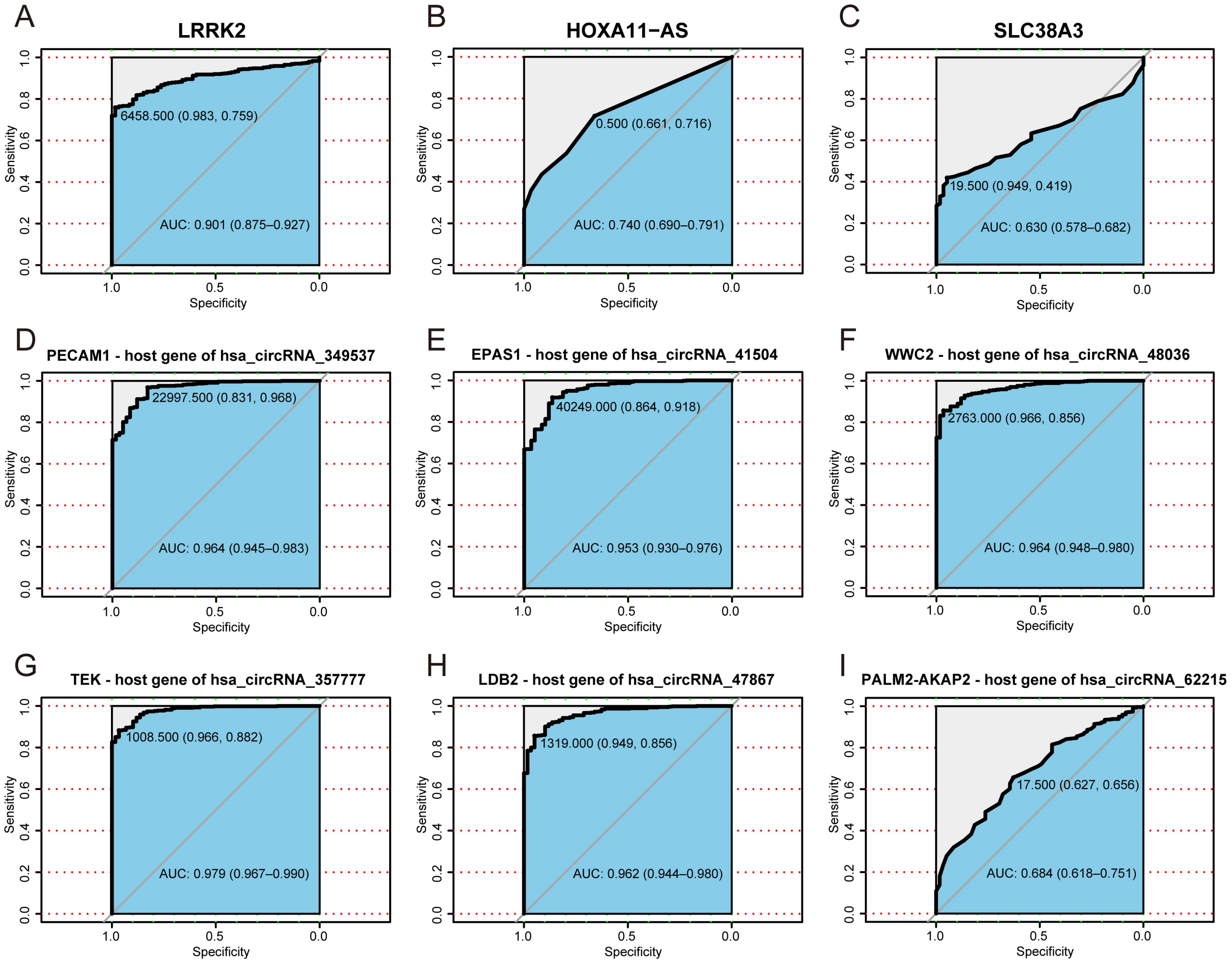

A
LRRK2
6458.500 (0.983, 0.759)
AUC: 0.901 (0.875–0.927)
B
HOXA11−AS
0.500 (0.661, 0.716)
AUC: 0.740 (0.690–0.791)
C
SLC38A3
19.500 (0.949, 0.419)
AUC: 0.630 (0.578–0.682)
D
PECAM1 - host gene of hsa_circRNA_349537
22997.500 (0.831, 0.968)
AUC: 0.964 (0.945–0.983)
E
EPAS1 - host gene of hsa_circRNA_41504
40249.000 (0.864, 0.918)
AUC: 0.953 (0.930–0.976)
F
WWC2 - host gene of hsa_circRNA_48036
2763.000 (0.966, 0.856)
AUC: 0.964 (0.948–0.980)
G
TEK - host gene of hsa_circRNA_357777
1008.500 (0.966, 0.882)
AUC: 0.979 (0.967–0.990)
H
LDB2 - host gene of hsa_circRNA_47867
1319.000 (0.949, 0.856)
AUC: 0.962 (0.944–0.980)
I
PALM2-AKAP2 - host gene of hsa_circRNA_62215
17.500 (0.627, 0.656)
AUC: 0.684 (0.618–0.751)
Sensitivity
Specificity

**Figure S7: ROC curve analysis of selected prognostic signature for the diagnosis of LUAD.** (A) LRRK2: AUC 0.901. (B) HOXA11-AS: AUC 0.740. (C) SLC38A3: AUC 0.630. (D) PECAM1- host gene of hsa_circRNA_349537: AUC 0.964. (E) EPAS1 – host gene of hsa_circRNA_41504: AUC 0.953. (F) WWC2 – host gene of hsa_circRNA_48036: AUC 0.964. (G) TEK – host gene of hsa_circRNA_357777: AUC 0.979. (H) LDB2 – host gene of hsa_circRNA_47867: AUC 0.962. (I) PALM2-AKAP2 – host gene of hsa_circRNA_62215: AUC 0.684.

Table Supplementary 1: Differentially expressed genes of LUAD

| Symbol | logFC | AveExpr | P.Value | adj.P.Val | Regulate |
|---|---|---|---|---|---|
| SFTPC | 5.965548022 | 7.793483642 | 1.20E-38 | 3.12E-37 | Up |
| SLC6A4 | 5.822445505 | 2.347200636 | 2.75E-84 | 3.14E-81 | Up |
| ITLN2 | 4.970585463 | 1.762531857 | 4.55E-77 | 2.17E-74 | Up |
| CLDN18 | 4.960270856 | 5.57052065 | 3.74E-41 | 1.15E-39 | Up |
| LGI3 | 4.779978809 | 3.227739445 | 2.22E-55 | 1.71E-53 | Up |
| C13orf36 | 4.741437888 | 1.488387961 | 6.74E-93 | 2.77E-89 | Up |
| AGER | 4.693623332 | 6.471155601 | 5.71E-74 | 1.99E-71 | Up |
| CD300LG | 4.399526681 | 1.664473451 | 1.59E-76 | 6.66E-74 | Up |
| FABP4 | 4.343763138 | 3.956220625 | 3.77E-75 | 1.38E-72 | Up |
| GPM6A | 4.263887681 | 3.253939052 | 3.46E-71 | 9.48E-69 | Up |
| CA4 | 4.252755899 | 2.737415109 | 2.99E-55 | 2.27E-53 | Up |
| HBA1 | 4.231314078 | 4.210514717 | 1.23E-42 | 4.16E-41 | Up |
| UPK3B | 4.164315688 | 4.081405715 | 1.57E-55 | 1.22E-53 | Up |
| ANKRD1 | 4.118495294 | 3.168839565 | 1.58E-56 | 1.32E-54 | Up |
| SCGB1A1 | 4.105268608 | 5.509245273 | 2.16E-22 | 1.88E-21 | Up |
| CNTN6 | 3.96993595 | 2.159717104 | 3.90E-57 | 3.44E-55 | Up |
| CPB2 | 3.916695838 | 3.076489379 | 7.05E-37 | 1.63E-35 | Up |
| SH3GL3 | 3.89777528 | 1.054349378 | 2.62E-90 | 6.72E-87 | Up |
| GKN2 | 3.866140028 | 3.415607128 | 3.49E-31 | 5.61E-30 | Up |
| SOSTDC1 | 3.865401604 | 2.802547107 | 6.84E-44 | 2.51E-42 | Up |
| WIF1 | 3.852962994 | 4.583039707 | 1.51E-32 | 2.64E-31 | Up |
| LOC149620 | 3.839554257 | 1.692238888 | 3.38E-51 | 1.95E-49 | Up |
| RPL13AP17 | 3.839442346 | 1.868299003 | 2.34E-57 | 2.11E-55 | Up |
| PTPRQ | 3.830945481 | 1.248976677 | 7.02E-84 | 7.59E-81 | Up |
| WNT3A | 3.768969645 | 2.272857527 | 1.15E-66 | 2.14E-64 | Up |
| GRIA1 | 3.765724438 | 2.613262958 | 3.16E-54 | 2.21E-52 | Up |
| SLCO1A2 | 3.716495305 | 1.641099484 | 2.21E-45 | 8.92E-44 | Up |
| MYOC | 3.631835976 | 1.012390359 | 2.05E-85 | 2.48E-82 | Up |
| C10orf67 | 3.623519796 | 1.168509601 | 1.81E-87 | 2.86E-84 | Up |
| CHRM1 | 3.600629317 | 1.105994032 | 1.14E-76 | 5.14E-74 | Up |
| FIGF | 3.585798524 | 3.728881294 | 8.47E-44 | 3.07E-42 | Up |
| C19orf59 | 3.574165143 | 4.640624086 | 1.24E-54 | 8.97E-53 | Up |
| TMEM100 | 3.516233713 | 4.58934492 | 1.08E-60 | 1.26E-58 | Up |
| F11 | 3.50851712 | 2.148018944 | 5.56E-41 | 1.68E-39 | Up |
| AGTR2 | 3.507991598 | 3.159732627 | 1.22E-29 | 1.75E-28 | Up |
| SFTPA1 | 3.497492913 | 9.323637888 | 8.83E-24 | 8.41E-23 | Up |
| ANGPTL7 | 3.483906577 | 0.777650493 | 1.48E-89 | 3.05E-86 | Up |
| GPD1 | 3.477105966 | 4.00683733 | 8.47E-60 | 8.97E-58 | Up |
| ADCY8 | 3.472184436 | 0.730599494 | 1.54E-77 | 7.92E-75 | Up |
| MS4A15 | 3.463964681 | 4.260266853 | 2.19E-28 | 2.89E-27 | Up |
| FAM107A | 3.45614528 | 5.245926324 | 1.02E-80 | 6.73E-78 | Up |
| KIAA0408 | 3.450107322 | 3.389251996 | 4.36E-31 | 6.95E-30 | Up |
| RS1 | 3.421287499 | 1.451834313 | 3.00E-82 | 2.40E-79 | Up |

| | | | | | |
|---|---|---|---|---|---|
| FCN3 | 3.417996175 | 4.839033311 | 1.14E-61 | 1.48E-59 | Up |
| HBB | 3.412590194 | 6.245297543 | 7.84E-53 | 4.97E-51 | Up |
| KRT4 | 3.40696451 | 3.521784017 | 6.46E-33 | 1.16E-31 | Up |
| ADH1B | 3.400825656 | 6.157537821 | 6.00E-33 | 1.07E-31 | Up |
| PEBP4 | 3.39661705 | 4.56345091 | 3.58E-29 | 4.99E-28 | Up |
| SFTPA2 | 3.366956562 | 10.07706548 | 1.07E-23 | 1.01E-22 | Up |
| HHIP | 3.36187023 | 3.917963295 | 1.94E-31 | 3.18E-30 | Up |
| ODAM | 3.355772606 | 0.931483878 | 1.52E-69 | 3.64E-67 | Up |
| C8B | 3.355251607 | 2.232380243 | 8.05E-34 | 1.53E-32 | Up |
| OVCH1 | 3.331684363 | 0.653282509 | 3.84E-92 | 1.32E-88 | Up |
| SGCG | 3.328360625 | 1.507236162 | 3.47E-78 | 1.83E-75 | Up |
| HBA2 | 3.327310145 | 6.074467882 | 1.13E-59 | 1.16E-57 | Up |
| KCNA4 | 3.318598773 | 0.943866564 | 3.71E-81 | 2.63E-78 | Up |
| ITLN1 | 3.303763708 | 1.820289494 | 8.24E-38 | 2.03E-36 | Up |
| AQP4 | 3.273021612 | 6.6048242 | 1.23E-27 | 1.54E-26 | Up |
| ADAMTS8 | 3.265044726 | 4.021885913 | 2.65E-49 | 1.36E-47 | Up |
| NCKAP5 | 3.25169054 | 4.107114786 | 1.23E-62 | 1.71E-60 | Up |
| TRHDE | 3.241400447 | 1.943838197 | 4.63E-46 | 1.94E-44 | Up |
| SCUBE1 | 3.220867318 | 3.19009257 | 2.86E-63 | 4.19E-61 | Up |
| ANGPT4 | 3.211481987 | 0.556476542 | 1.21E-104 | 1.24E-100 | Up |
| CLIC5 | 3.196443133 | 6.199644898 | 4.97E-63 | 7.13E-61 | Up |
| ACADL | 3.182319895 | 3.358623922 | 5.47E-41 | 1.66E-39 | Up |
| RTKN2 | 3.181543975 | 5.697670686 | 9.86E-98 | 6.74E-94 | Up |
| TNNC1 | 3.171708107 | 4.398378575 | 1.58E-52 | 9.92E-51 | Up |
| GPIHBP1 | 3.168970496 | 3.92429811 | 7.92E-64 | 1.24E-61 | Up |
| RBP2 | 3.161844274 | 0.773700783 | 1.74E-67 | 3.50E-65 | Up |
| CD5L | 3.156360929 | 0.835257028 | 1.96E-87 | 2.87E-84 | Up |
| RETN | 3.142197342 | 2.395100506 | 5.83E-37 | 1.36E-35 | Up |
| PLA2G1B | 3.141899981 | 3.010911647 | 1.56E-29 | 2.22E-28 | Up |
| PCDH15 | 3.072365691 | 1.355784377 | 7.96E-54 | 5.37E-52 | Up |
| CYP4B1 | 3.067413315 | 6.570000466 | 3.22E-28 | 4.19E-27 | Up |
| FOLR3 | 3.051358709 | 1.233321855 | 1.78E-49 | 9.23E-48 | Up |
| ANXA8L2 | 3.048972931 | 3.742248489 | 3.04E-33 | 5.57E-32 | Up |
| RSPO1 | 3.020034737 | 2.0176615 | 1.07E-57 | 9.74E-56 | Up |
| SH3GL2 | 3.009941663 | 1.431570803 | 4.91E-43 | 1.70E-41 | Up |
| GDF10 | 3.008461639 | 3.642680923 | 1.57E-47 | 7.11E-46 | Up |
| PI16 | 3.008111642 | 2.199824059 | 1.99E-39 | 5.46E-38 | Up |
| SEC14L3 | 2.993391982 | 1.036374604 | 1.41E-43 | 5.05E-42 | Up |
| WNT7A | 2.984868503 | 2.484405299 | 2.25E-30 | 3.38E-29 | Up |
| HTR3C | 2.983687703 | 0.439979424 | 1.46E-83 | 1.36E-80 | Up |
| GPA33 | 2.962624007 | 2.985103142 | 4.25E-46 | 1.79E-44 | Up |
| LRRC36 | 2.953048228 | 3.403625217 | 1.13E-48 | 5.42E-47 | Up |
| CSF3 | 2.950728347 | 2.679483713 | 7.54E-29 | 1.03E-27 | Up |
| C2orf71 | 2.947275561 | 0.878442248 | 1.26E-70 | 3.24E-68 | Up |
| CLEC3B | 2.94699397 | 5.540011589 | 7.35E-74 | 2.47E-71 | Up |
| CYP1A2 | 2.946107131 | 0.514130603 | 1.58E-81 | 1.16E-78 | Up |

| | | | | | |
|---|---|---|---|---|---|
| CCDC141 | 2.942081182 | 2.66404929 | 2.97E-59 | 2.97E-57 | Up |
| INMT | 2.938918682 | 6.051932671 | 2.75E-57 | 2.46E-55 | Up |
| ABCA8 | 2.932660704 | 4.443030782 | 3.24E-42 | 1.07E-40 | Up |
| RXRG | 2.91291817 | 2.149334475 | 6.91E-38 | 1.71E-36 | Up |
| FAM150B | 2.907579279 | 2.00733441 | 2.97E-53 | 1.93E-51 | Up |
| MAPK4 | 2.907443083 | 3.003826064 | 1.37E-28 | 1.84E-27 | Up |
| ST8SIA6 | 2.894589651 | 1.369688534 | 4.06E-60 | 4.49E-58 | Up |
| ADH1A | 2.884393795 | 1.731040901 | 6.66E-49 | 3.27E-47 | Up |
| C2orf40 | 2.871754466 | 2.788287255 | 1.54E-31 | 2.55E-30 | Up |
| CYP3A7 | 2.8706499 | 0.76505544 | 6.20E-69 | 1.40E-66 | Up |
| DNASE1L3 | 2.869790157 | 3.171431952 | 3.34E-39 | 9.01E-38 | Up |
| RSPO2 | 2.867962059 | 2.44701192 | 1.86E-37 | 4.47E-36 | Up |
| LOC723809 | 2.867276472 | 2.932679528 | 8.94E-29 | 1.22E-27 | Up |
| BTNL9 | 2.865278056 | 4.303379509 | 6.95E-59 | 6.86E-57 | Up |
| ADRA1A | 2.848019993 | 0.75336401 | 2.70E-73 | 8.80E-71 | Up |
| SFRP5 | 2.846119797 | 2.214389515 | 1.02E-32 | 1.81E-31 | Up |
| IHH | 2.84453784 | 1.959170324 | 1.41E-33 | 2.65E-32 | Up |
| CRTAC1 | 2.833141346 | 5.548592701 | 2.05E-30 | 3.09E-29 | Up |
| NXF3 | 2.832110945 | 1.450440187 | 2.05E-38 | 5.25E-37 | Up |
| CAV1 | 2.828324774 | 7.777042604 | 1.16E-78 | 6.44E-76 | Up |
| SYN2 | 2.827410588 | 1.37790907 | 6.38E-53 | 4.07E-51 | Up |
| SLC27A6 | 2.817902937 | 0.962791359 | 2.73E-49 | 1.39E-47 | Up |
| IL1RL1 | 2.815943292 | 3.498592255 | 4.45E-36 | 9.89E-35 | Up |
| ADRB1 | 2.815239447 | 3.745542908 | 3.88E-43 | 1.34E-41 | Up |
| ANXA8 | 2.813887534 | 3.993430273 | 2.82E-27 | 3.46E-26 | Up |
| RXFP1 | 2.809327768 | 1.917062339 | 1.23E-61 | 1.58E-59 | Up |
| FGFBP2 | 2.807788898 | 2.070356186 | 1.87E-57 | 1.69E-55 | Up |
| PGC | 2.804135933 | 6.556870886 | 2.54E-11 | 9.69E-11 | Up |
| NOS1 | 2.788286822 | 1.295174983 | 3.27E-38 | 8.24E-37 | Up |
| DES | 2.775048261 | 4.885927757 | 7.01E-39 | 1.83E-37 | Up |
| ACTN2 | 2.769665676 | 1.829896703 | 4.81E-52 | 2.95E-50 | Up |
| FHL1 | 2.765226621 | 6.403245027 | 1.04E-75 | 3.90E-73 | Up |
| MARCO | 2.752936123 | 6.522926429 | 8.46E-39 | 2.20E-37 | Up |
| CACNA2D2 | 2.750112401 | 6.239443603 | 2.02E-27 | 2.49E-26 | Up |
| STXBP6 | 2.736092745 | 3.373133255 | 3.36E-50 | 1.83E-48 | Up |
| GYPE | 2.721314189 | 2.048675178 | 4.00E-72 | 1.19E-69 | Up |
| COL6A6 | 2.719232778 | 4.404382178 | 6.88E-38 | 1.70E-36 | Up |
| ALAS2 | 2.716564531 | 0.58963272 | 1.90E-62 | 2.58E-60 | Up |
| CLEC4M | 2.711699591 | 0.578929727 | 2.75E-63 | 4.06E-61 | Up |
| PTPN5 | 2.68649989 | 0.735541252 | 6.11E-62 | 7.99E-60 | Up |
| EDNRB | 2.685404635 | 5.722973813 | 5.68E-80 | 3.43E-77 | Up |
| MGAT3 | 2.680295721 | 4.845713862 | 2.30E-60 | 2.61E-58 | Up |
| SFTPD | 2.680289376 | 7.469132762 | 6.98E-25 | 7.17E-24 | Up |
| C1orf87 | 2.673041808 | 1.862554939 | 1.05E-23 | 9.90E-23 | Up |
| C20orf85 | 2.672634822 | 3.128804162 | 8.41E-16 | 4.57E-15 | Up |
| CAMP | 2.652439431 | 1.518183626 | 9.13E-41 | 2.73E-39 | Up |

| | | | | | |
|---|---|---|---|---|---|
| FOSB | 2.651740988 | 6.60826939 | 6.83E-29 | 9.35E-28 | Up |
| IRX1 | 2.650401871 | 2.379767865 | 4.69E-23 | 4.25E-22 | Up |
| TCF21 | 2.650394607 | 5.045238553 | 6.15E-55 | 4.54E-53 | Up |
| SCARA5 | 2.641188126 | 3.563598166 | 2.91E-26 | 3.34E-25 | Up |
| SLC5A9 | 2.640463841 | 2.818818291 | 1.57E-41 | 4.98E-40 | Up |
| DNAH9 | 2.637191772 | 2.910346717 | 2.08E-18 | 1.37E-17 | Up |
| MGC27382 | 2.626023483 | 0.795064324 | 5.52E-62 | 7.27E-60 | Up |
| PPBP | 2.624966084 | 1.94405926 | 1.17E-27 | 1.47E-26 | Up |
| TCEAL2 | 2.621015405 | 2.452600401 | 1.60E-43 | 5.70E-42 | Up |
| TRIM58 | 2.617274495 | 2.150194623 | 1.64E-45 | 6.69E-44 | Up |
| CHRNA2 | 2.616791116 | 0.353913509 | 1.07E-85 | 1.38E-82 | Up |
| NECAB1 | 2.614688123 | 3.888198824 | 1.17E-67 | 2.42E-65 | Up |
| PRKG2 | 2.613590585 | 2.672340247 | 6.09E-45 | 2.38E-43 | Up |
| SDPR | 2.604962456 | 6.643802888 | 1.50E-67 | 3.04E-65 | Up |
| ARC | 2.602644103 | 2.767727588 | 2.69E-46 | 1.15E-44 | Up |
| FMO2 | 2.600938569 | 6.326995869 | 4.44E-57 | 3.88E-55 | Up |
| GCOM1 | 2.599922233 | 4.795540329 | 4.40E-70 | 1.09E-67 | Up |
| EMR3 | 2.594591573 | 1.940769299 | 7.29E-45 | 2.83E-43 | Up |
| IGSF10 | 2.594012789 | 4.578838708 | 1.02E-46 | 4.46E-45 | Up |
| FXYD1 | 2.580280428 | 3.397087106 | 2.60E-42 | 8.58E-41 | Up |
| PKHD1L1 | 2.58009675 | 2.652584822 | 9.86E-45 | 3.77E-43 | Up |
| CNTFR | 2.577713751 | 1.313384076 | 1.60E-37 | 3.85E-36 | Up |
| CD36 | 2.575421769 | 5.935923114 | 5.06E-57 | 4.36E-55 | Up |
| DPP6 | 2.573941309 | 1.538432713 | 2.27E-49 | 1.17E-47 | Up |
| VEPH1 | 2.568746228 | 4.738773438 | 1.10E-35 | 2.38E-34 | Up |
| DUOX1 | 2.566458314 | 6.175157634 | 1.75E-36 | 3.99E-35 | Up |
| SYT15 | 2.562218729 | 4.783676263 | 1.85E-51 | 1.11E-49 | Up |
| SLC19A3 | 2.557928833 | 3.942262211 | 1.12E-50 | 6.35E-49 | Up |
| AGRP | 2.548046235 | 1.21221161 | 3.92E-46 | 1.65E-44 | Up |
| EDN3 | 2.547842952 | 0.654154744 | 4.37E-49 | 2.18E-47 | Up |
| FAM189A2 | 2.545927698 | 5.032915037 | 4.93E-45 | 1.95E-43 | Up |
| SLC6A13 | 2.538100724 | 0.970489801 | 1.08E-54 | 7.87E-53 | Up |
| BDNF | 2.530587608 | 3.044241828 | 2.96E-40 | 8.54E-39 | Up |
| MME | 2.528523332 | 5.373662842 | 1.94E-45 | 7.85E-44 | Up |
| LRRN3 | 2.528358217 | 3.363650837 | 2.32E-55 | 1.78E-53 | Up |
| ASPG | 2.524585008 | 1.592682771 | 1.23E-20 | 9.45E-20 | Up |
| MAMDC2 | 2.521930363 | 4.851615387 | 3.14E-37 | 7.40E-36 | Up |
| KCNT2 | 2.520863891 | 2.979638469 | 1.10E-59 | 1.15E-57 | Up |
| NTNG1 | 2.518101283 | 2.670969864 | 2.76E-29 | 3.86E-28 | Up |
| BMPER | 2.509633592 | 3.071570277 | 7.23E-36 | 1.58E-34 | Up |
| PRG4 | 2.507145912 | 4.392165586 | 1.02E-25 | 1.12E-24 | Up |
| AADAC | 2.503916252 | 3.093961703 | 1.06E-21 | 8.84E-21 | Up |
| OVCH2 | 2.493210925 | 0.436267119 | 2.11E-79 | 1.24E-76 | Up |
| COLEC10 | 2.492855208 | 1.170274449 | 4.80E-54 | 3.32E-52 | Up |
| C11orf88 | 2.481387707 | 2.275572964 | 4.33E-19 | 2.98E-18 | Up |
| RASGRF1 | 2.479817424 | 4.926406269 | 3.05E-29 | 4.26E-28 | Up |

| | | | | | |
|---|---|---|---|---|---|
| TEK | 2.478572863 | 5.337073846 | 8.91E-81 | 6.10E-78 | Up |
| CASQ2 | 2.471744736 | 2.301991472 | 2.85E-36 | 6.43E-35 | Up |
| SLITRK2 | 2.467958993 | 1.267352953 | 3.46E-45 | 1.38E-43 | Up |
| ADRB2 | 2.463109106 | 4.560570618 | 3.61E-59 | 3.60E-57 | Up |
| LYVE1 | 2.457657551 | 4.767914731 | 1.28E-62 | 1.76E-60 | Up |
| SPOCK2 | 2.454517255 | 7.288265016 | 1.58E-76 | 6.66E-74 | Up |
| EMR1 | 2.452743875 | 3.224274083 | 1.31E-39 | 3.65E-38 | Up |
| LHFPL3 | 2.450655049 | 2.016764119 | 9.40E-28 | 1.19E-26 | Up |
| C10orf116 | 2.448412833 | 5.91287764 | 1.40E-43 | 5.05E-42 | Up |
| CDH19 | 2.443736787 | 1.065507158 | 7.67E-45 | 2.97E-43 | Up |
| C13orf30 | 2.441366575 | 3.318992202 | 6.00E-18 | 3.80E-17 | Up |
| VIPR1 | 2.439812613 | 5.450036793 | 2.16E-51 | 1.28E-49 | Up |
| MASP1 | 2.435970142 | 3.534943912 | 6.87E-44 | 2.51E-42 | Up |
| ZBTB16 | 2.429336771 | 3.528012181 | 6.74E-26 | 7.55E-25 | Up |
| DNASE2B | 2.428489334 | 1.836855555 | 1.78E-35 | 3.81E-34 | Up |
| TMEM132C | 2.427747737 | 1.381975989 | 1.60E-34 | 3.20E-33 | Up |
| NRG3 | 2.420391869 | 1.15409787 | 7.00E-51 | 4.02E-49 | Up |
| SUSD2 | 2.407035369 | 7.465478015 | 1.55E-28 | 2.07E-27 | Up |
| TEKT1 | 2.401491181 | 2.478014341 | 1.88E-15 | 9.97E-15 | Up |
| FAT3 | 2.398312111 | 2.736493014 | 2.95E-37 | 6.99E-36 | Up |
| PLA2G4F | 2.395218852 | 4.971169962 | 3.97E-36 | 8.86E-35 | Up |
| TNR | 2.394210997 | 0.429804914 | 2.73E-62 | 3.64E-60 | Up |
| FPR2 | 2.394033482 | 3.662660221 | 3.33E-44 | 1.23E-42 | Up |
| AGBL1 | 2.390695044 | 0.555444037 | 3.83E-73 | 1.23E-70 | Up |
| PRX | 2.387000289 | 5.530818095 | 6.72E-75 | 2.42E-72 | Up |
| KHDRBS2 | 2.386726205 | 2.145538418 | 3.81E-29 | 5.30E-28 | Up |
| ALPP | 2.385926519 | 2.690334798 | 2.12E-20 | 1.61E-19 | Up |
| OGN | 2.383449427 | 4.056300479 | 3.38E-28 | 4.39E-27 | Up |
| ZNF385B | 2.381393113 | 4.669604054 | 4.18E-23 | 3.80E-22 | Up |
| AFF3 | 2.374394563 | 4.388997994 | 2.96E-37 | 6.99E-36 | Up |
| GRIK4 | 2.369593957 | 1.448342955 | 5.47E-49 | 2.71E-47 | Up |
| RBP4 | 2.366410687 | 4.058564127 | 2.35E-28 | 3.10E-27 | Up |
| MGC42105 | 2.362646866 | 2.547399398 | 3.69E-37 | 8.69E-36 | Up |
| TNXB | 2.354783843 | 6.367859065 | 9.91E-45 | 3.78E-43 | Up |
| WFDC12 | 2.354310313 | 0.850918927 | 8.66E-33 | 1.54E-31 | Up |
| CCBE1 | 2.353713446 | 3.512697369 | 1.46E-31 | 2.42E-30 | Up |
| LAMP3 | 2.353067235 | 7.446681235 | 9.17E-50 | 4.84E-48 | Up |
| GALNT13 | 2.351886233 | 3.040806538 | 1.22E-23 | 1.15E-22 | Up |
| SCN1A | 2.347308837 | 3.17104993 | 1.03E-23 | 9.71E-23 | Up |
| SSTR1 | 2.347264175 | 3.781379956 | 1.71E-30 | 2.60E-29 | Up |
| PLAC2 | 2.343209648 | 3.345437579 | 3.49E-33 | 6.35E-32 | Up |
| HSD17B6 | 2.330767697 | 5.594526623 | 1.30E-37 | 3.17E-36 | Up |
| KAL1 | 2.330733718 | 6.344650181 | 4.35E-55 | 3.26E-53 | Up |
| PCOLCE2 | 2.324243375 | 4.356228478 | 7.36E-31 | 1.15E-29 | Up |
| CCL23 | 2.321247978 | 2.591342649 | 1.81E-34 | 3.60E-33 | Up |
| FHL5 | 2.320716778 | 3.493292586 | 2.46E-50 | 1.36E-48 | Up |

| | | | | | |
|---|---|---|---|---|---|
| TMEM90A | 2.317940882 | 2.170293351 | 2.09E-31 | 3.43E-30 | Up |
| CDO1 | 2.316558415 | 3.318554434 | 5.02E-48 | 2.34E-46 | Up |
| SCN4B | 2.315732522 | 4.608331623 | 2.02E-50 | 1.13E-48 | Up |
| XAGE2 | 2.315211642 | 1.451161382 | 4.35E-18 | 2.79E-17 | Up |
| DCC | 2.313629239 | 1.517426387 | 1.17E-39 | 3.28E-38 | Up |
| SCN7A | 2.30800787 | 4.780820082 | 1.35E-31 | 2.24E-30 | Up |
| C1orf173 | 2.306654936 | 2.877796791 | 1.33E-15 | 7.11E-15 | Up |
| DUSP27 | 2.301295748 | 0.780616683 | 6.89E-40 | 1.94E-38 | Up |
| C8orf85 | 2.298243343 | 2.729384422 | 1.58E-27 | 1.97E-26 | Up |
| FAM189A1 | 2.296743184 | 2.245277671 | 3.95E-36 | 8.82E-35 | Up |
| CAV3 | 2.296195762 | 0.442051651 | 5.63E-67 | 1.09E-64 | Up |
| CYP2F1 | 2.29233214 | 1.395321867 | 1.25E-19 | 8.94E-19 | Up |
| STX11 | 2.288445208 | 5.398364432 | 1.27E-87 | 2.17E-84 | Up |
| DEFA1B | 2.286211182 | 0.643632758 | 4.52E-39 | 1.20E-37 | Up |
| PLAC9 | 2.284252965 | 3.907889736 | 4.36E-49 | 2.18E-47 | Up |
| TMEM190 | 2.280860343 | 2.112683739 | 4.58E-15 | 2.36E-14 | Up |
| RSPO4 | 2.280405108 | 3.227447416 | 5.33E-29 | 7.37E-28 | Up |
| SLC14A1 | 2.279592619 | 3.221909727 | 3.48E-41 | 1.08E-39 | Up |
| KLB | 2.278075736 | 3.12447658 | 1.00E-29 | 1.45E-28 | Up |
| LRRK2 | 2.274270189 | 6.950077312 | 1.09E-27 | 1.38E-26 | Up |
| ASPA | 2.273156728 | 1.733867924 | 4.85E-49 | 2.42E-47 | Up |
| DNAI2 | 2.270304806 | 1.8818418 | 8.19E-17 | 4.76E-16 | Up |
| ALOX15 | 2.269957814 | 3.279789758 | 2.61E-15 | 1.37E-14 | Up |
| FRMD3 | 2.267849189 | 2.654366779 | 2.65E-54 | 1.87E-52 | Up |
| PCSK9 | 2.262052583 | 4.600007903 | 5.59E-21 | 4.41E-20 | Up |
| GPC5 | 2.259126093 | 2.137601096 | 2.32E-24 | 2.30E-23 | Up |
| C1QTNF7 | 2.257831363 | 3.862496724 | 3.55E-36 | 7.93E-35 | Up |
| BTNL8 | 2.257281107 | 1.292099195 | 7.88E-35 | 1.60E-33 | Up |
| CHRDL1 | 2.254082109 | 5.484133998 | 2.69E-28 | 3.54E-27 | Up |
| CTNND2 | 2.253363309 | 2.478678179 | 1.13E-12 | 4.83E-12 | Up |
| MYH2 | 2.251782697 | 0.50064199 | 3.75E-55 | 2.83E-53 | Up |
| FGFR4 | 2.24794387 | 5.289559987 | 2.41E-35 | 5.08E-34 | Up |
| LIN7A | 2.245920105 | 2.254580939 | 6.10E-47 | 2.70E-45 | Up |
| PLA2G3 | 2.244454913 | 2.663604803 | 1.56E-20 | 1.19E-19 | Up |
| ANGPTL1 | 2.243620493 | 3.535521349 | 7.48E-54 | 5.07E-52 | Up |
| LRRC18 | 2.243401571 | 1.538585257 | 1.46E-28 | 1.95E-27 | Up |
| IQSEC3 | 2.239413412 | 2.836330583 | 4.74E-46 | 1.97E-44 | Up |
| SFTA1P | 2.238925069 | 4.96152204 | 4.18E-22 | 3.57E-21 | Up |
| SLC46A2 | 2.238390513 | 4.470339025 | 7.13E-27 | 8.48E-26 | Up |
| ERBB4 | 2.238267387 | 3.424178871 | 1.20E-26 | 1.41E-25 | Up |
| OTC | 2.23751753 | 0.31685763 | 2.08E-80 | 1.29E-77 | Up |
| FAM181A | 2.234697802 | 1.629457845 | 1.95E-24 | 1.95E-23 | Up |
| CCDC85A | 2.232959198 | 4.152956233 | 9.65E-53 | 6.06E-51 | Up |
| KL | 2.231918567 | 3.945135312 | 1.43E-47 | 6.47E-46 | Up |
| C8orf84 | 2.231217079 | 3.918700337 | 5.73E-56 | 4.64E-54 | Up |
| PCSK2 | 2.229437828 | 2.802973228 | 1.55E-09 | 5.08E-09 | Up |

| | | | | | |
|---|---|---|---|---|---|
| FAM46B | 2.22795138 | 4.665704725 | 3.50E-58 | 3.33E-56 | Up |
| HMGCS2 | 2.221384797 | 1.934972948 | 1.34E-13 | 6.19E-13 | Up |
| CYP17A1 | 2.219346029 | 0.951262219 | 1.83E-35 | 3.89E-34 | Up |
| ACOXL | 2.213163864 | 3.470063023 | 3.99E-29 | 5.55E-28 | Up |
| BCHE | 2.212454688 | 3.554254913 | 1.60E-38 | 4.11E-37 | Up |
| DACH1 | 2.208973026 | 3.910777265 | 6.97E-48 | 3.22E-46 | Up |
| RAMP3 | 2.206775433 | 5.619581312 | 2.14E-76 | 8.80E-74 | Up |
| BMX | 2.201126012 | 2.225640864 | 5.96E-34 | 1.14E-32 | Up |
| LPL | 2.199029087 | 6.487573364 | 2.58E-32 | 4.48E-31 | Up |
| SCGB3A2 | 2.197990438 | 7.016274437 | 1.45E-10 | 5.17E-10 | Up |
| KCNK3 | 2.197000162 | 4.853425627 | 3.30E-32 | 5.66E-31 | Up |
| KRT79 | 2.195946896 | 0.676865312 | 6.99E-42 | 2.27E-40 | Up |
| PZP | 2.194854354 | 1.636671916 | 1.13E-19 | 8.11E-19 | Up |
| RNF182 | 2.191117081 | 2.72120164 | 2.18E-37 | 5.19E-36 | Up |
| ATP1A2 | 2.189160609 | 3.201569337 | 2.71E-29 | 3.80E-28 | Up |
| DMBT1 | 2.187901847 | 6.836370056 | 7.16E-12 | 2.88E-11 | Up |
| SEMA3G | 2.187541655 | 5.117931338 | 1.06E-71 | 2.99E-69 | Up |
| FBN3 | 2.185767927 | 2.799913794 | 2.24E-16 | 1.27E-15 | Up |
| NPR1 | 2.184428189 | 5.362627407 | 8.50E-55 | 6.19E-53 | Up |
| STOML3 | 2.183554861 | 2.033010647 | 1.25E-16 | 7.21E-16 | Up |
| CCRL1 | 2.182676621 | 3.620549688 | 5.46E-50 | 2.91E-48 | Up |
| MFAP4 | 2.176261911 | 7.296697552 | 1.53E-42 | 5.11E-41 | Up |
| C6 | 2.175548687 | 2.950896073 | 2.82E-16 | 1.59E-15 | Up |
| CHST9 | 2.17384044 | 2.855037306 | 6.72E-12 | 2.70E-11 | Up |
| ANKRD29 | 2.170619748 | 4.9176893 | 6.55E-34 | 1.25E-32 | Up |
| MAP3K15 | 2.170363924 | 1.215198769 | 9.78E-28 | 1.24E-26 | Up |
| EFCAB1 | 2.168953304 | 3.136282512 | 3.45E-17 | 2.06E-16 | Up |
| CXCR1 | 2.168592132 | 2.478644395 | 2.53E-33 | 4.65E-32 | Up |
| ABI3BP | 2.166074046 | 5.969867013 | 4.87E-41 | 1.49E-39 | Up |
| DCDC2B | 2.165242187 | 1.370422301 | 1.11E-18 | 7.45E-18 | Up |
| HBG1 | 2.16500035 | 0.491560757 | 3.40E-45 | 1.36E-43 | Up |
| GSTA3 | 2.164742077 | 0.644879229 | 1.94E-37 | 4.65E-36 | Up |
| C1orf92 | 2.161268704 | 1.435561756 | 6.06E-18 | 3.84E-17 | Up |
| LOC100287718 | 2.156139374 | 1.157411853 | 1.66E-21 | 1.36E-20 | Up |
| C9orf24 | 2.154739502 | 3.075453962 | 8.92E-17 | 5.17E-16 | Up |
| HPSE2 | 2.154371707 | 1.049719838 | 4.47E-39 | 1.19E-37 | Up |
| VSIG2 | 2.152963443 | 5.221515458 | 4.20E-17 | 2.49E-16 | Up |
| VWA3A | 2.150764028 | 2.230059578 | 2.62E-17 | 1.59E-16 | Up |
| ABCC13 | 2.14962445 | 1.007886723 | 5.13E-34 | 9.91E-33 | Up |
| WDR16 | 2.146857214 | 2.292717044 | 3.40E-17 | 2.03E-16 | Up |
| TUBA4B | 2.141085372 | 2.191395986 | 1.23E-16 | 7.07E-16 | Up |
| GPC3 | 2.132786284 | 5.698064171 | 3.31E-34 | 6.48E-33 | Up |
| KCNE1 | 2.130514209 | 2.990853559 | 4.26E-27 | 5.16E-26 | Up |
| ERVFRDE1 | 2.130339352 | 1.245984284 | 1.10E-48 | 5.31E-47 | Up |
| RADIL | 2.129703157 | 3.552493637 | 1.29E-39 | 3.60E-38 | Up |
| CDHR3 | 2.124931778 | 4.126360977 | 9.56E-15 | 4.81E-14 | Up |

| | | | | | |
|---|---|---|---|---|---|
| CACNA1S | 2.123939949 | 0.261410569 | 5.49E-61 | 6.55E-59 | Up |
| CES4 | 2.123127533 | 2.832861928 | 4.64E-21 | 3.68E-20 | Up |
| CWH43 | 2.122243792 | 1.079784573 | 1.83E-21 | 1.49E-20 | Up |
| EMCN | 2.120700529 | 5.400310341 | 7.48E-60 | 8.04E-58 | Up |
| TGM1 | 2.120574282 | 3.265256912 | 2.55E-54 | 1.81E-52 | Up |
| CHRM2 | 2.117011216 | 0.310126389 | 5.68E-70 | 1.39E-67 | Up |
| RIMS4 | 2.113563858 | 2.552945235 | 6.47E-25 | 6.66E-24 | Up |
| OSTBETA | 2.112623417 | 1.725084048 | 1.04E-21 | 8.63E-21 | Up |
| TMEM139 | 2.112108877 | 4.308980985 | 1.78E-25 | 1.92E-24 | Up |
| C9orf171 | 2.110868776 | 1.903185345 | 2.11E-16 | 1.20E-15 | Up |
| SEMA5A | 2.110350102 | 5.946347425 | 6.88E-54 | 4.69E-52 | Up |
| HSPB6 | 2.109244655 | 5.247497043 | 3.14E-46 | 1.34E-44 | Up |
| PIP5K1B | 2.108512408 | 4.677116952 | 1.58E-39 | 4.37E-38 | Up |
| MT1M | 2.107763049 | 3.57599377 | 4.97E-29 | 6.87E-28 | Up |
| MS4A8B | 2.107484357 | 3.293844569 | 3.75E-11 | 1.41E-10 | Up |
| NTRK2 | 2.107465188 | 3.976746985 | 1.41E-26 | 1.65E-25 | Up |
| CAPSL | 2.106347344 | 2.226283352 | 3.68E-14 | 1.77E-13 | Up |
| C14orf132 | 2.105300184 | 5.691363624 | 1.38E-59 | 1.40E-57 | Up |
| SYNPO2L | 2.103039698 | 1.559535559 | 7.11E-42 | 2.30E-40 | Up |
| TGFBR3 | 2.102978 | 5.610294938 | 4.75E-58 | 4.43E-56 | Up |
| CNKSR2 | 2.096845017 | 1.77430588 | 3.01E-32 | 5.18E-31 | Up |
| ITGA8 | 2.096618267 | 4.212366903 | 4.14E-28 | 5.35E-27 | Up |
| LRRC2 | 2.095644794 | 2.626132385 | 6.08E-42 | 1.98E-40 | Up |
| RGS9 | 2.092335507 | 2.822550456 | 1.84E-48 | 8.77E-47 | Up |
| JPH4 | 2.091769306 | 3.001982195 | 8.29E-58 | 7.63E-56 | Up |
| HIGD1B | 2.091184042 | 3.441092597 | 1.36E-50 | 7.69E-49 | Up |
| C15orf26 | 2.090206938 | 1.372576233 | 4.55E-24 | 4.43E-23 | Up |
| C6orf155 | 2.088849615 | 1.902342618 | 5.25E-48 | 2.44E-46 | Up |
| ADAMTSL3 | 2.087856171 | 4.555428571 | 5.45E-43 | 1.87E-41 | Up |
| SGEF | 2.086845613 | 5.659223921 | 4.67E-67 | 9.14E-65 | Up |
| DUOXA1 | 2.084253116 | 4.803047014 | 1.88E-25 | 2.02E-24 | Up |
| TAL1 | 2.082337488 | 3.924710369 | 1.02E-74 | 3.61E-72 | Up |
| SOX7 | 2.081869377 | 4.821734283 | 1.30E-60 | 1.50E-58 | Up |
| GLDN | 2.078182779 | 4.5284717 | 6.45E-39 | 1.69E-37 | Up |
| CASP12 | 2.076710163 | 0.714657138 | 3.01E-53 | 1.95E-51 | Up |
| CCDC48 | 2.076399787 | 4.182601942 | 1.37E-50 | 7.73E-49 | Up |
| ART4 | 2.075624807 | 1.963161124 | 2.18E-34 | 4.32E-33 | Up |
| PKNOX2 | 2.072934012 | 3.95604987 | 2.63E-43 | 9.21E-42 | Up |
| SCGB3A1 | 2.071497271 | 6.568940025 | 7.49E-10 | 2.52E-09 | Up |
| NMUR1 | 2.070632756 | 2.922489464 | 1.77E-50 | 9.92E-49 | Up |
| APOA1 | 2.07005628 | 1.079339324 | 8.97E-34 | 1.70E-32 | Up |
| OLR1 | 2.069231108 | 6.239781531 | 1.00E-41 | 3.22E-40 | Up |
| RGS22 | 2.06881278 | 2.478865963 | 1.59E-21 | 1.30E-20 | Up |
| PCDH9 | 2.067844783 | 3.254595592 | 4.84E-31 | 7.67E-30 | Up |
| PRSS35 | 2.063070183 | 2.260047912 | 2.05E-38 | 5.25E-37 | Up |
| LIMS2 | 2.062823391 | 5.789071035 | 6.76E-77 | 3.15E-74 | Up |

| | | | | | |
|---|---|---|---|---|---|
| CA3 | 2.062568393 | 3.377578348 | 3.10E-25 | 3.28E-24 | Up |
| TTC29 | 2.059990637 | 1.461290267 | 4.59E-16 | 2.54E-15 | Up |
| MRC1 | 2.05998662 | 7.082495101 | 5.68E-36 | 1.25E-34 | Up |
| WDR38 | 2.059767395 | 2.316035396 | 2.95E-13 | 1.32E-12 | Up |
| C6orf174 | 2.059736313 | 4.486888117 | 5.02E-36 | 1.11E-34 | Up |
| C13orf15 | 2.059518561 | 6.838068744 | 9.07E-84 | 8.87E-81 | Up |
| TPPP3 | 2.056156655 | 6.376262071 | 1.71E-35 | 3.68E-34 | Up |
| MORN5 | 2.05139628 | 1.779382767 | 9.71E-16 | 5.26E-15 | Up |
| RAMP2 | 2.047355479 | 5.647307612 | 8.33E-84 | 8.55E-81 | Up |
| GPR120 | 2.046738266 | 2.429749718 | 4.21E-30 | 6.23E-29 | Up |
| KANK3 | 2.045871917 | 4.6801662 | 7.73E-79 | 4.41E-76 | Up |
| S1PR1 | 2.045435465 | 6.239537453 | 7.77E-83 | 6.65E-80 | Up |
| AOC3 | 2.043288601 | 7.065938183 | 5.64E-55 | 4.21E-53 | Up |
| CCL14 | 2.041945641 | 4.956112122 | 1.27E-30 | 1.94E-29 | Up |
| CLDN5 | 2.038666466 | 6.127298516 | 1.12E-54 | 8.13E-53 | Up |
| PPP4R4 | 2.037900181 | 2.571250048 | 9.62E-25 | 9.79E-24 | Up |
| SEMA6A | 2.037268022 | 4.968810397 | 3.58E-61 | 4.33E-59 | Up |
| ROBO4 | 2.036145056 | 6.084417212 | 1.15E-76 | 5.14E-74 | Up |
| CALCRL | 2.035721369 | 6.414002814 | 2.85E-71 | 7.92E-69 | Up |
| HSPA12B | 2.034463541 | 4.743316754 | 2.75E-76 | 1.08E-73 | Up |
| SLC5A7 | 2.032643681 | 0.756679929 | 1.01E-29 | 1.46E-28 | Up |
| P2RX2 | 2.03094731 | 1.029533988 | 3.84E-30 | 5.71E-29 | Up |
| DARC | 2.028004504 | 5.152127834 | 2.91E-24 | 2.87E-23 | Up |
| SLIT2 | 2.026936868 | 5.423766319 | 6.29E-40 | 1.77E-38 | Up |
| EMP2 | 2.02362943 | 8.599805773 | 9.29E-82 | 7.07E-79 | Up |
| CXCR2 | 2.02181755 | 3.295048793 | 3.53E-39 | 9.50E-38 | Up |
| STAC | 2.021220703 | 4.500495768 | 1.18E-27 | 1.48E-26 | Up |
| AKAP2 | 2.020368483 | 6.643591543 | 2.87E-65 | 4.83E-63 | Up |
| SIGLEC11 | 2.018799465 | 2.855420668 | 8.11E-40 | 2.28E-38 | Up |
| LEPREL1 | 2.016491899 | 5.909060703 | 1.69E-28 | 2.25E-27 | Up |
| BAI3 | 2.015978857 | 1.958702405 | 7.13E-31 | 1.11E-29 | Up |
| C6orf118 | 2.015581866 | 1.593589729 | 1.90E-16 | 1.08E-15 | Up |
| WFIKKN2 | 2.013896114 | 1.027551728 | 9.91E-33 | 1.76E-31 | Up |
| TMEM232 | 2.010855109 | 2.167423603 | 8.97E-21 | 6.98E-20 | Up |
| C1orf158 | 2.010436515 | 1.374715862 | 9.67E-18 | 6.03E-17 | Up |
| C1orf129 | 2.009954714 | 0.872668424 | 1.79E-25 | 1.93E-24 | Up |
| PACRG | 2.006752706 | 1.997466095 | 4.42E-21 | 3.50E-20 | Up |
| SLC4A1 | 2.006510579 | 0.426022174 | 3.93E-53 | 2.54E-51 | Up |
| HSPB3 | 2.005596076 | 0.995330314 | 2.85E-40 | 8.23E-39 | Up |
| PTPRB | 2.00219304 | 6.635759182 | 3.96E-69 | 9.03E-67 | Up |
| GSTM5 | 2.001210975 | 3.963225625 | 1.69E-46 | 7.28E-45 | Up |
| CST5 | 2.000310288 | 1.388611781 | 1.16E-25 | 1.27E-24 | Up |
| GGTLC1 | 1.999282056 | 4.726809117 | 2.78E-15 | 1.46E-14 | Up |
| PDZD2 | 1.996362559 | 5.954792777 | 3.71E-41 | 1.15E-39 | Up |
| C6orf105 | 1.994934734 | 3.966084401 | 3.06E-36 | 6.90E-35 | Up |
| C6orf103 | 1.993568948 | 1.175692474 | 2.94E-19 | 2.05E-18 | Up |

| | | | | | |
|---|---|---|---|---|---|
| OR2W3 | 1.991921059 | 0.347077726 | 1.80E-54 | 1.29E-52 | Up |
| UNC45B | 1.991193005 | 1.256146857 | 1.37E-43 | 4.95E-42 | Up |
| CD101 | 1.991190944 | 4.316085018 | 1.83E-61 | 2.31E-59 | Up |
| AGTR1 | 1.9908047 | 3.266307256 | 2.13E-30 | 3.20E-29 | Up |
| CDHR4 | 1.989647903 | 2.171550554 | 2.20E-12 | 9.21E-12 | Up |
| TMEM146 | 1.989578409 | 1.035330559 | 1.03E-21 | 8.57E-21 | Up |
| PHACTR1 | 1.989007652 | 3.951580621 | 4.71E-45 | 1.87E-43 | Up |
| LOC150622 | 1.986616126 | 1.276940355 | 1.31E-21 | 1.08E-20 | Up |
| SH2D4B | 1.98432846 | 1.248642633 | 1.07E-46 | 4.70E-45 | Up |
| ANGPTL5 | 1.983766724 | 0.618702248 | 1.18E-44 | 4.48E-43 | Up |
| ITIH5 | 1.982924659 | 5.521123192 | 3.69E-34 | 7.19E-33 | Up |
| SIRPB1 | 1.979122629 | 4.884968258 | 8.49E-48 | 3.89E-46 | Up |
| PDK4 | 1.977130021 | 6.555994424 | 1.05E-38 | 2.72E-37 | Up |
| LEFTY2 | 1.975233303 | 2.43480165 | 2.99E-23 | 2.74E-22 | Up |
| TMEM212 | 1.972853941 | 1.215173356 | 9.39E-19 | 6.31E-18 | Up |
| PREX2 | 1.971773608 | 3.202321148 | 1.35E-28 | 1.82E-27 | Up |
| FOXF1 | 1.966598028 | 5.064352426 | 5.49E-68 | 1.20E-65 | Up |
| HYAL1 | 1.964913202 | 5.499396266 | 5.78E-32 | 9.79E-31 | Up |
| C10orf107 | 1.962493816 | 2.519713503 | 1.10E-17 | 6.82E-17 | Up |
| SULT1C4 | 1.960710942 | 3.185348735 | 1.78E-41 | 5.62E-40 | Up |
| SNTN | 1.959592334 | 3.607854584 | 3.04E-14 | 1.47E-13 | Up |
| PRIMA1 | 1.958490286 | 1.845604539 | 1.74E-21 | 1.42E-20 | Up |
| SLC1A1 | 1.957760331 | 5.685691482 | 9.88E-37 | 2.27E-35 | Up |
| ABCA3 | 1.957227569 | 8.172641142 | 5.25E-27 | 6.30E-26 | Up |
| ANGPT1 | 1.953055343 | 5.202381797 | 3.09E-40 | 8.91E-39 | Up |
| C1orf194 | 1.952555691 | 2.413949145 | 2.49E-13 | 1.12E-12 | Up |
| PADI4 | 1.952314218 | 0.919976337 | 2.43E-39 | 6.62E-38 | Up |
| TRIM71 | 1.951892005 | 0.658709073 | 6.78E-35 | 1.38E-33 | Up |
| APOBEC4 | 1.951299974 | 1.685798582 | 8.34E-16 | 4.54E-15 | Up |
| PSAPL1 | 1.950732486 | 1.20989622 | 8.19E-20 | 5.95E-19 | Up |
| SLC39A8 | 1.949704016 | 7.773743094 | 8.57E-67 | 1.63E-64 | Up |
| SEMA6D | 1.94563883 | 4.338264525 | 1.75E-40 | 5.16E-39 | Up |
| C7 | 1.944858428 | 6.872921881 | 2.96E-19 | 2.07E-18 | Up |
| RANBP3L | 1.944765649 | 1.877548816 | 1.47E-28 | 1.97E-27 | Up |
| CFD | 1.94221086 | 5.956242635 | 3.50E-39 | 9.44E-38 | Up |
| CES1 | 1.941553236 | 7.057170376 | 3.66E-21 | 2.92E-20 | Up |
| IL33 | 1.940180491 | 5.97069623 | 1.53E-36 | 3.49E-35 | Up |
| CAV2 | 1.938738101 | 7.365614409 | 1.61E-51 | 9.66E-50 | Up |
| CEACAM8 | 1.937587952 | 0.838771394 | 1.82E-19 | 1.29E-18 | Up |
| MYOCD | 1.93576354 | 2.71301098 | 1.67E-24 | 1.68E-23 | Up |
| INSC | 1.932578379 | 1.01089205 | 7.00E-26 | 7.81E-25 | Up |
| ADRA1D | 1.930920334 | 1.78847461 | 5.53E-34 | 1.06E-32 | Up |
| PCYT1B | 1.930389031 | 2.416252251 | 1.68E-26 | 1.95E-25 | Up |
| PTPRT | 1.92839465 | 2.60758356 | 2.72E-12 | 1.13E-11 | Up |
| ARHGAP6 | 1.927749479 | 4.236361576 | 1.23E-63 | 1.88E-61 | Up |
| TSPAN7 | 1.926485295 | 5.981546943 | 2.25E-29 | 3.18E-28 | Up |

| | | | | | |
|---|---|---|---|---|---|
| RP1 | 1.926476756 | 3.005412156 | 1.53E-15 | 8.15E-15 | Up |
| ZBED2 | 1.922457856 | 4.215403461 | 1.01E-33 | 1.90E-32 | Up |
| C20orf202 | 1.921539818 | 1.786887926 | 2.47E-64 | 3.97E-62 | Up |
| IL1A | 1.920764588 | 2.471990582 | 4.41E-21 | 3.50E-20 | Up |
| EYA4 | 1.920461364 | 3.07498314 | 1.13E-21 | 9.40E-21 | Up |
| LOC158376 | 1.919707481 | 2.863865678 | 3.04E-82 | 2.40E-79 | Up |
| GIPC2 | 1.915292417 | 3.381717874 | 2.47E-31 | 4.02E-30 | Up |
| LOXHD1 | 1.914090722 | 1.419427651 | 4.91E-41 | 1.50E-39 | Up |
| LOC283392 | 1.91340015 | 0.60508174 | 2.67E-34 | 5.25E-33 | Up |
| ATP13A4 | 1.912474685 | 5.674105311 | 2.74E-15 | 1.44E-14 | Up |
| HBG2 | 1.9115732 | 0.291994711 | 3.35E-41 | 1.04E-39 | Up |
| FGF10 | 1.909961568 | 0.227303231 | 9.06E-73 | 2.82E-70 | Up |
| LDB2 | 1.908440183 | 5.682382107 | 1.75E-80 | 1.12E-77 | Up |
| SELP | 1.90765597 | 4.759831668 | 4.93E-38 | 1.23E-36 | Up |
| ST6GALNAC5 | 1.907455951 | 4.714543776 | 1.50E-41 | 4.77E-40 | Up |
| VSIG4 | 1.906747327 | 6.720015525 | 9.42E-39 | 2.45E-37 | Up |
| SOX17 | 1.902384129 | 3.768297113 | 3.45E-58 | 3.30E-56 | Up |
| C20orf160 | 1.901847621 | 4.104092842 | 3.50E-70 | 8.75E-68 | Up |
| S100A12 | 1.899608215 | 1.580426126 | 8.53E-26 | 9.47E-25 | Up |
| SLIT3 | 1.896966531 | 5.812846835 | 5.10E-34 | 9.88E-33 | Up |
| ODF3L1 | 1.893718856 | 1.999992348 | 1.30E-35 | 2.80E-34 | Up |
| CFTR | 1.892980198 | 5.043027052 | 2.17E-14 | 1.06E-13 | Up |
| PTGFR | 1.892811884 | 3.43914484 | 9.16E-24 | 8.70E-23 | Up |
| MMRN1 | 1.89275859 | 4.957557953 | 3.40E-33 | 6.21E-32 | Up |
| AVPR2 | 1.891870146 | 2.1054065 | 1.77E-35 | 3.78E-34 | Up |
| GPR17 | 1.890565127 | 1.725984702 | 2.01E-32 | 3.49E-31 | Up |
| TTLL10 | 1.888952877 | 2.004652753 | 1.21E-18 | 8.08E-18 | Up |
| ROBO2 | 1.888529534 | 4.178417506 | 3.01E-22 | 2.60E-21 | Up |
| CXorf41 | 1.888099768 | 1.191906792 | 5.43E-17 | 3.20E-16 | Up |
| PTCRA | 1.885632654 | 1.788465374 | 1.64E-36 | 3.75E-35 | Up |
| C11orf9 | 1.880219633 | 6.098350536 | 9.32E-26 | 1.03E-24 | Up |
| TCTE1 | 1.879909174 | 1.252740525 | 3.65E-18 | 2.36E-17 | Up |
| CCDC60 | 1.877171006 | 1.455483101 | 6.25E-16 | 3.43E-15 | Up |
| HSD17B13 | 1.874473152 | 2.716407603 | 6.95E-18 | 4.38E-17 | Up |
| SGCA | 1.873973892 | 3.938448318 | 6.70E-27 | 7.99E-26 | Up |
| EPAS1 | 1.872772138 | 9.026133967 | 3.51E-88 | 6.54E-85 | Up |
| ATOH8 | 1.870060655 | 5.988266078 | 1.37E-21 | 1.12E-20 | Up |
| TUBB1 | 1.86929468 | 2.937325134 | 1.05E-50 | 5.96E-49 | Up |
| NXPH3 | 1.868684361 | 3.888588096 | 1.29E-51 | 7.76E-50 | Up |
| HBEGF | 1.867644352 | 6.178061025 | 2.74E-53 | 1.78E-51 | Up |
| MUSK | 1.864132312 | 0.790950267 | 5.46E-36 | 1.20E-34 | Up |
| LRRTM4 | 1.862943993 | 0.984499808 | 4.59E-31 | 7.31E-30 | Up |
| GFRA1 | 1.862821398 | 3.924754696 | 3.76E-19 | 2.60E-18 | Up |
| USHBP1 | 1.861832345 | 3.694584754 | 1.18E-60 | 1.37E-58 | Up |
| TMC2 | 1.861506404 | 0.793449127 | 1.09E-31 | 1.81E-30 | Up |
| CMTM5 | 1.858146615 | 0.208771401 | 2.81E-69 | 6.49E-67 | Up |

| | | | | | |
|---|---|---|---|---|---|
| MSR1 | 1.857025092 | 6.964226372 | 3.22E-41 | 9.99E-40 | Up |
| ANO2 | 1.856396047 | 1.902892359 | 1.50E-38 | 3.89E-37 | Up |
| TBX4 | 1.855190836 | 5.046654931 | 1.56E-33 | 2.91E-32 | Up |
| MS4A2 | 1.850645279 | 4.093110721 | 3.31E-24 | 3.26E-23 | Up |
| DPEP2 | 1.849489392 | 4.399383871 | 1.21E-47 | 5.48E-46 | Up |
| GRK5 | 1.848246344 | 5.926573626 | 1.55E-70 | 3.92E-68 | Up |
| DLC1 | 1.846044416 | 7.214570461 | 8.48E-42 | 2.72E-40 | Up |
| SVEP1 | 1.844694173 | 5.82869954 | 2.10E-35 | 4.45E-34 | Up |
| HMGCLL1 | 1.844644831 | 2.546893162 | 4.30E-28 | 5.55E-27 | Up |
| FCGR3B | 1.841382009 | 3.685398042 | 1.96E-24 | 1.96E-23 | Up |
| C4orf31 | 1.837962541 | 6.854696191 | 4.21E-22 | 3.60E-21 | Up |
| COX4I2 | 1.837735939 | 4.051392971 | 3.34E-43 | 1.16E-41 | Up |
| GPR123 | 1.836842453 | 0.658744575 | 1.57E-28 | 2.09E-27 | Up |
| CPAMD8 | 1.836755061 | 5.794140912 | 1.16E-21 | 9.63E-21 | Up |
| AQP1 | 1.835774701 | 8.736086568 | 9.11E-25 | 9.28E-24 | Up |
| SPAG6 | 1.834773213 | 3.483466951 | 5.36E-10 | 1.83E-09 | Up |
| RPH3A | 1.833596107 | 0.873200101 | 2.33E-35 | 4.92E-34 | Up |
| MYO7B | 1.833323683 | 2.465855514 | 6.32E-24 | 6.08E-23 | Up |
| CDH5 | 1.833163387 | 6.889487317 | 5.01E-76 | 1.91E-73 | Up |
| REEP1 | 1.831731366 | 3.665242507 | 6.64E-29 | 9.10E-28 | Up |
| CORO2B | 1.830525215 | 3.934446471 | 5.90E-43 | 2.02E-41 | Up |
| GPRIN2 | 1.83004308 | 5.555418064 | 3.13E-28 | 4.09E-27 | Up |
| PDE1C | 1.828383517 | 2.569380283 | 7.24E-23 | 6.48E-22 | Up |
| MMP28 | 1.827664697 | 5.581417815 | 8.73E-17 | 5.06E-16 | Up |
| AWAT2 | 1.826798793 | 0.531130195 | 1.15E-34 | 2.32E-33 | Up |
| GIMAP8 | 1.824842994 | 5.819835922 | 4.11E-67 | 8.12E-65 | Up |
| GHR | 1.824662666 | 3.549884379 | 1.32E-42 | 4.45E-41 | Up |
| JAM2 | 1.824138559 | 4.636949795 | 1.45E-67 | 2.97E-65 | Up |
| CCDC108 | 1.822458667 | 2.925413528 | 6.12E-14 | 2.90E-13 | Up |
| ALDH1A2 | 1.82160723 | 4.293212141 | 5.52E-18 | 3.52E-17 | Up |
| AKAP14 | 1.821151911 | 1.245987359 | 3.04E-15 | 1.59E-14 | Up |
| THBD | 1.81958876 | 6.726966581 | 5.87E-56 | 4.73E-54 | Up |
| FAM167A | 1.819418295 | 5.181000228 | 7.25E-27 | 8.62E-26 | Up |
| COL29A1 | 1.81936939 | 3.907586232 | 2.64E-20 | 1.99E-19 | Up |
| MYOZ1 | 1.816575355 | 3.120037588 | 1.49E-24 | 1.50E-23 | Up |
| PTPN21 | 1.815884514 | 5.942343708 | 1.53E-76 | 6.66E-74 | Up |
| ACVRL1 | 1.8132698 | 6.558538418 | 7.77E-86 | 1.06E-82 | Up |
| CLIC3 | 1.812696676 | 5.483601604 | 1.31E-26 | 1.53E-25 | Up |
| CACNG6 | 1.812557857 | 1.7967596 | 5.59E-13 | 2.46E-12 | Up |
| DYNLRB2 | 1.81242317 | 1.873633756 | 5.64E-16 | 3.11E-15 | Up |
| PDZRN4 | 1.812054919 | 1.537374197 | 6.05E-25 | 6.23E-24 | Up |
| RGS6 | 1.811678585 | 1.657747836 | 3.71E-17 | 2.22E-16 | Up |
| SLC24A4 | 1.810313905 | 2.198953363 | 4.31E-37 | 1.01E-35 | Up |
| PF4 | 1.809361119 | 0.811842047 | 3.72E-27 | 4.52E-26 | Up |
| GPX3 | 1.808883816 | 8.243007807 | 3.32E-50 | 1.82E-48 | Up |
| BEX1 | 1.8058494 | 1.589373876 | 5.37E-15 | 2.74E-14 | Up |

| | | | | | |
|---|---|---|---|---|---|
| MFAP3L | 1.80418557 | 4.157086065 | 4.18E-42 | 1.37E-40 | Up |
| KLF4 | 1.803218915 | 6.367068156 | 1.80E-40 | 5.28E-39 | Up |
| AATK | 1.799336793 | 5.046167813 | 3.37E-27 | 4.11E-26 | Up |
| KLK11 | 1.797876189 | 4.8834279 | 5.42E-09 | 1.69E-08 | Up |
| CMTM2 | 1.797171009 | 1.436602655 | 2.89E-42 | 9.54E-41 | Up |
| ARHGEF15 | 1.796724234 | 5.307585035 | 3.08E-64 | 4.90E-62 | Up |
| RSPH4A | 1.795182351 | 3.465322934 | 1.83E-16 | 1.04E-15 | Up |
| DNAI1 | 1.794418389 | 2.282545664 | 7.78E-12 | 3.11E-11 | Up |
| KRT27 | 1.793699308 | 0.744210386 | 2.65E-30 | 3.96E-29 | Up |
| CLEC12A | 1.793232433 | 3.992802031 | 8.07E-28 | 1.03E-26 | Up |
| NCRNA00162 | 1.792416612 | 0.534526166 | 3.65E-25 | 3.84E-24 | Up |
| SLC5A4 | 1.79036968 | 0.876708992 | 5.77E-39 | 1.52E-37 | Up |
| KLK10 | 1.789888118 | 4.591398524 | 5.51E-11 | 2.04E-10 | Up |
| C20orf56 | 1.787664075 | 4.830712898 | 9.59E-12 | 3.81E-11 | Up |
| MEGF11 | 1.787354803 | 2.077928813 | 5.26E-13 | 2.32E-12 | Up |
| IL5RA | 1.786707489 | 1.945547854 | 4.84E-20 | 3.57E-19 | Up |
| GLT25D2 | 1.784828995 | 3.403692918 | 5.33E-27 | 6.39E-26 | Up |
| FAM162B | 1.784395742 | 3.579757022 | 2.55E-42 | 8.45E-41 | Up |
| PALM2-AKAP2 | 1.783471999 | 6.980695042 | 1.47E-78 | 7.94E-76 | Up |
| DNAH12 | 1.782752531 | 2.282184918 | 2.21E-12 | 9.23E-12 | Up |
| SCN2B | 1.779967007 | 2.898457654 | 1.77E-26 | 2.05E-25 | Up |
| ROPN1L | 1.779521416 | 2.798972777 | 2.62E-14 | 1.28E-13 | Up |
| NTRK3 | 1.778785344 | 2.133012079 | 5.42E-27 | 6.50E-26 | Up |
| PRAM1 | 1.776046055 | 4.365139108 | 1.55E-38 | 3.99E-37 | Up |
| DKK2 | 1.774102732 | 4.234832729 | 5.56E-38 | 1.38E-36 | Up |
| L1TD1 | 1.773670018 | 2.109247785 | 2.03E-27 | 2.51E-26 | Up |
| C7orf58 | 1.770856015 | 5.09956304 | 3.47E-42 | 1.14E-40 | Up |
| NRXN1 | 1.766299941 | 0.87430191 | 3.77E-22 | 3.23E-21 | Up |
| NDRG4 | 1.763365908 | 4.819716173 | 4.69E-34 | 9.10E-33 | Up |
| PENK | 1.761884678 | 2.443544044 | 2.44E-13 | 1.10E-12 | Up |
| PTH1R | 1.761109098 | 3.47766352 | 2.10E-34 | 4.16E-33 | Up |
| CYP1A1 | 1.760965165 | 0.757981638 | 1.15E-12 | 4.93E-12 | Up |
| KCNIP1 | 1.759770049 | 1.232703564 | 2.00E-33 | 3.70E-32 | Up |
| SPN | 1.758664341 | 5.981276146 | 2.73E-45 | 1.10E-43 | Up |
| FGF2 | 1.757607676 | 4.347226479 | 2.39E-43 | 8.41E-42 | Up |
| VWF | 1.754685472 | 8.260326863 | 5.57E-46 | 2.30E-44 | Up |
| CGNL1 | 1.753355849 | 6.567662683 | 3.18E-51 | 1.85E-49 | Up |
| SLC5A1 | 1.753032141 | 3.748805618 | 2.16E-09 | 7.00E-09 | Up |
| FAM92B | 1.752763012 | 2.76841553 | 5.01E-13 | 2.21E-12 | Up |
| GRASP | 1.748635411 | 4.906781619 | 8.40E-59 | 8.25E-57 | Up |
| C1orf150 | 1.747679865 | 1.707490569 | 4.69E-24 | 4.56E-23 | Up |
| CD52 | 1.747613066 | 6.618795193 | 1.52E-42 | 5.07E-41 | Up |
| ZBBX | 1.746093896 | 2.02631719 | 2.24E-11 | 8.61E-11 | Up |
| GPM6B | 1.744786279 | 4.692857822 | 1.45E-42 | 4.88E-41 | Up |
| C2orf39 | 1.744693074 | 2.759956738 | 9.00E-11 | 3.27E-10 | Up |
| KCNJ16 | 1.743617091 | 1.988515899 | 1.81E-13 | 8.29E-13 | Up |

| | | | | | |
|---|---|---|---|---|---|
| SLC16A11 | 1.743323854 | 3.357998926 | 4.16E-27 | 5.05E-26 | Up |
| FIBIN | 1.742996972 | 5.06590028 | 1.45E-35 | 3.12E-34 | Up |
| GPER | 1.742452364 | 3.922501827 | 1.12E-35 | 2.42E-34 | Up |
| DCDC2 | 1.73935731 | 4.101852726 | 3.84E-16 | 2.14E-15 | Up |
| LOC100190938 | 1.738423697 | 2.772657829 | 9.52E-33 | 1.69E-31 | Up |
| RASIP1 | 1.736740948 | 5.299038834 | 2.39E-65 | 4.06E-63 | Up |
| CFP | 1.73568464 | 4.094494002 | 1.54E-41 | 4.88E-40 | Up |
| TSLP | 1.734509647 | 2.154095649 | 1.22E-25 | 1.33E-24 | Up |
| KANK4 | 1.734226949 | 3.733266161 | 4.72E-22 | 4.02E-21 | Up |
| KCNJ5 | 1.734007648 | 3.913501761 | 4.73E-26 | 5.34E-25 | Up |
| NR4A3 | 1.733607286 | 5.148484115 | 4.54E-27 | 5.48E-26 | Up |
| SMAD6 | 1.729857676 | 5.042147204 | 1.11E-55 | 8.75E-54 | Up |
| PDE8B | 1.725942921 | 3.411786963 | 4.45E-37 | 1.04E-35 | Up |
| PGM5 | 1.725494753 | 4.991537183 | 1.08E-31 | 1.80E-30 | Up |
| IL6 | 1.723321715 | 4.570701584 | 6.86E-22 | 5.78E-21 | Up |
| WISP2 | 1.723215284 | 5.091187286 | 1.03E-30 | 1.58E-29 | Up |
| ZNF536 | 1.723205929 | 0.415332344 | 2.83E-39 | 7.68E-38 | Up |
| KLK7 | 1.721408988 | 2.26205994 | 1.97E-08 | 5.88E-08 | Up |
| KIAA1324L | 1.721271109 | 5.628632245 | 1.89E-39 | 5.21E-38 | Up |
| DTHD1 | 1.720400801 | 2.394681579 | 5.73E-16 | 3.16E-15 | Up |
| NR0B2 | 1.719511617 | 3.06967598 | 4.06E-10 | 1.40E-09 | Up |
| MS4A7 | 1.717655058 | 6.38153747 | 2.63E-47 | 1.18E-45 | Up |
| IL7R | 1.717403156 | 5.345380919 | 7.92E-27 | 9.38E-26 | Up |
| OSCAR | 1.715411275 | 5.363193627 | 3.16E-41 | 9.83E-40 | Up |
| TM7SF4 | 1.715145122 | 3.073159888 | 1.15E-22 | 1.01E-21 | Up |
| ECSCR | 1.713756447 | 4.239864841 | 2.06E-62 | 2.79E-60 | Up |
| KIAA1683 | 1.713462901 | 4.483493636 | 6.90E-29 | 9.44E-28 | Up |
| WWC2 | 1.713356146 | 6.584240781 | 5.05E-83 | 4.51E-80 | Up |
| MACROD2 | 1.713126565 | 5.188398096 | 4.04E-17 | 2.40E-16 | Up |
| HLF | 1.712248861 | 5.482745864 | 2.91E-18 | 1.89E-17 | Up |
| PLCXD3 | 1.711478869 | 3.368970484 | 2.60E-16 | 1.47E-15 | Up |
| YSK4 | 1.709172659 | 2.104381317 | 1.23E-11 | 4.85E-11 | Up |
| KCNMB2 | 1.709101771 | 1.87157779 | 4.61E-22 | 3.93E-21 | Up |
| CNTN1 | 1.709093113 | 3.376430495 | 1.73E-12 | 7.30E-12 | Up |
| NLRC4 | 1.708291453 | 4.322117693 | 8.66E-53 | 5.47E-51 | Up |
| NRG1 | 1.708037348 | 3.089486098 | 1.05E-22 | 9.34E-22 | Up |
| CCDC147 | 1.707097239 | 2.198951803 | 1.31E-19 | 9.39E-19 | Up |
| CHIA | 1.706385065 | 3.887285076 | 4.11E-09 | 1.30E-08 | Up |
| CLEC1A | 1.704578123 | 3.996010576 | 8.03E-64 | 1.25E-61 | Up |
| COL4A3 | 1.701241288 | 5.419196108 | 1.95E-16 | 1.11E-15 | Up |
| CYS1 | 1.700964345 | 4.334765259 | 1.91E-23 | 1.77E-22 | Up |
| KCNA5 | 1.700728653 | 2.332188982 | 6.45E-29 | 8.84E-28 | Up |
| TMEM150B | 1.699772235 | 3.353780588 | 8.23E-28 | 1.05E-26 | Up |
| MAG | 1.695937238 | 0.677175539 | 4.20E-26 | 4.76E-25 | Up |
| CCDC37 | 1.695393783 | 1.475353402 | 1.12E-11 | 4.43E-11 | Up |
| MYH11 | 1.694407595 | 7.314739606 | 2.11E-24 | 2.10E-23 | Up |

| | | | | | |
|---|---|---|---|---|---|
| ADAMTS1 | 1.692668008 | 6.582502318 | 4.93E-44 | 1.82E-42 | Up |
| FLRT3 | 1.692612262 | 6.500586543 | 1.20E-24 | 1.21E-23 | Up |
| SMTNL2 | 1.692323868 | 2.68103698 | 4.06E-25 | 4.25E-24 | Up |
| NPNT | 1.691841352 | 7.088306109 | 1.23E-30 | 1.87E-29 | Up |
| S100A3 | 1.68840197 | 3.132323013 | 4.16E-33 | 7.51E-32 | Up |
| GRRP1 | 1.687715202 | 3.095859459 | 2.64E-53 | 1.72E-51 | Up |
| LILRA5 | 1.686640919 | 4.068534166 | 1.47E-36 | 3.37E-35 | Up |
| PCDH10 | 1.685622696 | 1.781015661 | 3.73E-22 | 3.21E-21 | Up |
| CACNB4 | 1.685240367 | 3.721938769 | 1.16E-30 | 1.77E-29 | Up |
| FGFR2 | 1.683420265 | 5.953655331 | 5.49E-28 | 7.05E-27 | Up |
| TNNI2 | 1.68338781 | 2.786427773 | 1.02E-23 | 9.62E-23 | Up |
| CA2 | 1.680769046 | 5.741410978 | 1.36E-28 | 1.83E-27 | Up |
| CHI3L2 | 1.680614504 | 5.312512802 | 1.92E-18 | 1.27E-17 | Up |
| CACNG4 | 1.679393072 | 4.811232339 | 8.61E-11 | 3.14E-10 | Up |
| ARHGAP31 | 1.679337818 | 6.584646421 | 1.26E-55 | 9.90E-54 | Up |
| ID4 | 1.67900527 | 5.519233702 | 4.70E-31 | 7.47E-30 | Up |
| NCRNA00092 | 1.67484543 | 2.193250091 | 1.02E-29 | 1.47E-28 | Up |
| IRX2 | 1.674076764 | 5.542392206 | 1.93E-11 | 7.46E-11 | Up |
| GBA3 | 1.673278831 | 0.906193219 | 4.36E-21 | 3.46E-20 | Up |
| AGAP11 | 1.671951909 | 2.751327491 | 5.17E-30 | 7.61E-29 | Up |
| CTSG | 1.671177768 | 2.513560155 | 3.68E-15 | 1.91E-14 | Up |
| FGD5 | 1.671164357 | 6.05178927 | 1.12E-65 | 1.94E-63 | Up |
| PID1 | 1.669988092 | 5.515275509 | 6.25E-35 | 1.28E-33 | Up |
| SFTPB | 1.66866682 | 11.20041815 | 1.50E-09 | 4.93E-09 | Up |
| ROR1 | 1.668518603 | 4.142266646 | 1.24E-30 | 1.89E-29 | Up |
| FABP5 | 1.667660021 | 4.696967344 | 2.78E-32 | 4.80E-31 | Up |
| CD300C | 1.667351742 | 4.066167645 | 7.48E-37 | 1.73E-35 | Up |
| RASGRP4 | 1.666346164 | 3.851903272 | 1.18E-41 | 3.77E-40 | Up |
| SLFNL1 | 1.666317378 | 3.125023439 | 1.40E-24 | 1.41E-23 | Up |
| CPA3 | 1.664874017 | 5.772049504 | 3.19E-19 | 2.22E-18 | Up |
| RSPH10B2 | 1.664166468 | 1.94256514 | 1.43E-14 | 7.12E-14 | Up |
| C15orf59 | 1.661359746 | 2.866976139 | 5.36E-23 | 4.84E-22 | Up |
| SMAD9 | 1.658014929 | 3.321768925 | 3.55E-27 | 4.33E-26 | Up |
| COL4A6 | 1.657676629 | 2.555137764 | 9.82E-16 | 5.31E-15 | Up |
| WDR63 | 1.656870439 | 2.324722866 | 1.02E-14 | 5.12E-14 | Up |
| CA1 | 1.655626079 | 0.26406176 | 1.79E-36 | 4.06E-35 | Up |
| SLC5A8 | 1.655061065 | 2.742945843 | 1.39E-08 | 4.19E-08 | Up |
| ABCG2 | 1.653293019 | 4.356822585 | 4.06E-40 | 1.15E-38 | Up |
| MYCT1 | 1.652327226 | 5.105380951 | 4.49E-66 | 8.15E-64 | Up |
| ST6GALNAC3 | 1.650572234 | 3.718258135 | 3.55E-44 | 1.31E-42 | Up |
| TMEM88 | 1.645272816 | 3.543658344 | 5.60E-53 | 3.59E-51 | Up |
| TSPAN19 | 1.645157287 | 1.319274643 | 1.32E-10 | 4.71E-10 | Up |
| ZDHHC19 | 1.645152542 | 0.637137047 | 5.11E-34 | 9.88E-33 | Up |
| ELMOD1 | 1.64483415 | 1.235539324 | 8.16E-28 | 1.04E-26 | Up |
| FAM183A | 1.644041615 | 2.469221595 | 9.42E-12 | 3.74E-11 | Up |
| RAI2 | 1.643960849 | 5.265579781 | 2.56E-51 | 1.51E-49 | Up |

| | | | | | |
|---|---|---|---|---|---|
| SEC14L4 | 1.643399766 | 3.75688107 | 6.07E-17 | 3.56E-16 | Up |
| CXCL2 | 1.643357833 | 6.080050907 | 8.53E-24 | 8.13E-23 | Up |
| HRASLS5 | 1.643061233 | 2.819019291 | 1.04E-13 | 4.86E-13 | Up |
| C1orf168 | 1.642842055 | 2.799779513 | 3.48E-12 | 1.43E-11 | Up |
| ZNF365 | 1.641450741 | 3.448059359 | 4.10E-34 | 7.97E-33 | Up |
| CLEC14A | 1.64067531 | 6.245535881 | 1.27E-64 | 2.12E-62 | Up |
| ZMYND10 | 1.639494473 | 4.202543717 | 2.26E-13 | 1.02E-12 | Up |
| SLPI | 1.639175073 | 8.245662663 | 1.38E-15 | 7.40E-15 | Up |
| FAM154B | 1.637593568 | 3.342439769 | 2.37E-14 | 1.16E-13 | Up |
| TNS1 | 1.636668692 | 8.52341268 | 1.85E-54 | 1.32E-52 | Up |
| GFI1B | 1.636658617 | 0.55811964 | 1.13E-31 | 1.88E-30 | Up |
| HAS1 | 1.636572504 | 1.74667504 | 3.01E-14 | 1.46E-13 | Up |
| WDR65 | 1.633785332 | 2.055584759 | 2.52E-15 | 1.33E-14 | Up |
| LRP2 | 1.633413126 | 4.907575556 | 2.93E-09 | 9.39E-09 | Up |
| CNGA4 | 1.632752667 | 1.936845663 | 1.21E-15 | 6.51E-15 | Up |
| NEGR1 | 1.632586463 | 4.16258517 | 1.34E-28 | 1.81E-27 | Up |
| PHOSPHO1 | 1.631880744 | 1.996328957 | 6.38E-35 | 1.31E-33 | Up |
| TBX3 | 1.631483313 | 5.291257597 | 8.09E-48 | 3.72E-46 | Up |
| GLIPR2 | 1.631248917 | 6.555402428 | 3.75E-54 | 2.61E-52 | Up |
| ADAMTS15 | 1.629850843 | 2.77214579 | 1.34E-22 | 1.18E-21 | Up |
| ACSS3 | 1.628957233 | 4.620994508 | 7.85E-29 | 1.07E-27 | Up |
| SEMA3B | 1.628594263 | 6.174535635 | 8.91E-23 | 7.93E-22 | Up |
| THSD1 | 1.627059373 | 4.47710626 | 2.53E-61 | 3.11E-59 | Up |
| SH2D1B | 1.626334961 | 2.314395437 | 1.24E-27 | 1.56E-26 | Up |
| SOX5 | 1.626282641 | 2.608402745 | 2.62E-30 | 3.93E-29 | Up |
| LTBP4 | 1.626216846 | 7.525172762 | 1.02E-50 | 5.82E-49 | Up |
| SH2D3C | 1.625378199 | 5.918776585 | 8.43E-71 | 2.22E-68 | Up |
| FRAS1 | 1.625022949 | 5.335418979 | 6.20E-22 | 5.25E-21 | Up |
| KIR3DL1 | 1.623828185 | 0.695035319 | 2.54E-29 | 3.56E-28 | Up |
| SIRPD | 1.623366505 | 0.379143165 | 9.38E-52 | 5.71E-50 | Up |
| GPR146 | 1.623213755 | 4.765807483 | 6.44E-66 | 1.16E-63 | Up |
| CD93 | 1.622167075 | 7.509849328 | 4.32E-58 | 4.05E-56 | Up |
| PCDHA12 | 1.622054099 | 3.947389389 | 2.64E-15 | 1.39E-14 | Up |
| CYYR1 | 1.621912688 | 5.543646076 | 1.07E-67 | 2.27E-65 | Up |
| ALPL | 1.620707977 | 7.076043244 | 9.24E-16 | 5.01E-15 | Up |
| CRYAB | 1.620271837 | 4.966548782 | 7.71E-41 | 2.32E-39 | Up |
| C3orf16 | 1.618913075 | 1.273262126 | 4.41E-13 | 1.95E-12 | Up |
| LEPR | 1.617978266 | 5.828338257 | 2.26E-39 | 6.16E-38 | Up |
| MT1A | 1.615638021 | 2.291903979 | 3.69E-13 | 1.65E-12 | Up |
| PGR | 1.61539844 | 3.721232102 | 5.49E-32 | 9.33E-31 | Up |
| LOC401093 | 1.614868113 | 4.111749223 | 4.52E-53 | 2.91E-51 | Up |
| TPSB2 | 1.613972191 | 6.479433349 | 2.49E-20 | 1.88E-19 | Up |
| CCDC65 | 1.613544301 | 2.732254408 | 3.85E-17 | 2.30E-16 | Up |
| KLF17 | 1.613405258 | 0.966690849 | 3.06E-34 | 6.02E-33 | Up |
| C4BPA | 1.612776978 | 7.525134328 | 2.09E-10 | 7.38E-10 | Up |
| VIT | 1.610192789 | 0.502778145 | 2.01E-29 | 2.84E-28 | Up |

| | | | | | |
|---|---|---|---|---|---|
| CCL24 | 1.609976938 | 1.095279269 | 4.92E-19 | 3.38E-18 | Up |
| KIF17 | 1.60963763 | 3.493293223 | 5.02E-46 | 2.09E-44 | Up |
| SIGLECP3 | 1.609178562 | 2.688769824 | 5.48E-25 | 5.67E-24 | Up |
| CCIN | 1.608768368 | 1.232887979 | 1.07E-33 | 2.02E-32 | Up |
| PALMD | 1.608526478 | 5.364548664 | 3.69E-48 | 1.73E-46 | Up |
| CAPN9 | 1.608130201 | 4.036009057 | 3.73E-11 | 1.40E-10 | Up |
| MAP1LC3C | 1.607918237 | 2.90685662 | 3.79E-16 | 2.12E-15 | Up |
| GUCA2A | 1.60574757 | 0.469725343 | 8.42E-24 | 8.03E-23 | Up |
| ADAMTSL4 | 1.602928402 | 6.28750137 | 4.83E-38 | 1.21E-36 | Up |
| GATA6 | 1.602155332 | 5.503605214 | 1.81E-34 | 3.59E-33 | Up |
| ANXA3 | 1.599854418 | 6.83875601 | 1.88E-33 | 3.48E-32 | Up |
| C22orf15 | 1.599311836 | 1.816694074 | 1.09E-14 | 5.47E-14 | Up |
| RGS13 | 1.598264599 | 2.535645757 | 6.72E-21 | 5.28E-20 | Up |
| ABCA9 | 1.598085375 | 3.651996197 | 1.02E-31 | 1.70E-30 | Up |
| CXorf57 | 1.598059196 | 3.484573597 | 1.43E-25 | 1.56E-24 | Up |
| IL20RA | 1.597767022 | 4.798311932 | 5.42E-20 | 3.99E-19 | Up |
| C21orf34 | 1.594310141 | 2.312465099 | 2.19E-28 | 2.90E-27 | Up |
| GATA1 | 1.59413296 | 0.798683277 | 3.04E-37 | 7.18E-36 | Up |
| PECAM1 | 1.593965647 | 7.259432742 | 8.01E-90 | 1.83E-86 | Up |
| HEG1 | 1.592907078 | 7.340956817 | 5.96E-55 | 4.42E-53 | Up |
| DAPK2 | 1.591643775 | 5.781231866 | 1.40E-41 | 4.46E-40 | Up |
| STARD8 | 1.591564344 | 5.402920858 | 6.03E-60 | 6.52E-58 | Up |
| RIC3 | 1.591526366 | 3.206517611 | 2.26E-17 | 1.37E-16 | Up |
| HSPB8 | 1.589777388 | 6.237820825 | 1.51E-37 | 3.65E-36 | Up |
| ACSBG1 | 1.588866858 | 1.847837588 | 2.08E-24 | 2.07E-23 | Up |
| CNR1 | 1.588508309 | 4.277376849 | 2.08E-18 | 1.37E-17 | Up |
| NKAPL | 1.58829272 | 1.631276377 | 1.29E-39 | 3.61E-38 | Up |
| ZNF366 | 1.587219417 | 3.154253327 | 1.06E-32 | 1.87E-31 | Up |
| WFDC1 | 1.58627208 | 4.274235491 | 2.69E-29 | 3.77E-28 | Up |
| LRRN4 | 1.585830602 | 5.097124672 | 1.40E-11 | 5.47E-11 | Up |
| TNNT3 | 1.585276598 | 0.748389718 | 5.05E-18 | 3.22E-17 | Up |
| ERG | 1.584956986 | 5.789727883 | 2.00E-63 | 2.98E-61 | Up |
| AHNAK | 1.582077661 | 9.504363881 | 3.13E-41 | 9.76E-40 | Up |
| FGR | 1.581332174 | 5.994709214 | 1.69E-45 | 6.84E-44 | Up |
| GP9 | 1.580215906 | 0.151437488 | 1.05E-48 | 5.07E-47 | Up |
| KCNJ15 | 1.578941212 | 6.040659093 | 7.94E-18 | 4.98E-17 | Up |
| C2orf62 | 1.577942208 | 2.118459993 | 1.59E-14 | 7.86E-14 | Up |
| VTN | 1.57698955 | 2.619602704 | 3.16E-16 | 1.77E-15 | Up |
| C1orf186 | 1.575542448 | 2.777787303 | 1.95E-20 | 1.48E-19 | Up |
| TLR8 | 1.575072441 | 4.82163549 | 4.97E-27 | 5.98E-26 | Up |
| FEZ1 | 1.574303153 | 4.942057107 | 3.45E-54 | 2.41E-52 | Up |
| EFHB | 1.574019453 | 2.507935171 | 4.07E-14 | 1.96E-13 | Up |
| TTLL7 | 1.573116864 | 3.493399556 | 9.78E-20 | 7.06E-19 | Up |
| PEAR1 | 1.572770531 | 4.812201566 | 1.52E-46 | 6.57E-45 | Up |
| SRPX | 1.572701 | 4.937605994 | 8.69E-36 | 1.89E-34 | Up |
| SIGLEC6 | 1.572271939 | 2.336498455 | 2.77E-19 | 1.94E-18 | Up |

| | | | | | |
|---|---|---|---|---|---|
| CSRNP1 | 1.571864735 | 6.958340649 | 1.21E-59 | 1.24E-57 | Up |
| GIMAP6 | 1.570001503 | 6.144367479 | 4.89E-54 | 3.37E-52 | Up |
| RAPGEF4 | 1.568407455 | 4.364607658 | 2.39E-49 | 1.23E-47 | Up |
| PLP1 | 1.568184321 | 0.643894497 | 9.10E-23 | 8.08E-22 | Up |
| LRRC32 | 1.566172245 | 6.558173549 | 3.30E-57 | 2.93E-55 | Up |
| C20orf26 | 1.56546675 | 2.03348825 | 8.42E-14 | 3.95E-13 | Up |
| C8orf34 | 1.56524559 | 3.054449007 | 3.22E-15 | 1.68E-14 | Up |
| SCEL | 1.565157481 | 6.527684843 | 2.75E-15 | 1.44E-14 | Up |
| AFF2 | 1.564418832 | 3.497524862 | 2.07E-17 | 1.26E-16 | Up |
| PCDHGB7 | 1.563987665 | 4.604036082 | 1.02E-30 | 1.56E-29 | Up |
| EXD1 | 1.563782251 | 0.509718753 | 2.46E-34 | 4.85E-33 | Up |
| TPSAB1 | 1.563646136 | 6.010467735 | 1.05E-21 | 8.75E-21 | Up |
| SLCO4C1 | 1.562863979 | 5.335879082 | 3.94E-17 | 2.35E-16 | Up |
| LPHN3 | 1.561363329 | 3.112178077 | 4.46E-16 | 2.48E-15 | Up |
| C5orf38 | 1.561190095 | 3.708038232 | 3.08E-11 | 1.17E-10 | Up |
| NOVA2 | 1.560106463 | 3.437881147 | 3.75E-51 | 2.17E-49 | Up |
| SLC15A2 | 1.558265381 | 5.973186264 | 7.15E-26 | 7.97E-25 | Up |
| TPPP | 1.557982957 | 5.744570282 | 8.65E-23 | 7.72E-22 | Up |
| SPATA4 | 1.557223932 | 1.0566932 | 1.51E-16 | 8.63E-16 | Up |
| SLC6A20 | 1.556645839 | 3.90535489 | 1.44E-09 | 4.73E-09 | Up |
| TMSB15A | 1.556364495 | 1.879870126 | 1.29E-20 | 9.88E-20 | Up |
| WASF3 | 1.555990842 | 5.071574767 | 5.41E-38 | 1.35E-36 | Up |
| SRL | 1.555425171 | 2.455601338 | 1.53E-34 | 3.06E-33 | Up |
| FCER1A | 1.554941149 | 3.883310591 | 7.92E-12 | 3.17E-11 | Up |
| GFRA2 | 1.552937555 | 2.875376709 | 1.38E-27 | 1.73E-26 | Up |
| RGS9BP | 1.552549913 | 1.794696243 | 9.70E-26 | 1.07E-24 | Up |
| CCDC81 | 1.552311724 | 2.244089986 | 5.91E-21 | 4.65E-20 | Up |
| AQP10 | 1.551950745 | 0.429928234 | 3.34E-35 | 7.00E-34 | Up |
| NRN1 | 1.551787345 | 4.790739506 | 3.73E-32 | 6.38E-31 | Up |
| FAM13C | 1.550963864 | 4.291057053 | 5.51E-34 | 1.06E-32 | Up |
| AJAP1 | 1.549728286 | 1.345884631 | 8.87E-23 | 7.90E-22 | Up |
| VGLL1 | 1.549323009 | 2.778863165 | 1.86E-09 | 6.06E-09 | Up |
| CCDC33 | 1.54908638 | 1.833208852 | 1.39E-10 | 4.97E-10 | Up |
| FRMPD1 | 1.545407397 | 0.795874581 | 1.36E-20 | 1.05E-19 | Up |
| KIAA1462 | 1.545066673 | 6.834032931 | 1.18E-51 | 7.17E-50 | Up |
| TMEM213 | 1.544211806 | 3.023484537 | 9.44E-10 | 3.15E-09 | Up |
| SELE | 1.543986118 | 3.447748412 | 5.26E-17 | 3.10E-16 | Up |
| LOC100144604 | 1.543352535 | 1.489532242 | 1.66E-14 | 8.23E-14 | Up |
| BMP2 | 1.540585118 | 5.923156903 | 7.40E-25 | 7.59E-24 | Up |
| COLEC12 | 1.540416332 | 6.421856894 | 3.70E-27 | 4.50E-26 | Up |
| GPR133 | 1.538548672 | 6.062066597 | 6.16E-18 | 3.90E-17 | Up |
| PTCHD1 | 1.536121411 | 1.864397941 | 2.45E-14 | 1.20E-13 | Up |
| TIMP3 | 1.534817556 | 9.057436198 | 6.56E-39 | 1.72E-37 | Up |
| APOH | 1.534550081 | 3.447444252 | 8.93E-08 | 2.50E-07 | Up |
| ALOX5AP | 1.534360891 | 6.613717878 | 8.49E-33 | 1.51E-31 | Up |
| MMRN2 | 1.533835599 | 6.182651516 | 8.02E-60 | 8.57E-58 | Up |

| | | | | | |
|---|---|---|---|---|---|
| PTRF | 1.53319808 | 8.292847117 | 9.45E-59 | 9.19E-57 | Up |
| LOC284276 | 1.530458396 | 2.042252482 | 1.24E-19 | 8.87E-19 | Up |
| DNAH10 | 1.529730421 | 3.704418976 | 8.61E-16 | 4.68E-15 | Up |
| TNNT2 | 1.527760977 | 2.672917568 | 1.56E-14 | 7.75E-14 | Up |
| LPPR3 | 1.525845438 | 2.359916626 | 5.09E-11 | 1.89E-10 | Up |
| KCNS1 | 1.525582448 | 2.449898912 | 4.08E-12 | 1.67E-11 | Up |
| HIF3A | 1.52537988 | 4.896223665 | 6.14E-13 | 2.69E-12 | Up |
| ARMC4 | 1.52507235 | 2.864019043 | 4.49E-11 | 1.67E-10 | Up |
| EDN1 | 1.524750574 | 6.018901733 | 2.92E-28 | 3.83E-27 | Up |
| PRSS12 | 1.523139393 | 4.558035009 | 1.50E-12 | 6.35E-12 | Up |
| PROK2 | 1.522810101 | 1.032357686 | 2.48E-22 | 2.15E-21 | Up |
| VWA3B | 1.521835017 | 3.23071286 | 1.48E-10 | 5.29E-10 | Up |
| STARD13 | 1.521553706 | 5.638062306 | 9.10E-71 | 2.37E-68 | Up |
| CDH13 | 1.519849437 | 5.046529556 | 1.10E-34 | 2.22E-33 | Up |
| HK3 | 1.516849017 | 5.494486385 | 5.72E-26 | 6.42E-25 | Up |
| GSG1L | 1.516786548 | 0.215670338 | 5.20E-42 | 1.70E-40 | Up |
| ECT2L | 1.516507703 | 2.626289976 | 1.88E-15 | 9.96E-15 | Up |
| SOCS2 | 1.515581583 | 5.856102441 | 5.99E-31 | 9.41E-30 | Up |
| SYNPO2 | 1.514741184 | 5.683618658 | 3.76E-37 | 8.84E-36 | Up |
| MEFV | 1.514168684 | 2.927977378 | 5.61E-33 | 1.01E-31 | Up |
| C11orf66 | 1.512510733 | 3.053709949 | 7.44E-19 | 5.06E-18 | Up |
| TSPAN18 | 1.51149765 | 5.7492144 | 1.27E-45 | 5.19E-44 | Up |
| CASS4 | 1.511186141 | 4.512873381 | 1.67E-32 | 2.92E-31 | Up |
| LRRC50 | 1.508265934 | 3.072220922 | 4.37E-12 | 1.78E-11 | Up |
| LDLRAD1 | 1.507781909 | 2.31216876 | 2.96E-10 | 1.03E-09 | Up |
| GALNTL1 | 1.507449549 | 3.103276079 | 2.20E-17 | 1.34E-16 | Up |
| C1orf141 | 1.506298427 | 0.721551907 | 7.50E-17 | 4.36E-16 | Up |
| HPGDS | 1.504559758 | 3.980571371 | 1.05E-17 | 6.55E-17 | Up |
| DPCR1 | 1.504374233 | 4.082697722 | 7.47E-09 | 2.31E-08 | Up |
| IRX6 | 1.503895389 | 3.036078652 | 8.35E-09 | 2.57E-08 | Up |
| CCDC42B | 1.503317339 | 2.686527074 | 3.89E-12 | 1.60E-11 | Up |
| KRT13 | 1.50330357 | 2.184351322 | 3.52E-09 | 1.12E-08 | Up |
| F10 | 1.502573547 | 3.825355776 | 8.31E-27 | 9.81E-26 | Up |
| MDGA1 | 1.502351053 | 4.557330932 | 6.78E-30 | 9.88E-29 | Up |
| KLRF1 | 1.500988596 | 2.302095848 | 1.04E-19 | 7.48E-19 | Up |
| PTGDS | 1.500728107 | 6.829904481 | 2.47E-22 | 2.14E-21 | Up |
| C14orf139 | 1.498925276 | 4.137601789 | 2.85E-39 | 7.72E-38 | Up |
| AMICA1 | 1.497826054 | 5.978797289 | 3.39E-34 | 6.64E-33 | Up |
| ESAM | 1.497417114 | 6.830258151 | 7.02E-50 | 3.73E-48 | Up |
| GPBAR1 | 1.49693288 | 2.862756655 | 5.63E-34 | 1.08E-32 | Up |
| CLEC4E | 1.496434625 | 3.892515953 | 7.37E-21 | 5.77E-20 | Up |
| METTL7A | 1.494596634 | 7.212401062 | 2.65E-43 | 9.30E-42 | Up |
| RERGL | 1.494030158 | 1.439346797 | 1.24E-10 | 4.44E-10 | Up |
| FAM166B | 1.493683372 | 2.647411669 | 5.91E-12 | 2.39E-11 | Up |
| ENDOU | 1.493423903 | 0.756455978 | 1.22E-30 | 1.86E-29 | Up |
| C5AR1 | 1.492396993 | 6.101040691 | 1.03E-40 | 3.05E-39 | Up |

| | | | | | |
|---|---|---|---|---|---|
| CD300LF | 1.49226034 | 5.013042677 | 3.41E-31 | 5.48E-30 | Up |
| ITM2A | 1.49165658 | 5.78861069 | 2.10E-39 | 5.74E-38 | Up |
| SLC8A3 | 1.491352815 | 1.856075567 | 9.01E-23 | 8.01E-22 | Up |
| C1QTNF2 | 1.490360386 | 3.119361947 | 2.35E-34 | 4.64E-33 | Up |
| GPR109A | 1.488250149 | 5.041409578 | 9.45E-20 | 6.82E-19 | Up |
| SYT4 | 1.48665087 | 0.667554417 | 2.35E-11 | 9.01E-11 | Up |
| ST6GALNAC2 | 1.486558583 | 5.322249928 | 2.17E-23 | 2.01E-22 | Up |
| LDB3 | 1.48644978 | 2.904238903 | 4.55E-28 | 5.87E-27 | Up |
| CYBRD1 | 1.484798199 | 7.844413844 | 2.77E-37 | 6.56E-36 | Up |
| P2RX6 | 1.484089175 | 1.452363215 | 2.00E-22 | 1.74E-21 | Up |
| A2M | 1.482816862 | 9.930422024 | 5.34E-39 | 1.41E-37 | Up |
| ENPP6 | 1.480384891 | 1.587395762 | 4.27E-27 | 5.17E-26 | Up |
| FCN1 | 1.480304166 | 4.876864635 | 2.18E-21 | 1.77E-20 | Up |
| FBLN5 | 1.478761588 | 6.781600047 | 1.18E-37 | 2.89E-36 | Up |
| NRGN | 1.476874821 | 5.876904498 | 4.15E-19 | 2.87E-18 | Up |
| KIF26A | 1.476433742 | 4.045165114 | 1.21E-23 | 1.14E-22 | Up |
| SLC13A2 | 1.474574262 | 1.730801488 | 1.23E-06 | 3.11E-06 | Up |
| TCTEX1D1 | 1.474566298 | 2.6288349 | 2.91E-22 | 2.52E-21 | Up |
| MSRB3 | 1.473952028 | 6.093601578 | 2.80E-43 | 9.78E-42 | Up |
| PRKCQ | 1.47340621 | 4.294679373 | 7.84E-27 | 9.29E-26 | Up |
| CAMK2A | 1.473011155 | 2.016920118 | 2.05E-28 | 2.72E-27 | Up |
| LPHN2 | 1.472626949 | 6.548007723 | 4.05E-40 | 1.15E-38 | Up |
| ABCA10 | 1.472054511 | 2.313292803 | 3.52E-21 | 2.82E-20 | Up |
| DUOXA2 | 1.471652987 | 2.824821721 | 5.80E-13 | 2.55E-12 | Up |
| C1orf114 | 1.47162555 | 2.609036088 | 1.22E-13 | 5.68E-13 | Up |
| LOC100302650 | 1.471267541 | 2.982143696 | 2.39E-24 | 2.37E-23 | Up |
| C2orf73 | 1.468877411 | 1.05251539 | 7.53E-17 | 4.38E-16 | Up |
| NR4A1 | 1.468507208 | 7.645675949 | 6.43E-21 | 5.06E-20 | Up |
| MUSTN1 | 1.468103943 | 3.406797432 | 2.57E-31 | 4.18E-30 | Up |
| CASP5 | 1.467921734 | 1.480036308 | 1.30E-21 | 1.07E-20 | Up |
| IL17D | 1.467003942 | 2.780876804 | 9.16E-31 | 1.42E-29 | Up |
| LRP2BP | 1.466933234 | 4.166717327 | 1.88E-42 | 6.26E-41 | Up |
| SLC11A1 | 1.465719281 | 6.212790247 | 4.39E-35 | 9.14E-34 | Up |
| SCD5 | 1.465609755 | 5.630975665 | 4.04E-26 | 4.59E-25 | Up |
| RTN4RL1 | 1.465271396 | 3.906364703 | 5.47E-14 | 2.60E-13 | Up |
| WDR49 | 1.465213433 | 2.247460926 | 7.73E-12 | 3.10E-11 | Up |
| AOX1 | 1.464562495 | 4.701749039 | 2.01E-19 | 1.42E-18 | Up |
| SPARCL1 | 1.463897551 | 8.092294296 | 6.52E-43 | 2.23E-41 | Up |
| C5orf23 | 1.46336197 | 5.329279758 | 4.62E-30 | 6.82E-29 | Up |
| CLEC6A | 1.463361914 | 1.212051822 | 1.50E-18 | 9.96E-18 | Up |
| PPARGC1A | 1.462395386 | 4.326773741 | 1.38E-11 | 5.42E-11 | Up |
| LOH3CR2A | 1.461237227 | 3.685065048 | 8.02E-38 | 1.98E-36 | Up |
| TEX14 | 1.460284757 | 2.017995272 | 2.77E-35 | 5.82E-34 | Up |
| GAS2L2 | 1.45996962 | 2.857752677 | 4.07E-10 | 1.40E-09 | Up |
| MYL3 | 1.45973574 | 0.94480882 | 5.87E-26 | 6.58E-25 | Up |
| TMEM178 | 1.457590218 | 3.54283958 | 1.98E-21 | 1.61E-20 | Up |

| | | | | | |
|---|---|---|---|---|---|
| ENHO | 1.456686203 | 2.118022399 | 2.80E-16 | 1.58E-15 | Up |
| PLLP | 1.454649185 | 6.116019796 | 7.45E-26 | 8.29E-25 | Up |
| ALOX5 | 1.454567731 | 6.804847935 | 1.49E-34 | 2.99E-33 | Up |
| DENND2A | 1.454149331 | 5.030869123 | 2.44E-38 | 6.23E-37 | Up |
| SEMA3E | 1.453162009 | 4.434357868 | 1.34E-09 | 4.40E-09 | Up |
| RICH2 | 1.452851267 | 5.954040467 | 1.05E-27 | 1.32E-26 | Up |
| SCNN1G | 1.452244393 | 5.175630403 | 1.12E-13 | 5.22E-13 | Up |
| HOXA4 | 1.450465192 | 3.428194994 | 1.09E-30 | 1.67E-29 | Up |
| TTPA | 1.450223114 | 0.577553114 | 6.68E-25 | 6.87E-24 | Up |
| FAM134B | 1.450103722 | 5.204508015 | 1.09E-22 | 9.63E-22 | Up |
| OTUD1 | 1.448833597 | 6.037745026 | 9.29E-94 | 4.77E-90 | Up |
| RTN1 | 1.44831633 | 4.710958641 | 8.04E-26 | 8.94E-25 | Up |
| PPARG | 1.448222139 | 5.582174842 | 2.50E-22 | 2.17E-21 | Up |
| FREM2 | 1.447507862 | 5.270734843 | 4.31E-12 | 1.76E-11 | Up |
| DOK2 | 1.447209198 | 5.7476481 | 3.17E-36 | 7.14E-35 | Up |
| TYRP1 | 1.446983734 | 3.125549546 | 6.57E-14 | 3.11E-13 | Up |
| TIE1 | 1.444086441 | 6.220864496 | 2.21E-51 | 1.31E-49 | Up |
| TEKT2 | 1.443055191 | 3.077322522 | 2.51E-09 | 8.08E-09 | Up |
| ALS2CR12 | 1.442549617 | 1.233162167 | 3.66E-15 | 1.90E-14 | Up |
| KLF15 | 1.441921447 | 4.681102973 | 2.53E-18 | 1.65E-17 | Up |
| ZNF423 | 1.441428267 | 4.066116936 | 4.69E-37 | 1.10E-35 | Up |
| CEACAM21 | 1.441396222 | 3.486548411 | 1.33E-24 | 1.35E-23 | Up |
| MATN3 | 1.440989191 | 4.948362285 | 4.87E-23 | 4.41E-22 | Up |
| C10orf79 | 1.438483835 | 3.65540634 | 4.46E-11 | 1.66E-10 | Up |
| IGFALS | 1.438415537 | 3.140699832 | 1.06E-13 | 4.92E-13 | Up |
| PPP1R14A | 1.436858326 | 4.675961493 | 5.26E-34 | 1.01E-32 | Up |
| CLDN11 | 1.435161931 | 4.18842117 | 1.15E-22 | 1.02E-21 | Up |
| PRKG1 | 1.433951492 | 3.820308104 | 2.17E-25 | 2.32E-24 | Up |
| LDLR | 1.432890895 | 7.60463917 | 4.60E-47 | 2.05E-45 | Up |
| SLC16A12 | 1.430087056 | 3.305166515 | 3.24E-12 | 1.34E-11 | Up |
| GIMAP5 | 1.428707114 | 6.088495537 | 7.46E-42 | 2.40E-40 | Up |
| C1orf110 | 1.428474108 | 1.242689748 | 7.53E-10 | 2.54E-09 | Up |
| KLRG2 | 1.428297391 | 2.65126875 | 5.41E-13 | 2.38E-12 | Up |
| MFSD2A | 1.428134376 | 6.508461167 | 1.02E-27 | 1.28E-26 | Up |
| SHROOM4 | 1.427272245 | 6.091562333 | 7.48E-32 | 1.26E-30 | Up |
| LRRC19 | 1.426448823 | 0.999320635 | 2.39E-20 | 1.81E-19 | Up |
| MLC1 | 1.425149412 | 2.796873968 | 1.55E-18 | 1.03E-17 | Up |
| AQP9 | 1.424990086 | 5.297952712 | 1.90E-20 | 1.45E-19 | Up |
| C1orf162 | 1.422796731 | 5.754727263 | 5.55E-40 | 1.57E-38 | Up |
| CCDC135 | 1.42272793 | 2.161354071 | 2.83E-09 | 9.08E-09 | Up |
| KCNK17 | 1.422434523 | 3.094861771 | 3.05E-15 | 1.60E-14 | Up |
| PLAC8 | 1.422013512 | 5.272419134 | 2.09E-16 | 1.19E-15 | Up |
| C10orf128 | 1.421854316 | 3.15815057 | 1.58E-26 | 1.84E-25 | Up |
| C3orf15 | 1.421133781 | 3.700363415 | 9.03E-13 | 3.90E-12 | Up |
| SLC18A2 | 1.420550362 | 2.872648218 | 2.34E-13 | 1.06E-12 | Up |
| HSPB2 | 1.419443365 | 3.905894335 | 1.27E-40 | 3.76E-39 | Up |

| | | | | | |
|---|---|---|---|---|---|
| GATA5 | 1.418858058 | 2.458004024 | 2.74E-18 | 1.78E-17 | Up |
| CX3CR1 | 1.418745297 | 4.249720665 | 9.89E-18 | 6.17E-17 | Up |
| FPR1 | 1.418723473 | 5.418379238 | 1.96E-25 | 2.10E-24 | Up |
| LAMC3 | 1.417877022 | 5.577500275 | 6.21E-24 | 5.98E-23 | Up |
| RBM24 | 1.416922962 | 2.024934807 | 4.67E-15 | 2.40E-14 | Up |
| MYH1 | 1.415402326 | 0.416680044 | 1.11E-24 | 1.12E-23 | Up |
| KCNAB1 | 1.414512531 | 3.75649816 | 1.77E-54 | 1.26E-52 | Up |
| TRPV2 | 1.413655935 | 6.205181967 | 5.93E-47 | 2.63E-45 | Up |
| SEMA3D | 1.413528727 | 3.813136151 | 4.26E-18 | 2.73E-17 | Up |
| CCDC17 | 1.412912879 | 3.386806545 | 1.46E-11 | 5.69E-11 | Up |
| SPATA18 | 1.412139983 | 4.907207266 | 4.45E-15 | 2.29E-14 | Up |
| TACC1 | 1.411854447 | 7.626417435 | 1.16E-53 | 7.68E-52 | Up |
| PDE5A | 1.411732495 | 5.891551014 | 3.32E-49 | 1.68E-47 | Up |
| CAT | 1.411188291 | 7.598418217 | 2.66E-66 | 4.87E-64 | Up |
| P2RY1 | 1.411030305 | 3.574597913 | 7.11E-34 | 1.36E-32 | Up |
| NPY1R | 1.410536707 | 2.606455712 | 3.08E-14 | 1.49E-13 | Up |
| ADCY4 | 1.410296933 | 4.778141984 | 1.48E-46 | 6.42E-45 | Up |
| FILIP1 | 1.409786306 | 5.053126605 | 6.71E-21 | 5.27E-20 | Up |
| MYOM2 | 1.409621628 | 2.958202775 | 1.07E-21 | 8.91E-21 | Up |
| FXYD6 | 1.409491556 | 6.307226924 | 5.75E-47 | 2.55E-45 | Up |
| ODZ2 | 1.409265935 | 1.843022979 | 8.34E-14 | 3.91E-13 | Up |
| TBX2 | 1.409159693 | 6.164452389 | 5.42E-39 | 1.43E-37 | Up |
| C1orf230 | 1.408924494 | 1.364519164 | 2.89E-11 | 1.10E-10 | Up |
| CXCL3 | 1.40699104 | 4.060972204 | 4.64E-18 | 2.98E-17 | Up |
| LMOD1 | 1.406342398 | 5.79710776 | 2.72E-32 | 4.70E-31 | Up |
| LRRC48 | 1.406063279 | 3.553601678 | 3.13E-17 | 1.88E-16 | Up |
| GNG11 | 1.404490856 | 6.417939366 | 7.43E-32 | 1.25E-30 | Up |
| MRAP2 | 1.404245819 | 2.285924915 | 9.05E-12 | 3.60E-11 | Up |
| LYPD2 | 1.403478508 | 0.320749324 | 3.69E-20 | 2.75E-19 | Up |
| LRCH2 | 1.403167033 | 4.133693028 | 1.07E-21 | 8.92E-21 | Up |
| AMOTL1 | 1.402772163 | 6.725619805 | 8.73E-45 | 3.36E-43 | Up |
| LILRA1 | 1.402278767 | 2.076726836 | 6.64E-25 | 6.83E-24 | Up |
| MAOB | 1.401649243 | 5.730107414 | 1.62E-24 | 1.62E-23 | Up |
| ABCA6 | 1.401169107 | 4.354511721 | 2.33E-23 | 2.15E-22 | Up |
| WDR69 | 1.401017304 | 2.872305324 | 1.05E-09 | 3.50E-09 | Up |
| TLL1 | 1.400706788 | 3.678014327 | 7.26E-27 | 8.62E-26 | Up |
| LAMA3 | 1.399436258 | 6.889725061 | 7.06E-13 | 3.08E-12 | Up |
| OSBPL6 | 1.399129218 | 4.120392262 | 3.28E-19 | 2.28E-18 | Up |
| PRKCE | 1.398837057 | 5.770301235 | 6.59E-73 | 2.08E-70 | Up |
| COX7A1 | 1.397653084 | 4.414462069 | 3.93E-39 | 1.05E-37 | Up |
| PCDH11X | 1.397253866 | 0.596523872 | 2.70E-22 | 2.34E-21 | Up |
| ALDH1A1 | 1.395767313 | 7.267007208 | 1.32E-17 | 8.14E-17 | Up |
| EFEMP1 | 1.394683081 | 7.630925321 | 8.12E-32 | 1.36E-30 | Up |
| NWD1 | 1.394112073 | 3.372193736 | 7.01E-08 | 1.99E-07 | Up |
| LMO2 | 1.394064822 | 5.463370868 | 2.43E-59 | 2.46E-57 | Up |
| SLCO2A1 | 1.393956423 | 7.040422203 | 3.66E-27 | 4.45E-26 | Up |

| | | | | | |
|---|---|---|---|---|---|
| SEPP1 | 1.392990631 | 8.583066721 | 3.25E-33 | 5.93E-32 | Up |
| TEKT3 | 1.392675746 | 1.611544616 | 3.25E-16 | 1.83E-15 | Up |
| VWC2 | 1.392612329 | 0.236306691 | 5.19E-36 | 1.15E-34 | Up |
| NEBL | 1.391993336 | 7.017119927 | 4.73E-31 | 7.51E-30 | Up |
| IQCA1 | 1.391154223 | 4.109430022 | 1.37E-13 | 6.34E-13 | Up |
| SPATS1 | 1.39068756 | 0.576331024 | 3.28E-15 | 1.71E-14 | Up |
| FREM1 | 1.389904421 | 3.448363774 | 5.98E-14 | 2.83E-13 | Up |
| PLCL1 | 1.389442319 | 4.433820337 | 3.97E-47 | 1.78E-45 | Up |
| CSMD1 | 1.389376409 | 1.614875696 | 1.23E-09 | 4.07E-09 | Up |
| ATP8A2 | 1.388000051 | 2.832322518 | 4.04E-14 | 1.94E-13 | Up |
| HRCT1 | 1.387792546 | 3.158807136 | 5.26E-21 | 4.16E-20 | Up |
| PDE2A | 1.386851849 | 4.602841421 | 3.60E-33 | 6.53E-32 | Up |
| NOSTRIN | 1.386790939 | 5.188856176 | 3.25E-26 | 3.71E-25 | Up |
| GAS6 | 1.385801063 | 7.573559237 | 4.64E-41 | 1.42E-39 | Up |
| NHSL1 | 1.385705787 | 6.111396688 | 6.66E-43 | 2.27E-41 | Up |
| RHOJ | 1.385288564 | 4.831056663 | 2.82E-52 | 1.74E-50 | Up |
| GIMAP7 | 1.385241849 | 5.561824072 | 2.61E-41 | 8.16E-40 | Up |
| EML1 | 1.384479083 | 5.403721633 | 2.15E-37 | 5.14E-36 | Up |
| KCTD16 | 1.381958662 | 2.126479282 | 1.64E-23 | 1.53E-22 | Up |
| GATA2 | 1.381281228 | 5.463519091 | 1.41E-33 | 2.64E-32 | Up |
| LTC4S | 1.381117516 | 2.898221254 | 4.30E-24 | 4.19E-23 | Up |
| CD300E | 1.379660457 | 1.335928275 | 1.47E-17 | 9.03E-17 | Up |
| DUSP26 | 1.379342318 | 2.093484319 | 1.13E-17 | 7.04E-17 | Up |
| FAM124B | 1.378730362 | 3.289829161 | 3.69E-37 | 8.69E-36 | Up |
| ITIH3 | 1.378470342 | 2.722917807 | 3.28E-21 | 2.63E-20 | Up |
| PAQR5 | 1.378308229 | 5.353598096 | 8.40E-26 | 9.33E-25 | Up |
| NPR3 | 1.377570721 | 4.059644221 | 1.84E-20 | 1.40E-19 | Up |
| DNAH6 | 1.37746233 | 3.218437347 | 1.40E-11 | 5.49E-11 | Up |
| FAM179A | 1.376004177 | 3.412022227 | 6.77E-14 | 3.20E-13 | Up |
| KLK5 | 1.374801464 | 1.20300247 | 1.09E-07 | 3.04E-07 | Up |
| SOBP | 1.373650584 | 4.241314536 | 4.29E-30 | 6.35E-29 | Up |
| SYNM | 1.373037223 | 5.66777721 | 3.61E-47 | 1.62E-45 | Up |
| BAALC | 1.372979947 | 4.308764592 | 1.72E-12 | 7.24E-12 | Up |
| HSPC159 | 1.372470098 | 6.007541359 | 2.31E-42 | 7.66E-41 | Up |
| PDLIM2 | 1.371721585 | 6.398342314 | 1.08E-59 | 1.14E-57 | Up |
| C18orf34 | 1.371624831 | 0.645507747 | 4.91E-21 | 3.89E-20 | Up |
| FLJ42875 | 1.370902404 | 2.687461157 | 9.78E-11 | 3.54E-10 | Up |
| C5orf49 | 1.370268445 | 3.775531028 | 4.05E-11 | 1.52E-10 | Up |
| CYP4Z1 | 1.368017438 | 1.151850388 | 7.92E-14 | 3.72E-13 | Up |
| LIPN | 1.366682181 | 0.140964368 | 2.22E-39 | 6.06E-38 | Up |
| STAB2 | 1.365436086 | 2.16042485 | 3.31E-13 | 1.48E-12 | Up |
| C10orf105 | 1.364497423 | 2.117535747 | 1.38E-24 | 1.40E-23 | Up |
| LSAMP | 1.363837046 | 4.271177217 | 8.93E-27 | 1.05E-25 | Up |
| LIFR | 1.360502429 | 7.092045754 | 2.42E-32 | 4.19E-31 | Up |
| NTN4 | 1.358587256 | 6.887977043 | 3.26E-34 | 6.39E-33 | Up |
| DHH | 1.357713815 | 1.281762196 | 2.31E-26 | 2.67E-25 | Up |

| | | | | | |
|---|---|---|---|---|---|
| HPD | 1.356251553 | 0.952334298 | 4.85E-15 | 2.49E-14 | Up |
| CADM1 | 1.355430792 | 7.342078552 | 2.65E-22 | 2.30E-21 | Up |
| GPR109B | 1.355273514 | 3.870618207 | 1.24E-18 | 8.31E-18 | Up |
| DENND3 | 1.354573215 | 6.730564577 | 7.68E-63 | 1.09E-60 | Up |
| C6orf165 | 1.35354247 | 2.955389415 | 2.93E-12 | 1.21E-11 | Up |
| SCTR | 1.352493733 | 4.710433601 | 1.90E-06 | 4.73E-06 | Up |
| CYP27A1 | 1.351783786 | 7.116556543 | 9.33E-32 | 1.56E-30 | Up |
| LILRA2 | 1.350598144 | 4.026062439 | 3.21E-17 | 1.93E-16 | Up |
| TMEM47 | 1.350540674 | 5.933496915 | 4.69E-39 | 1.24E-37 | Up |
| PDE1B | 1.350369043 | 4.324631216 | 8.28E-31 | 1.28E-29 | Up |
| S1PR5 | 1.349352733 | 2.721352007 | 1.77E-24 | 1.77E-23 | Up |
| LOC121838 | 1.349095872 | 0.669444731 | 1.53E-17 | 9.39E-17 | Up |
| SASH1 | 1.348934123 | 6.537455778 | 5.23E-60 | 5.71E-58 | Up |
| ACE | 1.348444052 | 6.937698516 | 3.67E-40 | 1.05E-38 | Up |
| CELA2B | 1.348086434 | 0.0772724 | 7.45E-66 | 1.33E-63 | Up |
| DYDC2 | 1.347727782 | 2.638626463 | 7.33E-10 | 2.47E-09 | Up |
| DYDC1 | 1.347095321 | 0.77154278 | 1.58E-13 | 7.26E-13 | Up |
| ABLIM3 | 1.344357929 | 5.650931609 | 4.26E-25 | 4.45E-24 | Up |
| FAM184A | 1.344348901 | 4.905915682 | 1.20E-17 | 7.43E-17 | Up |
| MAL | 1.344121008 | 4.313766841 | 2.52E-17 | 1.53E-16 | Up |
| SPHKAP | 1.343757561 | 0.237639553 | 3.63E-25 | 3.82E-24 | Up |
| SERPINA9 | 1.342684423 | 0.709295527 | 8.23E-17 | 4.78E-16 | Up |
| GRID1 | 1.34259918 | 2.994185443 | 6.28E-23 | 5.64E-22 | Up |
| CPEB1 | 1.342039204 | 1.592657243 | 2.65E-15 | 1.39E-14 | Up |
| KRT1 | 1.341926465 | 1.076735665 | 4.18E-13 | 1.85E-12 | Up |
| TRPC3 | 1.341391093 | 1.657607627 | 6.17E-23 | 5.55E-22 | Up |
| ATP1B2 | 1.340205311 | 3.572912495 | 3.40E-26 | 3.88E-25 | Up |
| AZU1 | 1.3395216 | 1.067105446 | 1.11E-10 | 4.01E-10 | Up |
| CHRM4 | 1.33732876 | 0.94571867 | 2.86E-19 | 2.00E-18 | Up |
| KLF2 | 1.336710183 | 6.01717386 | 6.82E-33 | 1.22E-31 | Up |
| ROS1 | 1.336098558 | 6.550734332 | 3.32E-09 | 1.06E-08 | Up |
| FOLR1 | 1.335746396 | 7.382066489 | 1.21E-09 | 4.00E-09 | Up |
| CDH23 | 1.334746251 | 4.039721498 | 1.19E-20 | 9.17E-20 | Up |
| DUSP1 | 1.334081736 | 8.57917755 | 3.13E-25 | 3.31E-24 | Up |
| PNMT | 1.333388434 | 1.843936805 | 1.05E-11 | 4.13E-11 | Up |
| PIGR | 1.333347141 | 8.538503874 | 2.43E-06 | 6.02E-06 | Up |
| UBASH3B | 1.332660749 | 5.485636807 | 2.01E-30 | 3.03E-29 | Up |
| TLR4 | 1.332236838 | 5.988850454 | 4.56E-35 | 9.47E-34 | Up |
| RNF144B | 1.331593816 | 6.469036249 | 2.07E-44 | 7.76E-43 | Up |
| PEG3 | 1.331135642 | 3.768191845 | 1.08E-22 | 9.53E-22 | Up |
| 90288 | 1.331080018 | 2.69919091 | 1.16E-12 | 4.96E-12 | Up |
| SNCA | 1.330685169 | 3.760721519 | 3.97E-29 | 5.52E-28 | Up |
| CNTNAP3 | 1.330399147 | 2.663089756 | 3.72E-13 | 1.66E-12 | Up |
| ACSM5 | 1.329889655 | 2.089971787 | 3.05E-16 | 1.72E-15 | Up |
| NEDD9 | 1.329462751 | 7.86094489 | 1.53E-32 | 2.69E-31 | Up |
| C17orf87 | 1.329258036 | 3.689093675 | 1.53E-22 | 1.34E-21 | Up |

| | | | | | |
|---|---|---|---|---|---|
| SLC7A4 | 1.329028818 | 2.844220365 | 4.64E-09 | 1.46E-08 | Up |
| PTGER4 | 1.327934768 | 5.588866646 | 3.48E-33 | 6.34E-32 | Up |
| PTPRO | 1.327811693 | 4.710625002 | 5.04E-24 | 4.89E-23 | Up |
| UPB1 | 1.327356084 | 1.458168008 | 2.95E-18 | 1.91E-17 | Up |
| S1PR4 | 1.326780927 | 4.764479348 | 7.09E-35 | 1.45E-33 | Up |
| LGI4 | 1.324140048 | 3.489037152 | 1.91E-36 | 4.32E-35 | Up |
| MUC15 | 1.323427572 | 5.258694585 | 3.14E-09 | 1.00E-08 | Up |
| PYGM | 1.321970472 | 2.511554273 | 5.87E-23 | 5.29E-22 | Up |
| KIR2DL3 | 1.321816555 | 0.625266373 | 9.12E-19 | 6.14E-18 | Up |
| SLITRK3 | 1.321419306 | 1.006896155 | 8.34E-15 | 4.20E-14 | Up |
| C1orf115 | 1.321126152 | 6.076952896 | 7.65E-29 | 1.04E-27 | Up |
| GRAMD2 | 1.320117336 | 5.700787556 | 5.91E-20 | 4.33E-19 | Up |
| RAB40A | 1.319697868 | 2.068725742 | 9.63E-27 | 1.13E-25 | Up |
| AG2 | 1.318037987 | 6.269891889 | 8.02E-27 | 9.49E-26 | Up |
| CXCL5 | 1.316871476 | 4.684993118 | 1.59E-07 | 4.36E-07 | Up |
| C1orf189 | 1.316401018 | 0.485100072 | 5.56E-16 | 3.06E-15 | Up |
| PHLDB2 | 1.316350805 | 6.106075163 | 2.54E-29 | 3.56E-28 | Up |
| RELN | 1.31634048 | 3.232586941 | 7.14E-12 | 2.87E-11 | Up |
| HEMGN | 1.315982739 | 0.191515192 | 2.01E-42 | 6.69E-41 | Up |
| MAPK10 | 1.315962247 | 4.164693262 | 7.21E-18 | 4.54E-17 | Up |
| RBMS3 | 1.315562524 | 3.526427516 | 1.13E-24 | 1.14E-23 | Up |
| DOK7 | 1.314459086 | 3.563444856 | 3.83E-15 | 1.99E-14 | Up |
| C6orf59 | 1.314286609 | 1.38559291 | 1.07E-23 | 1.01E-22 | Up |
| MYADM | 1.313993028 | 7.976880009 | 2.15E-47 | 9.68E-46 | Up |
| GFOD1 | 1.3137428 | 3.44567136 | 1.23E-37 | 2.99E-36 | Up |
| COL4A5 | 1.312373012 | 5.212239193 | 1.55E-14 | 7.69E-14 | Up |
| CCK | 1.310215834 | 0.737310943 | 4.51E-12 | 1.84E-11 | Up |
| GUCY1A2 | 1.309378497 | 3.0777312 | 3.23E-28 | 4.21E-27 | Up |
| C9orf125 | 1.308641039 | 4.308630246 | 2.91E-14 | 1.42E-13 | Up |
| ELF5 | 1.308511734 | 4.495932398 | 2.01E-08 | 6.00E-08 | Up |
| PCDH20 | 1.308314909 | 4.586434169 | 1.92E-08 | 5.72E-08 | Up |
| COL13A1 | 1.308008902 | 3.986342947 | 7.75E-19 | 5.24E-18 | Up |
| KCNRG | 1.307896315 | 2.638871971 | 4.12E-18 | 2.65E-17 | Up |
| TMEM204 | 1.307173294 | 5.572315531 | 3.29E-46 | 1.40E-44 | Up |
| NOTCH4 | 1.306753609 | 6.137919763 | 3.75E-46 | 1.59E-44 | Up |
| C6orf97 | 1.306601656 | 4.558796417 | 1.12E-15 | 6.03E-15 | Up |
| NCF2 | 1.306263591 | 6.637392124 | 4.57E-33 | 8.23E-32 | Up |
| LIMCH1 | 1.305306442 | 7.927960946 | 2.50E-35 | 5.26E-34 | Up |
| PLCE1 | 1.304972624 | 5.424302884 | 9.55E-26 | 1.06E-24 | Up |
| PAK7 | 1.304344511 | 0.437555872 | 1.06E-21 | 8.83E-21 | Up |
| EPHB6 | 1.303610028 | 4.384073673 | 3.27E-17 | 1.96E-16 | Up |
| FLJ34503 | 1.302563981 | 0.149205875 | 1.17E-37 | 2.87E-36 | Up |
| TMTC1 | 1.302410392 | 5.23932971 | 2.15E-19 | 1.51E-18 | Up |
| ADARB1 | 1.301961716 | 6.092157503 | 8.23E-49 | 4.03E-47 | Up |
| GIMAP1 | 1.301331553 | 4.739430376 | 1.40E-37 | 3.40E-36 | Up |
| FAR2 | 1.298429748 | 4.244656815 | 6.93E-24 | 6.63E-23 | Up |

| | | | | | |
|---|---|---|---|---|---|
| REM1 | 1.298158697 | 2.982580578 | 1.62E-29 | 2.31E-28 | Up |
| ANO5 | 1.298083758 | 3.928502475 | 1.57E-12 | 6.67E-12 | Up |
| GYPC | 1.298060007 | 5.968201639 | 3.11E-44 | 1.16E-42 | Up |
| KNDC1 | 1.298018876 | 4.687696383 | 7.20E-09 | 2.23E-08 | Up |
| TMEM35 | 1.297155236 | 3.002125345 | 7.88E-14 | 3.70E-13 | Up |
| SPINLW1 | 1.296378161 | 0.74909503 | 1.29E-13 | 5.96E-13 | Up |
| IL3RA | 1.296219841 | 5.186202127 | 8.85E-48 | 4.04E-46 | Up |
| CBX7 | 1.296199631 | 6.460316519 | 7.49E-44 | 2.73E-42 | Up |
| VGLL3 | 1.295968406 | 5.861339281 | 1.55E-25 | 1.67E-24 | Up |
| P2RY12 | 1.294717655 | 2.789467671 | 1.42E-13 | 6.57E-13 | Up |
| ADAM33 | 1.293990434 | 3.269303915 | 4.20E-15 | 2.17E-14 | Up |
| SYNPO | 1.293929245 | 7.804643864 | 4.62E-45 | 1.84E-43 | Up |
| SIGLEC16 | 1.293514547 | 2.060111509 | 1.36E-18 | 9.07E-18 | Up |
| HSPB7 | 1.293492573 | 4.292185955 | 2.70E-23 | 2.48E-22 | Up |
| CYP2A6 | 1.293167234 | 1.329801626 | 1.70E-08 | 5.09E-08 | Up |
| MYL9 | 1.292961985 | 8.065340431 | 5.17E-43 | 1.78E-41 | Up |
| C1QB | 1.29268205 | 8.331503472 | 5.87E-24 | 5.65E-23 | Up |
| HPR | 1.29258027 | 1.258577084 | 1.69E-10 | 5.99E-10 | Up |
| WNT11 | 1.292560471 | 3.356681117 | 3.20E-15 | 1.67E-14 | Up |
| SAMD5 | 1.29217227 | 4.603692015 | 1.09E-18 | 7.28E-18 | Up |
| CYP2B7P1 | 1.291355928 | 6.933528254 | 9.90E-08 | 2.77E-07 | Up |
| JAKMIP2 | 1.291033967 | 3.356510932 | 2.03E-15 | 1.08E-14 | Up |
| C11orf92 | 1.28990849 | 5.077588691 | 2.71E-11 | 1.03E-10 | Up |
| CHRM3 | 1.287959692 | 2.265423906 | 3.00E-10 | 1.04E-09 | Up |
| TARP | 1.287929999 | 2.680293932 | 7.26E-17 | 4.24E-16 | Up |
| LRRIQ1 | 1.287878031 | 3.089243938 | 6.69E-10 | 2.26E-09 | Up |
| DDO | 1.286049669 | 3.553666712 | 1.18E-20 | 9.06E-20 | Up |
| LRRC4 | 1.285928493 | 4.778355592 | 2.38E-16 | 1.35E-15 | Up |
| RMST | 1.285304379 | 0.29281824 | 5.53E-30 | 8.12E-29 | Up |
| PRELP | 1.285187671 | 7.236492231 | 1.93E-21 | 1.57E-20 | Up |
| CYP3A5 | 1.284844689 | 3.323116339 | 9.10E-08 | 2.55E-07 | Up |
| AKAP12 | 1.284650879 | 6.485235957 | 2.53E-17 | 1.53E-16 | Up |
| ACP5 | 1.284464869 | 7.736700106 | 3.13E-38 | 7.91E-37 | Up |
| SORBS1 | 1.284200195 | 6.090141068 | 2.30E-33 | 4.24E-32 | Up |
| SYNC | 1.282591776 | 4.983567736 | 3.01E-22 | 2.60E-21 | Up |
| NEK5 | 1.282358822 | 1.281232392 | 2.43E-11 | 9.29E-11 | Up |
| FLI1 | 1.282291676 | 6.047064653 | 1.87E-46 | 8.03E-45 | Up |
| KIF19 | 1.281512235 | 3.141401446 | 5.19E-09 | 1.62E-08 | Up |
| C20orf46 | 1.281415853 | 3.628462752 | 7.44E-27 | 8.82E-26 | Up |
| CETP | 1.280662831 | 2.879210582 | 1.80E-22 | 1.57E-21 | Up |
| EFR3B | 1.279694053 | 4.867411843 | 8.49E-17 | 4.92E-16 | Up |
| NFASC | 1.279535603 | 4.656093004 | 8.19E-20 | 5.95E-19 | Up |
| TKTL1 | 1.279003511 | 0.860577843 | 3.62E-09 | 1.15E-08 | Up |
| NDRG2 | 1.278942718 | 6.534760046 | 4.70E-30 | 6.94E-29 | Up |
| C9orf135 | 1.278207146 | 2.218525445 | 5.57E-06 | 1.33E-05 | Up |
| C11orf21 | 1.276761715 | 2.853125995 | 1.91E-19 | 1.35E-18 | Up |

| | | | | | |
|---|---|---|---|---|---|
| PCDH17 | 1.276544133 | 5.846491568 | 1.05E-34 | 2.13E-33 | Up |
| TGFBR2 | 1.276107064 | 8.237008012 | 5.59E-49 | 2.76E-47 | Up |
| ICAM4 | 1.275983961 | 4.946457705 | 2.04E-12 | 8.55E-12 | Up |
| CCNA1 | 1.275641721 | 2.072220471 | 2.33E-08 | 6.91E-08 | Up |
| LRRTM2 | 1.275541721 | 1.613498944 | 5.71E-24 | 5.51E-23 | Up |
| EGR2 | 1.274539606 | 5.549338127 | 5.94E-27 | 7.09E-26 | Up |
| FRY | 1.273922475 | 6.297614202 | 7.08E-36 | 1.55E-34 | Up |
| SYP | 1.273350412 | 3.840014403 | 2.37E-19 | 1.67E-18 | Up |
| TOX2 | 1.273306593 | 4.812687776 | 2.07E-30 | 3.12E-29 | Up |
| SFRP1 | 1.272545851 | 3.820049876 | 3.24E-09 | 1.03E-08 | Up |
| GCNT4 | 1.271662361 | 3.095258833 | 2.67E-17 | 1.61E-16 | Up |
| DPT | 1.27140873 | 5.862448478 | 1.05E-16 | 6.06E-16 | Up |
| CCRL2 | 1.270806698 | 4.967781367 | 1.10E-30 | 1.68E-29 | Up |
| C1QA | 1.270748165 | 8.210742929 | 2.60E-25 | 2.77E-24 | Up |
| SELENBP1 | 1.270678281 | 7.998842626 | 6.19E-19 | 4.23E-18 | Up |
| C4orf22 | 1.270660553 | 0.610841639 | 1.91E-13 | 8.74E-13 | Up |
| DOCK4 | 1.270542292 | 6.297848902 | 3.05E-50 | 1.68E-48 | Up |
| TMEM132D | 1.269262916 | 2.189159184 | 1.20E-06 | 3.04E-06 | Up |
| CCL14-CCL15 | 1.268682546 | 1.022993499 | 3.43E-11 | 1.29E-10 | Up |
| DOK6 | 1.267669183 | 3.923318218 | 1.05E-20 | 8.16E-20 | Up |
| RSPH9 | 1.267564595 | 2.948647134 | 5.46E-12 | 2.21E-11 | Up |
| C14orf49 | 1.267314917 | 3.274754527 | 1.26E-23 | 1.19E-22 | Up |
| KBTBD10 | 1.267142254 | 2.492109749 | 6.07E-23 | 5.46E-22 | Up |
| JPH2 | 1.264607752 | 3.656080152 | 7.81E-21 | 6.11E-20 | Up |
| OLFML1 | 1.264039405 | 5.225825197 | 7.32E-35 | 1.49E-33 | Up |
| KLF6 | 1.263614315 | 8.114746425 | 2.74E-51 | 1.61E-49 | Up |
| PLCB4 | 1.262594719 | 4.424002167 | 3.34E-11 | 1.26E-10 | Up |
| MAP6 | 1.26130199 | 4.650325027 | 3.54E-21 | 2.83E-20 | Up |
| CRISP3 | 1.260742653 | 1.185962424 | 4.52E-07 | 1.19E-06 | Up |
| AFAP1L1 | 1.260128223 | 5.513405507 | 9.65E-45 | 3.70E-43 | Up |
| C8orf79 | 1.260099235 | 3.661720001 | 3.07E-19 | 2.14E-18 | Up |
| PROS1 | 1.260071237 | 6.790427769 | 6.73E-34 | 1.29E-32 | Up |
| ZFP36 | 1.259758259 | 8.368230151 | 5.00E-27 | 6.02E-26 | Up |
| KIF6 | 1.259117682 | 2.7437387 | 1.27E-11 | 4.99E-11 | Up |
| FAT4 | 1.258423505 | 5.574652915 | 1.42E-23 | 1.33E-22 | Up |
| LOC400804 | 1.25800578 | 0.01750028 | 3.39E-49 | 1.72E-47 | Up |
| PRMT8 | 1.257704571 | 2.11177926 | 2.64E-07 | 7.10E-07 | Up |
| C12orf69 | 1.257227075 | 2.305365595 | 5.06E-22 | 4.31E-21 | Up |
| SESN1 | 1.256539209 | 5.982285648 | 2.51E-52 | 1.56E-50 | Up |
| CNTN4 | 1.256241304 | 3.693313058 | 1.19E-16 | 6.83E-16 | Up |
| TRPC2 | 1.255513047 | 0.97514742 | 3.33E-16 | 1.87E-15 | Up |
| TPRG1 | 1.253894717 | 3.55508573 | 1.69E-24 | 1.69E-23 | Up |
| F8 | 1.253356398 | 5.852702272 | 4.66E-44 | 1.72E-42 | Up |
| PTX3 | 1.252868732 | 3.437140325 | 1.40E-16 | 8.04E-16 | Up |
| RYR2 | 1.252843985 | 3.65249827 | 2.06E-15 | 1.09E-14 | Up |
| PRDM16 | 1.252569365 | 5.067457708 | 6.49E-10 | 2.20E-09 | Up |

| | | | | | |
|---|---|---|---|---|---|
| KAZ | 1.252084765 | 5.281935381 | 1.06E-28 | 1.43E-27 | Up |
| EGR1 | 1.25149047 | 8.194333706 | 2.13E-21 | 1.73E-20 | Up |
| C4orf38 | 1.251255257 | 2.061869814 | 1.04E-29 | 1.49E-28 | Up |
| C18orf16 | 1.251252621 | 1.096973478 | 2.61E-13 | 1.18E-12 | Up |
| ACR | 1.251078466 | 0.745691401 | 3.19E-24 | 3.14E-23 | Up |
| HOXA5 | 1.250898077 | 3.978158653 | 1.45E-29 | 2.07E-28 | Up |
| PKDCC | 1.25044424 | 5.300586973 | 3.45E-22 | 2.97E-21 | Up |
| RCAN1 | 1.250326645 | 6.629218759 | 1.21E-51 | 7.34E-50 | Up |
| FGF18 | 1.24947038 | 2.586969362 | 2.45E-12 | 1.02E-11 | Up |
| GPR116 | 1.248264445 | 8.17405472 | 9.95E-17 | 5.75E-16 | Up |
| LHX9 | 1.248257601 | 1.87667945 | 5.10E-08 | 1.47E-07 | Up |
| B3GALT2 | 1.247814595 | 3.223946924 | 5.16E-12 | 2.09E-11 | Up |
| NFIX | 1.247727621 | 7.224313915 | 1.55E-27 | 1.93E-26 | Up |
| PTPLA | 1.247403603 | 4.167378869 | 2.30E-15 | 1.22E-14 | Up |
| RECK | 1.247266311 | 5.287798286 | 5.60E-42 | 1.82E-40 | Up |
| C11orf16 | 1.24712753 | 2.346734441 | 3.49E-07 | 9.27E-07 | Up |
| SERPINB2 | 1.247042485 | 1.383687821 | 1.40E-08 | 4.24E-08 | Up |
| ATF3 | 1.246628583 | 6.76299527 | 1.44E-21 | 1.19E-20 | Up |
| MYO1A | 1.245994159 | 1.501503618 | 5.63E-10 | 1.92E-09 | Up |
| KCNH6 | 1.244437284 | 0.759729431 | 3.14E-11 | 1.19E-10 | Up |
| BCL6B | 1.244215726 | 5.52024537 | 9.59E-45 | 3.68E-43 | Up |
| FLJ26850 | 1.243893376 | 0.575591837 | 1.25E-15 | 6.70E-15 | Up |
| ENKUR | 1.243557457 | 3.139388415 | 1.22E-08 | 3.70E-08 | Up |
| MS4A14 | 1.243171934 | 3.669601017 | 7.20E-22 | 6.06E-21 | Up |
| ANKRD20A3 | 1.242579004 | 1.406872098 | 1.85E-18 | 1.22E-17 | Up |
| MICALCL | 1.242310363 | 4.250501958 | 9.65E-19 | 6.49E-18 | Up |
| MNDA | 1.241740959 | 5.776298146 | 8.90E-23 | 7.92E-22 | Up |
| S100A8 | 1.241652915 | 5.609830476 | 1.74E-11 | 6.74E-11 | Up |
| C21orf128 | 1.240589928 | 1.591593864 | 1.78E-10 | 6.31E-10 | Up |
| DPYSL2 | 1.240039336 | 8.021721 | 1.68E-39 | 4.65E-38 | Up |
| MYRIP | 1.240016046 | 3.266584975 | 2.30E-11 | 8.83E-11 | Up |
| NKD1 | 1.239392271 | 3.659575613 | 3.87E-15 | 2.00E-14 | Up |
| PTN | 1.239371638 | 4.542797193 | 2.08E-13 | 9.48E-13 | Up |
| FAM180A | 1.238632571 | 2.64202871 | 1.86E-17 | 1.14E-16 | Up |
| PPP1R15A | 1.238397968 | 7.486384363 | 3.17E-56 | 2.63E-54 | Up |
| NES | 1.23672351 | 6.85634617 | 5.61E-30 | 8.23E-29 | Up |
| SPTBN1 | 1.236687956 | 9.013490849 | 2.56E-62 | 3.43E-60 | Up |
| SLCO2B1 | 1.235760824 | 7.409401547 | 2.22E-25 | 2.37E-24 | Up |
| MYOZ3 | 1.235524458 | 2.54682242 | 1.18E-23 | 1.11E-22 | Up |
| ZDHHC15 | 1.235094881 | 3.505535012 | 5.36E-15 | 2.74E-14 | Up |
| RUNX1T1 | 1.23459716 | 3.12815859 | 9.36E-19 | 6.30E-18 | Up |
| ESYT3 | 1.233936869 | 5.216950235 | 3.23E-14 | 1.56E-13 | Up |
| LHFP | 1.233927583 | 6.302905899 | 5.55E-49 | 2.75E-47 | Up |
| PDPN | 1.233156839 | 6.164330388 | 1.10E-21 | 9.10E-21 | Up |
| FAM105A | 1.232213609 | 6.503368642 | 6.54E-23 | 5.87E-22 | Up |
| GPR182 | 1.231851896 | 1.004715812 | 8.46E-25 | 8.64E-24 | Up |

| | | | | | |
|---|---|---|---|---|---|
| BEX5 | 1.228421736 | 3.728566261 | 8.37E-14 | 3.92E-13 | Up |
| KIAA1751 | 1.227709109 | 1.692167312 | 4.34E-09 | 1.37E-08 | Up |
| SGK269 | 1.227041733 | 6.862310303 | 2.15E-58 | 2.06E-56 | Up |
| LYZ | 1.226857987 | 9.201266621 | 2.35E-15 | 1.24E-14 | Up |
| CTGF | 1.22685218 | 8.137542547 | 1.23E-25 | 1.34E-24 | Up |
| ADAM29 | 1.226462643 | 0.671219372 | 1.40E-17 | 8.66E-17 | Up |
| LRRC67 | 1.225615758 | 0.480941511 | 3.54E-15 | 1.84E-14 | Up |
| SHE | 1.225400234 | 5.986298231 | 3.53E-14 | 1.70E-13 | Up |
| C16orf89 | 1.224727798 | 7.4704333 | 2.27E-06 | 5.62E-06 | Up |
| GBP7 | 1.224274846 | 0.286278855 | 2.75E-33 | 5.04E-32 | Up |
| APLN | 1.223999344 | 5.298213487 | 2.80E-18 | 1.82E-17 | Up |
| LPPR1 | 1.223572103 | 3.713097715 | 3.06E-07 | 8.19E-07 | Up |
| CSF3R | 1.223309119 | 6.52939868 | 3.64E-16 | 2.04E-15 | Up |
| PCDH12 | 1.222391846 | 5.874380915 | 1.53E-39 | 4.24E-38 | Up |
| TTC16 | 1.222185855 | 2.317267131 | 5.40E-12 | 2.19E-11 | Up |
| CDKL1 | 1.221991175 | 3.228492825 | 5.04E-21 | 3.99E-20 | Up |
| UNC80 | 1.221864063 | 0.784114207 | 3.86E-15 | 2.00E-14 | Up |
| RASGRP2 | 1.221434056 | 4.549706339 | 4.24E-20 | 3.14E-19 | Up |
| TSPAN32 | 1.220862097 | 2.930886063 | 5.47E-17 | 3.22E-16 | Up |
| C1orf183 | 1.220677932 | 3.78973757 | 8.96E-37 | 2.07E-35 | Up |
| COL4A4 | 1.220491149 | 6.059354589 | 1.32E-15 | 7.09E-15 | Up |
| LST1 | 1.21953191 | 5.419583571 | 4.52E-24 | 4.40E-23 | Up |
| CACHD1 | 1.218530547 | 5.714793803 | 6.84E-15 | 3.47E-14 | Up |
| PTGIS | 1.218505205 | 6.033529928 | 8.08E-19 | 5.46E-18 | Up |
| CCDC69 | 1.218216127 | 6.800563388 | 1.12E-34 | 2.27E-33 | Up |
| WFS1 | 1.217176039 | 7.192308656 | 4.89E-57 | 4.23E-55 | Up |
| ITGA10 | 1.217164359 | 3.96460588 | 6.00E-17 | 3.52E-16 | Up |
| RD3 | 1.217049568 | 0.452824217 | 2.67E-23 | 2.45E-22 | Up |
| SRRM4 | 1.217027471 | 0.367425171 | 6.15E-16 | 3.37E-15 | Up |
| GNMT | 1.216106549 | 2.001494628 | 1.23E-13 | 5.71E-13 | Up |
| PAPSS2 | 1.215578034 | 7.349382959 | 4.58E-33 | 8.24E-32 | Up |
| PILRA | 1.215183156 | 5.349248306 | 5.02E-28 | 6.46E-27 | Up |
| DDX43 | 1.215095637 | 2.205027066 | 3.09E-06 | 7.57E-06 | Up |
| GJA5 | 1.215041693 | 6.392268013 | 1.68E-29 | 2.38E-28 | Up |
| BMP5 | 1.214827979 | 4.419359517 | 1.33E-09 | 4.38E-09 | Up |
| APOB48R | 1.214632767 | 5.873079893 | 1.57E-27 | 1.96E-26 | Up |
| PCDHGA12 | 1.213689144 | 3.661888586 | 2.26E-20 | 1.71E-19 | Up |
| FOXA2 | 1.212966518 | 5.718456621 | 1.88E-07 | 5.13E-07 | Up |
| SHC3 | 1.212398024 | 3.376083089 | 1.13E-12 | 4.83E-12 | Up |
| SIGLEC5 | 1.211985566 | 3.961111716 | 3.74E-21 | 2.98E-20 | Up |
| ABCB1 | 1.211259439 | 3.927657478 | 2.68E-20 | 2.02E-19 | Up |
| FAM23A | 1.209841137 | 3.15897328 | 6.81E-16 | 3.72E-15 | Up |
| ADARB2 | 1.209815419 | 1.058789264 | 3.80E-15 | 1.97E-14 | Up |
| RNF128 | 1.209616771 | 5.696445065 | 1.02E-09 | 3.40E-09 | Up |
| FAM81B | 1.209231146 | 3.35273015 | 1.37E-07 | 3.78E-07 | Up |
| WNT2B | 1.208367107 | 2.716575101 | 1.71E-28 | 2.27E-27 | Up |

| | | | | | |
|---|---|---|---|---|---|
| ARHGEF37 | 1.207094069 | 4.949930487 | 1.04E-21 | 8.67E-21 | Up |
| LOC572558 | 1.207006144 | 0.166578108 | 1.21E-39 | 3.38E-38 | Up |
| TBX5 | 1.206704454 | 5.015805557 | 1.45E-24 | 1.45E-23 | Up |
| LILRA6 | 1.206495971 | 4.884120006 | 2.20E-21 | 1.79E-20 | Up |
| C5orf4 | 1.205917962 | 6.440294242 | 2.45E-15 | 1.29E-14 | Up |
| LOC90586 | 1.20566238 | 0.904189239 | 3.80E-25 | 3.98E-24 | Up |
| WDR17 | 1.205378896 | 3.252032633 | 9.80E-12 | 3.89E-11 | Up |
| NEXN | 1.20508229 | 5.389137812 | 1.74E-31 | 2.86E-30 | Up |
| FZD4 | 1.204398583 | 6.543311293 | 3.43E-40 | 9.82E-39 | Up |
| PCDP1 | 1.203728497 | 4.816404262 | 7.66E-08 | 2.16E-07 | Up |
| ITIH2 | 1.203624192 | 2.098492685 | 1.67E-05 | 3.81E-05 | Up |
| PLEKHH2 | 1.203076565 | 5.779513752 | 4.72E-22 | 4.02E-21 | Up |
| SPI1 | 1.203072595 | 6.635743515 | 3.08E-28 | 4.04E-27 | Up |
| FOS | 1.202896833 | 8.501993365 | 5.04E-18 | 3.22E-17 | Up |
| RASSF8 | 1.202460575 | 5.971456318 | 7.00E-27 | 8.32E-26 | Up |
| CHRNA4 | 1.20196248 | 0.151893043 | 1.56E-29 | 2.22E-28 | Up |
| SULT1B1 | 1.201693488 | 0.904540893 | 5.92E-14 | 2.81E-13 | Up |
| SIGLEC14 | 1.199927645 | 4.091111918 | 9.86E-13 | 4.25E-12 | Up |
| APOLD1 | 1.199504115 | 5.953360027 | 5.56E-30 | 8.16E-29 | Up |
| PRICKLE1 | 1.199493032 | 5.169346981 | 7.54E-27 | 8.93E-26 | Up |
| CNN1 | 1.199147662 | 5.377726827 | 5.39E-19 | 3.70E-18 | Up |
| MDH1B | 1.198179652 | 2.638277516 | 1.06E-10 | 3.83E-10 | Up |
| FAM70A | 1.196945187 | 3.866778344 | 1.15E-15 | 6.18E-15 | Up |
| PHACTR2 | 1.196377561 | 6.599361688 | 1.00E-50 | 5.74E-49 | Up |
| LMOD3 | 1.196253202 | 1.832511732 | 8.59E-27 | 1.01E-25 | Up |
| CD33 | 1.195306878 | 4.612505964 | 5.89E-23 | 5.30E-22 | Up |
| PKD2L1 | 1.195274824 | 1.815928705 | 6.06E-13 | 2.66E-12 | Up |
| WNT9A | 1.195096143 | 3.111882538 | 1.26E-20 | 9.70E-20 | Up |
| CYR61 | 1.194921565 | 8.083980355 | 9.03E-24 | 8.59E-23 | Up |
| DCN | 1.194406343 | 8.842595966 | 9.45E-24 | 8.97E-23 | Up |
| DMRT2 | 1.193669384 | 1.835117453 | 4.57E-09 | 1.44E-08 | Up |
| TMEM220 | 1.191895547 | 4.362221307 | 1.19E-26 | 1.40E-25 | Up |
| CMA1 | 1.190285126 | 0.475554537 | 3.15E-17 | 1.89E-16 | Up |
| LMCD1 | 1.189348118 | 6.352130901 | 1.10E-44 | 4.16E-43 | Up |
| RPL23AP32 | 1.188513455 | 0.289654433 | 1.32E-26 | 1.54E-25 | Up |
| CDKL5 | 1.188038073 | 3.433748584 | 4.60E-20 | 3.41E-19 | Up |
| KLF9 | 1.187789695 | 7.026636098 | 7.39E-42 | 2.38E-40 | Up |
| P2RY14 | 1.187311587 | 4.192276685 | 6.39E-28 | 8.17E-27 | Up |
| TTLL6 | 1.186343601 | 2.117109476 | 7.87E-09 | 2.43E-08 | Up |
| SPAG8 | 1.186233117 | 2.81075452 | 1.51E-14 | 7.51E-14 | Up |
| LOC339524 | 1.185067784 | 3.867974218 | 9.64E-26 | 1.06E-24 | Up |
| ADPRH | 1.184430849 | 5.806967281 | 9.31E-54 | 6.23E-52 | Up |
| PDZD4 | 1.184214509 | 3.825195848 | 3.59E-15 | 1.86E-14 | Up |
| KIR2DL1 | 1.184112906 | 0.518656376 | 1.29E-13 | 5.95E-13 | Up |
| SLC1A2 | 1.184042417 | 3.382194645 | 2.25E-13 | 1.02E-12 | Up |
| IFLTD1 | 1.183330536 | 0.421056247 | 1.42E-15 | 7.57E-15 | Up |

| | | | | | |
|---|---|---|---|---|---|
| NAP1L2 | 1.182725808 | 3.37333196 | 3.25E-14 | 1.57E-13 | Up |
| MOSC2 | 1.182268608 | 5.37714823 | 7.64E-33 | 1.36E-31 | Up |
| SLC10A2 | 1.18073662 | 0.601603203 | 3.46E-09 | 1.10E-08 | Up |
| LRRC46 | 1.180555652 | 4.105558527 | 4.32E-11 | 1.61E-10 | Up |
| BTK | 1.180012983 | 5.488908277 | 3.47E-24 | 3.40E-23 | Up |
| CDKN2B | 1.179623422 | 6.075308635 | 6.80E-21 | 5.34E-20 | Up |
| NPY5R | 1.179225957 | 0.256133328 | 1.15E-20 | 8.89E-20 | Up |
| PAX6 | 1.178420737 | 3.171863555 | 2.58E-42 | 8.54E-41 | Up |
| CYP4Z2P | 1.17790113 | 0.78502657 | 2.37E-13 | 1.07E-12 | Up |
| MGAM | 1.177842765 | 2.078249113 | 5.04E-12 | 2.05E-11 | Up |
| CXorf30 | 1.176759165 | 1.100911026 | 7.73E-09 | 2.38E-08 | Up |
| AK7 | 1.176354 | 3.938124098 | 5.04E-13 | 2.22E-12 | Up |
| FBP1 | 1.1753988 | 7.590484184 | 1.36E-27 | 1.70E-26 | Up |
| RSPH1 | 1.175211768 | 4.344908192 | 9.21E-10 | 3.07E-09 | Up |
| RASL12 | 1.175170256 | 5.494673395 | 7.02E-33 | 1.25E-31 | Up |
| RFX2 | 1.174841206 | 5.381205963 | 1.91E-27 | 2.36E-26 | Up |
| AR | 1.174701154 | 2.658510875 | 2.20E-10 | 7.74E-10 | Up |
| PTPRM | 1.174596929 | 6.812465997 | 6.10E-35 | 1.25E-33 | Up |
| MFNG | 1.174409062 | 5.798287232 | 7.55E-42 | 2.43E-40 | Up |
| ACTG2 | 1.17415876 | 5.485840318 | 7.90E-15 | 3.99E-14 | Up |
| PHEX | 1.173537785 | 2.168494442 | 1.75E-12 | 7.37E-12 | Up |
| TWIST2 | 1.17220802 | 2.611003347 | 4.25E-17 | 2.52E-16 | Up |
| EGR3 | 1.171761599 | 4.920801066 | 1.59E-17 | 9.75E-17 | Up |
| ZEB2 | 1.171369259 | 6.62360972 | 3.24E-36 | 7.27E-35 | Up |
| PTPRD | 1.170483013 | 4.58735609 | 1.15E-18 | 7.66E-18 | Up |
| OXGR1 | 1.16908477 | 0.737026142 | 2.88E-13 | 1.29E-12 | Up |
| CALB2 | 1.168989651 | 2.636455182 | 4.84E-07 | 1.27E-06 | Up |
| CYBB | 1.168062753 | 7.536858333 | 3.13E-19 | 2.18E-18 | Up |
| PTPLAD2 | 1.16798567 | 5.910446656 | 1.32E-37 | 3.21E-36 | Up |
| WFDC6 | 1.166655179 | 0.482859544 | 1.61E-14 | 8.00E-14 | Up |
| C2orf65 | 1.166298766 | 2.24515651 | 1.34E-15 | 7.20E-15 | Up |
| DLEC1 | 1.164766111 | 3.589743361 | 2.01E-08 | 5.99E-08 | Up |
| NOTUM | 1.164533565 | 3.687041828 | 4.16E-07 | 1.10E-06 | Up |
| INE2 | 1.163968931 | 1.301366216 | 4.18E-11 | 1.56E-10 | Up |
| CLC | 1.163331065 | 0.672315715 | 3.82E-10 | 1.32E-09 | Up |
| CCDC13 | 1.163131699 | 2.452994123 | 1.05E-16 | 6.08E-16 | Up |
| GIMAP4 | 1.162818276 | 6.435184229 | 6.00E-35 | 1.24E-33 | Up |
| LRRC4B | 1.162462513 | 3.329684994 | 2.86E-21 | 2.31E-20 | Up |
| CXorf22 | 1.161902588 | 0.8077241 | 2.71E-11 | 1.03E-10 | Up |
| USP44 | 1.161667101 | 3.952659224 | 7.63E-12 | 3.06E-11 | Up |
| CD83 | 1.161635933 | 6.300955373 | 4.27E-31 | 6.82E-30 | Up |
| C10orf54 | 1.16161075 | 6.962540094 | 4.64E-48 | 2.17E-46 | Up |
| RRAD | 1.160763324 | 5.799380311 | 6.51E-11 | 2.39E-10 | Up |
| C2orf54 | 1.160513661 | 5.368725068 | 1.17E-10 | 4.21E-10 | Up |
| SLC22A3 | 1.160315962 | 6.098561831 | 1.20E-09 | 3.96E-09 | Up |
| CD34 | 1.159695996 | 6.847834037 | 1.15E-42 | 3.89E-41 | Up |

| | | | | | |
|---|---|---|---|---|---|
| ARHGEF6 | 1.159445351 | 6.189853444 | 1.21E-34 | 2.44E-33 | Up |
| CXorf36 | 1.15931685 | 5.570970066 | 5.39E-36 | 1.19E-34 | Up |
| GGTA1 | 1.15856407 | 5.044182612 | 2.63E-25 | 2.81E-24 | Up |
| DOCK11 | 1.158371885 | 6.066285534 | 3.80E-30 | 5.65E-29 | Up |
| ETV1 | 1.158299709 | 6.479690393 | 1.20E-20 | 9.21E-20 | Up |
| TCTEX1D4 | 1.157121017 | 1.918406981 | 8.26E-15 | 4.17E-14 | Up |
| ALOX15B | 1.156921934 | 6.451023171 | 1.33E-08 | 4.02E-08 | Up |
| KCNJ8 | 1.156753952 | 5.505996177 | 5.23E-25 | 5.42E-24 | Up |
| UST | 1.155738823 | 4.927413959 | 5.45E-19 | 3.73E-18 | Up |
| NMUR2 | 1.154943975 | 0.552642246 | 1.60E-12 | 6.79E-12 | Up |
| GRM3 | 1.154641158 | 0.47090153 | 4.19E-23 | 3.80E-22 | Up |
| SYPL2 | 1.153926177 | 2.703661088 | 8.85E-18 | 5.53E-17 | Up |
| KCNB1 | 1.153095166 | 0.971395127 | 1.56E-10 | 5.55E-10 | Up |
| CD302 | 1.152965545 | 7.189593627 | 1.96E-30 | 2.97E-29 | Up |
| KANK2 | 1.15199609 | 7.485208383 | 1.01E-60 | 1.19E-58 | Up |
| VIP | 1.151887097 | 0.57420103 | 5.46E-20 | 4.01E-19 | Up |
| SLC7A7 | 1.151634031 | 6.549313302 | 1.64E-30 | 2.50E-29 | Up |
| FAM71A | 1.151501814 | 0.127797069 | 6.17E-28 | 7.91E-27 | Up |
| ADRA2C | 1.15116371 | 2.989180251 | 1.30E-11 | 5.10E-11 | Up |
| C9orf117 | 1.150423743 | 2.706880462 | 4.00E-09 | 1.26E-08 | Up |
| PTPN13 | 1.150348939 | 6.867292046 | 3.55E-13 | 1.59E-12 | Up |
| OR7E37P | 1.148993528 | 0.120208063 | 1.23E-26 | 1.44E-25 | Up |
| LY86 | 1.148888987 | 5.153030478 | 1.63E-21 | 1.34E-20 | Up |
| TYROBP | 1.148835636 | 7.2097007 | 6.43E-25 | 6.62E-24 | Up |
| RASGEF1B | 1.148072734 | 5.679307416 | 8.32E-41 | 2.49E-39 | Up |
| BMP6 | 1.147908353 | 4.876486055 | 1.33E-10 | 4.75E-10 | Up |
| NHLRC4 | 1.147403614 | 3.325071165 | 9.41E-19 | 6.32E-18 | Up |
| DHDPSL | 1.147035705 | 2.162006387 | 1.69E-11 | 6.57E-11 | Up |
| PDE4C | 1.147012365 | 4.372817805 | 9.09E-13 | 3.93E-12 | Up |
| TDRD9 | 1.146794421 | 2.602393869 | 5.60E-08 | 1.61E-07 | Up |
| GJC2 | 1.144766191 | 3.432338361 | 3.07E-25 | 3.26E-24 | Up |
| CSTA | 1.143331351 | 4.779500464 | 5.22E-18 | 3.33E-17 | Up |
| ENG | 1.14282192 | 8.055595374 | 1.50E-43 | 5.36E-42 | Up |
| HPGD | 1.142362779 | 6.983774521 | 3.27E-08 | 9.57E-08 | Up |
| RAB40AL | 1.142255974 | 1.540630744 | 1.07E-24 | 1.08E-23 | Up |
| APOC1 | 1.142044953 | 7.457552803 | 2.63E-17 | 1.59E-16 | Up |
| ADCYAP1R1 | 1.14191767 | 0.156718812 | 1.65E-29 | 2.35E-28 | Up |
| LOC400891 | 1.141815336 | 1.005389054 | 1.70E-07 | 4.65E-07 | Up |
| IL34 | 1.141399309 | 4.112920143 | 1.93E-19 | 1.36E-18 | Up |
| CLEC4D | 1.14094878 | 1.589699156 | 7.70E-13 | 3.35E-12 | Up |
| SIGLEC1 | 1.139826498 | 6.259094951 | 2.38E-17 | 1.45E-16 | Up |
| CAMK2N1 | 1.139787721 | 6.358873229 | 2.37E-17 | 1.44E-16 | Up |
| ITPRIP | 1.139662047 | 6.5049118 | 5.06E-41 | 1.54E-39 | Up |
| CEACAM3 | 1.139333702 | 1.769837996 | 4.65E-16 | 2.57E-15 | Up |
| PCDHGB6 | 1.13889982 | 3.707319055 | 2.63E-18 | 1.71E-17 | Up |
| CD68 | 1.13790154 | 8.475751599 | 8.56E-31 | 1.33E-29 | Up |

| | | | | | |
|---|---|---|---|---|---|
| FAM125B | 1.137391243 | 6.162679288 | 5.00E-36 | 1.11E-34 | Up |
| ASTN1 | 1.136991029 | 1.076429998 | 8.47E-09 | 2.60E-08 | Up |
| APOC2 | 1.136170471 | 4.966939811 | 5.64E-11 | 2.08E-10 | Up |
| FCGR3A | 1.135960918 | 7.687933998 | 1.64E-20 | 1.25E-19 | Up |
| GJA1 | 1.135269322 | 7.527850744 | 4.76E-20 | 3.52E-19 | Up |
| GBP4 | 1.135002674 | 6.943124434 | 3.98E-21 | 3.17E-20 | Up |
| NAPSA | 1.134853508 | 9.677899958 | 9.44E-06 | 2.20E-05 | Up |
| TGFB2 | 1.134635989 | 5.721744119 | 1.24E-14 | 6.18E-14 | Up |
| OR6K3 | 1.134511705 | 0.038043428 | 1.40E-56 | 1.19E-54 | Up |
| MYH10 | 1.132097163 | 7.634494986 | 1.65E-33 | 3.06E-32 | Up |
| 4-Sep | 1.131788179 | 5.129221331 | 1.65E-37 | 3.99E-36 | Up |
| LRRC10B | 1.130621038 | 3.845661011 | 5.30E-08 | 1.52E-07 | Up |
| CADM3 | 1.129985217 | 3.858130136 | 2.44E-10 | 8.55E-10 | Up |
| MCOLN3 | 1.129707158 | 4.012321492 | 2.54E-10 | 8.90E-10 | Up |
| CNRIP1 | 1.129308139 | 4.969640551 | 4.96E-39 | 1.31E-37 | Up |
| CD244 | 1.128781277 | 3.434827297 | 1.13E-19 | 8.12E-19 | Up |
| CLEC4F | 1.128678254 | 1.737984 | 8.17E-10 | 2.74E-09 | Up |
| P2RX1 | 1.128521672 | 3.989771204 | 7.09E-18 | 4.46E-17 | Up |
| SELPLG | 1.127522135 | 6.660316283 | 2.43E-29 | 3.42E-28 | Up |
| PRICKLE2 | 1.127280259 | 5.965410212 | 2.43E-35 | 5.12E-34 | Up |
| S100A4 | 1.126625348 | 7.883984171 | 1.47E-18 | 9.79E-18 | Up |
| PGLYRP1 | 1.12581493 | 0.050589782 | 5.21E-31 | 8.23E-30 | Up |
| CXCL12 | 1.12485383 | 6.487049292 | 2.15E-19 | 1.51E-18 | Up |
| GPRC5A | 1.124746809 | 8.624939272 | 6.73E-18 | 4.25E-17 | Up |
| HDC | 1.12337664 | 3.576687908 | 2.03E-11 | 7.84E-11 | Up |
| TEKT4 | 1.123354239 | 2.017313771 | 2.74E-10 | 9.57E-10 | Up |
| psiTPTE22 | 1.123108275 | 3.673512979 | 1.13E-07 | 3.13E-07 | Up |
| SYN3 | 1.122603552 | 1.744276208 | 7.07E-10 | 2.38E-09 | Up |
| COBL | 1.121933179 | 6.246677118 | 1.00E-12 | 4.31E-12 | Up |
| HLA-DRB5 | 1.121580648 | 7.975025091 | 5.22E-11 | 1.94E-10 | Up |
| PLA1A | 1.121564947 | 4.672003704 | 8.64E-14 | 4.05E-13 | Up |
| DLGAP1 | 1.121560635 | 2.076122632 | 5.21E-07 | 1.36E-06 | Up |
| SECISBP2L | 1.120873583 | 7.25292549 | 1.84E-41 | 5.80E-40 | Up |
| NAALAD2 | 1.120800823 | 2.610117835 | 3.25E-23 | 2.97E-22 | Up |
| B3GALNT1 | 1.119921815 | 5.5605682 | 1.96E-24 | 1.95E-23 | Up |
| DRD1 | 1.11987868 | 1.987835714 | 6.89E-08 | 1.95E-07 | Up |
| GRP | 1.119807503 | 2.311177057 | 1.37E-06 | 3.47E-06 | Up |
| C17orf91 | 1.119769076 | 5.305677852 | 5.69E-41 | 1.72E-39 | Up |
| CASKIN2 | 1.119611907 | 6.606111326 | 9.98E-52 | 6.06E-50 | Up |
| ACACB | 1.119055443 | 5.40845646 | 7.96E-28 | 1.02E-26 | Up |
| PLSCR4 | 1.119046967 | 6.110219298 | 6.26E-36 | 1.37E-34 | Up |
| THRB | 1.118872349 | 5.046001248 | 3.84E-16 | 2.14E-15 | Up |
| TM6SF1 | 1.118377097 | 4.869613388 | 6.33E-31 | 9.94E-30 | Up |
| NOS2 | 1.118109931 | 2.954123304 | 1.18E-11 | 4.66E-11 | Up |
| DDC | 1.117150633 | 2.755220814 | 0.000227414 | 0.000459641 | Up |
| CDH10 | 1.117096512 | 0.552994624 | 2.79E-13 | 1.26E-12 | Up |

| | | | | | |
|---|---|---|---|---|---|
| PGM5P2 | 1.115846512 | 0.88152206 | 3.87E-19 | 2.68E-18 | Up |
| NCRNA00189 | 1.115660764 | 0.669754482 | 1.10E-15 | 5.91E-15 | Up |
| FOXF2 | 1.114973239 | 4.828217432 | 5.29E-20 | 3.89E-19 | Up |
| RORB | 1.112581175 | 1.917655116 | 8.34E-09 | 2.57E-08 | Up |
| HCK | 1.112190438 | 6.423808673 | 1.92E-21 | 1.56E-20 | Up |
| CRISP2 | 1.112036503 | 1.25697932 | 3.19E-07 | 8.52E-07 | Up |
| NRG2 | 1.111254186 | 1.43803472 | 1.68E-11 | 6.51E-11 | Up |
| PDE6A | 1.110577543 | 1.459381461 | 1.38E-12 | 5.89E-12 | Up |
| PPIL6 | 1.110563364 | 4.389017042 | 1.17E-17 | 7.23E-17 | Up |
| PARK2 | 1.110326493 | 3.015830395 | 1.01E-17 | 6.30E-17 | Up |
| KCTD12 | 1.110292308 | 7.745770083 | 7.53E-31 | 1.17E-29 | Up |
| GMFG | 1.110033483 | 6.008656438 | 6.46E-31 | 1.01E-29 | Up |
| NDST1 | 1.109916104 | 7.699264712 | 5.93E-56 | 4.76E-54 | Up |
| GABRB2 | 1.109593261 | 2.874596035 | 1.09E-07 | 3.03E-07 | Up |
| RAB11FIP1 | 1.109355824 | 8.448848939 | 3.40E-33 | 6.21E-32 | Up |
| LMO7 | 1.109161613 | 8.486739698 | 1.10E-34 | 2.22E-33 | Up |
| LOC145820 | 1.10881639 | 1.705729323 | 1.12E-15 | 6.05E-15 | Up |
| C15orf51 | 1.108485297 | 0.985288455 | 3.24E-17 | 1.94E-16 | Up |
| ARHGAP24 | 1.105223443 | 5.677952406 | 2.20E-37 | 5.24E-36 | Up |
| TEPP | 1.104905128 | 1.659559519 | 1.39E-06 | 3.51E-06 | Up |
| EPB41L2 | 1.104041497 | 6.738901145 | 5.19E-34 | 1.00E-32 | Up |
| SULT1A2 | 1.102139818 | 4.267877429 | 1.79E-15 | 9.53E-15 | Up |
| PCDHGA9 | 1.101499742 | 3.338562356 | 6.38E-16 | 3.50E-15 | Up |
| PRRG3 | 1.100889849 | 0.554984668 | 6.68E-19 | 4.56E-18 | Up |
| CARD16 | 1.099761106 | 5.301829307 | 4.45E-25 | 4.64E-24 | Up |
| GALNTL4 | 1.099669605 | 6.249185436 | 2.77E-32 | 4.80E-31 | Up |
| SCN11A | 1.09962805 | 1.143719118 | 2.23E-16 | 1.27E-15 | Up |
| LILRB3 | 1.099510591 | 5.034288994 | 3.46E-24 | 3.40E-23 | Up |
| CD163 | 1.097606697 | 7.313108123 | 6.77E-16 | 3.70E-15 | Up |
| OLIG1 | 1.096501923 | 1.328953063 | 4.39E-08 | 1.27E-07 | Up |
| UTRN | 1.095818551 | 7.657523148 | 4.22E-40 | 1.20E-38 | Up |
| LOC257358 | 1.09548337 | 0.798707796 | 1.41E-20 | 1.08E-19 | Up |
| KLRD1 | 1.095006279 | 3.543771616 | 3.71E-14 | 1.79E-13 | Up |
| APBB1 | 1.09429386 | 6.010654013 | 2.56E-30 | 3.84E-29 | Up |
| CASC1 | 1.092885164 | 2.881471886 | 4.93E-09 | 1.55E-08 | Up |
| F2RL3 | 1.092613814 | 4.544944672 | 4.39E-20 | 3.25E-19 | Up |
| GADD45B | 1.092114198 | 7.026936157 | 1.27E-28 | 1.71E-27 | Up |
| C14orf86 | 1.092082575 | 0.325148131 | 6.24E-17 | 3.66E-16 | Up |
| NEK10 | 1.090841803 | 2.053254548 | 7.20E-08 | 2.04E-07 | Up |
| KCNA3 | 1.089070434 | 4.360594528 | 9.77E-12 | 3.88E-11 | Up |
| CCL15 | 1.088810055 | 1.755523061 | 4.44E-06 | 1.07E-05 | Up |
| NKD2 | 1.088446805 | 4.97275959 | 4.11E-16 | 2.29E-15 | Up |
| CLEC9A | 1.088096896 | 1.430032285 | 1.72E-11 | 6.69E-11 | Up |
| CCND2 | 1.087349141 | 6.598549902 | 4.22E-27 | 5.12E-26 | Up |
| ABCG1 | 1.087214304 | 6.65961457 | 7.43E-30 | 1.08E-28 | Up |
| KIAA1529 | 1.087007373 | 4.173442167 | 5.69E-15 | 2.90E-14 | Up |

| | | | | | |
|---|---|---|---|---|---|
| GLT1D1 | 1.08665174 | 2.791462928 | 5.48E-13 | 2.41E-12 | Up |
| CCDC152 | 1.086065003 | 4.050722843 | 8.12E-27 | 9.60E-26 | Up |
| SCNN1B | 1.085706118 | 6.705421605 | 2.06E-09 | 6.69E-09 | Up |
| PRSS21 | 1.085164352 | 3.114995695 | 9.87E-05 | 0.000207636 | Up |
| NHLH2 | 1.085065486 | 0.788195057 | 5.10E-10 | 1.74E-09 | Up |
| PPYR1 | 1.084440527 | 2.419452709 | 5.44E-08 | 1.56E-07 | Up |
| MYLK3 | 1.08416781 | 2.346521677 | 1.23E-11 | 4.83E-11 | Up |
| IL18R1 | 1.082559469 | 4.343465054 | 1.14E-20 | 8.79E-20 | Up |
| FAM38B | 1.082006259 | 4.701255687 | 3.57E-15 | 1.86E-14 | Up |
| SULT1A1 | 1.081927164 | 6.261814589 | 5.59E-23 | 5.03E-22 | Up |
| ABCC9 | 1.081628128 | 5.429485405 | 6.78E-20 | 4.95E-19 | Up |
| PCDHAC2 | 1.080899987 | 4.505261617 | 8.52E-10 | 2.86E-09 | Up |
| RGS7BP | 1.080849429 | 2.592502516 | 3.90E-09 | 1.23E-08 | Up |
| CAB39L | 1.08073994 | 5.490321232 | 3.55E-38 | 8.91E-37 | Up |
| CST6 | 1.079232573 | 4.870117889 | 2.15E-07 | 5.83E-07 | Up |
| XKR6 | 1.07895685 | 1.389009207 | 1.28E-14 | 6.39E-14 | Up |
| CRHBP | 1.07874262 | 1.006841934 | 1.97E-17 | 1.21E-16 | Up |
| REN | 1.078533406 | 1.483087202 | 1.64E-09 | 5.38E-09 | Up |
| HYDIN | 1.078372953 | 4.241493969 | 2.82E-08 | 8.28E-08 | Up |
| SPSB4 | 1.077880574 | 1.725164003 | 1.28E-12 | 5.48E-12 | Up |
| HP | 1.077751631 | 6.083878631 | 0.00038687 | 0.000764762 | Up |
| TENC1 | 1.076370973 | 7.309934167 | 1.36E-33 | 2.55E-32 | Up |
| PRRT4 | 1.075583216 | 1.129701995 | 7.36E-11 | 2.70E-10 | Up |
| CCDC39 | 1.07472068 | 3.162740349 | 3.84E-13 | 1.71E-12 | Up |
| AGR3 | 1.074107863 | 6.246343697 | 2.72E-05 | 6.04E-05 | Up |
| CRIM1 | 1.073059615 | 7.377804753 | 2.54E-33 | 4.68E-32 | Up |
| C7orf41 | 1.072716225 | 6.626714615 | 1.76E-25 | 1.90E-24 | Up |
| PRF1 | 1.072248251 | 5.595679162 | 1.59E-17 | 9.77E-17 | Up |
| ARHGEF4 | 1.072210558 | 4.601063266 | 1.09E-09 | 3.61E-09 | Up |
| FAM95B1 | 1.072107952 | 2.830987382 | 1.74E-09 | 5.67E-09 | Up |
| TMOD1 | 1.072090881 | 5.185476709 | 5.56E-32 | 9.43E-31 | Up |
| DNAH7 | 1.072056108 | 3.363121612 | 6.86E-10 | 2.32E-09 | Up |
| APOL3 | 1.071316626 | 6.523843677 | 1.15E-25 | 1.25E-24 | Up |
| PHYHD1 | 1.071156916 | 5.304183662 | 1.92E-09 | 6.23E-09 | Up |
| TACR1 | 1.069714542 | 2.988666153 | 2.04E-10 | 7.20E-10 | Up |
| TDRD10 | 1.069587357 | 3.732157805 | 1.55E-07 | 4.26E-07 | Up |
| LCN6 | 1.069386282 | 0.133507438 | 8.96E-30 | 1.30E-28 | Up |
| DIO3 | 1.068958077 | 1.914550891 | 2.11E-09 | 6.84E-09 | Up |
| GAB1 | 1.068748369 | 6.170163002 | 1.63E-48 | 7.78E-47 | Up |
| NRN1L | 1.068657975 | 1.481889 | 6.29E-12 | 2.54E-11 | Up |
| P2RX7 | 1.068325503 | 4.275924087 | 4.54E-15 | 2.33E-14 | Up |
| PRDM5 | 1.067578961 | 3.734229028 | 3.32E-23 | 3.03E-22 | Up |
| HPCAL4 | 1.067184333 | 2.775689372 | 6.99E-07 | 1.81E-06 | Up |
| MEOX2 | 1.066891103 | 4.305614181 | 1.63E-14 | 8.06E-14 | Up |
| APOA5 | 1.066805893 | 0.255691757 | 6.71E-16 | 3.67E-15 | Up |
| MCC | 1.066756956 | 5.43430527 | 3.11E-26 | 3.56E-25 | Up |

| | | | | | |
|---|---|---|---|---|---|
| FBLN1 | 1.066650564 | 7.969050453 | 1.15E-20 | 8.88E-20 | Up |
| DAAM2 | 1.066480878 | 6.135544878 | 3.44E-14 | 1.66E-13 | Up |
| DKFZp779M0652 | 1.066184017 | 0.973556228 | 1.42E-16 | 8.13E-16 | Up |
| ST6GAL2 | 1.066125677 | 3.008201338 | 5.29E-08 | 1.52E-07 | Up |
| SIRPB2 | 1.065174553 | 4.515028613 | 7.47E-19 | 5.08E-18 | Up |
| GGT6 | 1.065026088 | 4.673053665 | 2.24E-05 | 5.03E-05 | Up |
| FMO3 | 1.064934596 | 5.174916595 | 3.01E-17 | 1.81E-16 | Up |
| RTDR1 | 1.064763064 | 1.536857008 | 1.81E-07 | 4.94E-07 | Up |
| SOX18 | 1.064298847 | 4.862709373 | 1.31E-26 | 1.53E-25 | Up |
| SLC24A3 | 1.06366538 | 5.097012763 | 2.45E-15 | 1.29E-14 | Up |
| CBFA2T3 | 1.063039713 | 4.642595419 | 1.57E-18 | 1.04E-17 | Up |
| CRTAM | 1.062995984 | 3.460584034 | 4.20E-15 | 2.17E-14 | Up |
| TSNAXIP1 | 1.062928054 | 2.47544419 | 1.05E-09 | 3.47E-09 | Up |
| VNN3 | 1.062387537 | 2.398044708 | 4.90E-07 | 1.29E-06 | Up |
| ECM2 | 1.062076093 | 5.29607415 | 1.41E-21 | 1.16E-20 | Up |
| SYNE1 | 1.061686308 | 7.390166826 | 4.10E-21 | 3.26E-20 | Up |
| WDR78 | 1.061557467 | 4.210348169 | 2.70E-12 | 1.12E-11 | Up |
| NCKAP1L | 1.061481073 | 6.532585289 | 1.06E-20 | 8.22E-20 | Up |
| ZEB1 | 1.061051693 | 6.10035385 | 4.95E-36 | 1.10E-34 | Up |
| FAM19A2 | 1.060955115 | 1.910832241 | 1.82E-15 | 9.67E-15 | Up |
| BST1 | 1.060362874 | 4.601450769 | 1.75E-25 | 1.89E-24 | Up |
| SLC31A2 | 1.060265989 | 6.090808024 | 1.83E-33 | 3.40E-32 | Up |
| WBSCR17 | 1.060017803 | 3.960582541 | 4.99E-10 | 1.71E-09 | Up |
| TSPAN12 | 1.059409112 | 6.677532343 | 2.56E-26 | 2.95E-25 | Up |
| C6orf25 | 1.059403418 | 0.058400481 | 1.23E-37 | 2.99E-36 | Up |
| CD69 | 1.059296796 | 5.164645624 | 4.22E-15 | 2.18E-14 | Up |
| NLGN4X | 1.058863239 | 3.305224156 | 3.14E-11 | 1.19E-10 | Up |
| CCDC68 | 1.058628737 | 5.239188707 | 4.21E-17 | 2.50E-16 | Up |
| OLAH | 1.058493583 | 0.569395402 | 1.42E-11 | 5.57E-11 | Up |
| KMO | 1.056863617 | 4.575159032 | 4.06E-20 | 3.02E-19 | Up |
| RGN | 1.056429413 | 4.458522265 | 4.71E-12 | 1.92E-11 | Up |
| CCDC89 | 1.056346612 | 2.659471075 | 2.96E-14 | 1.44E-13 | Up |
| SERPING1 | 1.055068036 | 8.756259379 | 2.82E-32 | 4.87E-31 | Up |
| PLAGL1 | 1.054730724 | 5.5896479 | 4.17E-22 | 3.57E-21 | Up |
| C10orf72 | 1.054653499 | 5.791335798 | 6.59E-23 | 5.92E-22 | Up |
| SLC16A6 | 1.054180124 | 3.518043492 | 3.49E-18 | 2.26E-17 | Up |
| KIAA0087 | 1.053760939 | 0.367051687 | 2.54E-18 | 1.66E-17 | Up |
| KLHL4 | 1.053597116 | 2.873538749 | 4.79E-13 | 2.12E-12 | Up |
| SLC15A3 | 1.053594206 | 6.795627674 | 3.13E-26 | 3.58E-25 | Up |
| PEG10 | 1.052456109 | 6.721452772 | 3.01E-06 | 7.38E-06 | Up |
| PPP1R14C | 1.051481659 | 5.396850598 | 6.91E-09 | 2.14E-08 | Up |
| CELF2 | 1.048992251 | 7.156890471 | 5.60E-24 | 5.41E-23 | Up |
| TTN | 1.048581304 | 4.946747368 | 5.30E-24 | 5.13E-23 | Up |
| LOC644165 | 1.048469553 | 2.841744337 | 1.51E-11 | 5.89E-11 | Up |
| GJA4 | 1.048084579 | 5.109112769 | 2.60E-28 | 3.41E-27 | Up |
| PNPLA6 | 1.047857257 | 7.282787877 | 3.45E-60 | 3.89E-58 | Up |

| | | | | | |
|---|---|---|---|---|---|
| DST | 1.047420573 | 7.62202514 | 3.17E-19 | 2.21E-18 | Up |
| PCDHA3 | 1.046145853 | 3.058254966 | 1.81E-12 | 7.62E-12 | Up |
| MYH15 | 1.045615792 | 2.136650971 | 1.39E-09 | 4.57E-09 | Up |
| HBD | 1.04498877 | 0.284928845 | 1.48E-17 | 9.14E-17 | Up |
| CAPN11 | 1.044490382 | 0.872158261 | 6.49E-17 | 3.80E-16 | Up |
| ITGAL | 1.043691748 | 6.554623488 | 1.11E-19 | 7.97E-19 | Up |
| PLA2G2A | 1.042651053 | 3.332938515 | 2.43E-05 | 5.43E-05 | Up |
| KIR2DS4 | 1.042424505 | 0.571371833 | 3.48E-11 | 1.31E-10 | Up |
| NFAM1 | 1.041363166 | 5.700256395 | 5.27E-20 | 3.88E-19 | Up |
| CFL2 | 1.041180266 | 6.327350082 | 3.27E-39 | 8.84E-38 | Up |
| ZC3H12B | 1.041033679 | 3.150523102 | 1.32E-25 | 1.43E-24 | Up |
| FERMT2 | 1.040884094 | 6.850004968 | 4.16E-47 | 1.86E-45 | Up |
| FAM167B | 1.040481181 | 4.382059341 | 1.09E-34 | 2.20E-33 | Up |
| GREM2 | 1.040327194 | 3.375133156 | 2.98E-06 | 7.32E-06 | Up |
| MYLK | 1.040281593 | 7.50847052 | 4.05E-24 | 3.95E-23 | Up |
| SHISA2 | 1.039372204 | 4.786068665 | 8.24E-07 | 2.12E-06 | Up |
| EPB41L3 | 1.039222467 | 6.153891686 | 4.96E-21 | 3.92E-20 | Up |
| HLA-DOA | 1.036768852 | 7.049051855 | 7.29E-15 | 3.69E-14 | Up |
| SLC34A2 | 1.036656149 | 10.11097705 | 1.09E-07 | 3.03E-07 | Up |
| SCN3B | 1.036645293 | 2.57382454 | 5.59E-13 | 2.46E-12 | Up |
| PDCD1LG2 | 1.036523627 | 4.563650832 | 4.95E-16 | 2.74E-15 | Up |
| NR3C2 | 1.036446841 | 5.587432153 | 3.02E-14 | 1.47E-13 | Up |
| EMP1 | 1.036246601 | 7.815043007 | 1.39E-23 | 1.30E-22 | Up |
| OASL | 1.035838096 | 5.363677494 | 8.62E-15 | 4.35E-14 | Up |
| NTS | 1.035807425 | 1.980266376 | 0.001058677 | 0.001990265 | Up |
| LOC134466 | 1.035236233 | 2.407896754 | 2.23E-11 | 8.58E-11 | Up |
| PDGFB | 1.034904897 | 6.114728174 | 1.53E-29 | 2.18E-28 | Up |
| RERG | 1.034641842 | 5.077932497 | 5.04E-17 | 2.98E-16 | Up |
| ZNF474 | 1.033364465 | 2.340087556 | 4.03E-10 | 1.39E-09 | Up |
| GVIN1 | 1.032972391 | 4.745885128 | 3.58E-16 | 2.00E-15 | Up |
| DSCAML1 | 1.032816354 | 2.498035158 | 6.23E-07 | 1.62E-06 | Up |
| PLA2G5 | 1.032204527 | 3.486067469 | 5.22E-18 | 3.33E-17 | Up |
| RNF180 | 1.031775255 | 4.490220445 | 1.11E-17 | 6.92E-17 | Up |
| GPR4 | 1.03119068 | 4.947812088 | 3.90E-32 | 6.65E-31 | Up |
| CCDC54 | 1.030160654 | -0.058930004 | 1.63E-40 | 4.80E-39 | Up |
| FLT4 | 1.02920207 | 5.894017787 | 4.65E-26 | 5.25E-25 | Up |
| STON1 | 1.028966819 | 5.344314975 | 4.60E-27 | 5.55E-26 | Up |
| TCEAL7 | 1.028634881 | 3.10981471 | 9.38E-21 | 7.29E-20 | Up |
| KLHL33 | 1.028120787 | 0.19225381 | 1.21E-24 | 1.23E-23 | Up |
| GLIPR1L2 | 1.027959455 | 2.061402868 | 1.14E-14 | 5.70E-14 | Up |
| CORIN | 1.027884636 | 3.680504537 | 7.98E-12 | 3.19E-11 | Up |
| SH3BP5 | 1.027210645 | 7.044508331 | 9.40E-31 | 1.45E-29 | Up |
| CLUL1 | 1.027098178 | 2.86672634 | 5.85E-08 | 1.67E-07 | Up |
| C1orf21 | 1.026642197 | 6.534085146 | 9.03E-22 | 7.56E-21 | Up |
| FBXO15 | 1.025289044 | 2.761359392 | 4.59E-10 | 1.58E-09 | Up |
| DNM3 | 1.025234237 | 4.437708803 | 3.47E-16 | 1.94E-15 | Up |

| | | | | | |
|---|---|---|---|---|---|
| PTGER3 | 1.024972813 | 3.243300092 | 3.96E-09 | 1.25E-08 | Up |
| SLC6A12 | 1.024503129 | 3.752681934 | 7.61E-14 | 3.58E-13 | Up |
| N4BP2L1 | 1.022804605 | 5.459482453 | 5.80E-30 | 8.49E-29 | Up |
| NLGN1 | 1.021527298 | 1.594019469 | 4.57E-08 | 1.32E-07 | Up |
| MOCS1 | 1.021518287 | 5.697818759 | 2.30E-28 | 3.04E-27 | Up |
| IL1B | 1.021010399 | 4.758061828 | 4.55E-13 | 2.01E-12 | Up |
| LRRN4CL | 1.020932819 | 2.935148125 | 1.48E-13 | 6.82E-13 | Up |
| PPARGC1B | 1.020909155 | 2.86319667 | 3.51E-18 | 2.27E-17 | Up |
| SPEF1 | 1.019170182 | 2.820563011 | 3.73E-07 | 9.90E-07 | Up |
| SLC2A3 | 1.0191565 | 7.032915965 | 6.82E-18 | 4.30E-17 | Up |
| CX3CL1 | 1.019102419 | 7.25659568 | 4.05E-11 | 1.52E-10 | Up |
| IQUB | 1.017573263 | 2.33873066 | 1.47E-10 | 5.24E-10 | Up |
| LOC441204 | 1.017343376 | 2.944603476 | 4.24E-16 | 2.36E-15 | Up |
| EFHA2 | 1.017001505 | 3.854122147 | 2.90E-21 | 2.34E-20 | Up |
| C2orf77 | 1.016969661 | 4.783727109 | 9.50E-16 | 5.14E-15 | Up |
| MS4A4A | 1.01648234 | 5.927855946 | 6.44E-18 | 4.07E-17 | Up |
| SLC27A3 | 1.016300444 | 6.404485273 | 5.24E-34 | 1.01E-32 | Up |
| FAM153A | 1.0156903 | 1.549006256 | 1.13E-06 | 2.88E-06 | Up |
| NCF1 | 1.015173818 | 5.190005292 | 9.19E-16 | 4.98E-15 | Up |
| TMIE | 1.015066294 | 2.433166078 | 1.94E-15 | 1.03E-14 | Up |
| SHH | 1.014827651 | 2.514096108 | 1.38E-07 | 3.82E-07 | Up |
| RORA | 1.014824918 | 5.857208081 | 4.21E-27 | 5.11E-26 | Up |
| ICAM2 | 1.014387084 | 6.171470791 | 1.15E-32 | 2.03E-31 | Up |
| 10-Mar | 1.014160983 | 2.425523725 | 1.52E-07 | 4.19E-07 | Up |
| ABCC8 | 1.013114961 | 1.19645491 | 2.30E-06 | 5.70E-06 | Up |
| MITF | 1.013101636 | 5.619099681 | 6.30E-29 | 8.65E-28 | Up |
| ZMYND15 | 1.012853335 | 4.562155833 | 6.66E-22 | 5.61E-21 | Up |
| BCO2 | 1.011922477 | 2.772312603 | 1.32E-12 | 5.63E-12 | Up |
| SLC47A1 | 1.011888726 | 5.16766123 | 3.40E-10 | 1.18E-09 | Up |
| BNIPL | 1.011847841 | 4.758502101 | 5.60E-07 | 1.46E-06 | Up |
| BTG2 | 1.011743672 | 7.92059933 | 3.13E-19 | 2.18E-18 | Up |
| PLBD1 | 1.011700301 | 6.612605982 | 4.24E-17 | 2.52E-16 | Up |
| CRB1 | 1.010516252 | 0.857463759 | 2.55E-11 | 9.72E-11 | Up |
| TXNDC6 | 1.009197179 | 2.193933785 | 2.90E-10 | 1.01E-09 | Up |
| LOC344595 | 1.008515349 | 4.173593966 | 1.66E-29 | 2.36E-28 | Up |
| PLA2G12B | 1.00834594 | 2.569483929 | 0.000149004 | 0.000307458 | Up |
| GPR44 | 1.006034412 | 2.034184128 | 1.75E-14 | 8.63E-14 | Up |
| KLF13 | 1.005037794 | 7.600780362 | 8.22E-40 | 2.31E-38 | Up |
| MATK | 1.005014394 | 4.269480378 | 5.20E-16 | 2.87E-15 | Up |
| SULT1E1 | 1.004328797 | 1.40435768 | 1.92E-07 | 5.22E-07 | Up |
| DNAJC5B | 1.004301743 | 3.437359794 | 1.45E-11 | 5.67E-11 | Up |
| EXOC3L | 1.004273495 | 4.426634628 | 2.73E-26 | 3.14E-25 | Up |
| MMP19 | 1.004079201 | 6.157745019 | 3.64E-31 | 5.85E-30 | Up |
| FLVCR2 | 1.003902385 | 5.866382311 | 5.89E-27 | 7.04E-26 | Up |
| C3orf54 | 1.003828295 | 3.440657587 | 7.85E-26 | 8.74E-25 | Up |
| FRMD4A | 1.003345311 | 5.907218704 | 2.56E-41 | 8.05E-40 | Up |

| | | | | | |
|---|---|---|---|---|---|
| SNX25 | 1.002984437 | 6.452143727 | 3.64E-28 | 4.71E-27 | Up |
| CDK15 | 1.002851018 | 0.606116695 | 5.52E-15 | 2.82E-14 | Up |
| HELT | 1.002649815 | 0.090083981 | 1.08E-31 | 1.80E-30 | Up |
| PARD3B | 1.002413335 | 3.909376609 | 7.31E-14 | 3.45E-13 | Up |
| BCL2A1 | 1.002360509 | 5.246513795 | 2.81E-17 | 1.69E-16 | Up |
| CYP11A1 | 1.002076824 | 2.263296662 | 1.91E-07 | 5.19E-07 | Up |
| OLFM1 | 1.001232914 | 4.979726939 | 6.71E-10 | 2.27E-09 | Up |
| GPR65 | 1.000938535 | 4.573256681 | 2.10E-20 | 1.60E-19 | Up |
| DDR2 | 1.000705051 | 4.626544646 | 2.19E-14 | 1.07E-13 | Up |
| WNT2 | 1.000395392 | 4.970349228 | 3.46E-13 | 1.55E-12 | Up |
| PPP1R16B | 1.000270825 | 5.698785983 | 9.47E-18 | 5.91E-17 | Up |
| ZFYVE9 | 1.000267067 | 5.978318859 | 3.56E-36 | 7.96E-35 | Up |
| UCK2 | -1.000515621 | 5.335157249 | 3.16E-15 | 1.65E-14 | Down |
| TRIM59 | -1.000730529 | 5.254534222 | 1.15E-31 | 1.90E-30 | Down |
| PRAP1 | -1.001233332 | 0.90877101 | 0.000131917 | 0.000273768 | Down |
| CBX8 | -1.001238673 | 5.18944732 | 7.12E-44 | 2.60E-42 | Down |
| APBA2 | -1.001560017 | 5.299599913 | 1.05E-10 | 3.80E-10 | Down |
| LOC100131551 | -1.001823456 | 0.82117582 | 9.52E-09 | 2.91E-08 | Down |
| KIAA0125 | -1.001825561 | 3.871429179 | 1.43E-07 | 3.94E-07 | Down |
| CSAG1 | -1.002327972 | 0.905329489 | 0.000473318 | 0.000927172 | Down |
| LOC389791 | -1.002534447 | 1.064698675 | 4.49E-13 | 1.99E-12 | Down |
| SGPP2 | -1.005227913 | 5.860898375 | 1.27E-14 | 6.35E-14 | Down |
| RPSAP52 | -1.0053814 | 0.773028772 | 6.98E-08 | 1.98E-07 | Down |
| HGFAC | -1.006087468 | 0.895170232 | 1.53E-08 | 4.59E-08 | Down |
| FNDC4 | -1.007691747 | 4.986691204 | 8.18E-13 | 3.55E-12 | Down |
| RNF207 | -1.008057502 | 5.065249689 | 5.83E-17 | 3.42E-16 | Down |
| MSTO2P | -1.008283411 | 4.68250547 | 3.38E-25 | 3.56E-24 | Down |
| MIF | -1.00848028 | 8.479591832 | 9.61E-29 | 1.30E-27 | Down |
| ELOVL2 | -1.009661911 | 2.944086633 | 5.31E-11 | 1.97E-10 | Down |
| KIAA1199 | -1.009948216 | 6.45186907 | 3.70E-09 | 1.17E-08 | Down |
| CSMD2 | -1.010175745 | 3.048568959 | 8.94E-11 | 3.25E-10 | Down |
| SBSN | -1.012838022 | 1.15728621 | 1.17E-05 | 2.72E-05 | Down |
| HSF4 | -1.012948071 | 4.853709375 | 2.80E-11 | 1.06E-10 | Down |
| HIST2H3C | -1.013231864 | 1.103784245 | 1.22E-11 | 4.80E-11 | Down |
| TSPAN10 | -1.013623131 | 3.134188631 | 2.36E-12 | 9.85E-12 | Down |
| SPP2 | -1.014650355 | 0.88294746 | 1.71E-06 | 4.29E-06 | Down |
| ZIC5 | -1.014664972 | 0.801265796 | 1.35E-06 | 3.40E-06 | Down |
| ALS2CR11 | -1.015716944 | 2.622393985 | 1.11E-07 | 3.09E-07 | Down |
| PSMG3 | -1.016095043 | 6.530154034 | 5.48E-40 | 1.55E-38 | Down |
| SCN4A | -1.016984589 | 2.008694398 | 4.81E-06 | 1.15E-05 | Down |
| TSHR | -1.017601841 | 1.407574669 | 2.54E-10 | 8.90E-10 | Down |
| TMEM182 | -1.017764744 | 4.901722452 | 9.58E-46 | 3.94E-44 | Down |
| SNHG3 | -1.018143761 | 4.143024943 | 1.67E-23 | 1.56E-22 | Down |
| TFPI2 | -1.018367476 | 5.846267985 | 0.000344041 | 0.000683786 | Down |
| PKP3 | -1.018552404 | 7.223641336 | 1.29E-35 | 2.80E-34 | Down |
| TMEM132E | -1.01905033 | 2.080148102 | 2.17E-09 | 7.03E-09 | Down |

| | | | | | |
|---|---|---|---|---|---|
| SLC6A3 | -1.01914832 | 2.77385575 | 0.00084321 | 0.001604443 | Down |
| WISP3 | -1.019408154 | 0.8718514 | 4.35E-06 | 1.05E-05 | Down |
| LOC440356 | -1.021487838 | 0.883623639 | 9.96E-08 | 2.78E-07 | Down |
| MS4A1 | -1.022127547 | 4.485104988 | 1.18E-05 | 2.72E-05 | Down |
| LOC642846 | -1.022165236 | 4.752727804 | 4.74E-22 | 4.04E-21 | Down |
| SERPINB4 | -1.023691328 | 1.535155925 | 0.000357691 | 0.00070961 | Down |
| NLN | -1.024693865 | 5.921883949 | 7.95E-44 | 2.89E-42 | Down |
| HABP2 | -1.025682543 | 4.915579006 | 0.000215764 | 0.000437301 | Down |
| IER5L | -1.02587931 | 5.659157903 | 4.43E-19 | 3.06E-18 | Down |
| POU6F2 | -1.02789822 | 1.133983039 | 7.03E-06 | 1.66E-05 | Down |
| SLC4A11 | -1.028046502 | 4.60353782 | 2.61E-08 | 7.69E-08 | Down |
| C6orf129 | -1.028609334 | 5.614945116 | 1.70E-37 | 4.09E-36 | Down |
| SPRR3 | -1.030061213 | 1.143029445 | 8.18E-05 | 0.00017331 | Down |
| SAA4 | -1.031065781 | 1.488147302 | 3.10E-06 | 7.59E-06 | Down |
| FEN1 | -1.03158461 | 6.402521845 | 2.08E-39 | 5.70E-38 | Down |
| MANEAL | -1.031614967 | 5.837075407 | 6.04E-18 | 3.83E-17 | Down |
| UBE2S | -1.032590354 | 5.689524115 | 9.60E-22 | 8.01E-21 | Down |
| SLC23A3 | -1.033751875 | 2.412345324 | 6.37E-12 | 2.57E-11 | Down |
| SLC4A3 | -1.033912455 | 4.33672065 | 6.09E-11 | 2.25E-10 | Down |
| PI15 | -1.035187277 | 4.122782171 | 2.80E-09 | 8.99E-09 | Down |
| NR0B1 | -1.035490231 | 1.090549297 | 0.000806517 | 0.001538046 | Down |
| AGXT2L1 | -1.035873875 | 0.865049706 | 2.44E-05 | 5.45E-05 | Down |
| C19orf45 | -1.036326399 | 0.972255793 | 7.64E-10 | 2.57E-09 | Down |
| C8orf51 | -1.03694693 | 3.64566394 | 1.67E-23 | 1.56E-22 | Down |
| C1orf126 | -1.037945679 | 4.119457207 | 1.98E-18 | 1.30E-17 | Down |
| LOC100190940 | -1.038101916 | 0.885549126 | 0.000131826 | 0.000273607 | Down |
| ZC3HAV1L | -1.038496601 | 3.819906392 | 1.40E-25 | 1.52E-24 | Down |
| LOC145837 | -1.038545691 | 3.119019571 | 0.0003933 | 0.0007771 | Down |
| GLTPD2 | -1.039060318 | 1.131272992 | 1.69E-05 | 3.84E-05 | Down |
| TRIM67 | -1.039385356 | 1.24159375 | 1.93E-13 | 8.81E-13 | Down |
| RPL36A | -1.040665119 | 4.176394379 | 9.74E-30 | 1.41E-28 | Down |
| SEMA4B | -1.041037773 | 8.103641975 | 3.80E-31 | 6.10E-30 | Down |
| ZNF239 | -1.042498595 | 4.579792369 | 3.79E-30 | 5.63E-29 | Down |
| INSL4 | -1.043674305 | 0.925315323 | 0.000323092 | 0.00064377 | Down |
| MSI2 | -1.044183432 | 5.461903631 | 3.28E-43 | 1.14E-41 | Down |
| ODZ4 | -1.045078361 | 5.350468261 | 3.32E-16 | 1.86E-15 | Down |
| PAX5 | -1.04587631 | 2.357431688 | 2.80E-07 | 7.51E-07 | Down |
| KLK8 | -1.047948137 | 1.344085723 | 0.00078778 | 0.001504129 | Down |
| CDX2 | -1.048441325 | 0.937743448 | 9.04E-06 | 2.11E-05 | Down |
| ACAN | -1.049523647 | 3.571045807 | 2.51E-09 | 8.08E-09 | Down |
| FAM132A | -1.049791925 | 1.450226477 | 3.19E-08 | 9.32E-08 | Down |
| SLC28A2 | -1.050200838 | 1.109730046 | 0.000185006 | 0.000377759 | Down |
| TRY6 | -1.050578863 | 1.102070744 | 2.63E-05 | 5.85E-05 | Down |
| NUP62CL | -1.050930623 | 3.711509447 | 2.56E-18 | 1.67E-17 | Down |
| DUSP4 | -1.052021566 | 6.017881275 | 8.17E-11 | 2.98E-10 | Down |
| PDK1 | -1.054484666 | 6.173533237 | 7.48E-35 | 1.52E-33 | Down |

| | | | | | |
|---|---|---|---|---|---|
| BAI2 | -1.055522756 | 4.284201303 | 8.48E-13 | 3.68E-12 | Down |
| MT1H | -1.055813574 | 1.566279234 | 1.35E-07 | 3.72E-07 | Down |
| SRPK1 | -1.056769939 | 7.373495523 | 2.17E-68 | 4.80E-66 | Down |
| SYNJ2 | -1.057546677 | 5.91846519 | 2.16E-36 | 4.88E-35 | Down |
| MSTO1 | -1.057595687 | 6.606882498 | 3.23E-62 | 4.28E-60 | Down |
| AHNAK2 | -1.058486732 | 7.214981926 | 2.95E-09 | 9.46E-09 | Down |
| EPCAM | -1.060823575 | 9.015236061 | 3.73E-41 | 1.15E-39 | Down |
| DQX1 | -1.060973799 | 1.446386547 | 3.77E-07 | 1.00E-06 | Down |
| MPP6 | -1.062398855 | 4.358934701 | 5.35E-20 | 3.94E-19 | Down |
| MGC12982 | -1.064204059 | 4.286396718 | 1.42E-35 | 3.05E-34 | Down |
| FBXO16 | -1.064750447 | 3.707953819 | 7.35E-22 | 6.18E-21 | Down |
| KCND2 | -1.064810296 | 3.321842631 | 1.10E-09 | 3.65E-09 | Down |
| CAPN12 | -1.066076526 | 5.811460684 | 6.96E-18 | 4.39E-17 | Down |
| TAS1R3 | -1.068321816 | 3.257174956 | 2.56E-11 | 9.78E-11 | Down |
| SLC9A7 | -1.068537384 | 4.164500962 | 1.56E-22 | 1.36E-21 | Down |
| HIST1H2BH | -1.068537652 | 0.999218425 | 3.06E-09 | 9.79E-09 | Down |
| PLD5 | -1.068908128 | 2.752698235 | 3.74E-05 | 8.21E-05 | Down |
| GALNT7 | -1.069761123 | 7.229943121 | 1.89E-39 | 5.21E-38 | Down |
| HIST2H4A | -1.069824553 | 5.772313723 | 2.26E-21 | 1.83E-20 | Down |
| CDX1 | -1.069939719 | 1.481228843 | 1.92E-15 | 1.02E-14 | Down |
| TMEM45B | -1.070266965 | 6.653324409 | 1.05E-16 | 6.08E-16 | Down |
| TTBK1 | -1.071661535 | 1.909106816 | 7.45E-18 | 4.68E-17 | Down |
| PFN4 | -1.07253338 | 1.89235198 | 1.14E-15 | 6.15E-15 | Down |
| GCM1 | -1.073327597 | 1.052199559 | 5.85E-10 | 1.99E-09 | Down |
| NSUN5P1 | -1.073848776 | 4.785809412 | 8.75E-19 | 5.90E-18 | Down |
| HIST1H2BN | -1.076334173 | 2.188795392 | 7.05E-18 | 4.44E-17 | Down |
| GGCT | -1.076401983 | 7.06984949 | 1.59E-46 | 6.86E-45 | Down |
| TACC3 | -1.076662051 | 6.636298656 | 2.26E-33 | 4.17E-32 | Down |
| DDX11 | -1.076700737 | 6.051728336 | 4.33E-27 | 5.23E-26 | Down |
| POC1A | -1.077124488 | 5.021869268 | 1.82E-35 | 3.88E-34 | Down |
| CKS1B | -1.078193591 | 6.517200534 | 2.82E-31 | 4.57E-30 | Down |
| PNOC | -1.078378699 | 2.861688408 | 9.47E-10 | 3.16E-09 | Down |
| GCKR | -1.078694106 | 0.883536111 | 3.96E-08 | 1.15E-07 | Down |
| T | -1.078851147 | 0.936114837 | 1.01E-06 | 2.59E-06 | Down |
| RASGEF1A | -1.079177234 | 4.919216152 | 4.95E-13 | 2.19E-12 | Down |
| RAB19 | -1.079259385 | 2.709069432 | 1.62E-21 | 1.33E-20 | Down |
| SYNGR4 | -1.079874827 | 0.886652033 | 1.48E-10 | 5.27E-10 | Down |
| KCNQ5 | -1.080255044 | 3.601559988 | 5.53E-08 | 1.58E-07 | Down |
| SFXN1 | -1.080591649 | 6.599815698 | 1.71E-63 | 2.60E-61 | Down |
| TIMP1 | -1.081361856 | 8.990475865 | 4.84E-31 | 7.67E-30 | Down |
| KIFC2 | -1.08151681 | 6.011844486 | 6.48E-20 | 4.74E-19 | Down |
| ANKRD23 | -1.082274378 | 3.655185846 | 7.06E-26 | 7.87E-25 | Down |
| SYCE2 | -1.082666185 | 1.668661884 | 9.98E-16 | 5.40E-15 | Down |
| SYT13 | -1.08377055 | 3.160908098 | 0.001774647 | 0.003243304 | Down |
| HN1 | -1.083902013 | 7.826661623 | 8.67E-40 | 2.43E-38 | Down |
| FGF19 | -1.08412082 | 0.96746569 | 1.06E-05 | 2.47E-05 | Down |

| | | | | | |
|---|---|---|---|---|---|
| RLTPR | -1.086131387 | 4.236643891 | 3.37E-12 | 1.39E-11 | Down |
| KRT83 | -1.086172196 | 0.917123689 | 2.14E-07 | 5.80E-07 | Down |
| STK32A | -1.088042264 | 4.021877609 | 1.23E-08 | 3.72E-08 | Down |
| CLCN2 | -1.089907777 | 4.62689719 | 2.87E-38 | 7.29E-37 | Down |
| COL9A3 | -1.090368131 | 2.739864243 | 5.81E-10 | 1.98E-09 | Down |
| ZNF643 | -1.090666966 | 3.564063155 | 2.69E-39 | 7.33E-38 | Down |
| GUCA1A | -1.090694311 | 0.892120491 | 2.84E-11 | 1.08E-10 | Down |
| SLFN13 | -1.090895929 | 6.564659174 | 4.88E-25 | 5.08E-24 | Down |
| UGT2B15 | -1.091725282 | 1.633793414 | 5.03E-05 | 0.000109204 | Down |
| C9orf173 | -1.092273639 | 1.133730795 | 2.88E-09 | 9.22E-09 | Down |
| FBXO41 | -1.092762709 | 5.386126307 | 5.20E-24 | 5.03E-23 | Down |
| FAM171A2 | -1.092813181 | 3.909982502 | 9.31E-12 | 3.70E-11 | Down |
| ENO3 | -1.093170884 | 4.076200992 | 1.99E-08 | 5.93E-08 | Down |
| ENTPD2 | -1.093226317 | 3.524565036 | 3.37E-09 | 1.07E-08 | Down |
| HIST1H4H | -1.094219675 | 4.29984002 | 1.98E-16 | 1.13E-15 | Down |
| FCRL4 | -1.094781197 | 1.264842477 | 6.43E-10 | 2.18E-09 | Down |
| KIF4B | -1.096558312 | 1.717391129 | 1.03E-18 | 6.91E-18 | Down |
| PRRX2 | -1.096655719 | 3.86171957 | 5.99E-09 | 1.87E-08 | Down |
| CPT1B | -1.097732253 | 5.169042293 | 7.15E-16 | 3.90E-15 | Down |
| SCUBE3 | -1.098647554 | 4.178946965 | 5.81E-08 | 1.66E-07 | Down |
| PKD1L2 | -1.099088133 | 2.53819196 | 1.82E-06 | 4.55E-06 | Down |
| DSCC1 | -1.099972722 | 4.638472447 | 2.50E-23 | 2.30E-22 | Down |
| HCN3 | -1.10135059 | 4.514118767 | 5.55E-29 | 7.65E-28 | Down |
| SFRS13B | -1.101633622 | 3.103511795 | 2.07E-13 | 9.43E-13 | Down |
| TIMELESS | -1.102488815 | 6.61055619 | 1.19E-41 | 3.82E-40 | Down |
| HIST1H1D | -1.10302738 | 0.903772212 | 1.32E-11 | 5.17E-11 | Down |
| CFB | -1.103147503 | 8.099579787 | 5.72E-14 | 2.72E-13 | Down |
| POSTN | -1.103283851 | 8.082206222 | 8.27E-16 | 4.50E-15 | Down |
| HOXD3 | -1.104248616 | 1.942513357 | 1.09E-07 | 3.03E-07 | Down |
| ALDH18A1 | -1.10672834 | 7.59082498 | 2.50E-76 | 1.00E-73 | Down |
| GAPDH | -1.10752611 | 10.68768505 | 8.19E-41 | 2.46E-39 | Down |
| NCAPG2 | -1.108249561 | 6.129494626 | 2.46E-33 | 4.53E-32 | Down |
| ZNF692 | -1.108574064 | 6.082383991 | 9.25E-31 | 1.43E-29 | Down |
| IGFBPL1 | -1.108963245 | 2.601602788 | 5.32E-08 | 1.53E-07 | Down |
| CLDN3 | -1.110799997 | 7.110235783 | 1.13E-13 | 5.28E-13 | Down |
| HOXD4 | -1.111856014 | 1.684141953 | 4.35E-07 | 1.15E-06 | Down |
| GJA9 | -1.112067502 | 2.120902674 | 3.93E-29 | 5.46E-28 | Down |
| C20orf197 | -1.115037212 | 2.545890673 | 6.78E-08 | 1.92E-07 | Down |
| XPR1 | -1.116775093 | 7.85806375 | 1.10E-33 | 2.07E-32 | Down |
| MSH5 | -1.117715694 | 5.410532011 | 1.04E-28 | 1.41E-27 | Down |
| HIST1H3H | -1.118028047 | 2.27562942 | 1.47E-12 | 6.24E-12 | Down |
| THY1 | -1.118647613 | 7.410678647 | 2.26E-22 | 1.97E-21 | Down |
| CNGB1 | -1.11934694 | 2.542034569 | 9.59E-08 | 2.68E-07 | Down |
| FAM150A | -1.120405006 | 2.265102083 | 7.01E-07 | 1.82E-06 | Down |
| KCNG2 | -1.121414156 | 1.482812733 | 3.60E-12 | 1.48E-11 | Down |
| B4GALNT1 | -1.121622618 | 3.423367885 | 2.23E-09 | 7.22E-09 | Down |

| | | | | | |
|---|---|---|---|---|---|
| ATAD2 | -1.122163414 | 6.612502943 | 6.17E-32 | 1.04E-30 | Down |
| RFC4 | -1.123563674 | 5.604879977 | 3.42E-35 | 7.17E-34 | Down |
| GPR84 | -1.123963911 | 3.683906621 | 1.42E-15 | 7.62E-15 | Down |
| SMPDL3B | -1.124219236 | 6.837139285 | 2.18E-21 | 1.77E-20 | Down |
| GRIN2D | -1.124606081 | 4.009866742 | 3.78E-10 | 1.31E-09 | Down |
| SOX4 | -1.12607891 | 8.243959871 | 5.00E-42 | 1.63E-40 | Down |
| HIST2H2AA3 | -1.126165215 | 6.18655977 | 4.03E-18 | 2.59E-17 | Down |
| TPBG | -1.126878752 | 6.418359935 | 2.23E-31 | 3.65E-30 | Down |
| RHPN1 | -1.127190795 | 6.01677752 | 1.25E-21 | 1.03E-20 | Down |
| ADCK5 | -1.127452356 | 5.235100325 | 2.65E-31 | 4.31E-30 | Down |
| QPCT | -1.129116289 | 5.938637337 | 1.15E-09 | 3.81E-09 | Down |
| ATP2A1 | -1.12916501 | 2.009015898 | 7.09E-15 | 3.59E-14 | Down |
| STRC | -1.130862484 | 1.704688292 | 3.54E-11 | 1.33E-10 | Down |
| MYO19 | -1.131996548 | 6.657134946 | 5.56E-58 | 5.17E-56 | Down |
| DONSON | -1.132791815 | 5.431133146 | 1.86E-51 | 1.11E-49 | Down |
| ABCB6 | -1.133477194 | 6.378205278 | 1.28E-28 | 1.72E-27 | Down |
| ATG9B | -1.133576346 | 3.883884858 | 1.40E-12 | 5.95E-12 | Down |
| TMEM52 | -1.134007082 | 3.42654618 | 3.18E-17 | 1.91E-16 | Down |
| SPTB | -1.134607743 | 5.621994342 | 7.33E-08 | 2.07E-07 | Down |
| FANCB | -1.134615962 | 2.696104596 | 3.59E-24 | 3.52E-23 | Down |
| CARD11 | -1.134792812 | 6.029656507 | 4.41E-16 | 2.45E-15 | Down |
| FLJ12825 | -1.13571049 | 1.467983763 | 2.34E-13 | 1.06E-12 | Down |
| ARHGEF16 | -1.135806932 | 6.374205239 | 2.83E-38 | 7.19E-37 | Down |
| TMEM177 | -1.136740669 | 5.290272774 | 8.60E-74 | 2.85E-71 | Down |
| RPP40 | -1.136790123 | 4.556557947 | 1.71E-41 | 5.40E-40 | Down |
| RALGPS2 | -1.139653327 | 6.025706382 | 2.99E-35 | 6.27E-34 | Down |
| KAT2A | -1.139803825 | 7.024290622 | 3.28E-52 | 2.02E-50 | Down |
| IL1F9 | -1.142686083 | 0.989811006 | 2.41E-09 | 7.77E-09 | Down |
| WFDC10B | -1.143941437 | 1.83554998 | 5.94E-09 | 1.85E-08 | Down |
| GNB3 | -1.144361257 | 2.622584916 | 9.11E-17 | 5.27E-16 | Down |
| SLC16A14 | -1.144894464 | 5.55146733 | 9.16E-10 | 3.06E-09 | Down |
| PLEKHG6 | -1.14688937 | 5.158188359 | 7.71E-20 | 5.61E-19 | Down |
| ADAMTS18 | -1.147055246 | 1.756440566 | 2.97E-10 | 1.03E-09 | Down |
| FSD1 | -1.147514153 | 1.42113471 | 2.58E-10 | 9.03E-10 | Down |
| PHKA1 | -1.147657644 | 6.0254744 | 7.44E-47 | 3.29E-45 | Down |
| HOXC6 | -1.149323309 | 2.994686101 | 1.66E-07 | 4.55E-07 | Down |
| SLC6A17 | -1.149753356 | 1.763834092 | 3.22E-08 | 9.41E-08 | Down |
| KIF5A | -1.149828468 | 1.361037601 | 8.83E-09 | 2.71E-08 | Down |
| C22orf41 | -1.150099254 | 1.491037803 | 9.40E-13 | 4.06E-12 | Down |
| SPINK13 | -1.15202093 | 1.03037831 | 2.14E-07 | 5.80E-07 | Down |
| PC | -1.152330879 | 6.401709514 | 7.81E-36 | 1.71E-34 | Down |
| GLDC | -1.152876344 | 3.150753659 | 1.29E-07 | 3.58E-07 | Down |
| PGM2L1 | -1.153548505 | 6.507231989 | 1.49E-35 | 3.20E-34 | Down |
| SLAMF9 | -1.154547249 | 2.210000875 | 8.19E-11 | 2.99E-10 | Down |
| REEP6 | -1.155462069 | 5.484780774 | 6.82E-13 | 2.98E-12 | Down |
| PPEF1 | -1.156541156 | 2.12466814 | 3.24E-12 | 1.34E-11 | Down |

| | | | | | |
|---|---|---|---|---|---|
| FANCD2 | -1.157601275 | 5.463562429 | 1.19E-45 | 4.87E-44 | Down |
| WDHD1 | -1.158027921 | 5.330637746 | 2.34E-31 | 3.81E-30 | Down |
| PDIA4 | -1.158155032 | 9.004354108 | 3.03E-56 | 2.53E-54 | Down |
| ALOX12P2 | -1.159548562 | 2.529729902 | 1.13E-06 | 2.88E-06 | Down |
| THPO | -1.160669956 | 2.42740589 | 3.39E-07 | 9.04E-07 | Down |
| NPM3 | -1.162748677 | 5.923752773 | 8.74E-48 | 4.00E-46 | Down |
| TLCD1 | -1.163537566 | 5.840151715 | 3.08E-37 | 7.26E-36 | Down |
| RAG1AP1 | -1.164284034 | 7.454415992 | 9.15E-44 | 3.31E-42 | Down |
| FAM131C | -1.166957486 | 0.968781828 | 6.41E-11 | 2.36E-10 | Down |
| ASAH2B | -1.170455547 | 1.727218182 | 3.57E-15 | 1.85E-14 | Down |
| SNHG4 | -1.170767653 | 2.013723752 | 2.38E-20 | 1.80E-19 | Down |
| CYP2D7P1 | -1.171099295 | 2.31108823 | 1.15E-13 | 5.33E-13 | Down |
| SAMD10 | -1.172035595 | 5.296325338 | 2.25E-46 | 9.64E-45 | Down |
| HIST1H4J | -1.172609629 | 2.859610648 | 4.62E-22 | 3.94E-21 | Down |
| GJB3 | -1.172912608 | 4.1437938 | 3.38E-06 | 8.24E-06 | Down |
| HTR1D | -1.174548078 | 2.934341128 | 2.39E-07 | 6.45E-07 | Down |
| SULT4A1 | -1.175983606 | 1.415692932 | 2.85E-06 | 7.00E-06 | Down |
| MAGEA12 | -1.176099109 | 1.076649859 | 0.000294182 | 0.000588185 | Down |
| MUC4 | -1.176358631 | 7.017339909 | 2.05E-06 | 5.10E-06 | Down |
| DLX6 | -1.177379257 | 1.100015954 | 6.76E-07 | 1.75E-06 | Down |
| MUC20 | -1.177611471 | 6.944579176 | 2.83E-14 | 1.38E-13 | Down |
| TNFRSF17 | -1.177656498 | 3.632844029 | 4.95E-10 | 1.69E-09 | Down |
| LHX2 | -1.178293204 | 1.025257355 | 6.26E-10 | 2.12E-09 | Down |
| LCT | -1.178479857 | 1.201154138 | 1.17E-06 | 2.98E-06 | Down |
| CPS1 | -1.179641512 | 4.507882305 | 0.001149022 | 0.002149874 | Down |
| FOXI3 | -1.179756241 | 0.994736436 | 1.00E-07 | 2.79E-07 | Down |
| XYLB | -1.180383356 | 4.376936619 | 1.48E-38 | 3.84E-37 | Down |
| GPR35 | -1.182420678 | 4.392006724 | 9.87E-12 | 3.91E-11 | Down |
| TFAP2D | -1.182664963 | 1.063996926 | 6.09E-07 | 1.59E-06 | Down |
| BCAN | -1.183800614 | 2.086991345 | 8.78E-10 | 2.94E-09 | Down |
| ABCB4 | -1.186819388 | 2.789296309 | 4.04E-14 | 1.94E-13 | Down |
| FAM83B | -1.187925314 | 2.128725087 | 6.06E-06 | 1.44E-05 | Down |
| HCN2 | -1.188123249 | 2.46345388 | 2.64E-12 | 1.10E-11 | Down |
| MTBP | -1.18910843 | 4.29504352 | 1.03E-32 | 1.83E-31 | Down |
| AQP11 | -1.190001102 | 3.024329293 | 2.06E-37 | 4.92E-36 | Down |
| KLHDC8A | -1.190336592 | 2.754267188 | 1.88E-12 | 7.92E-12 | Down |
| ATAD5 | -1.192720578 | 4.474105568 | 6.27E-32 | 1.06E-30 | Down |
| DNASE1 | -1.192922696 | 4.361119527 | 2.15E-39 | 5.88E-38 | Down |
| GPR37 | -1.194238029 | 4.053397129 | 3.85E-10 | 1.33E-09 | Down |
| RNFT2 | -1.198494156 | 4.104365318 | 4.82E-20 | 3.56E-19 | Down |
| DDN | -1.199036987 | 1.894228642 | 2.84E-13 | 1.28E-12 | Down |
| AGR2 | -1.200194456 | 8.569625615 | 2.58E-11 | 9.84E-11 | Down |
| RFX8 | -1.200650798 | 1.315076083 | 5.26E-14 | 2.51E-13 | Down |
| IL17REL | -1.201803588 | 1.381042023 | 6.31E-10 | 2.14E-09 | Down |
| SRD5A1 | -1.201941755 | 5.838272058 | 4.28E-39 | 1.14E-37 | Down |
| NKX3-2 | -1.204003988 | 1.125120667 | 9.75E-12 | 3.87E-11 | Down |

| | | | | | |
|---|---|---|---|---|---|
| NUP210L | -1.204374828 | 2.107633281 | 6.23E-08 | 1.77E-07 | Down |
| MAGED4 | -1.20538106 | 4.291217551 | 5.70E-14 | 2.71E-13 | Down |
| PDX1 | -1.205990283 | 1.09109792 | 2.19E-06 | 5.44E-06 | Down |
| PTHLH | -1.206022838 | 3.723849683 | 9.46E-10 | 3.15E-09 | Down |
| CEACAM1 | -1.208304804 | 6.716469379 | 1.50E-24 | 1.51E-23 | Down |
| CECR7 | -1.209052672 | 2.303618576 | 3.48E-08 | 1.01E-07 | Down |
| HAL | -1.209660903 | 3.630144208 | 2.59E-07 | 6.97E-07 | Down |
| GLRA3 | -1.212957039 | 1.05119083 | 3.07E-09 | 9.81E-09 | Down |
| FADS6 | -1.2130204 | 1.038831861 | 4.01E-07 | 1.06E-06 | Down |
| CACNA1E | -1.213948973 | 1.44721874 | 1.63E-12 | 6.88E-12 | Down |
| RAMP1 | -1.21467295 | 5.993673618 | 1.56E-16 | 8.94E-16 | Down |
| ARL14 | -1.215023913 | 1.659718654 | 3.51E-06 | 8.55E-06 | Down |
| MYBPC2 | -1.215940936 | 2.198471323 | 1.22E-09 | 4.02E-09 | Down |
| GABRA3 | -1.21675926 | 1.076131456 | 4.56E-06 | 1.10E-05 | Down |
| CLDN14 | -1.217184195 | 1.498254442 | 5.78E-16 | 3.18E-15 | Down |
| SALL1 | -1.217292344 | 1.150385478 | 3.69E-06 | 8.97E-06 | Down |
| FCRLA | -1.219791285 | 3.659924 | 6.64E-10 | 2.25E-09 | Down |
| EPHX3 | -1.219981511 | 5.3541346 | 3.14E-14 | 1.52E-13 | Down |
| CILP | -1.220209524 | 5.022499727 | 2.72E-09 | 8.75E-09 | Down |
| KRTAP5-1 | -1.220780733 | 2.080382578 | 6.48E-16 | 3.55E-15 | Down |
| STK31 | -1.22124718 | 3.774882055 | 1.42E-12 | 6.02E-12 | Down |
| C14orf105 | -1.222158209 | 1.109187373 | 2.41E-07 | 6.49E-07 | Down |
| SLC6A11 | -1.222482116 | 1.043008202 | 1.32E-07 | 3.65E-07 | Down |
| LMNB1 | -1.224436385 | 6.582558862 | 6.62E-39 | 1.73E-37 | Down |
| KIAA1024 | -1.227969364 | 3.216913254 | 8.80E-30 | 1.28E-28 | Down |
| CCR8 | -1.228690234 | 2.272553969 | 1.60E-18 | 1.06E-17 | Down |
| KCNJ10 | -1.22904624 | 2.654367187 | 1.23E-12 | 5.24E-12 | Down |
| LOC286467 | -1.22934818 | 1.488984191 | 8.52E-10 | 2.86E-09 | Down |
| C8ORFK29 | -1.229567022 | 1.210692219 | 6.80E-18 | 4.29E-17 | Down |
| LOC554202 | -1.229802964 | 1.731624532 | 7.04E-07 | 1.82E-06 | Down |
| DNMT3B | -1.230985972 | 4.291262957 | 1.78E-23 | 1.65E-22 | Down |
| NOX1 | -1.23190912 | 1.557921047 | 4.09E-10 | 1.41E-09 | Down |
| TDO2 | -1.232853639 | 4.032062243 | 4.39E-14 | 2.10E-13 | Down |
| HIST1H1E | -1.232952677 | 1.374185259 | 6.52E-18 | 4.12E-17 | Down |
| DEPDC7 | -1.239018975 | 3.344195025 | 1.23E-13 | 5.69E-13 | Down |
| IGLON5 | -1.239032407 | 1.787244477 | 1.77E-15 | 9.43E-15 | Down |
| C3orf67 | -1.239041396 | 3.527702965 | 6.70E-18 | 4.23E-17 | Down |
| IL4I1 | -1.239478637 | 5.532542637 | 8.59E-21 | 6.70E-20 | Down |
| EGF | -1.239591108 | 4.430258927 | 1.18E-10 | 4.25E-10 | Down |
| DLX5 | -1.239638037 | 2.079222235 | 5.39E-11 | 2.00E-10 | Down |
| ELAVL2 | -1.240390991 | 1.429665295 | 2.96E-08 | 8.70E-08 | Down |
| SRCIN1 | -1.240839448 | 5.005533328 | 2.41E-15 | 1.27E-14 | Down |
| ANO7 | -1.241538731 | 3.341979215 | 4.05E-34 | 7.88E-33 | Down |
| TNFRSF13C | -1.241659336 | 2.06627863 | 9.26E-14 | 4.33E-13 | Down |
| FOXH1 | -1.242495067 | 1.324423517 | 6.70E-13 | 2.93E-12 | Down |
| BOP1 | -1.242982619 | 5.490575728 | 1.33E-24 | 1.34E-23 | Down |

| | | | | | |
|---|---|---|---|---|---|
| PAICS | -1.243199097 | 7.680957687 | 1.46E-65 | 2.49E-63 | Down |
| BIK | -1.243278347 | 4.475363934 | 1.50E-20 | 1.15E-19 | Down |
| BCMO1 | -1.243903168 | 2.95459423 | 2.16E-09 | 6.99E-09 | Down |
| MC1R | -1.243985971 | 5.02344383 | 3.07E-31 | 4.97E-30 | Down |
| RAD54B | -1.244923003 | 4.433277148 | 1.15E-36 | 2.64E-35 | Down |
| KIAA1524 | -1.245321408 | 4.753930487 | 1.62E-24 | 1.63E-23 | Down |
| GSG2 | -1.245542473 | 3.730660247 | 5.56E-28 | 7.14E-27 | Down |
| BCL2L10 | -1.246980052 | 1.026935289 | 6.89E-13 | 3.01E-12 | Down |
| WDR86 | -1.247066507 | 3.778920369 | 7.53E-11 | 2.76E-10 | Down |
| POU2AF1 | -1.247908154 | 5.602310164 | 1.01E-12 | 4.34E-12 | Down |
| COL6A4P2 | -1.248021921 | 1.472417108 | 2.78E-16 | 1.57E-15 | Down |
| CHTF18 | -1.24865717 | 5.498354934 | 3.66E-32 | 6.26E-31 | Down |
| ARHGAP11A | -1.249078308 | 5.5382157 | 6.46E-31 | 1.01E-29 | Down |
| NPC1L1 | -1.249324059 | 2.291440839 | 6.67E-07 | 1.73E-06 | Down |
| TMEM132A | -1.251039061 | 6.716339658 | 3.18E-28 | 4.15E-27 | Down |
| LAD1 | -1.252981582 | 7.721367268 | 1.29E-30 | 1.97E-29 | Down |
| VTCN1 | -1.253053986 | 3.803495854 | 1.19E-06 | 3.02E-06 | Down |
| ERO1L | -1.25322092 | 7.593181905 | 1.70E-34 | 3.39E-33 | Down |
| TMEM105 | -1.253225003 | 3.415504597 | 1.19E-15 | 6.41E-15 | Down |
| KPNA2 | -1.254421826 | 7.463621072 | 1.11E-38 | 2.87E-37 | Down |
| MCM2 | -1.254495952 | 6.727733098 | 2.45E-37 | 5.83E-36 | Down |
| COL5A1 | -1.256145879 | 8.300951339 | 5.95E-22 | 5.04E-21 | Down |
| ACMSD | -1.256200826 | 1.14548108 | 1.02E-07 | 2.85E-07 | Down |
| FKBP11 | -1.256636165 | 6.691827601 | 3.87E-39 | 1.04E-37 | Down |
| MYCN | -1.257072092 | 2.81215975 | 1.47E-08 | 4.42E-08 | Down |
| SLC35F2 | -1.258873034 | 6.562767771 | 1.17E-44 | 4.43E-43 | Down |
| C1orf112 | -1.259308227 | 5.080988639 | 1.10E-55 | 8.75E-54 | Down |
| SLC16A9 | -1.260852188 | 5.030173034 | 4.12E-08 | 1.19E-07 | Down |
| IVL | -1.261318666 | 2.727939951 | 0.000190687 | 0.000388756 | Down |
| FAM133A | -1.264442375 | 1.649756224 | 3.43E-05 | 7.56E-05 | Down |
| CD79A | -1.265617587 | 5.760375633 | 2.77E-12 | 1.15E-11 | Down |
| SPINK2 | -1.26637083 | 1.728294679 | 1.61E-10 | 5.73E-10 | Down |
| CCL7 | -1.266901304 | 2.031977603 | 1.14E-09 | 3.76E-09 | Down |
| KIAA1875 | -1.269054148 | 2.779285803 | 2.36E-12 | 9.85E-12 | Down |
| HOTAIR | -1.269132323 | 1.06895149 | 1.51E-08 | 4.56E-08 | Down |
| ALDH3B2 | -1.270721893 | 4.763028468 | 6.94E-13 | 3.03E-12 | Down |
| PRSS1 | -1.273673275 | 1.311669509 | 3.88E-06 | 9.40E-06 | Down |
| RUFY4 | -1.273716394 | 2.42419422 | 6.67E-12 | 2.69E-11 | Down |
| C7orf10 | -1.274868513 | 3.397623175 | 1.09E-16 | 6.30E-16 | Down |
| MFSD2B | -1.275240615 | 3.645279773 | 7.10E-31 | 1.11E-29 | Down |
| TNNT1 | -1.275949031 | 4.274314203 | 3.85E-05 | 8.45E-05 | Down |
| ANXA10 | -1.275974151 | 1.052635614 | 1.34E-05 | 3.08E-05 | Down |
| FA2H | -1.276117563 | 5.65733995 | 2.73E-17 | 1.65E-16 | Down |
| APOBEC3B | -1.27657839 | 4.760969403 | 1.90E-14 | 9.35E-14 | Down |
| MAGEA2 | -1.27672526 | 1.22845058 | 0.000177308 | 0.000362798 | Down |
| TEKT5 | -1.277645255 | 1.281696789 | 2.99E-15 | 1.56E-14 | Down |

| | | | | | |
|---|---|---|---|---|---|
| MUC21 | -1.278700607 | 5.907616688 | 5.99E-05 | 0.000128691 | Down |
| PRB3 | -1.278976003 | 1.084896027 | 1.68E-12 | 7.11E-12 | Down |
| TNFRSF21 | -1.280101228 | 8.08968892 | 6.86E-44 | 2.51E-42 | Down |
| MGC29506 | -1.281445944 | 6.052816643 | 3.36E-12 | 1.38E-11 | Down |
| COL5A2 | -1.281809659 | 7.838497545 | 1.35E-23 | 1.27E-22 | Down |
| EDN2 | -1.282430952 | 3.670906509 | 8.13E-09 | 2.50E-08 | Down |
| C8orf77 | -1.283462645 | 1.806311764 | 1.38E-22 | 1.22E-21 | Down |
| SRMS | -1.285321015 | 1.276159756 | 3.53E-10 | 1.22E-09 | Down |
| PLAU | -1.285502628 | 7.542353667 | 1.05E-16 | 6.04E-16 | Down |
| PPAT | -1.286828382 | 5.794173265 | 8.07E-61 | 9.52E-59 | Down |
| RTBDN | -1.289205567 | 1.099504307 | 3.83E-09 | 1.21E-08 | Down |
| DSP | -1.289614073 | 8.449573926 | 7.48E-17 | 4.36E-16 | Down |
| ADAM8 | -1.291580938 | 6.78394523 | 1.09E-26 | 1.28E-25 | Down |
| TWIST1 | -1.29549945 | 2.949669207 | 9.12E-11 | 3.31E-10 | Down |
| DBNDD1 | -1.295960092 | 6.268486714 | 1.68E-40 | 4.95E-39 | Down |
| SLC6A10P | -1.297417714 | 1.322409581 | 5.69E-13 | 2.50E-12 | Down |
| C12orf36 | -1.297947061 | 1.247469003 | 6.86E-07 | 1.78E-06 | Down |
| C12orf48 | -1.299618851 | 4.51026016 | 1.24E-32 | 2.18E-31 | Down |
| FCRL2 | -1.302740582 | 3.264460124 | 4.31E-11 | 1.61E-10 | Down |
| GSDMB | -1.304528289 | 5.401396352 | 4.68E-23 | 4.25E-22 | Down |
| PFKP | -1.304607555 | 7.694761841 | 6.39E-30 | 9.32E-29 | Down |
| MCHR1 | -1.305011216 | 2.179455058 | 1.30E-16 | 7.49E-16 | Down |
| ATP6V1C2 | -1.305750706 | 4.93629783 | 1.83E-22 | 1.60E-21 | Down |
| ALOXE3 | -1.305859046 | 1.460939309 | 3.05E-14 | 1.48E-13 | Down |
| HOXC8 | -1.308807697 | 1.938619357 | 3.33E-09 | 1.06E-08 | Down |
| IL17C | -1.308964872 | 1.112176151 | 2.85E-11 | 1.08E-10 | Down |
| MARCKSL1 | -1.30948527 | 8.104086109 | 1.27E-46 | 5.55E-45 | Down |
| FAM40B | -1.310122663 | 4.520174966 | 2.36E-15 | 1.24E-14 | Down |
| WNK2 | -1.310703603 | 4.691535675 | 6.34E-12 | 2.56E-11 | Down |
| SRPX2 | -1.314657061 | 5.792572711 | 1.07E-19 | 7.69E-19 | Down |
| PLK4 | -1.317064953 | 4.788133825 | 2.53E-34 | 4.98E-33 | Down |
| DOK5 | -1.317256113 | 3.278409483 | 7.50E-15 | 3.79E-14 | Down |
| GSDMC | -1.317660909 | 3.875565634 | 1.77E-11 | 6.86E-11 | Down |
| RAET1K | -1.31872802 | 1.282742491 | 5.79E-25 | 5.98E-24 | Down |
| KRT15 | -1.319080678 | 5.932471672 | 2.70E-11 | 1.03E-10 | Down |
| FANCA | -1.319319372 | 5.68303187 | 1.49E-43 | 5.34E-42 | Down |
| RAD51AP1 | -1.319500587 | 4.865670494 | 4.11E-29 | 5.70E-28 | Down |
| PANX2 | -1.319648515 | 5.230221071 | 3.21E-17 | 1.93E-16 | Down |
| FAM83F | -1.320775237 | 3.135847505 | 2.74E-07 | 7.35E-07 | Down |
| KIF1A | -1.322164365 | 3.034334814 | 0.000562326 | 0.001091427 | Down |
| INSL3 | -1.322166701 | 1.416984776 | 3.71E-27 | 4.51E-26 | Down |
| UCN2 | -1.32221274 | 1.172084229 | 2.26E-12 | 9.46E-12 | Down |
| ARL9 | -1.322664599 | 1.496586886 | 1.06E-13 | 4.94E-13 | Down |
| WNT3 | -1.323477155 | 3.721980161 | 3.82E-32 | 6.53E-31 | Down |
| GPR81 | -1.323806479 | 3.678482448 | 1.30E-08 | 3.93E-08 | Down |
| CTSL2 | -1.325231002 | 4.249411675 | 1.47E-13 | 6.76E-13 | Down |

| | | | | | |
|---|---|---|---|---|---|
| SERINC2 | -1.325442189 | 8.018263596 | 2.16E-43 | 7.65E-42 | Down |
| XKR9 | -1.326608481 | 3.772325098 | 2.59E-13 | 1.17E-12 | Down |
| LY6K | -1.326799664 | 4.075386111 | 8.87E-06 | 2.08E-05 | Down |
| CYP2D6 | -1.327107093 | 2.921474159 | 1.46E-11 | 5.68E-11 | Down |
| GCSH | -1.32817609 | 2.297360208 | 3.62E-21 | 2.89E-20 | Down |
| RHCE | -1.330102829 | 2.522238875 | 2.52E-14 | 1.23E-13 | Down |
| KRT14 | -1.330942523 | 1.98366457 | 2.65E-06 | 6.54E-06 | Down |
| TNFRSF25 | -1.331150489 | 4.713374056 | 5.43E-22 | 4.60E-21 | Down |
| SC65 | -1.331412664 | 6.396005378 | 8.76E-46 | 3.61E-44 | Down |
| TRIM46 | -1.332929239 | 4.012976148 | 3.74E-26 | 4.25E-25 | Down |
| HES6 | -1.333714262 | 5.366864117 | 2.63E-18 | 1.71E-17 | Down |
| CHEK1 | -1.333938311 | 5.08918962 | 7.26E-38 | 1.79E-36 | Down |
| SPOCK1 | -1.333971076 | 4.499441952 | 1.58E-10 | 5.62E-10 | Down |
| LRRC15 | -1.334637017 | 5.492461563 | 1.61E-11 | 6.28E-11 | Down |
| EPHB2 | -1.336168108 | 6.127374954 | 7.13E-23 | 6.39E-22 | Down |
| LCN2 | -1.337353733 | 7.139923638 | 1.01E-09 | 3.37E-09 | Down |
| FUT3 | -1.338384245 | 6.113707906 | 1.45E-21 | 1.19E-20 | Down |
| CCDC154 | -1.338400405 | 1.866236202 | 2.54E-12 | 1.06E-11 | Down |
| LINGO1 | -1.339233876 | 3.989218958 | 1.57E-18 | 1.04E-17 | Down |
| DIRAS1 | -1.340497305 | 4.037674372 | 4.67E-12 | 1.90E-11 | Down |
| DUSP5P | -1.340532294 | 1.559007126 | 3.01E-12 | 1.25E-11 | Down |
| ABCC2 | -1.340684881 | 2.941644247 | 1.55E-06 | 3.90E-06 | Down |
| CCNF | -1.341128846 | 5.683207035 | 5.41E-48 | 2.51E-46 | Down |
| TMEM171 | -1.341722255 | 2.414531501 | 2.73E-11 | 1.04E-10 | Down |
| ECT2 | -1.34494813 | 6.640393916 | 6.12E-40 | 1.73E-38 | Down |
| LBX2 | -1.345615425 | 2.362252247 | 2.78E-28 | 3.65E-27 | Down |
| HAMP | -1.346604835 | 2.514467771 | 5.17E-13 | 2.28E-12 | Down |
| RAC3 | -1.347467118 | 5.137389533 | 2.87E-26 | 3.30E-25 | Down |
| PCSK1 | -1.348394741 | 2.639201921 | 7.76E-06 | 1.83E-05 | Down |
| CDK5R2 | -1.35223933 | 1.377131681 | 2.94E-09 | 9.42E-09 | Down |
| GREB1 | -1.352348571 | 4.419914637 | 1.00E-15 | 5.42E-15 | Down |
| ADAM6 | -1.352644977 | 11.04985249 | 1.60E-13 | 7.36E-13 | Down |
| TCAM1P | -1.35329739 | 1.398730039 | 5.40E-08 | 1.55E-07 | Down |
| C4orf48 | -1.353483027 | 3.791003347 | 4.94E-20 | 3.64E-19 | Down |
| TTYH1 | -1.354242013 | 1.623891234 | 9.01E-16 | 4.89E-15 | Down |
| IGFBP3 | -1.356321431 | 8.366579063 | 2.40E-22 | 2.09E-21 | Down |
| HPCA | -1.358037416 | 1.4906052 | 1.06E-20 | 8.23E-20 | Down |
| SRRM3 | -1.358412033 | 3.987493433 | 6.63E-24 | 6.36E-23 | Down |
| LEMD1 | -1.359038088 | 2.738982787 | 7.62E-09 | 2.35E-08 | Down |
| ARHGAP11B | -1.360667897 | 2.725121494 | 3.34E-25 | 3.53E-24 | Down |
| CCDC64 | -1.361132101 | 5.653517655 | 5.74E-29 | 7.90E-28 | Down |
| GRIK2 | -1.361708727 | 2.314743352 | 8.76E-10 | 2.93E-09 | Down |
| MCM4 | -1.361871828 | 6.929944296 | 1.58E-41 | 4.99E-40 | Down |
| TESC | -1.362632498 | 6.042016133 | 2.36E-08 | 6.99E-08 | Down |
| CRLF1 | -1.3634605 | 5.652166395 | 4.88E-06 | 1.17E-05 | Down |
| SYT2 | -1.365564669 | 3.618505944 | 3.21E-11 | 1.21E-10 | Down |

| | | | | | |
|---|---|---|---|---|---|
| RPL39L | -1.369753419 | 5.196671533 | 4.18E-23 | 3.80E-22 | Down |
| CRYGN | -1.369940574 | 1.178846503 | 9.54E-13 | 4.11E-12 | Down |
| DDX12 | -1.370780393 | 4.3252092 | 1.92E-34 | 3.80E-33 | Down |
| CHAF1B | -1.371273461 | 5.145839467 | 7.29E-53 | 4.64E-51 | Down |
| AKR7L | -1.371377145 | 2.553990996 | 7.32E-14 | 3.45E-13 | Down |
| MGC87042 | -1.372085171 | 3.236377576 | 9.01E-20 | 6.52E-19 | Down |
| C1orf65 | -1.37257705 | 1.152571271 | 2.40E-15 | 1.26E-14 | Down |
| KIRREL2 | -1.372628167 | 1.399908128 | 6.25E-09 | 1.94E-08 | Down |
| HMGA1 | -1.37384825 | 8.049346083 | 4.40E-39 | 1.17E-37 | Down |
| FANCI | -1.376813273 | 6.212669083 | 4.55E-50 | 2.45E-48 | Down |
| UPK3A | -1.376897165 | 1.897468004 | 4.56E-10 | 1.56E-09 | Down |
| CKMT1B | -1.379657833 | 4.956274859 | 2.54E-09 | 8.17E-09 | Down |
| ARTN | -1.380268049 | 2.893964711 | 1.14E-17 | 7.09E-17 | Down |
| PPFIA4 | -1.380462662 | 3.810446611 | 4.73E-16 | 2.62E-15 | Down |
| TOX3 | -1.38154436 | 5.451319572 | 3.41E-09 | 1.09E-08 | Down |
| C1orf106 | -1.382715169 | 7.094745936 | 4.79E-31 | 7.60E-30 | Down |
| HSF2BP | -1.385197473 | 2.907672237 | 5.62E-28 | 7.21E-27 | Down |
| FNDC1 | -1.387000299 | 5.87348077 | 1.24E-15 | 6.64E-15 | Down |
| NCCRP1 | -1.387801082 | 3.221546697 | 2.88E-08 | 8.46E-08 | Down |
| PNCK | -1.3897907 | 1.638133686 | 1.15E-09 | 3.81E-09 | Down |
| CHRNB4 | -1.390128289 | 1.255053838 | 2.94E-17 | 1.77E-16 | Down |
| INA | -1.391361347 | 1.824605494 | 5.44E-07 | 1.42E-06 | Down |
| POU3F2 | -1.391484609 | 1.237809417 | 9.01E-07 | 2.32E-06 | Down |
| DTL | -1.392027231 | 5.437488268 | 4.17E-35 | 8.69E-34 | Down |
| LCN12 | -1.394417441 | 2.354081326 | 6.68E-16 | 3.66E-15 | Down |
| WISP1 | -1.394564675 | 4.805062309 | 1.46E-25 | 1.58E-24 | Down |
| ECE2 | -1.394803935 | 5.285199968 | 1.97E-48 | 9.38E-47 | Down |
| DNAH14 | -1.397814391 | 4.538060537 | 6.28E-55 | 4.62E-53 | Down |
| CENPM | -1.399079053 | 4.758881163 | 2.52E-35 | 5.31E-34 | Down |
| RGS20 | -1.399505872 | 2.257736252 | 6.07E-11 | 2.24E-10 | Down |
| IL23A | -1.40341181 | 3.087575197 | 2.58E-23 | 2.37E-22 | Down |
| PTPRN | -1.406632404 | 1.929364949 | 6.22E-08 | 1.77E-07 | Down |
| ADAM28 | -1.409231077 | 5.669383188 | 3.25E-25 | 3.44E-24 | Down |
| OCIAD2 | -1.411236205 | 7.435092031 | 6.48E-54 | 4.43E-52 | Down |
| C3orf57 | -1.411630722 | 2.601380227 | 5.58E-07 | 1.46E-06 | Down |
| GFAP | -1.412944846 | 1.64927268 | 1.94E-12 | 8.14E-12 | Down |
| FAM72B | -1.413635456 | 4.470992027 | 1.10E-26 | 1.30E-25 | Down |
| SLC25A10 | -1.416214536 | 6.521691447 | 9.23E-57 | 7.86E-55 | Down |
| MYBPH | -1.417269684 | 2.017011716 | 1.03E-07 | 2.89E-07 | Down |
| TRAIP | -1.417649212 | 4.253022728 | 3.41E-50 | 1.85E-48 | Down |
| CBX2 | -1.419981285 | 5.272676502 | 2.16E-21 | 1.75E-20 | Down |
| SPERT | -1.421515104 | 1.191254595 | 2.87E-11 | 1.09E-10 | Down |
| MESP1 | -1.423124545 | 3.885561929 | 5.42E-22 | 4.60E-21 | Down |
| EN2 | -1.423544266 | 1.346386275 | 1.52E-10 | 5.42E-10 | Down |
| RCC1 | -1.423777948 | 6.767689228 | 2.31E-64 | 3.77E-62 | Down |
| PRR19 | -1.424144052 | 3.471662515 | 1.61E-43 | 5.72E-42 | Down |

| | | | | | |
|---|---|---|---|---|---|
| KDELR3 | -1.425120919 | 6.695898192 | 1.65E-45 | 6.75E-44 | Down |
| COL24A1 | -1.42799349 | 3.503135605 | 9.52E-31 | 1.47E-29 | Down |
| AIM2 | -1.4282635 | 4.218513625 | 1.67E-12 | 7.06E-12 | Down |
| MLF1IP | -1.429120449 | 5.534901756 | 1.39E-39 | 3.86E-38 | Down |
| METTL7B | -1.431547621 | 5.229995491 | 8.59E-18 | 5.38E-17 | Down |
| ZPLD1 | -1.432352508 | 1.283589845 | 2.05E-13 | 9.32E-13 | Down |
| PPP1R14B | -1.432398716 | 7.39178216 | 1.14E-66 | 2.14E-64 | Down |
| TMEM145 | -1.432796824 | 1.457888765 | 1.94E-14 | 9.52E-14 | Down |
| TYMS | -1.433600568 | 6.094093828 | 7.33E-42 | 2.37E-40 | Down |
| CCNE2 | -1.434359893 | 4.794999243 | 9.48E-32 | 1.58E-30 | Down |
| C1orf135 | -1.434533632 | 3.545300085 | 1.89E-30 | 2.86E-29 | Down |
| RAB3B | -1.437203583 | 1.321248477 | 7.50E-11 | 2.75E-10 | Down |
| KCNK12 | -1.438059573 | 1.275339128 | 1.76E-14 | 8.68E-14 | Down |
| CKMT1A | -1.438337376 | 3.836773418 | 3.25E-09 | 1.04E-08 | Down |
| CDKN2A | -1.43925713 | 4.78196531 | 3.13E-10 | 1.09E-09 | Down |
| NAT8L | -1.441296396 | 2.356606191 | 2.05E-12 | 8.60E-12 | Down |
| LOC96610 | -1.442133695 | 10.1979319 | 7.55E-16 | 4.12E-15 | Down |
| XKRX | -1.442750793 | 4.880478364 | 6.83E-15 | 3.47E-14 | Down |
| SLC17A9 | -1.442963314 | 5.4672577 | 2.77E-23 | 2.54E-22 | Down |
| MTL5 | -1.447136758 | 3.75836329 | 8.70E-17 | 5.04E-16 | Down |
| ANKS4B | -1.447766639 | 1.269821461 | 9.69E-07 | 2.48E-06 | Down |
| C8orf73 | -1.447849662 | 6.212450855 | 1.43E-25 | 1.56E-24 | Down |
| HIST1H2AE | -1.448629829 | 2.213124987 | 5.39E-11 | 2.00E-10 | Down |
| FBXO43 | -1.449004778 | 2.219714946 | 1.13E-30 | 1.73E-29 | Down |
| IL20RB | -1.450147248 | 3.655843481 | 1.97E-11 | 7.61E-11 | Down |
| NRK | -1.452772606 | 3.675958888 | 2.46E-09 | 7.94E-09 | Down |
| SIX2 | -1.453122852 | 3.481153813 | 5.10E-09 | 1.60E-08 | Down |
| NKAIN4 | -1.454113269 | 1.620139493 | 1.54E-12 | 6.52E-12 | Down |
| NKPD1 | -1.454238205 | 1.657286092 | 3.25E-23 | 2.97E-22 | Down |
| GYLTL1B | -1.454786076 | 6.011812583 | 1.95E-23 | 1.81E-22 | Down |
| FAM72D | -1.45741443 | 3.794452328 | 2.12E-23 | 1.96E-22 | Down |
| PAFAH1B3 | -1.45765345 | 6.430488147 | 1.01E-57 | 9.19E-56 | Down |
| LGI2 | -1.457971723 | 4.932717916 | 2.66E-21 | 2.15E-20 | Down |
| SBK1 | -1.461696557 | 5.954566223 | 1.15E-22 | 1.02E-21 | Down |
| FHL2 | -1.463468565 | 6.711991308 | 1.35E-26 | 1.58E-25 | Down |
| CKM | -1.463743738 | 1.952182509 | 8.38E-12 | 3.35E-11 | Down |
| ABCA4 | -1.46442196 | 4.229974639 | 5.96E-08 | 1.70E-07 | Down |
| C1QTNF6 | -1.464643311 | 6.115559761 | 3.08E-41 | 9.60E-40 | Down |
| UCHL1 | -1.46496576 | 5.757850143 | 3.79E-10 | 1.31E-09 | Down |
| VSIG1 | -1.470271292 | 4.626821041 | 5.54E-07 | 1.45E-06 | Down |
| FOXA3 | -1.470887736 | 4.075656321 | 1.35E-11 | 5.28E-11 | Down |
| SP8 | -1.474960352 | 1.310054388 | 5.62E-09 | 1.76E-08 | Down |
| FAP | -1.476529814 | 5.877912656 | 2.58E-28 | 3.39E-27 | Down |
| PABPC1L | -1.479043238 | 5.75791053 | 5.74E-25 | 5.93E-24 | Down |
| LOC647946 | -1.480197828 | 1.499051489 | 7.60E-17 | 4.43E-16 | Down |
| HIST1H3D | -1.48254196 | 1.972087115 | 1.61E-14 | 7.98E-14 | Down |

| | | | | | |
|---|---|---|---|---|---|
| HGD | -1.483907891 | 4.288896338 | 3.25E-07 | 8.68E-07 | Down |
| OVOL1 | -1.486594038 | 4.481484483 | 1.12E-22 | 9.95E-22 | Down |
| KRT6C | -1.488281928 | 1.899415746 | 8.65E-07 | 2.22E-06 | Down |
| CATSPERB | -1.490252446 | 2.656159794 | 1.17E-11 | 4.61E-11 | Down |
| PAX9 | -1.492617748 | 4.640987114 | 3.73E-15 | 1.93E-14 | Down |
| CCDC129 | -1.493439059 | 1.787046622 | 1.30E-07 | 3.61E-07 | Down |
| C12orf56 | -1.493616613 | 1.343975141 | 1.28E-08 | 3.88E-08 | Down |
| EFNA4 | -1.493683975 | 5.819097949 | 1.63E-66 | 3.02E-64 | Down |
| HOXC9 | -1.496823631 | 2.158483993 | 2.54E-10 | 8.91E-10 | Down |
| GYG2 | -1.497933528 | 5.127839482 | 7.08E-28 | 9.04E-27 | Down |
| ARNTL2 | -1.498075569 | 4.67008744 | 2.22E-19 | 1.56E-18 | Down |
| GPR19 | -1.499166521 | 2.599847093 | 3.18E-28 | 4.15E-27 | Down |
| KCNQ3 | -1.500892255 | 4.180594498 | 9.91E-19 | 6.66E-18 | Down |
| ASPHD1 | -1.502003098 | 4.398321272 | 1.73E-16 | 9.87E-16 | Down |
| SLC44A5 | -1.502045894 | 4.067749549 | 1.74E-08 | 5.22E-08 | Down |
| PVRL4 | -1.503912205 | 6.883701579 | 9.10E-35 | 1.84E-33 | Down |
| C1orf220 | -1.50464868 | 2.013572536 | 3.07E-33 | 5.62E-32 | Down |
| HECW1 | -1.507164906 | 2.508799276 | 6.80E-18 | 4.29E-17 | Down |
| PIF1 | -1.507549574 | 3.647748704 | 1.25E-28 | 1.68E-27 | Down |
| SULF1 | -1.508694008 | 7.57417591 | 1.77E-24 | 1.77E-23 | Down |
| TMEM156 | -1.509433614 | 3.951549393 | 7.55E-19 | 5.12E-18 | Down |
| ABCC3 | -1.509645068 | 8.024710548 | 4.39E-26 | 4.97E-25 | Down |
| SERPINA4 | -1.510430362 | 1.251979375 | 1.36E-07 | 3.76E-07 | Down |
| MYO7A | -1.510485505 | 5.460933723 | 3.90E-50 | 2.10E-48 | Down |
| C2orf48 | -1.510610343 | 1.46533896 | 8.64E-21 | 6.73E-20 | Down |
| BPIL1 | -1.511776458 | 2.221814119 | 3.09E-05 | 6.85E-05 | Down |
| CDK1 | -1.513943077 | 6.127997617 | 6.37E-35 | 1.31E-33 | Down |
| FAM83D | -1.514297756 | 5.311150662 | 3.91E-31 | 6.26E-30 | Down |
| FLJ40330 | -1.517154588 | 3.765700689 | 2.57E-14 | 1.25E-13 | Down |
| CPNE4 | -1.517572356 | 2.844954421 | 9.05E-12 | 3.60E-11 | Down |
| FAM72A | -1.517765598 | 2.793749549 | 2.11E-24 | 2.10E-23 | Down |
| MMP17 | -1.518385759 | 4.293643351 | 1.80E-19 | 1.28E-18 | Down |
| KNTC1 | -1.519316196 | 5.923803382 | 2.64E-52 | 1.64E-50 | Down |
| NME1 | -1.519916213 | 7.374804538 | 7.33E-63 | 1.05E-60 | Down |
| BLM | -1.520641326 | 4.756334783 | 1.11E-47 | 5.04E-46 | Down |
| FBXO32 | -1.521883874 | 5.924401422 | 1.28E-37 | 3.13E-36 | Down |
| KRT81 | -1.523182426 | 3.35016925 | 9.04E-10 | 3.02E-09 | Down |
| TLL2 | -1.526091958 | 1.932871026 | 3.43E-18 | 2.22E-17 | Down |
| C9orf84 | -1.52853163 | 2.26966683 | 4.95E-12 | 2.01E-11 | Down |
| GINS4 | -1.528845032 | 4.807592622 | 2.60E-41 | 8.16E-40 | Down |
| RTN4RL2 | -1.53096944 | 4.476436084 | 6.51E-18 | 4.11E-17 | Down |
| SIX4 | -1.531266867 | 5.597756989 | 1.13E-43 | 4.09E-42 | Down |
| SOX11 | -1.531649791 | 2.184191004 | 3.90E-09 | 1.24E-08 | Down |
| FOXP3 | -1.532013115 | 4.108129832 | 3.84E-33 | 6.95E-32 | Down |
| TRIM15 | -1.532560193 | 1.26933971 | 3.02E-09 | 9.67E-09 | Down |
| AKR1B15 | -1.533685364 | 1.374758064 | 2.28E-08 | 6.77E-08 | Down |

| | | | | | |
|---|---|---|---|---|---|
| DERL3 | -1.534421758 | 6.218135326 | 8.14E-29 | 1.11E-27 | Down |
| ONECUT1 | -1.534509603 | 1.360050734 | 1.66E-16 | 9.48E-16 | Down |
| C5orf34 | -1.534832341 | 3.785295975 | 1.95E-40 | 5.72E-39 | Down |
| PTTG1 | -1.53597178 | 5.774098827 | 2.42E-38 | 6.18E-37 | Down |
| MIOX | -1.536816431 | 1.460042293 | 1.71E-11 | 6.65E-11 | Down |
| CENPK | -1.53906261 | 4.324484995 | 1.09E-37 | 2.67E-36 | Down |
| C2CD4D | -1.540647694 | 2.54527887 | 2.19E-26 | 2.53E-25 | Down |
| C5orf46 | -1.541069572 | 1.331590462 | 8.27E-14 | 3.88E-13 | Down |
| DIO2 | -1.541769304 | 4.905705719 | 2.72E-19 | 1.90E-18 | Down |
| KRT86 | -1.543747181 | 3.177459368 | 3.32E-20 | 2.48E-19 | Down |
| CST2 | -1.547999773 | 2.604651836 | 1.57E-11 | 6.11E-11 | Down |
| OGDHL | -1.548909884 | 2.267091726 | 2.85E-09 | 9.15E-09 | Down |
| TUBB2B | -1.550094568 | 3.74312886 | 2.34E-09 | 7.56E-09 | Down |
| EGLN3 | -1.553369574 | 6.395045422 | 1.92E-19 | 1.36E-18 | Down |
| HIST1H2BD | -1.556217793 | 5.610833998 | 3.42E-40 | 9.80E-39 | Down |
| PODNL1 | -1.557178116 | 4.895359127 | 1.31E-20 | 1.00E-19 | Down |
| EPS8L3 | -1.557698211 | 1.20631864 | 6.35E-07 | 1.65E-06 | Down |
| SHOX2 | -1.559354808 | 2.417462251 | 3.91E-15 | 2.02E-14 | Down |
| PAX7 | -1.56193421 | 1.616404463 | 7.75E-06 | 1.83E-05 | Down |
| LYPD3 | -1.563919268 | 4.885388923 | 5.48E-17 | 3.23E-16 | Down |
| HRASLS | -1.56405635 | 2.48736562 | 7.37E-14 | 3.47E-13 | Down |
| COX6B2 | -1.568024512 | 1.577484368 | 1.15E-16 | 6.62E-16 | Down |
| DNA2 | -1.571356178 | 4.667128344 | 9.23E-49 | 4.49E-47 | Down |
| PACSIN1 | -1.573514895 | 3.219928765 | 3.63E-22 | 3.12E-21 | Down |
| MMP9 | -1.574864469 | 6.878113115 | 8.70E-21 | 6.78E-20 | Down |
| EPYC | -1.574953737 | 1.293483369 | 6.29E-11 | 2.32E-10 | Down |
| SHCBP1 | -1.575708703 | 5.017032222 | 3.75E-42 | 1.23E-40 | Down |
| TRIM54 | -1.579218568 | 1.9578599 | 3.49E-10 | 1.21E-09 | Down |
| WDR62 | -1.584736343 | 4.897374662 | 3.87E-32 | 6.61E-31 | Down |
| NQO1 | -1.585851089 | 8.019097007 | 3.20E-21 | 2.58E-20 | Down |
| KRT80 | -1.586844442 | 6.716745879 | 6.58E-32 | 1.11E-30 | Down |
| C16orf75 | -1.590073331 | 5.435664473 | 1.12E-59 | 1.15E-57 | Down |
| PDIA2 | -1.590716713 | 1.718431177 | 2.83E-09 | 9.09E-09 | Down |
| SLC7A11 | -1.591571573 | 5.811661838 | 3.30E-16 | 1.85E-15 | Down |
| S100A2 | -1.592493476 | 5.327652545 | 3.21E-15 | 1.67E-14 | Down |
| HIST1H2BG | -1.593390069 | 1.892000966 | 4.67E-14 | 2.24E-13 | Down |
| RAD51 | -1.59406454 | 4.495273609 | 1.07E-41 | 3.44E-40 | Down |
| DDIT4L | -1.595053295 | 4.514335036 | 7.47E-17 | 4.35E-16 | Down |
| ASF1B | -1.599879893 | 5.653560642 | 1.07E-48 | 5.15E-47 | Down |
| C9orf100 | -1.601171589 | 4.74964784 | 1.53E-55 | 1.19E-53 | Down |
| SLC7A5 | -1.601372386 | 7.400625309 | 2.09E-33 | 3.86E-32 | Down |
| SGK2 | -1.602529638 | 3.113888471 | 2.01E-15 | 1.06E-14 | Down |
| NFKBIL2 | -1.604116648 | 5.653539779 | 6.00E-52 | 3.67E-50 | Down |
| MSL3L2 | -1.604584535 | 4.876785799 | 7.82E-32 | 1.31E-30 | Down |
| CLDN10 | -1.605316944 | 4.290954663 | 1.69E-07 | 4.63E-07 | Down |
| E2F2 | -1.606378839 | 4.581505697 | 5.73E-45 | 2.25E-43 | Down |

| | | | | | |
|---|---|---|---|---|---|
| PROM2 | -1.60725887 | 7.020946993 | 3.07E-31 | 4.97E-30 | Down |
| CYP27B1 | -1.607943329 | 4.515591151 | 4.58E-38 | 1.15E-36 | Down |
| BHLHA15 | -1.60801873 | 2.792125308 | 3.82E-21 | 3.05E-20 | Down |
| PRR11 | -1.610288631 | 4.0256366 | 1.01E-23 | 9.53E-23 | Down |
| FOXE1 | -1.612860697 | 2.080546443 | 2.63E-08 | 7.77E-08 | Down |
| KRT6B | -1.617842488 | 2.578226064 | 9.06E-07 | 2.33E-06 | Down |
| ANKRD22 | -1.618487174 | 5.728999143 | 4.95E-24 | 4.81E-23 | Down |
| MESP2 | -1.619441672 | 2.251561271 | 6.78E-19 | 4.62E-18 | Down |
| C1orf182 | -1.619842799 | 1.366565646 | 4.05E-25 | 4.24E-24 | Down |
| POU4F1 | -1.621029504 | 1.478934195 | 1.90E-11 | 7.34E-11 | Down |
| MFI2 | -1.621790445 | 4.838921752 | 2.15E-20 | 1.63E-19 | Down |
| LOC150197 | -1.623392704 | 1.791765495 | 6.21E-20 | 4.54E-19 | Down |
| NKAIN1 | -1.624640396 | 1.491809444 | 1.90E-15 | 1.01E-14 | Down |
| MAD2L1 | -1.627810204 | 5.455523058 | 9.90E-41 | 2.95E-39 | Down |
| STYK1 | -1.630551434 | 4.747814793 | 6.40E-31 | 1.01E-29 | Down |
| CDC25A | -1.634068467 | 4.208274975 | 4.30E-39 | 1.15E-37 | Down |
| TNFRSF18 | -1.63412072 | 4.422920582 | 3.28E-26 | 3.74E-25 | Down |
| SLC15A1 | -1.63456066 | 2.364279271 | 2.27E-09 | 7.34E-09 | Down |
| C20orf151 | -1.637093731 | 4.173120143 | 4.25E-35 | 8.85E-34 | Down |
| CDH3 | -1.637767411 | 7.071954692 | 7.68E-19 | 5.20E-18 | Down |
| C13orf38 | -1.640288008 | 1.812099867 | 1.69E-21 | 1.38E-20 | Down |
| C18orf56 | -1.640623038 | 2.115156639 | 3.28E-32 | 5.63E-31 | Down |
| ESPN | -1.642207305 | 4.889381999 | 1.29E-16 | 7.42E-16 | Down |
| KPNA7 | -1.643489964 | 3.462798758 | 6.26E-22 | 5.29E-21 | Down |
| AURKA | -1.644667102 | 5.733486127 | 1.88E-41 | 5.91E-40 | Down |
| PRC1 | -1.64591073 | 6.184732436 | 6.16E-49 | 3.03E-47 | Down |
| C19orf26 | -1.645923946 | 2.595122777 | 5.64E-25 | 5.83E-24 | Down |
| COL3A1 | -1.649717044 | 10.10954592 | 4.06E-30 | 6.01E-29 | Down |
| CLDN6 | -1.649900925 | 2.571734329 | 2.08E-06 | 5.19E-06 | Down |
| C19orf77 | -1.651014573 | 1.791334892 | 4.10E-11 | 1.54E-10 | Down |
| SCN8A | -1.651995109 | 3.07074692 | 2.93E-19 | 2.05E-18 | Down |
| VPREB3 | -1.652477882 | 2.830909277 | 9.30E-21 | 7.23E-20 | Down |
| E2F7 | -1.652877593 | 3.801816762 | 1.39E-28 | 1.86E-27 | Down |
| IL11 | -1.653492812 | 2.512001015 | 1.04E-20 | 8.08E-20 | Down |
| DRP2 | -1.655747183 | 2.11819362 | 1.28E-21 | 1.06E-20 | Down |
| TERT | -1.655988067 | 1.41942944 | 2.34E-17 | 1.42E-16 | Down |
| STEAP1 | -1.658695931 | 5.535450543 | 5.50E-24 | 5.32E-23 | Down |
| BRSK1 | -1.659695942 | 3.691221409 | 5.94E-27 | 7.09E-26 | Down |
| SERPINB5 | -1.661077443 | 3.215425943 | 6.97E-07 | 1.81E-06 | Down |
| DGCR9 | -1.661168575 | 2.980882356 | 1.09E-18 | 7.28E-18 | Down |
| SOHLH2 | -1.668719035 | 1.888535798 | 2.00E-11 | 7.72E-11 | Down |
| CAMK2N2 | -1.670296304 | 2.355635909 | 1.15E-22 | 1.02E-21 | Down |
| CEACAM7 | -1.671358389 | 2.549027944 | 9.75E-12 | 3.87E-11 | Down |
| GAL | -1.671683159 | 1.641126494 | 4.54E-10 | 1.56E-09 | Down |
| ALPK2 | -1.673575837 | 2.909346192 | 2.53E-21 | 2.04E-20 | Down |
| BMP8A | -1.679072469 | 5.169943976 | 6.37E-37 | 1.48E-35 | Down |

| | | | | | |
|---|---|---|---|---|---|
| PHLDA2 | -1.680946373 | 5.962006569 | 9.68E-38 | 2.38E-36 | Down |
| PTGES | -1.682507822 | 6.186099648 | 2.89E-30 | 4.31E-29 | Down |
| MYO3B | -1.692000101 | 2.195343203 | 3.38E-13 | 1.51E-12 | Down |
| GOLGA7B | -1.693539036 | 5.579877603 | 3.32E-24 | 3.27E-23 | Down |
| MAGEA3 | -1.693683845 | 1.578325866 | 5.84E-06 | 1.39E-05 | Down |
| GPRIN1 | -1.695024675 | 4.995613504 | 4.69E-46 | 1.96E-44 | Down |
| FCRL5 | -1.695638917 | 4.804312088 | 7.30E-19 | 4.96E-18 | Down |
| CXCL14 | -1.697671673 | 6.21411383 | 4.00E-10 | 1.38E-09 | Down |
| C4orf7 | -1.699647846 | 2.622822061 | 1.80E-09 | 5.88E-09 | Down |
| RNF186 | -1.703120201 | 1.530715342 | 7.52E-14 | 3.54E-13 | Down |
| P4HA3 | -1.703219484 | 4.420232354 | 1.55E-34 | 3.10E-33 | Down |
| HIST1H2AM | -1.70393433 | 1.525046443 | 8.67E-29 | 1.18E-27 | Down |
| CPXM1 | -1.70642847 | 5.120018222 | 2.63E-30 | 3.94E-29 | Down |
| PVT1 | -1.706513577 | 4.467109458 | 3.56E-50 | 1.92E-48 | Down |
| STIL | -1.707672239 | 5.191639435 | 1.85E-61 | 2.32E-59 | Down |
| UCA1 | -1.707979709 | 1.85434226 | 1.92E-10 | 6.79E-10 | Down |
| RCOR2 | -1.715190311 | 3.637479455 | 1.45E-19 | 1.04E-18 | Down |
| ADAMDEC1 | -1.716751104 | 4.491917786 | 1.80E-15 | 9.57E-15 | Down |
| SLC29A4 | -1.721437824 | 5.489069048 | 1.18E-22 | 1.04E-21 | Down |
| EPHX4 | -1.721679167 | 3.555901416 | 3.49E-21 | 2.79E-20 | Down |
| TNS4 | -1.722210512 | 4.926047411 | 2.31E-11 | 8.87E-11 | Down |
| GRIN1 | -1.722981151 | 2.730073651 | 1.45E-19 | 1.03E-18 | Down |
| DCST2 | -1.723385972 | 2.523104318 | 5.92E-26 | 6.64E-25 | Down |
| HOXB13 | -1.723551286 | 1.669365359 | 1.26E-08 | 3.81E-08 | Down |
| IGF2BP3 | -1.724490353 | 4.976088676 | 5.21E-14 | 2.49E-13 | Down |
| SLCO5A1 | -1.725634354 | 2.743537539 | 3.35E-25 | 3.54E-24 | Down |
| GOLM1 | -1.727638238 | 8.432090804 | 1.02E-69 | 2.47E-67 | Down |
| CYP27C1 | -1.728925209 | 3.18010511 | 2.74E-16 | 1.55E-15 | Down |
| SYNGR3 | -1.728963136 | 3.637701114 | 3.56E-28 | 4.61E-27 | Down |
| KRTAP4-1 | -1.732180758 | 1.590771204 | 1.08E-13 | 5.03E-13 | Down |
| NUSAP1 | -1.733735325 | 6.016329746 | 2.53E-53 | 1.66E-51 | Down |
| CDH17 | -1.733854489 | 2.827303374 | 3.97E-09 | 1.26E-08 | Down |
| OIP5 | -1.739831654 | 3.668901819 | 9.58E-37 | 2.20E-35 | Down |
| PLEK2 | -1.740417133 | 5.49641485 | 5.00E-49 | 2.49E-47 | Down |
| KIF23 | -1.742218097 | 5.410065532 | 1.07E-40 | 3.18E-39 | Down |
| TK1 | -1.743073999 | 6.691566385 | 8.53E-47 | 3.75E-45 | Down |
| TMEM59L | -1.745577449 | 4.10431478 | 6.58E-10 | 2.23E-09 | Down |
| CENPE | -1.748564417 | 4.950641989 | 1.29E-39 | 3.61E-38 | Down |
| ZWINT | -1.750013845 | 6.053847439 | 4.30E-56 | 3.53E-54 | Down |
| PAH | -1.752511261 | 1.579519232 | 1.41E-09 | 4.64E-09 | Down |
| NETO1 | -1.755027383 | 1.561867269 | 1.04E-14 | 5.21E-14 | Down |
| CRABP1 | -1.755116777 | 1.7368369 | 6.31E-10 | 2.14E-09 | Down |
| LGR4 | -1.755930271 | 6.903777769 | 8.12E-55 | 5.95E-53 | Down |
| PRSS50 | -1.758194935 | 2.247529397 | 1.22E-15 | 6.57E-15 | Down |
| OR51E1 | -1.761488313 | 1.848945825 | 1.64E-21 | 1.34E-20 | Down |
| MAGEA6 | -1.763134145 | 1.674050278 | 5.10E-06 | 1.22E-05 | Down |

| | | | | | |
|---|---|---|---|---|---|
| MMP10 | -1.763310726 | 2.929828305 | 1.30E-10 | 4.67E-10 | Down |
| LOC148709 | -1.764888944 | 2.029497704 | 1.71E-27 | 2.12E-26 | Down |
| CD19 | -1.765419522 | 3.378856282 | 2.35E-15 | 1.24E-14 | Down |
| CELF5 | -1.765528433 | 2.141649986 | 1.97E-15 | 1.04E-14 | Down |
| FAM111B | -1.768714039 | 5.047165714 | 3.78E-46 | 1.60E-44 | Down |
| FUT9 | -1.771016027 | 1.602292173 | 3.09E-10 | 1.08E-09 | Down |
| CASC5 | -1.775645248 | 4.335054521 | 1.35E-37 | 3.27E-36 | Down |
| B3GNT4 | -1.77575309 | 2.772625963 | 1.34E-21 | 1.10E-20 | Down |
| C20orf70 | -1.777464019 | 1.878000886 | 1.93E-10 | 6.80E-10 | Down |
| DPEP1 | -1.780040539 | 2.986358414 | 2.64E-19 | 1.85E-18 | Down |
| FUT6 | -1.780826766 | 4.342907219 | 4.34E-20 | 3.21E-19 | Down |
| AK3L1 | -1.784495365 | 4.889195515 | 1.07E-25 | 1.18E-24 | Down |
| ESCO2 | -1.784588774 | 4.12010569 | 4.04E-45 | 1.61E-43 | Down |
| C21orf125 | -1.784601097 | 2.877985391 | 4.86E-16 | 2.69E-15 | Down |
| ADAM12 | -1.785355921 | 5.57703237 | 4.18E-25 | 4.37E-24 | Down |
| KIFC1 | -1.788081736 | 5.75944716 | 1.66E-45 | 6.76E-44 | Down |
| HOXA10 | -1.788688668 | 2.911586061 | 5.33E-11 | 1.98E-10 | Down |
| RHBG | -1.789435129 | 1.583240836 | 1.50E-20 | 1.15E-19 | Down |
| TRIP13 | -1.790657427 | 5.550962703 | 4.22E-37 | 9.90E-36 | Down |
| PODXL2 | -1.791848797 | 6.241888908 | 7.82E-21 | 6.11E-20 | Down |
| THBS2 | -1.792982374 | 7.725183914 | 1.92E-33 | 3.56E-32 | Down |
| GNG4 | -1.796610649 | 3.45802164 | 5.84E-12 | 2.36E-11 | Down |
| CNFN | -1.797946916 | 3.310452964 | 4.05E-32 | 6.90E-31 | Down |
| C15orf48 | -1.800592654 | 6.221957456 | 1.28E-22 | 1.12E-21 | Down |
| C17orf53 | -1.801738845 | 4.36166169 | 2.22E-48 | 1.05E-46 | Down |
| SPTBN2 | -1.806886279 | 6.63812741 | 4.61E-59 | 4.57E-57 | Down |
| BEAN | -1.807603053 | 4.454690926 | 2.63E-24 | 2.61E-23 | Down |
| EZH2 | -1.808877538 | 5.550975326 | 1.84E-64 | 3.03E-62 | Down |
| TDRD5 | -1.808978118 | 2.558205862 | 8.11E-11 | 2.96E-10 | Down |
| SLC7A10 | -1.813573229 | 1.97373431 | 7.66E-11 | 2.81E-10 | Down |
| KIF11 | -1.814630125 | 5.874872343 | 1.26E-54 | 9.07E-53 | Down |
| IBSP | -1.817796371 | 1.958306449 | 2.40E-13 | 1.09E-12 | Down |
| TRIM31 | -1.81950694 | 3.176311962 | 1.77E-13 | 8.09E-13 | Down |
| FAM177B | -1.82020848 | 2.295611021 | 1.84E-13 | 8.39E-13 | Down |
| KREMEN2 | -1.822713457 | 2.230941707 | 4.55E-25 | 4.74E-24 | Down |
| BRIP1 | -1.8232261 | 4.016307175 | 2.52E-44 | 9.38E-43 | Down |
| RNF183 | -1.826563433 | 2.310300314 | 8.44E-13 | 3.66E-12 | Down |
| ADAMTS14 | -1.826782002 | 4.440732639 | 4.45E-39 | 1.18E-37 | Down |
| SIX1 | -1.831924476 | 5.578559174 | 4.65E-23 | 4.21E-22 | Down |
| CCNO | -1.833014974 | 4.50106411 | 5.50E-25 | 5.69E-24 | Down |
| VGF | -1.833285812 | 1.935477307 | 1.04E-14 | 5.22E-14 | Down |
| FAM178B | -1.836303231 | 3.116975474 | 2.43E-20 | 1.84E-19 | Down |
| P2RY6 | -1.842878729 | 5.328284221 | 7.14E-35 | 1.45E-33 | Down |
| SPDEF | -1.843456236 | 5.97178701 | 5.41E-22 | 4.59E-21 | Down |
| BUB1 | -1.844828959 | 5.641485651 | 5.08E-43 | 1.75E-41 | Down |
| HPDL | -1.845073643 | 2.846229235 | 2.09E-26 | 2.42E-25 | Down |

| | | | | | |
|---|---|---|---|---|---|
| CNGA3 | -1.84673938 | 2.608311674 | 6.81E-11 | 2.50E-10 | Down |
| SCG5 | -1.848024915 | 4.468702553 | 1.25E-21 | 1.03E-20 | Down |
| PLAC1 | -1.848091661 | 1.546010386 | 2.96E-19 | 2.06E-18 | Down |
| CST4 | -1.849474144 | 1.657168206 | 1.25E-15 | 6.72E-15 | Down |
| EME1 | -1.850115736 | 3.940598593 | 8.31E-49 | 4.06E-47 | Down |
| GREB1L | -1.85029131 | 2.417070753 | 5.79E-17 | 3.40E-16 | Down |
| EFNA3 | -1.852266047 | 4.777655633 | 3.49E-60 | 3.91E-58 | Down |
| FAM155B | -1.853682109 | 3.275610832 | 8.24E-20 | 5.98E-19 | Down |
| PADI1 | -1.855663806 | 1.839966468 | 4.46E-11 | 1.66E-10 | Down |
| MMP3 | -1.860281007 | 2.056378916 | 4.73E-14 | 2.26E-13 | Down |
| HORMAD1 | -1.860953955 | 2.273651272 | 1.42E-08 | 4.27E-08 | Down |
| RHBDL2 | -1.863336958 | 4.856115521 | 1.07E-43 | 3.88E-42 | Down |
| BRDT | -1.864332006 | 1.744387221 | 3.18E-08 | 9.32E-08 | Down |
| ACY3 | -1.868659429 | 3.405198167 | 1.01E-24 | 1.03E-23 | Down |
| SPRR1B | -1.870947251 | 1.939599798 | 3.43E-09 | 1.09E-08 | Down |
| CCNA2 | -1.871262867 | 5.586401732 | 2.83E-48 | 1.33E-46 | Down |
| DMRTA2 | -1.872044733 | 1.921615701 | 2.23E-11 | 8.56E-11 | Down |
| APCDD1L | -1.872127971 | 2.268349997 | 6.16E-17 | 3.61E-16 | Down |
| CCNB1 | -1.875338818 | 6.297961723 | 2.36E-55 | 1.80E-53 | Down |
| ANKRD34B | -1.875715843 | 1.948059794 | 7.75E-12 | 3.10E-11 | Down |
| IL1RL2 | -1.878972809 | 2.97408406 | 1.47E-36 | 3.36E-35 | Down |
| HOXC11 | -1.884962754 | 1.704999175 | 8.44E-11 | 3.08E-10 | Down |
| C15orf42 | -1.888610506 | 4.16187578 | 2.16E-43 | 7.65E-42 | Down |
| GUCY1B2 | -1.890483319 | 2.915552454 | 4.23E-20 | 3.14E-19 | Down |
| PLUNC | -1.892776718 | 5.055896492 | 2.41E-05 | 5.39E-05 | Down |
| COL22A1 | -1.893834868 | 3.166064188 | 7.45E-20 | 5.43E-19 | Down |
| GPC2 | -1.905435565 | 2.840782815 | 1.12E-27 | 1.41E-26 | Down |
| HELLS | -1.909520193 | 4.447226479 | 3.54E-55 | 2.68E-53 | Down |
| SMC1B | -1.913166932 | 1.717269528 | 3.02E-14 | 1.47E-13 | Down |
| PPAP2C | -1.915131825 | 7.120244021 | 1.62E-55 | 1.25E-53 | Down |
| C1orf61 | -1.91877185 | 1.719199566 | 6.77E-14 | 3.20E-13 | Down |
| MUC5B | -1.921246904 | 7.470308705 | 2.06E-07 | 5.58E-07 | Down |
| MDK | -1.925964639 | 8.327680704 | 1.13E-49 | 5.90E-48 | Down |
| CDT1 | -1.933712914 | 5.354259733 | 1.95E-52 | 1.22E-50 | Down |
| NXPH4 | -1.93386022 | 3.301837294 | 5.85E-20 | 4.29E-19 | Down |
| NPW | -1.934427035 | 2.1691249 | 1.56E-13 | 7.17E-13 | Down |
| CDCA5 | -1.940219967 | 5.671371363 | 9.79E-50 | 5.14E-48 | Down |
| GINS1 | -1.94075965 | 5.261674206 | 7.47E-57 | 6.42E-55 | Down |
| GINS2 | -1.941921496 | 4.994753364 | 1.13E-53 | 7.49E-52 | Down |
| CR2 | -1.945390131 | 4.272979114 | 7.67E-16 | 4.18E-15 | Down |
| COCH | -1.953015182 | 4.229566021 | 9.51E-20 | 6.87E-19 | Down |
| C1orf170 | -1.954417728 | 3.615716945 | 5.95E-50 | 3.17E-48 | Down |
| COL1A1 | -1.956268976 | 10.34462342 | 5.65E-35 | 1.17E-33 | Down |
| STX1A | -1.960933523 | 5.233868396 | 9.84E-54 | 6.56E-52 | Down |
| CPNE7 | -1.962090416 | 3.979323385 | 6.38E-30 | 9.31E-29 | Down |
| CASKIN1 | -1.962884217 | 1.98507001 | 5.28E-24 | 5.11E-23 | Down |

| | | | | | |
|---|---|---|---|---|---|
| INHA | -1.963854732 | 2.47900723 | 4.66E-11 | 1.73E-10 | Down |
| CLSPN | -1.968864844 | 4.263748362 | 2.75E-38 | 6.99E-37 | Down |
| C6orf222 | -1.968900659 | 1.900638413 | 8.40E-16 | 4.57E-15 | Down |
| IGF2BP1 | -1.971153255 | 2.021646114 | 4.13E-09 | 1.30E-08 | Down |
| PGLYRP4 | -1.971560162 | 1.99540924 | 2.40E-17 | 1.46E-16 | Down |
| POLE2 | -1.972778819 | 3.95981905 | 1.73E-46 | 7.45E-45 | Down |
| ZIC2 | -1.973377767 | 1.82943028 | 2.57E-13 | 1.16E-12 | Down |
| SLC2A5 | -1.974390368 | 4.514655805 | 3.59E-34 | 7.00E-33 | Down |
| XRCC2 | -1.975099162 | 3.829065854 | 2.22E-53 | 1.46E-51 | Down |
| UMODL1 | -1.976988302 | 2.832013196 | 9.36E-13 | 4.04E-12 | Down |
| SYT7 | -1.978069252 | 6.483289903 | 1.95E-31 | 3.21E-30 | Down |
| SLCO1B3 | -1.981322301 | 1.890843942 | 1.61E-09 | 5.28E-09 | Down |
| DUSP13 | -1.981392977 | 1.74342954 | 2.11E-13 | 9.62E-13 | Down |
| MKI67 | -1.982513414 | 6.881909625 | 4.52E-48 | 2.12E-46 | Down |
| CARD14 | -1.986970089 | 4.346570837 | 7.88E-30 | 1.14E-28 | Down |
| SKA1 | -1.987851484 | 4.206053951 | 1.71E-44 | 6.43E-43 | Down |
| JSRP1 | -1.992563334 | 3.415635163 | 1.56E-20 | 1.19E-19 | Down |
| ITGA11 | -1.993186579 | 5.946814054 | 5.02E-31 | 7.94E-30 | Down |
| HOXC13 | -1.99570102 | 1.829803453 | 8.73E-12 | 3.48E-11 | Down |
| LOC285629 | -1.996071969 | 2.356156846 | 1.83E-19 | 1.30E-18 | Down |
| KIAA0101 | -2.006068106 | 5.342095532 | 5.60E-56 | 4.54E-54 | Down |
| KCNN4 | -2.006857052 | 6.263026874 | 1.27E-29 | 1.82E-28 | Down |
| F12 | -2.007376802 | 4.000225634 | 3.56E-40 | 1.02E-38 | Down |
| RDM1 | -2.00889929 | 1.840574183 | 5.01E-34 | 9.72E-33 | Down |
| ORC1L | -2.014553457 | 4.440010635 | 5.11E-51 | 2.94E-49 | Down |
| GPX2 | -2.018615777 | 4.895524069 | 1.07E-07 | 2.98E-07 | Down |
| SPC25 | -2.018781275 | 4.03724049 | 3.38E-50 | 1.84E-48 | Down |
| DUSP9 | -2.018875407 | 2.203221901 | 2.52E-16 | 1.43E-15 | Down |
| SPC24 | -2.020179773 | 2.467997294 | 4.06E-41 | 1.25E-39 | Down |
| HAVCR1 | -2.02155165 | 1.807714104 | 1.99E-13 | 9.09E-13 | Down |
| GPT2 | -2.025942966 | 6.704706532 | 8.69E-53 | 5.47E-51 | Down |
| AGMAT | -2.032980471 | 3.518093538 | 3.01E-50 | 1.67E-48 | Down |
| C11orf86 | -2.034800376 | 1.759973754 | 3.88E-15 | 2.01E-14 | Down |
| SLC22A18AS | -2.035476665 | 3.717419744 | 6.59E-35 | 1.35E-33 | Down |
| MAST1 | -2.035506598 | 2.820085785 | 2.14E-33 | 3.95E-32 | Down |
| UNC5CL | -2.047463825 | 5.249924344 | 2.17E-43 | 7.68E-42 | Down |
| BCL2L15 | -2.050701166 | 4.982831065 | 3.28E-28 | 4.27E-27 | Down |
| BAIAP2L2 | -2.052915488 | 3.298628291 | 2.78E-21 | 2.24E-20 | Down |
| MAP7D2 | -2.058092148 | 4.006632911 | 1.11E-19 | 8.01E-19 | Down |
| PPP2R2C | -2.058449436 | 3.272701 | 5.49E-11 | 2.03E-10 | Down |
| PRSS3 | -2.063780208 | 2.235637771 | 2.36E-13 | 1.07E-12 | Down |
| CDCA2 | -2.066788519 | 4.223742213 | 4.98E-50 | 2.67E-48 | Down |
| ADAMTS16 | -2.066819386 | 3.902052534 | 1.12E-25 | 1.23E-24 | Down |
| KLK6 | -2.068327185 | 2.004045633 | 2.55E-10 | 8.92E-10 | Down |
| ERCC6L | -2.069370917 | 4.109705732 | 3.50E-63 | 5.10E-61 | Down |
| CHRNA5 | -2.071248018 | 4.195040405 | 3.34E-33 | 6.10E-32 | Down |

| | | | | | |
|---|---|---|---|---|---|
| RHBDL1 | -2.074383793 | 3.911376668 | 2.46E-35 | 5.18E-34 | Down |
| CDCA8 | -2.077125514 | 5.549336243 | 1.79E-63 | 2.69E-61 | Down |
| FERMT1 | -2.07992592 | 5.854684353 | 2.14E-51 | 1.27E-49 | Down |
| MUC16 | -2.080871701 | 5.118914419 | 8.16E-09 | 2.51E-08 | Down |
| CDCA7 | -2.085778431 | 5.662451182 | 2.31E-43 | 8.16E-42 | Down |
| SLC2A1 | -2.086990398 | 7.554724928 | 1.04E-44 | 3.95E-43 | Down |
| IL31RA | -2.094080742 | 2.469455327 | 6.29E-17 | 3.68E-16 | Down |
| HMGB3 | -2.095122175 | 8.082228208 | 5.07E-48 | 2.36E-46 | Down |
| HMGA2 | -2.096617124 | 3.013509344 | 3.04E-10 | 1.06E-09 | Down |
| ECEL1 | -2.102663172 | 2.301088703 | 6.47E-16 | 3.54E-15 | Down |
| PROC | -2.103393832 | 3.008663303 | 9.86E-22 | 8.23E-21 | Down |
| COMP | -2.104230163 | 5.599877491 | 5.14E-18 | 3.28E-17 | Down |
| KIF15 | -2.10442948 | 4.445100849 | 4.05E-49 | 2.04E-47 | Down |
| TNFSF11 | -2.105091216 | 2.674793674 | 7.19E-24 | 6.87E-23 | Down |
| HOXC10 | -2.107897178 | 2.176722266 | 1.21E-09 | 4.00E-09 | Down |
| S100P | -2.11163892 | 6.56436211 | 1.24E-11 | 4.86E-11 | Down |
| CCDC150 | -2.112199969 | 2.859161818 | 1.30E-42 | 4.37E-41 | Down |
| SPAG4 | -2.112793793 | 4.935444109 | 8.53E-63 | 1.20E-60 | Down |
| CELSR3 | -2.116497439 | 5.160952434 | 2.96E-44 | 1.10E-42 | Down |
| TFR2 | -2.117276382 | 3.416245094 | 2.73E-49 | 1.39E-47 | Down |
| RHOV | -2.119633919 | 5.329476433 | 1.45E-27 | 1.81E-26 | Down |
| COL7A1 | -2.11990384 | 4.706550741 | 1.28E-20 | 9.83E-20 | Down |
| CCNE1 | -2.121028893 | 4.511823667 | 1.53E-48 | 7.35E-47 | Down |
| TMEM184A | -2.124229234 | 6.513699415 | 1.10E-63 | 1.70E-61 | Down |
| CKAP2L | -2.125104653 | 4.560105413 | 1.57E-49 | 8.16E-48 | Down |
| LOC399815 | -2.128748052 | 2.203007019 | 4.68E-46 | 1.96E-44 | Down |
| GPR87 | -2.130354482 | 3.532741789 | 1.10E-11 | 4.34E-11 | Down |
| DLL3 | -2.130923075 | 1.910103262 | 9.05E-14 | 4.23E-13 | Down |
| SRPK3 | -2.130934243 | 3.609280491 | 1.02E-24 | 1.04E-23 | Down |
| CDKN3 | -2.133871795 | 4.50488929 | 6.93E-45 | 2.70E-43 | Down |
| GJB6 | -2.137164765 | 2.394883355 | 5.25E-15 | 2.68E-14 | Down |
| RRM2 | -2.140187305 | 6.469685189 | 6.89E-54 | 4.69E-52 | Down |
| CENPI | -2.150558734 | 3.395275148 | 2.73E-40 | 7.92E-39 | Down |
| NCAPH | -2.150973305 | 5.121451133 | 5.77E-54 | 3.96E-52 | Down |
| KIF26B | -2.151927266 | 5.680347093 | 3.43E-57 | 3.03E-55 | Down |
| ORC6L | -2.153901708 | 4.530925203 | 1.24E-65 | 2.14E-63 | Down |
| CEP55 | -2.159195659 | 5.60165766 | 7.55E-56 | 6.01E-54 | Down |
| IL1F5 | -2.161366404 | 2.016882724 | 6.07E-15 | 3.09E-14 | Down |
| TBX15 | -2.161576764 | 4.318528616 | 5.07E-25 | 5.25E-24 | Down |
| NGEF | -2.162344933 | 4.379699438 | 1.96E-25 | 2.10E-24 | Down |
| IL22RA2 | -2.165040012 | 2.238992739 | 6.82E-24 | 6.53E-23 | Down |
| SPAG5 | -2.168792054 | 6.047109472 | 1.03E-65 | 1.81E-63 | Down |
| CBLC | -2.173625036 | 5.340287302 | 4.81E-45 | 1.90E-43 | Down |
| FUT2 | -2.173788279 | 5.66397035 | 8.83E-54 | 5.95E-52 | Down |
| RECQL4 | -2.175434857 | 5.574068943 | 7.84E-62 | 1.02E-59 | Down |
| HMMR | -2.18011568 | 5.16347788 | 5.24E-56 | 4.27E-54 | Down |

| | | | | | |
|---|---|---|---|---|---|
| POLQ | -2.181524766 | 4.141628869 | 4.23E-49 | 2.13E-47 | Down |
| CENPF | -2.190507207 | 6.451308798 | 1.47E-56 | 1.24E-54 | Down |
| CP | -2.194854654 | 8.287007351 | 1.25E-25 | 1.36E-24 | Down |
| FEZF1 | -2.195296331 | 2.106026605 | 4.08E-16 | 2.27E-15 | Down |
| PLXNB3 | -2.199619249 | 5.517113597 | 8.69E-26 | 9.65E-25 | Down |
| GLB1L3 | -2.205513636 | 2.806342928 | 8.55E-10 | 2.86E-09 | Down |
| LYPD1 | -2.20784223 | 4.754066494 | 2.01E-35 | 4.27E-34 | Down |
| FAM64A | -2.210254097 | 4.156037735 | 4.33E-46 | 1.82E-44 | Down |
| PKMYT1 | -2.211936879 | 5.004481042 | 1.19E-55 | 9.38E-54 | Down |
| GTSE1 | -2.215012935 | 4.841771155 | 9.06E-59 | 8.86E-57 | Down |
| GALNT14 | -2.222616395 | 5.040076071 | 9.04E-26 | 1.00E-24 | Down |
| DMBX1 | -2.223037879 | 1.951922686 | 5.63E-21 | 4.44E-20 | Down |
| NCAPG | -2.227142828 | 5.369764782 | 4.24E-57 | 3.72E-55 | Down |
| CCNB2 | -2.229881192 | 5.466527465 | 1.48E-60 | 1.68E-58 | Down |
| FOXM1 | -2.237715819 | 6.201636023 | 1.62E-53 | 1.07E-51 | Down |
| EPR1 | -2.240034905 | 5.459382069 | 1.91E-45 | 7.75E-44 | Down |
| IL1F7 | -2.24183911 | 2.118687601 | 6.47E-12 | 2.61E-11 | Down |
| DNAJC22 | -2.246655938 | 3.618628946 | 9.34E-32 | 1.56E-30 | Down |
| PSAT1 | -2.250161195 | 5.758532166 | 6.85E-48 | 3.17E-46 | Down |
| ESPL1 | -2.256052196 | 5.029163951 | 9.38E-50 | 4.94E-48 | Down |
| SKA3 | -2.257086083 | 4.403614905 | 2.02E-58 | 1.94E-56 | Down |
| DGCR5 | -2.261705802 | 4.149482159 | 2.94E-31 | 4.76E-30 | Down |
| IGFL2 | -2.273285701 | 2.39094451 | 5.57E-22 | 4.72E-21 | Down |
| MB | -2.282823118 | 4.261668458 | 1.72E-27 | 2.13E-26 | Down |
| ABP1 | -2.284632817 | 5.019347387 | 4.71E-16 | 2.61E-15 | Down |
| HOXB9 | -2.28688439 | 2.286458334 | 4.85E-11 | 1.80E-10 | Down |
| CXorf61 | -2.288826942 | 2.030188996 | 2.13E-12 | 8.92E-12 | Down |
| PPAPDC1A | -2.289741148 | 2.461671029 | 2.40E-23 | 2.21E-22 | Down |
| ZNF695 | -2.289959807 | 2.331066526 | 1.02E-25 | 1.12E-24 | Down |
| UBE2T | -2.293800227 | 5.442629353 | 9.61E-73 | 2.95E-70 | Down |
| PLK1 | -2.296746794 | 5.729785235 | 3.81E-64 | 6.01E-62 | Down |
| CDCA3 | -2.297733251 | 4.71494055 | 2.05E-61 | 2.55E-59 | Down |
| TMEM63C | -2.299353269 | 4.261438239 | 3.15E-21 | 2.53E-20 | Down |
| NDC80 | -2.3031415 | 4.830401941 | 4.04E-55 | 3.04E-53 | Down |
| WFDC3 | -2.304596014 | 3.854368903 | 1.91E-24 | 1.90E-23 | Down |
| CDC6 | -2.312581816 | 5.529077531 | 1.62E-61 | 2.06E-59 | Down |
| ALG1L | -2.318242529 | 4.2242087 | 1.00E-30 | 1.54E-29 | Down |
| SALL4 | -2.319893717 | 3.150264342 | 3.70E-43 | 1.28E-41 | Down |
| COL17A1 | -2.324579436 | 5.213457151 | 9.10E-16 | 4.94E-15 | Down |
| SGOL1 | -2.345278262 | 3.659429076 | 5.17E-56 | 4.23E-54 | Down |
| LY6D | -2.350671445 | 2.531136161 | 1.35E-13 | 6.24E-13 | Down |
| PBK | -2.363742753 | 4.609071573 | 9.10E-49 | 4.44E-47 | Down |
| EPHA10 | -2.364092648 | 4.714699572 | 2.06E-35 | 4.37E-34 | Down |
| DNAJC12 | -2.366582955 | 5.002640964 | 1.09E-25 | 1.19E-24 | Down |
| HNF4G | -2.369062899 | 4.27097214 | 3.29E-34 | 6.44E-33 | Down |
| BARX2 | -2.372841195 | 4.205618424 | 1.58E-25 | 1.71E-24 | Down |

| | | | | | |
|---|---|---|---|---|---|
| ADM2 | -2.387381611 | 5.391345993 | 7.06E-74 | 2.41E-71 | Down |
| KIF20A | -2.391106217 | 5.479956383 | 1.01E-71 | 2.92E-69 | Down |
| PCP4 | -2.391431047 | 3.226414597 | 6.11E-14 | 2.90E-13 | Down |
| KRT6A | -2.39492689 | 4.017047348 | 3.24E-10 | 1.13E-09 | Down |
| GAD1 | -2.398175973 | 2.880850153 | 1.05E-25 | 1.15E-24 | Down |
| TTK | -2.400073635 | 4.637559713 | 8.73E-52 | 5.33E-50 | Down |
| IGSF9 | -2.400691433 | 5.725018375 | 3.96E-56 | 3.27E-54 | Down |
| UHRF1 | -2.401085656 | 5.246188829 | 2.53E-69 | 5.90E-67 | Down |
| CENPA | -2.402054299 | 4.168594658 | 1.64E-50 | 9.24E-49 | Down |
| RAD54L | -2.406931977 | 4.422455688 | 4.57E-61 | 5.49E-59 | Down |
| TPX2 | -2.409652786 | 6.442514406 | 6.88E-58 | 6.36E-56 | Down |
| CABYR | -2.409828334 | 4.25356655 | 9.72E-28 | 1.23E-26 | Down |
| CGREF1 | -2.410487609 | 3.887410116 | 4.97E-30 | 7.33E-29 | Down |
| ASPM | -2.410492823 | 5.453081007 | 4.06E-60 | 4.49E-58 | Down |
| LGSN | -2.412644489 | 4.449709062 | 2.82E-15 | 1.48E-14 | Down |
| PLEKHN1 | -2.412751082 | 4.351258502 | 1.74E-61 | 2.20E-59 | Down |
| EPN3 | -2.416854868 | 5.472560281 | 8.22E-55 | 6.00E-53 | Down |
| KISS1R | -2.420734112 | 2.089517428 | 9.07E-27 | 1.07E-25 | Down |
| HS6ST2 | -2.43168624 | 6.083153122 | 2.96E-39 | 8.02E-38 | Down |
| RGS17 | -2.434702972 | 3.209155937 | 2.60E-33 | 4.78E-32 | Down |
| ONECUT2 | -2.441117563 | 2.938497139 | 2.36E-23 | 2.18E-22 | Down |
| E2F8 | -2.444130114 | 4.515114794 | 1.04E-71 | 2.97E-69 | Down |
| C9orf140 | -2.449042158 | 5.510708508 | 1.80E-72 | 5.42E-70 | Down |
| MCM10 | -2.44992112 | 4.479937317 | 2.97E-51 | 1.74E-49 | Down |
| FGF11 | -2.451205788 | 4.770322835 | 4.23E-54 | 2.93E-52 | Down |
| CEACAM5 | -2.457826901 | 7.98485659 | 1.30E-14 | 6.47E-14 | Down |
| CILP2 | -2.47057945 | 4.710208001 | 7.42E-41 | 2.23E-39 | Down |
| TCN1 | -2.474377435 | 3.002546338 | 1.74E-11 | 6.74E-11 | Down |
| RAB26 | -2.478223562 | 3.356099715 | 2.82E-48 | 1.33E-46 | Down |
| CNTD2 | -2.486912642 | 3.115181907 | 4.72E-30 | 6.96E-29 | Down |
| ANLN | -2.488502109 | 6.189234337 | 8.29E-60 | 8.82E-58 | Down |
| TFF1 | -2.499991699 | 2.45381171 | 1.42E-10 | 5.06E-10 | Down |
| DEPDC1 | -2.505179585 | 4.758350474 | 6.42E-56 | 5.13E-54 | Down |
| EXO1 | -2.506990021 | 4.550430962 | 1.11E-59 | 1.15E-57 | Down |
| BUB1B | -2.51678771 | 5.176475366 | 1.10E-68 | 2.46E-66 | Down |
| DEPDC1B | -2.520637293 | 4.407496351 | 1.46E-60 | 1.68E-58 | Down |
| YBX2 | -2.523564918 | 2.838929081 | 3.78E-20 | 2.81E-19 | Down |
| XDH | -2.526182452 | 5.008856622 | 3.39E-36 | 7.60E-35 | Down |
| MNX1 | -2.539712658 | 2.753401753 | 1.43E-33 | 2.68E-32 | Down |
| AURKB | -2.541790484 | 4.874240175 | 2.63E-59 | 2.65E-57 | Down |
| KIF14 | -2.544398705 | 4.593140005 | 6.45E-61 | 7.65E-59 | Down |
| KIF18B | -2.545145405 | 4.872967257 | 3.53E-58 | 3.34E-56 | Down |
| PYCR1 | -2.54963416 | 7.607459183 | 4.35E-110 | 8.94E-106 | Down |
| C2CD4A | -2.550232092 | 4.689684776 | 2.34E-35 | 4.95E-34 | Down |
| OTX1 | -2.559501152 | 3.607226371 | 2.57E-67 | 5.13E-65 | Down |
| NEK2 | -2.563659931 | 5.148807027 | 2.47E-64 | 3.97E-62 | Down |

| | | | | | |
|---|---|---|---|---|---|
| GPR115 | -2.567548665 | 3.317974271 | 3.28E-23 | 3.00E-22 | Down |
| MUC13 | -2.574336896 | 4.14579405 | 2.81E-11 | 1.07E-10 | Down |
| MEX3A | -2.58644671 | 5.724850915 | 1.09E-58 | 1.06E-56 | Down |
| AKR7A3 | -2.597385926 | 3.689398424 | 9.42E-22 | 7.88E-21 | Down |
| TOP2A | -2.603588505 | 7.224343128 | 7.71E-71 | 2.06E-68 | Down |
| GRHL3 | -2.612713658 | 3.136370819 | 1.97E-40 | 5.77E-39 | Down |
| C16orf59 | -2.615580817 | 4.321952156 | 7.90E-91 | 2.32E-87 | Down |
| HJURP | -2.615719052 | 5.127953634 | 1.49E-64 | 2.48E-62 | Down |
| TFAP2A | -2.619541242 | 4.838130696 | 5.94E-44 | 2.18E-42 | Down |
| IQGAP3 | -2.619865651 | 5.826262426 | 4.63E-76 | 1.79E-73 | Down |
| MND1 | -2.621360727 | 3.47752998 | 2.94E-77 | 1.47E-74 | Down |
| ETV4 | -2.63214237 | 6.339476096 | 7.22E-71 | 1.95E-68 | Down |
| MELK | -2.639423784 | 5.057195635 | 6.04E-60 | 6.52E-58 | Down |
| CXCL13 | -2.639546525 | 5.506618197 | 1.84E-29 | 2.61E-28 | Down |
| CTHRC1 | -2.640010084 | 6.656871574 | 1.28E-62 | 1.76E-60 | Down |
| KIF2C | -2.649254816 | 5.521724836 | 1.16E-67 | 2.42E-65 | Down |
| NEIL3 | -2.65293088 | 3.493331976 | 1.60E-46 | 6.88E-45 | Down |
| CDC20 | -2.668653403 | 5.916760171 | 2.11E-69 | 4.99E-67 | Down |
| TROAP | -2.681639119 | 4.87087224 | 1.10E-59 | 1.15E-57 | Down |
| UBE2C | -2.711612731 | 5.842669247 | 1.68E-55 | 1.30E-53 | Down |
| NMU | -2.71185887 | 2.925141508 | 7.84E-24 | 7.49E-23 | Down |
| BIRC5 | -2.714403869 | 5.266369524 | 3.24E-61 | 3.96E-59 | Down |
| GJB2 | -2.718750401 | 5.653837637 | 6.93E-45 | 2.70E-43 | Down |
| TUBB3 | -2.721046089 | 7.01185776 | 2.40E-61 | 2.97E-59 | Down |
| FRMD5 | -2.729668317 | 3.50338214 | 2.58E-51 | 1.52E-49 | Down |
| BARX1 | -2.738797148 | 2.679135106 | 1.83E-13 | 8.35E-13 | Down |
| DLGAP5 | -2.74404439 | 5.121749104 | 7.99E-67 | 1.53E-64 | Down |
| CDC45 | -2.74992894 | 4.628621345 | 8.03E-66 | 1.42E-63 | Down |
| MYBL2 | -2.762183674 | 6.19062377 | 5.75E-55 | 4.28E-53 | Down |
| NUF2 | -2.766524828 | 4.852452191 | 7.93E-68 | 1.70E-65 | Down |
| HHIPL2 | -2.767398411 | 2.897113727 | 1.22E-18 | 8.12E-18 | Down |
| FGB | -2.80538272 | 3.385251381 | 2.64E-09 | 8.48E-09 | Down |
| LOC100131726 | -2.809906098 | 2.441043385 | 1.45E-46 | 6.33E-45 | Down |
| GCNT3 | -2.843299071 | 5.478449815 | 1.09E-32 | 1.93E-31 | Down |
| KIF4A | -2.847595724 | 5.314585562 | 5.86E-68 | 1.27E-65 | Down |
| ENTPD8 | -2.849410011 | 3.327787125 | 1.50E-37 | 3.63E-36 | Down |
| TRPM8 | -2.875797209 | 2.758497099 | 1.14E-23 | 1.08E-22 | Down |
| B4GALNT4 | -2.897010259 | 3.855115417 | 5.07E-26 | 5.72E-25 | Down |
| CDC25C | -2.898891592 | 3.758226045 | 8.96E-72 | 2.63E-69 | Down |
| AKR1B10 | -2.901656967 | 3.42864685 | 3.38E-12 | 1.39E-11 | Down |
| KRT16 | -2.916949007 | 3.303133811 | 3.60E-21 | 2.88E-20 | Down |
| ITPKA | -2.927843285 | 4.121856431 | 8.73E-32 | 1.46E-30 | Down |
| FER1L4 | -2.94743442 | 4.704068182 | 4.69E-45 | 1.86E-43 | Down |
| RASAL1 | -2.959266063 | 4.357594556 | 1.34E-56 | 1.13E-54 | Down |
| MMP12 | -2.974705414 | 5.157811794 | 6.57E-32 | 1.11E-30 | Down |
| B3GNT3 | -2.974871198 | 6.137140936 | 4.20E-63 | 6.07E-61 | Down |

| | | | | | |
|---|---|---|---|---|---|
| GREM1 | -2.987634456 | 6.005879029 | 6.03E-39 | 1.58E-37 | Down |
| PAEP | -2.993611366 | 3.602576488 | 5.19E-13 | 2.29E-12 | Down |
| MYEOV | -2.994208113 | 3.899594736 | 1.09E-24 | 1.10E-23 | Down |
| ZYG11A | -3.005269787 | 2.803644749 | 2.15E-35 | 4.56E-34 | Down |
| VIL1 | -3.026545622 | 2.904646708 | 6.11E-15 | 3.11E-14 | Down |
| XAGE1D | -3.038586133 | 5.785284216 | 4.17E-13 | 1.85E-12 | Down |
| STRA6 | -3.040757701 | 4.384992655 | 1.08E-36 | 2.49E-35 | Down |
| EEF1A2 | -3.067652711 | 4.87963894 | 7.13E-19 | 4.85E-18 | Down |
| FGL1 | -3.112236167 | 3.639711642 | 2.33E-16 | 1.32E-15 | Down |
| B3GNT6 | -3.205374131 | 3.793442973 | 3.52E-25 | 3.71E-24 | Down |
| PITX1 | -3.209124846 | 4.427744894 | 5.82E-35 | 1.20E-33 | Down |
| HTR3A | -3.21770986 | 3.304830343 | 5.86E-32 | 9.92E-31 | Down |
| CRABP2 | -3.233362637 | 6.965002276 | 4.81E-41 | 1.47E-39 | Down |
| 729884 | -3.242338298 | 3.383742985 | 3.46E-24 | 3.40E-23 | Down |
| PITX2 | -3.243498758 | 2.891941112 | 1.70E-25 | 1.84E-24 | Down |
| COL10A1 | -3.26769159 | 6.158526362 | 1.13E-62 | 1.58E-60 | Down |
| SPP1 | -3.297516687 | 8.297337444 | 2.75E-51 | 1.61E-49 | Down |
| MMP1 | -3.326719556 | 5.926495777 | 1.86E-35 | 3.97E-34 | Down |
| ATP10B | -3.353608223 | 4.238136977 | 6.39E-45 | 2.50E-43 | Down |
| TMPRSS4 | -3.367464885 | 6.733025788 | 4.32E-49 | 2.17E-47 | Down |
| PRAME | -3.390047121 | 3.351174535 | 1.41E-18 | 9.36E-18 | Down |
| SPINK1 | -3.416930566 | 5.239855845 | 1.38E-19 | 9.85E-19 | Down |
| MMP13 | -3.565235681 | 4.219062484 | 5.64E-31 | 8.89E-30 | Down |
| SYT12 | -3.566268979 | 4.840156825 | 1.89E-42 | 6.30E-41 | Down |
| PTPRH | -3.603734334 | 4.480195882 | 5.46E-46 | 2.26E-44 | Down |
| MMP11 | -3.617949569 | 6.667394795 | 1.82E-63 | 2.73E-61 | Down |
| PPP1R14D | -3.665699079 | 3.663107375 | 1.22E-34 | 2.46E-33 | Down |
| CA9 | -3.66898258 | 4.041996856 | 1.09E-29 | 1.56E-28 | Down |
| LOC84740 | -3.69592949 | 5.876941277 | 5.99E-33 | 1.07E-31 | Down |
| CYP24A1 | -3.783972773 | 5.374661118 | 6.24E-35 | 1.28E-33 | Down |
| ABCA12 | -3.858281793 | 3.97638025 | 5.54E-43 | 1.90E-41 | Down |
| COL11A1 | -4.273838013 | 5.173444892 | 3.39E-40 | 9.75E-39 | Down |
| CST1 | -4.28927857 | 4.348862315 | 4.81E-41 | 1.47E-39 | Down |
| FAM83A | -4.41531909 | 6.546931671 | 3.23E-77 | 1.58E-74 | Down |

Table Supplementary 2: Differentially expressed circRNAs of LUAD

| circRNA-id | logFC | AveExpr | P.Value | adj.P.Val |
|---|---|---|---|---|
| HSA_CIRCpedia_120801 | 3.89777528 | 1.054349378 | 2.62E-90 | 6.72E-87 |
| HSA_CIRCpedia_349537 | 1.593965647 | 7.259432742 | 8.01E-90 | 1.83E-86 |
| HSA_CIRCpedia_346334 | 1.593965647 | 7.259432742 | 8.01E-90 | 1.83E-86 |
| HSA_CIRCpedia_345029 | 1.593965647 | 7.259432742 | 8.01E-90 | 1.83E-86 |
| HSA_CIRCpedia_41504 | 1.872772138 | 9.026133967 | 3.51E-88 | 6.54E-85 |
| HSA_CIRCpedia_343770 | 3.623519796 | 1.168509601 | 1.81E-87 | 2.86E-84 |
| HSA_CIRCpedia_127614 | 3.830945481 | 1.248976677 | 7.02E-84 | 7.59E-81 |
| HSA_CIRCpedia_357665 | 1.713356146 | 6.584240781 | 5.05E-83 | 4.51E-80 |
| HSA_CIRCpedia_48036 | 1.713356146 | 6.584240781 | 5.05E-83 | 4.51E-80 |
| HSA_CIRCpedia_357777 | 2.478572863 | 5.337073846 | 8.91E-81 | 6.10E-78 |
| HSA_CIRCpedia_47867 | 1.908440183 | 5.682382107 | 1.75E-80 | 1.12E-77 |
| HSA_CIRCpedia_62215 | 1.783471999 | 6.980695042 | 1.47E-78 | 7.94E-76 |
| HSA_CIRCpedia_62213 | 1.783471999 | 6.980695042 | 1.47E-78 | 7.94E-76 |
| HSA_CIRCpedia_357547 | -1.136740669 | 5.290272774 | 8.60E-74 | 2.85E-71 |
| HSA_CIRCpedia_41500 | 1.398837057 | 5.770301235 | 6.59E-73 | 2.08E-70 |
| HSA_CIRCpedia_41491 | 1.398837057 | 5.770301235 | 6.59E-73 | 2.08E-70 |
| HSA_CIRCpedia_163938 | 2.035721369 | 6.414002814 | 2.85E-71 | 7.92E-69 |
| HSA_CIRCpedia_146340 | 2.035721369 | 6.414002814 | 2.85E-71 | 7.92E-69 |
| HSA_CIRCpedia_122777 | 2.035721369 | 6.414002814 | 2.85E-71 | 7.92E-69 |
| HSA_CIRCpedia_122778 | 2.035721369 | 6.414002814 | 2.85E-71 | 7.92E-69 |
| HSA_CIRCpedia_78606 | -2.603588505 | 7.224343128 | 7.71E-71 | 2.06E-68 |
| HSA_CIRCpedia_20602 | -2.603588505 | 7.224343128 | 7.71E-71 | 2.06E-68 |
| HSA_CIRCpedia_64606 | -1.727638238 | 8.432090804 | 1.02E-69 | 2.47E-67 |
| HSA_CIRCpedia_64607 | -1.727638238 | 8.432090804 | 1.02E-69 | 2.47E-67 |
| HSA_CIRCpedia_64605 | -1.727638238 | 8.432090804 | 1.02E-69 | 2.47E-67 |
| HSA_CIRCpedia_357417 | -2.51678771 | 5.176475366 | 1.10E-68 | 2.46E-66 |
| HSA_CIRCpedia_54861 | -1.056769939 | 7.373495523 | 2.17E-68 | 4.80E-66 |
| HSA_CIRCpedia_94677 | -2.847595724 | 5.314585562 | 5.86E-68 | 1.27E-65 |
| HSA_CIRCpedia_46985 | 1.949704016 | 7.773743094 | 8.57E-67 | 1.63E-64 |
| HSA_CIRCpedia_4201 | 1.411188291 | 7.598418217 | 2.66E-66 | 4.87E-64 |
| HSA_CIRCpedia_357806 | 1.671164357 | 6.05178927 | 1.12E-65 | 1.94E-63 |
| HSA_CIRCpedia_57174 | -1.808877538 | 5.550975326 | 1.84E-64 | 3.03E-62 |
| HSA_CIRCpedia_65031 | 1.927749479 | 4.236361576 | 1.23E-63 | 1.88E-61 |
| HSA_CIRCpedia_65032 | 1.927749479 | 4.236361576 | 1.23E-63 | 1.88E-61 |
| HSA_CIRCpedia_146143 | 3.25169054 | 4.107114786 | 1.23E-62 | 1.71E-60 |
| HSA_CIRCpedia_41669 | 1.236687956 | 9.013490849 | 2.56E-62 | 3.43E-60 |
| HSA_CIRCpedia_154212 | 2.626023483 | 0.795064324 | 5.52E-62 | 7.27E-60 |
| HSA_CIRCpedia_137869 | 2.809327768 | 1.917062339 | 1.23E-61 | 1.58E-59 |
| HSA_CIRCpedia_10972 | 1.627059373 | 4.47710626 | 2.53E-61 | 3.11E-59 |
| HSA_CIRCpedia_10971 | 1.627059373 | 4.47710626 | 2.53E-61 | 3.11E-59 |
| HSA_CIRCpedia_10970 | 1.627059373 | 4.47710626 | 2.53E-61 | 3.11E-59 |
| HSA_CIRCpedia_1138 | 2.894589651 | 1.369688534 | 4.06E-60 | 4.49E-58 |
| HSA_CIRCpedia_53848 | 1.348934123 | 6.537455778 | 5.23E-60 | 5.71E-58 |

| | | | | |
|---|---|---|---|---|
| HSA_CIRCpedia_357086 | 2.520863891 | 2.979638469 | 1.10E-59 | 1.15E-57 |
| HSA_CIRCpedia_29318 | 2.520863891 | 2.979638469 | 1.10E-59 | 1.15E-57 |
| HSA_CIRCpedia_140211 | 2.520863891 | 2.979638469 | 1.10E-59 | 1.15E-57 |
| HSA_CIRCpedia_125263 | 2.520863891 | 2.979638469 | 1.10E-59 | 1.15E-57 |
| HSA_CIRCpedia_347355 | 2.520863891 | 2.979638469 | 1.10E-59 | 1.15E-57 |
| HSA_CIRCpedia_10339 | -2.257086083 | 4.403614905 | 2.02E-58 | 1.94E-56 |
| HSA_CIRCpedia_357053 | 2.102978 | 5.610294938 | 4.75E-58 | 4.43E-56 |
| HSA_CIRCpedia_20287 | -1.131996548 | 6.657134946 | 5.56E-58 | 5.17E-56 |
| HSA_CIRCpedia_20284 | -1.131996548 | 6.657134946 | 5.56E-58 | 5.17E-56 |
| HSA_CIRCpedia_30983 | -2.151927266 | 5.680347093 | 3.43E-57 | 3.03E-55 |
| HSA_CIRCpedia_124237 | 3.96993595 | 2.159717104 | 3.90E-57 | 3.44E-55 |
| HSA_CIRCpedia_124240 | 3.96993595 | 2.159717104 | 3.90E-57 | 3.44E-55 |
| HSA_CIRCpedia_47981 | -2.227142828 | 5.369764782 | 4.24E-57 | 3.72E-55 |
| HSA_CIRCpedia_357379 | 2.575421769 | 5.935923114 | 5.06E-57 | 4.36E-55 |
| HSA_CIRCpedia_163048 | 2.575421769 | 5.935923114 | 5.06E-57 | 4.36E-55 |
| HSA_CIRCpedia_121631 | 2.575421769 | 5.935923114 | 5.06E-57 | 4.36E-55 |
| HSA_CIRCpedia_357380 | 2.575421769 | 5.935923114 | 5.06E-57 | 4.36E-55 |
| HSA_CIRCpedia_57179 | -1.158155032 | 9.004354108 | 3.03E-56 | 2.53E-54 |
| HSA_CIRCpedia_28665 | -1.259308227 | 5.080988639 | 1.10E-55 | 8.75E-54 |
| HSA_CIRCpedia_43057 | 1.679337818 | 6.584646421 | 1.26E-55 | 9.90E-54 |
| HSA_CIRCpedia_2877 | -1.909520193 | 4.447226479 | 3.54E-55 | 2.68E-53 |
| HSA_CIRCpedia_30276 | -1.397814391 | 4.538060537 | 6.28E-55 | 4.62E-53 |
| HSA_CIRCpedia_30268 | -1.397814391 | 4.538060537 | 6.28E-55 | 4.62E-53 |
| HSA_CIRCpedia_30258 | -1.397814391 | 4.538060537 | 6.28E-55 | 4.62E-53 |
| HSA_CIRCpedia_30257 | -1.397814391 | 4.538060537 | 6.28E-55 | 4.62E-53 |
| HSA_CIRCpedia_30292 | -1.397814391 | 4.538060537 | 6.28E-55 | 4.62E-53 |
| HSA_CIRCpedia_4031 | -1.755930271 | 6.903777769 | 8.12E-55 | 5.95E-53 |
| HSA_CIRCpedia_2794 | -1.814630125 | 5.874872343 | 1.26E-54 | 9.07E-53 |
| HSA_CIRCpedia_357588 | 1.636668692 | 8.52341268 | 1.85E-54 | 1.32E-52 |
| HSA_CIRCpedia_93540 | 1.636668692 | 8.52341268 | 1.85E-54 | 1.32E-52 |
| HSA_CIRCpedia_357587 | 1.636668692 | 8.52341268 | 1.85E-54 | 1.32E-52 |
| HSA_CIRCpedia_92398 | -2.150973305 | 5.121451133 | 5.77E-54 | 3.96E-52 |
| HSA_CIRCpedia_119887 | 3.072365691 | 1.355784377 | 7.96E-54 | 5.37E-52 |
| HSA_CIRCpedia_1823 | 3.072365691 | 1.355784377 | 7.96E-54 | 5.37E-52 |
| HSA_CIRCpedia_60997 | 1.411854447 | 7.626417435 | 1.16E-53 | 7.68E-52 |
| HSA_CIRCpedia_60998 | 1.411854447 | 7.626417435 | 1.16E-53 | 7.68E-52 |
| HSA_CIRCpedia_61002 | 1.411854447 | 7.626417435 | 1.16E-53 | 7.68E-52 |
| HSA_CIRCpedia_89107 | 1.411854447 | 7.626417435 | 1.16E-53 | 7.68E-52 |
| HSA_CIRCpedia_114480 | -1.733735325 | 6.016329746 | 2.53E-53 | 1.66E-51 |
| HSA_CIRCpedia_14220 | -1.733735325 | 6.016329746 | 2.53E-53 | 1.66E-51 |
| HSA_CIRCpedia_7350 | -1.519316196 | 5.923803382 | 2.64E-52 | 1.64E-50 |
| HSA_CIRCpedia_7378 | -1.519316196 | 5.923803382 | 2.64E-52 | 1.64E-50 |
| HSA_CIRCpedia_7328 | -1.519316196 | 5.923803382 | 2.64E-52 | 1.64E-50 |
| HSA_CIRCpedia_31942 | 1.444086441 | 6.220864496 | 2.21E-51 | 1.31E-49 |
| HSA_CIRCpedia_14884 | 1.753355849 | 6.567662683 | 3.18E-51 | 1.85E-49 |
| HSA_CIRCpedia_14883 | 1.753355849 | 6.567662683 | 3.18E-51 | 1.85E-49 |

| | | | | |
|---|---|---|---|---|
| HSA_CIRCpedia_14881 | 1.753355849 | 6.567662683 | 3.18E-51 | 1.85E-49 |
| HSA_CIRCpedia_14880 | 1.753355849 | 6.567662683 | 3.18E-51 | 1.85E-49 |
| HSA_CIRCpedia_152413 | 1.753355849 | 6.567662683 | 3.18E-51 | 1.85E-49 |
| HSA_CIRCpedia_53759 | 1.196377561 | 6.599361688 | 1.00E-50 | 5.74E-49 |
| HSA_CIRCpedia_53752 | 1.196377561 | 6.599361688 | 1.00E-50 | 5.74E-49 |
| HSA_CIRCpedia_53753 | 1.196377561 | 6.599361688 | 1.00E-50 | 5.74E-49 |
| HSA_CIRCpedia_53755 | 1.196377561 | 6.599361688 | 1.00E-50 | 5.74E-49 |
| HSA_CIRCpedia_357485 | 1.626216846 | 7.525172762 | 1.02E-50 | 5.82E-49 |
| HSA_CIRCpedia_357486 | 1.626216846 | 7.525172762 | 1.02E-50 | 5.82E-49 |
| HSA_CIRCpedia_56320 | 1.270542292 | 6.297848902 | 3.05E-50 | 1.68E-48 |
| HSA_CIRCpedia_357399 | 1.270542292 | 6.297848902 | 3.05E-50 | 1.68E-48 |
| HSA_CIRCpedia_56337 | 1.270542292 | 6.297848902 | 3.05E-50 | 1.68E-48 |
| HSA_CIRCpedia_56325 | 1.270542292 | 6.297848902 | 3.05E-50 | 1.68E-48 |
| HSA_CIRCpedia_56335 | 1.270542292 | 6.297848902 | 3.05E-50 | 1.68E-48 |
| HSA_CIRCpedia_56333 | 1.270542292 | 6.297848902 | 3.05E-50 | 1.68E-48 |
| HSA_CIRCpedia_121754 | 1.270542292 | 6.297848902 | 3.05E-50 | 1.68E-48 |
| HSA_CIRCpedia_98499 | 1.270542292 | 6.297848902 | 3.05E-50 | 1.68E-48 |
| HSA_CIRCpedia_357398 | 1.270542292 | 6.297848902 | 3.05E-50 | 1.68E-48 |
| HSA_CIRCpedia_145235 | 1.270542292 | 6.297848902 | 3.05E-50 | 1.68E-48 |
| HSA_CIRCpedia_357907 | 2.736092745 | 3.373133255 | 3.36E-50 | 1.83E-48 |
| HSA_CIRCpedia_46157 | -1.417649212 | 4.253022728 | 3.41E-50 | 1.85E-48 |
| HSA_CIRCpedia_357711 | -1.510485505 | 5.460933723 | 3.90E-50 | 2.10E-48 |
| HSA_CIRCpedia_16056 | -1.376813273 | 6.212669083 | 4.55E-50 | 2.45E-48 |
| HSA_CIRCpedia_346796 | 2.353067235 | 7.446681235 | 9.17E-50 | 4.84E-48 |
| HSA_CIRCpedia_357866 | 2.353067235 | 7.446681235 | 9.17E-50 | 4.84E-48 |
| HSA_CIRCpedia_38657 | 1.568407455 | 4.364607658 | 2.39E-49 | 1.23E-47 |
| HSA_CIRCpedia_150979 | 1.411732495 | 5.891551014 | 3.32E-49 | 1.68E-47 |
| HSA_CIRCpedia_357645 | 1.411732495 | 5.891551014 | 3.32E-49 | 1.68E-47 |
| HSA_CIRCpedia_103497 | 1.411732495 | 5.891551014 | 3.32E-49 | 1.68E-47 |
| HSA_CIRCpedia_47279 | 1.411732495 | 5.891551014 | 3.32E-49 | 1.68E-47 |
| HSA_CIRCpedia_47278 | 1.411732495 | 5.891551014 | 3.32E-49 | 1.68E-47 |
| HSA_CIRCpedia_45728 | -2.10442948 | 4.445100849 | 4.05E-49 | 2.04E-47 |
| HSA_CIRCpedia_45722 | -2.10442948 | 4.445100849 | 4.05E-49 | 2.04E-47 |
| HSA_CIRCpedia_357810 | -2.10442948 | 4.445100849 | 4.05E-49 | 2.04E-47 |
| HSA_CIRCpedia_43135 | -2.181524766 | 4.141628869 | 4.23E-49 | 2.13E-47 |
| HSA_CIRCpedia_119743 | 2.369593957 | 1.448342955 | 5.47E-49 | 2.71E-47 |
| HSA_CIRCpedia_35715 | 1.301961716 | 6.092157503 | 8.23E-49 | 4.03E-47 |
| HSA_CIRCpedia_35710 | 1.301961716 | 6.092157503 | 8.23E-49 | 4.03E-47 |
| HSA_CIRCpedia_35721 | 1.301961716 | 6.092157503 | 8.23E-49 | 4.03E-47 |
| HSA_CIRCpedia_2076 | -1.571356178 | 4.667128344 | 9.23E-49 | 4.49E-47 |
| HSA_CIRCpedia_120476 | 2.953048228 | 3.403625217 | 1.13E-48 | 5.42E-47 |
| HSA_CIRCpedia_18177 | 2.953048228 | 3.403625217 | 1.13E-48 | 5.42E-47 |
| HSA_CIRCpedia_47530 | 1.068748369 | 6.170163002 | 1.63E-48 | 7.78E-47 |
| HSA_CIRCpedia_120291 | 2.092335507 | 2.822550456 | 1.84E-48 | 8.77E-47 |
| HSA_CIRCpedia_17111 | -1.341128846 | 5.683207035 | 5.41E-48 | 2.51E-46 |
| HSA_CIRCpedia_116255 | 2.208973026 | 3.910777265 | 6.97E-48 | 3.22E-46 |

| | | | | |
|---|---|---|---|---|
| HSA_CIRCpedia_11041 | 2.208973026 | 3.910777265 | 6.97E-48 | 3.22E-46 |
| HSA_CIRCpedia_11040 | 2.208973026 | 3.910777265 | 6.97E-48 | 3.22E-46 |
| HSA_CIRCpedia_146387 | 1.389442319 | 4.433820337 | 3.97E-47 | 1.78E-45 |
| HSA_CIRCpedia_12393 | 1.040884094 | 6.850004968 | 4.16E-47 | 1.86E-45 |
| HSA_CIRCpedia_12392 | 1.040884094 | 6.850004968 | 4.16E-47 | 1.86E-45 |
| HSA_CIRCpedia_12395 | 1.040884094 | 6.850004968 | 4.16E-47 | 1.86E-45 |
| HSA_CIRCpedia_12391 | 1.040884094 | 6.850004968 | 4.16E-47 | 1.86E-45 |
| HSA_CIRCpedia_115462 | 1.040884094 | 6.850004968 | 4.16E-47 | 1.86E-45 |
| HSA_CIRCpedia_47993 | -2.65293088 | 3.493331976 | 1.60E-46 | 6.88E-45 |
| HSA_CIRCpedia_47994 | -2.65293088 | 3.493331976 | 1.60E-46 | 6.88E-45 |
| HSA_CIRCpedia_12208 | -1.972778819 | 3.95981905 | 1.73E-46 | 7.45E-45 |
| HSA_CIRCpedia_12211 | -1.972778819 | 3.95981905 | 1.73E-46 | 7.45E-45 |
| HSA_CIRCpedia_3696 | 1.282291676 | 6.047064653 | 1.87E-46 | 8.03E-45 |
| HSA_CIRCpedia_9584 | 3.241400447 | 1.943838197 | 4.63E-46 | 1.94E-44 |
| HSA_CIRCpedia_9582 | 3.241400447 | 1.943838197 | 4.63E-46 | 1.94E-44 |
| HSA_CIRCpedia_53562 | 1.989007652 | 3.951580621 | 4.71E-45 | 1.87E-43 |
| HSA_CIRCpedia_99098 | 1.989007652 | 3.951580621 | 4.71E-45 | 1.87E-43 |
| HSA_CIRCpedia_147565 | 2.613590585 | 2.672340247 | 6.09E-45 | 2.38E-43 |
| HSA_CIRCpedia_137492 | 2.613590585 | 2.672340247 | 6.09E-45 | 2.38E-43 |
| HSA_CIRCpedia_6132 | 1.402772163 | 6.725619805 | 8.73E-45 | 3.36E-43 |
| HSA_CIRCpedia_6143 | 1.402772163 | 6.725619805 | 8.73E-45 | 3.36E-43 |
| HSA_CIRCpedia_6130 | 1.402772163 | 6.725619805 | 8.73E-45 | 3.36E-43 |
| HSA_CIRCpedia_357148 | 2.354783843 | 6.367859065 | 9.91E-45 | 3.78E-43 |
| HSA_CIRCpedia_3091 | -1.258873034 | 6.562767771 | 1.17E-44 | 4.43E-43 |
| HSA_CIRCpedia_54562 | 1.331593816 | 6.469036249 | 2.07E-44 | 7.76E-43 |
| HSA_CIRCpedia_55208 | -1.280101228 | 8.08968892 | 6.86E-44 | 2.51E-42 |
| HSA_CIRCpedia_68625 | -1.024693865 | 5.921883949 | 7.95E-44 | 2.89E-42 |
| HSA_CIRCpedia_101130 | -1.024693865 | 5.921883949 | 7.95E-44 | 2.89E-42 |
| HSA_CIRCpedia_9346 | 1.473952028 | 6.093601578 | 2.80E-43 | 9.78E-42 |
| HSA_CIRCpedia_357757 | -1.044183432 | 5.461903631 | 3.28E-43 | 1.14E-41 |
| HSA_CIRCpedia_37463 | -1.844828959 | 5.641485651 | 5.08E-43 | 1.75E-41 |
| HSA_CIRCpedia_131014 | 2.087856171 | 4.555428571 | 5.45E-43 | 1.87E-41 |
| HSA_CIRCpedia_162455 | 2.087856171 | 4.555428571 | 5.45E-43 | 1.87E-41 |
| HSA_CIRCpedia_53709 | 1.385705787 | 6.111396688 | 6.66E-43 | 2.27E-41 |
| HSA_CIRCpedia_357666 | 1.466933234 | 4.166717327 | 1.88E-42 | 6.26E-41 |
| HSA_CIRCpedia_162165 | -1.575708703 | 5.017032222 | 3.75E-42 | 1.23E-40 |
| HSA_CIRCpedia_60040 | 1.846044416 | 7.214570461 | 8.48E-42 | 2.72E-40 |
| HSA_CIRCpedia_60043 | 1.846044416 | 7.214570461 | 8.48E-42 | 2.72E-40 |
| HSA_CIRCpedia_69363 | 1.846044416 | 7.214570461 | 8.48E-42 | 2.72E-40 |
| HSA_CIRCpedia_60046 | 1.846044416 | 7.214570461 | 8.48E-42 | 2.72E-40 |
| HSA_CIRCpedia_60041 | 1.846044416 | 7.214570461 | 8.48E-42 | 2.72E-40 |
| HSA_CIRCpedia_122537 | 1.960710942 | 3.185348735 | 1.78E-41 | 5.62E-40 |
| HSA_CIRCpedia_120620 | 1.120873583 | 7.25292549 | 1.84E-41 | 5.80E-40 |
| HSA_CIRCpedia_14606 | 1.120873583 | 7.25292549 | 1.84E-41 | 5.80E-40 |
| HSA_CIRCpedia_14605 | 1.120873583 | 7.25292549 | 1.84E-41 | 5.80E-40 |
| HSA_CIRCpedia_34792 | -1.644667102 | 5.733486127 | 1.88E-41 | 5.91E-40 |

| | | | | |
|---|---|---|---|---|
| HSA_CIRCpedia_1067 | 1.003345311 | 5.907218704 | 2.56E-41 | 8.05E-40 |
| HSA_CIRCpedia_75811 | 1.003345311 | 5.907218704 | 2.56E-41 | 8.05E-40 |
| HSA_CIRCpedia_1066 | 1.003345311 | 5.907218704 | 2.56E-41 | 8.05E-40 |
| HSA_CIRCpedia_4879 | 1.582077661 | 9.504363881 | 3.13E-41 | 9.76E-40 |
| HSA_CIRCpedia_51553 | 1.996362559 | 5.954792777 | 3.71E-41 | 1.15E-39 |
| HSA_CIRCpedia_51556 | 1.996362559 | 5.954792777 | 3.71E-41 | 1.15E-39 |
| HSA_CIRCpedia_134187 | -1.060823575 | 9.015236061 | 3.73E-41 | 1.15E-39 |
| HSA_CIRCpedia_122326 | -1.060823575 | 9.015236061 | 3.73E-41 | 1.15E-39 |
| HSA_CIRCpedia_151067 | 2.166074046 | 5.969867013 | 4.87E-41 | 1.49E-39 |
| HSA_CIRCpedia_124005 | 1.148072734 | 5.679307416 | 8.32E-41 | 2.49E-39 |
| HSA_CIRCpedia_357445 | -1.742218097 | 5.410065532 | 1.07E-40 | 3.18E-39 |
| HSA_CIRCpedia_144167 | 1.94563883 | 4.338264525 | 1.75E-40 | 5.16E-39 |
| HSA_CIRCpedia_64913 | -2.150558734 | 3.395275148 | 2.73E-40 | 7.92E-39 |
| HSA_CIRCpedia_166374 | 1.953055343 | 5.202381797 | 3.09E-40 | 8.91E-39 |
| HSA_CIRCpedia_357635 | 1.653293019 | 4.356822585 | 4.06E-40 | 1.15E-38 |
| HSA_CIRCpedia_100284 | 1.095818551 | 7.657523148 | 4.22E-40 | 1.20E-38 |
| HSA_CIRCpedia_53787 | 1.095818551 | 7.657523148 | 4.22E-40 | 1.20E-38 |
| HSA_CIRCpedia_53785 | 1.095818551 | 7.657523148 | 4.22E-40 | 1.20E-38 |
| HSA_CIRCpedia_357194 | 1.095818551 | 7.657523148 | 4.22E-40 | 1.20E-38 |
| HSA_CIRCpedia_357190 | 1.095818551 | 7.657523148 | 4.22E-40 | 1.20E-38 |
| HSA_CIRCpedia_357195 | 1.095818551 | 7.657523148 | 4.22E-40 | 1.20E-38 |
| HSA_CIRCpedia_68539 | 1.095818551 | 7.657523148 | 4.22E-40 | 1.20E-38 |
| HSA_CIRCpedia_53768 | 1.095818551 | 7.657523148 | 4.22E-40 | 1.20E-38 |
| HSA_CIRCpedia_53774 | 1.095818551 | 7.657523148 | 4.22E-40 | 1.20E-38 |
| HSA_CIRCpedia_53801 | 1.095818551 | 7.657523148 | 4.22E-40 | 1.20E-38 |
| HSA_CIRCpedia_357192 | 1.095818551 | 7.657523148 | 4.22E-40 | 1.20E-38 |
| HSA_CIRCpedia_53788 | 1.095818551 | 7.657523148 | 4.22E-40 | 1.20E-38 |
| HSA_CIRCpedia_356616 | 1.095818551 | 7.657523148 | 4.22E-40 | 1.20E-38 |
| HSA_CIRCpedia_53776 | 1.095818551 | 7.657523148 | 4.22E-40 | 1.20E-38 |
| HSA_CIRCpedia_53772 | 1.095818551 | 7.657523148 | 4.22E-40 | 1.20E-38 |
| HSA_CIRCpedia_68538 | 1.095818551 | 7.657523148 | 4.22E-40 | 1.20E-38 |
| HSA_CIRCpedia_68546 | 1.095818551 | 7.657523148 | 4.22E-40 | 1.20E-38 |
| HSA_CIRCpedia_357191 | 1.095818551 | 7.657523148 | 4.22E-40 | 1.20E-38 |
| HSA_CIRCpedia_53786 | 1.095818551 | 7.657523148 | 4.22E-40 | 1.20E-38 |
| HSA_CIRCpedia_53781 | 1.095818551 | 7.657523148 | 4.22E-40 | 1.20E-38 |
| HSA_CIRCpedia_357193 | 1.095818551 | 7.657523148 | 4.22E-40 | 1.20E-38 |
| HSA_CIRCpedia_357196 | 1.095818551 | 7.657523148 | 4.22E-40 | 1.20E-38 |
| HSA_CIRCpedia_147421 | 2.026936868 | 5.423766319 | 6.29E-40 | 1.77E-38 |
| HSA_CIRCpedia_123823 | 2.026936868 | 5.423766319 | 6.29E-40 | 1.77E-38 |
| HSA_CIRCpedia_48185 | 2.026936868 | 5.423766319 | 6.29E-40 | 1.77E-38 |
| HSA_CIRCpedia_48189 | 2.026936868 | 5.423766319 | 6.29E-40 | 1.77E-38 |
| HSA_CIRCpedia_357601 | 2.026936868 | 5.423766319 | 6.29E-40 | 1.77E-38 |
| HSA_CIRCpedia_102888 | 2.026936868 | 5.423766319 | 6.29E-40 | 1.77E-38 |
| HSA_CIRCpedia_48191 | 2.026936868 | 5.423766319 | 6.29E-40 | 1.77E-38 |
| HSA_CIRCpedia_48190 | 2.026936868 | 5.423766319 | 6.29E-40 | 1.77E-38 |
| HSA_CIRCpedia_48197 | 2.026936868 | 5.423766319 | 6.29E-40 | 1.77E-38 |

| | | | | |
|---|---|---|---|---|
| HSA_CIRCpedia_358006 | 2.154371707 | 1.049719838 | 4.47E-39 | 1.19E-37 |
| HSA_CIRCpedia_9767 | 1.482816862 | 9.930422024 | 5.34E-39 | 1.41E-37 |
| HSA_CIRCpedia_142414 | 1.856396047 | 1.902892359 | 1.50E-38 | 3.89E-37 |
| HSA_CIRCpedia_118984 | 1.856396047 | 1.902892359 | 1.50E-38 | 3.89E-37 |
| HSA_CIRCpedia_9218 | 1.856396047 | 1.902892359 | 1.50E-38 | 3.89E-37 |
| HSA_CIRCpedia_68377 | 1.454149331 | 5.030869123 | 2.44E-38 | 6.23E-37 |
| HSA_CIRCpedia_57049 | 1.454149331 | 5.030869123 | 2.44E-38 | 6.23E-37 |
| HSA_CIRCpedia_57062 | 1.454149331 | 5.030869123 | 2.44E-38 | 6.23E-37 |
| HSA_CIRCpedia_115141 | 1.08073994 | 5.490321232 | 3.55E-38 | 8.91E-37 |
| HSA_CIRCpedia_52197 | -1.53906261 | 4.324484995 | 1.09E-37 | 2.67E-36 |
| HSA_CIRCpedia_13433 | 1.478761588 | 6.781600047 | 1.18E-37 | 2.89E-36 |
| HSA_CIRCpedia_141733 | 2.867962059 | 2.44701192 | 1.86E-37 | 4.47E-36 |
| HSA_CIRCpedia_11359 | 1.384479083 | 5.403721633 | 2.15E-37 | 5.14E-36 |
| HSA_CIRCpedia_128474 | 2.398312111 | 2.736493014 | 2.95E-37 | 6.99E-36 |
| HSA_CIRCpedia_6028 | 2.398312111 | 2.736493014 | 2.95E-37 | 6.99E-36 |
| HSA_CIRCpedia_119614 | 2.398312111 | 2.736493014 | 2.95E-37 | 6.99E-36 |
| HSA_CIRCpedia_6027 | 2.398312111 | 2.736493014 | 2.95E-37 | 6.99E-36 |
| HSA_CIRCpedia_37224 | 2.374394563 | 4.388997994 | 2.96E-37 | 6.99E-36 |
| HSA_CIRCpedia_37223 | 2.374394563 | 4.388997994 | 2.96E-37 | 6.99E-36 |
| HSA_CIRCpedia_357223 | 1.441428267 | 4.066116936 | 4.69E-37 | 1.10E-35 |
| HSA_CIRCpedia_149037 | 1.957760331 | 5.685691482 | 9.88E-37 | 2.27E-35 |
| HSA_CIRCpedia_15627 | -3.040757701 | 4.384992655 | 1.08E-36 | 2.49E-35 |
| HSA_CIRCpedia_61815 | -1.244923003 | 4.433277148 | 1.15E-36 | 2.64E-35 |
| HSA_CIRCpedia_151289 | 1.940180491 | 5.97069623 | 1.53E-36 | 3.49E-35 |
| HSA_CIRCpedia_32325 | 1.000267067 | 5.978318859 | 3.56E-36 | 7.96E-35 |
| HSA_CIRCpedia_1449 | 1.061051693 | 6.10035385 | 4.95E-36 | 1.10E-34 |
| HSA_CIRCpedia_1451 | 1.061051693 | 6.10035385 | 4.95E-36 | 1.10E-34 |
| HSA_CIRCpedia_1444 | 1.061051693 | 6.10035385 | 4.95E-36 | 1.10E-34 |
| HSA_CIRCpedia_1459 | 1.061051693 | 6.10035385 | 4.95E-36 | 1.10E-34 |
| HSA_CIRCpedia_1452 | 1.061051693 | 6.10035385 | 4.95E-36 | 1.10E-34 |
| HSA_CIRCpedia_126273 | 1.15931685 | 5.570970066 | 5.39E-36 | 1.19E-34 |
| HSA_CIRCpedia_10537 | 1.273922475 | 6.297614202 | 7.08E-36 | 1.55E-34 |
| HSA_CIRCpedia_69918 | 1.273922475 | 6.297614202 | 7.08E-36 | 1.55E-34 |
| HSA_CIRCpedia_68258 | 2.509633592 | 3.071570277 | 7.23E-36 | 1.58E-34 |
| HSA_CIRCpedia_57947 | 2.509633592 | 3.071570277 | 7.23E-36 | 1.58E-34 |
| HSA_CIRCpedia_62219 | 1.844694173 | 5.82869954 | 2.10E-35 | 4.45E-34 |
| HSA_CIRCpedia_48645 | 1.305306442 | 7.927960946 | 2.50E-35 | 5.26E-34 |
| HSA_CIRCpedia_48653 | 1.305306442 | 7.927960946 | 2.50E-35 | 5.26E-34 |
| HSA_CIRCpedia_137287 | 1.305306442 | 7.927960946 | 2.50E-35 | 5.26E-34 |
| HSA_CIRCpedia_137293 | 1.305306442 | 7.927960946 | 2.50E-35 | 5.26E-34 |
| HSA_CIRCpedia_158924 | 1.305306442 | 7.927960946 | 2.50E-35 | 5.26E-34 |
| HSA_CIRCpedia_140129 | -1.139653327 | 6.025706382 | 2.99E-35 | 6.27E-34 |
| HSA_CIRCpedia_28950 | -1.139653327 | 6.025706382 | 2.99E-35 | 6.27E-34 |
| HSA_CIRCpedia_44854 | -1.123563674 | 5.604879977 | 3.42E-35 | 7.17E-34 |
| HSA_CIRCpedia_357755 | -1.956268976 | 10.34462342 | 5.65E-35 | 1.17E-33 |
| HSA_CIRCpedia_144809 | 1.174596929 | 6.812465997 | 6.10E-35 | 1.25E-33 |

| | | | | |
|---|---|---|---|---|
| HSA_CIRCpedia_24434 | 1.174596929 | 6.812465997 | 6.10E-35 | 1.25E-33 |
| HSA_CIRCpedia_24427 | 1.174596929 | 6.812465997 | 6.10E-35 | 1.25E-33 |
| HSA_CIRCpedia_24431 | 1.174596929 | 6.812465997 | 6.10E-35 | 1.25E-33 |
| HSA_CIRCpedia_38638 | -1.054484666 | 6.173533237 | 7.48E-35 | 1.52E-33 |
| HSA_CIRCpedia_38653 | -1.054484666 | 6.173533237 | 7.48E-35 | 1.52E-33 |
| HSA_CIRCpedia_38643 | -1.054484666 | 6.173533237 | 7.48E-35 | 1.52E-33 |
| HSA_CIRCpedia_38642 | -1.054484666 | 6.173533237 | 7.48E-35 | 1.52E-33 |
| HSA_CIRCpedia_11093 | 1.109161613 | 8.486739698 | 1.10E-34 | 2.22E-33 |
| HSA_CIRCpedia_161000 | 1.109161613 | 8.486739698 | 1.10E-34 | 2.22E-33 |
| HSA_CIRCpedia_150891 | 1.218216127 | 6.800563388 | 1.12E-34 | 2.27E-33 |
| HSA_CIRCpedia_65287 | 1.159445351 | 6.189853444 | 1.21E-34 | 2.44E-33 |
| HSA_CIRCpedia_7629 | 2.427747737 | 1.381975989 | 1.60E-34 | 3.20E-33 |
| HSA_CIRCpedia_9877 | 1.358587256 | 6.887977043 | 3.26E-34 | 6.39E-33 |
| HSA_CIRCpedia_65252 | 2.132786284 | 5.698064171 | 3.31E-34 | 6.48E-33 |
| HSA_CIRCpedia_51095 | 1.896966531 | 5.812846835 | 5.10E-34 | 9.88E-33 |
| HSA_CIRCpedia_53530 | 1.104041497 | 6.738901145 | 5.19E-34 | 1.00E-32 |
| HSA_CIRCpedia_53531 | 1.104041497 | 6.738901145 | 5.19E-34 | 1.00E-32 |
| HSA_CIRCpedia_29078 | -1.116775093 | 7.85806375 | 1.10E-33 | 2.07E-32 |
| HSA_CIRCpedia_23008 | 1.132097163 | 7.634494986 | 1.65E-33 | 3.06E-32 |
| HSA_CIRCpedia_23009 | 1.132097163 | 7.634494986 | 1.65E-33 | 3.06E-32 |
| HSA_CIRCpedia_23022 | 1.132097163 | 7.634494986 | 1.65E-33 | 3.06E-32 |
| HSA_CIRCpedia_2888 | 1.284200195 | 6.090141068 | 2.30E-33 | 4.24E-32 |
| HSA_CIRCpedia_2891 | 1.284200195 | 6.090141068 | 2.30E-33 | 4.24E-32 |
| HSA_CIRCpedia_352130 | -1.108249561 | 6.129494626 | 2.46E-33 | 4.53E-32 |
| HSA_CIRCpedia_41044 | 1.073059615 | 7.377804753 | 2.54E-33 | 4.68E-32 |
| HSA_CIRCpedia_41042 | 1.073059615 | 7.377804753 | 2.54E-33 | 4.68E-32 |
| HSA_CIRCpedia_41043 | 1.073059615 | 7.377804753 | 2.54E-33 | 4.68E-32 |
| HSA_CIRCpedia_41048 | 1.073059615 | 7.377804753 | 2.54E-33 | 4.68E-32 |
| HSA_CIRCpedia_41049 | 1.073059615 | 7.377804753 | 2.54E-33 | 4.68E-32 |
| HSA_CIRCpedia_60932 | 1.109355824 | 8.448848939 | 3.40E-33 | 6.21E-32 |
| HSA_CIRCpedia_60929 | 1.109355824 | 8.448848939 | 3.40E-33 | 6.21E-32 |
| HSA_CIRCpedia_358001 | 1.215578034 | 7.349382959 | 4.58E-33 | 8.24E-32 |
| HSA_CIRCpedia_15217 | 1.175170256 | 5.494673395 | 7.02E-33 | 1.25E-31 |
| HSA_CIRCpedia_53352 | 1.329462751 | 7.86094489 | 1.53E-32 | 2.69E-31 |
| HSA_CIRCpedia_51794 | 1.360502429 | 7.092045754 | 2.42E-32 | 4.19E-31 |
| HSA_CIRCpedia_51792 | 1.360502429 | 7.092045754 | 2.42E-32 | 4.19E-31 |
| HSA_CIRCpedia_51796 | 1.360502429 | 7.092045754 | 2.42E-32 | 4.19E-31 |
| HSA_CIRCpedia_136374 | 1.360502429 | 7.092045754 | 2.42E-32 | 4.19E-31 |
| HSA_CIRCpedia_51791 | 1.360502429 | 7.092045754 | 2.42E-32 | 4.19E-31 |
| HSA_CIRCpedia_125788 | 2.199029087 | 6.487573364 | 2.58E-32 | 4.48E-31 |
| HSA_CIRCpedia_125787 | 2.199029087 | 6.487573364 | 2.58E-32 | 4.48E-31 |
| HSA_CIRCpedia_118452 | 2.096845017 | 1.77430588 | 3.01E-32 | 5.18E-31 |
| HSA_CIRCpedia_117981 | 2.096845017 | 1.77430588 | 3.01E-32 | 5.18E-31 |
| HSA_CIRCpedia_25709 | -1.584736343 | 4.897374662 | 3.87E-32 | 6.61E-31 |
| HSA_CIRCpedia_59975 | -1.122163414 | 6.612502943 | 6.17E-32 | 1.04E-30 |
| HSA_CIRCpedia_59987 | -1.122163414 | 6.612502943 | 6.17E-32 | 1.04E-30 |

| | | | | |
|---|---|---|---|---|
| HSA_CIRCpedia_59974 | -1.122163414 | 6.612502943 | 6.17E-32 | 1.04E-30 |
| HSA_CIRCpedia_89636 | -1.122163414 | 6.612502943 | 6.17E-32 | 1.04E-30 |
| HSA_CIRCpedia_59970 | -1.122163414 | 6.612502943 | 6.17E-32 | 1.04E-30 |
| HSA_CIRCpedia_20046 | -1.192720578 | 4.474105568 | 6.27E-32 | 1.06E-30 |
| HSA_CIRCpedia_20047 | -1.192720578 | 4.474105568 | 6.27E-32 | 1.06E-30 |
| HSA_CIRCpedia_20041 | -1.192720578 | 4.474105568 | 6.27E-32 | 1.06E-30 |
| HSA_CIRCpedia_355530 | 1.427272245 | 6.091562333 | 7.48E-32 | 1.26E-30 |
| HSA_CIRCpedia_41745 | 1.394683081 | 7.630925321 | 8.12E-32 | 1.36E-30 |
| HSA_CIRCpedia_61886 | -1.434359893 | 4.794999243 | 9.48E-32 | 1.58E-30 |
| HSA_CIRCpedia_160272 | 1.725494753 | 4.991537183 | 1.08E-31 | 1.80E-30 |
| HSA_CIRCpedia_135013 | 2.30800787 | 4.780820082 | 1.35E-31 | 2.24E-30 |
| HSA_CIRCpedia_357565 | 2.30800787 | 4.780820082 | 1.35E-31 | 2.24E-30 |
| HSA_CIRCpedia_146258 | 2.30800787 | 4.780820082 | 1.35E-31 | 2.24E-30 |
| HSA_CIRCpedia_357564 | 2.30800787 | 4.780820082 | 1.35E-31 | 2.24E-30 |
| HSA_CIRCpedia_357566 | 2.30800787 | 4.780820082 | 1.35E-31 | 2.24E-30 |
| HSA_CIRCpedia_122714 | 2.30800787 | 4.780820082 | 1.35E-31 | 2.24E-30 |
| HSA_CIRCpedia_357049 | 1.20508229 | 5.389137812 | 1.74E-31 | 2.86E-30 |
| HSA_CIRCpedia_357048 | 1.20508229 | 5.389137812 | 1.74E-31 | 2.86E-30 |
| HSA_CIRCpedia_357651 | 3.36187023 | 3.917963295 | 1.94E-31 | 3.18E-30 |
| HSA_CIRCpedia_357927 | -1.158027921 | 5.330637746 | 2.34E-31 | 3.81E-30 |
| HSA_CIRCpedia_357770 | 1.49226034 | 5.013042677 | 3.41E-31 | 5.48E-30 |
| HSA_CIRCpedia_16095 | -1.041037773 | 8.103641975 | 3.80E-31 | 6.10E-30 |
| HSA_CIRCpedia_33057 | -1.42799349 | 3.503135605 | 9.52E-31 | 1.47E-29 |
| HSA_CIRCpedia_33052 | -1.42799349 | 3.503135605 | 9.52E-31 | 1.47E-29 |
| HSA_CIRCpedia_357050 | -1.42799349 | 3.503135605 | 9.52E-31 | 1.47E-29 |
| HSA_CIRCpedia_137638 | 1.691841352 | 7.088306109 | 1.23E-30 | 1.87E-29 |
| HSA_CIRCpedia_11708 | 1.151634031 | 6.549313302 | 1.64E-30 | 2.50E-29 |
| HSA_CIRCpedia_3560 | 1.332660749 | 5.485636807 | 2.01E-30 | 3.03E-29 |
| HSA_CIRCpedia_7993 | 1.626282641 | 2.608402745 | 2.62E-30 | 3.93E-29 |
| HSA_CIRCpedia_356954 | 1.626282641 | 2.608402745 | 2.62E-30 | 3.93E-29 |
| HSA_CIRCpedia_65070 | 1.158371885 | 6.066285534 | 3.80E-30 | 5.65E-29 |
| HSA_CIRCpedia_357898 | 1.158371885 | 6.066285534 | 3.80E-30 | 5.65E-29 |
| HSA_CIRCpedia_154877 | 1.158371885 | 6.066285534 | 3.80E-30 | 5.65E-29 |
| HSA_CIRCpedia_65080 | 1.158371885 | 6.066285534 | 3.80E-30 | 5.65E-29 |
| HSA_CIRCpedia_65068 | 1.158371885 | 6.066285534 | 3.80E-30 | 5.65E-29 |
| HSA_CIRCpedia_357575 | -1.649717044 | 10.10954592 | 4.06E-30 | 6.01E-29 |
| HSA_CIRCpedia_117103 | -1.649717044 | 10.10954592 | 4.06E-30 | 6.01E-29 |
| HSA_CIRCpedia_53221 | 1.373650584 | 4.241314536 | 4.29E-30 | 6.35E-29 |
| HSA_CIRCpedia_53222 | 1.373650584 | 4.241314536 | 4.29E-30 | 6.35E-29 |
| HSA_CIRCpedia_9912 | 1.285304379 | 0.29281824 | 5.53E-30 | 8.12E-29 |
| HSA_CIRCpedia_356949 | 1.199504115 | 5.953360027 | 5.56E-30 | 8.16E-29 |
| HSA_CIRCpedia_54910 | 1.502351053 | 4.557330932 | 6.78E-30 | 9.88E-29 |
| HSA_CIRCpedia_87497 | -2.006857052 | 6.263026874 | 1.27E-29 | 1.82E-28 |
| HSA_CIRCpedia_42939 | 1.316350805 | 6.106075163 | 2.54E-29 | 3.56E-28 |
| HSA_CIRCpedia_42945 | 1.316350805 | 6.106075163 | 2.54E-29 | 3.56E-28 |
| HSA_CIRCpedia_27144 | 2.518101283 | 2.670969864 | 2.76E-29 | 3.86E-28 |

| | | | | |
|---|---|---|---|---|
| HSA_CIRCpedia_125099 | 2.518101283 | 2.670969864 | 2.76E-29 | 3.86E-28 |
| HSA_CIRCpedia_27143 | 2.518101283 | 2.670969864 | 2.76E-29 | 3.86E-28 |
| HSA_CIRCpedia_344104 | 2.386726205 | 2.145538418 | 3.81E-29 | 5.30E-28 |
| HSA_CIRCpedia_146627 | 2.386726205 | 2.145538418 | 3.81E-29 | 5.30E-28 |
| HSA_CIRCpedia_123054 | 2.386726205 | 2.145538418 | 3.81E-29 | 5.30E-28 |
| HSA_CIRCpedia_123055 | 2.386726205 | 2.145538418 | 3.81E-29 | 5.30E-28 |
| HSA_CIRCpedia_123053 | 2.386726205 | 2.145538418 | 3.81E-29 | 5.30E-28 |
| HSA_CIRCpedia_344101 | 2.386726205 | 2.145538418 | 3.81E-29 | 5.30E-28 |
| HSA_CIRCpedia_135700 | 2.386726205 | 2.145538418 | 3.81E-29 | 5.30E-28 |
| HSA_CIRCpedia_37471 | 2.213163864 | 3.470063023 | 3.99E-29 | 5.55E-28 |
| HSA_CIRCpedia_116094 | 1.831731366 | 3.665242507 | 6.64E-29 | 9.10E-28 |
| HSA_CIRCpedia_356977 | 1.628957233 | 4.620994508 | 7.85E-29 | 1.07E-27 |
| HSA_CIRCpedia_9677 | 1.628957233 | 4.620994508 | 7.85E-29 | 1.07E-27 |
| HSA_CIRCpedia_141532 | 1.971773608 | 3.202321148 | 1.35E-28 | 1.82E-27 |
| HSA_CIRCpedia_166321 | 1.971773608 | 3.202321148 | 1.35E-28 | 1.82E-27 |
| HSA_CIRCpedia_61579 | 1.971773608 | 3.202321148 | 1.35E-28 | 1.82E-27 |
| HSA_CIRCpedia_160591 | 1.971773608 | 3.202321148 | 1.35E-28 | 1.82E-27 |
| HSA_CIRCpedia_149547 | 1.971773608 | 3.202321148 | 1.35E-28 | 1.82E-27 |
| HSA_CIRCpedia_54935 | 1.021518287 | 5.697818759 | 2.30E-28 | 3.04E-27 |
| HSA_CIRCpedia_357563 | -1.476529814 | 5.877912656 | 2.58E-28 | 3.39E-27 |
| HSA_CIRCpedia_4564 | 1.203072595 | 6.635743515 | 3.08E-28 | 4.04E-27 |
| HSA_CIRCpedia_4565 | 1.203072595 | 6.635743515 | 3.08E-28 | 4.04E-27 |
| HSA_CIRCpedia_357719 | 1.309378497 | 3.0777312 | 3.23E-28 | 4.21E-27 |
| HSA_CIRCpedia_3078 | 1.309378497 | 3.0777312 | 3.23E-28 | 4.21E-27 |
| HSA_CIRCpedia_48107 | 1.002984437 | 6.452143727 | 3.64E-28 | 4.71E-27 |
| HSA_CIRCpedia_48103 | 1.002984437 | 6.452143727 | 3.64E-28 | 4.71E-27 |
| HSA_CIRCpedia_48097 | 1.002984437 | 6.452143727 | 3.64E-28 | 4.71E-27 |
| HSA_CIRCpedia_48096 | 1.002984437 | 6.452143727 | 3.64E-28 | 4.71E-27 |
| HSA_CIRCpedia_103801 | 1.002984437 | 6.452143727 | 3.64E-28 | 4.71E-27 |
| HSA_CIRCpedia_48109 | 1.002984437 | 6.452143727 | 3.64E-28 | 4.71E-27 |
| HSA_CIRCpedia_75826 | 2.096618267 | 4.212366903 | 4.14E-28 | 5.35E-27 |
| HSA_CIRCpedia_357976 | 2.096618267 | 4.212366903 | 4.14E-28 | 5.35E-27 |
| HSA_CIRCpedia_357972 | 2.096618267 | 4.212366903 | 4.14E-28 | 5.35E-27 |
| HSA_CIRCpedia_357974 | 2.096618267 | 4.212366903 | 4.14E-28 | 5.35E-27 |
| HSA_CIRCpedia_128811 | 2.096618267 | 4.212366903 | 4.14E-28 | 5.35E-27 |
| HSA_CIRCpedia_1106 | 2.096618267 | 4.212366903 | 4.14E-28 | 5.35E-27 |
| HSA_CIRCpedia_1104 | 2.096618267 | 4.212366903 | 4.14E-28 | 5.35E-27 |
| HSA_CIRCpedia_143218 | 2.096618267 | 4.212366903 | 4.14E-28 | 5.35E-27 |
| HSA_CIRCpedia_357973 | 2.096618267 | 4.212366903 | 4.14E-28 | 5.35E-27 |
| HSA_CIRCpedia_357975 | 2.096618267 | 4.212366903 | 4.14E-28 | 5.35E-27 |
| HSA_CIRCpedia_143220 | 2.096618267 | 4.212366903 | 4.14E-28 | 5.35E-27 |
| HSA_CIRCpedia_1107 | 2.096618267 | 4.212366903 | 4.14E-28 | 5.35E-27 |
| HSA_CIRCpedia_75829 | 2.096618267 | 4.212366903 | 4.14E-28 | 5.35E-27 |
| HSA_CIRCpedia_75827 | 2.096618267 | 4.212366903 | 4.14E-28 | 5.35E-27 |
| HSA_CIRCpedia_123035 | 1.844644831 | 2.546893162 | 4.30E-28 | 5.55E-27 |
| HSA_CIRCpedia_123036 | 1.844644831 | 2.546893162 | 4.30E-28 | 5.55E-27 |

| | | | | |
|---|---|---|---|---|
| HSA_CIRCpedia_748 | 1.683420265 | 5.953655331 | 5.49E-28 | 7.05E-27 |
| HSA_CIRCpedia_358015 | 1.683420265 | 5.953655331 | 5.49E-28 | 7.05E-27 |
| HSA_CIRCpedia_746 | 1.683420265 | 5.953655331 | 5.49E-28 | 7.05E-27 |
| HSA_CIRCpedia_155929 | 1.683420265 | 5.953655331 | 5.49E-28 | 7.05E-27 |
| HSA_CIRCpedia_747 | 1.683420265 | 5.953655331 | 5.49E-28 | 7.05E-27 |
| HSA_CIRCpedia_145693 | -1.385197473 | 2.907672237 | 5.62E-28 | 7.21E-27 |
| HSA_CIRCpedia_31797 | 1.428134376 | 6.508461167 | 1.02E-27 | 1.28E-26 |
| HSA_CIRCpedia_127328 | 2.274270189 | 6.950077312 | 1.09E-27 | 1.38E-26 |
| HSA_CIRCpedia_356961 | 2.274270189 | 6.950077312 | 1.09E-27 | 1.38E-26 |
| HSA_CIRCpedia_8389 | 2.274270189 | 6.950077312 | 1.09E-27 | 1.38E-26 |
| HSA_CIRCpedia_357476 | 1.247727621 | 7.224313915 | 1.55E-27 | 1.93E-26 |
| HSA_CIRCpedia_24868 | 1.247727621 | 7.224313915 | 1.55E-27 | 1.93E-26 |
| HSA_CIRCpedia_24869 | 1.247727621 | 7.224313915 | 1.55E-27 | 1.93E-26 |
| HSA_CIRCpedia_26752 | 1.174841206 | 5.381205963 | 1.91E-27 | 2.36E-26 |
| HSA_CIRCpedia_357237 | 1.658014929 | 3.321768925 | 3.55E-27 | 4.33E-26 |
| HSA_CIRCpedia_23781 | 1.540416332 | 6.421856894 | 3.70E-27 | 4.50E-26 |
| HSA_CIRCpedia_23782 | 1.540416332 | 6.421856894 | 3.70E-27 | 4.50E-26 |
| HSA_CIRCpedia_144784 | 1.540416332 | 6.421856894 | 3.70E-27 | 4.50E-26 |
| HSA_CIRCpedia_17061 | 1.957227569 | 8.172641142 | 5.25E-27 | 6.30E-26 |
| HSA_CIRCpedia_17060 | 1.957227569 | 8.172641142 | 5.25E-27 | 6.30E-26 |
| HSA_CIRCpedia_143855 | 1.957227569 | 8.172641142 | 5.25E-27 | 6.30E-26 |
| HSA_CIRCpedia_17056 | 1.957227569 | 8.172641142 | 5.25E-27 | 6.30E-26 |
| HSA_CIRCpedia_16005 | 1.778785344 | 2.133012079 | 5.42E-27 | 6.50E-26 |
| HSA_CIRCpedia_8030 | 1.202460575 | 5.971456318 | 7.00E-27 | 8.32E-26 |
| HSA_CIRCpedia_66558 | 1.202460575 | 5.971456318 | 7.00E-27 | 8.32E-26 |
| HSA_CIRCpedia_8032 | 1.202460575 | 5.971456318 | 7.00E-27 | 8.32E-26 |
| HSA_CIRCpedia_47885 | 1.400706788 | 3.678014327 | 7.26E-27 | 8.62E-26 |
| HSA_CIRCpedia_64545 | 2.107465188 | 3.976746985 | 1.41E-26 | 1.65E-25 |
| HSA_CIRCpedia_64546 | 2.107465188 | 3.976746985 | 1.41E-26 | 1.65E-25 |
| HSA_CIRCpedia_56405 | 1.059409112 | 6.677532343 | 2.56E-26 | 2.95E-25 |
| HSA_CIRCpedia_146262 | 1.386790939 | 5.188856176 | 3.25E-26 | 3.71E-25 |
| HSA_CIRCpedia_67259 | -1.509645068 | 8.024710548 | 4.39E-26 | 4.97E-25 |
| HSA_CIRCpedia_151912 | 2.429336771 | 3.528012181 | 6.74E-26 | 7.55E-25 |
| HSA_CIRCpedia_151915 | 2.429336771 | 3.528012181 | 6.74E-26 | 7.55E-25 |
| HSA_CIRCpedia_113393 | 1.44831633 | 4.710958641 | 8.04E-26 | 8.94E-25 |
| HSA_CIRCpedia_129203 | 1.304972624 | 5.424302884 | 9.55E-26 | 1.06E-24 |
| HSA_CIRCpedia_2841 | 1.304972624 | 5.424302884 | 9.55E-26 | 1.06E-24 |
| HSA_CIRCpedia_2842 | 1.304972624 | 5.424302884 | 9.55E-26 | 1.06E-24 |
| HSA_CIRCpedia_2843 | 1.304972624 | 5.424302884 | 9.55E-26 | 1.06E-24 |
| HSA_CIRCpedia_1818 | 1.433951492 | 3.820308104 | 2.17E-25 | 2.32E-24 |
| HSA_CIRCpedia_357261 | -1.409231077 | 5.669383188 | 3.25E-25 | 3.44E-24 |
| HSA_CIRCpedia_122924 | 1.343757561 | 0.237639553 | 3.63E-25 | 3.82E-24 |
| HSA_CIRCpedia_34332 | -1.479043238 | 5.75791053 | 5.74E-25 | 5.93E-24 |
| HSA_CIRCpedia_45266 | 1.315562524 | 3.526427516 | 1.13E-24 | 1.14E-23 |
| HSA_CIRCpedia_45270 | 1.315562524 | 3.526427516 | 1.13E-24 | 1.14E-23 |
| HSA_CIRCpedia_6931 | 1.206704454 | 5.015805557 | 1.45E-24 | 1.45E-23 |

| | | | | |
|---|---|---|---|---|
| HSA_CIRCpedia_357839 | -1.245321408 | 4.753930487 | 1.62E-24 | 1.63E-23 |
| HSA_CIRCpedia_11182 | 2.259126093 | 2.137601096 | 2.32E-24 | 2.30E-23 |
| HSA_CIRCpedia_43226 | 1.040281593 | 7.50847052 | 4.05E-24 | 3.95E-23 |
| HSA_CIRCpedia_150668 | 1.048581304 | 4.946747368 | 5.30E-24 | 5.13E-23 |
| HSA_CIRCpedia_150659 | 1.048581304 | 4.946747368 | 5.30E-24 | 5.13E-23 |
| HSA_CIRCpedia_8184 | 1.298429748 | 4.244656815 | 6.93E-24 | 6.63E-23 |
| HSA_CIRCpedia_21631 | -1.610288631 | 4.0256366 | 1.01E-23 | 9.53E-23 |
| HSA_CIRCpedia_357043 | 2.673041808 | 1.862554939 | 1.05E-23 | 9.90E-23 |
| HSA_CIRCpedia_47362 | 1.258423505 | 5.574652915 | 1.42E-23 | 1.33E-22 |
| HSA_CIRCpedia_357648 | 1.258423505 | 5.574652915 | 1.42E-23 | 1.33E-22 |
| HSA_CIRCpedia_357766 | 1.401169107 | 4.354511721 | 2.33E-23 | 2.15E-22 |
| HSA_CIRCpedia_79635 | 1.401169107 | 4.354511721 | 2.33E-23 | 2.15E-22 |
| HSA_CIRCpedia_114351 | 1.120800823 | 2.610117835 | 3.25E-23 | 2.97E-22 |
| HSA_CIRCpedia_5995 | 1.120800823 | 2.610117835 | 3.25E-23 | 2.97E-22 |
| HSA_CIRCpedia_47287 | 1.067578961 | 3.734229028 | 3.32E-23 | 3.03E-22 |
| HSA_CIRCpedia_47291 | 1.067578961 | 3.734229028 | 3.32E-23 | 3.03E-22 |
| HSA_CIRCpedia_47297 | 1.067578961 | 3.734229028 | 3.32E-23 | 3.03E-22 |
| HSA_CIRCpedia_47296 | 1.067578961 | 3.734229028 | 3.32E-23 | 3.03E-22 |
| HSA_CIRCpedia_47294 | 1.067578961 | 3.734229028 | 3.32E-23 | 3.03E-22 |
| HSA_CIRCpedia_47288 | 1.067578961 | 3.734229028 | 3.32E-23 | 3.03E-22 |
| HSA_CIRCpedia_357646 | 1.067578961 | 3.734229028 | 3.32E-23 | 3.03E-22 |
| HSA_CIRCpedia_135106 | 2.381393113 | 4.669604054 | 4.18E-23 | 3.80E-22 |
| HSA_CIRCpedia_152289 | 1.081927164 | 6.261814589 | 5.59E-23 | 5.03E-22 |
| HSA_CIRCpedia_57854 | 1.828383517 | 2.569380283 | 7.24E-23 | 6.48E-22 |
| HSA_CIRCpedia_12836 | 1.491352815 | 1.856075567 | 9.01E-23 | 8.01E-22 |
| HSA_CIRCpedia_43256 | 1.448222139 | 5.582174842 | 2.50E-22 | 2.17E-21 |
| HSA_CIRCpedia_113163 | 1.355430792 | 7.342078552 | 2.65E-22 | 2.30E-21 |
| HSA_CIRCpedia_357832 | 1.888529534 | 4.178417506 | 3.01E-22 | 2.60E-21 |
| HSA_CIRCpedia_105153 | 1.888529534 | 4.178417506 | 3.01E-22 | 2.60E-21 |
| HSA_CIRCpedia_116182 | 1.888529534 | 4.178417506 | 3.01E-22 | 2.60E-21 |
| HSA_CIRCpedia_134166 | 1.203076565 | 5.779513752 | 4.72E-22 | 4.02E-21 |
| HSA_CIRCpedia_357508 | 1.203076565 | 5.779513752 | 4.72E-22 | 4.02E-21 |
| HSA_CIRCpedia_357511 | 1.203076565 | 5.779513752 | 4.72E-22 | 4.02E-21 |
| HSA_CIRCpedia_32636 | 1.734226949 | 3.733266161 | 4.72E-22 | 4.02E-21 |
| HSA_CIRCpedia_357510 | 1.203076565 | 5.779513752 | 4.72E-22 | 4.02E-21 |
| HSA_CIRCpedia_357513 | 1.203076565 | 5.779513752 | 4.72E-22 | 4.02E-21 |
| HSA_CIRCpedia_357514 | 1.203076565 | 5.779513752 | 4.72E-22 | 4.02E-21 |
| HSA_CIRCpedia_41373 | 1.203076565 | 5.779513752 | 4.72E-22 | 4.02E-21 |
| HSA_CIRCpedia_357509 | 1.203076565 | 5.779513752 | 4.72E-22 | 4.02E-21 |
| HSA_CIRCpedia_41374 | 1.203076565 | 5.779513752 | 4.72E-22 | 4.02E-21 |
| HSA_CIRCpedia_41370 | 1.203076565 | 5.779513752 | 4.72E-22 | 4.02E-21 |
| HSA_CIRCpedia_41372 | 1.203076565 | 5.779513752 | 4.72E-22 | 4.02E-21 |
| HSA_CIRCpedia_41371 | 1.203076565 | 5.779513752 | 4.72E-22 | 4.02E-21 |
| HSA_CIRCpedia_41375 | 1.203076565 | 5.779513752 | 4.72E-22 | 4.02E-21 |
| HSA_CIRCpedia_357515 | 1.203076565 | 5.779513752 | 4.72E-22 | 4.02E-21 |
| HSA_CIRCpedia_41378 | 1.203076565 | 5.779513752 | 4.72E-22 | 4.02E-21 |

| | | | | |
|---|---|---|---|---|
| HSA_CIRCpedia_357512 | 1.203076565 | 5.779513752 | 4.72E-22 | 4.02E-21 |
| HSA_CIRCpedia_134167 | 1.203076565 | 5.779513752 | 4.72E-22 | 4.02E-21 |
| HSA_CIRCpedia_122316 | 1.203076565 | 5.779513752 | 4.72E-22 | 4.02E-21 |
| HSA_CIRCpedia_63375 | -1.256145879 | 8.300951339 | 5.95E-22 | 5.04E-21 |
| HSA_CIRCpedia_49199 | 1.625022949 | 5.335418979 | 6.20E-22 | 5.25E-21 |
| HSA_CIRCpedia_65036 | 1.403167033 | 4.133693028 | 1.07E-21 | 8.92E-21 |
| HSA_CIRCpedia_126424 | 1.403167033 | 4.133693028 | 1.07E-21 | 8.92E-21 |
| HSA_CIRCpedia_152644 | 1.836755061 | 5.794140912 | 1.16E-21 | 9.63E-21 |
| HSA_CIRCpedia_357477 | 1.836755061 | 5.794140912 | 1.16E-21 | 9.63E-21 |
| HSA_CIRCpedia_357478 | 1.836755061 | 5.794140912 | 1.16E-21 | 9.63E-21 |
| HSA_CIRCpedia_357288 | 2.06881278 | 2.478865963 | 1.59E-21 | 1.30E-20 |
| HSA_CIRCpedia_125996 | 2.06881278 | 2.478865963 | 1.59E-21 | 1.30E-20 |
| HSA_CIRCpedia_125998 | 2.06881278 | 2.478865963 | 1.59E-21 | 1.30E-20 |
| HSA_CIRCpedia_125999 | 2.06881278 | 2.478865963 | 1.59E-21 | 1.30E-20 |
| HSA_CIRCpedia_357767 | 1.472054511 | 2.313292803 | 3.52E-21 | 2.82E-20 |
| HSA_CIRCpedia_79637 | 1.472054511 | 2.313292803 | 3.52E-21 | 2.82E-20 |
| HSA_CIRCpedia_53984 | 1.061686308 | 7.390166826 | 4.10E-21 | 3.26E-20 |
| HSA_CIRCpedia_164331 | 1.061686308 | 7.390166826 | 4.10E-21 | 3.26E-20 |
| HSA_CIRCpedia_53968 | 1.061686308 | 7.390166826 | 4.10E-21 | 3.26E-20 |
| HSA_CIRCpedia_100392 | 1.061686308 | 7.390166826 | 4.10E-21 | 3.26E-20 |
| HSA_CIRCpedia_100387 | 1.061686308 | 7.390166826 | 4.10E-21 | 3.26E-20 |
| HSA_CIRCpedia_153709 | 1.673278831 | 0.906193219 | 4.36E-21 | 3.46E-20 |
| HSA_CIRCpedia_357105 | 1.039222467 | 6.153891686 | 4.96E-21 | 3.92E-20 |
| HSA_CIRCpedia_357161 | 1.409786306 | 5.053126605 | 6.71E-21 | 5.27E-20 |
| HSA_CIRCpedia_101466 | 2.010855109 | 2.167423603 | 8.97E-21 | 6.98E-20 |
| HSA_CIRCpedia_113749 | 2.010855109 | 2.167423603 | 8.97E-21 | 6.98E-20 |
| HSA_CIRCpedia_123606 | 2.010855109 | 2.167423603 | 8.97E-21 | 6.98E-20 |
| HSA_CIRCpedia_132294 | 1.267669183 | 3.923318218 | 1.05E-20 | 8.16E-20 |
| HSA_CIRCpedia_132296 | 1.267669183 | 3.923318218 | 1.05E-20 | 8.16E-20 |
| HSA_CIRCpedia_37020 | 1.066650564 | 7.969050453 | 1.15E-20 | 8.88E-20 |
| HSA_CIRCpedia_91036 | 1.066650564 | 7.969050453 | 1.15E-20 | 8.88E-20 |
| HSA_CIRCpedia_121480 | 1.158299709 | 6.479690393 | 1.20E-20 | 9.21E-20 |
| HSA_CIRCpedia_51590 | 1.377570721 | 4.059644221 | 1.84E-20 | 1.40E-19 |
| HSA_CIRCpedia_154773 | 1.188038073 | 3.433748584 | 4.60E-20 | 3.41E-19 |
| HSA_CIRCpedia_36914 | 1.041363166 | 5.700256395 | 5.27E-20 | 3.88E-19 |
| HSA_CIRCpedia_57718 | -1.062398855 | 4.358934701 | 5.35E-20 | 3.94E-19 |
| HSA_CIRCpedia_57707 | -1.062398855 | 4.358934701 | 5.35E-20 | 3.94E-19 |
| HSA_CIRCpedia_57715 | -1.062398855 | 4.358934701 | 5.35E-20 | 3.94E-19 |
| HSA_CIRCpedia_57706 | -1.062398855 | 4.358934701 | 5.35E-20 | 3.94E-19 |
| HSA_CIRCpedia_57708 | -1.062398855 | 4.358934701 | 5.35E-20 | 3.94E-19 |
| HSA_CIRCpedia_57717 | -1.062398855 | 4.358934701 | 5.35E-20 | 3.94E-19 |
| HSA_CIRCpedia_142456 | 1.081628128 | 5.429485405 | 6.78E-20 | 4.95E-19 |
| HSA_CIRCpedia_69132 | 1.573116864 | 3.493399556 | 9.78E-20 | 7.06E-19 |
| HSA_CIRCpedia_17426 | 1.043691748 | 6.554623488 | 1.11E-19 | 7.97E-19 |
| HSA_CIRCpedia_357306 | 1.944858428 | 6.872921881 | 2.96E-19 | 2.07E-18 |
| HSA_CIRCpedia_357308 | 1.944858428 | 6.872921881 | 2.96E-19 | 2.07E-18 |

| | | | | |
|---|---|---|---|---|
| HSA_CIRCpedia_357307 | 1.944858428 | 6.872921881 | 2.96E-19 | 2.07E-18 |
| HSA_CIRCpedia_169165 | 1.047420573 | 7.62202514 | 3.17E-19 | 2.21E-18 |
| HSA_CIRCpedia_357278 | 1.23459716 | 3.12815859 | 9.36E-19 | 6.30E-18 |
| HSA_CIRCpedia_61777 | 1.23459716 | 3.12815859 | 9.36E-19 | 6.30E-18 |
| HSA_CIRCpedia_61782 | 1.23459716 | 3.12815859 | 9.36E-19 | 6.30E-18 |
| HSA_CIRCpedia_61775 | 1.23459716 | 3.12815859 | 9.36E-19 | 6.30E-18 |
| HSA_CIRCpedia_64509 | 1.170483013 | 4.58735609 | 1.15E-18 | 7.66E-18 |
| HSA_CIRCpedia_64516 | 1.170483013 | 4.58735609 | 1.15E-18 | 7.66E-18 |
| HSA_CIRCpedia_140663 | 1.170483013 | 4.58735609 | 1.15E-18 | 7.66E-18 |
| HSA_CIRCpedia_125473 | 1.170483013 | 4.58735609 | 1.15E-18 | 7.66E-18 |
| HSA_CIRCpedia_125467 | 1.170483013 | 4.58735609 | 1.15E-18 | 7.66E-18 |
| HSA_CIRCpedia_357737 | 2.637191772 | 2.910346717 | 2.08E-18 | 1.37E-17 |
| HSA_CIRCpedia_143604 | 2.637191772 | 2.910346717 | 2.08E-18 | 1.37E-17 |
| HSA_CIRCpedia_97943 | 1.413528727 | 3.813136151 | 4.26E-18 | 2.73E-17 |
| HSA_CIRCpedia_59146 | 1.413528727 | 3.813136151 | 4.26E-18 | 2.73E-17 |
| HSA_CIRCpedia_59147 | 1.413528727 | 3.813136151 | 4.26E-18 | 2.73E-17 |
| HSA_CIRCpedia_14889 | 1.82160723 | 4.293212141 | 5.52E-18 | 3.52E-17 |
| HSA_CIRCpedia_159031 | 1.315962247 | 4.164693262 | 7.21E-18 | 4.54E-17 |
| HSA_CIRCpedia_49449 | 1.315962247 | 4.164693262 | 7.21E-18 | 4.54E-17 |
| HSA_CIRCpedia_357631 | 1.315962247 | 4.164693262 | 7.21E-18 | 4.54E-17 |
| HSA_CIRCpedia_124025 | 1.315962247 | 4.164693262 | 7.21E-18 | 4.54E-17 |
| HSA_CIRCpedia_49450 | 1.315962247 | 4.164693262 | 7.21E-18 | 4.54E-17 |
| HSA_CIRCpedia_52151 | 1.031775255 | 4.490220445 | 1.11E-17 | 6.92E-17 |
| HSA_CIRCpedia_123205 | 1.110563364 | 4.389017042 | 1.17E-17 | 7.23E-17 |
| HSA_CIRCpedia_123239 | 1.344348901 | 4.905915682 | 1.20E-17 | 7.43E-17 |
| HSA_CIRCpedia_65321 | 1.564418832 | 3.497524862 | 2.07E-17 | 1.26E-16 |
| HSA_CIRCpedia_65320 | 1.564418832 | 3.497524862 | 2.07E-17 | 1.26E-16 |
| HSA_CIRCpedia_5807 | 1.591526366 | 3.206517611 | 2.26E-17 | 1.37E-16 |
| HSA_CIRCpedia_53956 | 1.284650879 | 6.485235957 | 2.53E-17 | 1.53E-16 |
| HSA_CIRCpedia_16956 | 2.150764028 | 2.230059578 | 2.62E-17 | 1.59E-16 |
| HSA_CIRCpedia_16955 | 2.150764028 | 2.230059578 | 2.62E-17 | 1.59E-16 |
| HSA_CIRCpedia_120400 | 2.150764028 | 2.230059578 | 2.62E-17 | 1.59E-16 |
| HSA_CIRCpedia_357122 | 1.058628737 | 5.239188707 | 4.21E-17 | 2.50E-16 |
| HSA_CIRCpedia_23287 | -1.85029131 | 2.417070753 | 5.79E-17 | 3.40E-16 |
| HSA_CIRCpedia_357591 | 1.701241288 | 5.419196108 | 1.95E-16 | 1.11E-15 |
| HSA_CIRCpedia_104113 | 1.118872349 | 5.046001248 | 3.84E-16 | 2.14E-15 |
| HSA_CIRCpedia_123850 | 1.720400801 | 2.394681579 | 5.73E-16 | 3.16E-15 |
| HSA_CIRCpedia_155407 | 1.529730421 | 3.704418976 | 8.61E-16 | 4.68E-15 |
| HSA_CIRCpedia_128040 | 1.529730421 | 3.704418976 | 8.61E-16 | 4.68E-15 |
| HSA_CIRCpedia_58841 | 1.015173818 | 5.190005292 | 9.19E-16 | 4.98E-15 |
| HSA_CIRCpedia_357270 | 1.926476756 | 3.005412156 | 1.53E-15 | 8.15E-15 |
| HSA_CIRCpedia_357268 | 1.926476756 | 3.005412156 | 1.53E-15 | 8.15E-15 |
| HSA_CIRCpedia_357273 | 1.926476756 | 3.005412156 | 1.53E-15 | 8.15E-15 |
| HSA_CIRCpedia_357269 | 1.926476756 | 3.005412156 | 1.53E-15 | 8.15E-15 |
| HSA_CIRCpedia_357271 | 1.926476756 | 3.005412156 | 1.53E-15 | 8.15E-15 |
| HSA_CIRCpedia_357272 | 1.926476756 | 3.005412156 | 1.53E-15 | 8.15E-15 |

| | | | | |
|---|---|---|---|---|
| HSA_CIRCpedia_136114 | 1.516507703 | 2.626289976 | 1.88E-15 | 9.96E-15 |
| HSA_CIRCpedia_30835 | 1.252843985 | 3.65249827 | 2.06E-15 | 1.09E-14 |
| HSA_CIRCpedia_30856 | 1.252843985 | 3.65249827 | 2.06E-15 | 1.09E-14 |
| HSA_CIRCpedia_30831 | 1.252843985 | 3.65249827 | 2.06E-15 | 1.09E-14 |
| HSA_CIRCpedia_30855 | 1.252843985 | 3.65249827 | 2.06E-15 | 1.09E-14 |
| HSA_CIRCpedia_30854 | 1.252843985 | 3.65249827 | 2.06E-15 | 1.09E-14 |
| HSA_CIRCpedia_357101 | 1.252843985 | 3.65249827 | 2.06E-15 | 1.09E-14 |
| HSA_CIRCpedia_30836 | 1.252843985 | 3.65249827 | 2.06E-15 | 1.09E-14 |
| HSA_CIRCpedia_30833 | 1.252843985 | 3.65249827 | 2.06E-15 | 1.09E-14 |
| HSA_CIRCpedia_139069 | 1.912474685 | 5.674105311 | 2.74E-15 | 1.44E-14 |
| HSA_CIRCpedia_149553 | 1.56524559 | 3.054449007 | 3.22E-15 | 1.68E-14 |
| HSA_CIRCpedia_141541 | 1.56524559 | 3.054449007 | 3.22E-15 | 1.68E-14 |
| HSA_CIRCpedia_48806 | 1.412139983 | 4.907207266 | 4.45E-15 | 2.29E-14 |
| HSA_CIRCpedia_356999 | 1.068325503 | 4.275924087 | 4.54E-15 | 2.33E-14 |
| HSA_CIRCpedia_32727 | 1.218530547 | 5.714793803 | 6.84E-15 | 3.47E-14 |
| HSA_CIRCpedia_118312 | 1.218530547 | 5.714793803 | 6.84E-15 | 3.47E-14 |
| HSA_CIRCpedia_32724 | 1.218530547 | 5.714793803 | 6.84E-15 | 3.47E-14 |
| HSA_CIRCpedia_32738 | 1.218530547 | 5.714793803 | 6.84E-15 | 3.47E-14 |
| HSA_CIRCpedia_32726 | 1.218530547 | 5.714793803 | 6.84E-15 | 3.47E-14 |
| HSA_CIRCpedia_32735 | 1.218530547 | 5.714793803 | 6.84E-15 | 3.47E-14 |
| HSA_CIRCpedia_357044 | 1.218530547 | 5.714793803 | 6.84E-15 | 3.47E-14 |
| HSA_CIRCpedia_98451 | 2.124931778 | 4.126360977 | 9.56E-15 | 4.81E-14 |
| HSA_CIRCpedia_168344 | 2.124931778 | 4.126360977 | 9.56E-15 | 4.81E-14 |
| HSA_CIRCpedia_108808 | 1.656870439 | 2.324722866 | 1.02E-14 | 5.12E-14 |
| HSA_CIRCpedia_39795 | -1.005227913 | 5.860898375 | 1.27E-14 | 6.35E-14 |
| HSA_CIRCpedia_357402 | 1.892980198 | 5.043027052 | 2.17E-14 | 1.06E-13 |
| HSA_CIRCpedia_152856 | 1.892980198 | 5.043027052 | 2.17E-14 | 1.06E-13 |
| HSA_CIRCpedia_121770 | 1.892980198 | 5.043027052 | 2.17E-14 | 1.06E-13 |
| HSA_CIRCpedia_357403 | 1.892980198 | 5.043027052 | 2.17E-14 | 1.06E-13 |
| HSA_CIRCpedia_357401 | 1.892980198 | 5.043027052 | 2.17E-14 | 1.06E-13 |
| HSA_CIRCpedia_345236 | 1.892980198 | 5.043027052 | 2.17E-14 | 1.06E-13 |
| HSA_CIRCpedia_47626 | 1.036446841 | 5.587432153 | 3.02E-14 | 1.47E-13 |
| HSA_CIRCpedia_57662 | -1.724490353 | 4.976088676 | 5.21E-14 | 2.49E-13 |
| HSA_CIRCpedia_57660 | -1.724490353 | 4.976088676 | 5.21E-14 | 2.49E-13 |
| HSA_CIRCpedia_39369 | 1.002413335 | 3.909376609 | 7.31E-14 | 3.45E-13 |
| HSA_CIRCpedia_39365 | 1.002413335 | 3.909376609 | 7.31E-14 | 3.45E-13 |
| HSA_CIRCpedia_39368 | 1.002413335 | 3.909376609 | 7.31E-14 | 3.45E-13 |
| HSA_CIRCpedia_39367 | 1.002413335 | 3.909376609 | 7.31E-14 | 3.45E-13 |
| HSA_CIRCpedia_123375 | 1.117096512 | 0.552994624 | 2.79E-13 | 1.26E-12 |
| HSA_CIRCpedia_49467 | 1.150348939 | 6.867292046 | 3.55E-13 | 1.59E-12 |
| HSA_CIRCpedia_49464 | 1.150348939 | 6.867292046 | 3.55E-13 | 1.59E-12 |
| HSA_CIRCpedia_137544 | 1.150348939 | 6.867292046 | 3.55E-13 | 1.59E-12 |
| HSA_CIRCpedia_49459 | 1.150348939 | 6.867292046 | 3.55E-13 | 1.59E-12 |
| HSA_CIRCpedia_49457 | 1.150348939 | 6.867292046 | 3.55E-13 | 1.59E-12 |
| HSA_CIRCpedia_49463 | 1.150348939 | 6.867292046 | 3.55E-13 | 1.59E-12 |
| HSA_CIRCpedia_137545 | 1.150348939 | 6.867292046 | 3.55E-13 | 1.59E-12 |

| | | | | |
|---|---|---|---|---|
| HSA_CIRCpedia_49478 | 1.150348939 | 6.867292046 | 3.55E-13 | 1.59E-12 |
| HSA_CIRCpedia_357632 | 1.150348939 | 6.867292046 | 3.55E-13 | 1.59E-12 |
| HSA_CIRCpedia_164902 | 1.150348939 | 6.867292046 | 3.55E-13 | 1.59E-12 |
| HSA_CIRCpedia_103314 | 1.150348939 | 6.867292046 | 3.55E-13 | 1.59E-12 |
| HSA_CIRCpedia_124032 | 1.150348939 | 6.867292046 | 3.55E-13 | 1.59E-12 |
| HSA_CIRCpedia_49479 | 1.150348939 | 6.867292046 | 3.55E-13 | 1.59E-12 |
| HSA_CIRCpedia_357864 | 1.07472068 | 3.162740349 | 3.84E-13 | 1.71E-12 |
| HSA_CIRCpedia_26228 | 1.52537988 | 4.896223665 | 6.14E-13 | 2.69E-12 |
| HSA_CIRCpedia_26227 | 1.52537988 | 4.896223665 | 6.14E-13 | 2.69E-12 |
| HSA_CIRCpedia_357489 | 1.52537988 | 4.896223665 | 6.14E-13 | 2.69E-12 |
| HSA_CIRCpedia_26226 | 1.52537988 | 4.896223665 | 6.14E-13 | 2.69E-12 |
| HSA_CIRCpedia_26231 | 1.52537988 | 4.896223665 | 6.14E-13 | 2.69E-12 |
| HSA_CIRCpedia_23464 | 1.399436258 | 6.889725061 | 7.06E-13 | 3.08E-12 |
| HSA_CIRCpedia_25177 | 1.147012365 | 4.372817805 | 9.09E-13 | 3.93E-12 |
| HSA_CIRCpedia_3277 | 1.011922477 | 2.772312603 | 1.32E-12 | 5.63E-12 |
| HSA_CIRCpedia_357722 | 1.011922477 | 2.772312603 | 1.32E-12 | 5.63E-12 |
| HSA_CIRCpedia_47228 | 1.523139393 | 4.558035009 | 1.50E-12 | 6.35E-12 |
| HSA_CIRCpedia_65630 | 1.173537785 | 2.168494442 | 1.75E-12 | 7.37E-12 |
| HSA_CIRCpedia_65632 | 1.173537785 | 2.168494442 | 1.75E-12 | 7.37E-12 |
| HSA_CIRCpedia_357821 | 1.782752531 | 2.282184918 | 2.21E-12 | 9.23E-12 |
| HSA_CIRCpedia_355234 | 1.782752531 | 2.282184918 | 2.21E-12 | 9.23E-12 |
| HSA_CIRCpedia_343727 | 1.782752531 | 2.282184918 | 2.21E-12 | 9.23E-12 |
| HSA_CIRCpedia_124445 | 1.782752531 | 2.282184918 | 2.21E-12 | 9.23E-12 |
| HSA_CIRCpedia_148009 | 1.782752531 | 2.282184918 | 2.21E-12 | 9.23E-12 |
| HSA_CIRCpedia_357822 | 1.782752531 | 2.282184918 | 2.21E-12 | 9.23E-12 |
| HSA_CIRCpedia_357823 | 1.782752531 | 2.282184918 | 2.21E-12 | 9.23E-12 |
| HSA_CIRCpedia_357824 | 1.782752531 | 2.282184918 | 2.21E-12 | 9.23E-12 |
| HSA_CIRCpedia_108698 | 1.061557467 | 4.210348169 | 2.70E-12 | 1.12E-11 |
| HSA_CIRCpedia_32818 | 1.061557467 | 4.210348169 | 2.70E-12 | 1.12E-11 |
| HSA_CIRCpedia_165625 | 1.061557467 | 4.210348169 | 2.70E-12 | 1.12E-11 |
| HSA_CIRCpedia_139581 | 1.061557467 | 4.210348169 | 2.70E-12 | 1.12E-11 |
| HSA_CIRCpedia_117783 | 1.061557467 | 4.210348169 | 2.70E-12 | 1.12E-11 |
| HSA_CIRCpedia_32817 | 1.061557467 | 4.210348169 | 2.70E-12 | 1.12E-11 |
| HSA_CIRCpedia_122079 | 1.92839465 | 2.60758356 | 2.72E-12 | 1.13E-11 |
| HSA_CIRCpedia_115680 | 1.31634048 | 3.232586941 | 7.14E-12 | 2.87E-11 |
| HSA_CIRCpedia_357392 | 1.31634048 | 3.232586941 | 7.14E-12 | 2.87E-11 |
| HSA_CIRCpedia_124683 | 1.465213433 | 2.247460926 | 7.73E-12 | 3.10E-11 |
| HSA_CIRCpedia_357857 | 1.465213433 | 2.247460926 | 7.73E-12 | 3.10E-11 |
| HSA_CIRCpedia_357858 | 1.465213433 | 2.247460926 | 7.73E-12 | 3.10E-11 |
| HSA_CIRCpedia_124682 | 1.465213433 | 2.247460926 | 7.73E-12 | 3.10E-11 |
| HSA_CIRCpedia_48725 | 1.027884636 | 3.680504537 | 7.98E-12 | 3.19E-11 |
| HSA_CIRCpedia_47973 | 1.205378896 | 3.252032633 | 9.80E-12 | 3.89E-11 |
| HSA_CIRCpedia_357939 | -1.490252446 | 2.656159794 | 1.17E-11 | 4.61E-11 |
| HSA_CIRCpedia_54931 | 1.259117682 | 2.7437387 | 1.27E-11 | 4.99E-11 |
| HSA_CIRCpedia_134449 | 1.37746233 | 3.218437347 | 1.40E-11 | 5.49E-11 |
| HSA_CIRCpedia_122480 | 1.37746233 | 3.218437347 | 1.40E-11 | 5.49E-11 |

| | | | | |
|---|---|---|---|---|
| HSA_CIRCpedia_148257 | 1.746093896 | 2.02631719 | 2.24E-11 | 8.61E-11 |
| HSA_CIRCpedia_66120 | 1.058863239 | 3.305224156 | 3.14E-11 | 1.19E-10 |
| HSA_CIRCpedia_75926 | 1.52507235 | 2.864019043 | 4.49E-11 | 1.67E-10 |
| HSA_CIRCpedia_1379 | 1.52507235 | 2.864019043 | 4.49E-11 | 1.67E-10 |
| HSA_CIRCpedia_1380 | 1.52507235 | 2.864019043 | 4.49E-11 | 1.67E-10 |
| HSA_CIRCpedia_121797 | 1.017573263 | 2.33873066 | 1.47E-10 | 5.24E-10 |
| HSA_CIRCpedia_75879 | 1.834773213 | 3.483466951 | 5.36E-10 | 1.83E-09 |
| HSA_CIRCpedia_356978 | 1.287878031 | 3.089243938 | 6.69E-10 | 2.26E-09 |
| HSA_CIRCpedia_71984 | 1.287878031 | 3.089243938 | 6.69E-10 | 2.26E-09 |
| HSA_CIRCpedia_161231 | 1.287878031 | 3.089243938 | 6.69E-10 | 2.26E-09 |
| HSA_CIRCpedia_356979 | 1.287878031 | 3.089243938 | 6.69E-10 | 2.26E-09 |
| HSA_CIRCpedia_356980 | 1.287878031 | 3.089243938 | 6.69E-10 | 2.26E-09 |
| HSA_CIRCpedia_119201 | 1.287878031 | 3.089243938 | 6.69E-10 | 2.26E-09 |
| HSA_CIRCpedia_122798 | 1.072056108 | 3.363121612 | 6.86E-10 | 2.32E-09 |
| HSA_CIRCpedia_93224 | 1.072056108 | 3.363121612 | 6.86E-10 | 2.32E-09 |
| HSA_CIRCpedia_122799 | 1.072056108 | 3.363121612 | 6.86E-10 | 2.32E-09 |
| HSA_CIRCpedia_54248 | 1.160315962 | 6.098561831 | 1.20E-09 | 3.96E-09 |
| HSA_CIRCpedia_357174 | 1.336098558 | 6.550734332 | 3.32E-09 | 1.06E-08 |
| HSA_CIRCpedia_357173 | 1.336098558 | 6.550734332 | 3.32E-09 | 1.06E-08 |
| HSA_CIRCpedia_357176 | 1.336098558 | 6.550734332 | 3.32E-09 | 1.06E-08 |
| HSA_CIRCpedia_357175 | 1.336098558 | 6.550734332 | 3.32E-09 | 1.06E-08 |
| HSA_CIRCpedia_357178 | 1.336098558 | 6.550734332 | 3.32E-09 | 1.06E-08 |
| HSA_CIRCpedia_357177 | 1.336098558 | 6.550734332 | 3.32E-09 | 1.06E-08 |
| HSA_CIRCpedia_357769 | 1.281512235 | 3.141401446 | 5.19E-09 | 1.62E-08 |
| HSA_CIRCpedia_151042 | 1.164766111 | 3.589743361 | 2.01E-08 | 5.99E-08 |
| HSA_CIRCpedia_120487 | 1.078372953 | 4.241493969 | 2.82E-08 | 8.28E-08 |
| HSA_CIRCpedia_105970 | 1.021527298 | 1.594019469 | 4.57E-08 | 1.32E-07 |
| HSA_CIRCpedia_44470 | 1.021527298 | 1.594019469 | 4.57E-08 | 1.32E-07 |
| HSA_CIRCpedia_37360 | 1.066125677 | 3.008201338 | 5.29E-08 | 1.52E-07 |
| HSA_CIRCpedia_357062 | -1.204374828 | 2.107633281 | 6.23E-08 | 1.77E-07 |
| HSA_CIRCpedia_104125 | 1.090841803 | 2.053254548 | 7.20E-08 | 2.04E-07 |
| HSA_CIRCpedia_124326 | 1.090841803 | 2.053254548 | 7.20E-08 | 2.04E-07 |
| HSA_CIRCpedia_357602 | 1.036656149 | 10.11097705 | 1.09E-07 | 3.03E-07 |
| HSA_CIRCpedia_9881 | -1.209660903 | 3.630144208 | 2.59E-07 | 6.97E-07 |
| HSA_CIRCpedia_100188 | 1.062387537 | 2.398044708 | 4.90E-07 | 1.29E-06 |
| HSA_CIRCpedia_23786 | 1.121560635 | 2.076122632 | 5.21E-07 | 1.36E-06 |

Table Supplementary 3: Differentially expressed lncRNAs of LUAD

| lncRNA-id | logFC | AveExpr | P.Value | adj.P.Val |
|---|---|---|---|---|
| UCA1 | -1.707979709 | 1.85434226 | 1.92E-10 | 6.79E-10 |
| PVT1 | -1.706513577 | 4.467109458 | 3.56E-50 | 1.92E-48 |
| DGCR9 | -1.661168575 | 2.980882356 | 1.09E-18 | 7.28E-18 |
| C2orf48 | -1.510610343 | 1.46533896 | 8.64E-21 | 6.73E-20 |
| C1orf220 | -1.50464868 | 2.013572536 | 3.07E-33 | 5.62E-32 |
| HOTAIR | -1.269132323 | 1.06895149 | 1.51E-08 | 4.56E-08 |
| SNHG4 | -1.170767653 | 2.013723752 | 2.38E-20 | 1.80E-19 |
| FLJ12825 | -1.13571049 | 1.467983763 | 2.34E-13 | 1.06E-12 |
| C20orf197 | -1.115037212 | 2.545890673 | 6.78E-08 | 1.92E-07 |
| SNHG3 | -1.018143761 | 4.143024943 | 1.67E-23 | 1.56E-22 |
| KIAA0087 | 1.053760939 | 0.367051687 | 2.54E-18 | 1.66E-17 |
| DKFZp779M0652 | 1.066184017 | 0.973556228 | 1.42E-16 | 8.13E-16 |
| FAM95B1 | 1.072107952 | 2.830987382 | 1.74E-09 | 5.67E-09 |
| INE2 | 1.163968931 | 1.301366216 | 4.18E-11 | 1.56E-10 |
| RMST | 1.285304379 | 0.29281824 | 5.53E-30 | 8.12E-29 |
| AGAP11 | 1.671951909 | 2.751327491 | 5.17E-30 | 7.61E-29 |
| SFTA1P | 2.238925069 | 4.96152204 | 4.18E-22 | 3.57E-21 |
| MGC27382 | 2.626023483 | 0.795064324 | 5.52E-62 | 7.27E-60 |

Table Supplementary 4: KEGG analysis of DEGs

| Pathway | All_Unigene | qvalue | richFactor |
|---|---|---|---|
| Wnt signaling pathway | 122 | 0.002113553 | 3 |
| ERBB signaling pathway | 55 | 1.52E-06 | 2 |
| ephrin receptor signaling pathway | 31 | 0.000733366 | 1.75 |
| epidermal growth factor receptor signaling pathway | 46 | 1.74E-05 | 1.25 |
| ErbB signaling pathway | 36 | 1.99E-06 | 1 |
| MAPK signaling pathway | 79 | 0.002551331 | 2.5 |
| mTOR signaling pathway | 47 | 0.000994651 | 2.25 |
| Non-small cell lung cancer | 24 | 0.001444923 | 1.5 |

Table Supplementary 5: Hub driving genes were selected based on PPI Network

| Symbol | logFC | AveExpr | P.Value | adj.P.Val |
|---|---|---|---|---|
| SFTPC | 5.965548022 | 7.793483642 | 1.20E-38 | 3.12E-37 |
| SLC6A4 | 5.822445505 | 2.347200636 | 2.75E-84 | 3.14E-81 |
| ITLN2 | 4.970585463 | 1.762531857 | 4.55E-77 | 2.17E-74 |
| CLDN18 | 4.960270856 | 5.57052065 | 3.74E-41 | 1.15E-39 |
| LGI3 | 4.779978809 | 3.227739445 | 2.22E-55 | 1.71E-53 |
| C13orf36 | 4.741437888 | 1.488387961 | 6.74E-93 | 2.77E-89 |
| AGER | 4.693623332 | 6.471155601 | 5.71E-74 | 1.99E-71 |
| CD300LG | 4.399526681 | 1.664473451 | 1.59E-76 | 6.66E-74 |
| FABP4 | 4.343763138 | 3.956220625 | 3.77E-75 | 1.38E-72 |
| GPM6A | 4.263887681 | 3.253939052 | 3.46E-71 | 9.48E-69 |
| CA4 | 4.252755899 | 2.737415109 | 2.99E-55 | 2.27E-53 |
| HBA1 | 4.231314078 | 4.210514717 | 1.23E-42 | 4.16E-41 |
| UPK3B | 4.164315688 | 4.081405715 | 1.57E-55 | 1.22E-53 |
| ANKRD1 | 4.118495294 | 3.168839565 | 1.58E-56 | 1.32E-54 |
| SCGB1A1 | 4.105268608 | 5.509245273 | 2.16E-22 | 1.88E-21 |
| CNTN6 | 3.96993595 | 2.159717104 | 3.90E-57 | 3.44E-55 |
| CPB2 | 3.916695838 | 3.076489379 | 7.05E-37 | 1.63E-35 |
| SH3GL3 | 3.89777528 | 1.054349378 | 2.62E-90 | 6.72E-87 |
| GKN2 | 3.866140028 | 3.415607128 | 3.49E-31 | 5.61E-30 |
| SOSTDC1 | 3.865401604 | 2.802547107 | 6.84E-44 | 2.51E-42 |
| WIF1 | 3.852962994 | 4.583039707 | 1.51E-32 | 2.64E-31 |
| LOC149620 | 3.839554257 | 1.692238888 | 3.38E-51 | 1.95E-49 |
| RPL13AP17 | 3.839442346 | 1.868299003 | 2.34E-57 | 2.11E-55 |
| PTPRQ | 3.830945481 | 1.248976677 | 7.02E-84 | 7.59E-81 |
| WNT3A | 3.768969645 | 2.272857527 | 1.15E-66 | 2.14E-64 |
| GRIA1 | 3.765724438 | 2.613262958 | 3.16E-54 | 2.21E-52 |
| SLCO1A2 | 3.716495305 | 1.641099484 | 2.21E-45 | 8.92E-44 |
| MYOC | 3.631835976 | 1.012390359 | 2.05E-85 | 2.48E-82 |
| C10orf67 | 3.623519796 | 1.168509601 | 1.81E-87 | 2.86E-84 |
| CHRM1 | 3.600629317 | 1.105994032 | 1.14E-76 | 5.14E-74 |
| FIGF | 3.585798524 | 3.728881294 | 8.47E-44 | 3.07E-42 |
| C19orf59 | 3.574165143 | 4.640624086 | 1.24E-54 | 8.97E-53 |
| TMEM100 | 3.516233713 | 4.58934492 | 1.08E-60 | 1.26E-58 |
| F11 | 3.50851712 | 2.148018944 | 5.56E-41 | 1.68E-39 |
| AGTR2 | 3.507991598 | 3.159732627 | 1.22E-29 | 1.75E-28 |
| SFTPA1 | 3.497492913 | 9.323637888 | 8.83E-24 | 8.41E-23 |
| ANGPTL7 | 3.483906577 | 0.777650493 | 1.48E-89 | 3.05E-86 |
| GPD1 | 3.477105966 | 4.00683733 | 8.47E-60 | 8.97E-58 |
| ADCY8 | 3.472184436 | 0.730599494 | 1.54E-77 | 7.92E-75 |
| MS4A15 | 3.463964681 | 4.260266853 | 2.19E-28 | 2.89E-27 |
| FAM107A | 3.45614528 | 5.245926324 | 1.02E-80 | 6.73E-78 |
| KIAA0408 | 3.450107322 | 3.389251996 | 4.36E-31 | 6.95E-30 |
| RS1 | 3.421287499 | 1.451834313 | 3.00E-82 | 2.40E-79 |

| | | | | |
|---|---|---|---|---|
| FCN3 | 3.417996175 | 4.839033311 | 1.14E-61 | 1.48E-59 |
| HBB | 3.412590194 | 6.245297543 | 7.84E-53 | 4.97E-51 |
| KRT4 | 3.40696451 | 3.521784017 | 6.46E-33 | 1.16E-31 |
| ADH1B | 3.400825656 | 6.157537821 | 6.00E-33 | 1.07E-31 |
| PEBP4 | 3.39661705 | 4.56345091 | 3.58E-29 | 4.99E-28 |
| SFTPA2 | 3.366956562 | 10.07706548 | 1.07E-23 | 1.01E-22 |
| HHIP | 3.36187023 | 3.917963295 | 1.94E-31 | 3.18E-30 |
| ODAM | 3.355772606 | 0.931483878 | 1.52E-69 | 3.64E-67 |
| C8B | 3.355251607 | 2.232380243 | 8.05E-34 | 1.53E-32 |
| OVCH1 | 3.331684363 | 0.653282509 | 3.84E-92 | 1.32E-88 |
| SGCG | 3.328360625 | 1.507236162 | 3.47E-78 | 1.83E-75 |
| HBA2 | 3.327310145 | 6.074467882 | 1.13E-59 | 1.16E-57 |
| KCNA4 | 3.318598773 | 0.943866564 | 3.71E-81 | 2.63E-78 |
| ITLN1 | 3.303763708 | 1.820289494 | 8.24E-38 | 2.03E-36 |
| AQP4 | 3.273021612 | 6.6048242 | 1.23E-27 | 1.54E-26 |
| ADAMTS8 | 3.265044726 | 4.021885913 | 2.65E-49 | 1.36E-47 |
| NCKAP5 | 3.25169054 | 4.107114786 | 1.23E-62 | 1.71E-60 |
| TRHDE | 3.241400447 | 1.943838197 | 4.63E-46 | 1.94E-44 |
| SCUBE1 | 3.220867318 | 3.19009257 | 2.86E-63 | 4.19E-61 |
| ANGPT4 | 3.211481987 | 0.556476542 | 1.21E-104 | 1.24E-100 |
| CLIC5 | 3.196443133 | 6.199644898 | 4.97E-63 | 7.13E-61 |
| ACADL | 3.182319895 | 3.358623922 | 5.47E-41 | 1.66E-39 |
| RTKN2 | 3.181543975 | 5.697670686 | 9.86E-98 | 6.74E-94 |
| TNNC1 | 3.171708107 | 4.398378575 | 1.58E-52 | 9.92E-51 |
| GPIHBP1 | 3.168970496 | 3.92429811 | 7.92E-64 | 1.24E-61 |
| RBP2 | 3.161844274 | 0.773700783 | 1.74E-67 | 3.50E-65 |
| CD5L | 3.156360929 | 0.835257028 | 1.96E-87 | 2.87E-84 |
| RETN | 3.142197342 | 2.395100506 | 5.83E-37 | 1.36E-35 |
| PLA2G1B | 3.141899981 | 3.010911647 | 1.56E-29 | 2.22E-28 |
| PCDH15 | 3.072365691 | 1.355784377 | 7.96E-54 | 5.37E-52 |
| CYP4B1 | 3.067413315 | 6.570000466 | 3.22E-28 | 4.19E-27 |
| FOLR3 | 3.051358709 | 1.233321855 | 1.78E-49 | 9.23E-48 |
| ANXA8L2 | 3.048972931 | 3.742248489 | 3.04E-33 | 5.57E-32 |
| RSPO1 | 3.020034737 | 2.0176615 | 1.07E-57 | 9.74E-56 |
| SH3GL2 | 3.009941663 | 1.431570803 | 4.91E-43 | 1.70E-41 |
| GDF10 | 3.008461639 | 3.642680923 | 1.57E-47 | 7.11E-46 |
| PI16 | 3.008111642 | 2.199824059 | 1.99E-39 | 5.46E-38 |
| SEC14L3 | 2.993391982 | 1.036374604 | 1.41E-43 | 5.05E-42 |
| WNT7A | 2.984868503 | 2.484405299 | 2.25E-30 | 3.38E-29 |
| HTR3C | 2.983687703 | 0.439979424 | 1.46E-83 | 1.36E-80 |
| GPA33 | 2.962624007 | 2.985103142 | 4.25E-46 | 1.79E-44 |
| LRRC36 | 2.953048228 | 3.403625217 | 1.13E-48 | 5.42E-47 |
| CSF3 | 2.950728347 | 2.679483713 | 7.54E-29 | 1.03E-27 |
| C2orf71 | 2.947275561 | 0.878442248 | 1.26E-70 | 3.24E-68 |
| CLEC3B | 2.94699397 | 5.540011589 | 7.35E-74 | 2.47E-71 |
| CYP1A2 | 2.946107131 | 0.514130603 | 1.58E-81 | 1.16E-78 |

| | | | | |
|---|---|---|---|---|
| CCDC141 | 2.942081182 | 2.66404929 | 2.97E-59 | 2.97E-57 |
| INMT | 2.938918682 | 6.051932671 | 2.75E-57 | 2.46E-55 |
| ABCA8 | 2.932660704 | 4.443030782 | 3.24E-42 | 1.07E-40 |
| RXRG | 2.91291817 | 2.149334475 | 6.91E-38 | 1.71E-36 |
| FAM150B | 2.907579279 | 2.00733441 | 2.97E-53 | 1.93E-51 |
| MAPK4 | 2.907443083 | 3.003826064 | 1.37E-28 | 1.84E-27 |
| ST8SIA6 | 2.894589651 | 1.369688534 | 4.06E-60 | 4.49E-58 |
| ADH1A | 2.884393795 | 1.731040901 | 6.66E-49 | 3.27E-47 |
| C2orf40 | 2.871754466 | 2.788287255 | 1.54E-31 | 2.55E-30 |
| CYP3A7 | 2.8706499 | 0.76505544 | 6.20E-69 | 1.40E-66 |
| DNASE1L3 | 2.869790157 | 3.171431952 | 3.34E-39 | 9.01E-38 |
| RSPO2 | 2.867962059 | 2.44701192 | 1.86E-37 | 4.47E-36 |
| LOC723809 | 2.867276472 | 2.932679528 | 8.94E-29 | 1.22E-27 |
| BTNL9 | 2.865278056 | 4.303379509 | 6.95E-59 | 6.86E-57 |
| ADRA1A | 2.848019993 | 0.75336401 | 2.70E-73 | 8.80E-71 |
| SFRP5 | 2.846119797 | 2.214389515 | 1.02E-32 | 1.81E-31 |
| IHH | 2.84453784 | 1.959170324 | 1.41E-33 | 2.65E-32 |
| CRTAC1 | 2.833141346 | 5.548592701 | 2.05E-30 | 3.09E-29 |
| NXF3 | 2.832110945 | 1.450440187 | 2.05E-38 | 5.25E-37 |
| CAV1 | 2.828324774 | 7.777042604 | 1.16E-78 | 6.44E-76 |
| SYN2 | 2.827410588 | 1.37790907 | 6.38E-53 | 4.07E-51 |
| SLC27A6 | 2.817902937 | 0.962791359 | 2.73E-49 | 1.39E-47 |
| IL1RL1 | 2.815943292 | 3.498592255 | 4.45E-36 | 9.89E-35 |
| ADRB1 | 2.815239447 | 3.745542908 | 3.88E-43 | 1.34E-41 |
| ANXA8 | 2.813887534 | 3.993430273 | 2.82E-27 | 3.46E-26 |
| RXFP1 | 2.809327768 | 1.917062339 | 1.23E-61 | 1.58E-59 |
| FGFBP2 | 2.807788898 | 2.070356186 | 1.87E-57 | 1.69E-55 |
| PGC | 2.804135933 | 6.556870886 | 2.54E-11 | 9.69E-11 |
| NOS1 | 2.788286822 | 1.295174983 | 3.27E-38 | 8.24E-37 |
| DES | 2.775048261 | 4.885927757 | 7.01E-39 | 1.83E-37 |
| ACTN2 | 2.769665676 | 1.829896703 | 4.81E-52 | 2.95E-50 |
| FHL1 | 2.765226621 | 6.403245027 | 1.04E-75 | 3.90E-73 |
| MARCO | 2.752936123 | 6.522926429 | 8.46E-39 | 2.20E-37 |
| CACNA2D2 | 2.750112401 | 6.239443603 | 2.02E-27 | 2.49E-26 |
| STXBP6 | 2.736092745 | 3.373133255 | 3.36E-50 | 1.83E-48 |
| GYPE | 2.721314189 | 2.048675178 | 4.00E-72 | 1.19E-69 |
| COL6A6 | 2.719232778 | 4.404382178 | 6.88E-38 | 1.70E-36 |
| ALAS2 | 2.716564531 | 0.58963272 | 1.90E-62 | 2.58E-60 |
| CLEC4M | 2.711699591 | 0.578929727 | 2.75E-63 | 4.06E-61 |
| PTPN5 | 2.68649989 | 0.735541252 | 6.11E-62 | 7.99E-60 |
| EDNRB | 2.685404635 | 5.722973813 | 5.68E-80 | 3.43E-77 |
| MGAT3 | 2.680295721 | 4.845713862 | 2.30E-60 | 2.61E-58 |
| SFTPD | 2.680289376 | 7.469132762 | 6.98E-25 | 7.17E-24 |
| C1orf87 | 2.673041808 | 1.862554939 | 1.05E-23 | 9.90E-23 |
| C20orf85 | 2.672634822 | 3.128804162 | 8.41E-16 | 4.57E-15 |
| CAMP | 2.652439431 | 1.518183626 | 9.13E-41 | 2.73E-39 |

| | | | | |
|---|---|---|---|---|
| FOSB | 2.651740988 | 6.60826939 | 6.83E-29 | 9.35E-28 |
| IRX1 | 2.650401871 | 2.379767865 | 4.69E-23 | 4.25E-22 |
| TCF21 | 2.650394607 | 5.045238553 | 6.15E-55 | 4.54E-53 |
| SCARA5 | 2.641188126 | 3.563598166 | 2.91E-26 | 3.34E-25 |
| SLC5A9 | 2.640463841 | 2.818818291 | 1.57E-41 | 4.98E-40 |
| DNAH9 | 2.637191772 | 2.910346717 | 2.08E-18 | 1.37E-17 |
| MGC27382 | 2.626023483 | 0.795064324 | 5.52E-62 | 7.27E-60 |
| PPBP | 2.624966084 | 1.94405926 | 1.17E-27 | 1.47E-26 |
| TCEAL2 | 2.621015405 | 2.452600401 | 1.60E-43 | 5.70E-42 |
| TRIM58 | 2.617274495 | 2.150194623 | 1.64E-45 | 6.69E-44 |
| CHRNA2 | 2.616791116 | 0.353913509 | 1.07E-85 | 1.38E-82 |
| NECAB1 | 2.614688123 | 3.888198824 | 1.17E-67 | 2.42E-65 |
| PRKG2 | 2.613590585 | 2.672340247 | 6.09E-45 | 2.38E-43 |
| SDPR | 2.604962456 | 6.643802888 | 1.50E-67 | 3.04E-65 |
| ARC | 2.602644103 | 2.767727588 | 2.69E-46 | 1.15E-44 |
| FMO2 | 2.600938569 | 6.326995869 | 4.44E-57 | 3.88E-55 |
| GCOM1 | 2.599922233 | 4.795540329 | 4.40E-70 | 1.09E-67 |
| EMR3 | 2.594591573 | 1.940769299 | 7.29E-45 | 2.83E-43 |
| IGSF10 | 2.594012789 | 4.578838708 | 1.02E-46 | 4.46E-45 |
| FXYD1 | 2.580280428 | 3.397087106 | 2.60E-42 | 8.58E-41 |
| PKHD1L1 | 2.58009675 | 2.652584822 | 9.86E-45 | 3.77E-43 |
| CNTFR | 2.577713751 | 1.313384076 | 1.60E-37 | 3.85E-36 |
| CD36 | 2.575421769 | 5.935923114 | 5.06E-57 | 4.36E-55 |
| DPP6 | 2.573941309 | 1.538432713 | 2.27E-49 | 1.17E-47 |
| VEPH1 | 2.568746228 | 4.738773438 | 1.10E-35 | 2.38E-34 |
| DUOX1 | 2.566458314 | 6.175157634 | 1.75E-36 | 3.99E-35 |
| SYT15 | 2.562218729 | 4.783676263 | 1.85E-51 | 1.11E-49 |
| SLC19A3 | 2.557928833 | 3.942262211 | 1.12E-50 | 6.35E-49 |
| AGRP | 2.548046235 | 1.21221161 | 3.92E-46 | 1.65E-44 |
| EDN3 | 2.547842952 | 0.654154744 | 4.37E-49 | 2.18E-47 |
| FAM189A2 | 2.545927698 | 5.032915037 | 4.93E-45 | 1.95E-43 |
| SLC6A13 | 2.538100724 | 0.970489801 | 1.08E-54 | 7.87E-53 |
| BDNF | 2.530587608 | 3.044241828 | 2.96E-40 | 8.54E-39 |
| MME | 2.528523332 | 5.373662842 | 1.94E-45 | 7.85E-44 |
| LRRN3 | 2.528358217 | 3.363650837 | 2.32E-55 | 1.78E-53 |
| ASPG | 2.524585008 | 1.592682771 | 1.23E-20 | 9.45E-20 |
| MAMDC2 | 2.521930363 | 4.851615387 | 3.14E-37 | 7.40E-36 |
| KCNT2 | 2.520863891 | 2.979638469 | 1.10E-59 | 1.15E-57 |
| NTNG1 | 2.518101283 | 2.670969864 | 2.76E-29 | 3.86E-28 |
| BMPER | 2.509633592 | 3.071570277 | 7.23E-36 | 1.58E-34 |
| PRG4 | 2.507145912 | 4.392165586 | 1.02E-25 | 1.12E-24 |
| AADAC | 2.503916252 | 3.093961703 | 1.06E-21 | 8.84E-21 |
| OVCH2 | 2.493210925 | 0.436267119 | 2.11E-79 | 1.24E-76 |
| COLEC10 | 2.492855208 | 1.170274449 | 4.80E-54 | 3.32E-52 |
| C11orf88 | 2.481387707 | 2.275572964 | 4.33E-19 | 2.98E-18 |
| RASGRF1 | 2.479817424 | 4.926406269 | 3.05E-29 | 4.26E-28 |

| | | | | |
|---|---|---|---|---|
| TEK | 2.478572863 | 5.337073846 | 8.91E-81 | 6.10E-78 |
| CASQ2 | 2.471744736 | 2.301991472 | 2.85E-36 | 6.43E-35 |
| SLITRK2 | 2.467958993 | 1.267352953 | 3.46E-45 | 1.38E-43 |
| ADRB2 | 2.463109106 | 4.560570618 | 3.61E-59 | 3.60E-57 |
| LYVE1 | 2.457657551 | 4.767914731 | 1.28E-62 | 1.76E-60 |
| SPOCK2 | 2.454517255 | 7.288265016 | 1.58E-76 | 6.66E-74 |
| EMR1 | 2.452743875 | 3.224274083 | 1.31E-39 | 3.65E-38 |
| LHFPL3 | 2.450655049 | 2.016764119 | 9.40E-28 | 1.19E-26 |
| C10orf116 | 2.448412833 | 5.91287764 | 1.40E-43 | 5.05E-42 |
| CDH19 | 2.443736787 | 1.065507158 | 7.67E-45 | 2.97E-43 |
| C13orf30 | 2.441366575 | 3.318992202 | 6.00E-18 | 3.80E-17 |
| VIPR1 | 2.439812613 | 5.450036793 | 2.16E-51 | 1.28E-49 |
| MASP1 | 2.435970142 | 3.534943912 | 6.87E-44 | 2.51E-42 |
| ZBTB16 | 2.429336771 | 3.528012181 | 6.74E-26 | 7.55E-25 |
| DNASE2B | 2.428489334 | 1.836855555 | 1.78E-35 | 3.81E-34 |
| TMEM132C | 2.427747737 | 1.381975989 | 1.60E-34 | 3.20E-33 |
| NRG3 | 2.420391869 | 1.15409787 | 7.00E-51 | 4.02E-49 |
| SUSD2 | 2.407035369 | 7.465478015 | 1.55E-28 | 2.07E-27 |
| TEKT1 | 2.401491181 | 2.478014341 | 1.88E-15 | 9.97E-15 |
| FAT3 | 2.398312111 | 2.736493014 | 2.95E-37 | 6.99E-36 |
| PLA2G4F | 2.395218852 | 4.971169962 | 3.97E-36 | 8.86E-35 |
| TNR | 2.394210997 | 0.429804914 | 2.73E-62 | 3.64E-60 |
| FPR2 | 2.394033482 | 3.662660221 | 3.33E-44 | 1.23E-42 |
| AGBL1 | 2.390695044 | 0.555444037 | 3.83E-73 | 1.23E-70 |
| PRX | 2.387000289 | 5.530818095 | 6.72E-75 | 2.42E-72 |
| KHDRBS2 | 2.386726205 | 2.145538418 | 3.81E-29 | 5.30E-28 |
| ALPP | 2.385926519 | 2.690334798 | 2.12E-20 | 1.61E-19 |
| OGN | 2.383449427 | 4.056300479 | 3.38E-28 | 4.39E-27 |
| ZNF385B | 2.381393113 | 4.669604054 | 4.18E-23 | 3.80E-22 |
| AFF3 | 2.374394563 | 4.388997994 | 2.96E-37 | 6.99E-36 |
| GRIK4 | 2.369593957 | 1.448342955 | 5.47E-49 | 2.71E-47 |
| RBP4 | 2.366410687 | 4.058564127 | 2.35E-28 | 3.10E-27 |
| MGC42105 | 2.362646866 | 2.547399398 | 3.69E-37 | 8.69E-36 |
| TNXB | 2.354783843 | 6.367859065 | 9.91E-45 | 3.78E-43 |
| WFDC12 | 2.354310313 | 0.850918927 | 8.66E-33 | 1.54E-31 |
| CCBE1 | 2.353713446 | 3.512697369 | 1.46E-31 | 2.42E-30 |
| LAMP3 | 2.353067235 | 7.446681235 | 9.17E-50 | 4.84E-48 |
| GALNT13 | 2.351886233 | 3.040806538 | 1.22E-23 | 1.15E-22 |
| SCN1A | 2.347308837 | 3.17104993 | 1.03E-23 | 9.71E-23 |
| SSTR1 | 2.347264175 | 3.781379956 | 1.71E-30 | 2.60E-29 |
| PLAC2 | 2.343209648 | 3.345437579 | 3.49E-33 | 6.35E-32 |
| HSD17B6 | 2.330767697 | 5.594526623 | 1.30E-37 | 3.17E-36 |
| KAL1 | 2.330733718 | 6.344650181 | 4.35E-55 | 3.26E-53 |
| PCOLCE2 | 2.324243375 | 4.356228478 | 7.36E-31 | 1.15E-29 |
| CCL23 | 2.321247978 | 2.591342649 | 1.81E-34 | 3.60E-33 |
| FHL5 | 2.320716778 | 3.493292586 | 2.46E-50 | 1.36E-48 |

| | | | | |
|---|---|---|---|---|
| TMEM90A | 2.317940882 | 2.170293351 | 2.09E-31 | 3.43E-30 |
| CDO1 | 2.316558415 | 3.318554434 | 5.02E-48 | 2.34E-46 |
| SCN4B | 2.315732522 | 4.608331623 | 2.02E-50 | 1.13E-48 |
| XAGE2 | 2.315211642 | 1.451161382 | 4.35E-18 | 2.79E-17 |
| DCC | 2.313629239 | 1.517426387 | 1.17E-39 | 3.28E-38 |
| SCN7A | 2.30800787 | 4.780820082 | 1.35E-31 | 2.24E-30 |
| C1orf173 | 2.306654936 | 2.877796791 | 1.33E-15 | 7.11E-15 |
| DUSP27 | 2.301295748 | 0.780616683 | 6.89E-40 | 1.94E-38 |
| C8orf85 | 2.298243343 | 2.729384422 | 1.58E-27 | 1.97E-26 |
| FAM189A1 | 2.296743184 | 2.245277671 | 3.95E-36 | 8.82E-35 |
| CAV3 | 2.296195762 | 0.442051651 | 5.63E-67 | 1.09E-64 |
| CYP2F1 | 2.29233214 | 1.395321867 | 1.25E-19 | 8.94E-19 |
| STX11 | 2.288445208 | 5.398364432 | 1.27E-87 | 2.17E-84 |
| DEFA1B | 2.286211182 | 0.643632758 | 4.52E-39 | 1.20E-37 |
| PLAC9 | 2.284252965 | 3.907889736 | 4.36E-49 | 2.18E-47 |
| TMEM190 | 2.280860343 | 2.112683739 | 4.58E-15 | 2.36E-14 |
| RSPO4 | 2.280405108 | 3.227447416 | 5.33E-29 | 7.37E-28 |
| SLC14A1 | 2.279592619 | 3.221909727 | 3.48E-41 | 1.08E-39 |
| KLB | 2.278075736 | 3.12447658 | 1.00E-29 | 1.45E-28 |
| LRRK2 | 2.274270189 | 6.950077312 | 1.09E-27 | 1.38E-26 |
| ASPA | 2.273156728 | 1.733867924 | 4.85E-49 | 2.42E-47 |
| DNAI2 | 2.270304806 | 1.8818418 | 8.19E-17 | 4.76E-16 |
| ALOX15 | 2.269957814 | 3.279789758 | 2.61E-15 | 1.37E-14 |
| FRMD3 | 2.267849189 | 2.654366779 | 2.65E-54 | 1.87E-52 |
| PCSK9 | 2.262052583 | 4.600007903 | 5.59E-21 | 4.41E-20 |
| GPC5 | 2.259126093 | 2.137601096 | 2.32E-24 | 2.30E-23 |
| C1QTNF7 | 2.257831363 | 3.862496724 | 3.55E-36 | 7.93E-35 |
| BTNL8 | 2.257281107 | 1.292099195 | 7.88E-35 | 1.60E-33 |
| CHRDL1 | 2.254082109 | 5.484133998 | 2.69E-28 | 3.54E-27 |
| CTNND2 | 2.253363309 | 2.478678179 | 1.13E-12 | 4.83E-12 |
| MYH2 | 2.251782697 | 0.50064199 | 3.75E-55 | 2.83E-53 |
| FGFR4 | 2.24794387 | 5.289559987 | 2.41E-35 | 5.08E-34 |
| LIN7A | 2.245920105 | 2.254580939 | 6.10E-47 | 2.70E-45 |
| PLA2G3 | 2.244454913 | 2.663604803 | 1.56E-20 | 1.19E-19 |
| ANGPTL1 | 2.243620493 | 3.535521349 | 7.48E-54 | 5.07E-52 |
| LRRC18 | 2.243401571 | 1.538585257 | 1.46E-28 | 1.95E-27 |
| IQSEC3 | 2.239413412 | 2.836330583 | 4.74E-46 | 1.97E-44 |
| SFTA1P | 2.238925069 | 4.96152204 | 4.18E-22 | 3.57E-21 |
| SLC46A2 | 2.238390513 | 4.470339025 | 7.13E-27 | 8.48E-26 |
| ERBB4 | 2.238267387 | 3.424178871 | 1.20E-26 | 1.41E-25 |
| OTC | 2.23751753 | 0.31685763 | 2.08E-80 | 1.29E-77 |
| FAM181A | 2.234697802 | 1.629457845 | 1.95E-24 | 1.95E-23 |
| CCDC85A | 2.232959198 | 4.152956233 | 9.65E-53 | 6.06E-51 |
| KL | 2.231918567 | 3.945135312 | 1.43E-47 | 6.47E-46 |
| C8orf84 | 2.231217079 | 3.918700337 | 5.73E-56 | 4.64E-54 |
| PCSK2 | 2.229437828 | 2.802973228 | 1.55E-09 | 5.08E-09 |

| | | | | |
|---|---|---|---|---|
| FAM46B | 2.22795138 | 4.665704725 | 3.50E-58 | 3.33E-56 |
| HMGCS2 | 2.221384797 | 1.934972948 | 1.34E-13 | 6.19E-13 |
| CYP17A1 | 2.219346029 | 0.951262219 | 1.83E-35 | 3.89E-34 |
| ACOXL | 2.213163864 | 3.470063023 | 3.99E-29 | 5.55E-28 |
| BCHE | 2.212454688 | 3.554254913 | 1.60E-38 | 4.11E-37 |
| DACH1 | 2.208973026 | 3.910777265 | 6.97E-48 | 3.22E-46 |
| RAMP3 | 2.206775433 | 5.619581312 | 2.14E-76 | 8.80E-74 |
| BMX | 2.201126012 | 2.225640864 | 5.96E-34 | 1.14E-32 |
| LPL | 2.199029087 | 6.487573364 | 2.58E-32 | 4.48E-31 |
| SCGB3A2 | 2.197990438 | 7.016274437 | 1.45E-10 | 5.17E-10 |
| KCNK3 | 2.197000162 | 4.853425627 | 3.30E-32 | 5.66E-31 |
| KRT79 | 2.195946896 | 0.676865312 | 6.99E-42 | 2.27E-40 |
| PZP | 2.194854354 | 1.636671916 | 1.13E-19 | 8.11E-19 |
| RNF182 | 2.191117081 | 2.72120164 | 2.18E-37 | 5.19E-36 |
| ATP1A2 | 2.189160609 | 3.201569337 | 2.71E-29 | 3.80E-28 |
| DMBT1 | 2.187901847 | 6.836370056 | 7.16E-12 | 2.88E-11 |
| SEMA3G | 2.187541655 | 5.117931338 | 1.06E-71 | 2.99E-69 |
| FBN3 | 2.185767927 | 2.799913794 | 2.24E-16 | 1.27E-15 |
| NPR1 | 2.184428189 | 5.362627407 | 8.50E-55 | 6.19E-53 |
| STOML3 | 2.183554861 | 2.033010647 | 1.25E-16 | 7.21E-16 |
| CCRL1 | 2.182676621 | 3.620549688 | 5.46E-50 | 2.91E-48 |
| MFAP4 | 2.176261911 | 7.296697552 | 1.53E-42 | 5.11E-41 |
| C6 | 2.175548687 | 2.950896073 | 2.82E-16 | 1.59E-15 |
| CHST9 | 2.17384044 | 2.855037306 | 6.72E-12 | 2.70E-11 |
| ANKRD29 | 2.170619748 | 4.9176893 | 6.55E-34 | 1.25E-32 |
| MAP3K15 | 2.170363924 | 1.215198769 | 9.78E-28 | 1.24E-26 |
| EFCAB1 | 2.168953304 | 3.136282512 | 3.45E-17 | 2.06E-16 |
| CXCR1 | 2.168592132 | 2.478644395 | 2.53E-33 | 4.65E-32 |
| ABI3BP | 2.166074046 | 5.969867013 | 4.87E-41 | 1.49E-39 |
| DCDC2B | 2.165242187 | 1.370422301 | 1.11E-18 | 7.45E-18 |
| HBG1 | 2.16500035 | 0.491560757 | 3.40E-45 | 1.36E-43 |
| GSTA3 | 2.164742077 | 0.644879229 | 1.94E-37 | 4.65E-36 |
| C1orf92 | 2.161268704 | 1.435561756 | 6.06E-18 | 3.84E-17 |
| LOC100287718 | 2.156139374 | 1.157411853 | 1.66E-21 | 1.36E-20 |
| C9orf24 | 2.154739502 | 3.075453962 | 8.92E-17 | 5.17E-16 |
| HPSE2 | 2.154371707 | 1.049719838 | 4.47E-39 | 1.19E-37 |
| VSIG2 | 2.152963443 | 5.221515458 | 4.20E-17 | 2.49E-16 |
| VWA3A | 2.150764028 | 2.230059578 | 2.62E-17 | 1.59E-16 |
| ABCC13 | 2.14962445 | 1.007886723 | 5.13E-34 | 9.91E-33 |
| WDR16 | 2.146857214 | 2.292717044 | 3.40E-17 | 2.03E-16 |
| TUBA4B | 2.141085372 | 2.191395986 | 1.23E-16 | 7.07E-16 |
| GPC3 | 2.132786284 | 5.698064171 | 3.31E-34 | 6.48E-33 |
| KCNE1 | 2.130514209 | 2.990853559 | 4.26E-27 | 5.16E-26 |
| ERVFRDE1 | 2.130339352 | 1.245984284 | 1.10E-48 | 5.31E-47 |
| RADIL | 2.129703157 | 3.552493637 | 1.29E-39 | 3.60E-38 |

| | | | | |
|---|---|---|---|---|
| CDHR3 | 2.124931778 | 4.126360977 | 9.56E-15 | 4.81E-14 |
| CACNA1S | 2.123939949 | 0.261410569 | 5.49E-61 | 6.55E-59 |
| CES4 | 2.123127533 | 2.832861928 | 4.64E-21 | 3.68E-20 |
| CWH43 | 2.122243792 | 1.079784573 | 1.83E-21 | 1.49E-20 |
| EMCN | 2.120700529 | 5.400310341 | 7.48E-60 | 8.04E-58 |
| TGM1 | 2.120574282 | 3.265256912 | 2.55E-54 | 1.81E-52 |
| CHRM2 | 2.117011216 | 0.310126389 | 5.68E-70 | 1.39E-67 |
| RIMS4 | 2.113563858 | 2.552945235 | 6.47E-25 | 6.66E-24 |
| OSTBETA | 2.112623417 | 1.725084048 | 1.04E-21 | 8.63E-21 |
| TMEM139 | 2.112108877 | 4.308980985 | 1.78E-25 | 1.92E-24 |
| C9orf171 | 2.110868776 | 1.903185345 | 2.11E-16 | 1.20E-15 |
| SEMA5A | 2.110350102 | 5.946347425 | 6.88E-54 | 4.69E-52 |
| HSPB6 | 2.109244655 | 5.247497043 | 3.14E-46 | 1.34E-44 |
| PIP5K1B | 2.108512408 | 4.677116952 | 1.58E-39 | 4.37E-38 |
| MT1M | 2.107763049 | 3.57599377 | 4.97E-29 | 6.87E-28 |
| MS4A8B | 2.107484357 | 3.293844569 | 3.75E-11 | 1.41E-10 |
| NTRK2 | 2.107465188 | 3.976746985 | 1.41E-26 | 1.65E-25 |
| CAPSL | 2.106347344 | 2.226283352 | 3.68E-14 | 1.77E-13 |
| C14orf132 | 2.105300184 | 5.691363624 | 1.38E-59 | 1.40E-57 |
| SYNPO2L | 2.103039698 | 1.559535559 | 7.11E-42 | 2.30E-40 |
| TGFBR3 | 2.102978 | 5.610294938 | 4.75E-58 | 4.43E-56 |
| CNKSR2 | 2.096845017 | 1.77430588 | 3.01E-32 | 5.18E-31 |
| ITGA8 | 2.096618267 | 4.212366903 | 4.14E-28 | 5.35E-27 |
| LRRC2 | 2.095644794 | 2.626132385 | 6.08E-42 | 1.98E-40 |
| RGS9 | 2.092335507 | 2.822550456 | 1.84E-48 | 8.77E-47 |
| JPH4 | 2.091769306 | 3.001982195 | 8.29E-58 | 7.63E-56 |
| HIGD1B | 2.091184042 | 3.441092597 | 1.36E-50 | 7.69E-49 |
| C15orf26 | 2.090206938 | 1.372576233 | 4.55E-24 | 4.43E-23 |
| C6orf155 | 2.088849615 | 1.902342618 | 5.25E-48 | 2.44E-46 |
| ADAMTSL3 | 2.087856171 | 4.555428571 | 5.45E-43 | 1.87E-41 |
| SGEF | 2.086845613 | 5.659223921 | 4.67E-67 | 9.14E-65 |
| DUOXA1 | 2.084253116 | 4.803047014 | 1.88E-25 | 2.02E-24 |
| TAL1 | 2.082337488 | 3.924710369 | 1.02E-74 | 3.61E-72 |
| SOX7 | 2.081869377 | 4.821734283 | 1.30E-60 | 1.50E-58 |
| GLDN | 2.078182779 | 4.5284717 | 6.45E-39 | 1.69E-37 |
| CASP12 | 2.076710163 | 0.714657138 | 3.01E-53 | 1.95E-51 |
| CCDC48 | 2.076399787 | 4.182601942 | 1.37E-50 | 7.73E-49 |
| ART4 | 2.075624807 | 1.963161124 | 2.18E-34 | 4.32E-33 |
| PKNOX2 | 2.072934012 | 3.95604987 | 2.63E-43 | 9.21E-42 |
| SCGB3A1 | 2.071497271 | 6.568940025 | 7.49E-10 | 2.52E-09 |
| NMUR1 | 2.070632756 | 2.922489464 | 1.77E-50 | 9.92E-49 |
| APOA1 | 2.07005628 | 1.079339324 | 8.97E-34 | 1.70E-32 |
| OLR1 | 2.069231108 | 6.239781531 | 1.00E-41 | 3.22E-40 |
| RGS22 | 2.06881278 | 2.478865963 | 1.59E-21 | 1.30E-20 |
| PCDH9 | 2.067844783 | 3.254595592 | 4.84E-31 | 7.67E-30 |
| PRSS35 | 2.063070183 | 2.260047912 | 2.05E-38 | 5.25E-37 |

| | | | | |
|---|---|---|---|---|
| LIMS2 | 2.062823391 | 5.789071035 | 6.76E-77 | 3.15E-74 |
| CA3 | 2.062568393 | 3.377578348 | 3.10E-25 | 3.28E-24 |
| TTC29 | 2.059990637 | 1.461290267 | 4.59E-16 | 2.54E-15 |
| MRC1 | 2.05998662 | 7.082495101 | 5.68E-36 | 1.25E-34 |
| WDR38 | 2.059767395 | 2.316035396 | 2.95E-13 | 1.32E-12 |
| C6orf174 | 2.059736313 | 4.486888117 | 5.02E-36 | 1.11E-34 |
| C13orf15 | 2.059518561 | 6.838068744 | 9.07E-84 | 8.87E-81 |
| TPPP3 | 2.056156655 | 6.376262071 | 1.71E-35 | 3.68E-34 |
| MORN5 | 2.05139628 | 1.779382767 | 9.71E-16 | 5.26E-15 |
| RAMP2 | 2.047355479 | 5.647307612 | 8.33E-84 | 8.55E-81 |
| GPR120 | 2.046738266 | 2.429749718 | 4.21E-30 | 6.23E-29 |
| KANK3 | 2.045871917 | 4.6801662 | 7.73E-79 | 4.41E-76 |
| S1PR1 | 2.045435465 | 6.239537453 | 7.77E-83 | 6.65E-80 |
| AOC3 | 2.043288601 | 7.065938183 | 5.64E-55 | 4.21E-53 |
| CCL14 | 2.041945641 | 4.956112122 | 1.27E-30 | 1.94E-29 |
| CLDN5 | 2.038666466 | 6.127298516 | 1.12E-54 | 8.13E-53 |
| PPP4R4 | 2.037900181 | 2.571250048 | 9.62E-25 | 9.79E-24 |
| SEMA6A | 2.037268022 | 4.968810397 | 3.58E-61 | 4.33E-59 |
| ROBO4 | 2.036145056 | 6.084417212 | 1.15E-76 | 5.14E-74 |
| CALCRL | 2.035721369 | 6.414002814 | 2.85E-71 | 7.92E-69 |
| HSPA12B | 2.034463541 | 4.743316754 | 2.75E-76 | 1.08E-73 |
| SLC5A7 | 2.032643681 | 0.756679929 | 1.01E-29 | 1.46E-28 |
| P2RX2 | 2.03094731 | 1.029533988 | 3.84E-30 | 5.71E-29 |
| DARC | 2.028004504 | 5.152127834 | 2.91E-24 | 2.87E-23 |
| SLIT2 | 2.026936868 | 5.423766319 | 6.29E-40 | 1.77E-38 |
| EMP2 | 2.02362943 | 8.599805773 | 9.29E-82 | 7.07E-79 |
| CXCR2 | 2.02181755 | 3.295048793 | 3.53E-39 | 9.50E-38 |
| STAC | 2.021220703 | 4.500495768 | 1.18E-27 | 1.48E-26 |
| AKAP2 | 2.020368483 | 6.643591543 | 2.87E-65 | 4.83E-63 |
| SIGLEC11 | 2.018799465 | 2.855420668 | 8.11E-40 | 2.28E-38 |
| LEPREL1 | 2.016491899 | 5.909060703 | 1.69E-28 | 2.25E-27 |
| BAI3 | 2.015978857 | 1.958702405 | 7.13E-31 | 1.11E-29 |
| C6orf118 | 2.015581866 | 1.593589729 | 1.90E-16 | 1.08E-15 |
| WFIKKN2 | 2.013896114 | 1.027551728 | 9.91E-33 | 1.76E-31 |
| TMEM232 | 2.010855109 | 2.167423603 | 8.97E-21 | 6.98E-20 |
| C1orf158 | 2.010436515 | 1.374715862 | 9.67E-18 | 6.03E-17 |
| C1orf129 | 2.009954714 | 0.872668424 | 1.79E-25 | 1.93E-24 |
| PACRG | 2.006752706 | 1.997466095 | 4.42E-21 | 3.50E-20 |
| SLC4A1 | 2.006510579 | 0.426022174 | 3.93E-53 | 2.54E-51 |
| HSPB3 | 2.005596076 | 0.995330314 | 2.85E-40 | 8.23E-39 |
| PTPRB | 2.00219304 | 6.635759182 | 3.96E-69 | 9.03E-67 |
| GSTM5 | 2.001210975 | 3.963225625 | 1.69E-46 | 7.28E-45 |
| CST5 | 2.000310288 | 1.388611781 | 1.16E-25 | 1.27E-24 |
| KIAA0101 | -2.006068106 | 5.342095532 | 5.60E-56 | 4.54E-54 |
| KCNN4 | -2.006857052 | 6.263026874 | 1.27E-29 | 1.82E-28 |
| F12 | -2.007376802 | 4.000225634 | 3.56E-40 | 1.02E-38 |

| | | | | |
|---|---|---|---|---|
| RDM1 | -2.00889929 | 1.840574183 | 5.01E-34 | 9.72E-33 |
| ORC1L | -2.014553457 | 4.440010635 | 5.11E-51 | 2.94E-49 |
| GPX2 | -2.018615777 | 4.895524069 | 1.07E-07 | 2.98E-07 |
| SPC25 | -2.018781275 | 4.03724049 | 3.38E-50 | 1.84E-48 |
| DUSP9 | -2.018875407 | 2.203221901 | 2.52E-16 | 1.43E-15 |
| SPC24 | -2.020179773 | 2.467997294 | 4.06E-41 | 1.25E-39 |
| HAVCR1 | -2.02155165 | 1.807714104 | 1.99E-13 | 9.09E-13 |
| GPT2 | -2.025942966 | 6.704706532 | 8.69E-53 | 5.47E-51 |
| AGMAT | -2.032980471 | 3.518093538 | 3.01E-50 | 1.67E-48 |
| C11orf86 | -2.034800376 | 1.759973754 | 3.88E-15 | 2.01E-14 |
| SLC22A18AS | -2.035476665 | 3.717419744 | 6.59E-35 | 1.35E-33 |
| MAST1 | -2.035506598 | 2.820085785 | 2.14E-33 | 3.95E-32 |
| UNC5CL | -2.047463825 | 5.249924344 | 2.17E-43 | 7.68E-42 |
| BCL2L15 | -2.050701166 | 4.982831065 | 3.28E-28 | 4.27E-27 |
| BAIAP2L2 | -2.052915488 | 3.298628291 | 2.78E-21 | 2.24E-20 |
| MAP7D2 | -2.058092148 | 4.006632911 | 1.11E-19 | 8.01E-19 |
| PPP2R2C | -2.058449436 | 3.272701 | 5.49E-11 | 2.03E-10 |
| PRSS3 | -2.063780208 | 2.235637771 | 2.36E-13 | 1.07E-12 |
| CDCA2 | -2.066788519 | 4.223742213 | 4.98E-50 | 2.67E-48 |
| ADAMTS16 | -2.066819386 | 3.902052534 | 1.12E-25 | 1.23E-24 |
| KLK6 | -2.068327185 | 2.004045633 | 2.55E-10 | 8.92E-10 |
| ERCC6L | -2.069370917 | 4.109705732 | 3.50E-63 | 5.10E-61 |
| CHRNA5 | -2.071248018 | 4.195040405 | 3.34E-33 | 6.10E-32 |
| RHBDL1 | -2.074383793 | 3.911376668 | 2.46E-35 | 5.18E-34 |
| CDCA8 | -2.077125514 | 5.549336243 | 1.79E-63 | 2.69E-61 |
| FERMT1 | -2.07992592 | 5.854684353 | 2.14E-51 | 1.27E-49 |
| MUC16 | -2.080871701 | 5.118914419 | 8.16E-09 | 2.51E-08 |
| CDCA7 | -2.085778431 | 5.662451182 | 2.31E-43 | 8.16E-42 |
| SLC2A1 | -2.086990398 | 7.554724928 | 1.04E-44 | 3.95E-43 |
| IL31RA | -2.094080742 | 2.469455327 | 6.29E-17 | 3.68E-16 |
| HMGB3 | -2.095122175 | 8.082228208 | 5.07E-48 | 2.36E-46 |
| HMGA2 | -2.096617124 | 3.013509344 | 3.04E-10 | 1.06E-09 |
| ECEL1 | -2.102663172 | 2.301088703 | 6.47E-16 | 3.54E-15 |
| PROC | -2.103393832 | 3.008663303 | 9.86E-22 | 8.23E-21 |
| COMP | -2.104230163 | 5.599877491 | 5.14E-18 | 3.28E-17 |
| KIF15 | -2.10442948 | 4.445100849 | 4.05E-49 | 2.04E-47 |
| TNFSF11 | -2.105091216 | 2.674793674 | 7.19E-24 | 6.87E-23 |
| HOXC10 | -2.107897178 | 2.176722266 | 1.21E-09 | 4.00E-09 |
| S100P | -2.11163892 | 6.56436211 | 1.24E-11 | 4.86E-11 |
| CCDC150 | -2.112199969 | 2.859161818 | 1.30E-42 | 4.37E-41 |
| SPAG4 | -2.112793793 | 4.935444109 | 8.53E-63 | 1.20E-60 |
| CELSR3 | -2.116497439 | 5.160952434 | 2.96E-44 | 1.10E-42 |
| TFR2 | -2.117276382 | 3.416245094 | 2.73E-49 | 1.39E-47 |
| RHOV | -2.119633919 | 5.329476433 | 1.45E-27 | 1.81E-26 |
| COL7A1 | -2.11990384 | 4.706550741 | 1.28E-20 | 9.83E-20 |
| CCNE1 | -2.121028893 | 4.511823667 | 1.53E-48 | 7.35E-47 |

| | | | | |
|---|---|---|---|---|
| TMEM184A | -2.124229234 | 6.513699415 | 1.10E-63 | 1.70E-61 |
| CKAP2L | -2.125104653 | 4.560105413 | 1.57E-49 | 8.16E-48 |
| LOC399815 | -2.128748052 | 2.203007019 | 4.68E-46 | 1.96E-44 |
| GPR87 | -2.130354482 | 3.532741789 | 1.10E-11 | 4.34E-11 |
| DLL3 | -2.130923075 | 1.910103262 | 9.05E-14 | 4.23E-13 |
| SRPK3 | -2.130934243 | 3.609280491 | 1.02E-24 | 1.04E-23 |
| CDKN3 | -2.133871795 | 4.50488929 | 6.93E-45 | 2.70E-43 |
| GJB6 | -2.137164765 | 2.394883355 | 5.25E-15 | 2.68E-14 |
| RRM2 | -2.140187305 | 6.469685189 | 6.89E-54 | 4.69E-52 |
| CENPI | -2.150558734 | 3.395275148 | 2.73E-40 | 7.92E-39 |
| NCAPH | -2.150973305 | 5.121451133 | 5.77E-54 | 3.96E-52 |
| KIF26B | -2.151927266 | 5.680347093 | 3.43E-57 | 3.03E-55 |
| ORC6L | -2.153901708 | 4.530925203 | 1.24E-65 | 2.14E-63 |
| CEP55 | -2.159195659 | 5.60165766 | 7.55E-56 | 6.01E-54 |
| IL1F5 | -2.161366404 | 2.016882724 | 6.07E-15 | 3.09E-14 |
| TBX15 | -2.161576764 | 4.318528616 | 5.07E-25 | 5.25E-24 |
| NGEF | -2.162344933 | 4.379699438 | 1.96E-25 | 2.10E-24 |
| IL22RA2 | -2.165040012 | 2.238992739 | 6.82E-24 | 6.53E-23 |
| SPAG5 | -2.168792054 | 6.047109472 | 1.03E-65 | 1.81E-63 |
| CBLC | -2.173625036 | 5.340287302 | 4.81E-45 | 1.90E-43 |
| FUT2 | -2.173788279 | 5.66397035 | 8.83E-54 | 5.95E-52 |
| RECQL4 | -2.175434857 | 5.574068943 | 7.84E-62 | 1.02E-59 |
| HMMR | -2.18011568 | 5.16347788 | 5.24E-56 | 4.27E-54 |
| POLQ | -2.181524766 | 4.141628869 | 4.23E-49 | 2.13E-47 |
| CENPF | -2.190507207 | 6.451308798 | 1.47E-56 | 1.24E-54 |
| CP | -2.194854654 | 8.287007351 | 1.25E-25 | 1.36E-24 |
| FEZF1 | -2.195296331 | 2.106026605 | 4.08E-16 | 2.27E-15 |
| PLXNB3 | -2.199619249 | 5.517113597 | 8.69E-26 | 9.65E-25 |
| GLB1L3 | -2.205513636 | 2.806342928 | 8.55E-10 | 2.86E-09 |
| LYPD1 | -2.20784223 | 4.754066494 | 2.01E-35 | 4.27E-34 |
| FAM64A | -2.210254097 | 4.156037735 | 4.33E-46 | 1.82E-44 |
| PKMYT1 | -2.211936879 | 5.004481042 | 1.19E-55 | 9.38E-54 |
| GTSE1 | -2.215012935 | 4.841771155 | 9.06E-59 | 8.86E-57 |
| GALNT14 | -2.222616395 | 5.040076071 | 9.04E-26 | 1.00E-24 |
| DMBX1 | -2.223037879 | 1.951922686 | 5.63E-21 | 4.44E-20 |
| NCAPG | -2.227142828 | 5.369764782 | 4.24E-57 | 3.72E-55 |
| CCNB2 | -2.229881192 | 5.466527465 | 1.48E-60 | 1.68E-58 |
| FOXM1 | -2.237715819 | 6.201636023 | 1.62E-53 | 1.07E-51 |
| EPR1 | -2.240034905 | 5.459382069 | 1.91E-45 | 7.75E-44 |
| IL1F7 | -2.24183911 | 2.118687601 | 6.47E-12 | 2.61E-11 |
| DNAJC22 | -2.246655938 | 3.618628946 | 9.34E-32 | 1.56E-30 |
| PSAT1 | -2.250161195 | 5.758532166 | 6.85E-48 | 3.17E-46 |
| ESPL1 | -2.256052196 | 5.029163951 | 9.38E-50 | 4.94E-48 |
| SKA3 | -2.257086083 | 4.403614905 | 2.02E-58 | 1.94E-56 |
| DGCR5 | -2.261705802 | 4.149482159 | 2.94E-31 | 4.76E-30 |
| IGFL2 | -2.273285701 | 2.39094451 | 5.57E-22 | 4.72E-21 |

| | | | | |
|---|---|---|---|---|
| MB | -2.282823118 | 4.261668458 | 1.72E-27 | 2.13E-26 |
| ABP1 | -2.284632817 | 5.019347387 | 4.71E-16 | 2.61E-15 |
| HOXB9 | -2.28688439 | 2.286458334 | 4.85E-11 | 1.80E-10 |
| CXorf61 | -2.288826942 | 2.030188996 | 2.13E-12 | 8.92E-12 |
| PPAPDC1A | -2.289741148 | 2.461671029 | 2.40E-23 | 2.21E-22 |
| ZNF695 | -2.289959807 | 2.331066526 | 1.02E-25 | 1.12E-24 |
| UBE2T | -2.293800227 | 5.442629353 | 9.61E-73 | 2.95E-70 |
| PLK1 | -2.296746794 | 5.729785235 | 3.81E-64 | 6.01E-62 |
| CDCA3 | -2.297733251 | 4.71494055 | 2.05E-61 | 2.55E-59 |
| TMEM63C | -2.299353269 | 4.261438239 | 3.15E-21 | 2.53E-20 |
| NDC80 | -2.3031415 | 4.830401941 | 4.04E-55 | 3.04E-53 |
| WFDC3 | -2.304596014 | 3.854368903 | 1.91E-24 | 1.90E-23 |
| CDC6 | -2.312581816 | 5.529077531 | 1.62E-61 | 2.06E-59 |
| ALG1L | -2.318242529 | 4.2242087 | 1.00E-30 | 1.54E-29 |
| SALL4 | -2.319893717 | 3.150264342 | 3.70E-43 | 1.28E-41 |
| COL17A1 | -2.324579436 | 5.213457151 | 9.10E-16 | 4.94E-15 |
| SGOL1 | -2.345278262 | 3.659429076 | 5.17E-56 | 4.23E-54 |
| LY6D | -2.350671445 | 2.531136161 | 1.35E-13 | 6.24E-13 |
| PBK | -2.363742753 | 4.609071573 | 9.10E-49 | 4.44E-47 |
| EPHA10 | -2.364092648 | 4.714699572 | 2.06E-35 | 4.37E-34 |
| DNAJC12 | -2.366582955 | 5.002640964 | 1.09E-25 | 1.19E-24 |
| HNF4G | -2.369062899 | 4.27097214 | 3.29E-34 | 6.44E-33 |
| BARX2 | -2.372841195 | 4.205618424 | 1.58E-25 | 1.71E-24 |
| ADM2 | -2.387381611 | 5.391345993 | 7.06E-74 | 2.41E-71 |
| KIF20A | -2.391106217 | 5.479956383 | 1.01E-71 | 2.92E-69 |
| PCP4 | -2.391431047 | 3.226414597 | 6.11E-14 | 2.90E-13 |
| KRT6A | -2.39492689 | 4.017047348 | 3.24E-10 | 1.13E-09 |
| GAD1 | -2.398175973 | 2.880850153 | 1.05E-25 | 1.15E-24 |
| TTK | -2.400073635 | 4.637559713 | 8.73E-52 | 5.33E-50 |
| IGSF9 | -2.400691433 | 5.725018375 | 3.96E-56 | 3.27E-54 |
| UHRF1 | -2.401085656 | 5.246188829 | 2.53E-69 | 5.90E-67 |
| CENPA | -2.402054299 | 4.168594658 | 1.64E-50 | 9.24E-49 |
| RAD54L | -2.406931977 | 4.422455688 | 4.57E-61 | 5.49E-59 |
| TPX2 | -2.409652786 | 6.442514406 | 6.88E-58 | 6.36E-56 |
| CABYR | -2.409828334 | 4.25356655 | 9.72E-28 | 1.23E-26 |
| CGREF1 | -2.410487609 | 3.887410116 | 4.97E-30 | 7.33E-29 |
| ASPM | -2.410492823 | 5.453081007 | 4.06E-60 | 4.49E-58 |
| LGSN | -2.412644489 | 4.449709062 | 2.82E-15 | 1.48E-14 |
| PLEKHN1 | -2.412751082 | 4.351258502 | 1.74E-61 | 2.20E-59 |
| EPN3 | -2.416854868 | 5.472560281 | 8.22E-55 | 6.00E-53 |
| KISS1R | -2.420734112 | 2.089517428 | 9.07E-27 | 1.07E-25 |
| HS6ST2 | -2.43168624 | 6.083153122 | 2.96E-39 | 8.02E-38 |
| RGS17 | -2.434702972 | 3.209155937 | 2.60E-33 | 4.78E-32 |
| ONECUT2 | -2.441117563 | 2.938497139 | 2.36E-23 | 2.18E-22 |
| E2F8 | -2.444130114 | 4.515114794 | 1.04E-71 | 2.97E-69 |
| C9orf140 | -2.449042158 | 5.510708508 | 1.80E-72 | 5.42E-70 |

| | | | | |
|---|---|---|---|---|
| MCM10 | -2.44992112 | 4.479937317 | 2.97E-51 | 1.74E-49 |
| FGF11 | -2.451205788 | 4.770322835 | 4.23E-54 | 2.93E-52 |
| CEACAM5 | -2.457826901 | 7.98485659 | 1.30E-14 | 6.47E-14 |
| CILP2 | -2.47057945 | 4.710208001 | 7.42E-41 | 2.23E-39 |
| TCN1 | -2.474377435 | 3.002546338 | 1.74E-11 | 6.74E-11 |
| RAB26 | -2.478223562 | 3.356099715 | 2.82E-48 | 1.33E-46 |
| CNTD2 | -2.486912642 | 3.115181907 | 4.72E-30 | 6.96E-29 |
| ANLN | -2.488502109 | 6.189234337 | 8.29E-60 | 8.82E-58 |
| TFF1 | -2.499991699 | 2.45381171 | 1.42E-10 | 5.06E-10 |
| DEPDC1 | -2.505179585 | 4.758350474 | 6.42E-56 | 5.13E-54 |
| EXO1 | -2.506990021 | 4.550430962 | 1.11E-59 | 1.15E-57 |
| BUB1B | -2.51678771 | 5.176475366 | 1.10E-68 | 2.46E-66 |
| DEPDC1B | -2.520637293 | 4.407496351 | 1.46E-60 | 1.68E-58 |
| YBX2 | -2.523564918 | 2.838929081 | 3.78E-20 | 2.81E-19 |
| XDH | -2.526182452 | 5.008856622 | 3.39E-36 | 7.60E-35 |
| MNX1 | -2.539712658 | 2.753401753 | 1.43E-33 | 2.68E-32 |
| AURKB | -2.541790484 | 4.874240175 | 2.63E-59 | 2.65E-57 |
| KIF14 | -2.544398705 | 4.593140005 | 6.45E-61 | 7.65E-59 |
| KIF18B | -2.545145405 | 4.872967257 | 3.53E-58 | 3.34E-56 |
| PYCR1 | -2.54963416 | 7.607459183 | 4.35E-110 | 8.94E-106 |
| C2CD4A | -2.550232092 | 4.689684776 | 2.34E-35 | 4.95E-34 |
| OTX1 | -2.559501152 | 3.607226371 | 2.57E-67 | 5.13E-65 |
| NEK2 | -2.563659931 | 5.148807027 | 2.47E-64 | 3.97E-62 |
| GPR115 | -2.567548665 | 3.317974271 | 3.28E-23 | 3.00E-22 |
| MUC13 | -2.574336896 | 4.14579405 | 2.81E-11 | 1.07E-10 |
| MEX3A | -2.58644671 | 5.724850915 | 1.09E-58 | 1.06E-56 |
| AKR7A3 | -2.597385926 | 3.689398424 | 9.42E-22 | 7.88E-21 |
| TOP2A | -2.603588505 | 7.224343128 | 7.71E-71 | 2.06E-68 |
| GRHL3 | -2.612713658 | 3.136370819 | 1.97E-40 | 5.77E-39 |
| C16orf59 | -2.615580817 | 4.321952156 | 7.90E-91 | 2.32E-87 |
| HJURP | -2.615719052 | 5.127953634 | 1.49E-64 | 2.48E-62 |
| TFAP2A | -2.619541242 | 4.838130696 | 5.94E-44 | 2.18E-42 |
| IQGAP3 | -2.619865651 | 5.826262426 | 4.63E-76 | 1.79E-73 |
| MND1 | -2.621360727 | 3.47752998 | 2.94E-77 | 1.47E-74 |
| ETV4 | -2.63214237 | 6.339476096 | 7.22E-71 | 1.95E-68 |
| MELK | -2.639423784 | 5.057195635 | 6.04E-60 | 6.52E-58 |
| CXCL13 | -2.639546525 | 5.506618197 | 1.84E-29 | 2.61E-28 |
| CTHRC1 | -2.640010084 | 6.656871574 | 1.28E-62 | 1.76E-60 |
| KIF2C | -2.649254816 | 5.521724836 | 1.16E-67 | 2.42E-65 |
| NEIL3 | -2.65293088 | 3.493331976 | 1.60E-46 | 6.88E-45 |
| CDC20 | -2.668653403 | 5.916760171 | 2.11E-69 | 4.99E-67 |
| TROAP | -2.681639119 | 4.87087224 | 1.10E-59 | 1.15E-57 |
| UBE2C | -2.711612731 | 5.842669247 | 1.68E-55 | 1.30E-53 |
| NMU | -2.71185887 | 2.925141508 | 7.84E-24 | 7.49E-23 |
| BIRC5 | -2.714403869 | 5.266369524 | 3.24E-61 | 3.96E-59 |
| GJB2 | -2.718750401 | 5.653837637 | 6.93E-45 | 2.70E-43 |

| | | | | |
|---|---|---|---|---|
| TUBB3 | -2.721046089 | 7.01185776 | 2.40E-61 | 2.97E-59 |
| FRMD5 | -2.729668317 | 3.50338214 | 2.58E-51 | 1.52E-49 |
| BARX1 | -2.738797148 | 2.679135106 | 1.83E-13 | 8.35E-13 |
| DLGAP5 | -2.74404439 | 5.121749104 | 7.99E-67 | 1.53E-64 |
| CDC45 | -2.74992894 | 4.628621345 | 8.03E-66 | 1.42E-63 |
| MYBL2 | -2.762183674 | 6.19062377 | 5.75E-55 | 4.28E-53 |
| NUF2 | -2.766524828 | 4.852452191 | 7.93E-68 | 1.70E-65 |
| HHIPL2 | -2.767398411 | 2.897113727 | 1.22E-18 | 8.12E-18 |
| FGB | -2.80538272 | 3.385251381 | 2.64E-09 | 8.48E-09 |
| LOC100131726 | -2.809906098 | 2.441043385 | 1.45E-46 | 6.33E-45 |
| GCNT3 | -2.843299071 | 5.478449815 | 1.09E-32 | 1.93E-31 |
| KIF4A | -2.847595724 | 5.314585562 | 5.86E-68 | 1.27E-65 |
| ENTPD8 | -2.849410011 | 3.327787125 | 1.50E-37 | 3.63E-36 |
| TRPM8 | -2.875797209 | 2.758497099 | 1.14E-23 | 1.08E-22 |
| B4GALNT4 | -2.897010259 | 3.855115417 | 5.07E-26 | 5.72E-25 |
| CDC25C | -2.898891592 | 3.758226045 | 8.96E-72 | 2.63E-69 |
| AKR1B10 | -2.901656967 | 3.42864685 | 3.38E-12 | 1.39E-11 |
| KRT16 | -2.916949007 | 3.303133811 | 3.60E-21 | 2.88E-20 |
| ITPKA | -2.927843285 | 4.121856431 | 8.73E-32 | 1.46E-30 |
| FER1L4 | -2.94743442 | 4.704068182 | 4.69E-45 | 1.86E-43 |
| RASAL1 | -2.959266063 | 4.357594556 | 1.34E-56 | 1.13E-54 |
| MMP12 | -2.974705414 | 5.157811794 | 6.57E-32 | 1.11E-30 |
| B3GNT3 | -2.974871198 | 6.137140936 | 4.20E-63 | 6.07E-61 |
| GREM1 | -2.987634456 | 6.005879029 | 6.03E-39 | 1.58E-37 |
| PAEP | -2.993611366 | 3.602576488 | 5.19E-13 | 2.29E-12 |
| MYEOV | -2.994208113 | 3.899594736 | 1.09E-24 | 1.10E-23 |
| ZYG11A | -3.005269787 | 2.803644749 | 2.15E-35 | 4.56E-34 |
| VIL1 | -3.026545622 | 2.904646708 | 6.11E-15 | 3.11E-14 |
| XAGE1D | -3.038586133 | 5.785284216 | 4.17E-13 | 1.85E-12 |
| STRA6 | -3.040757701 | 4.384992655 | 1.08E-36 | 2.49E-35 |
| EEF1A2 | -3.067652711 | 4.87963894 | 7.13E-19 | 4.85E-18 |
| FGL1 | -3.112236167 | 3.639711642 | 2.33E-16 | 1.32E-15 |
| B3GNT6 | -3.205374131 | 3.793442973 | 3.52E-25 | 3.71E-24 |
| PITX1 | -3.209124846 | 4.427744894 | 5.82E-35 | 1.20E-33 |
| HTR3A | -3.21770986 | 3.304830343 | 5.86E-32 | 9.92E-31 |
| CRABP2 | -3.233362637 | 6.965002276 | 4.81E-41 | 1.47E-39 |
| 729884 | -3.242338298 | 3.383742985 | 3.46E-24 | 3.40E-23 |
| PITX2 | -3.243498758 | 2.891941112 | 1.70E-25 | 1.84E-24 |
| COL10A1 | -3.26769159 | 6.158526362 | 1.13E-62 | 1.58E-60 |
| SPP1 | -3.297516687 | 8.297337444 | 2.75E-51 | 1.61E-49 |
| MMP1 | -3.326719556 | 5.926495777 | 1.86E-35 | 3.97E-34 |
| ATP10B | -3.353608223 | 4.238136977 | 6.39E-45 | 2.50E-43 |
| TMPRSS4 | -3.367464885 | 6.733025788 | 4.32E-49 | 2.17E-47 |
| PRAME | -3.390047121 | 3.351174535 | 1.41E-18 | 9.36E-18 |
| SPINK1 | -3.416930566 | 5.239855845 | 1.38E-19 | 9.85E-19 |

| | | | | |
|---|---|---|---|---|
| MMP13 | -3.565235681 | 4.219062484 | 5.64E-31 | 8.89E-30 |
| SYT12 | -3.566268979 | 4.840156825 | 1.89E-42 | 6.30E-41 |
| PTPRH | -3.603734334 | 4.480195882 | 5.46E-46 | 2.26E-44 |
| MMP11 | -3.617949569 | 6.667394795 | 1.82E-63 | 2.73E-61 |
| PPP1R14D | -3.665699079 | 3.663107375 | 1.22E-34 | 2.46E-33 |
| CA9 | -3.66898258 | 4.041996856 | 1.09E-29 | 1.56E-28 |
| LOC84740 | -3.69592949 | 5.876941277 | 5.99E-33 | 1.07E-31 |
| CYP24A1 | -3.783972773 | 5.374661118 | 6.24E-35 | 1.28E-33 |
| ABCA12 | -3.858281793 | 3.97638025 | 5.54E-43 | 1.90E-41 |
| COL11A1 | -4.273838013 | 5.173444892 | 3.39E-40 | 9.75E-39 |
| CST1 | -4.28927857 | 4.348862315 | 4.81E-41 | 1.47E-39 |
| FAM83A | -4.41531909 | 6.546931671 | 3.23E-77 | 1.58E-74 |

Table Supplementary 6: KEGG and GO analysis of hub driving genes

| ID | Description | GeneRatio | BgRatio | P-value | Count |
|---|---|---|---|---|---|
| GO:0000226 | microtubule cytoskeleton organization | 170/3478 | 443/16992 | 1.41E-18 | 170 |
| GO:0060271 | cilium assembly | 136/3478 | 335/16992 | 1.80E-17 | 136 |
| GO:0044782 | cilium organization | 138/3478 | 343/16992 | 2.58E-17 | 138 |
| GO:0048193 | Golgi vesicle transport | 130/3478 | 334/16992 | 4.69E-15 | 130 |
| GO:0007264 | small GTPase mediated signal transduction | 168/3478 | 490/16992 | 3.52E-13 | 168 |
| GO:0007067 | mitotic nuclear division | 158/3478 | 453/16992 | 3.67E-13 | 158 |
| GO:0007030 | Golgi organization | 53/3478 | 101/16992 | 9.84E-13 | 53 |
| GO:0007059 | chromosome segregation | 122/3478 | 330/16992 | 2.22E-12 | 122 |
| GO:0051056 | regulation of small GTPase mediated signal transduction | 113/3478 | 300/16992 | 3.60E-12 | 113 |
| GO:0007018 | microtubule-based movement | 99/3478 | 254/16992 | 7.79E-12 | 99 |
| GO:0022604 | regulation of cell morphogenesis | 145/3478 | 424/16992 | 1.84E-11 | 145 |
| GO:0006417 | regulation of translation | 121/3478 | 344/16992 | 1.17E-10 | 121 |
| GO:0016570 | histone modification | 144/3478 | 432/16992 | 1.72E-10 | 144 |
| GO:0034248 | regulation of cellular amide metabolic process | 130/3478 | 384/16992 | 4.33E-10 | 130 |
| GO:0051640 | organelle localization | 159/3478 | 496/16992 | 5.22E-10 | 159 |
| GO:0010608 | posttranscriptional regulation of gene expression | 152/3478 | 469/16992 | 5.31E-10 | 152 |
| GO:0000819 | sister chromatid segregation | 84/3478 | 218/16992 | 5.74E-10 | 84 |
| GO:0097711 | ciliary basal body docking | 47/3478 | 97/16992 | 6.48E-10 | 47 |
| GO:0031570 | DNA integrity checkpoint | 67/3478 | 161/16992 | 7.45E-10 | 67 |
| GO:1901990 | regulation of mitotic cell cycle phase transition | 111/3478 | 316/16992 | 7.47E-10 | 111 |
| GO:0016482 | cytosolic transport | 59/3478 | 136/16992 | 1.12E-09 | 59 |
| GO:1901987 | regulation of cell cycle phase transition | 116/3478 | 337/16992 | 1.24E-09 | 116 |
| GO:0000075 | cell cycle checkpoint | 84/3478 | 221/16992 | 1.25E-09 | 84 |
| GO:0000077 | DNA damage checkpoint | 63/3478 | 151/16992 | 2.10E-09 | 63 |
| GO:0031023 | microtubule organizing center organization | 53/3478 | 119/16992 | 2.51E-09 | 53 |
| GO:0098813 | nuclear chromosome segregation | 101/3478 | 287/16992 | 3.85E-09 | 101 |
| GO:0051052 | regulation of DNA metabolic process | 124/3478 | 376/16992 | 6.57E-09 | 124 |
| GO:0022406 | membrane docking | 68/3478 | 173/16992 | 9.49E-09 | 68 |
| GO:0000086 | G2/M transition of mitotic cell cycle | 85/3478 | 233/16992 | 9.76E-09 | 85 |
| GO:0007051 | spindle organization | 60/3478 | 146/16992 | 9.95E-09 | 60 |
| GO:0070646 | protein modification by small protein removal | 104/3478 | 304/16992 | 1.28E-08 | 104 |
| GO:0006892 | post-Golgi vesicle-mediated transport | 42/3478 | 89/16992 | 1.40E-08 | 42 |
| GO:0044839 | cell cycle G2/M phase transition | 87/3478 | 242/16992 | 1.46E-08 | 87 |
| GO:0000209 | protein polyubiquitination | 99/3478 | 287/16992 | 1.77E-08 | 99 |
| GO:0016197 | endosomal transport | 91/3478 | 260/16992 | 3.02E-08 | 91 |
| GO:1901991 | negative regulation of mitotic cell cycle phase transition | 74/3478 | 200/16992 | 4.41E-08 | 74 |
| GO:0045930 | negative regulation of mitotic cell cycle | 94/3478 | 274/16992 | 5.42E-08 | 94 |
| GO:0007507 | heart development | 152/3478 | 499/16992 | 5.59E-08 | 152 |
| GO:0009896 | positive regulation of catabolic process | 130/3478 | 412/16992 | 5.60E-08 | 130 |
| GO:0000289 | nuclear-transcribed mRNA poly(A) tail shortening | 22/3478 | 35/16992 | 5.89E-08 | 22 |
| GO:0006338 | chromatin remodeling | 62/3478 | 159/16992 | 5.94E-08 | 62 |
| GO:0007163 | establishment or maintenance of cell polarity | 64/3478 | 166/16992 | 6.05E-08 | 64 |
| GO:0018205 | peptidyl-lysine modification | 118/3478 | 366/16992 | 6.25E-08 | 118 |

| | | | | | |
|---|---|---|---|---|---|
| GO:0007265 | Ras protein signal transduction | 107/3478 | 324/16992 | 6.50E-08 | 107 |
| GO:0043161 | proteasome-mediated ubiquitin-dependent protein catabolic process | 121/3478 | 378/16992 | 6.66E-08 | 121 |
| GO:0051297 | centrosome organization | 48/3478 | 113/16992 | 8.60E-08 | 48 |
| GO:0051656 | establishment of organelle localization | 132/3478 | 426/16992 | 1.43E-07 | 132 |
| GO:0010498 | proteasomal protein catabolic process | 127/3478 | 407/16992 | 1.60E-07 | 127 |
| GO:0006260 | DNA replication | 97/3478 | 291/16992 | 1.61E-07 | 97 |
| GO:0010970 | transport along microtubule | 51/3478 | 125/16992 | 1.63E-07 | 51 |
| GO:0099111 | microtubule-based transport | 51/3478 | 125/16992 | 1.63E-07 | 51 |
| GO:0030705 | cytoskeleton-dependent intracellular transport | 56/3478 | 142/16992 | 1.63E-07 | 56 |
| GO:0044843 | cell cycle G1/S phase transition | 87/3478 | 254/16992 | 1.81E-07 | 87 |
| GO:0016579 | protein deubiquitination | 96/3478 | 289/16992 | 2.24E-07 | 96 |
| GO:0042787 | protein ubiquitination involved in ubiquitin-dependent protein catabolic process | 73/3478 | 204/16992 | 2.54E-07 | 73 |
| GO:0000082 | G1/S transition of mitotic cell cycle | 83/3478 | 241/16992 | 2.65E-07 | 83 |
| GO:0051650 | establishment of vesicle localization | 83/3478 | 241/16992 | 2.65E-07 | 83 |
| GO:1901988 | negative regulation of cell cycle phase transition | 74/3478 | 208/16992 | 2.75E-07 | 74 |
| GO:0007093 | mitotic cell cycle checkpoint | 58/3478 | 151/16992 | 2.87E-07 | 58 |
| GO:0051648 | vesicle localization | 87/3478 | 257/16992 | 3.25E-07 | 87 |
| GO:0032886 | regulation of microtubule-based process | 59/3478 | 155/16992 | 3.27E-07 | 59 |
| GO:0051298 | centrosome duplication | 31/3478 | 63/16992 | 3.34E-07 | 31 |
| GO:0007009 | plasma membrane organization | 92/3478 | 277/16992 | 4.00E-07 | 92 |
| GO:0007098 | centrosome cycle | 37/3478 | 82/16992 | 4.14E-07 | 37 |
| GO:0044774 | mitotic DNA integrity checkpoint | 44/3478 | 105/16992 | 4.65E-07 | 44 |
| GO:0010948 | negative regulation of cell cycle process | 90/3478 | 271/16992 | 5.34E-07 | 90 |
| GO:0006914 | autophagy | 135/3478 | 449/16992 | 6.88E-07 | 135 |
| GO:0010769 | regulation of cell morphogenesis involved in differentiation | 84/3478 | 250/16992 | 7.33E-07 | 84 |
| GO:0031331 | positive regulation of cellular catabolic process | 112/3478 | 358/16992 | 7.35E-07 | 112 |
| GO:0034332 | adherens junction organization | 48/3478 | 120/16992 | 7.48E-07 | 48 |
| GO:0010639 | negative regulation of organelle organization | 103/3478 | 323/16992 | 7.56E-07 | 103 |
| GO:0051225 | spindle assembly | 38/3478 | 87/16992 | 7.99E-07 | 38 |
| GO:0000070 | mitotic sister chromatid segregation | 52/3478 | 134/16992 | 8.02E-07 | 52 |
| GO:1902115 | regulation of organelle assembly | 55/3478 | 145/16992 | 9.20E-07 | 55 |
| GO:0072659 | protein localization to plasma membrane | 74/3478 | 214/16992 | 9.68E-07 | 74 |
| GO:0042770 | signal transduction in response to DNA damage | 48/3478 | 121/16992 | 9.95E-07 | 48 |
| GO:0046777 | protein autophosphorylation | 79/3478 | 233/16992 | 1.04E-06 | 79 |
| GO:0007052 | mitotic spindle organization | 39/3478 | 91/16992 | 1.04E-06 | 39 |
| GO:1902850 | microtubule cytoskeleton organization involved in mitosis | 39/3478 | 91/16992 | 1.04E-06 | 39 |
| GO:0006997 | nucleus organization | 54/3478 | 142/16992 | 1.05E-06 | 54 |
| GO:0090161 | Golgi ribbon formation | 10/3478 | 11/16992 | 1.14E-06 | 10 |
| GO:0000301 | retrograde transport, vesicle recycling within Golgi | 16/3478 | 24/16992 | 1.24E-06 | 16 |
| GO:0010506 | regulation of autophagy | 89/3478 | 272/16992 | 1.26E-06 | 89 |
| GO:0016358 | dendrite development | 69/3478 | 197/16992 | 1.31E-06 | 69 |
| GO:0033044 | regulation of chromosome organization | 93/3478 | 288/16992 | 1.40E-06 | 93 |
| GO:0038127 | ERBB signaling pathway | 55/3478 | 147/16992 | 1.52E-06 | 55 |
| GO:0007062 | sister chromatid cohesion | 49/3478 | 126/16992 | 1.53E-06 | 49 |

| | | | | | |
|---|---|---|---|---|---|
| GO:0010770 | positive regulation of cell morphogenesis involved in differentiation | 51/3478 | 133/16992 | 1.55E-06 | 51 |
| GO:0051169 | nuclear transport | 139/3478 | 472/16992 | 1.69E-06 | 139 |
| GO:0017148 | negative regulation of translation | 57/3478 | 155/16992 | 1.88E-06 | 57 |
| GO:0006312 | mitotic recombination | 25/3478 | 49/16992 | 1.94E-06 | 25 |
| GO:0030010 | establishment of cell polarity | 42/3478 | 103/16992 | 1.99E-06 | 42 |
| GO:0034333 | adherens junction assembly | 35/3478 | 80/16992 | 2.05E-06 | 35 |
| GO:0035329 | hippo signaling | 20/3478 | 35/16992 | 2.07E-06 | 20 |
| GO:0046578 | regulation of Ras protein signal transduction | 70/3478 | 203/16992 | 2.11E-06 | 70 |
| GO:0072331 | signal transduction by p53 class mediator | 85/3478 | 260/16992 | 2.25E-06 | 85 |
| GO:0006261 | DNA-dependent DNA replication | 52/3478 | 138/16992 | 2.27E-06 | 52 |
| GO:0051493 | regulation of cytoskeleton organization | 127/3478 | 426/16992 | 2.37E-06 | 127 |
| GO:0030330 | DNA damage response, signal transduction by p53 class mediator | 43/3478 | 107/16992 | 2.39E-06 | 43 |
| GO:0034249 | negative regulation of cellular amide metabolic process | 60/3478 | 167/16992 | 2.47E-06 | 60 |
| GO:0072384 | organelle transport along microtubule | 31/3478 | 68/16992 | 2.70E-06 | 31 |
| GO:0016050 | vesicle organization | 115/3478 | 379/16992 | 2.79E-06 | 115 |
| GO:0006913 | nucleocytoplasmic transport | 136/3478 | 464/16992 | 2.87E-06 | 136 |
| GO:0007077 | mitotic nuclear envelope disassembly | 23/3478 | 44/16992 | 2.90E-06 | 23 |
| GO:0018105 | peptidyl-serine phosphorylation | 89/3478 | 277/16992 | 2.95E-06 | 89 |
| GO:0034329 | cell junction assembly | 67/3478 | 194/16992 | 3.25E-06 | 67 |
| GO:0007033 | vacuole organization | 58/3478 | 161/16992 | 3.27E-06 | 58 |
| GO:0044773 | mitotic DNA damage checkpoint | 40/3478 | 98/16992 | 3.36E-06 | 40 |
| GO:1990778 | protein localization to cell periphery | 77/3478 | 232/16992 | 3.52E-06 | 77 |
| GO:0006998 | nuclear envelope organization | 36/3478 | 85/16992 | 3.71E-06 | 36 |
| GO:0008156 | negative regulation of DNA replication | 20/3478 | 36/16992 | 3.75E-06 | 20 |
| GO:0006403 | RNA localization | 75/3478 | 225/16992 | 3.92E-06 | 75 |
| GO:0034446 | substrate adhesion-dependent cell spreading | 35/3478 | 82/16992 | 4.08E-06 | 35 |
| GO:0042176 | regulation of protein catabolic process | 115/3478 | 382/16992 | 4.22E-06 | 115 |
| GO:0030397 | membrane disassembly | 24/3478 | 48/16992 | 4.92E-06 | 24 |
| GO:0051081 | nuclear envelope disassembly | 24/3478 | 48/16992 | 4.92E-06 | 24 |
| GO:0010827 | regulation of glucose transport | 41/3478 | 103/16992 | 5.40E-06 | 41 |
| GO:0006397 | mRNA processing | 138/3478 | 478/16992 | 5.82E-06 | 138 |
| GO:0009411 | response to UV | 48/3478 | 128/16992 | 6.39E-06 | 48 |
| GO:0010810 | regulation of cell-substrate adhesion | 62/3478 | 179/16992 | 6.68E-06 | 62 |
| GO:0031346 | positive regulation of cell projection organization | 98/3478 | 318/16992 | 7.23E-06 | 98 |
| GO:0044766 | multi-organism transport | 32/3478 | 74/16992 | 7.49E-06 | 32 |
| GO:0097581 | lamellipodium organization | 32/3478 | 74/16992 | 7.49E-06 | 32 |
| GO:1902579 | multi-organism localization | 32/3478 | 74/16992 | 7.49E-06 | 32 |
| GO:1905037 | autophagosome organization | 32/3478 | 74/16992 | 7.49E-06 | 32 |
| GO:0008360 | regulation of cell shape | 52/3478 | 143/16992 | 7.57E-06 | 52 |
| GO:0016577 | histone demethylation | 17/3478 | 29/16992 | 7.59E-06 | 17 |
| GO:0006470 | protein dephosphorylation | 81/3478 | 252/16992 | 7.92E-06 | 81 |
| GO:0000045 | autophagosome assembly | 31/3478 | 71/16992 | 8.15E-06 | 31 |
| GO:0000042 | protein targeting to Golgi | 13/3478 | 19/16992 | 8.40E-06 | 13 |
| GO:2000104 | negative regulation of DNA-dependent DNA replication | 13/3478 | 19/16992 | 8.40E-06 | 13 |
| GO:0006275 | regulation of DNA replication | 44/3478 | 115/16992 | 8.41E-06 | 44 |

| | | | | | |
|---|---|---|---|---|---|
| GO:0016925 | protein sumoylation | 37/3478 | 91/16992 | 8.59E-06 | 37 |
| GO:1902581 | multi-organism cellular localization | 30/3478 | 68/16992 | 8.82E-06 | 30 |
| GO:1902583 | multi-organism intracellular transport | 30/3478 | 68/16992 | 8.82E-06 | 30 |
| GO:0045216 | cell-cell junction organization | 73/3478 | 222/16992 | 9.02E-06 | 73 |
| GO:0016441 | posttranscriptional gene silencing | 29/3478 | 65/16992 | 9.48E-06 | 29 |
| GO:1900034 | regulation of cellular response to heat | 33/3478 | 78/16992 | 9.57E-06 | 33 |
| GO:0051053 | negative regulation of DNA metabolic process | 46/3478 | 123/16992 | 1.07E-05 | 46 |
| GO:0034330 | cell junction organization | 80/3478 | 250/16992 | 1.08E-05 | 80 |
| GO:0031589 | cell-substrate adhesion | 94/3478 | 305/16992 | 1.10E-05 | 94 |
| GO:0051168 | nuclear export | 66/3478 | 197/16992 | 1.21E-05 | 66 |
| GO:0006891 | intra-Golgi vesicle-mediated transport | 23/3478 | 47/16992 | 1.22E-05 | 23 |
| GO:0018209 | peptidyl-serine modification | 91/3478 | 294/16992 | 1.25E-05 | 91 |
| GO:0090002 | establishment of protein localization to plasma membrane | 50/3478 | 138/16992 | 1.26E-05 | 50 |
| GO:0000723 | telomere maintenance | 53/3478 | 149/16992 | 1.29E-05 | 53 |
| GO:0072600 | establishment of protein localization to Golgi | 14/3478 | 22/16992 | 1.32E-05 | 14 |
| GO:0006302 | double-strand break repair | 69/3478 | 209/16992 | 1.35E-05 | 69 |
| GO:0034504 | protein localization to nucleus | 107/3478 | 359/16992 | 1.45E-05 | 107 |
| GO:0016236 | macroautophagy | 82/3478 | 260/16992 | 1.54E-05 | 82 |
| GO:0035195 | gene silencing by miRNA | 27/3478 | 60/16992 | 1.57E-05 | 27 |
| GO:0031047 | gene silencing by RNA | 56/3478 | 161/16992 | 1.59E-05 | 56 |
| GO:0006409 | tRNA export from nucleus | 18/3478 | 33/16992 | 1.62E-05 | 18 |
| GO:0071431 | tRNA-containing ribonucleoprotein complex export from nucleus | 18/3478 | 33/16992 | 1.62E-05 | 18 |
| GO:0007173 | epidermal growth factor receptor signaling pathway | 46/3478 | 125/16992 | 1.74E-05 | 46 |
| GO:0035023 | regulation of Rho protein signal transduction | 46/3478 | 125/16992 | 1.74E-05 | 46 |
| GO:0007045 | cell-substrate adherens junction assembly | 30/3478 | 70/16992 | 1.77E-05 | 30 |
| GO:0046794 | transport of virus | 30/3478 | 70/16992 | 1.77E-05 | 30 |
| GO:0048041 | focal adhesion assembly | 30/3478 | 70/16992 | 1.77E-05 | 30 |
| GO:0043254 | regulation of protein complex assembly | 111/3478 | 377/16992 | 1.83E-05 | 111 |
| GO:0016311 | dephosphorylation | 121/3478 | 418/16992 | 1.87E-05 | 121 |
| GO:0075733 | intracellular transport of virus | 29/3478 | 67/16992 | 1.94E-05 | 29 |
| GO:0051236 | establishment of RNA localization | 65/3478 | 196/16992 | 2.02E-05 | 65 |
| GO:0006289 | nucleotide-excision repair | 43/3478 | 115/16992 | 2.06E-05 | 43 |
| GO:0035194 | posttranscriptional gene silencing by RNA | 28/3478 | 64/16992 | 2.11E-05 | 28 |
| GO:0048813 | dendrite morphogenesis | 44/3478 | 119/16992 | 2.29E-05 | 44 |
| GO:0044783 | G1 DNA damage checkpoint | 30/3478 | 71/16992 | 2.47E-05 | 30 |
| GO:0043414 | macromolecule methylation | 86/3478 | 279/16992 | 2.53E-05 | 86 |
| GO:0006482 | protein demethylation | 17/3478 | 31/16992 | 2.54E-05 | 17 |
| GO:0008214 | protein dealkylation | 17/3478 | 31/16992 | 2.54E-05 | 17 |
| GO:0016458 | gene silencing | 80/3478 | 256/16992 | 2.80E-05 | 80 |
| GO:0035735 | intraciliary transport involved in cilium assembly | 20/3478 | 40/16992 | 3.02E-05 | 20 |
| GO:0030900 | forebrain development | 106/3478 | 361/16992 | 3.17E-05 | 106 |
| GO:0070076 | histone lysine demethylation | 15/3478 | 26/16992 | 3.43E-05 | 15 |
| GO:0051028 | mRNA transport | 52/3478 | 150/16992 | 3.47E-05 | 52 |
| GO:0043297 | apical junction assembly | 25/3478 | 56/16992 | 3.80E-05 | 25 |
| GO:0006888 | ER to Golgi vesicle-mediated transport | 58/3478 | 173/16992 | 3.86E-05 | 58 |

| | | | | | |
|---|---|---|---|---|---|
| GO:0034067 | protein localization to Golgi apparatus | 17/3478 | 32/16992 | 4.39E-05 | 17 |
| GO:0007041 | lysosomal transport | 33/3478 | 83/16992 | 4.43E-05 | 33 |
| GO:0006310 | DNA recombination | 82/3478 | 267/16992 | 4.45E-05 | 82 |
| GO:0072413 | signal transduction involved in mitotic cell cycle checkpoint | 27/3478 | 63/16992 | 4.59E-05 | 27 |
| GO:1902402 | signal transduction involved in mitotic DNA damage checkpoint | 27/3478 | 63/16992 | 4.59E-05 | 27 |
| GO:1902403 | signal transduction involved in mitotic DNA integrity checkpoint | 27/3478 | 63/16992 | 4.59E-05 | 27 |
| GO:0050657 | nucleic acid transport | 63/3478 | 193/16992 | 4.60E-05 | 63 |
| GO:0050658 | RNA transport | 63/3478 | 193/16992 | 4.60E-05 | 63 |
| GO:0051031 | tRNA transport | 18/3478 | 35/16992 | 4.64E-05 | 18 |
| GO:0090329 | regulation of DNA-dependent DNA replication | 21/3478 | 44/16992 | 4.67E-05 | 21 |
| GO:0010976 | positive regulation of neuron projection development | 74/3478 | 236/16992 | 4.85E-05 | 74 |
| GO:0010508 | positive regulation of autophagy | 38/3478 | 101/16992 | 5.20E-05 | 38 |
| GO:0031571 | mitotic G1 DNA damage checkpoint | 29/3478 | 70/16992 | 5.20E-05 | 29 |
| GO:0044819 | mitotic G1/S transition checkpoint | 29/3478 | 70/16992 | 5.20E-05 | 29 |
| GO:0042147 | retrograde transport, endosome to Golgi | 31/3478 | 77/16992 | 5.63E-05 | 31 |
| GO:1900006 | positive regulation of dendrite development | 28/3478 | 67/16992 | 5.78E-05 | 28 |
| GO:0045732 | positive regulation of protein catabolic process | 79/3478 | 257/16992 | 5.88E-05 | 79 |
| GO:0006895 | Golgi to endosome transport | 12/3478 | 19/16992 | 6.27E-05 | 12 |
| GO:0034453 | microtubule anchoring | 12/3478 | 19/16992 | 6.27E-05 | 12 |
| GO:0000288 | nuclear-transcribed mRNA catabolic process, deadenylation-dependent decay | 30/3478 | 74/16992 | 6.33E-05 | 30 |
| GO:1905515 | non-motile cilium assembly | 23/3478 | 51/16992 | 6.34E-05 | 23 |
| GO:2000134 | negative regulation of G1/S transition of mitotic cell cycle | 36/3478 | 95/16992 | 6.80E-05 | 36 |
| GO:0007034 | vacuolar transport | 39/3478 | 106/16992 | 7.40E-05 | 39 |
| GO:0032535 | regulation of cellular component size | 95/3478 | 323/16992 | 7.43E-05 | 95 |
| GO:0007266 | Rho protein signal transduction | 56/3478 | 169/16992 | 7.49E-05 | 56 |
| GO:0060999 | positive regulation of dendritic spine development | 19/3478 | 39/16992 | 7.51E-05 | 19 |
| GO:0000722 | telomere maintenance via recombination | 18/3478 | 36/16992 | 7.51E-05 | 18 |
| GO:0015931 | nucleobase-containing compound transport | 72/3478 | 231/16992 | 7.53E-05 | 72 |
| GO:0051304 | chromosome separation | 28/3478 | 68/16992 | 7.93E-05 | 28 |
| GO:0045332 | phospholipid translocation | 13/3478 | 22/16992 | 8.25E-05 | 13 |
| GO:0070507 | regulation of microtubule cytoskeleton organization | 46/3478 | 132/16992 | 8.28E-05 | 46 |
| GO:0007224 | smoothened signaling pathway | 43/3478 | 121/16992 | 8.33E-05 | 43 |
| GO:0072401 | signal transduction involved in DNA integrity checkpoint | 27/3478 | 65/16992 | 8.83E-05 | 27 |
| GO:0072422 | signal transduction involved in DNA damage checkpoint | 27/3478 | 65/16992 | 8.83E-05 | 27 |
| GO:0030522 | intracellular receptor signaling pathway | 82/3478 | 272/16992 | 9.05E-05 | 82 |
| GO:1901185 | negative regulation of ERBB signaling pathway | 23/3478 | 52/16992 | 9.17E-05 | 23 |
| GO:0072431 | signal transduction involved in mitotic G1 DNA damage checkpoint | 26/3478 | 62/16992 | 9.81E-05 | 26 |
| GO:1902400 | intracellular signal transduction involved in G1 DNA damage checkpoint | 26/3478 | 62/16992 | 9.81E-05 | 26 |
| GO:0007044 | cell-substrate junction assembly | 33/3478 | 86/16992 | 0.000101226 | 33 |
| GO:0016241 | regulation of macroautophagy | 46/3478 | 133/16992 | 0.000101822 | 46 |
| GO:0008286 | insulin receptor signaling pathway | 43/3478 | 122/16992 | 0.000103504 | 43 |
| GO:0071478 | cellular response to radiation | 55/3478 | 167/16992 | 0.000103823 | 55 |
| GO:0061640 | cytoskeleton-dependent cytokinesis | 21/3478 | 46/16992 | 0.000105325 | 21 |

| | | | | | |
|---|---|---|---|---|---|
| GO:1900024 | regulation of substrate adhesion-dependent cell spreading | 21/3478 | 46/16992 | 0.000105325 | 21 |
| GO:0071897 | DNA biosynthetic process | 64/3478 | 202/16992 | 0.000108589 | 64 |
| GO:2001251 | negative regulation of chromosome organization | 41/3478 | 115/16992 | 0.000111017 | 41 |
| GO:0006611 | protein export from nucleus | 58/3478 | 179/16992 | 0.000113292 | 58 |
| GO:0006890 | retrograde vesicle-mediated transport, Golgi to ER | 32/3478 | 83/16992 | 0.000115386 | 32 |
| GO:0051983 | regulation of chromosome segregation | 32/3478 | 83/16992 | 0.000115386 | 32 |
| GO:0021987 | cerebral cortex development | 39/3478 | 108/16992 | 0.000117998 | 39 |
| GO:0031647 | regulation of protein stability | 73/3478 | 238/16992 | 0.00011854 | 73 |
| GO:0051494 | negative regulation of cytoskeleton organization | 42/3478 | 119/16992 | 0.00011958 | 42 |
| GO:0072395 | signal transduction involved in cell cycle checkpoint | 27/3478 | 66/16992 | 0.000120593 | 27 |
| GO:0033962 | cytoplasmic mRNA processing body assembly | 12/3478 | 20/16992 | 0.000127452 | 12 |
| GO:0001701 | in utero embryonic development | 91/3478 | 311/16992 | 0.000127487 | 91 |
| GO:0006283 | transcription-coupled nucleotide-excision repair | 29/3478 | 73/16992 | 0.00012803 | 29 |
| GO:0006476 | protein deacetylation | 35/3478 | 94/16992 | 0.000128712 | 35 |
| GO:0033047 | regulation of mitotic sister chromatid segregation | 23/3478 | 53/16992 | 0.000130919 | 23 |
| GO:0090307 | mitotic spindle assembly | 23/3478 | 53/16992 | 0.000130919 | 23 |
| GO:0006999 | nuclear pore organization | 10/3478 | 15/16992 | 0.000137998 | 10 |
| GO:1902807 | negative regulation of cell cycle G1/S phase transition | 36/3478 | 98/16992 | 0.000142712 | 36 |
| GO:0010720 | positive regulation of cell development | 125/3478 | 453/16992 | 0.000142865 | 125 |
| GO:0010171 | body morphogenesis | 21/3478 | 47/16992 | 0.000153917 | 21 |
| GO:0070830 | bicellular tight junction assembly | 21/3478 | 47/16992 | 0.000153917 | 21 |
| GO:0034204 | lipid translocation | 13/3478 | 23/16992 | 0.000154162 | 13 |
| GO:0051642 | centrosome localization | 13/3478 | 23/16992 | 0.000154162 | 13 |
| GO:0006479 | protein methylation | 57/3478 | 177/16992 | 0.000155324 | 57 |
| GO:0008213 | protein alkylation | 57/3478 | 177/16992 | 0.000155324 | 57 |
| GO:0000910 | cytokinesis | 47/3478 | 139/16992 | 0.000159587 | 47 |
| GO:0032200 | telomere organization | 53/3478 | 162/16992 | 0.000166175 | 53 |
| GO:0007160 | cell-matrix adhesion | 62/3478 | 197/16992 | 0.000169879 | 62 |
| GO:1901796 | regulation of signal transduction by p53 class mediator | 54/3478 | 166/16992 | 0.00017098 | 54 |
| GO:0006903 | vesicle targeting | 31/3478 | 81/16992 | 0.000171562 | 31 |
| GO:0035601 | protein deacylation | 36/3478 | 99/16992 | 0.000180589 | 36 |
| GO:0044786 | cell cycle DNA replication | 18/3478 | 38/16992 | 0.000182207 | 18 |
| GO:0033045 | regulation of sister chromatid segregation | 26/3478 | 64/16992 | 0.000183233 | 26 |
| GO:1903391 | regulation of adherens junction organization | 23/3478 | 54/16992 | 0.000184229 | 23 |
| GO:0000377 | RNA splicing, via transesterification reactions with bulged adenosine as nucleophile | 91/3478 | 314/16992 | 0.000184469 | 91 |
| GO:0000398 | mRNA splicing, via spliceosome | 91/3478 | 314/16992 | 0.000184469 | 91 |
| GO:0072698 | protein localization to microtubule cytoskeleton | 17/3478 | 35/16992 | 0.000187109 | 17 |
| GO:0090503 | RNA phosphodiester bond hydrolysis, exonucleolytic | 17/3478 | 35/16992 | 0.000187109 | 17 |
| GO:0006446 | regulation of translational initiation | 28/3478 | 71/16992 | 0.000192573 | 28 |
| GO:1990138 | neuron projection extension | 47/3478 | 140/16992 | 0.000193185 | 47 |
| GO:0050773 | regulation of dendrite development | 43/3478 | 125/16992 | 0.00019332 | 43 |
| GO:0006622 | protein targeting to lysosome | 11/3478 | 18/16992 | 0.000196207 | 11 |
| GO:0030518 | intracellular steroid hormone receptor signaling pathway | 44/3478 | 129/16992 | 0.000203883 | 44 |
| GO:0006977 | DNA damage response, signal transduction by p53 class mediator resulting in cell cycle arrest | 25/3478 | 61/16992 | 0.000205473 | 25 |
| GO:0010975 | regulation of neuron projection development | 112/3478 | 402/16992 | 0.000206383 | 112 |

| | | | | | |
|---|---|---|---|---|---|
| GO:0001952 | regulation of cell-matrix adhesion | 35/3478 | 96/16992 | 0.000208045 | 35 |
| GO:0030218 | erythrocyte differentiation | 35/3478 | 96/16992 | 0.000208045 | 35 |
| GO:0046605 | regulation of centrosome cycle | 21/3478 | 48/16992 | 0.000221247 | 21 |
| GO:0098732 | macromolecule deacylation | 36/3478 | 100/16992 | 0.000227242 | 36 |
| GO:0031122 | cytoplasmic microtubule organization | 20/3478 | 45/16992 | 0.000239918 | 20 |
| GO:0032970 | regulation of actin filament-based process | 94/3478 | 329/16992 | 0.000252806 | 94 |
| GO:0000375 | RNA splicing, via transesterification reactions | 91/3478 | 317/16992 | 0.000263884 | 91 |
| GO:0061842 | microtubule organizing center localization | 13/3478 | 24/16992 | 0.000273445 | 13 |
| GO:0033683 | nucleotide-excision repair, DNA incision | 18/3478 | 39/16992 | 0.000274046 | 18 |
| GO:0021537 | telencephalon development | 71/3478 | 236/16992 | 0.000276434 | 71 |
| GO:0060998 | regulation of dendritic spine development | 25/3478 | 62/16992 | 0.000278011 | 25 |
| GO:0043038 | amino acid activation | 22/3478 | 52/16992 | 0.00028375 | 22 |
| GO:0000281 | mitotic cytokinesis | 17/3478 | 36/16992 | 0.000287298 | 17 |
| GO:0031572 | G2 DNA damage checkpoint | 17/3478 | 36/16992 | 0.000287298 | 17 |
| GO:0046931 | pore complex assembly | 10/3478 | 16/16992 | 0.000300348 | 10 |
| GO:0015758 | glucose transport | 50/3478 | 154/16992 | 0.000307091 | 50 |
| GO:0051258 | protein polymerization | 72/3478 | 241/16992 | 0.000315802 | 72 |
| GO:0031532 | actin cytoskeleton reorganization | 32/3478 | 87/16992 | 0.000317685 | 32 |
| GO:0032006 | regulation of TOR signaling | 32/3478 | 87/16992 | 0.000317685 | 32 |
| GO:0021543 | pallium development | 52/3478 | 162/16992 | 0.000322052 | 52 |
| GO:0008589 | regulation of smoothened signaling pathway | 26/3478 | 66/16992 | 0.000328332 | 26 |
| GO:0000731 | DNA synthesis involved in DNA repair | 28/3478 | 73/16992 | 0.000332727 | 28 |
| GO:0032204 | regulation of telomere maintenance | 28/3478 | 73/16992 | 0.000332727 | 28 |
| GO:0009314 | response to radiation | 118/3478 | 432/16992 | 0.000334253 | 118 |
| GO:1902593 | single-organism nuclear import | 80/3478 | 274/16992 | 0.000339697 | 80 |
| GO:0016239 | positive regulation of macroautophagy | 23/3478 | 56/16992 | 0.000350846 | 23 |
| GO:0031929 | TOR signaling | 36/3478 | 102/16992 | 0.000354024 | 36 |
| GO:1902806 | regulation of cell cycle G1/S phase transition | 49/3478 | 151/16992 | 0.000355604 | 49 |
| GO:0090068 | positive regulation of cell cycle process | 74/3478 | 250/16992 | 0.000356863 | 74 |
| GO:0016571 | histone methylation | 44/3478 | 132/16992 | 0.000362494 | 44 |
| GO:0032392 | DNA geometric change | 31/3478 | 84/16992 | 0.000365635 | 31 |
| GO:0018022 | peptidyl-lysine methylation | 40/3478 | 117/16992 | 0.000367031 | 40 |
| GO:0034968 | histone lysine methylation | 37/3478 | 106/16992 | 0.00037871 | 37 |
| GO:0006606 | protein import into nucleus | 79/3478 | 271/16992 | 0.000388592 | 79 |
| GO:0044744 | protein targeting to nucleus | 79/3478 | 271/16992 | 0.000388592 | 79 |
| GO:0042059 | negative regulation of epidermal growth factor receptor signaling pathway | 18/3478 | 40/16992 | 0.000403626 | 18 |
| GO:1902743 | regulation of lamellipodium organization | 18/3478 | 40/16992 | 0.000403626 | 18 |
| GO:0007368 | determination of left/right symmetry | 39/3478 | 114/16992 | 0.000425298 | 39 |
| GO:0008645 | hexose transport | 50/3478 | 156/16992 | 0.000431386 | 50 |
| GO:0032508 | DNA duplex unwinding | 28/3478 | 74/16992 | 0.000431917 | 28 |
| GO:1901888 | regulation of cell junction assembly | 28/3478 | 74/16992 | 0.000431917 | 28 |
| GO:0007064 | mitotic sister chromatid cohesion | 12/3478 | 22/16992 | 0.000433463 | 12 |
| GO:0008053 | mitochondrial fusion | 12/3478 | 22/16992 | 0.000433463 | 12 |
| GO:0032878 | regulation of establishment or maintenance of cell polarity | 12/3478 | 22/16992 | 0.000433463 | 12 |
| GO:0030865 | cortical cytoskeleton organization | 16/3478 | 34/16992 | 0.000453505 | 16 |

| | | | | | |
|---|---|---|---|---|---|
| GO:1903362 | regulation of cellular protein catabolic process | 76/3478 | 260/16992 | 0.000453551 | 76 |
| GO:0071539 | protein localization to centrosome | 13/3478 | 25/16992 | 0.000463294 | 13 |
| GO:1905508 | protein localization to microtubule organizing center | 13/3478 | 25/16992 | 0.000463294 | 13 |
| GO:0016601 | Rac protein signal transduction | 15/3478 | 31/16992 | 0.000468912 | 15 |
| GO:0007099 | centriole replication | 14/3478 | 28/16992 | 0.00047357 | 14 |
| GO:1903050 | regulation of proteolysis involved in cellular protein catabolic process | 70/3478 | 236/16992 | 0.000475337 | 70 |
| GO:0045666 | positive regulation of neuron differentiation | 90/3478 | 318/16992 | 0.000475504 | 90 |
| GO:0035107 | appendage morphogenesis | 47/3478 | 145/16992 | 0.000477008 | 47 |
| GO:0035108 | limb morphogenesis | 47/3478 | 145/16992 | 0.000477008 | 47 |
| GO:0042073 | intraciliary transport | 20/3478 | 47/16992 | 0.000483848 | 20 |
| GO:0048008 | platelet-derived growth factor receptor signaling pathway | 20/3478 | 47/16992 | 0.000483848 | 20 |
| GO:0006406 | mRNA export from nucleus | 38/3478 | 111/16992 | 0.000492895 | 38 |
| GO:0071427 | mRNA-containing ribonucleoprotein complex export from nucleus | 38/3478 | 111/16992 | 0.000492895 | 38 |
| GO:0051865 | protein autoubiquitination | 22/3478 | 54/16992 | 0.000535199 | 22 |
| GO:0034101 | erythrocyte homeostasis | 36/3478 | 104/16992 | 0.000540223 | 36 |
| GO:1901184 | regulation of ERBB signaling pathway | 33/3478 | 93/16992 | 0.000547945 | 33 |
| GO:0015749 | monosaccharide transport | 50/3478 | 158/16992 | 0.000598919 | 50 |
| GO:0043039 | tRNA aminoacylation | 21/3478 | 51/16992 | 0.000600594 | 21 |
| GO:0051893 | regulation of focal adhesion assembly | 21/3478 | 51/16992 | 0.000600594 | 21 |
| GO:0090109 | regulation of cell-substrate junction assembly | 21/3478 | 51/16992 | 0.000600594 | 21 |
| GO:0090305 | nucleic acid phosphodiester bond hydrolysis | 81/3478 | 283/16992 | 0.000613492 | 81 |
| GO:1903829 | positive regulation of cellular protein localization | 103/3478 | 375/16992 | 0.000626777 | 103 |
| GO:0044380 | protein localization to cytoskeleton | 17/3478 | 38/16992 | 0.000632092 | 17 |
| GO:0050769 | positive regulation of neurogenesis | 107/3478 | 392/16992 | 0.000633325 | 107 |
| GO:0030032 | lamellipodium assembly | 23/3478 | 58/16992 | 0.000636868 | 23 |
| GO:2000045 | regulation of G1/S transition of mitotic cell cycle | 45/3478 | 139/16992 | 0.000640207 | 45 |
| GO:0032869 | cellular response to insulin stimulus | 59/3478 | 194/16992 | 0.000642531 | 59 |
| GO:0040029 | regulation of gene expression, epigenetic | 77/3478 | 267/16992 | 0.000647887 | 77 |
| GO:0051170 | nuclear import | 83/3478 | 292/16992 | 0.000667951 | 83 |
| GO:0006418 | tRNA aminoacylation for protein translation | 20/3478 | 48/16992 | 0.000671421 | 20 |
| GO:0051306 | mitotic sister chromatid separation | 20/3478 | 48/16992 | 0.000671421 | 20 |
| GO:0098840 | protein transport along microtubule | 20/3478 | 48/16992 | 0.000671421 | 20 |
| GO:0099118 | microtubule-based protein transport | 20/3478 | 48/16992 | 0.000671421 | 20 |
| GO:0010972 | negative regulation of G2/M transition of mitotic cell cycle | 30/3478 | 83/16992 | 0.000676037 | 30 |
| GO:0060996 | dendritic spine development | 30/3478 | 83/16992 | 0.000676037 | 30 |
| GO:2000114 | regulation of establishment of cell polarity | 11/3478 | 20/16992 | 0.000688271 | 11 |
| GO:0071482 | cellular response to light stimulus | 37/3478 | 109/16992 | 0.000696814 | 37 |
| GO:0014911 | positive regulation of smooth muscle cell migration | 15/3478 | 32/16992 | 0.000716753 | 15 |
| GO:0072666 | establishment of protein localization to vacuole | 15/3478 | 32/16992 | 0.000716753 | 15 |
| GO:1900026 | positive regulation of substrate adhesion-dependent cell spreading | 15/3478 | 32/16992 | 0.000716753 | 15 |
| GO:0032259 | methylation | 96/3478 | 347/16992 | 0.000726999 | 96 |
| GO:0044089 | positive regulation of cellular component biogenesis | 124/3478 | 466/16992 | 0.000732102 | 124 |
| GO:0048013 | ephrin receptor signaling pathway | 31/3478 | 87/16992 | 0.000733366 | 31 |
| GO:1903364 | positive regulation of cellular protein catabolic process | 58/3478 | 191/16992 | 0.000740147 | 58 |

| | | | | | |
|---|---|---|---|---|---|
| GO:0006623 | protein targeting to vacuole | 14/3478 | 29/16992 | 0.000744136 | 14 |
| GO:0032956 | regulation of actin cytoskeleton organization | 82/3478 | 289/16992 | 0.000760442 | 82 |
| GO:0071359 | cellular response to dsRNA | 21/3478 | 52/16992 | 0.000815231 | 21 |
| GO:0048872 | homeostasis of number of cells | 67/3478 | 228/16992 | 0.000816322 | 67 |
| GO:0061647 | histone H3-K9 modification | 18/3478 | 42/16992 | 0.000826847 | 18 |
| GO:1903052 | positive regulation of proteolysis involved in cellular protein catabolic process | 54/3478 | 176/16992 | 0.000846643 | 54 |
| GO:0034605 | cellular response to heat | 38/3478 | 114/16992 | 0.000881183 | 38 |
| GO:0070936 | protein K48-linked ubiquitination | 20/3478 | 49/16992 | 0.000918524 | 20 |
| GO:0072673 | lamellipodium morphogenesis | 9/3478 | 15/16992 | 0.000932526 | 9 |
| GO:0034502 | protein localization to chromosome | 26/3478 | 70/16992 | 0.000943283 | 26 |
| GO:0009855 | determination of bilateral symmetry | 40/3478 | 122/16992 | 0.000943931 | 40 |
| GO:0002262 | myeloid cell homeostasis | 41/3478 | 126/16992 | 0.000971505 | 41 |
| GO:0019886 | antigen processing and presentation of exogenous peptide antigen via MHC class II | 32/3478 | 92/16992 | 0.000975078 | 32 |
| GO:0048010 | vascular endothelial growth factor receptor signaling pathway | 32/3478 | 92/16992 | 0.000975078 | 32 |
| GO:0022618 | ribonucleoprotein complex assembly | 58/3478 | 193/16992 | 0.000979685 | 58 |
| GO:0016575 | histone deacetylation | 29/3478 | 81/16992 | 0.000981708 | 29 |
| GO:0070897 | DNA-templated transcriptional preinitiation complex assembly | 16/3478 | 36/16992 | 0.00099027 | 16 |
| GO:0010826 | negative regulation of centrosome duplication | 7/3478 | 10/16992 | 0.000997688 | 7 |
| GO:0046606 | negative regulation of centrosome cycle | 7/3478 | 10/16992 | 0.000997688 | 7 |
| GO:0051292 | nuclear pore complex assembly | 7/3478 | 10/16992 | 0.000997688 | 7 |
| GO:0070102 | interleukin-6-mediated signaling pathway | 7/3478 | 10/16992 | 0.000997688 | 7 |
| GO:0006893 | Golgi to plasma membrane transport | 19/3478 | 46/16992 | 0.001031268 | 19 |
| GO:0007091 | metaphase/anaphase transition of mitotic cell cycle | 19/3478 | 46/16992 | 0.001031268 | 19 |
| GO:0010965 | regulation of mitotic sister chromatid separation | 19/3478 | 46/16992 | 0.001031268 | 19 |
| GO:0043044 | ATP-dependent chromatin remodeling | 27/3478 | 74/16992 | 0.001041403 | 27 |
| GO:0071158 | positive regulation of cell cycle arrest | 30/3478 | 85/16992 | 0.001058146 | 30 |
| GO:1902750 | negative regulation of cell cycle G2/M phase transition | 30/3478 | 85/16992 | 0.001058146 | 30 |
| GO:0032212 | positive regulation of telomere maintenance via telomerase | 15/3478 | 33/16992 | 0.001067395 | 15 |
| GO:0045070 | positive regulation of viral genome replication | 15/3478 | 33/16992 | 0.001067395 | 15 |
| GO:0050775 | positive regulation of dendrite morphogenesis | 15/3478 | 33/16992 | 0.001067395 | 15 |
| GO:0008361 | regulation of cell size | 47/3478 | 150/16992 | 0.001086997 | 47 |
| GO:0048015 | phosphatidylinositol-mediated signaling | 61/3478 | 206/16992 | 0.001106395 | 61 |
| GO:0009799 | specification of symmetry | 40/3478 | 123/16992 | 0.0011267 | 40 |
| GO:0009791 | post-embryonic development | 31/3478 | 89/16992 | 0.001130724 | 31 |
| GO:0032007 | negative regulation of TOR signaling | 18/3478 | 43/16992 | 0.001152905 | 18 |
| GO:1990090 | cellular response to nerve growth factor stimulus | 18/3478 | 43/16992 | 0.001152905 | 18 |
| GO:0006293 | nucleotide-excision repair, preincision complex stabilization | 11/3478 | 21/16992 | 0.001179153 | 11 |
| GO:0006295 | nucleotide-excision repair, DNA incision, 3'-to lesion | 11/3478 | 21/16992 | 0.001179153 | 11 |
| GO:0051647 | nucleus localization | 11/3478 | 21/16992 | 0.001179153 | 11 |
| GO:0070536 | protein K63-linked deubiquitination | 13/3478 | 27/16992 | 0.001183198 | 13 |
| GO:0034644 | cellular response to UV | 26/3478 | 71/16992 | 0.001202404 | 26 |
| GO:0032386 | regulation of intracellular transport | 130/3478 | 498/16992 | 0.001231047 | 130 |
| GO:0010212 | response to ionizing radiation | 46/3478 | 147/16992 | 0.001257346 | 46 |

| | | | | | |
|---|---|---|---|---|---|
| GO:0099518 | vesicle cytoskeletal trafficking | 17/3478 | 40/16992 | 0.001282126 | 17 |
| GO:0048814 | regulation of dendrite morphogenesis | 27/3478 | 75/16992 | 0.001314305 | 27 |
| GO:0000724 | double-strand break repair via homologous recombination | 34/3478 | 101/16992 | 0.001322829 | 34 |
| GO:0044784 | metaphase/anaphase transition of cell cycle | 19/3478 | 47/16992 | 0.001402731 | 19 |
| GO:1905818 | regulation of chromosome separation | 19/3478 | 47/16992 | 0.001402731 | 19 |
| GO:0006296 | nucleotide-excision repair, DNA incision, 5'-to lesion | 16/3478 | 37/16992 | 0.001416512 | 16 |
| GO:0010824 | regulation of centrosome duplication | 16/3478 | 37/16992 | 0.001416512 | 16 |
| GO:1901998 | toxin transport | 16/3478 | 37/16992 | 0.001416512 | 16 |
| GO:0021915 | neural tube development | 48/3478 | 156/16992 | 0.001488678 | 48 |
| GO:0008154 | actin polymerization or depolymerization | 55/3478 | 184/16992 | 0.001491553 | 55 |
| GO:0061001 | regulation of dendritic spine morphogenesis | 15/3478 | 34/16992 | 0.001552045 | 15 |
| GO:2000785 | regulation of autophagosome assembly | 15/3478 | 34/16992 | 0.001552045 | 15 |
| GO:1902903 | regulation of supramolecular fiber organization | 79/3478 | 283/16992 | 0.001554985 | 79 |
| GO:0030071 | regulation of mitotic metaphase/anaphase transition | 18/3478 | 44/16992 | 0.001582072 | 18 |
| GO:0017038 | protein import | 88/3478 | 321/16992 | 0.001587075 | 88 |
| GO:0030326 | embryonic limb morphogenesis | 40/3478 | 125/16992 | 0.001587273 | 40 |
| GO:0035113 | embryonic appendage morphogenesis | 40/3478 | 125/16992 | 0.001587273 | 40 |
| GO:0000725 | recombinational repair | 34/3478 | 102/16992 | 0.001600202 | 34 |
| GO:0061564 | axon development | 120/3478 | 458/16992 | 0.001612199 | 120 |
| GO:0048017 | inositol lipid-mediated signaling | 61/3478 | 209/16992 | 0.001627994 | 61 |
| GO:0060560 | developmental growth involved in morphogenesis | 60/3478 | 205/16992 | 0.00164404 | 60 |
| GO:0071826 | ribonucleoprotein complex subunit organization | 60/3478 | 205/16992 | 0.00164404 | 60 |
| GO:0097061 | dendritic spine organization | 22/3478 | 58/16992 | 0.001651453 | 22 |
| GO:1903322 | positive regulation of protein modification by small protein conjugation or removal | 58/3478 | 197/16992 | 0.001673022 | 58 |
| GO:0045005 | DNA-dependent DNA replication maintenance of fidelity | 14/3478 | 31/16992 | 0.001682434 | 14 |
| GO:0098534 | centriole assembly | 14/3478 | 31/16992 | 0.001682434 | 14 |
| GO:0006405 | RNA export from nucleus | 44/3478 | 141/16992 | 0.00168277 | 44 |
| GO:0060491 | regulation of cell projection assembly | 46/3478 | 149/16992 | 0.001710117 | 46 |
| GO:0008064 | regulation of actin polymerization or depolymerization | 49/3478 | 161/16992 | 0.001729589 | 49 |
| GO:0031032 | actomyosin structure organization | 50/3478 | 165/16992 | 0.001731012 | 50 |
| GO:0007095 | mitotic G2 DNA damage checkpoint | 9/3478 | 16/16992 | 0.001745327 | 9 |
| GO:0010389 | regulation of G2/M transition of mitotic cell cycle | 37/3478 | 114/16992 | 0.001759798 | 37 |
| GO:0042058 | regulation of epidermal growth factor receptor signaling pathway | 28/3478 | 80/16992 | 0.00176547 | 28 |
| GO:0002495 | antigen processing and presentation of peptide antigen via MHC class II | 32/3478 | 95/16992 | 0.001784529 | 32 |
| GO:1902749 | regulation of cell cycle G2/M phase transition | 39/3478 | 122/16992 | 0.0018405 | 39 |
| GO:0007004 | telomere maintenance via telomerase | 23/3478 | 62/16992 | 0.001848223 | 23 |
| GO:0035335 | peptidyl-tyrosine dephosphorylation | 33/3478 | 99/16992 | 0.001858888 | 33 |
| GO:0032210 | regulation of telomere maintenance via telomerase | 19/3478 | 48/16992 | 0.001881865 | 19 |
| GO:0006198 | cAMP catabolic process | 10/3478 | 19/16992 | 0.001889431 | 10 |
| GO:0032506 | cytokinetic process | 10/3478 | 19/16992 | 0.001889431 | 10 |
| GO:0071166 | ribonucleoprotein complex localization | 41/3478 | 130/16992 | 0.001902344 | 41 |
| GO:0000717 | nucleotide-excision repair, DNA duplex unwinding | 11/3478 | 22/16992 | 0.001924843 | 11 |
| GO:0009214 | cyclic nucleotide catabolic process | 11/3478 | 22/16992 | 0.001924843 | 11 |
| GO:0016578 | histone deubiquitination | 11/3478 | 22/16992 | 0.001924843 | 11 |

| | | | | | |
|---|---|---|---|---|---|
| GO:0031398 | positive regulation of protein ubiquitination | 54/3478 | 182/16992 | 0.001962201 | 54 |
| GO:0030833 | regulation of actin filament polymerization | 44/3478 | 142/16992 | 0.001963485 | 44 |
| GO:0050821 | protein stabilization | 45/3478 | 146/16992 | 0.001976313 | 45 |
| GO:0050688 | regulation of defense response to virus | 30/3478 | 88/16992 | 0.001979072 | 30 |
| GO:0050770 | regulation of axonogenesis | 46/3478 | 150/16992 | 0.001985728 | 46 |
| GO:0098927 | vesicle-mediated transport between endosomal compartments | 16/3478 | 38/16992 | 0.001986999 | 16 |
| GO:0030832 | regulation of actin filament length | 49/3478 | 162/16992 | 0.001995582 | 49 |
| GO:0030521 | androgen receptor signaling pathway | 24/3478 | 66/16992 | 0.002035941 | 24 |
| GO:0006900 | membrane budding | 36/3478 | 111/16992 | 0.002042787 | 36 |
| GO:0016055 | Wnt signaling pathway | 122/3478 | 470/16992 | 0.002113553 | 122 |
| GO:0010501 | RNA secondary structure unwinding | 18/3478 | 45/16992 | 0.002138674 | 18 |
| GO:1902099 | regulation of metaphase/anaphase transition of cell cycle | 18/3478 | 45/16992 | 0.002138674 | 18 |
| GO:0002504 | antigen processing and presentation of peptide or polysaccharide antigen via MHC class II | 32/3478 | 96/16992 | 0.002160073 | 32 |
| GO:1903320 | regulation of protein modification by small protein conjugation or removal | 78/3478 | 282/16992 | 0.002178103 | 78 |
| GO:1904358 | positive regulation of telomere maintenance via telomere lengthening | 15/3478 | 35/16992 | 0.0022077 | 15 |
| GO:0008643 | carbohydrate transport | 55/3478 | 187/16992 | 0.002221663 | 55 |
| GO:0048675 | axon extension | 33/3478 | 100/16992 | 0.002238453 | 33 |
| GO:1903008 | organelle disassembly | 33/3478 | 100/16992 | 0.002238453 | 33 |
| GO:0003356 | regulation of cilium beat frequency | 7/3478 | 11/16992 | 0.002258243 | 7 |
| GO:0016322 | neuron remodeling | 7/3478 | 11/16992 | 0.002258243 | 7 |
| GO:0033169 | histone H3-K9 demethylation | 7/3478 | 11/16992 | 0.002258243 | 7 |
| GO:0060211 | regulation of nuclear-transcribed mRNA poly(A) tail shortening | 7/3478 | 11/16992 | 0.002258243 | 7 |
| GO:0060213 | positive regulation of nuclear-transcribed mRNA poly(A) tail shortening | 7/3478 | 11/16992 | 0.002258243 | 7 |
| GO:0071044 | histone mRNA catabolic process | 7/3478 | 11/16992 | 0.002258243 | 7 |
| GO:1904754 | positive regulation of vascular associated smooth muscle cell migration | 7/3478 | 11/16992 | 0.002258243 | 7 |
| GO:0198738 | cell-cell signaling by wnt | 122/3478 | 471/16992 | 0.002288013 | 122 |
| GO:0043331 | response to dsRNA | 29/3478 | 85/16992 | 0.002300004 | 29 |
| GO:1904668 | positive regulation of ubiquitin protein ligase activity | 29/3478 | 85/16992 | 0.002300004 | 29 |
| GO:0071559 | response to transforming growth factor beta | 62/3478 | 216/16992 | 0.002322633 | 62 |
| GO:0051567 | histone H3-K9 methylation | 14/3478 | 32/16992 | 0.002433519 | 14 |
| GO:0070911 | global genome nucleotide-excision repair | 14/3478 | 32/16992 | 0.002433519 | 14 |
| GO:0032206 | positive regulation of telomere maintenance | 19/3478 | 49/16992 | 0.002491981 | 19 |
| GO:0000726 | non-recombinational repair | 27/3478 | 78/16992 | 0.002532557 | 27 |
| GO:0008380 | RNA splicing | 109/3478 | 416/16992 | 0.002558206 | 109 |
| GO:0007015 | actin filament organization | 92/3478 | 343/16992 | 0.002570306 | 92 |
| GO:0071426 | ribonucleoprotein complex export from nucleus | 40/3478 | 128/16992 | 0.002583355 | 40 |
| GO:0019884 | antigen processing and presentation of exogenous antigen | 52/3478 | 176/16992 | 0.002591368 | 52 |
| GO:0032271 | regulation of protein polymerization | 52/3478 | 176/16992 | 0.002591368 | 52 |
| GO:0030041 | actin filament polymerization | 47/3478 | 156/16992 | 0.002651957 | 47 |
| GO:0006294 | nucleotide-excision repair, preincision complex assembly | 13/3478 | 29/16992 | 0.002654644 | 13 |
| GO:0030866 | cortical actin cytoskeleton organization | 13/3478 | 29/16992 | 0.002654644 | 13 |
| GO:2001252 | positive regulation of chromosome organization | 46/3478 | 152/16992 | 0.002654801 | 46 |

| | | | | | |
|---|---|---|---|---|---|
| GO:0098876 | vesicle-mediated transport to the plasma membrane | 25/3478 | 71/16992 | 0.002762466 | 25 |
| GO:0022613 | ribonucleoprotein complex biogenesis | 115/3478 | 443/16992 | 0.002774972 | 115 |
| GO:0050855 | regulation of B cell receptor signaling pathway | 8/3478 | 14/16992 | 0.002788289 | 8 |
| GO:0003007 | heart morphogenesis | 65/3478 | 230/16992 | 0.002794758 | 65 |
| GO:0071214 | cellular response to abiotic stimulus | 80/3478 | 293/16992 | 0.002827621 | 80 |
| GO:1990089 | response to nerve growth factor | 18/3478 | 46/16992 | 0.002850578 | 18 |
| GO:0021591 | ventricular system development | 12/3478 | 26/16992 | 0.002855581 | 12 |
| GO:1902745 | positive regulation of lamellipodium organization | 12/3478 | 26/16992 | 0.002855581 | 12 |
| GO:1904353 | regulation of telomere capping | 12/3478 | 26/16992 | 0.002855581 | 12 |
| GO:0007409 | axonogenesis | 110/3478 | 422/16992 | 0.00293571 | 110 |
| GO:0048284 | organelle fusion | 61/3478 | 214/16992 | 0.002984278 | 61 |
| GO:0071560 | cellular response to transforming growth factor beta stimulus | 61/3478 | 214/16992 | 0.002984278 | 61 |
| GO:0002478 | antigen processing and presentation of exogenous peptide antigen | 50/3478 | 169/16992 | 0.002999395 | 50 |
| GO:0048736 | appendage development | 50/3478 | 169/16992 | 0.002999395 | 50 |
| GO:0060173 | limb development | 50/3478 | 169/16992 | 0.002999395 | 50 |
| GO:0007020 | microtubule nucleation | 11/3478 | 23/16992 | 0.003012641 | 11 |
| GO:0009081 | branched-chain amino acid metabolic process | 11/3478 | 23/16992 | 0.003012641 | 11 |
| GO:0045948 | positive regulation of translational initiation | 11/3478 | 23/16992 | 0.003012641 | 11 |
| GO:0006884 | cell volume homeostasis | 9/3478 | 17/16992 | 0.003038742 | 9 |
| GO:0031958 | corticosteroid receptor signaling pathway | 9/3478 | 17/16992 | 0.003038742 | 9 |
| GO:0061003 | positive regulation of dendritic spine morphogenesis | 9/3478 | 17/16992 | 0.003038742 | 9 |
| GO:0097320 | plasma membrane tubulation | 9/3478 | 17/16992 | 0.003038742 | 9 |
| GO:0031396 | regulation of protein ubiquitination | 72/3478 | 260/16992 | 0.003039623 | 72 |
| GO:0047496 | vesicle transport along microtubule | 15/3478 | 36/16992 | 0.003077293 | 15 |
| GO:0007063 | regulation of sister chromatid cohesion | 10/3478 | 20/16992 | 0.003090591 | 10 |
| GO:0009083 | branched-chain amino acid catabolic process | 10/3478 | 20/16992 | 0.003090591 | 10 |
| GO:0044030 | regulation of DNA methylation | 10/3478 | 20/16992 | 0.003090591 | 10 |
| GO:1901889 | negative regulation of cell junction assembly | 10/3478 | 20/16992 | 0.003090591 | 10 |
| GO:0045787 | positive regulation of cell cycle | 91/3478 | 341/16992 | 0.003163284 | 91 |
| GO:0098781 | ncRNA transcription | 33/3478 | 102/16992 | 0.003200564 | 33 |
| GO:2000058 | regulation of protein ubiquitination involved in ubiquitin-dependent protein catabolic process | 34/3478 | 106/16992 | 0.003272451 | 34 |
| GO:0060627 | regulation of vesicle-mediated transport | 116/3478 | 450/16992 | 0.00341787 | 116 |
| GO:0031146 | SCF-dependent proteasomal ubiquitin-dependent protein catabolic process | 25/3478 | 72/16992 | 0.003422948 | 25 |
| GO:0031333 | negative regulation of protein complex assembly | 37/3478 | 118/16992 | 0.003430446 | 37 |
| GO:0051653 | spindle localization | 14/3478 | 33/16992 | 0.003440137 | 14 |
| GO:0042733 | embryonic digit morphogenesis | 22/3478 | 61/16992 | 0.003451453 | 22 |
| GO:0070988 | demethylation | 22/3478 | 61/16992 | 0.003451453 | 22 |
| GO:2000060 | positive regulation of protein ubiquitination involved in ubiquitin-dependent protein catabolic process | 30/3478 | 91/16992 | 0.003517944 | 30 |
| GO:0035264 | multicellular organism growth | 43/3478 | 142/16992 | 0.003536076 | 43 |
| GO:0051098 | regulation of binding | 86/3478 | 321/16992 | 0.003588142 | 86 |
| GO:0006301 | postreplication repair | 20/3478 | 54/16992 | 0.003644903 | 20 |
| GO:0060324 | face development | 18/3478 | 47/16992 | 0.003749242 | 18 |
| GO:0043401 | steroid hormone mediated signaling pathway | 53/3478 | 183/16992 | 0.003763611 | 53 |

| | | | | | |
|---|---|---|---|---|---|
| GO:0009416 | response to light stimulus | 80/3478 | 296/16992 | 0.003786037 | 80 |
| GO:0001953 | negative regulation of cell-matrix adhesion | 13/3478 | 30/16992 | 0.00381719 | 13 |
| GO:1902904 | negative regulation of supramolecular fiber organization | 35/3478 | 111/16992 | 0.003929158 | 35 |
| GO:0031109 | microtubule polymerization or depolymerization | 28/3478 | 84/16992 | 0.003952775 | 28 |
| GO:0043112 | receptor metabolic process | 46/3478 | 155/16992 | 0.004020872 | 46 |
| GO:0007088 | regulation of mitotic nuclear division | 45/3478 | 151/16992 | 0.004043548 | 45 |
| GO:0098727 | maintenance of cell number | 45/3478 | 151/16992 | 0.004043548 | 45 |
| GO:0034250 | positive regulation of cellular amide metabolic process | 38/3478 | 123/16992 | 0.004044168 | 38 |
| GO:0007050 | cell cycle arrest | 68/3478 | 246/16992 | 0.004073876 | 68 |
| GO:0006359 | regulation of transcription from RNA polymerase III promoter | 12/3478 | 27/16992 | 0.004193487 | 12 |
| GO:2000300 | regulation of synaptic vesicle exocytosis | 12/3478 | 27/16992 | 0.004193487 | 12 |
| GO:0097035 | regulation of membrane lipid distribution | 15/3478 | 37/16992 | 0.004209643 | 15 |
| GO:0007043 | cell-cell junction assembly | 30/3478 | 92/16992 | 0.004216795 | 30 |
| GO:0003352 | regulation of cilium movement | 7/3478 | 12/16992 | 0.004464604 | 7 |
| GO:0045727 | positive regulation of translation | 33/3478 | 104/16992 | 0.004494673 | 33 |
| GO:0021904 | dorsal/ventral neural tube patterning | 11/3478 | 24/16992 | 0.004543992 | 11 |
| GO:0044818 | mitotic G2/M transition checkpoint | 11/3478 | 24/16992 | 0.004543992 | 11 |
| GO:0046856 | phosphatidylinositol dephosphorylation | 11/3478 | 24/16992 | 0.004543992 | 11 |
| GO:0048524 | positive regulation of viral process | 34/3478 | 108/16992 | 0.004559409 | 34 |
| GO:0061515 | myeloid cell development | 20/3478 | 55/16992 | 0.00464412 | 20 |
| GO:1904356 | regulation of telomere maintenance via telomere lengthening | 20/3478 | 55/16992 | 0.00464412 | 20 |
| GO:0019827 | stem cell population maintenance | 44/3478 | 148/16992 | 0.004657084 | 44 |
| GO:0033046 | negative regulation of sister chromatid segregation | 14/3478 | 34/16992 | 0.004761802 | 14 |
| GO:0035196 | production of miRNAs involved in gene silencing by miRNA | 14/3478 | 34/16992 | 0.004761802 | 14 |
| GO:0060323 | head morphogenesis | 14/3478 | 34/16992 | 0.004761802 | 14 |

| ID | Description | GeneRatio | BgRatio | P-value | Count |
|---|---|---|---|---|---|
| hsa04120 | Ubiquitin mediated proteolysis | 61/1507 | 137/7528 | 4.84E-11 | 61 |
| hsa04141 | Protein processing in endoplasmic reticulum | 60/1507 | 165/7528 | 6.10E-07 | 60 |
| hsa05211 | Renal cell carcinoma | 32/1507 | 69/7528 | 6.48E-07 | 32 |
| hsa04012 | ErbB signaling pathway | 36/1507 | 85/7528 | 1.99E-06 | 36 |
| hsa04510 | Focal adhesion | 67/1507 | 199/7528 | 3.31E-06 | 67 |
| hsa04722 | Neurotrophin signaling pathway | 45/1507 | 119/7528 | 4.68E-06 | 45 |
| hsa05231 | Choline metabolism in cancer | 39/1507 | 99/7528 | 6.42E-06 | 39 |
| hsa00310 | Lysine degradation | 27/1507 | 59/7528 | 6.62E-06 | 27 |
| hsa04520 | Adherens junction | 31/1507 | 72/7528 | 6.74E-06 | 31 |
| hsa03013 | RNA transport | 57/1507 | 165/7528 | 7.37E-06 | 57 |
| hsa04360 | Axon guidance | 61/1507 | 181/7528 | 8.74E-06 | 61 |
| hsa04144 | Endocytosis | 77/1507 | 244/7528 | 9.84E-06 | 77 |
| hsa04810 | Regulation of actin cytoskeleton | 69/1507 | 214/7528 | 1.28E-05 | 69 |
| hsa04140 | Autophagy - animal | 46/1507 | 128/7528 | 1.76E-05 | 46 |
| hsa05205 | Proteoglycans in cancer | 65/1507 | 201/7528 | 2.05E-05 | 65 |
| hsa04071 | Sphingolipid signaling pathway | 43/1507 | 119/7528 | 2.81E-05 | 43 |
| hsa04340 | Hedgehog signaling pathway | 22/1507 | 47/7528 | 3.04E-05 | 22 |

| | | | | | |
|---|---|---|---|---|---|
| hsa05212 | Pancreatic cancer | 30/1507 | 75/7528 | 5.25E-05 | 30 |
| hsa03018 | RNA degradation | 31/1507 | 79/7528 | 6.12E-05 | 31 |
| hsa04110 | Cell cycle | 43/1507 | 124/7528 | 8.62E-05 | 43 |
| hsa01521 | EGFR tyrosine kinase inhibitor resistance | 30/1507 | 79/7528 | 0.000161302 | 30 |
| hsa03420 | Nucleotide excision repair | 20/1507 | 47/7528 | 0.000347605 | 20 |
| hsa05166 | Human T-cell leukemia virus 1 infection | 65/1507 | 219/7528 | 0.000350007 | 65 |
| hsa05210 | Colorectal cancer | 31/1507 | 86/7528 | 0.000375163 | 31 |
| hsa05213 | Endometrial cancer | 23/1507 | 58/7528 | 0.000444601 | 23 |
| hsa05220 | Chronic myeloid leukemia | 28/1507 | 76/7528 | 0.000472067 | 28 |
| hsa03022 | Basal transcription factors | 19/1507 | 45/7528 | 0.000547291 | 19 |
| hsa04931 | Insulin resistance | 36/1507 | 108/7528 | 0.00074185 | 36 |
| hsa04919 | Thyroid hormone signaling pathway | 38/1507 | 116/7528 | 0.000782222 | 38 |
| hsa04611 | Platelet activation | 40/1507 | 124/7528 | 0.000812369 | 40 |
| hsa04068 | FoxO signaling pathway | 42/1507 | 132/7528 | 0.000833149 | 42 |
| hsa04015 | Rap1 signaling pathway | 60/1507 | 206/7528 | 0.000981904 | 60 |
| hsa04150 | mTOR signaling pathway | 47/1507 | 153/7528 | 0.000994651 | 47 |
| hsa05131 | Shigellosis | 24/1507 | 65/7528 | 0.001130143 | 24 |
| hsa04070 | Phosphatidylinositol signaling system | 33/1507 | 99/7528 | 0.001211225 | 33 |
| hsa04710 | Circadian rhythm | 14/1507 | 31/7528 | 0.001324633 | 14 |
| hsa00280 | Valine, leucine and isoleucine degradation | 19/1507 | 48/7528 | 0.00140271 | 19 |
| hsa05223 | Non-small cell lung cancer | 24/1507 | 66/7528 | 0.001444923 | 24 |
| hsa04066 | HIF-1 signaling pathway | 33/1507 | 100/7528 | 0.001467647 | 33 |
| hsa04218 | Cellular senescence | 48/1507 | 160/7528 | 0.001549223 | 48 |
| hsa04211 | Longevity regulating pathway | 30/1507 | 89/7528 | 0.001626158 | 30 |
| hsa00562 | Inositol phosphate metabolism | 26/1507 | 74/7528 | 0.001663365 | 26 |
| hsa05215 | Prostate cancer | 32/1507 | 97/7528 | 0.001729175 | 32 |
| hsa04114 | Oocyte meiosis | 39/1507 | 125/7528 | 0.001887361 | 39 |
| hsa04662 | B cell receptor signaling pathway | 25/1507 | 71/7528 | 0.001957607 | 25 |
| hsa01522 | Endocrine resistance | 32/1507 | 98/7528 | 0.002085268 | 32 |
| hsa05170 | Human immunodeficiency virus 1 infection | 60/1507 | 212/7528 | 0.002095355 | 60 |
| hsa04666 | Fc gamma R-mediated phagocytosis | 30/1507 | 91/7528 | 0.002402339 | 30 |
| hsa03430 | Mismatch repair | 11/1507 | 23/7528 | 0.002480802 | 11 |
| hsa04914 | Progesterone-mediated oocyte maturation | 32/1507 | 99/7528 | 0.002502721 | 32 |
| hsa04010 | MAPK signaling pathway | 79/1507 | 295/7528 | 0.002551331 | 79 |
| hsa05165 | Human papillomavirus infection | 87/1507 | 330/7528 | 0.002596132 | 87 |
| hsa04137 | Mitophagy - animal | 23/1507 | 65/7528 | 0.002713166 | 23 |
| hsa03460 | Fanconi anemia pathway | 20/1507 | 54/7528 | 0.002727783 | 20 |
| hsa01524 | Platinum drug resistance | 25/1507 | 73/7528 | 0.003019858 | 25 |
| hsa04910 | Insulin signaling pathway | 41/1507 | 137/7528 | 0.003454536 | 41 |
| hsa05100 | Bacterial invasion of epithelial cells | 25/1507 | 74/7528 | 0.003711946 | 25 |
| hsa02010 | ABC transporters | 17/1507 | 45/7528 | 0.004377539 | 17 |
| hsa04530 | Tight junction | 48/1507 | 170/7528 | 0.005841351 | 48 |
| hsa00072 | Synthesis and degradation of ketone bodies | 6/1507 | 10/7528 | 0.006364973 | 6 |
| hsa04660 | T cell receptor signaling pathway | 31/1507 | 101/7528 | 0.006847325 | 31 |
| hsa04392 | Hippo signaling pathway - multiple species | 12/1507 | 29/7528 | 0.006890612 | 12 |
| hsa05161 | Hepatitis B | 46/1507 | 163/7528 | 0.006936975 | 46 |

| | | | | | |
|---|---|---|---|---|---|
| hsa04014 | Ras signaling pathway | 62/1507 | 232/7528 | 0.007375259 | 62 |
| hsa05221 | Acute myeloid leukemia | 22/1507 | 66/7528 | 0.00748774 | 22 |
| hsa04917 | Prolactin signaling pathway | 23/1507 | 70/7528 | 0.007684455 | 23 |
| hsa05132 | Salmonella infection | 27/1507 | 86/7528 | 0.008070524 | 27 |
| hsa04142 | Lysosome | 36/1507 | 123/7528 | 0.008659522 | 36 |
| hsa04024 | cAMP signaling pathway | 57/1507 | 212/7528 | 0.008702241 | 57 |
| hsa04720 | Long-term potentiation | 22/1507 | 67/7528 | 0.0090918 | 22 |
| hsa04152 | AMPK signaling pathway | 35/1507 | 120/7528 | 0.010107303 | 35 |
| hsa03440 | Homologous recombination | 15/1507 | 41/7528 | 0.010113818 | 15 |
| hsa04664 | Fc epsilon RI signaling pathway | 22/1507 | 68/7528 | 0.010963133 | 22 |

Table Supplementary 7: Target genes of DEregulators

| Symbol | logFC | AveExpr | P.Value | adj.P.Val |
|---|---|---|---|---|
| CNTN6 | 3.96993595 | 2.159717104 | 3.90E-57 | 3.44E-55 |
| SH3GL3 | 3.89777528 | 1.054349378 | 2.62E-90 | 6.72E-87 |
| PTPRQ | 3.830945481 | 1.248976677 | 7.02E-84 | 7.59E-81 |
| C10orf67 | 3.623519796 | 1.168509601 | 1.81E-87 | 2.86E-84 |
| HHIP | 3.36187023 | 3.917963295 | 1.94E-31 | 3.18E-30 |
| NCKAP5 | 3.25169054 | 4.107114786 | 1.23E-62 | 1.71E-60 |
| TRHDE | 3.241400447 | 1.943838197 | 4.63E-46 | 1.94E-44 |
| PCDH15 | 3.072365691 | 1.355784377 | 7.96E-54 | 5.37E-52 |
| LRRC36 | 2.953048228 | 3.403625217 | 1.13E-48 | 5.42E-47 |
| ST8SIA6 | 2.894589651 | 1.369688534 | 4.06E-60 | 4.49E-58 |
| RSPO2 | 2.867962059 | 2.44701192 | 1.86E-37 | 4.47E-36 |
| RXFP1 | 2.809327768 | 1.917062339 | 1.23E-61 | 1.58E-59 |
| STXBP6 | 2.736092745 | 3.373133255 | 3.36E-50 | 1.83E-48 |
| C1orf87 | 2.673041808 | 1.862554939 | 1.05E-23 | 9.90E-23 |
| DNAH9 | 2.637191772 | 2.910346717 | 2.08E-18 | 1.37E-17 |
| MGC27382 | 2.626023483 | 0.795064324 | 5.52E-62 | 7.27E-60 |
| PRKG2 | 2.613590585 | 2.672340247 | 6.09E-45 | 2.38E-43 |
| CD36 | 2.575421769 | 5.935923114 | 5.06E-57 | 4.36E-55 |
| KCNT2 | 2.520863891 | 2.979638469 | 1.10E-59 | 1.15E-57 |
| NTNG1 | 2.518101283 | 2.670969864 | 2.76E-29 | 3.86E-28 |
| BMPER | 2.509633592 | 3.071570277 | 7.23E-36 | 1.58E-34 |
| TEK | 2.478572863 | 5.337073846 | 8.91E-81 | 6.10E-78 |
| ZBTB16 | 2.429336771 | 3.528012181 | 6.74E-26 | 7.55E-25 |
| TMEM132C | 2.427747737 | 1.381975989 | 1.60E-34 | 3.20E-33 |
| FAT3 | 2.398312111 | 2.736493014 | 2.95E-37 | 6.99E-36 |
| KHDRBS2 | 2.386726205 | 2.145538418 | 3.81E-29 | 5.30E-28 |
| ZNF385B | 2.381393113 | 4.669604054 | 4.18E-23 | 3.80E-22 |
| AFF3 | 2.374394563 | 4.388997994 | 2.96E-37 | 6.99E-36 |
| GRIK4 | 2.369593957 | 1.448342955 | 5.47E-49 | 2.71E-47 |
| TNXB | 2.354783843 | 6.367859065 | 9.91E-45 | 3.78E-43 |
| LAMP3 | 2.353067235 | 7.446681235 | 9.17E-50 | 4.84E-48 |
| SCN7A | 2.30800787 | 4.780820082 | 1.35E-31 | 2.24E-30 |
| LRRK2 | 2.274270189 | 6.950077312 | 1.09E-27 | 1.38E-26 |
| GPC5 | 2.259126093 | 2.137601096 | 2.32E-24 | 2.30E-23 |
| ACOXL | 2.213163864 | 3.470063023 | 3.99E-29 | 5.55E-28 |
| DACH1 | 2.208973026 | 3.910777265 | 6.97E-48 | 3.22E-46 |
| LPL | 2.199029087 | 6.487573364 | 2.58E-32 | 4.48E-31 |
| ABI3BP | 2.166074046 | 5.969867013 | 4.87E-41 | 1.49E-39 |
| HPSE2 | 2.154371707 | 1.049719838 | 4.47E-39 | 1.19E-37 |
| VWA3A | 2.150764028 | 2.230059578 | 2.62E-17 | 1.59E-16 |
| GPC3 | 2.132786284 | 5.698064171 | 3.31E-34 | 6.48E-33 |
| CDHR3 | 2.124931778 | 4.126360977 | 9.56E-15 | 4.81E-14 |
| NTRK2 | 2.107465188 | 3.976746985 | 1.41E-26 | 1.65E-25 |
| TGFBR3 | 2.102978 | 5.610294938 | 4.75E-58 | 4.43E-56 |

| | | | | |
|---|---|---|---|---|
| CNKSR2 | 2.096845017 | 1.77430588 | 3.01E-32 | 5.18E-31 |
| ITGA8 | 2.096618267 | 4.212366903 | 4.14E-28 | 5.35E-27 |
| RGS9 | 2.092335507 | 2.822550456 | 1.84E-48 | 8.77E-47 |
| ADAMTSL3 | 2.087856171 | 4.555428571 | 5.45E-43 | 1.87E-41 |
| RGS22 | 2.06881278 | 2.478865963 | 1.59E-21 | 1.30E-20 |
| CALCRL | 2.035721369 | 6.414002814 | 2.85E-71 | 7.92E-69 |
| SLIT2 | 2.026936868 | 5.423766319 | 6.29E-40 | 1.77E-38 |
| TMEM232 | 2.010855109 | 2.167423603 | 8.97E-21 | 6.98E-20 |
| PDZD2 | 1.996362559 | 5.954792777 | 3.71E-41 | 1.15E-39 |
| PHACTR1 | 1.989007652 | 3.951580621 | 4.71E-45 | 1.87E-43 |
| PREX2 | 1.971773608 | 3.202321148 | 1.35E-28 | 1.82E-27 |
| SULT1C4 | 1.960710942 | 3.185348735 | 1.78E-41 | 5.62E-40 |
| SLC1A1 | 1.957760331 | 5.685691482 | 9.88E-37 | 2.27E-35 |
| ABCA3 | 1.957227569 | 8.172641142 | 5.25E-27 | 6.30E-26 |
| ANGPT1 | 1.953055343 | 5.202381797 | 3.09E-40 | 8.91E-39 |
| SLC39A8 | 1.949704016 | 7.773743094 | 8.57E-67 | 1.63E-64 |
| SEMA6D | 1.94563883 | 4.338264525 | 1.75E-40 | 5.16E-39 |
| C7 | 1.944858428 | 6.872921881 | 2.96E-19 | 2.07E-18 |
| IL33 | 1.940180491 | 5.97069623 | 1.53E-36 | 3.49E-35 |
| PTPRT | 1.92839465 | 2.60758356 | 2.72E-12 | 1.13E-11 |
| ARHGAP6 | 1.927749479 | 4.236361576 | 1.23E-63 | 1.88E-61 |
| RP1 | 1.926476756 | 3.005412156 | 1.53E-15 | 8.15E-15 |
| ATP13A4 | 1.912474685 | 5.674105311 | 2.74E-15 | 1.44E-14 |
| LDB2 | 1.908440183 | 5.682382107 | 1.75E-80 | 1.12E-77 |
| SLIT3 | 1.896966531 | 5.812846835 | 5.10E-34 | 9.88E-33 |
| CFTR | 1.892980198 | 5.043027052 | 2.17E-14 | 1.06E-13 |
| ROBO2 | 1.888529534 | 4.178417506 | 3.01E-22 | 2.60E-21 |
| EPAS1 | 1.872772138 | 9.026133967 | 3.51E-88 | 6.54E-85 |
| ANO2 | 1.856396047 | 1.902892359 | 1.50E-38 | 3.89E-37 |
| DLC1 | 1.846044416 | 7.214570461 | 8.48E-42 | 2.72E-40 |
| SVEP1 | 1.844694173 | 5.82869954 | 2.10E-35 | 4.45E-34 |
| HMGCLL1 | 1.844644831 | 2.546893162 | 4.30E-28 | 5.55E-27 |
| CPAMD8 | 1.836755061 | 5.794140912 | 1.16E-21 | 9.63E-21 |
| SPAG6 | 1.834773213 | 3.483466951 | 5.36E-10 | 1.83E-09 |
| REEP1 | 1.831731366 | 3.665242507 | 6.64E-29 | 9.10E-28 |
| PDE1C | 1.828383517 | 2.569380283 | 7.24E-23 | 6.48E-22 |
| ALDH1A2 | 1.82160723 | 4.293212141 | 5.52E-18 | 3.52E-17 |
| PALM2-AKAP2 | 1.783471999 | 6.980695042 | 1.47E-78 | 7.94E-76 |
| DNAH12 | 1.782752531 | 2.282184918 | 2.21E-12 | 9.23E-12 |
| NTRK3 | 1.778785344 | 2.133012079 | 5.42E-27 | 6.50E-26 |
| CGNL1 | 1.753355849 | 6.567662683 | 3.18E-51 | 1.85E-49 |
| ZBBX | 1.746093896 | 2.02631719 | 2.24E-11 | 8.61E-11 |
| KANK4 | 1.734226949 | 3.733266161 | 4.72E-22 | 4.02E-21 |
| PGM5 | 1.725494753 | 4.991537183 | 1.08E-31 | 1.80E-30 |
| DTHD1 | 1.720400801 | 2.394681579 | 5.73E-16 | 3.16E-15 |
| WWC2 | 1.713356146 | 6.584240781 | 5.05E-83 | 4.51E-80 |

| | | | | |
|---|---|---|---|---|
| COL4A3 | 1.701241288 | 5.419196108 | 1.95E-16 | 1.11E-15 |
| NPNT | 1.691841352 | 7.088306109 | 1.23E-30 | 1.87E-29 |
| FGFR2 | 1.683420265 | 5.953655331 | 5.49E-28 | 7.05E-27 |
| ARHGAP31 | 1.679337818 | 6.584646421 | 1.26E-55 | 9.90E-54 |
| GBA3 | 1.673278831 | 0.906193219 | 4.36E-21 | 3.46E-20 |
| FGD5 | 1.671164357 | 6.05178927 | 1.12E-65 | 1.94E-63 |
| SMAD9 | 1.658014929 | 3.321768925 | 3.55E-27 | 4.33E-26 |
| WDR63 | 1.656870439 | 2.324722866 | 1.02E-14 | 5.12E-14 |
| ABCG2 | 1.653293019 | 4.356822585 | 4.06E-40 | 1.15E-38 |
| TNS1 | 1.636668692 | 8.52341268 | 1.85E-54 | 1.32E-52 |
| ACSS3 | 1.628957233 | 4.620994508 | 7.85E-29 | 1.07E-27 |
| THSD1 | 1.627059373 | 4.47710626 | 2.53E-61 | 3.11E-59 |
| SOX5 | 1.626282641 | 2.608402745 | 2.62E-30 | 3.93E-29 |
| LTBP4 | 1.626216846 | 7.525172762 | 1.02E-50 | 5.82E-49 |
| FRAS1 | 1.625022949 | 5.335418979 | 6.20E-22 | 5.25E-21 |
| PECAM1 | 1.593965647 | 7.259432742 | 8.01E-90 | 1.83E-86 |
| RIC3 | 1.591526366 | 3.206517611 | 2.26E-17 | 1.37E-16 |
| AHNAK | 1.582077661 | 9.504363881 | 3.13E-41 | 9.76E-40 |
| TTLL7 | 1.573116864 | 3.493399556 | 9.78E-20 | 7.06E-19 |
| RAPGEF4 | 1.568407455 | 4.364607658 | 2.39E-49 | 1.23E-47 |
| C8orf34 | 1.56524559 | 3.054449007 | 3.22E-15 | 1.68E-14 |
| AFF2 | 1.564418832 | 3.497524862 | 2.07E-17 | 1.26E-16 |
| COLEC12 | 1.540416332 | 6.421856894 | 3.70E-27 | 4.50E-26 |
| DNAH10 | 1.529730421 | 3.704418976 | 8.61E-16 | 4.68E-15 |
| HIF3A | 1.52537988 | 4.896223665 | 6.14E-13 | 2.69E-12 |
| ARMC4 | 1.52507235 | 2.864019043 | 4.49E-11 | 1.67E-10 |
| PRSS12 | 1.523139393 | 4.558035009 | 1.50E-12 | 6.35E-12 |
| ECT2L | 1.516507703 | 2.626289976 | 1.88E-15 | 9.96E-15 |
| MDGA1 | 1.502351053 | 4.557330932 | 6.78E-30 | 9.88E-29 |
| CD300LF | 1.49226034 | 5.013042677 | 3.41E-31 | 5.48E-30 |
| SLC8A3 | 1.491352815 | 1.856075567 | 9.01E-23 | 8.01E-22 |
| A2M | 1.482816862 | 9.930422024 | 5.34E-39 | 1.41E-37 |
| FBLN5 | 1.478761588 | 6.781600047 | 1.18E-37 | 2.89E-36 |
| MSRB3 | 1.473952028 | 6.093601578 | 2.80E-43 | 9.78E-42 |
| ABCA10 | 1.472054511 | 2.313292803 | 3.52E-21 | 2.82E-20 |
| LRP2BP | 1.466933234 | 4.166717327 | 1.88E-42 | 6.26E-41 |
| WDR49 | 1.465213433 | 2.247460926 | 7.73E-12 | 3.10E-11 |
| DENND2A | 1.454149331 | 5.030869123 | 2.44E-38 | 6.23E-37 |
| RTN1 | 1.44831633 | 4.710958641 | 8.04E-26 | 8.94E-25 |
| PPARG | 1.448222139 | 5.582174842 | 2.50E-22 | 2.17E-21 |
| TIE1 | 1.444086441 | 6.220864496 | 2.21E-51 | 1.31E-49 |
| ZNF423 | 1.441428267 | 4.066116936 | 4.69E-37 | 1.10E-35 |
| PRKG1 | 1.433951492 | 3.820308104 | 2.17E-25 | 2.32E-24 |
| MFSD2A | 1.428134376 | 6.508461167 | 1.02E-27 | 1.28E-26 |
| SHROOM4 | 1.427272245 | 6.091562333 | 7.48E-32 | 1.26E-30 |
| SEMA3D | 1.413528727 | 3.813136151 | 4.26E-18 | 2.73E-17 |

| | | | | |
|---|---|---|---|---|
| SPATA18 | 1.412139983 | 4.907207266 | 4.45E-15 | 2.29E-14 |
| TACC1 | 1.411854447 | 7.626417435 | 1.16E-53 | 7.68E-52 |
| PDE5A | 1.411732495 | 5.891551014 | 3.32E-49 | 1.68E-47 |
| CAT | 1.411188291 | 7.598418217 | 2.66E-66 | 4.87E-64 |
| FILIP1 | 1.409786306 | 5.053126605 | 6.71E-21 | 5.27E-20 |
| LRCH2 | 1.403167033 | 4.133693028 | 1.07E-21 | 8.92E-21 |
| AMOTL1 | 1.402772163 | 6.725619805 | 8.73E-45 | 3.36E-43 |
| ABCA6 | 1.401169107 | 4.354511721 | 2.33E-23 | 2.15E-22 |
| TLL1 | 1.400706788 | 3.678014327 | 7.26E-27 | 8.62E-26 |
| LAMA3 | 1.399436258 | 6.889725061 | 7.06E-13 | 3.08E-12 |
| PRKCE | 1.398837057 | 5.770301235 | 6.59E-73 | 2.08E-70 |
| EFEMP1 | 1.394683081 | 7.630925321 | 8.12E-32 | 1.36E-30 |
| PLCL1 | 1.389442319 | 4.433820337 | 3.97E-47 | 1.78E-45 |
| NOSTRIN | 1.386790939 | 5.188856176 | 3.25E-26 | 3.71E-25 |
| NHSL1 | 1.385705787 | 6.111396688 | 6.66E-43 | 2.27E-41 |
| EML1 | 1.384479083 | 5.403721633 | 2.15E-37 | 5.14E-36 |
| NPR3 | 1.377570721 | 4.059644221 | 1.84E-20 | 1.40E-19 |
| DNAH6 | 1.37746233 | 3.218437347 | 1.40E-11 | 5.49E-11 |
| SOBP | 1.373650584 | 4.241314536 | 4.29E-30 | 6.35E-29 |
| LIFR | 1.360502429 | 7.092045754 | 2.42E-32 | 4.19E-31 |
| NTN4 | 1.358587256 | 6.887977043 | 3.26E-34 | 6.39E-33 |
| CADM1 | 1.355430792 | 7.342078552 | 2.65E-22 | 2.30E-21 |
| SASH1 | 1.348934123 | 6.537455778 | 5.23E-60 | 5.71E-58 |
| FAM184A | 1.344348901 | 4.905915682 | 1.20E-17 | 7.43E-17 |
| SPHKAP | 1.343757561 | 0.237639553 | 3.63E-25 | 3.82E-24 |
| ROS1 | 1.336098558 | 6.550734332 | 3.32E-09 | 1.06E-08 |
| UBASH3B | 1.332660749 | 5.485636807 | 2.01E-30 | 3.03E-29 |
| RNF144B | 1.331593816 | 6.469036249 | 2.07E-44 | 7.76E-43 |
| NEDD9 | 1.329462751 | 7.86094489 | 1.53E-32 | 2.69E-31 |
| PHLDB2 | 1.316350805 | 6.106075163 | 2.54E-29 | 3.56E-28 |
| RELN | 1.31634048 | 3.232586941 | 7.14E-12 | 2.87E-11 |
| MAPK10 | 1.315962247 | 4.164693262 | 7.21E-18 | 4.54E-17 |
| RBMS3 | 1.315562524 | 3.526427516 | 1.13E-24 | 1.14E-23 |
| GUCY1A2 | 1.309378497 | 3.0777312 | 3.23E-28 | 4.21E-27 |
| LIMCH1 | 1.305306442 | 7.927960946 | 2.50E-35 | 5.26E-34 |
| PLCE1 | 1.304972624 | 5.424302884 | 9.55E-26 | 1.06E-24 |
| ADARB1 | 1.301961716 | 6.092157503 | 8.23E-49 | 4.03E-47 |
| FAR2 | 1.298429748 | 4.244656815 | 6.93E-24 | 6.63E-23 |
| LRRIQ1 | 1.287878031 | 3.089243938 | 6.69E-10 | 2.26E-09 |
| RMST | 1.285304379 | 0.29281824 | 5.53E-30 | 8.12E-29 |
| AKAP12 | 1.284650879 | 6.485235957 | 2.53E-17 | 1.53E-16 |
| SORBS1 | 1.284200195 | 6.090141068 | 2.30E-33 | 4.24E-32 |
| FLI1 | 1.282291676 | 6.047064653 | 1.87E-46 | 8.03E-45 |
| KIF19 | 1.281512235 | 3.141401446 | 5.19E-09 | 1.62E-08 |
| FRY | 1.273922475 | 6.297614202 | 7.08E-36 | 1.55E-34 |
| DOCK4 | 1.270542292 | 6.297848902 | 3.05E-50 | 1.68E-48 |

| | | | | |
|---|---|---|---|---|
| DOK6 | 1.267669183 | 3.923318218 | 1.05E-20 | 8.16E-20 |
| KIF6 | 1.259117682 | 2.7437387 | 1.27E-11 | 4.99E-11 |
| FAT4 | 1.258423505 | 5.574652915 | 1.42E-23 | 1.33E-22 |
| RYR2 | 1.252843985 | 3.65249827 | 2.06E-15 | 1.09E-14 |
| NFIX | 1.247727621 | 7.224313915 | 1.55E-27 | 1.93E-26 |
| SPTBN1 | 1.236687956 | 9.013490849 | 2.56E-62 | 3.43E-60 |
| RUNX1T1 | 1.23459716 | 3.12815859 | 9.36E-19 | 6.30E-18 |
| CACHD1 | 1.218530547 | 5.714793803 | 6.84E-15 | 3.47E-14 |
| CCDC69 | 1.218216127 | 6.800563388 | 1.12E-34 | 2.27E-33 |
| PAPSS2 | 1.215578034 | 7.349382959 | 4.58E-33 | 8.24E-32 |
| TBX5 | 1.206704454 | 5.015805557 | 1.45E-24 | 1.45E-23 |
| WDR17 | 1.205378896 | 3.252032633 | 9.80E-12 | 3.89E-11 |
| NEXN | 1.20508229 | 5.389137812 | 1.74E-31 | 2.86E-30 |
| PLEKHH2 | 1.203076565 | 5.779513752 | 4.72E-22 | 4.02E-21 |
| SPI1 | 1.203072595 | 6.635743515 | 3.08E-28 | 4.04E-27 |
| RASSF8 | 1.202460575 | 5.971456318 | 7.00E-27 | 8.32E-26 |
| APOLD1 | 1.199504115 | 5.953360027 | 5.56E-30 | 8.16E-29 |
| PHACTR2 | 1.196377561 | 6.599361688 | 1.00E-50 | 5.74E-49 |
| CDKL5 | 1.188038073 | 3.433748584 | 4.60E-20 | 3.41E-19 |
| RASL12 | 1.175170256 | 5.494673395 | 7.02E-33 | 1.25E-31 |
| RFX2 | 1.174841206 | 5.381205963 | 1.91E-27 | 2.36E-26 |
| PTPRM | 1.174596929 | 6.812465997 | 6.10E-35 | 1.25E-33 |
| PHEX | 1.173537785 | 2.168494442 | 1.75E-12 | 7.37E-12 |
| PTPRD | 1.170483013 | 4.58735609 | 1.15E-18 | 7.66E-18 |
| DLEC1 | 1.164766111 | 3.589743361 | 2.01E-08 | 5.99E-08 |
| SLC22A3 | 1.160315962 | 6.098561831 | 1.20E-09 | 3.96E-09 |
| ARHGEF6 | 1.159445351 | 6.189853444 | 1.21E-34 | 2.44E-33 |
| CXorf36 | 1.15931685 | 5.570970066 | 5.39E-36 | 1.19E-34 |
| DOCK11 | 1.158371885 | 6.066285534 | 3.80E-30 | 5.65E-29 |
| ETV1 | 1.158299709 | 6.479690393 | 1.20E-20 | 9.21E-20 |
| SLC7A7 | 1.151634031 | 6.549313302 | 1.64E-30 | 2.50E-29 |
| PTPN13 | 1.150348939 | 6.867292046 | 3.55E-13 | 1.59E-12 |
| RASGEF1B | 1.148072734 | 5.679307416 | 8.32E-41 | 2.49E-39 |
| PDE4C | 1.147012365 | 4.372817805 | 9.09E-13 | 3.93E-12 |
| MYH10 | 1.132097163 | 7.634494986 | 1.65E-33 | 3.06E-32 |
| DLGAP1 | 1.121560635 | 2.076122632 | 5.21E-07 | 1.36E-06 |
| SECISBP2L | 1.120873583 | 7.25292549 | 1.84E-41 | 5.80E-40 |
| NAALAD2 | 1.120800823 | 2.610117835 | 3.25E-23 | 2.97E-22 |
| THRB | 1.118872349 | 5.046001248 | 3.84E-16 | 2.14E-15 |
| CDH10 | 1.117096512 | 0.552994624 | 2.79E-13 | 1.26E-12 |
| PPIL6 | 1.110563364 | 4.389017042 | 1.17E-17 | 7.23E-17 |
| RAB11FIP1 | 1.109355824 | 8.448848939 | 3.40E-33 | 6.21E-32 |
| LMO7 | 1.109161613 | 8.486739698 | 1.10E-34 | 2.22E-33 |
| EPB41L2 | 1.104041497 | 6.738901145 | 5.19E-34 | 1.00E-32 |
| UTRN | 1.095818551 | 7.657523148 | 4.22E-40 | 1.20E-38 |
| NEK10 | 1.090841803 | 2.053254548 | 7.20E-08 | 2.04E-07 |

| | | | | |
|---|---|---|---|---|
| SULT1A1 | 1.081927164 | 6.261814589 | 5.59E-23 | 5.03E-22 |
| ABCC9 | 1.081628128 | 5.429485405 | 6.78E-20 | 4.95E-19 |
| CAB39L | 1.08073994 | 5.490321232 | 3.55E-38 | 8.91E-37 |
| HYDIN | 1.078372953 | 4.241493969 | 2.82E-08 | 8.28E-08 |
| CCDC39 | 1.07472068 | 3.162740349 | 3.84E-13 | 1.71E-12 |
| CRIM1 | 1.073059615 | 7.377804753 | 2.54E-33 | 4.68E-32 |
| DNAH7 | 1.072056108 | 3.363121612 | 6.86E-10 | 2.32E-09 |
| GAB1 | 1.068748369 | 6.170163002 | 1.63E-48 | 7.78E-47 |
| P2RX7 | 1.068325503 | 4.275924087 | 4.54E-15 | 2.33E-14 |
| PRDM5 | 1.067578961 | 3.734229028 | 3.32E-23 | 3.03E-22 |
| FBLN1 | 1.066650564 | 7.969050453 | 1.15E-20 | 8.88E-20 |
| ST6GAL2 | 1.066125677 | 3.008201338 | 5.29E-08 | 1.52E-07 |
| VNN3 | 1.062387537 | 2.398044708 | 4.90E-07 | 1.29E-06 |
| SYNE1 | 1.061686308 | 7.390166826 | 4.10E-21 | 3.26E-20 |
| WDR78 | 1.061557467 | 4.210348169 | 2.70E-12 | 1.12E-11 |
| ZEB1 | 1.061051693 | 6.10035385 | 4.95E-36 | 1.10E-34 |
| TSPAN12 | 1.059409112 | 6.677532343 | 2.56E-26 | 2.95E-25 |
| NLGN4X | 1.058863239 | 3.305224156 | 3.14E-11 | 1.19E-10 |
| CCDC68 | 1.058628737 | 5.239188707 | 4.21E-17 | 2.50E-16 |
| TTN | 1.048581304 | 4.946747368 | 5.30E-24 | 5.13E-23 |
| DST | 1.047420573 | 7.62202514 | 3.17E-19 | 2.21E-18 |
| ITGAL | 1.043691748 | 6.554623488 | 1.11E-19 | 7.97E-19 |
| NFAM1 | 1.041363166 | 5.700256395 | 5.27E-20 | 3.88E-19 |
| FERMT2 | 1.040884094 | 6.850004968 | 4.16E-47 | 1.86E-45 |
| MYLK | 1.040281593 | 7.50847052 | 4.05E-24 | 3.95E-23 |
| EPB41L3 | 1.039222467 | 6.153891686 | 4.96E-21 | 3.92E-20 |
| SLC34A2 | 1.036656149 | 10.11097705 | 1.09E-07 | 3.03E-07 |
| NR3C2 | 1.036446841 | 5.587432153 | 3.02E-14 | 1.47E-13 |
| RNF180 | 1.031775255 | 4.490220445 | 1.11E-17 | 6.92E-17 |
| CORIN | 1.027884636 | 3.680504537 | 7.98E-12 | 3.19E-11 |
| NLGN1 | 1.021527298 | 1.594019469 | 4.57E-08 | 1.32E-07 |
| MOCS1 | 1.021518287 | 5.697818759 | 2.30E-28 | 3.04E-27 |
| IQUB | 1.017573263 | 2.33873066 | 1.47E-10 | 5.24E-10 |
| NCF1 | 1.015173818 | 5.190005292 | 9.19E-16 | 4.98E-15 |
| 10-Mar | 1.014160983 | 2.425523725 | 1.52E-07 | 4.19E-07 |
| BCO2 | 1.011922477 | 2.772312603 | 1.32E-12 | 5.63E-12 |
| FRMD4A | 1.003345311 | 5.907218704 | 2.56E-41 | 8.05E-40 |
| SNX25 | 1.002984437 | 6.452143727 | 3.64E-28 | 4.71E-27 |
| PARD3B | 1.002413335 | 3.909376609 | 7.31E-14 | 3.45E-13 |
| ZFYVE9 | 1.000267067 | 5.978318859 | 3.56E-36 | 7.96E-35 |
| SGPP2 | -1.005227913 | 5.860898375 | 1.27E-14 | 6.35E-14 |
| NLN | -1.024693865 | 5.921883949 | 7.95E-44 | 2.89E-42 |
| SEMA4B | -1.041037773 | 8.103641975 | 3.80E-31 | 6.10E-30 |
| MSI2 | -1.044183432 | 5.461903631 | 3.28E-43 | 1.14E-41 |
| PDK1 | -1.054484666 | 6.173533237 | 7.48E-35 | 1.52E-33 |
| SRPK1 | -1.056769939 | 7.373495523 | 2.17E-68 | 4.80E-66 |

| | | | | |
|---|---|---|---|---|
| EPCAM | -1.060823575 | 9.015236061 | 3.73E-41 | 1.15E-39 |
| MPP6 | -1.062398855 | 4.358934701 | 5.35E-20 | 3.94E-19 |
| NCAPG2 | -1.108249561 | 6.129494626 | 2.46E-33 | 4.53E-32 |
| XPR1 | -1.116775093 | 7.85806375 | 1.10E-33 | 2.07E-32 |
| ATAD2 | -1.122163414 | 6.612502943 | 6.17E-32 | 1.04E-30 |
| RFC4 | -1.123563674 | 5.604879977 | 3.42E-35 | 7.17E-34 |
| MYO19 | -1.131996548 | 6.657134946 | 5.56E-58 | 5.17E-56 |
| TMEM177 | -1.136740669 | 5.290272774 | 8.60E-74 | 2.85E-71 |
| RALGPS2 | -1.139653327 | 6.025706382 | 2.99E-35 | 6.27E-34 |
| WDHD1 | -1.158027921 | 5.330637746 | 2.34E-31 | 3.81E-30 |
| PDIA4 | -1.158155032 | 9.004354108 | 3.03E-56 | 2.53E-54 |
| ATAD5 | -1.192720578 | 4.474105568 | 6.27E-32 | 1.06E-30 |
| NUP210L | -1.204374828 | 2.107633281 | 6.23E-08 | 1.77E-07 |
| HAL | -1.209660903 | 3.630144208 | 2.59E-07 | 6.97E-07 |
| RAD54B | -1.244923003 | 4.433277148 | 1.15E-36 | 2.64E-35 |
| KIAA1524 | -1.245321408 | 4.753930487 | 1.62E-24 | 1.63E-23 |
| COL5A1 | -1.256145879 | 8.300951339 | 5.95E-22 | 5.04E-21 |
| SLC35F2 | -1.258873034 | 6.562767771 | 1.17E-44 | 4.43E-43 |
| C1orf112 | -1.259308227 | 5.080988639 | 1.10E-55 | 8.75E-54 |
| TNFRSF21 | -1.280101228 | 8.08968892 | 6.86E-44 | 2.51E-42 |
| CCNF | -1.341128846 | 5.683207035 | 5.41E-48 | 2.51E-46 |
| FANCI | -1.376813273 | 6.212669083 | 4.55E-50 | 2.45E-48 |
| HSF2BP | -1.385197473 | 2.907672237 | 5.62E-28 | 7.21E-27 |
| DNAH14 | -1.397814391 | 4.538060537 | 6.28E-55 | 4.62E-53 |
| ADAM28 | -1.409231077 | 5.669383188 | 3.25E-25 | 3.44E-24 |
| TRAIP | -1.417649212 | 4.253022728 | 3.41E-50 | 1.85E-48 |
| COL24A1 | -1.42799349 | 3.503135605 | 9.52E-31 | 1.47E-29 |
| CCNE2 | -1.434359893 | 4.794999243 | 9.48E-32 | 1.58E-30 |
| FAP | -1.476529814 | 5.877912656 | 2.58E-28 | 3.39E-27 |
| PABPC1L | -1.479043238 | 5.75791053 | 5.74E-25 | 5.93E-24 |
| CATSPERB | -1.490252446 | 2.656159794 | 1.17E-11 | 4.61E-11 |
| ABCC3 | -1.509645068 | 8.024710548 | 4.39E-26 | 4.97E-25 |
| MYO7A | -1.510485505 | 5.460933723 | 3.90E-50 | 2.10E-48 |
| KNTC1 | -1.519316196 | 5.923803382 | 2.64E-52 | 1.64E-50 |
| CENPK | -1.53906261 | 4.324484995 | 1.09E-37 | 2.67E-36 |
| DNA2 | -1.571356178 | 4.667128344 | 9.23E-49 | 4.49E-47 |
| SHCBP1 | -1.575708703 | 5.017032222 | 3.75E-42 | 1.23E-40 |
| WDR62 | -1.584736343 | 4.897374662 | 3.87E-32 | 6.61E-31 |
| PRR11 | -1.610288631 | 4.0256366 | 1.01E-23 | 9.53E-23 |
| AURKA | -1.644667102 | 5.733486127 | 1.88E-41 | 5.91E-40 |
| COL3A1 | -1.649717044 | 10.10954592 | 4.06E-30 | 6.01E-29 |
| IGF2BP3 | -1.724490353 | 4.976088676 | 5.21E-14 | 2.49E-13 |
| GOLM1 | -1.727638238 | 8.432090804 | 1.02E-69 | 2.47E-67 |
| NUSAP1 | -1.733735325 | 6.016329746 | 2.53E-53 | 1.66E-51 |
| KIF23 | -1.742218097 | 5.410065532 | 1.07E-40 | 3.18E-39 |
| LGR4 | -1.755930271 | 6.903777769 | 8.12E-55 | 5.95E-53 |

| | | | | |
|---|---|---|---|---|
| EZH2 | -1.808877538 | 5.550975326 | 1.84E-64 | 3.03E-62 |
| KIF11 | -1.814630125 | 5.874872343 | 1.26E-54 | 9.07E-53 |
| BUB1 | -1.844828959 | 5.641485651 | 5.08E-43 | 1.75E-41 |
| GREB1L | -1.85029131 | 2.417070753 | 5.79E-17 | 3.40E-16 |
| HELLS | -1.909520193 | 4.447226479 | 3.54E-55 | 2.68E-53 |
| COL1A1 | -1.956268976 | 10.34462342 | 5.65E-35 | 1.17E-33 |
| POLE2 | -1.972778819 | 3.95981905 | 1.73E-46 | 7.45E-45 |
| KCNN4 | -2.006857052 | 6.263026874 | 1.27E-29 | 1.82E-28 |
| KIF15 | -2.10442948 | 4.445100849 | 4.05E-49 | 2.04E-47 |
| CENPI | -2.150558734 | 3.395275148 | 2.73E-40 | 7.92E-39 |
| NCAPH | -2.150973305 | 5.121451133 | 5.77E-54 | 3.96E-52 |
| KIF26B | -2.151927266 | 5.680347093 | 3.43E-57 | 3.03E-55 |
| POLQ | -2.181524766 | 4.141628869 | 4.23E-49 | 2.13E-47 |
| NCAPG | -2.227142828 | 5.369764782 | 4.24E-57 | 3.72E-55 |
| SKA3 | -2.257086083 | 4.403614905 | 2.02E-58 | 1.94E-56 |
| BUB1B | -2.51678771 | 5.176475366 | 1.10E-68 | 2.46E-66 |
| TOP2A | -2.603588505 | 7.224343128 | 7.71E-71 | 2.06E-68 |
| NEIL3 | -2.65293088 | 3.493331976 | 1.60E-46 | 6.88E-45 |
| KIF4A | -2.847595724 | 5.314585562 | 5.86E-68 | 1.27E-65 |
| STRA6 | -3.040757701 | 4.384992655 | 1.08E-36 | 2.49E-35 |

Table Supplementary 8: KEGG and GO analysis of target genes

| Category | Description | LogP | Enrichment | Z-score | GeneInGO |
|---|---|---|---|---|---|
| GO Biological Processes | apoptosis | -35 | 3.2 | 5 | 300 |
| GO Biological Processes | cell cycle checkpoint | -35 | 1.9 | 4.7 | 216 |
| GO Biological Processes | cell growth | -35 | 1.6 | 4.8 | 479 |
| GO Biological Processes | cell-cell signaling by wnt | -34.4 | 1.5 | 4.3 | 471 |
| GO Biological Processes | lung epithelial cell differentiation | -32.1 | 2.9 | 3.1 | 21 |
| GO Biological Processes | lung epithelium development | -32.8 | 2.7 | 3.7 | 35 |
| GO Biological Processes | lung lobe development | -32.3 | 4.9 | 3.8 | 7 |
| GO Biological Processes | lung-associated mesenchyme development | -32.1 | 4.3 | 3.4 | 8 |
| GO Biological Processes | ERK1 and ERK2 cascade | -28 | 2 | 6.3 | 320 |
| GO Biological Processes | epithelial cell migration | -25.1 | 1.6 | 4.1 | 348 |
| GO Biological Processes | epithelial to mesenchymal transition | -25 | 1.8 | 3.5 | 137 |
| GO Biological Processes | calcitonin family receptor signaling pathway | -25 | 4.9 | 3.8 | 7 |
| GO Biological Processes | lung lobe morphogenesis | -25 | 4.9 | 3.8 | 7 |
| GO Biological Processes | extracellular structure organization | -25 | 2.6 | 12 | 414 |
| GO Biological Processes | extracellular matrix organization | -23 | 2.7 | 12 | 359 |
| GO Biological Processes | regulation of ion transport | -21 | 2.1 | 11 | 668 |
| GO Biological Processes | tissue morphogenesis | -20 | 2.2 | 11 | 620 |
| GO Biological Processes | blood vessel development | -20 | 2 | 10 | 763 |
| GO Biological Processes | response to wounding | -19 | 2.1 | 10 | 671 |
| GO Biological Processes | vasculature development | -19 | 2 | 9.9 | 797 |
| GO Biological Processes | blood vessel morphogenesis | -18 | 2 | 9.8 | 684 |
| GO Biological Processes | urogenital system development | -17 | 2.5 | 9.9 | 327 |
| GO Biological Processes | chemotaxis | -16 | 2 | 9.3 | 632 |
| GO Biological Processes | wound healing | -16 | 2.1 | 9.3 | 556 |
| GO Biological Processes | renal system development | -16 | 2.6 | 9.7 | 290 |
| GO Biological Processes | taxis | -16 | 2 | 9.2 | 635 |
| GO Biological Processes | embryonic morphogenesis | -15 | 2 | 8.9 | 577 |
| GO Biological Processes | kidney development | -15 | 2.5 | 9.3 | 274 |
| GO Biological Processes | behavior | -15 | 2 | 8.9 | 590 |
| GO Biological Processes | angiogenesis | -15 | 2 | 8.9 | 592 |
| GO Biological Processes | cell-cell adhesion via plasma-membrane adhesion molecules | -15 | 2.5 | 9.3 | 276 |
| GO Biological Processes | regulation of transmembrane transport | -15 | 2 | 8.8 | 542 |
| GO Biological Processes | regulation of signaling receptor activity | -15 | 2 | 8.7 | 558 |
| GO Biological Processes | morphogenesis of an epithelium | -14 | 2.1 | 8.7 | 484 |
| GO Biological Processes | circulatory system process | -14 | 2 | 8.6 | 506 |
| GO Biological Processes | heart morphogenesis | -14 | 2.5 | 8.9 | 256 |
| GO Biological Processes | blood circulation | -14 | 2 | 8.5 | 497 |
| GO Biological Processes | skeletal system development | -14 | 2 | 8.5 | 515 |
| GO Biological Processes | muscle structure development | -14 | 1.9 | 8.3 | 660 |
| GO Biological Processes | regulation of system process | -14 | 2 | 8.4 | 574 |
| GO Biological Processes | regulation of ion transmembrane transport | -13 | 2.1 | 8.4 | 456 |
| GO Biological Processes | cell-substrate adhesion | -12 | 2.2 | 8.2 | 343 |
| GO Biological Processes | cell morphogenesis involved in differentiation | -12 | 1.8 | 7.8 | 727 |

| | | | | | |
|---|---|---|---|---|---|
| GO Biological Processes | muscle tissue development | -12 | 2.1 | 8 | 408 |
| GO Biological Processes | negative regulation of response to external stimulus | -12 | 2.2 | 8.1 | 346 |
| GO Biological Processes | regulation of body fluid levels | -12 | 2 | 7.9 | 501 |
| GO Biological Processes | muscle contraction | -12 | 2.2 | 8 | 354 |
| GO Biological Processes | regulation of secretion | -12 | 1.7 | 7.7 | 785 |
| GO Biological Processes | mitotic nuclear division | -12 | 2.3 | 8.1 | 282 |
| GO Biological Processes | head development | -12 | 1.7 | 7.7 | 768 |
| GO Biological Processes | heart development | -12 | 1.9 | 7.7 | 573 |
| GO Biological Processes | regulation of secretion by cell | -12 | 1.8 | 7.6 | 726 |
| GO Biological Processes | ion homeostasis | -12 | 1.7 | 7.6 | 799 |
| GO Biological Processes | brain development | -12 | 1.8 | 7.6 | 722 |
| GO Biological Processes | muscle system process | -12 | 2 | 7.7 | 461 |
| GO Biological Processes | developmental growth | -11 | 1.8 | 7.4 | 655 |
| GO Biological Processes | positive regulation of ion transport | -11 | 2.3 | 7.8 | 265 |
| GO Biological Processes | striated muscle tissue development | -11 | 2 | 7.6 | 391 |
| GO Biological Processes | digestive tract development | -11 | 2.9 | 8.1 | 132 |
| GO Biological Processes | multicellular organismal homeostasis | -11 | 1.9 | 7.5 | 476 |
| GO Biological Processes | pattern specification process | -11 | 2 | 7.5 | 434 |
| GO Biological Processes | adenylate cyclase-modulating G protein-coupled receptor signaling pathway | -11 | 2.4 | 7.6 | 222 |
| GO Biological Processes | inorganic ion homeostasis | -11 | 1.7 | 7.2 | 731 |
| GO Biological Processes | epithelial tube morphogenesis | -10 | 2.1 | 7.4 | 313 |
| GO Biological Processes | nuclear division | -10 | 2 | 7.2 | 421 |
| GO Biological Processes | cation homeostasis | -10 | 1.7 | 7 | 720 |
| GO Biological Processes | cell projection morphogenesis | -10 | 1.7 | 7 | 655 |
| GO Biological Processes | digestive system development | -10 | 2.8 | 7.6 | 144 |
| GO Biological Processes | regulation of hormone levels | -10 | 1.8 | 7.1 | 522 |
| GO Biological Processes | regulation of cell-substrate adhesion | -10 | 2.4 | 7.4 | 212 |
| GO Biological Processes | neuron projection morphogenesis | -10 | 1.8 | 7 | 639 |
| GO Biological Processes | plasma membrane bounded cell projection morphogenesis | -10 | 1.7 | 7 | 653 |
| GO Biological Processes | metal ion homeostasis | -10 | 1.7 | 6.9 | 654 |
| GO Biological Processes | positive regulation of secretion by cell | -9.9 | 2 | 7.1 | 379 |
| GO Biological Processes | organic hydroxy compound transport | -9.9 | 2.2 | 7.2 | 258 |
| GO Biological Processes | second-messenger-mediated signaling | -9.9 | 1.9 | 7 | 441 |
| GO Biological Processes | cellular response to growth factor stimulus | -9.8 | 1.7 | 6.9 | 703 |
| GO Biological Processes | regulation of response to wounding | -9.8 | 2.6 | 7.4 | 168 |
| GO Biological Processes | organelle fission | -9.7 | 1.9 | 6.9 | 463 |
| GO Biological Processes | cardiac chamber morphogenesis | -9.6 | 2.8 | 7.4 | 129 |
| GO Biological Processes | cell part morphogenesis | -9.6 | 1.7 | 6.8 | 674 |
| GO Biological Processes | regulation of metal ion transport | -9.6 | 2 | 7 | 378 |
| GO Biological Processes | response to growth factor | -9.6 | 1.7 | 6.8 | 734 |
| GO Biological Processes | organic anion transport | -9.6 | 1.9 | 6.9 | 464 |
| GO Biological Processes | positive regulation of secretion | -9.6 | 1.9 | 6.9 | 409 |
| GO Biological Processes | negative regulation of response to wounding | -9.5 | 3.2 | 7.6 | 88 |
| GO Biological Processes | regulation of membrane potential | -9.5 | 1.9 | 6.9 | 429 |
| GO Biological Processes | embryonic organ development | -9.5 | 1.9 | 6.9 | 423 |

| | | | | | |
|---|---|---|---|---|---|
| GO Biological Processes | regulation of growth | -9.5 | 1.7 | 6.7 | 691 |
| GO Biological Processes | negative regulation of cell differentiation | -9.4 | 1.7 | 6.7 | 738 |
| GO Biological Processes | regulation of mitotic nuclear division | -9.4 | 2.5 | 7.2 | 182 |
| GO Biological Processes | regulation of wound healing | -9.4 | 2.7 | 7.3 | 141 |
| GO Biological Processes | nuclear chromosome segregation | -9.4 | 2.2 | 7 | 265 |
| GO Biological Processes | anion transport | -9.3 | 1.7 | 6.7 | 597 |
| GO Biological Processes | embryonic organ morphogenesis | -9.3 | 2.1 | 6.9 | 283 |
| GO Biological Processes | regulation of neurotransmitter levels | -9.3 | 2 | 6.9 | 342 |
| GO Biological Processes | cilium movement | -9.3 | 3.6 | 7.6 | 64 |
| GO Biological Processes | regulated exocytosis | -9.2 | 1.6 | 6.6 | 790 |
| GO Biological Processes | chromosome segregation | -9.2 | 2.1 | 6.9 | 319 |
| GO Biological Processes | regulation of neurogenesis | -9.2 | 1.6 | 6.6 | 798 |
| GO Biological Processes | synaptic signaling | -9.2 | 1.7 | 6.6 | 705 |
| GO Biological Processes | negative regulation of cell proliferation | -9.2 | 1.6 | 6.6 | 765 |
| GO Biological Processes | G protein-coupled receptor signaling pathway, coupled to cyclic nucleotide second messenger | -9.1 | 2.2 | 6.9 | 257 |
| GO Biological Processes | axon development | -9.1 | 1.8 | 6.7 | 505 |
| GO Biological Processes | cellular ion homeostasis | -9.1 | 1.7 | 6.6 | 648 |
| GO Biological Processes | regulation of hemostasis | -9 | 3.3 | 7.4 | 78 |
| GO Biological Processes | sensory organ development | -9 | 1.8 | 6.6 | 532 |
| GO Biological Processes | lipid transport | -9 | 2 | 6.7 | 358 |
| GO Biological Processes | cellular cation homeostasis | -9 | 1.7 | 6.5 | 636 |
| GO Biological Processes | response to purine-containing compound | -9 | 2.6 | 7.1 | 150 |
| GO Biological Processes | cardiac chamber development | -8.9 | 2.5 | 7 | 166 |
| GO Biological Processes | epithelial cell differentiation | -8.9 | 1.6 | 6.5 | 771 |
| GO Biological Processes | forebrain development | -8.9 | 1.9 | 6.7 | 378 |
| GO Biological Processes | muscle organ development | -8.9 | 1.9 | 6.6 | 409 |
| GO Biological Processes | cellular metal ion homeostasis | -8.9 | 1.7 | 6.5 | 574 |
| GO Biological Processes | trans-synaptic signaling | -8.9 | 1.7 | 6.5 | 699 |
| GO Biological Processes | regulation of peptide secretion | -8.8 | 1.8 | 6.5 | 473 |
| GO Biological Processes | regulation of cell adhesion | -8.7 | 1.7 | 6.4 | 682 |
| GO Biological Processes | positive regulation of response to external stimulus | -8.7 | 2 | 6.6 | 309 |
| GO Biological Processes | cell division | -8.7 | 1.7 | 6.4 | 591 |
| GO Biological Processes | negative regulation of wound healing | -8.7 | 3.3 | 7.2 | 76 |
| GO Biological Processes | divalent inorganic cation homeostasis | -8.6 | 1.8 | 6.4 | 508 |
| GO Biological Processes | peptide secretion | -8.6 | 1.7 | 6.4 | 593 |
| GO Biological Processes | positive regulation of peptide secretion | -8.6 | 2.1 | 6.6 | 270 |
| GO Biological Processes | hormone metabolic process | -8.5 | 2.2 | 6.7 | 225 |
| GO Biological Processes | regulation of cytosolic calcium ion concentration | -8.5 | 1.9 | 6.5 | 354 |
| GO Biological Processes | cell morphogenesis involved in neuron differentiation | -8.5 | 1.7 | 6.4 | 575 |
| GO Biological Processes | hemostasis | -8.5 | 2 | 6.5 | 342 |
| GO Biological Processes | sister chromatid segregation | -8.5 | 2.3 | 6.7 | 192 |
| GO Biological Processes | calcium ion transport | -8.5 | 1.9 | 6.4 | 422 |
| GO Biological Processes | calcium ion homeostasis | -8.5 | 1.8 | 6.4 | 466 |
| GO Biological Processes | regulation of blood coagulation | -8.5 | 3.2 | 7.1 | 77 |
| GO Biological Processes | regulation of animal organ morphogenesis | -8.5 | 2.3 | 6.7 | 209 |

| | | | | | |
|---|---|---|---|---|---|
| GO Biological Processes | gland development | -8.4 | 1.8 | 6.4 | 436 |
| GO Biological Processes | cellular divalent inorganic cation homeostasis | -8.4 | 1.8 | 6.4 | 487 |
| GO Biological Processes | mesenchyme development | -8.4 | 2.1 | 6.5 | 273 |
| GO Biological Processes | actin filament-based process | -8.4 | 1.6 | 6.2 | 738 |
| GO Biological Processes | chemical synaptic transmission | -8.4 | 1.6 | 6.2 | 691 |
| GO Biological Processes | anterograde trans-synaptic signaling | -8.4 | 1.6 | 6.2 | 691 |
| GO Biological Processes | regulation of nuclear division | -8.3 | 2.3 | 6.6 | 205 |
| GO Biological Processes | negative regulation of growth | -8.3 | 2.1 | 6.5 | 262 |
| GO Biological Processes | regulation of trans-synaptic signaling | -8.3 | 1.8 | 6.3 | 426 |
| GO Biological Processes | regulation of protein secretion | -8.3 | 1.8 | 6.3 | 445 |
| GO Biological Processes | negative regulation of hemostasis | -8.3 | 3.7 | 7.2 | 53 |
| GO Biological Processes | regulation of cation transmembrane transport | -8.3 | 2 | 6.4 | 316 |
| GO Biological Processes | cellular calcium ion homeostasis | -8.3 | 1.8 | 6.3 | 452 |
| GO Biological Processes | response to inorganic substance | -8.2 | 1.7 | 6.2 | 555 |
| GO Biological Processes | response to xenobiotic stimulus | -8.2 | 2.1 | 6.4 | 281 |
| GO Biological Processes | axon guidance | -8.2 | 2.1 | 6.4 | 270 |
| GO Biological Processes | response to metal ion | -8.2 | 1.9 | 6.3 | 360 |
| GO Biological Processes | cell chemotaxis | -8.1 | 2 | 6.4 | 300 |
| GO Biological Processes | positive regulation of cellular component movement | -8.1 | 1.7 | 6.2 | 564 |
| GO Biological Processes | vascular process in circulatory system | -8.1 | 2.4 | 6.6 | 164 |
| GO Biological Processes | neuron projection guidance | -8.1 | 2.1 | 6.4 | 271 |
| GO Biological Processes | lipid localization | -8.1 | 1.9 | 6.3 | 392 |
| GO Biological Processes | response to toxic substance | -8.1 | 1.7 | 6.2 | 519 |
| GO Biological Processes | blood coagulation | -8.1 | 1.9 | 6.3 | 337 |
| GO Biological Processes | microtubule bundle formation | -8.1 | 3 | 6.9 | 89 |
| GO Biological Processes | mitotic sister chromatid segregation | -8.1 | 2.5 | 6.6 | 154 |
| GO Biological Processes | axonogenesis | -8 | 1.8 | 6.2 | 463 |
| GO Biological Processes | modulation of chemical synaptic transmission | -8 | 1.8 | 6.2 | 425 |
| GO Biological Processes | positive regulation of locomotion | -8 | 1.7 | 6.1 | 580 |
| GO Biological Processes | drug transport | -8 | 2.2 | 6.4 | 215 |
| GO Biological Processes | extracellular matrix disassembly | -8 | 3.2 | 6.9 | 76 |
| GO Biological Processes | myeloid leukocyte migration | -8 | 2.2 | 6.4 | 204 |
| GO Biological Processes | respiratory system development | -8 | 2.3 | 6.5 | 193 |
| GO Biological Processes | positive regulation of protein secretion | -8 | 2.1 | 6.3 | 250 |
| GO Biological Processes | cardiac muscle tissue development | -7.9 | 2.1 | 6.4 | 233 |
| GO Biological Processes | negative regulation of peptidase activity | -7.9 | 2.1 | 6.3 | 262 |
| GO Biological Processes | cytokine secretion | -7.9 | 2.2 | 6.4 | 216 |
| GO Biological Processes | regionalization | -7.9 | 1.9 | 6.2 | 340 |
| GO Biological Processes | cellular chemical homeostasis | -7.9 | 1.6 | 6 | 791 |
| GO Biological Processes | peptidyl-tyrosine phosphorylation | -7.8 | 1.9 | 6.1 | 391 |
| GO Biological Processes | stem cell differentiation | -7.8 | 2.2 | 6.4 | 206 |
| GO Biological Processes | cell fate commitment | -7.8 | 2.1 | 6.3 | 258 |
| GO Biological Processes | regulation of coagulation | -7.8 | 3 | 6.7 | 82 |
| GO Biological Processes | regulation of MAPK cascade | -7.8 | 1.6 | 6 | 767 |
| GO Biological Processes | lung development | -7.8 | 2.4 | 6.4 | 168 |
| GO Biological Processes | coagulation | -7.7 | 1.9 | 6.1 | 343 |

| | | | | | |
|---|---|---|---|---|---|
| GO Biological Processes | developmental maturation | -7.7 | 2 | 6.2 | 277 |
| GO Biological Processes | response to organophosphorus | -7.7 | 2.5 | 6.4 | 137 |
| GO Biological Processes | negative regulation of blood coagulation | -7.7 | 3.6 | 6.9 | 52 |
| GO Biological Processes | peptidyl-tyrosine modification | -7.7 | 1.8 | 6.1 | 394 |
| GO Biological Processes | epithelial cell proliferation | -7.7 | 1.8 | 6 | 432 |
| GO Biological Processes | positive regulation of cytokine secretion | -7.7 | 2.6 | 6.5 | 127 |
| GO Biological Processes | neutrophil chemotaxis | -7.7 | 2.8 | 6.6 | 102 |
| GO Biological Processes | regulation of ERK1 and ERK2 cascade | -7.6 | 2 | 6.1 | 302 |
| GO Biological Processes | muscle tissue morphogenesis | -7.6 | 3 | 6.6 | 83 |
| GO Biological Processes | smooth muscle contraction | -7.6 | 2.7 | 6.5 | 107 |
| GO Biological Processes | monocarboxylic acid transport | -7.6 | 2.4 | 6.3 | 159 |
| GO Biological Processes | regulation of transporter activity | -7.6 | 2 | 6.2 | 261 |
| GO Biological Processes | positive regulation of cell motility | -7.6 | 1.7 | 5.9 | 550 |
| GO Biological Processes | positive regulation of cytosolic calcium ion concentration | -7.6 | 1.9 | 6.1 | 315 |
| GO Biological Processes | mesenchymal cell differentiation | -7.6 | 2.2 | 6.2 | 215 |
| GO Biological Processes | carboxylic acid biosynthetic process | -7.6 | 1.8 | 6 | 415 |
| GO Biological Processes | leukocyte migration | -7.5 | 1.7 | 5.9 | 486 |
| GO Biological Processes | feeding behavior | -7.5 | 2.8 | 6.5 | 103 |
| GO Biological Processes | organic acid biosynthetic process | -7.5 | 1.8 | 6 | 416 |
| GO Biological Processes | appendage development | -7.5 | 2.3 | 6.2 | 177 |
| GO Biological Processes | limb development | -7.5 | 2.3 | 6.2 | 177 |
| GO Biological Processes | respiratory tube development | -7.4 | 2.3 | 6.2 | 172 |
| GO Biological Processes | regulation of transmembrane transporter activity | -7.4 | 2.1 | 6.1 | 246 |
| GO Biological Processes | response to bacterium | -7.4 | 1.6 | 5.8 | 688 |
| GO Biological Processes | regulation of angiogenesis | -7.4 | 1.8 | 5.9 | 387 |
| GO Biological Processes | telencephalon development | -7.4 | 2 | 6 | 253 |
| GO Biological Processes | regulation of cytokine secretion | -7.3 | 2.2 | 6.1 | 190 |
| GO Biological Processes | neutrophil migration | -7.3 | 2.6 | 6.3 | 115 |
| GO Biological Processes | cellular component assembly involved in morphogenesis | -7.3 | 2.7 | 6.3 | 110 |
| GO Biological Processes | ossification | -7.3 | 1.8 | 5.9 | 383 |
| GO Biological Processes | response to lipopolysaccharide | -7.3 | 1.9 | 5.9 | 327 |
| GO Biological Processes | cardiac ventricle development | -7.2 | 2.5 | 6.2 | 126 |
| GO Biological Processes | regulation of vasculature development | -7.2 | 1.8 | 5.8 | 428 |
| GO Biological Processes | inorganic cation transmembrane transport | -7.2 | 1.6 | 5.7 | 748 |
| GO Biological Processes | antimicrobial humoral response | -7.2 | 2.6 | 6.2 | 121 |
| GO Biological Processes | stem cell proliferation | -7.2 | 2.6 | 6.3 | 116 |
| GO Biological Processes | phenol-containing compound metabolic process | -7.2 | 2.7 | 6.3 | 101 |
| GO Biological Processes | regulation of epithelial cell proliferation | -7.2 | 1.8 | 5.8 | 378 |
| GO Biological Processes | protein secretion | -7.2 | 1.7 | 5.7 | 560 |
| GO Biological Processes | gliogenesis | -7.2 | 2 | 5.9 | 274 |
| GO Biological Processes | regulation of protein kinase activity | -7.1 | 1.6 | 5.7 | 778 |
| GO Biological Processes | cellular response to xenobiotic stimulus | -7.1 | 2.3 | 6.1 | 170 |
| GO Biological Processes | regulation of ion transmembrane transporter activity | -7.1 | 2.1 | 5.9 | 239 |
| GO Biological Processes | regulation of peptidase activity | -7 | 1.7 | 5.7 | 445 |
| GO Biological Processes | divalent inorganic cation transport | -7 | 1.7 | 5.7 | 471 |

| | | | | | |
|---|---|---|---|---|---|
| GO Biological Processes | reactive oxygen species metabolic process | -7 | 2 | 5.9 | 276 |
| GO Biological Processes | actomyosin structure organization | -7 | 2.2 | 6 | 194 |
| GO Biological Processes | positive regulation of MAPK cascade | -7 | 1.7 | 5.7 | 544 |
| GO Biological Processes | negative regulation of coagulation | -7 | 3.4 | 6.5 | 56 |
| GO Biological Processes | regulation of inflammatory response | -6.9 | 1.7 | 5.7 | 460 |
| GO Biological Processes | regulation of muscle contraction | -6.9 | 2.3 | 6 | 167 |
| GO Biological Processes | positive regulation of nervous system development | -6.9 | 1.7 | 5.6 | 526 |
| GO Biological Processes | chordate embryonic development | -6.9 | 1.6 | 5.6 | 600 |
| GO Biological Processes | myofibril assembly | -6.9 | 3.1 | 6.3 | 70 |
| GO Biological Processes | axoneme assembly | -6.9 | 3.2 | 6.4 | 61 |
| GO Biological Processes | ear development | -6.9 | 2.1 | 5.9 | 213 |
| GO Biological Processes | muscle organ morphogenesis | -6.9 | 2.8 | 6.2 | 89 |
| GO Biological Processes | monoamine transport | -6.9 | 2.8 | 6.2 | 89 |
| GO Biological Processes | response to molecule of bacterial origin | -6.9 | 1.8 | 5.7 | 340 |
| GO Biological Processes | divalent metal ion transport | -6.9 | 1.7 | 5.6 | 468 |
| GO Biological Processes | phospholipase C-activating G protein-coupled receptor signaling pathway | -6.9 | 2.7 | 6.1 | 99 |
| GO Biological Processes | cell-matrix adhesion | -6.9 | 2.1 | 5.8 | 225 |
| GO Biological Processes | muscle cell differentiation | -6.8 | 1.8 | 5.7 | 379 |
| GO Biological Processes | supramolecular fiber organization | -6.7 | 1.6 | 5.5 | 659 |
| GO Biological Processes | organic acid transport | -6.7 | 1.9 | 5.7 | 318 |
| GO Biological Processes | carboxylic acid transport | -6.7 | 1.9 | 5.7 | 318 |
| GO Biological Processes | negative regulation of endopeptidase activity | -6.7 | 2 | 5.7 | 251 |
| GO Biological Processes | positive regulation of cell development | -6.7 | 1.6 | 5.5 | 538 |
| GO Biological Processes | homophilic cell adhesion via plasma membrane adhesion molecules | -6.7 | 2.2 | 5.8 | 170 |
| GO Biological Processes | embryo development ending in birth or egg hatching | -6.7 | 1.6 | 5.5 | 620 |
| GO Biological Processes | positive regulation of cell migration | -6.7 | 1.7 | 5.5 | 526 |
| GO Biological Processes | response to acid chemical | -6.7 | 1.8 | 5.6 | 338 |
| GO Biological Processes | mesonephros development | -6.7 | 2.6 | 6 | 101 |
| GO Biological Processes | regulation of lipase activity | -6.6 | 2.7 | 6 | 96 |
| GO Biological Processes | anterior/posterior pattern specification | -6.6 | 2.1 | 5.7 | 211 |
| GO Biological Processes | regulation of neuron differentiation | -6.6 | 1.6 | 5.4 | 642 |
| GO Biological Processes | neurotransmitter transport | -6.6 | 2 | 5.6 | 265 |
| GO Biological Processes | response to hypoxia | -6.6 | 1.9 | 5.6 | 315 |
| GO Biological Processes | ameboidal-type cell migration | -6.5 | 1.7 | 5.5 | 463 |
| GO Biological Processes | kidney epithelium development | -6.5 | 2.4 | 5.8 | 139 |
| GO Biological Processes | regulation of blood pressure | -6.5 | 2.2 | 5.7 | 178 |
| GO Biological Processes | microtubule-based process | -6.5 | 1.5 | 5.4 | 748 |
| GO Biological Processes | positive regulation of epithelial cell proliferation | -6.5 | 2.1 | 5.7 | 201 |
| GO Biological Processes | signal release | -6.5 | 1.7 | 5.4 | 457 |
| GO Biological Processes | regulation of anatomical structure size | -6.5 | 1.7 | 5.4 | 497 |
| GO Biological Processes | skeletal system morphogenesis | -6.5 | 2 | 5.6 | 237 |
| GO Biological Processes | positive regulation of protein tyrosine kinase activity | -6.5 | 3.3 | 6.2 | 55 |
| GO Biological Processes | regulation of smooth muscle contraction | -6.5 | 3.1 | 6.1 | 64 |
| GO Biological Processes | bone remodeling | -6.4 | 2.8 | 6 | 83 |
| GO Biological Processes | regulation of chromosome segregation | -6.4 | 2.6 | 5.9 | 103 |

| | | | | | |
|---|---|---|---|---|---|
| GO Biological Processes | cellular response to inorganic substance | -6.4 | 2.1 | 5.6 | 208 |
| GO Biological Processes | leukocyte chemotaxis | -6.4 | 2 | 5.6 | 220 |
| GO Biological Processes | negative regulation of hydrolase activity | -6.4 | 1.7 | 5.4 | 459 |
| GO Biological Processes | digestion | -6.4 | 2.4 | 5.7 | 135 |
| GO Biological Processes | cardiac muscle tissue morphogenesis | -6.4 | 3 | 6 | 69 |
| GO Biological Processes | striated muscle cell differentiation | -6.4 | 1.9 | 5.5 | 287 |
| GO Biological Processes | sensory organ morphogenesis | -6.3 | 2 | 5.5 | 251 |
| GO Biological Processes | appendage morphogenesis | -6.3 | 2.3 | 5.7 | 147 |
| GO Biological Processes | limb morphogenesis | -6.3 | 2.3 | 5.7 | 147 |
| GO Biological Processes | response to decreased oxygen levels | -6.3 | 1.8 | 5.4 | 326 |
| GO Biological Processes | positive regulation of lipase activity | -6.3 | 3 | 5.9 | 70 |
| GO Biological Processes | regulation of phospholipase activity | -6.3 | 3 | 5.9 | 70 |
| GO Biological Processes | regulation of cell morphogenesis involved in differentiation | -6.2 | 1.9 | 5.4 | 296 |
| GO Biological Processes | developmental growth involved in morphogenesis | -6.2 | 2 | 5.5 | 229 |
| GO Biological Processes | regulation of systemic arterial blood pressure mediated by a chemical signal | -6.2 | 3.4 | 6.1 | 48 |
| GO Biological Processes | microtubule cytoskeleton organization involved in mitosis | -6.2 | 2.4 | 5.6 | 127 |
| GO Biological Processes | regulation of mitotic sister chromatid separation | -6.2 | 3.2 | 6 | 57 |
| GO Biological Processes | myeloid leukocyte activation | -6.1 | 1.6 | 5.2 | 649 |
| GO Biological Processes | cartilage development | -6.1 | 2 | 5.4 | 207 |
| GO Biological Processes | action potential | -6.1 | 2.3 | 5.6 | 133 |
| GO Biological Processes | positive regulation of transferase activity | -6.1 | 1.6 | 5.2 | 636 |
| GO Biological Processes | regulation of developmental growth | -6.1 | 1.8 | 5.3 | 343 |
| GO Biological Processes | cellular response to metal ion | -6.1 | 2.1 | 5.5 | 184 |
| GO Biological Processes | branching morphogenesis of an epithelial tube | -6.1 | 2.2 | 5.5 | 150 |
| GO Biological Processes | regeneration | -6.1 | 2.1 | 5.4 | 190 |
| GO Biological Processes | morphogenesis of a branching structure | -6.1 | 2.1 | 5.4 | 196 |
| GO Biological Processes | response to oxygen levels | -6.1 | 1.8 | 5.3 | 350 |
| GO Biological Processes | negative regulation of cell development | -6 | 1.8 | 5.3 | 331 |
| GO Biological Processes | small molecule biosynthetic process | -6 | 1.5 | 5.1 | 763 |
| GO Biological Processes | trabecula morphogenesis | -6 | 3.3 | 5.9 | 49 |
| GO Biological Processes | positive regulation of phospholipase activity | -6 | 3.1 | 5.9 | 58 |
| GO Biological Processes | negative regulation of cellular component organization | -6 | 1.5 | 5.1 | 715 |
| GO Biological Processes | fatty acid derivative biosynthetic process | -6 | 2.6 | 5.6 | 92 |
| GO Biological Processes | microtubule cytoskeleton organization | -6 | 1.6 | 5.1 | 550 |
| GO Biological Processes | glial cell differentiation | -6 | 2 | 5.4 | 203 |
| GO Biological Processes | adenylate cyclase-activating G protein-coupled receptor signaling pathway | -6 | 2.3 | 5.5 | 140 |
| GO Biological Processes | positive regulation of ERK1 and ERK2 cascade | -6 | 2 | 5.4 | 215 |
| GO Biological Processes | negative regulation of cellular component movement | -6 | 1.7 | 5.2 | 384 |
| GO Biological Processes | cell surface receptor signaling pathway involved in cell-cell signaling | -6 | 1.6 | 5.1 | 571 |
| GO Biological Processes | inner ear development | -5.9 | 2.1 | 5.4 | 186 |
| GO Biological Processes | metaphase/anaphase transition of mitotic cell cycle | -5.9 | 3.2 | 5.8 | 54 |
| GO Biological Processes | positive regulation of transmembrane transport | -5.9 | 2 | 5.3 | 198 |
| GO Biological Processes | positive regulation of vasculature development | -5.9 | 2 | 5.3 | 228 |

| | | | | | |
|---|---|---|---|---|---|
| GO Biological Processes | cardiac ventricle morphogenesis | -5.9 | 2.8 | 5.7 | 73 |
| GO Biological Processes | cognition | -5.9 | 1.8 | 5.2 | 290 |
| GO Biological Processes | endocrine process | -5.9 | 2.7 | 5.6 | 83 |
| GO Biological Processes | icosanoid biosynthetic process | -5.9 | 3.3 | 5.8 | 50 |
| GO Biological Processes | regulation of calcium ion transport | -5.9 | 1.9 | 5.3 | 241 |
| GO Biological Processes | embryonic appendage morphogenesis | -5.9 | 2.3 | 5.5 | 125 |
| GO Biological Processes | embryonic skeletal system development | -5.9 | 2.3 | 5.5 | 125 |
| GO Biological Processes | embryonic limb morphogenesis | -5.9 | 2.3 | 5.5 | 125 |
| GO Biological Processes | nephron development | -5.9 | 2.3 | 5.4 | 136 |
| GO Biological Processes | bone mineralization | -5.8 | 2.4 | 5.5 | 109 |
| GO Biological Processes | regulation of endopeptidase activity | -5.8 | 1.7 | 5.1 | 420 |
| GO Biological Processes | morphogenesis of a branching epithelium | -5.8 | 2.1 | 5.3 | 182 |
| GO Biological Processes | muscle cell development | -5.8 | 2.1 | 5.3 | 182 |
| GO Biological Processes | cardioblast differentiation | -5.8 | 5 | 6.3 | 19 |
| GO Biological Processes | response to cAMP | -5.8 | 2.5 | 5.5 | 99 |
| GO Biological Processes | neurotransmitter metabolic process | -5.8 | 2.2 | 5.4 | 148 |
| GO Biological Processes | connective tissue development | -5.8 | 1.9 | 5.2 | 267 |
| GO Biological Processes | granulocyte migration | -5.8 | 2.3 | 5.4 | 137 |
| GO Biological Processes | regulation of blood vessel size | -5.8 | 2.3 | 5.4 | 137 |
| GO Biological Processes | actin cytoskeleton organization | -5.7 | 1.5 | 5 | 646 |
| GO Biological Processes | granulocyte chemotaxis | -5.7 | 2.3 | 5.4 | 121 |
| GO Biological Processes | negative regulation of nuclear division | -5.7 | 3 | 5.7 | 60 |
| GO Biological Processes | mitotic sister chromatid separation | -5.7 | 3 | 5.7 | 60 |
| GO Biological Processes | endothelium development | -5.7 | 2.3 | 5.4 | 132 |
| GO Biological Processes | negative regulation of locomotion | -5.7 | 1.7 | 5.1 | 396 |
| GO Biological Processes | response to mechanical stimulus | -5.7 | 2 | 5.2 | 207 |
| GO Biological Processes | glial cell development | -5.7 | 2.5 | 5.4 | 105 |
| GO Biological Processes | organic hydroxy compound metabolic process | -5.7 | 1.6 | 5 | 523 |
| GO Biological Processes | sensory system development | -5.7 | 1.7 | 5.1 | 357 |
| GO Biological Processes | spindle checkpoint | -5.7 | 3.8 | 5.9 | 34 |
| GO Biological Processes | mitotic spindle checkpoint | -5.7 | 3.8 | 5.9 | 34 |
| GO Biological Processes | mitotic spindle assembly checkpoint | -5.7 | 3.8 | 5.9 | 34 |
| GO Biological Processes | heart trabecula morphogenesis | -5.7 | 3.8 | 5.9 | 34 |
| GO Biological Processes | spindle assembly checkpoint | -5.7 | 3.8 | 5.9 | 34 |
| GO Biological Processes | embryonic digestive tract development | -5.7 | 3.8 | 5.9 | 34 |
| GO Biological Processes | regulation of mitotic metaphase/anaphase transition | -5.7 | 3.2 | 5.7 | 51 |
| GO Biological Processes | regulation of tube size | -5.7 | 2.2 | 5.3 | 138 |
| GO Biological Processes | potassium ion transport | -5.7 | 1.9 | 5.2 | 238 |
| GO Biological Processes | cellular response to drug | -5.7 | 1.7 | 5.1 | 358 |
| GO Biological Processes | chromosome separation | -5.7 | 2.6 | 5.5 | 90 |
| GO Biological Processes | visual system development | -5.7 | 1.7 | 5.1 | 352 |
| GO Biological Processes | regulation of sister chromatid segregation | -5.7 | 2.7 | 5.5 | 80 |
| GO Biological Processes | outflow tract morphogenesis | -5.7 | 2.7 | 5.5 | 80 |
| GO Biological Processes | metaphase/anaphase transition of cell cycle | -5.6 | 3.1 | 5.6 | 56 |
| GO Biological Processes | temperature homeostasis | -5.6 | 2.1 | 5.2 | 173 |
| GO Biological Processes | monocarboxylic acid biosynthetic process | -5.6 | 1.8 | 5.1 | 295 |

| | | | | | |
|---|---|---|---|---|---|
| GO Biological Processes | developmental process involved in reproduction | -5.6 | 1.5 | 4.9 | 671 |
| GO Biological Processes | response to estradiol | -5.6 | 2.3 | 5.3 | 128 |
| GO Biological Processes | positive regulation of synaptic transmission | -5.6 | 2.1 | 5.2 | 162 |
| GO Biological Processes | neural precursor cell proliferation | -5.6 | 2.2 | 5.2 | 145 |
| GO Biological Processes | ureteric bud development | -5.6 | 2.5 | 5.4 | 96 |
| GO Biological Processes | heart contraction | -5.6 | 1.9 | 5.1 | 246 |
| GO Biological Processes | negative regulation of mitotic nuclear division | -5.6 | 3.1 | 5.6 | 52 |
| GO Biological Processes | regulation of systemic arterial blood pressure | -5.6 | 2.6 | 5.4 | 91 |
| GO Biological Processes | negative regulation of mitotic sister chromatid separation | -5.5 | 3.5 | 5.7 | 39 |
| GO Biological Processes | positive regulation of kinase activity | -5.5 | 1.6 | 4.9 | 562 |
| GO Biological Processes | positive regulation of protein kinase activity | -5.5 | 1.6 | 4.9 | 521 |
| GO Biological Processes | negative regulation of neurogenesis | -5.5 | 1.8 | 5 | 284 |
| GO Biological Processes | negative regulation of nervous system development | -5.5 | 1.8 | 5 | 303 |
| GO Biological Processes | regulation of muscle system process | -5.5 | 1.9 | 5.1 | 253 |
| GO Biological Processes | microtubule-based movement | -5.5 | 1.8 | 5 | 278 |
| GO Biological Processes | positive regulation of cell-substrate adhesion | -5.5 | 2.3 | 5.3 | 118 |
| GO Biological Processes | mesonephric epithelium development | -5.5 | 2.5 | 5.3 | 97 |
| GO Biological Processes | mesonephric tubule development | -5.5 | 2.5 | 5.3 | 97 |
| GO Biological Processes | animal organ formation | -5.5 | 2.9 | 5.5 | 62 |
| GO Biological Processes | regulation of chromosome separation | -5.5 | 2.9 | 5.5 | 62 |
| GO Biological Processes | drug metabolic process | -5.5 | 1.5 | 4.8 | 789 |
| GO Biological Processes | regulation of cell morphogenesis | -5.5 | 1.6 | 4.9 | 476 |
| GO Biological Processes | response to corticosteroid | -5.5 | 2.1 | 5.1 | 164 |
| GO Biological Processes | eye development | -5.5 | 1.7 | 4.9 | 350 |
| GO Biological Processes | regulation of blood vessel diameter | -5.4 | 2.3 | 5.2 | 130 |
| GO Biological Processes | regulation of tube diameter | -5.4 | 2.3 | 5.2 | 130 |
| GO Biological Processes | positive regulation of animal organ morphogenesis | -5.4 | 2.6 | 5.3 | 82 |
| GO Biological Processes | vasculogenesis | -5.4 | 2.7 | 5.4 | 77 |
| GO Biological Processes | regulation of bone remodeling | -5.4 | 3.3 | 5.6 | 44 |
| GO Biological Processes | negative regulation of sister chromatid segregation | -5.4 | 3.3 | 5.6 | 44 |
| GO Biological Processes | regulation of metaphase/anaphase transition of cell cycle | -5.4 | 3.1 | 5.5 | 53 |
| GO Biological Processes | heart process | -5.4 | 1.9 | 5 | 255 |
| GO Biological Processes | regulation of cell growth | -5.4 | 1.7 | 4.9 | 410 |
| GO Biological Processes | heat generation | -5.4 | 5.1 | 6.1 | 17 |
| GO Biological Processes | aging | -5.4 | 1.8 | 4.9 | 319 |
| GO Biological Processes | atrioventricular valve development | -5.4 | 4.3 | 5.9 | 24 |
| GO Biological Processes | negative regulation of chromosome separation | -5.4 | 3.4 | 5.6 | 40 |
| GO Biological Processes | regulation of synaptic plasticity | -5.4 | 2 | 5.1 | 177 |
| GO Biological Processes | cellular response to lipid | -5.4 | 1.5 | 4.8 | 616 |
| GO Biological Processes | sarcomere organization | -5.3 | 3.2 | 5.5 | 49 |
| GO Biological Processes | regulation of protein tyrosine kinase activity | -5.3 | 2.5 | 5.3 | 88 |
| GO Biological Processes | negative regulation of mitotic metaphase/anaphase transition | -5.3 | 3.6 | 5.6 | 36 |
| GO Biological Processes | cytokine production | -5.3 | 1.5 | 4.7 | 737 |
| GO Biological Processes | actin-mediated cell contraction | -5.3 | 2.3 | 5.2 | 115 |

| | | | | | |
|---|---|---|---|---|---|
| GO Biological Processes | skeletal muscle organ development | -5.3 | 2.1 | 5 | 172 |
| GO Biological Processes | cell maturation | -5.3 | 2.1 | 5 | 172 |
| GO Biological Processes | learning or memory | -5.3 | 1.9 | 4.9 | 251 |
| GO Biological Processes | ventricular cardiac muscle tissue development | -5.3 | 3 | 5.4 | 54 |
| GO Biological Processes | regulation of embryonic development | -5.3 | 2.2 | 5.1 | 132 |
| GO Biological Processes | negative regulation of chromosome segregation | -5.3 | 3.3 | 5.5 | 45 |
| GO Biological Processes | reproductive structure development | -5.3 | 1.6 | 4.8 | 427 |
| GO Biological Processes | regulation of cytokine production | -5.3 | 1.5 | 4.7 | 668 |
| GO Biological Processes | fatty acid derivative metabolic process | -5.3 | 2.1 | 5 | 161 |
| GO Biological Processes | anatomical structure maturation | -5.3 | 2.1 | 5 | 161 |
| GO Biological Processes | negative regulation of proteolysis | -5.3 | 1.7 | 4.8 | 361 |
| GO Biological Processes | tissue homeostasis | -5.2 | 1.9 | 4.9 | 221 |
| GO Biological Processes | cardiocyte differentiation | -5.2 | 2.1 | 5 | 167 |
| GO Biological Processes | roof of mouth development | -5.2 | 2.5 | 5.2 | 89 |
| GO Biological Processes | locomotory behavior | -5.2 | 2 | 4.9 | 197 |
| GO Biological Processes | neurotransmitter uptake | -5.2 | 3.4 | 5.5 | 41 |
| GO Biological Processes | negative regulation of developmental growth | -5.2 | 2.3 | 5.1 | 111 |
| GO Biological Processes | synapse organization | -5.2 | 1.7 | 4.8 | 389 |
| GO Biological Processes | kidney morphogenesis | -5.2 | 2.4 | 5.1 | 95 |
| GO Biological Processes | regulation of blood circulation | -5.2 | 1.8 | 4.8 | 266 |
| GO Biological Processes | negative regulation of metaphase/anaphase transition of cell cycle | -5.2 | 3.5 | 5.5 | 37 |
| GO Biological Processes | transmembrane receptor protein serine/threonine kinase signaling pathway | -5.2 | 1.7 | 4.8 | 350 |
| GO Biological Processes | reproductive system development | -5.1 | 1.6 | 4.7 | 430 |
| GO Biological Processes | mitotic spindle organization | -5.1 | 2.4 | 5.1 | 106 |
| GO Biological Processes | fatty acid biosynthetic process | -5.1 | 2.1 | 4.9 | 157 |
| GO Biological Processes | skeletal muscle tissue development | -5.1 | 2.1 | 4.9 | 163 |
| GO Biological Processes | regulation of chemotaxis | -5.1 | 1.9 | 4.9 | 211 |
| GO Biological Processes | integrin-mediated signaling pathway | -5.1 | 2.4 | 5.1 | 101 |
| GO Biological Processes | striated muscle cell development | -5.1 | 2 | 4.9 | 169 |
| GO Biological Processes | collagen metabolic process | -5.1 | 2.3 | 5 | 112 |
| GO Biological Processes | astrocyte differentiation | -5.1 | 2.6 | 5.2 | 75 |
| GO Biological Processes | regulation of cellular response to growth factor stimulus | -5.1 | 1.8 | 4.8 | 287 |
| GO Biological Processes | endothelial cell differentiation | -5.1 | 2.3 | 5 | 118 |
| GO Biological Processes | positive regulation of calcium ion transport | -5.1 | 2.3 | 5 | 118 |
| GO Biological Processes | cellular response to acid chemical | -5.1 | 1.9 | 4.8 | 206 |
| GO Biological Processes | negative regulation of mitotic sister chromatid segregation | -5.1 | 3.3 | 5.4 | 42 |
| GO Biological Processes | positive regulation of angiogenesis | -5 | 1.9 | 4.8 | 200 |
| GO Biological Processes | humoral immune response | -5 | 1.7 | 4.7 | 353 |
| GO Biological Processes | determination of bilateral symmetry | -5 | 2.2 | 4.9 | 124 |
| GO Biological Processes | amine transport | -5 | 2.4 | 5 | 102 |
| GO Biological Processes | sensory perception of pain | -5 | 2.4 | 5 | 102 |
| GO Biological Processes | cellular response to transforming growth factor beta stimulus | -5 | 1.8 | 4.8 | 250 |
| GO Biological Processes | positive regulation of cell cycle | -5 | 1.6 | 4.7 | 400 |
| GO Biological Processes | positive regulation of Wnt signaling pathway | -5 | 2.2 | 4.9 | 130 |

| | | | | | |
|---|---|---|---|---|---|
| GO Biological Processes | regulation of transmembrane receptor protein serine/threonine kinase signaling pathway | -5 | 1.8 | 4.8 | 238 |
| GO Biological Processes | regulation of animal organ formation | -5 | 3.4 | 5.4 | 38 |
| GO Biological Processes | response to transforming growth factor beta | -5 | 1.8 | 4.7 | 257 |
| GO Biological Processes | mitotic cell cycle checkpoint | -5 | 2 | 4.8 | 165 |
| GO Biological Processes | atrioventricular valve morphogenesis | -5 | 4.3 | 5.6 | 22 |
| GO Biological Processes | organ induction | -5 | 4.3 | 5.6 | 22 |
| GO Biological Processes | multicellular organismal signaling | -5 | 2 | 4.8 | 177 |
| GO Biological Processes | ventricular cardiac muscle tissue morphogenesis | -5 | 3.1 | 5.3 | 47 |
| GO Biological Processes | drug catabolic process | -5 | 2.1 | 4.9 | 142 |
| GO Biological Processes | plasma membrane organization | -5 | 2.4 | 5 | 92 |
| GO Biological Processes | specification of symmetry | -5 | 2.2 | 4.9 | 125 |
| GO Biological Processes | chemokine-mediated signaling pathway | -4.9 | 2.5 | 5 | 87 |
| GO Biological Processes | tissue remodeling | -4.9 | 2 | 4.8 | 172 |
| GO Biological Processes | negative regulation of cell growth | -4.9 | 2 | 4.8 | 184 |
| GO Biological Processes | actin filament-based movement | -4.9 | 2.1 | 4.8 | 137 |
| GO Biological Processes | negative regulation of immune system process | -4.9 | 1.6 | 4.6 | 450 |
| GO Biological Processes | hydrogen peroxide metabolic process | -4.9 | 2.9 | 5.1 | 57 |
| GO Biological Processes | animal organ regeneration | -4.9 | 2.6 | 5 | 77 |
| GO Biological Processes | catecholamine transport | -4.9 | 2.6 | 5 | 77 |
| GO Biological Processes | biomineral tissue development | -4.9 | 2.1 | 4.8 | 155 |
| GO Biological Processes | negative regulation of secretion | -4.9 | 1.9 | 4.7 | 228 |
| GO Biological Processes | cyclic-nucleotide-mediated signaling | -4.8 | 1.9 | 4.7 | 216 |
| GO Biological Processes | lung alveolus development | -4.8 | 3.3 | 5.2 | 39 |
| GO Biological Processes | regulation of systemic arterial blood pressure by hormone | -4.8 | 3.3 | 5.2 | 39 |
| GO Biological Processes | transmembrane receptor protein tyrosine kinase signaling pathway | -4.8 | 1.5 | 4.5 | 711 |
| GO Biological Processes | chondrocyte differentiation | -4.8 | 2.2 | 4.8 | 121 |
| GO Biological Processes | response to antibiotic | -4.8 | 1.7 | 4.6 | 325 |
| GO Biological Processes | icosanoid metabolic process | -4.8 | 2.3 | 4.8 | 110 |
| GO Biological Processes | positive regulation of ion transmembrane transport | -4.8 | 2.1 | 4.8 | 150 |
| GO Biological Processes | monovalent inorganic cation transport | -4.8 | 1.5 | 4.5 | 529 |
| GO Biological Processes | reactive oxygen species biosynthetic process | -4.8 | 2.2 | 4.8 | 116 |
| GO Biological Processes | fatty acid transport | -4.8 | 2.4 | 4.9 | 94 |
| GO Biological Processes | regulation of neuron projection development | -4.8 | 1.6 | 4.5 | 481 |
| GO Biological Processes | hormone secretion | -4.8 | 1.7 | 4.6 | 313 |
| GO Biological Processes | vasoconstriction | -4.8 | 2.6 | 4.9 | 73 |
| GO Biological Processes | regulation of synapse assembly | -4.8 | 2.3 | 4.8 | 105 |
| GO Biological Processes | regulation of mitotic sister chromatid segregation | -4.8 | 2.7 | 5 | 68 |
| GO Biological Processes | positive regulation of neurogenesis | -4.7 | 1.6 | 4.5 | 461 |
| GO Biological Processes | regulation of peptidyl-tyrosine phosphorylation | -4.7 | 1.8 | 4.6 | 249 |
| GO Biological Processes | neurotransmitter reuptake | -4.7 | 4.1 | 5.4 | 23 |
| GO Biological Processes | axonemal dynein complex assembly | -4.7 | 3.6 | 5.3 | 31 |
| GO Biological Processes | endocrine system development | -4.7 | 2.2 | 4.7 | 128 |
| GO Biological Processes | platelet degranulation | -4.7 | 2.2 | 4.7 | 128 |
| GO Biological Processes | synaptic membrane adhesion | -4.7 | 3.8 | 5.3 | 27 |

| | | | | | |
|---|---|---|---|---|---|
| GO Biological Processes | cellular potassium ion transport | -4.7 | 1.9 | 4.6 | 212 |
| GO Biological Processes | potassium ion transmembrane transport | -4.7 | 1.9 | 4.6 | 212 |
| GO Biological Processes | cellular response to chemokine | -4.7 | 2.4 | 4.8 | 95 |
| GO Biological Processes | response to chemokine | -4.7 | 2.4 | 4.8 | 95 |
| GO Biological Processes | regulation of Wnt signaling pathway | -4.7 | 1.7 | 4.5 | 315 |
| GO Biological Processes | camera-type eye development | -4.7 | 1.7 | 4.5 | 302 |
| GO Biological Processes | cell junction organization | -4.7 | 1.7 | 4.5 | 289 |
| GO Biological Processes | positive regulation of peptidyl-tyrosine phosphorylation | -4.7 | 1.9 | 4.6 | 188 |
| GO Biological Processes | catecholamine metabolic process | -4.7 | 2.9 | 5 | 54 |
| GO Biological Processes | positive regulation of calcium ion transport into cytosol | -4.7 | 2.9 | 5 | 54 |
| GO Biological Processes | catechol-containing compound metabolic process | -4.7 | 2.9 | 5 | 54 |
| GO Biological Processes | calcium ion transmembrane transport | -4.7 | 1.7 | 4.5 | 309 |
| GO Biological Processes | negative regulation of secretion by cell | -4.6 | 1.9 | 4.6 | 201 |
| GO Biological Processes | protein complex oligomerization | -4.6 | 1.5 | 4.4 | 555 |
| GO Biological Processes | stem cell development | -4.6 | 2.4 | 4.8 | 85 |
| GO Biological Processes | DNA-dependent DNA replication | -4.6 | 2.1 | 4.6 | 147 |
| GO Biological Processes | collagen-activated signaling pathway | -4.6 | 5.3 | 5.6 | 13 |
| GO Biological Processes | cilium-dependent cell motility | -4.6 | 3.4 | 5.1 | 36 |
| GO Biological Processes | cilium or flagellum-dependent cell motility | -4.6 | 3.4 | 5.1 | 36 |
| GO Biological Processes | negative regulation of cell morphogenesis involved in differentiation | -4.6 | 2.3 | 4.7 | 96 |
| GO Biological Processes | neutrophil mediated immunity | -4.6 | 1.5 | 4.4 | 500 |
| GO Biological Processes | negative regulation of transport | -4.6 | 1.5 | 4.4 | 507 |
| GO Biological Processes | tissue migration | -4.6 | 1.6 | 4.4 | 357 |
| GO Biological Processes | acid secretion | -4.6 | 2.2 | 4.7 | 113 |
| GO Biological Processes | DNA conformation change | -4.6 | 1.7 | 4.4 | 285 |
| GO Biological Processes | attachment of spindle microtubules to kinetochore | -4.6 | 3.5 | 5.1 | 32 |
| GO Biological Processes | cellular response to vitamin | -4.6 | 3.5 | 5.1 | 32 |
| GO Biological Processes | negative regulation of interferon-gamma production | -4.6 | 3.5 | 5.1 | 32 |
| GO Biological Processes | neurotransmitter biosynthetic process | -4.6 | 2.3 | 4.7 | 102 |
| GO Biological Processes | receptor-mediated endocytosis | -4.5 | 1.6 | 4.4 | 358 |
| GO Biological Processes | sex differentiation | -4.5 | 1.7 | 4.5 | 266 |
| GO Biological Processes | platelet activation | -4.5 | 2 | 4.6 | 154 |
| GO Biological Processes | synapse assembly | -4.5 | 2 | 4.6 | 166 |
| GO Biological Processes | regulation of stem cell proliferation | -4.5 | 2.7 | 4.8 | 65 |
| GO Biological Processes | negative regulation of axon guidance | -4.5 | 3.7 | 5.2 | 28 |
| GO Biological Processes | fluid transport | -4.5 | 3.7 | 5.2 | 28 |
| GO Biological Processes | fibrinolysis | -4.5 | 3.7 | 5.2 | 28 |
| GO Biological Processes | negative chemotaxis | -4.5 | 3.2 | 5 | 41 |
| GO Biological Processes | macrophage activation | -4.5 | 2.4 | 4.7 | 86 |
| GO Biological Processes | regulation of synapse organization | -4.5 | 1.8 | 4.5 | 216 |
| GO Biological Processes | neutrophil activation involved in immune response | -4.5 | 1.5 | 4.3 | 489 |
| GO Biological Processes | morphogenesis of embryonic epithelium | -4.5 | 2 | 4.5 | 149 |
| GO Biological Processes | regulation of muscle tissue development | -4.5 | 2 | 4.5 | 155 |
| GO Biological Processes | somite development | -4.5 | 2.3 | 4.7 | 92 |

| | | | | | |
|---|---|---|---|---|---|
| GO Biological Processes | organ growth | -4.5 | 1.9 | 4.5 | 198 |
| GO Biological Processes | negative regulation of cell adhesion | -4.5 | 1.7 | 4.4 | 281 |
| GO Biological Processes | regulation of morphogenesis of a branching structure | -4.4 | 2.8 | 4.8 | 56 |
| GO Biological Processes | regulation of vasoconstriction | -4.4 | 2.8 | 4.8 | 56 |
| GO Biological Processes | regulation of neurotransmitter transport | -4.4 | 2 | 4.5 | 144 |
| GO Biological Processes | positive regulation of defense response | -4.4 | 1.6 | 4.3 | 450 |
| GO Biological Processes | regulation of canonical Wnt signaling pathway | -4.4 | 1.8 | 4.4 | 237 |
| GO Biological Processes | embryonic forelimb morphogenesis | -4.4 | 3.4 | 5 | 33 |
| GO Biological Processes | specification of animal organ identity | -4.4 | 3.4 | 5 | 33 |
| GO Biological Processes | response to iron ion | -4.4 | 3.4 | 5 | 33 |
| GO Biological Processes | developmental induction | -4.4 | 3.4 | 5 | 33 |
| GO Biological Processes | regulation of peptide transport | -4.4 | 1.4 | 4.2 | 698 |
| GO Biological Processes | cAMP-mediated signaling | -4.4 | 1.9 | 4.4 | 187 |
| GO Biological Processes | negative regulation of cell motility | -4.4 | 1.6 | 4.3 | 349 |
| GO Biological Processes | regulation of cation channel activity | -4.4 | 2 | 4.4 | 163 |
| GO Biological Processes | ventricular septum development | -4.3 | 2.5 | 4.7 | 72 |
| GO Biological Processes | hormone transport | -4.3 | 1.7 | 4.3 | 323 |
| GO Biological Processes | ear morphogenesis | -4.3 | 2.2 | 4.5 | 116 |
| GO Biological Processes | xenobiotic metabolic process | -4.3 | 2.2 | 4.5 | 116 |
| GO Biological Processes | calcium-mediated signaling | -4.3 | 1.8 | 4.4 | 219 |
| GO Biological Processes | icosanoid transport | -4.3 | 2.9 | 4.8 | 47 |
| GO Biological Processes | fatty acid derivative transport | -4.3 | 2.9 | 4.8 | 47 |
| GO Biological Processes | positive regulation of synapse assembly | -4.3 | 2.6 | 4.7 | 67 |
| GO Biological Processes | regulation of actin filament-based process | -4.3 | 1.6 | 4.2 | 384 |
| GO Biological Processes | response to steroid hormone | -4.3 | 1.6 | 4.2 | 391 |
| GO Biological Processes | regulation of axonogenesis | -4.3 | 1.9 | 4.4 | 182 |
| GO Biological Processes | heart looping | -4.3 | 2.7 | 4.7 | 57 |
| GO Biological Processes | myeloid cell activation involved in immune response | -4.3 | 1.5 | 4.2 | 544 |
| GO Biological Processes | artery development | -4.3 | 2.3 | 4.5 | 94 |
| GO Biological Processes | astrocyte development | -4.3 | 3.2 | 4.9 | 38 |
| GO Biological Processes | striated muscle contraction | -4.3 | 1.9 | 4.4 | 170 |
| GO Biological Processes | sodium ion transmembrane transport | -4.3 | 2 | 4.4 | 140 |
| GO Biological Processes | regulation of postsynaptic membrane potential | -4.3 | 2 | 4.4 | 140 |
| GO Biological Processes | regulation of heart contraction | -4.3 | 1.8 | 4.3 | 220 |
| GO Biological Processes | regulation of striated muscle tissue development | -4.3 | 2 | 4.4 | 152 |
| GO Biological Processes | ammonium transport | -4.3 | 2.2 | 4.5 | 111 |
| GO Biological Processes | homotypic cell-cell adhesion | -4.3 | 2.4 | 4.6 | 78 |
| GO Biological Processes | response to ketone | -4.3 | 1.9 | 4.3 | 189 |
| GO Biological Processes | regulation of calcium ion transport into cytosol | -4.3 | 2.2 | 4.5 | 100 |
| GO Biological Processes | meiotic chromosome segregation | -4.3 | 2.3 | 4.5 | 89 |
| GO Biological Processes | positive regulation of muscle tissue development | -4.3 | 2.3 | 4.5 | 89 |
| GO Biological Processes | metanephros development | -4.3 | 2.3 | 4.5 | 89 |
| GO Biological Processes | muscle cell proliferation | -4.3 | 1.8 | 4.3 | 240 |
| GO Biological Processes | regulation of neurological system process | -4.2 | 2.1 | 4.4 | 129 |
| GO Biological Processes | mitotic cell cycle phase transition | -4.2 | 1.5 | 4.1 | 532 |

| | | | | | |
|---|---|---|---|---|---|
| GO Biological Processes | positive regulation of cell adhesion | -4.2 | 1.6 | 4.2 | 400 |
| GO Biological Processes | regulation of small molecule metabolic process | -4.2 | 1.6 | 4.2 | 366 |
| GO Biological Processes | regulation of morphogenesis of an epithelium | -4.2 | 2 | 4.4 | 135 |
| GO Biological Processes | neuron migration | -4.2 | 2 | 4.4 | 153 |
| GO Biological Processes | positive chemotaxis | -4.2 | 2.5 | 4.6 | 68 |
| GO Biological Processes | cell cycle phase transition | -4.2 | 1.5 | 4.1 | 575 |
| GO Biological Processes | regulation of amine transport | -4.2 | 2.3 | 4.5 | 95 |
| GO Biological Processes | cardiac muscle cell myoblast differentiation | -4.2 | 5.5 | 5.4 | 11 |
| GO Biological Processes | surfactant homeostasis | -4.2 | 5.5 | 5.4 | 11 |
| GO Biological Processes | cellular response to organic cyclic compound | -4.2 | 1.5 | 4.1 | 547 |
| GO Biological Processes | determination of heart left/right asymmetry | -4.2 | 2.6 | 4.6 | 63 |
| GO Biological Processes | negative regulation of chemotaxis | -4.2 | 2.6 | 4.6 | 63 |
| GO Biological Processes | embryonic heart tube morphogenesis | -4.2 | 2.6 | 4.6 | 63 |
| GO Biological Processes | antibiotic catabolic process | -4.2 | 2.7 | 4.6 | 58 |
| GO Biological Processes | neural crest cell migration | -4.2 | 2.7 | 4.6 | 58 |
| GO Biological Processes | multi-multicellular organism process | -4.2 | 1.8 | 4.3 | 222 |
| GO Biological Processes | membrane depolarization | -4.2 | 2.3 | 4.5 | 90 |
| GO Biological Processes | sodium ion transport | -4.2 | 1.8 | 4.3 | 216 |
| GO Biological Processes | cardiac atrium morphogenesis | -4.2 | 3.4 | 4.9 | 30 |
| GO Biological Processes | antibiotic metabolic process | -4.2 | 2 | 4.3 | 148 |
| GO Biological Processes | embryonic digestive tract morphogenesis | -4.2 | 4.3 | 5.1 | 18 |
| GO Biological Processes | nephron tubule morphogenesis | -4.2 | 2.4 | 4.5 | 74 |
| GO Biological Processes | neutrophil activation | -4.1 | 1.5 | 4.1 | 500 |
| GO Biological Processes | cellular response to nitrogen compound | -4.1 | 1.4 | 4.1 | 628 |
| GO Biological Processes | mesenchymal cell development | -4.1 | 2.3 | 4.5 | 85 |
| GO Biological Processes | positive regulation of cell cycle process | -4.1 | 1.7 | 4.2 | 295 |
| GO Biological Processes | regulation of cell-matrix adhesion | -4.1 | 2.1 | 4.4 | 119 |
| GO Biological Processes | negative regulation of axonogenesis | -4.1 | 2.5 | 4.5 | 69 |
| GO Biological Processes | positive regulation of hydrolase activity | -4.1 | 1.4 | 4 | 766 |
| GO Biological Processes | integrin activation | -4.1 | 3.9 | 5 | 22 |
| GO Biological Processes | protein localization to chromosome, centromeric region | -4.1 | 3.9 | 5 | 22 |
| GO Biological Processes | acute inflammatory response | -4.1 | 1.8 | 4.2 | 217 |
| GO Biological Processes | positive regulation of cytokine production | -4.1 | 1.5 | 4.1 | 431 |
| GO Biological Processes | response to calcium ion | -4.1 | 2 | 4.3 | 149 |
| GO Biological Processes | positive regulation of inflammatory response | -4.1 | 2 | 4.3 | 143 |
| GO Biological Processes | digestive tract morphogenesis | -4.1 | 2.8 | 4.6 | 49 |
| GO Biological Processes | nephron epithelium development | -4.1 | 2.2 | 4.4 | 108 |
| GO Biological Processes | digestive system process | -4.1 | 2.2 | 4.4 | 97 |
| GO Biological Processes | anatomical structure homeostasis | -4.1 | 1.5 | 4.1 | 426 |
| GO Biological Processes | regulation of muscle organ development | -4 | 1.9 | 4.2 | 156 |
| GO Biological Processes | regulation of synapse structure or activity | -4 | 1.8 | 4.2 | 225 |
| GO Biological Processes | regulation of reproductive process | -4 | 2 | 4.2 | 144 |
| GO Biological Processes | positive regulation of cation transmembrane transport | -4 | 2 | 4.3 | 138 |
| GO Biological Processes | epithelium migration | -4 | 1.6 | 4.1 | 351 |
| GO Biological Processes | myeloid leukocyte mediated immunity | -4 | 1.5 | 4 | 553 |
| GO Biological Processes | cellular response to organonitrogen compound | -4 | 1.5 | 4 | 568 |

| | | | | | |
|---|---|---|---|---|---|
| GO Biological Processes | cellular response to calcium ion | -4 | 2.3 | 4.4 | 81 |
| GO Biological Processes | negative regulation of blood vessel diameter | -4 | 2.3 | 4.4 | 81 |
| GO Biological Processes | neural crest cell development | -4 | 2.3 | 4.4 | 81 |
| GO Biological Processes | ureteric bud morphogenesis | -4 | 2.5 | 4.4 | 65 |
| GO Biological Processes | determination of left/right symmetry | -4 | 2.1 | 4.3 | 115 |
| GO Biological Processes | mesenchymal cell proliferation | -4 | 2.9 | 4.6 | 45 |
| GO Biological Processes | icosanoid secretion | -4 | 2.9 | 4.6 | 45 |
| GO Biological Processes | endocardial cushion development | -4 | 2.9 | 4.6 | 45 |
| GO Biological Processes | sensory perception of sound | -4 | 2 | 4.2 | 145 |
| GO Biological Processes | hepaticobiliary system development | -4 | 2 | 4.2 | 139 |
| GO Biological Processes | heart valve morphogenesis | -4 | 2.8 | 4.5 | 50 |
| GO Biological Processes | cardiac septum morphogenesis | -4 | 2.4 | 4.4 | 76 |
| GO Biological Processes | nephron epithelium morphogenesis | -4 | 2.4 | 4.4 | 76 |
| GO Biological Processes | positive regulation of protein transport | -4 | 1.6 | 4 | 394 |
| GO Biological Processes | regulation of hormone secretion | -4 | 1.7 | 4.1 | 266 |
| GO Biological Processes | granulocyte activation | -4 | 1.5 | 4 | 506 |
| GO Biological Processes | regulation of small GTPase mediated signal transduction | -3.9 | 1.6 | 4 | 333 |
| GO Biological Processes | response to heparin | -3.9 | 7.2 | 5.5 | 6 |
| GO Biological Processes | serotonin uptake | -3.9 | 7.2 | 5.5 | 6 |
| GO Biological Processes | receptor internalization | -3.9 | 2.1 | 4.2 | 110 |
| GO Biological Processes | sensory perception of mechanical stimulus | -3.9 | 1.9 | 4.1 | 164 |
| GO Biological Processes | response to vitamin | -3.9 | 2.2 | 4.3 | 93 |
| GO Biological Processes | cell migration involved in heart development | -3.9 | 4.1 | 4.9 | 19 |
| GO Biological Processes | gas transport | -3.9 | 4.1 | 4.9 | 19 |
| GO Biological Processes | hyaluronan metabolic process | -3.9 | 3.1 | 4.6 | 36 |
| GO Biological Processes | neutrophil degranulation | -3.9 | 1.5 | 3.9 | 486 |
| GO Biological Processes | eye morphogenesis | -3.9 | 1.9 | 4.2 | 146 |
| GO Biological Processes | DNA replication | -3.9 | 1.7 | 4 | 267 |
| GO Biological Processes | negative regulation of cell migration | -3.9 | 1.6 | 4 | 334 |
| GO Biological Processes | protein homooligomerization | -3.9 | 1.6 | 4 | 334 |
| GO Biological Processes | mesonephric tubule morphogenesis | -3.9 | 2.5 | 4.4 | 66 |
| GO Biological Processes | aminoglycan catabolic process | -3.9 | 2.5 | 4.4 | 66 |
| GO Biological Processes | bone development | -3.9 | 1.8 | 4.1 | 209 |
| GO Biological Processes | forelimb morphogenesis | -3.9 | 2.9 | 4.5 | 41 |
| GO Biological Processes | regulation of heart morphogenesis | -3.9 | 2.9 | 4.5 | 41 |
| GO Biological Processes | positive regulation of muscle organ development | -3.9 | 2.3 | 4.3 | 88 |
| GO Biological Processes | positive regulation of striated muscle tissue development | -3.9 | 2.3 | 4.3 | 88 |
| GO Biological Processes | heart valve development | -3.9 | 2.6 | 4.4 | 56 |
| GO Biological Processes | heterophilic cell-cell adhesion via plasma membrane cell adhesion molecules | -3.9 | 2.8 | 4.5 | 46 |
| GO Biological Processes | regulation of gliogenesis | -3.9 | 2.1 | 4.2 | 117 |
| GO Biological Processes | regulation of cellular ketone metabolic process | -3.9 | 2 | 4.1 | 129 |
| GO Biological Processes | embryonic heart tube development | -3.8 | 2.4 | 4.3 | 72 |
| GO Biological Processes | transmission of nerve impulse | -3.8 | 2.4 | 4.3 | 72 |
| GO Biological Processes | regulation of protein transport | -3.8 | 1.4 | 3.9 | 668 |

| | | | | | |
|---|---|---|---|---|---|
| GO Biological Processes | ammonium ion metabolic process | -3.8 | 1.8 | 4 | 204 |
| GO Biological Processes | negative regulation of cell cycle phase transition | -3.8 | 1.7 | 4 | 217 |
| GO Biological Processes | negative regulation of cell-substrate adhesion | -3.8 | 2.4 | 4.3 | 67 |
| GO Biological Processes | adenylate cyclase-inhibiting G protein-coupled receptor signaling pathway | -3.8 | 2.2 | 4.2 | 89 |
| GO Biological Processes | response to glucocorticoid | -3.8 | 1.9 | 4.1 | 148 |
| GO Biological Processes | renal tubule morphogenesis | -3.8 | 2.3 | 4.2 | 78 |
| GO Biological Processes | primary alcohol metabolic process | -3.8 | 2.3 | 4.2 | 78 |
| GO Biological Processes | nephron morphogenesis | -3.8 | 2.3 | 4.2 | 78 |
| GO Biological Processes | axon extension involved in axon guidance | -3.8 | 3 | 4.5 | 37 |
| GO Biological Processes | neuron projection extension involved in neuron projection guidance | -3.8 | 3 | 4.5 | 37 |
| GO Biological Processes | liver development | -3.8 | 2 | 4.1 | 136 |
| GO Biological Processes | positive regulation of chemotaxis | -3.8 | 2 | 4.1 | 130 |
| GO Biological Processes | blood coagulation, fibrin clot formation | -3.8 | 3.4 | 4.6 | 28 |
| GO Biological Processes | embryonic hindlimb morphogenesis | -3.8 | 3.4 | 4.6 | 28 |
| GO Biological Processes | regulation of catecholamine secretion | -3.8 | 2.5 | 4.3 | 62 |
| GO Biological Processes | regulation of cell division | -3.8 | 1.9 | 4 | 167 |
| GO Biological Processes | aminoglycan metabolic process | -3.8 | 1.9 | 4 | 167 |
| GO Biological Processes | skeletal muscle contraction | -3.8 | 2.9 | 4.4 | 42 |
| GO Biological Processes | regulation of cyclin-dependent protein kinase activity | -3.8 | 2.1 | 4.1 | 101 |
| GO Biological Processes | leukocyte degranulation | -3.8 | 1.5 | 3.8 | 534 |
| GO Biological Processes | artery morphogenesis | -3.8 | 2.4 | 4.2 | 73 |
| GO Biological Processes | DNA replication checkpoint | -3.8 | 4.3 | 4.8 | 16 |
| GO Biological Processes | morphogenesis of an epithelial bud | -3.8 | 4.3 | 4.8 | 16 |
| GO Biological Processes | calcium ion transport into cytosol | -3.7 | 1.9 | 4 | 155 |
| GO Biological Processes | mesenchyme morphogenesis | -3.7 | 2.7 | 4.3 | 52 |
| GO Biological Processes | dopamine transport | -3.7 | 2.7 | 4.3 | 52 |
| GO Biological Processes | collagen catabolic process | -3.7 | 2.7 | 4.4 | 47 |
| GO Biological Processes | positive regulation of cell cycle phase transition | -3.7 | 2.1 | 4.1 | 107 |
| GO Biological Processes | vein smooth muscle contraction | -3.7 | 8.6 | 5.5 | 4 |
| GO Biological Processes | neural crest cell differentiation | -3.7 | 2.2 | 4.1 | 90 |
| GO Biological Processes | oligodendrocyte differentiation | -3.7 | 2.2 | 4.1 | 90 |
| GO Biological Processes | glutamate receptor signaling pathway | -3.7 | 2.2 | 4.1 | 90 |
| GO Biological Processes | regulation of actin cytoskeleton organization | -3.7 | 1.6 | 3.9 | 339 |
| GO Biological Processes | meiotic cell cycle process | -3.7 | 1.8 | 4 | 187 |
| GO Biological Processes | regulation of renal system process | -3.7 | 3.1 | 4.4 | 33 |
| GO Biological Processes | cell junction assembly | -3.7 | 1.7 | 3.9 | 239 |
| GO Biological Processes | regulation of cell cycle process | -3.7 | 1.4 | 3.7 | 747 |
| GO Biological Processes | positive regulation of cardiac muscle tissue development | -3.7 | 2.5 | 4.2 | 63 |
| GO Biological Processes | response to alcohol | -3.7 | 1.7 | 3.9 | 233 |
| GO Biological Processes | cardiac septum development | -3.7 | 2.1 | 4.1 | 108 |
| GO Biological Processes | terpenoid metabolic process | -3.7 | 2.1 | 4.1 | 108 |
| GO Biological Processes | regulation of cytoskeleton organization | -3.7 | 1.4 | 3.8 | 523 |
| GO Biological Processes | bone resorption | -3.7 | 2.5 | 4.2 | 58 |
| GO Biological Processes | positive regulation of stem cell proliferation | -3.7 | 2.9 | 4.4 | 38 |

| | | | | | |
|---|---|---|---|---|---|
| GO Biological Processes | regulation of bone resorption | -3.7 | 2.9 | 4.4 | 38 |
| GO Biological Processes | positive regulation of signaling receptor activity | -3.7 | 2.9 | 4.4 | 38 |
| GO Biological Processes | cellular response to hypoxia | -3.7 | 1.8 | 3.9 | 163 |
| GO Biological Processes | regulation of axon guidance | -3.6 | 2.8 | 4.3 | 43 |
| GO Biological Processes | unsaturated fatty acid biosynthetic process | -3.6 | 2.7 | 4.3 | 48 |
| GO Biological Processes | negative regulation of organelle organization | -3.6 | 1.5 | 3.8 | 383 |
| GO Biological Processes | reactive nitrogen species metabolic process | -3.6 | 2.3 | 4.1 | 80 |
| GO Biological Processes | long-chain fatty acid biosynthetic process | -3.6 | 3.3 | 4.4 | 29 |
| GO Biological Processes | regulation of presynapse assembly | -3.6 | 3.3 | 4.4 | 29 |
| GO Biological Processes | regulation of cell projection organization | -3.6 | 1.4 | 3.7 | 684 |
| GO Biological Processes | camera-type eye morphogenesis | -3.6 | 2.1 | 4 | 109 |
| GO Biological Processes | cellular hormone metabolic process | -3.6 | 2 | 4 | 121 |
| GO Biological Processes | regulation of sodium ion transport | -3.6 | 2.2 | 4.1 | 86 |
| GO Biological Processes | meiotic cell cycle | -3.6 | 1.7 | 3.8 | 248 |
| GO Biological Processes | regulation of hemopoiesis | -3.6 | 1.5 | 3.7 | 426 |
| GO Biological Processes | activation of transmembrane receptor protein tyrosine kinase activity | -3.6 | 4.6 | 4.8 | 13 |
| GO Biological Processes | regulation of integrin activation | -3.6 | 4.6 | 4.8 | 13 |
| GO Biological Processes | complement activation, lectin pathway | -3.6 | 4.6 | 4.8 | 13 |
| GO Biological Processes | regulation of heat generation | -3.6 | 4.6 | 4.8 | 13 |
| GO Biological Processes | chemical homeostasis within a tissue | -3.6 | 4.6 | 4.8 | 13 |
| GO Biological Processes | one-carbon compound transport | -3.6 | 4.6 | 4.8 | 13 |
| GO Biological Processes | catecholamine secretion | -3.6 | 2.4 | 4.1 | 64 |
| GO Biological Processes | positive regulation of mitotic cell cycle phase transition | -3.6 | 2.2 | 4 | 92 |
| GO Biological Processes | amine metabolic process | -3.6 | 2.2 | 4 | 92 |
| GO Biological Processes | cellular response to oxygen levels | -3.6 | 1.8 | 3.9 | 190 |
| GO Biological Processes | regulation of systemic arterial blood pressure by renin-angiotensin | -3.6 | 3.4 | 4.4 | 25 |
| GO Biological Processes | extracellular matrix assembly | -3.6 | 3.4 | 4.4 | 25 |
| GO Biological Processes | neuronal action potential | -3.6 | 3 | 4.3 | 34 |
| GO Biological Processes | cardiac atrium development | -3.6 | 3 | 4.3 | 34 |
| GO Biological Processes | leukocyte activation involved in immune response | -3.6 | 1.4 | 3.7 | 701 |
| GO Biological Processes | actin filament organization | -3.6 | 1.5 | 3.7 | 399 |
| GO Biological Processes | platelet aggregation | -3.6 | 2.5 | 4.1 | 59 |
| GO Biological Processes | meiotic nuclear division | -3.6 | 1.8 | 3.9 | 171 |
| GO Biological Processes | positive regulation of small molecule metabolic process | -3.6 | 1.9 | 3.9 | 146 |
| GO Biological Processes | negative regulation of cytokine production | -3.6 | 1.6 | 3.8 | 269 |
| GO Biological Processes | skeletal muscle cell differentiation | -3.5 | 2.3 | 4.1 | 70 |
| GO Biological Processes | cellular response to nutrient | -3.5 | 2.3 | 4.1 | 70 |
| GO Biological Processes | heart growth | -3.5 | 2 | 3.9 | 110 |
| GO Biological Processes | negative regulation of mitotic cell cycle phase transition | -3.5 | 1.7 | 3.8 | 197 |
| GO Biological Processes | ensheathment of neurons | -3.5 | 2 | 3.9 | 122 |
| GO Biological Processes | axon ensheathment | -3.5 | 2 | 3.9 | 122 |
| GO Biological Processes | positive regulation of neurotransmitter transport | -3.5 | 2.9 | 4.2 | 39 |
| GO Biological Processes | positive regulation of heart contraction | -3.5 | 2.9 | 4.2 | 39 |
| GO Biological Processes | ethanol metabolic process | -3.5 | 3.7 | 4.5 | 21 |
| GO Biological Processes | catecholamine biosynthetic process | -3.5 | 3.7 | 4.5 | 21 |

| | | | | | |
|---|---|---|---|---|---|
| GO Biological Processes | water transport | -3.5 | 3.7 | 4.5 | 21 |
| GO Biological Processes | catechol-containing compound biosynthetic process | -3.5 | 3.7 | 4.5 | 21 |
| GO Biological Processes | protein localization to kinetochore | -3.5 | 4.1 | 4.6 | 17 |
| GO Biological Processes | phenol-containing compound biosynthetic process | -3.5 | 2.7 | 4.2 | 44 |
| GO Biological Processes | regulation of endothelial cell differentiation | -3.5 | 2.7 | 4.2 | 44 |
| GO Biological Processes | nephron tubule development | -3.5 | 2.1 | 4 | 93 |
| GO Biological Processes | interleukin-1 production | -3.5 | 2.1 | 4 | 93 |
| GO Biological Processes | negative regulation of cell cycle process | -3.5 | 1.6 | 3.7 | 311 |
| GO Biological Processes | regulation of presynapse organization | -3.5 | 3.2 | 4.3 | 30 |
| GO Biological Processes | activation of phospholipase C activity | -3.5 | 3.2 | 4.3 | 30 |
| GO Biological Processes | extracellular matrix constituent secretion | -3.5 | 5.2 | 4.8 | 10 |
| GO Biological Processes | fever generation | -3.5 | 5.2 | 4.8 | 10 |
| GO Biological Processes | collagen-activated tyrosine kinase receptor signaling pathway | -3.5 | 5.2 | 4.8 | 10 |
| GO Biological Processes | mucopolysaccharide metabolic process | -3.5 | 2 | 3.9 | 111 |
| GO Biological Processes | response to nutrient | -3.5 | 1.7 | 3.8 | 218 |
| GO Biological Processes | long-term synaptic potentiation | -3.5 | 2.2 | 4 | 82 |
| GO Biological Processes | cell activation involved in immune response | -3.5 | 1.4 | 3.6 | 705 |
| GO Biological Processes | branching involved in ureteric bud morphogenesis | -3.5 | 2.4 | 4 | 60 |
| GO Biological Processes | glomerulus development | -3.5 | 2.4 | 4 | 60 |
| GO Biological Processes | positive regulation of blood circulation | -3.5 | 2.3 | 4 | 71 |
| GO Biological Processes | negative regulation of neuron apoptotic process | -3.4 | 1.9 | 3.8 | 148 |
| GO Biological Processes | mammary gland formation | -3.4 | 6.2 | 4.9 | 7 |
| GO Biological Processes | alkaloid metabolic process | -3.4 | 6.2 | 4.9 | 7 |
| GO Biological Processes | paracrine signaling | -3.4 | 6.2 | 4.9 | 7 |
| GO Biological Processes | plasma membrane raft organization | -3.4 | 6.2 | 4.9 | 7 |
| GO Biological Processes | regulation of transcription from RNA polymerase II promoter involved in myocardial precursor cell di | -3.4 | 6.2 | 4.9 | 7 |
| GO Biological Processes | cytosolic calcium ion transport | -3.4 | 1.8 | 3.8 | 167 |
| GO Biological Processes | mammary gland development | -3.4 | 1.9 | 3.8 | 142 |
| GO Biological Processes | learning | -3.4 | 1.9 | 3.8 | 142 |
| GO Biological Processes | regulation of vesicle-mediated transport | -3.4 | 1.4 | 3.6 | 524 |
| GO Biological Processes | negative regulation of animal organ morphogenesis | -3.4 | 3 | 4.2 | 35 |
| GO Biological Processes | negative regulation of leukocyte proliferation | -3.4 | 2.2 | 3.9 | 77 |
| GO Biological Processes | nitric oxide metabolic process | -3.4 | 2.2 | 3.9 | 77 |
| GO Biological Processes | response to alkaloid | -3.4 | 2 | 3.9 | 106 |
| GO Biological Processes | osteoblast differentiation | -3.4 | 1.7 | 3.7 | 213 |
| GO Biological Processes | negative regulation of axon extension involved in axon guidance | -3.4 | 3.3 | 4.3 | 26 |
| GO Biological Processes | trabecula formation | -3.4 | 3.3 | 4.3 | 26 |
| GO Biological Processes | carbohydrate transport | -3.4 | 1.9 | 3.8 | 149 |
| GO Biological Processes | regulation of Ras protein signal transduction | -3.4 | 1.7 | 3.7 | 233 |
| GO Biological Processes | cold-induced thermogenesis | -3.4 | 1.9 | 3.8 | 143 |
| GO Biological Processes | regulation of cold-induced thermogenesis | -3.4 | 1.9 | 3.8 | 143 |
| GO Biological Processes | DNA packaging | -3.4 | 1.7 | 3.7 | 207 |
| GO Biological Processes | glycosaminoglycan catabolic process | -3.4 | 2.4 | 4 | 61 |
| GO Biological Processes | negative regulation of lymphocyte proliferation | -3.4 | 2.3 | 3.9 | 72 |

| | | | | | |
|---|---|---|---|---|---|
| GO Biological Processes | negative regulation of mononuclear cell proliferation | -3.4 | 2.3 | 3.9 | 72 |
| GO Biological Processes | renal tubule development | -3.4 | 2.1 | 3.8 | 95 |
| GO Biological Processes | glycosaminoglycan metabolic process | -3.4 | 1.8 | 3.7 | 156 |
| GO Biological Processes | meiosis I cell cycle process | -3.3 | 2 | 3.8 | 119 |
| GO Biological Processes | regulation of calcium ion-dependent exocytosis | -3.3 | 2 | 3.8 | 113 |
| GO Biological Processes | purinergic nucleotide receptor signaling pathway | -3.3 | 3.5 | 4.3 | 22 |
| GO Biological Processes | male genitalia development | -3.3 | 3.5 | 4.3 | 22 |
| GO Biological Processes | pancreas development | -3.3 | 2.2 | 3.9 | 78 |
| GO Biological Processes | inner dynein arm assembly | -3.3 | 4.3 | 4.5 | 14 |
| GO Biological Processes | positive regulation of inositol phosphate biosynthetic process | -3.3 | 4.3 | 4.5 | 14 |
| GO Biological Processes | DNA replication initiation | -3.3 | 3.1 | 4.2 | 31 |
| GO Biological Processes | regulation of plasma membrane bounded cell projection organization | -3.3 | 1.4 | 3.5 | 674 |
| GO Biological Processes | developmental cell growth | -3.3 | 1.7 | 3.6 | 228 |
| GO Biological Processes | negative regulation of phosphate metabolic process | -3.3 | 1.4 | 3.5 | 572 |
| GO Biological Processes | blood coagulation, intrinsic pathway | -3.3 | 3.8 | 4.4 | 18 |
| GO Biological Processes | regulation of systemic arterial blood pressure by circulatory renin-angiotensin | -3.3 | 3.8 | 4.4 | 18 |
| GO Biological Processes | negative regulation of cell division | -3.3 | 3.8 | 4.4 | 18 |
| GO Biological Processes | endocardial cushion morphogenesis | -3.3 | 2.9 | 4.1 | 36 |
| GO Biological Processes | retinoid metabolic process | -3.3 | 2.1 | 3.8 | 90 |
| GO Biological Processes | negative regulation of phosphorus metabolic process | -3.3 | 1.4 | 3.5 | 573 |
| GO Biological Processes | diterpenoid metabolic process | -3.3 | 2.1 | 3.8 | 96 |
| GO Biological Processes | inner ear morphogenesis | -3.3 | 2.1 | 3.8 | 96 |
| GO Biological Processes | antimicrobial humoral immune response mediated by antimicrobial peptide | -3.3 | 2.2 | 3.9 | 73 |
| GO Biological Processes | somitogenesis | -3.3 | 2.2 | 3.9 | 73 |
| GO Biological Processes | nitric oxide biosynthetic process | -3.3 | 2.2 | 3.9 | 73 |
| GO Biological Processes | adaptive thermogenesis | -3.3 | 1.8 | 3.7 | 151 |
| GO Biological Processes | myelination | -3.3 | 1.9 | 3.7 | 120 |
| GO Biological Processes | formation of primary germ layer | -3.3 | 1.9 | 3.7 | 120 |
| GO Biological Processes | regulation of sodium ion transmembrane transport | -3.3 | 2.4 | 3.9 | 62 |
| GO Biological Processes | female sex differentiation | -3.3 | 2 | 3.7 | 114 |
| GO Biological Processes | regulation of calcium ion transmembrane transport | -3.3 | 1.8 | 3.7 | 145 |
| GO Biological Processes | neurotransmitter secretion | -3.3 | 1.8 | 3.7 | 164 |
| GO Biological Processes | interleukin-1 beta production | -3.3 | 2.2 | 3.8 | 79 |
| GO Biological Processes | positive regulation of neurological system process | -3.2 | 2.4 | 3.9 | 57 |
| GO Biological Processes | heart formation | -3.2 | 3.2 | 4.1 | 27 |
| GO Biological Processes | regulation of neural precursor cell proliferation | -3.2 | 2.1 | 3.8 | 85 |
| GO Biological Processes | regulation of tissue remodeling | -3.2 | 2.1 | 3.8 | 85 |
| GO Biological Processes | cell cycle G1/S phase transition | -3.2 | 1.6 | 3.5 | 291 |
| GO Biological Processes | regulation of cyclin-dependent protein serine/threonine kinase activity | -3.2 | 2 | 3.7 | 97 |
| GO Biological Processes | positive regulation of cellular component biogenesis | -3.2 | 1.4 | 3.5 | 532 |
| GO Biological Processes | signal release from synapse | -3.2 | 1.8 | 3.6 | 165 |
| GO Biological Processes | positive regulation of mitotic cell cycle | -3.2 | 1.8 | 3.6 | 165 |
| GO Biological Processes | cardiac muscle tissue growth | -3.2 | 2 | 3.7 | 103 |

| | | | | | |
|---|---|---|---|---|---|
| GO Biological Processes | segmentation | -3.2 | 2 | 3.7 | 103 |
| GO Biological Processes | adherens junction organization | -3.2 | 1.8 | 3.6 | 140 |
| GO Biological Processes | hydrogen peroxide catabolic process | -3.2 | 3 | 4 | 32 |
| GO Biological Processes | regulation of axon extension involved in axon guidance | -3.2 | 3 | 4 | 32 |
| GO Biological Processes | neuron recognition | -3.2 | 2.6 | 3.9 | 47 |
| GO Biological Processes | endocrine hormone secretion | -3.2 | 2.6 | 3.9 | 47 |
| GO Biological Processes | pallium development | -3.2 | 1.8 | 3.6 | 172 |
| GO Biological Processes | negative regulation of axon extension | -3.2 | 2.7 | 3.9 | 42 |
| GO Biological Processes | presynapse assembly | -3.2 | 2.7 | 3.9 | 42 |
| GO Biological Processes | positive regulation of phospholipase C activity | -3.2 | 2.7 | 3.9 | 42 |
| GO Biological Processes | membrane depolarization during action potential | -3.2 | 2.8 | 4 | 37 |
| GO Biological Processes | embryonic camera-type eye development | -3.2 | 2.8 | 4 | 37 |
| GO Biological Processes | anaphase-promoting complex-dependent catabolic process | -3.2 | 2.8 | 4 | 37 |
| GO Biological Processes | regulation of leukocyte differentiation | -3.2 | 1.6 | 3.5 | 272 |
| GO Biological Processes | G1/S transition of mitotic cell cycle | -3.2 | 1.6 | 3.5 | 272 |
| GO Biological Processes | limb bud formation | -3.2 | 4.7 | 4.4 | 11 |
| GO Biological Processes | oligopeptide transmembrane transport | -3.2 | 4.7 | 4.4 | 11 |
| GO Biological Processes | regulation of angiotensin levels in blood | -3.2 | 4.7 | 4.4 | 11 |
| GO Biological Processes | positive regulation of T cell differentiation in thymus | -3.2 | 4.7 | 4.4 | 11 |
| GO Biological Processes | angiotensin maturation | -3.2 | 4.7 | 4.4 | 11 |
| GO Biological Processes | positive regulation of heat generation | -3.2 | 4.7 | 4.4 | 11 |
| GO Biological Processes | cellular response to purine-containing compound | -3.2 | 4.7 | 4.4 | 11 |
| GO Biological Processes | calcium-independent cell-cell adhesion via plasma membrane cell-adhesion molecules | -3.2 | 3.4 | 4.1 | 23 |
| GO Biological Processes | glomerulus vasculature development | -3.2 | 3.4 | 4.1 | 23 |
| GO Biological Processes | negative regulation of cell activation | -3.2 | 1.7 | 3.6 | 192 |
| GO Biological Processes | sprouting angiogenesis | -3.2 | 1.7 | 3.6 | 192 |
| GO Biological Processes | regulation of G protein-coupled receptor signaling pathway | -3.2 | 1.8 | 3.6 | 147 |
| GO Biological Processes | negative regulation of synaptic transmission | -3.2 | 2.2 | 3.8 | 69 |
| GO Biological Processes | neuron apoptotic process | -3.2 | 1.6 | 3.5 | 232 |
| GO Biological Processes | cellular extravasation | -3.2 | 2.4 | 3.8 | 58 |
| GO Biological Processes | regulation of macrophage activation | -3.2 | 2.4 | 3.8 | 58 |
| GO Biological Processes | positive regulation of lipid metabolic process | -3.1 | 1.8 | 3.6 | 141 |
| GO Biological Processes | cellular response to decreased oxygen levels | -3.1 | 1.7 | 3.6 | 173 |
| GO Biological Processes | negative regulation of neuron differentiation | -3.1 | 1.7 | 3.5 | 219 |
| GO Biological Processes | peripheral nervous system development | -3.1 | 2.2 | 3.7 | 75 |
| GO Biological Processes | monocarboxylic acid metabolic process | -3.1 | 1.4 | 3.4 | 594 |
| GO Biological Processes | axon ensheathment in central nervous system | -3.1 | 3.6 | 4.2 | 19 |
| GO Biological Processes | central nervous system myelination | -3.1 | 3.6 | 4.2 | 19 |
| GO Biological Processes | glomerular filtration | -3.1 | 3.6 | 4.2 | 19 |
| GO Biological Processes | collagen fibril organization | -3.1 | 2.4 | 3.8 | 53 |
| GO Biological Processes | primary alcohol catabolic process | -3.1 | 4 | 4.2 | 15 |
| GO Biological Processes | serotonin transport | -3.1 | 4 | 4.2 | 15 |
| GO Biological Processes | regulation of killing of cells of other organism | -3.1 | 4 | 4.2 | 15 |
| GO Biological Processes | oxygen transport | -3.1 | 4 | 4.2 | 15 |

| | | | | | |
|---|---|---|---|---|---|
| GO Biological Processes | positive regulation of alcohol biosynthetic process | -3.1 | 3.1 | 4 | 28 |
| GO Biological Processes | prostate gland morphogenesis | -3.1 | 3.1 | 4 | 28 |
| GO Biological Processes | negative regulation of cytoskeleton organization | -3.1 | 1.8 | 3.6 | 142 |
| GO Biological Processes | embryonic skeletal system morphogenesis | -3.1 | 2 | 3.6 | 93 |
| GO Biological Processes | regulation of glutamate receptor signaling pathway | -3.1 | 2.5 | 3.8 | 48 |
| GO Biological Processes | leukocyte differentiation | -3.1 | 1.4 | 3.4 | 508 |
| GO Biological Processes | positive regulation of canonical Wnt signaling pathway | -3.1 | 2 | 3.6 | 99 |
| GO Biological Processes | epithelial cell development | -3.1 | 1.7 | 3.5 | 207 |
| GO Biological Processes | glomerular mesangial cell differentiation | -3.1 | 6.9 | 4.8 | 5 |
| GO Biological Processes | positive regulation of transcription from RNA polymerase II promoter involved in heart development | -3.1 | 6.9 | 4.8 | 5 |
| GO Biological Processes | dipeptide transmembrane transport | -3.1 | 6.9 | 4.8 | 5 |
| GO Biological Processes | regulation of eosinophil migration | -3.1 | 6.9 | 4.8 | 5 |
| GO Biological Processes | cellular response to heparin | -3.1 | 6.9 | 4.8 | 5 |
| GO Biological Processes | interleukin-1 beta biosynthetic process | -3.1 | 6.9 | 4.8 | 5 |
| GO Biological Processes | integrin biosynthetic process | -3.1 | 6.9 | 4.8 | 5 |
| GO Biological Processes | twitch skeletal muscle contraction | -3.1 | 6.9 | 4.8 | 5 |
| GO Biological Processes | interleukin-1 biosynthetic process | -3.1 | 6.9 | 4.8 | 5 |
| GO Biological Processes | caveola assembly | -3.1 | 6.9 | 4.8 | 5 |
| GO Biological Processes | dipeptide transport | -3.1 | 6.9 | 4.8 | 5 |
| GO Biological Processes | voluntary skeletal muscle contraction | -3.1 | 6.9 | 4.8 | 5 |
| GO Biological Processes | ductus arteriosus closure | -3.1 | 6.9 | 4.8 | 5 |
| GO Biological Processes | regulation of acute inflammatory response | -3.1 | 1.8 | 3.5 | 155 |
| GO Biological Processes | cellular response to retinoic acid | -3.1 | 2.2 | 3.7 | 70 |
| GO Biological Processes | chromosome condensation | -3.1 | 2.6 | 3.8 | 43 |
| GO Biological Processes | bicarbonate transport | -3.1 | 2.6 | 3.8 | 43 |
| GO Biological Processes | olfactory bulb development | -3.1 | 2.9 | 3.9 | 33 |
| GO Biological Processes | peptide hormone processing | -3.1 | 2.9 | 3.9 | 33 |
| GO Biological Processes | negative regulation of neuron projection development | -3.1 | 1.8 | 3.5 | 149 |
| GO Biological Processes | response to anticoagulant | -3.1 | 5.4 | 4.5 | 8 |
| GO Biological Processes | positive regulation of epithelial cell proliferation involved in wound healing | -3.1 | 5.4 | 4.5 | 8 |
| GO Biological Processes | regulation of cardioblast differentiation | -3.1 | 5.4 | 4.5 | 8 |
| GO Biological Processes | regulation of fever generation | -3.1 | 5.4 | 4.5 | 8 |
| GO Biological Processes | heart induction | -3.1 | 5.4 | 4.5 | 8 |
| GO Biological Processes | positive regulation of cardiac epithelial to mesenchymal transition | -3.1 | 5.4 | 4.5 | 8 |
| GO Biological Processes | regulation of hormone metabolic process | -3.1 | 2.7 | 3.8 | 38 |
| GO Biological Processes | vitamin transport | -3.1 | 2.7 | 3.8 | 38 |
| GO Biological Processes | leukocyte proliferation | -3.1 | 1.6 | 3.4 | 289 |
| GO Biological Processes | diencephalon development | -3.1 | 2.2 | 3.7 | 76 |
| GO Biological Processes | negative regulation of chromosome organization | -3 | 1.8 | 3.5 | 143 |
| GO Biological Processes | carbohydrate metabolic process | -3 | 1.4 | 3.3 | 583 |
| GO Biological Processes | cornification | -3 | 1.9 | 3.5 | 112 |
| GO Biological Processes | cell-cell junction organization | -3 | 1.8 | 3.5 | 156 |
| GO Biological Processes | gastrulation | -3 | 1.7 | 3.5 | 182 |
| GO Biological Processes | pathway-restricted SMAD protein phosphorylation | -3 | 2.3 | 3.7 | 65 |

| | | | | | |
|---|---|---|---|---|---|
| GO Biological Processes | neuromuscular process | -3 | 2 | 3.6 | 106 |
| GO Biological Processes | regulation of neurotransmitter secretion | -3 | 2 | 3.6 | 106 |
| GO Biological Processes | response to retinoic acid | -3 | 2 | 3.6 | 106 |
| GO Biological Processes | cellular response to toxic substance | -3 | 1.6 | 3.4 | 242 |
| GO Biological Processes | positive regulation of chemokine production | -3 | 2.4 | 3.7 | 54 |
| GO Biological Processes | multicellular organismal movement | -3 | 2.4 | 3.7 | 54 |
| GO Biological Processes | musculoskeletal movement | -3 | 2.4 | 3.7 | 54 |
| GO Biological Processes | regulation of cardiac muscle tissue development | -3 | 2 | 3.6 | 100 |
| GO Biological Processes | secondary palate development | -3 | 3.2 | 4 | 24 |
| GO Biological Processes | endothelial cell migration | -3 | 1.6 | 3.4 | 270 |
| GO Biological Processes | regulation of chondrocyte differentiation | -3 | 2.5 | 3.7 | 49 |
| GO Biological Processes | retina morphogenesis in camera-type eye | -3 | 2.5 | 3.7 | 49 |
| GO Biological Processes | smooth muscle cell differentiation | -3 | 2.1 | 3.6 | 77 |
| GO Biological Processes | DNA geometric change | -3 | 2.1 | 3.6 | 77 |
| GO Biological Processes | regulation of smooth muscle cell proliferation | -3 | 1.7 | 3.4 | 170 |
| GO Biological Processes | hexose transmembrane transport | -3 | 1.9 | 3.5 | 113 |
| GO Biological Processes | regulation of cellular carbohydrate metabolic process | -3 | 1.8 | 3.5 | 138 |
| GO Biological Processes | presynapse organization | -3 | 2.5 | 3.7 | 44 |
| GO Biological Processes | glial cell proliferation | -3 | 2.5 | 3.7 | 44 |
| GO Biological Processes | regulation of endocrine process | -3 | 2.5 | 3.7 | 44 |
| GO Biological Processes | plasma lipoprotein particle remodeling | -3 | 3 | 3.8 | 29 |
| GO Biological Processes | positive regulation of pri-miRNA transcription by RNA polymerase II | -3 | 3 | 3.8 | 29 |
| GO Biological Processes | nitric oxide mediated signal transduction | -3 | 3 | 3.8 | 29 |
| GO Biological Processes | protein-lipid complex remodeling | -3 | 3 | 3.8 | 29 |
| GO Biological Processes | mononuclear cell migration | -3 | 2 | 3.5 | 89 |
| GO Biological Processes | alpha-amino acid catabolic process | -3 | 2 | 3.5 | 101 |
| GO Biological Processes | actin-myosin filament sliding | -2.9 | 2.7 | 3.7 | 39 |
| GO Biological Processes | muscle filament sliding | -2.9 | 2.7 | 3.7 | 39 |
| GO Biological Processes | regulation of vascular permeability | -2.9 | 2.8 | 3.8 | 34 |
| GO Biological Processes | olfactory lobe development | -2.9 | 2.8 | 3.8 | 34 |
| GO Biological Processes | regulation of mesenchymal cell proliferation | -2.9 | 2.8 | 3.8 | 34 |
| GO Biological Processes | mesodermal cell differentiation | -2.9 | 2.8 | 3.8 | 34 |
| GO Biological Processes | arginine metabolic process | -2.9 | 3.4 | 4 | 20 |
| GO Biological Processes | regulation of neurotransmitter uptake | -2.9 | 3.4 | 4 | 20 |
| GO Biological Processes | leukotriene biosynthetic process | -2.9 | 3.4 | 4 | 20 |
| GO Biological Processes | thyroid hormone metabolic process | -2.9 | 3.4 | 4 | 20 |
| GO Biological Processes | renal filtration | -2.9 | 3.4 | 4 | 20 |
| GO Biological Processes | regulation of NMDA receptor activity | -2.9 | 3.4 | 4 | 20 |
| GO Biological Processes | regulation of myeloid cell differentiation | -2.9 | 1.6 | 3.3 | 251 |
| GO Biological Processes | tube formation | -2.9 | 1.8 | 3.4 | 145 |
| GO Biological Processes | positive regulation of developmental growth | -2.9 | 1.7 | 3.4 | 184 |
| GO Biological Processes | aorta development | -2.9 | 2.3 | 3.6 | 55 |
| GO Biological Processes | regulation of neuron apoptotic process | -2.9 | 1.6 | 3.4 | 204 |
| GO Biological Processes | gland morphogenesis | -2.9 | 1.9 | 3.4 | 120 |
| GO Biological Processes | regulation of Rho protein signal transduction | -2.9 | 1.8 | 3.4 | 139 |

| | | | | | |
|---|---|---|---|---|---|
| GO Biological Processes | carbohydrate biosynthetic process | -2.9 | 1.6 | 3.3 | 211 |
| GO Biological Processes | phospholipid efflux | -2.9 | 4.3 | 4.2 | 12 |
| GO Biological Processes | cardiac conduction system development | -2.9 | 4.3 | 4.2 | 12 |
| GO Biological Processes | oligopeptide transport | -2.9 | 4.3 | 4.2 | 12 |
| GO Biological Processes | dopamine biosynthetic process | -2.9 | 4.3 | 4.2 | 12 |
| GO Biological Processes | cardiac chamber formation | -2.9 | 4.3 | 4.2 | 12 |
| GO Biological Processes | ethanol catabolic process | -2.9 | 4.3 | 4.2 | 12 |
| GO Biological Processes | steroid metabolic process | -2.9 | 1.5 | 3.3 | 321 |
| GO Biological Processes | regulation of synaptic vesicle cycle | -2.9 | 1.9 | 3.4 | 114 |
| GO Biological Processes | meiosis I | -2.9 | 1.9 | 3.4 | 114 |
| GO Biological Processes | glucose transmembrane transport | -2.9 | 1.9 | 3.5 | 108 |
| GO Biological Processes | pulmonary valve morphogenesis | -2.9 | 3.8 | 4 | 16 |
| GO Biological Processes | prostaglandin transport | -2.9 | 3.8 | 4 | 16 |
| GO Biological Processes | regulation of inositol phosphate biosynthetic process | -2.9 | 3.8 | 4 | 16 |
| GO Biological Processes | ventricular trabecula myocardium morphogenesis | -2.9 | 3.8 | 4 | 16 |
| GO Biological Processes | mesoderm development | -2.9 | 1.8 | 3.4 | 133 |
| GO Biological Processes | axis specification | -2.9 | 2.1 | 3.5 | 84 |
| GO Biological Processes | cell fate specification | -2.9 | 2.1 | 3.5 | 84 |
| GO Biological Processes | O-glycan processing | -2.9 | 2.3 | 3.6 | 61 |
| GO Biological Processes | smooth muscle cell proliferation | -2.9 | 1.7 | 3.4 | 172 |
| GO Biological Processes | response to ethanol | -2.9 | 1.8 | 3.4 | 127 |
| GO Biological Processes | cytokine-mediated signaling pathway | -2.9 | 1.3 | 3.2 | 739 |
| GO Biological Processes | positive regulation of establishment of protein localization | -2.9 | 1.4 | 3.2 | 429 |
| GO Biological Processes | regulation of DNA-binding transcription factor activity | -2.9 | 1.4 | 3.2 | 422 |
| GO Biological Processes | DNA duplex unwinding | -2.9 | 2.2 | 3.5 | 67 |
| GO Biological Processes | long-chain fatty acid transport | -2.9 | 2.2 | 3.5 | 67 |
| GO Biological Processes | regulation of glial cell differentiation | -2.9 | 2.2 | 3.5 | 67 |
| GO Biological Processes | morphogenesis of an epithelial fold | -2.9 | 3.1 | 3.8 | 25 |
| GO Biological Processes | prostanoid biosynthetic process | -2.9 | 3.1 | 3.8 | 25 |
| GO Biological Processes | positive regulation of positive chemotaxis | -2.9 | 3.1 | 3.8 | 25 |
| GO Biological Processes | prostaglandin biosynthetic process | -2.9 | 3.1 | 3.8 | 25 |
| GO Biological Processes | kidney vasculature development | -2.9 | 3.1 | 3.8 | 25 |
| GO Biological Processes | renal system vasculature development | -2.9 | 3.1 | 3.8 | 25 |
| GO Biological Processes | positive regulation of DNA-binding transcription factor activity | -2.9 | 1.6 | 3.3 | 260 |
| GO Biological Processes | regulation of actin filament organization | -2.9 | 1.6 | 3.3 | 260 |
| GO Biological Processes | regulation of phospholipase C activity | -2.9 | 2.5 | 3.6 | 45 |
| GO Biological Processes | ventricular septum morphogenesis | -2.9 | 2.5 | 3.6 | 45 |
| GO Biological Processes | endothelial cell proliferation | -2.9 | 1.6 | 3.3 | 199 |
| GO Biological Processes | hindbrain development | -2.9 | 1.7 | 3.4 | 153 |
| GO Biological Processes | regulation of lipid localization | -2.9 | 1.7 | 3.4 | 153 |
| GO Biological Processes | negative regulation of inflammatory response | -2.9 | 1.7 | 3.4 | 153 |
| GO Biological Processes | monosaccharide transmembrane transport | -2.9 | 1.9 | 3.4 | 115 |
| GO Biological Processes | regulation of endocytosis | -2.9 | 1.5 | 3.3 | 274 |
| GO Biological Processes | cardiac muscle contraction | -2.9 | 1.8 | 3.4 | 134 |
| GO Biological Processes | regulation of leukocyte migration | -2.9 | 1.7 | 3.3 | 186 |

| | | | | | |
|---|---|---|---|---|---|
| GO Biological Processes | cardiac muscle cell action potential involved in contraction | -2.9 | 2.3 | 3.5 | 56 |
| GO Biological Processes | long-chain fatty acid metabolic process | -2.8 | 1.9 | 3.4 | 109 |
| GO Biological Processes | positive regulation of transmembrane receptor protein serine/threonine kinase signaling pathway | -2.8 | 1.9 | 3.4 | 103 |
| GO Biological Processes | regulation of establishment of protein localization | -2.8 | 1.3 | 3.1 | 711 |
| GO Biological Processes | regulation of platelet activation | -2.8 | 2.9 | 3.7 | 30 |
| GO Biological Processes | regulation of macrophage derived foam cell differentiation | -2.8 | 2.9 | 3.7 | 30 |
| GO Biological Processes | protein-containing complex remodeling | -2.8 | 2.9 | 3.7 | 30 |
| GO Biological Processes | regulation of cytokine biosynthetic process | -2.8 | 2 | 3.4 | 97 |
| GO Biological Processes | response to fatty acid | -2.8 | 2 | 3.4 | 85 |
| GO Biological Processes | positive regulation of interleukin-6 production | -2.8 | 2 | 3.4 | 85 |
| GO Biological Processes | fat cell differentiation | -2.8 | 1.6 | 3.3 | 220 |
| GO Biological Processes | transforming growth factor beta receptor signaling pathway | -2.8 | 1.6 | 3.3 | 200 |
| GO Biological Processes | protein localization to cell surface | -2.8 | 2.2 | 3.5 | 62 |
| GO Biological Processes | sodium ion homeostasis | -2.8 | 2.4 | 3.5 | 51 |
| GO Biological Processes | positive regulation of transcription from RNA polymerase II promoter involved in smooth muscle cell | -2.8 | 8.6 | 4.8 | 3 |
| GO Biological Processes | positive regulation of antimicrobial peptide production | -2.8 | 8.6 | 4.8 | 3 |
| GO Biological Processes | bronchiole development | -2.8 | 8.6 | 4.8 | 3 |
| GO Biological Processes | apoptotic process involved in luteolysis | -2.8 | 8.6 | 4.8 | 3 |
| GO Biological Processes | positive regulation of the force of heart contraction by chemical signal | -2.8 | 8.6 | 4.8 | 3 |
| GO Biological Processes | nitric oxide transport | -2.8 | 8.6 | 4.8 | 3 |
| GO Biological Processes | senescence-associated heterochromatin focus assembly | -2.8 | 8.6 | 4.8 | 3 |
| GO Biological Processes | cellular response to interleukin-8 | -2.8 | 8.6 | 4.8 | 3 |
| GO Biological Processes | low-density lipoprotein particle mediated signaling | -2.8 | 8.6 | 4.8 | 3 |
| GO Biological Processes | negative regulation of endodermal cell differentiation | -2.8 | 8.6 | 4.8 | 3 |
| GO Biological Processes | lipoprotein particle mediated signaling | -2.8 | 8.6 | 4.8 | 3 |
| GO Biological Processes | cardiac cell fate specification | -2.8 | 8.6 | 4.8 | 3 |
| GO Biological Processes | negative regulation of serotonin uptake | -2.8 | 8.6 | 4.8 | 3 |
| GO Biological Processes | mammary gland bud morphogenesis | -2.8 | 8.6 | 4.8 | 3 |
| GO Biological Processes | positive regulation of integrin biosynthetic process | -2.8 | 8.6 | 4.8 | 3 |
| GO Biological Processes | animal organ senescence | -2.8 | 8.6 | 4.8 | 3 |
| GO Biological Processes | regulation of serotonin uptake | -2.8 | 8.6 | 4.8 | 3 |
| GO Biological Processes | mammary placode formation | -2.8 | 8.6 | 4.8 | 3 |
| GO Biological Processes | positive regulation of eosinophil migration | -2.8 | 8.6 | 4.8 | 3 |
| GO Biological Processes | positive regulation of antibacterial peptide production | -2.8 | 8.6 | 4.8 | 3 |
| GO Biological Processes | response to interleukin-8 | -2.8 | 8.6 | 4.8 | 3 |
| GO Biological Processes | renal system process | -2.8 | 1.9 | 3.4 | 116 |
| GO Biological Processes | peptide hormone secretion | -2.8 | 1.6 | 3.2 | 248 |
| GO Biological Processes | regulation of protein serine/threonine kinase activity | -2.8 | 1.4 | 3.1 | 512 |
| GO Biological Processes | negative regulation of supramolecular fiber organization | -2.8 | 1.8 | 3.3 | 135 |
| GO Biological Processes | regulation of ossification | -2.8 | 1.6 | 3.3 | 194 |
| GO Biological Processes | bone morphogenesis | -2.8 | 1.9 | 3.4 | 110 |
| GO Biological Processes | response to hyperoxia | -2.8 | 3.3 | 3.8 | 21 |

| | | | | | |
|---|---|---|---|---|---|
| GO Biological Processes | smooth muscle tissue development | -2.8 | 3.3 | 3.8 | 21 |
| GO Biological Processes | phasic smooth muscle contraction | -2.8 | 3.3 | 3.8 | 21 |
| GO Biological Processes | response to ammonium ion | -2.8 | 1.8 | 3.3 | 129 |
| GO Biological Processes | positive regulation of hormone secretion | -2.8 | 1.8 | 3.3 | 129 |
| GO Biological Processes | regulation of carbohydrate metabolic process | -2.8 | 1.7 | 3.3 | 168 |
| GO Biological Processes | adult behavior | -2.8 | 1.8 | 3.3 | 142 |
| GO Biological Processes | retina development in camera-type eye | -2.8 | 1.8 | 3.3 | 142 |
| GO Biological Processes | negative regulation of ion transport | -2.8 | 1.7 | 3.3 | 155 |
| GO Biological Processes | genitalia development | -2.8 | 2.4 | 3.5 | 46 |
| GO Biological Processes | regulation of interleukin-10 production | -2.8 | 2.4 | 3.5 | 46 |
| GO Biological Processes | positive regulation of myeloid cell differentiation | -2.8 | 2 | 3.4 | 92 |
| GO Biological Processes | regulation of lipid transport | -2.8 | 1.8 | 3.3 | 123 |
| GO Biological Processes | regulation of mitotic cell cycle | -2.8 | 1.3 | 3.1 | 625 |
| GO Biological Processes | synaptic vesicle cycle | -2.8 | 1.6 | 3.2 | 195 |
| GO Biological Processes | regulation of glomerular filtration | -2.8 | 4.8 | 4.1 | 9 |
| GO Biological Processes | iris morphogenesis | -2.8 | 4.8 | 4.1 | 9 |
| GO Biological Processes | mitotic spindle elongation | -2.8 | 4.8 | 4.1 | 9 |
| GO Biological Processes | urea transport | -2.8 | 4.8 | 4.1 | 9 |
| GO Biological Processes | regulation of somitogenesis | -2.8 | 4.8 | 4.1 | 9 |
| GO Biological Processes | carbohydrate transmembrane transport | -2.8 | 1.8 | 3.3 | 117 |
| GO Biological Processes | positive regulation of endocytosis | -2.7 | 1.7 | 3.3 | 149 |
| GO Biological Processes | regulation of cell cycle phase transition | -2.7 | 1.4 | 3.1 | 434 |
| GO Biological Processes | regulation of mitotic cell cycle phase transition | -2.7 | 1.4 | 3.1 | 398 |
| GO Biological Processes | response to ischemia | -2.7 | 2.5 | 3.5 | 41 |
| GO Biological Processes | metanephric nephron development | -2.7 | 2.5 | 3.5 | 41 |
| GO Biological Processes | respiratory gaseous exchange | -2.7 | 2.2 | 3.4 | 63 |
| GO Biological Processes | embryonic camera-type eye morphogenesis | -2.7 | 3 | 3.7 | 26 |
| GO Biological Processes | positive regulation of heart rate | -2.7 | 3 | 3.7 | 26 |
| GO Biological Processes | prostate gland epithelium morphogenesis | -2.7 | 3 | 3.7 | 26 |
| GO Biological Processes | regulation of positive chemotaxis | -2.7 | 3 | 3.7 | 26 |
| GO Biological Processes | negative regulation of leukocyte activation | -2.7 | 1.7 | 3.2 | 169 |
| GO Biological Processes | neuropeptide signaling pathway | -2.7 | 1.9 | 3.3 | 105 |
| GO Biological Processes | hydrogen peroxide biosynthetic process | -2.7 | 3.5 | 3.8 | 17 |
| GO Biological Processes | chondrocyte proliferation | -2.7 | 3.5 | 3.8 | 17 |
| GO Biological Processes | sulfation | -2.7 | 3.5 | 3.8 | 17 |
| GO Biological Processes | positive regulation of macrophage derived foam cell differentiation | -2.7 | 3.5 | 3.8 | 17 |
| GO Biological Processes | hindlimb morphogenesis | -2.7 | 2.6 | 3.6 | 36 |
| GO Biological Processes | cardiac epithelial to mesenchymal transition | -2.7 | 2.6 | 3.6 | 36 |
| GO Biological Processes | regulation of sodium ion transmembrane transporter activity | -2.7 | 2.3 | 3.5 | 52 |
| GO Biological Processes | positive regulation of mitotic nuclear division | -2.7 | 2.3 | 3.5 | 52 |
| GO Biological Processes | water homeostasis | -2.7 | 2.1 | 3.4 | 69 |
| GO Biological Processes | regulation of cartilage development | -2.7 | 2.1 | 3.4 | 69 |
| GO Biological Processes | cellular ketone metabolic process | -2.7 | 1.6 | 3.2 | 196 |
| GO Biological Processes | excitatory postsynaptic potential | -2.7 | 1.9 | 3.3 | 99 |
| GO Biological Processes | positive regulation of acute inflammatory response | -2.7 | 2.8 | 3.6 | 31 |

| | | | | | |
|---|---|---|---|---|---|
| GO Biological Processes | regulation of vascular endothelial growth factor production | -2.7 | 2.8 | 3.6 | 31 |
| GO Biological Processes | leukotriene metabolic process | -2.7 | 2.8 | 3.6 | 31 |
| GO Biological Processes | membrane assembly | -2.7 | 2.8 | 3.6 | 31 |
| GO Biological Processes | striated muscle cell proliferation | -2.7 | 2 | 3.3 | 81 |
| GO Biological Processes | hyaluronan biosynthetic process | -2.7 | 4 | 3.9 | 13 |
| GO Biological Processes | positive regulation of glutamate receptor signaling pathway | -2.7 | 4 | 3.9 | 13 |
| GO Biological Processes | negative regulation of mitotic cell cycle | -2.7 | 1.5 | 3.1 | 286 |
| GO Biological Processes | positive regulation of wound healing | -2.7 | 2.2 | 3.4 | 58 |
| GO Biological Processes | epithelial tube formation | -2.7 | 1.8 | 3.2 | 131 |
| GO Biological Processes | antibacterial humoral response | -2.7 | 2.4 | 3.4 | 47 |
| GO Biological Processes | acute-phase response | -2.7 | 2.4 | 3.4 | 47 |
| GO Biological Processes | in utero embryonic development | -2.7 | 1.5 | 3.1 | 343 |
| GO Biological Processes | regulation of extent of cell growth | -2.7 | 1.9 | 3.3 | 106 |
| GO Biological Processes | positive regulation of transporter activity | -2.7 | 1.9 | 3.3 | 106 |
| GO Biological Processes | synaptic vesicle localization | -2.7 | 1.7 | 3.2 | 164 |
| GO Biological Processes | spindle organization | -2.7 | 1.6 | 3.2 | 184 |
| GO Biological Processes | calcium ion transmembrane import into cytosol | -2.7 | 1.7 | 3.2 | 138 |
| GO Biological Processes | plasma membrane raft assembly | -2.7 | 5.7 | 4.2 | 6 |
| GO Biological Processes | mineralocorticoid secretion | -2.7 | 5.7 | 4.2 | 6 |
| GO Biological Processes | aldosterone secretion | -2.7 | 5.7 | 4.2 | 6 |
| GO Biological Processes | mesangial cell differentiation | -2.7 | 5.7 | 4.2 | 6 |
| GO Biological Processes | regulation of heart induction | -2.7 | 5.7 | 4.2 | 6 |
| GO Biological Processes | regulation of aldosterone secretion | -2.7 | 5.7 | 4.2 | 6 |
| GO Biological Processes | regulation of the force of heart contraction by chemical signal | -2.7 | 5.7 | 4.2 | 6 |
| GO Biological Processes | protein K29-linked ubiquitination | -2.7 | 5.7 | 4.2 | 6 |
| GO Biological Processes | regulation of mineralocorticoid secretion | -2.7 | 5.7 | 4.2 | 6 |
| GO Biological Processes | regulation of exocytosis | -2.6 | 1.6 | 3.1 | 218 |
| GO Biological Processes | endochondral bone morphogenesis | -2.6 | 2.1 | 3.3 | 70 |
| GO Biological Processes | negative regulation of protein phosphorylation | -2.6 | 1.4 | 3 | 416 |
| GO Biological Processes | inositol phosphate biosynthetic process | -2.6 | 2.5 | 3.4 | 42 |
| GO Biological Processes | pituitary gland development | -2.6 | 2.5 | 3.4 | 42 |
| GO Biological Processes | cell fate determination | -2.6 | 2.5 | 3.4 | 42 |
| GO Biological Processes | regulation of chemokine production | -2.6 | 2 | 3.3 | 76 |
| GO Biological Processes | regulation of interleukin-1 production | -2.6 | 2 | 3.3 | 82 |
| GO Biological Processes | positive regulation of heart growth | -2.6 | 2.3 | 3.4 | 53 |
| GO Biological Processes | positive regulation of neural precursor cell proliferation | -2.6 | 2.3 | 3.4 | 53 |
| GO Biological Processes | regulation of cell maturation | -2.6 | 3.1 | 3.6 | 22 |
| GO Biological Processes | negative regulation of protein secretion | -2.6 | 1.8 | 3.2 | 132 |
| GO Biological Processes | myeloid cell differentiation | -2.6 | 1.4 | 3 | 410 |
| GO Biological Processes | interferon-gamma production | -2.6 | 1.9 | 3.2 | 107 |
| GO Biological Processes | chemical synaptic transmission, postsynaptic | -2.6 | 1.9 | 3.2 | 107 |
| GO Biological Processes | negative regulation of phosphorylation | -2.6 | 1.4 | 3 | 454 |
| GO Biological Processes | negative regulation of peptide secretion | -2.6 | 1.7 | 3.2 | 139 |
| GO Biological Processes | female pregnancy | -2.6 | 1.6 | 3.1 | 192 |

| | | | | | |
|---|---|---|---|---|---|
| GO Biological Processes | neuroinflammatory response | -2.6 | 2.2 | 3.3 | 59 |
| GO Biological Processes | neuroblast proliferation | -2.6 | 2.2 | 3.3 | 59 |
| GO Biological Processes | endochondral ossification | -2.6 | 2.9 | 3.5 | 27 |
| GO Biological Processes | replacement ossification | -2.6 | 2.9 | 3.5 | 27 |
| GO Biological Processes | response to angiotensin | -2.6 | 2.9 | 3.5 | 27 |
| GO Biological Processes | wound healing, spreading of cells | -2.6 | 2.7 | 3.5 | 32 |
| GO Biological Processes | epiboly involved in wound healing | -2.6 | 2.7 | 3.5 | 32 |
| GO Biological Processes | regulation of response to drug | -2.6 | 1.9 | 3.2 | 101 |
| GO Biological Processes | regulation of cell activation | -2.6 | 1.3 | 3 | 610 |
| GO Biological Processes | chondrocyte development | -2.6 | 2.3 | 3.4 | 48 |
| GO Biological Processes | intermediate filament-based process | -2.6 | 2.3 | 3.4 | 48 |
| GO Biological Processes | interleukin-10 production | -2.6 | 2.3 | 3.4 | 48 |
| GO Biological Processes | multicellular organismal water homeostasis | -2.6 | 2.1 | 3.3 | 65 |
| GO Biological Processes | negative regulation of protein modification process | -2.6 | 1.3 | 3 | 603 |
| GO Biological Processes | negative regulation of canonical Wnt signaling pathway | -2.6 | 1.7 | 3.1 | 133 |
| GO Biological Processes | positive regulation of hemopoiesis | -2.6 | 1.6 | 3.1 | 186 |
| GO Biological Processes | development of primary sexual characteristics | -2.6 | 1.6 | 3.1 | 220 |
| GO Biological Processes | chemokine production | -2.6 | 2 | 3.2 | 83 |
| GO Biological Processes | memory | -2.6 | 1.8 | 3.2 | 114 |
| GO Biological Processes | positive regulation of neuron differentiation | -2.6 | 1.4 | 3 | 361 |
| GO Biological Processes | isoprenoid metabolic process | -2.6 | 1.8 | 3.1 | 127 |
| GO Biological Processes | response to peptide | -2.6 | 1.4 | 3 | 500 |
| GO Biological Processes | positive regulation of protein kinase B signaling | -2.6 | 1.6 | 3.1 | 173 |
| GO Biological Processes | monovalent inorganic cation homeostasis | -2.6 | 1.7 | 3.1 | 140 |
| GO Biological Processes | cytokine biosynthetic process | -2.6 | 1.8 | 3.2 | 108 |
| GO Biological Processes | centromere complex assembly | -2.6 | 2.2 | 3.3 | 54 |
| GO Biological Processes | cell differentiation involved in kidney development | -2.6 | 2.2 | 3.3 | 54 |
| GO Biological Processes | disruption of cells of other organism involved in symbiotic interaction | -2.6 | 3.4 | 3.6 | 18 |
| GO Biological Processes | response to vitamin A | -2.6 | 3.4 | 3.6 | 18 |
| GO Biological Processes | mammary gland lobule development | -2.6 | 3.4 | 3.6 | 18 |
| GO Biological Processes | mammary gland alveolus development | -2.6 | 3.4 | 3.6 | 18 |
| GO Biological Processes | regulation of binding | -2.5 | 1.4 | 3 | 369 |
| GO Biological Processes | cellular response to hormone stimulus | -2.5 | 1.3 | 2.9 | 703 |
| GO Biological Processes | regulation of reactive oxygen species metabolic process | -2.5 | 1.6 | 3 | 187 |
| GO Biological Processes | protein localization to cell periphery | -2.5 | 1.5 | 3 | 305 |
| GO Biological Processes | response to BMP | -2.5 | 1.7 | 3.1 | 167 |
| GO Biological Processes | cellular response to BMP stimulus | -2.5 | 1.7 | 3.1 | 167 |
| GO Biological Processes | cell-substrate junction assembly | -2.5 | 1.9 | 3.1 | 96 |
| GO Biological Processes | somatic stem cell population maintenance | -2.5 | 2.2 | 3.2 | 60 |
| GO Biological Processes | organic hydroxy compound biosynthetic process | -2.5 | 1.5 | 3 | 270 |
| GO Biological Processes | regulation of behavior | -2.5 | 2.1 | 3.2 | 66 |
| GO Biological Processes | regulation of cardiocyte differentiation | -2.5 | 2.1 | 3.2 | 66 |
| GO Biological Processes | monocyte chemotaxis | -2.5 | 2.1 | 3.2 | 66 |
| GO Biological Processes | histone phosphorylation | -2.5 | 2.5 | 3.3 | 38 |
| GO Biological Processes | leukocyte activation involved in inflammatory response | -2.5 | 2.5 | 3.3 | 38 |

| | | | | | |
|---|---|---|---|---|---|
| GO Biological Processes | mating | -2.5 | 2.5 | 3.3 | 38 |
| GO Biological Processes | microglial cell activation | -2.5 | 2.5 | 3.3 | 38 |
| GO Biological Processes | regulation of cellular amine metabolic process | -2.5 | 2.5 | 3.3 | 38 |
| GO Biological Processes | myoblast differentiation | -2.5 | 1.9 | 3.2 | 84 |
| GO Biological Processes | regulation of bone mineralization | -2.5 | 2 | 3.2 | 72 |
| GO Biological Processes | response to estrogen | -2.5 | 2 | 3.2 | 72 |
| GO Biological Processes | regulation of regulated secretory pathway | -2.5 | 1.7 | 3 | 161 |
| GO Biological Processes | cytokine metabolic process | -2.5 | 1.8 | 3.1 | 109 |
| GO Biological Processes | positive regulation of anion transport | -2.5 | 2.3 | 3.3 | 49 |
| GO Biological Processes | positive regulation of epithelial to mesenchymal transition | -2.5 | 2.3 | 3.3 | 49 |
| GO Biological Processes | coronary vasculature development | -2.5 | 2.3 | 3.3 | 49 |
| GO Biological Processes | positive regulation of cardiac muscle tissue growth | -2.5 | 2.3 | 3.3 | 49 |
| GO Biological Processes | response to reactive oxygen species | -2.5 | 1.5 | 3 | 229 |
| GO Biological Processes | regulation of spindle checkpoint | -2.5 | 3.7 | 3.7 | 14 |
| GO Biological Processes | prostaglandin secretion | -2.5 | 3.7 | 3.7 | 14 |
| GO Biological Processes | disruption by host of symbiont cells | -2.5 | 3.7 | 3.7 | 14 |
| GO Biological Processes | regulation of transcription from RNA polymerase II promoter involved in heart development | -2.5 | 3.7 | 3.7 | 14 |
| GO Biological Processes | olfactory bulb interneuron differentiation | -2.5 | 3.7 | 3.7 | 14 |
| GO Biological Processes | angiotensin-activated signaling pathway | -2.5 | 3.7 | 3.7 | 14 |
| GO Biological Processes | regulation of fibrinolysis | -2.5 | 3.7 | 3.7 | 14 |
| GO Biological Processes | induction of positive chemotaxis | -2.5 | 3.7 | 3.7 | 14 |
| GO Biological Processes | regulation of excitatory synapse assembly | -2.5 | 3.7 | 3.7 | 14 |
| GO Biological Processes | regulation of vasculogenesis | -2.5 | 3.7 | 3.7 | 14 |
| GO Biological Processes | positive regulation of chemokine secretion | -2.5 | 3.7 | 3.7 | 14 |
| GO Biological Processes | lipoxygenase pathway | -2.5 | 3.7 | 3.7 | 14 |
| GO Biological Processes | heart field specification | -2.5 | 3.7 | 3.7 | 14 |
| GO Biological Processes | regulation of ketone biosynthetic process | -2.5 | 3.7 | 3.7 | 14 |
| GO Biological Processes | negative regulation of bone remodeling | -2.5 | 3.7 | 3.7 | 14 |
| GO Biological Processes | regulation of mitotic spindle checkpoint | -2.5 | 3.7 | 3.7 | 14 |
| GO Biological Processes | cardiac left ventricle morphogenesis | -2.5 | 3.7 | 3.7 | 14 |
| GO Biological Processes | regulation of mitotic cell cycle spindle assembly checkpoint | -2.5 | 3.7 | 3.7 | 14 |
| GO Biological Processes | spindle midzone assembly | -2.5 | 4.3 | 3.8 | 10 |
| GO Biological Processes | spindle elongation | -2.5 | 4.3 | 3.8 | 10 |
| GO Biological Processes | lung secretory cell differentiation | -2.5 | 4.3 | 3.8 | 10 |
| GO Biological Processes | retrograde trans-synaptic signaling | -2.5 | 4.3 | 3.8 | 10 |
| GO Biological Processes | negative regulation of erythrocyte differentiation | -2.5 | 4.3 | 3.8 | 10 |
| GO Biological Processes | positive regulation of protein kinase A signaling | -2.5 | 4.3 | 3.8 | 10 |
| GO Biological Processes | cardiac ventricle formation | -2.5 | 4.3 | 3.8 | 10 |
| GO Biological Processes | positive regulation of prostaglandin secretion | -2.5 | 4.3 | 3.8 | 10 |
| GO Biological Processes | vascular smooth muscle contraction | -2.5 | 3 | 3.5 | 23 |
| GO Biological Processes | renal system process involved in regulation of systemic arterial blood pressure | -2.5 | 3 | 3.5 | 23 |
| GO Biological Processes | multicellular organism growth | -2.5 | 1.7 | 3 | 148 |
| GO Biological Processes | cellular response to alkaloid | -2.5 | 2.6 | 3.4 | 33 |
| GO Biological Processes | vascular endothelial growth factor production | -2.5 | 2.6 | 3.4 | 33 |

| | | | | | |
|---|---|---|---|---|---|
| GO Biological Processes | epiboly | -2.5 | 2.6 | 3.4 | 33 |
| GO Biological Processes | cellular response to lipopolysaccharide | -2.5 | 1.6 | 3 | 202 |
| GO Biological Processes | negative regulation of neuron death | -2.5 | 1.6 | 3 | 202 |
| GO Biological Processes | Rho protein signal transduction | -2.5 | 1.6 | 3 | 202 |
| GO Biological Processes | regulation of the force of heart contraction | -2.5 | 2.8 | 3.4 | 28 |
| GO Biological Processes | outflow tract septum morphogenesis | -2.5 | 2.8 | 3.4 | 28 |
| GO Biological Processes | aromatic amino acid family catabolic process | -2.5 | 2.8 | 3.4 | 28 |
| GO Biological Processes | protein kinase C signaling | -2.5 | 2.8 | 3.4 | 28 |
| GO Biological Processes | cellular response to fatty acid | -2.5 | 2.2 | 3.2 | 55 |
| GO Biological Processes | positive regulation of cold-induced thermogenesis | -2.5 | 1.9 | 3.1 | 97 |
| GO Biological Processes | small GTPase mediated signal transduction | -2.5 | 1.3 | 2.9 | 556 |
| GO Biological Processes | regulation of protein binding | -2.5 | 1.6 | 3 | 216 |
| GO Biological Processes | negative regulation of blood vessel morphogenesis | -2.5 | 1.6 | 3 | 182 |
| GO Biological Processes | central nervous system neuron differentiation | -2.5 | 1.6 | 3 | 182 |
| GO Biological Processes | positive regulation of cell projection organization | -2.5 | 1.4 | 2.9 | 372 |
| GO Biological Processes | mononuclear cell proliferation | -2.5 | 1.5 | 2.9 | 272 |
| GO Biological Processes | midbrain development | -2.5 | 1.9 | 3.1 | 91 |
| GO Biological Processes | regulation of phagocytosis | -2.5 | 1.9 | 3.1 | 91 |
| GO Biological Processes | pri-miRNA transcription by RNA polymerase II | -2.5 | 2.3 | 3.2 | 44 |
| GO Biological Processes | disruption of cells of other organism | -2.5 | 2.1 | 3.2 | 61 |
| GO Biological Processes | positive regulation of DNA binding | -2.5 | 2.1 | 3.2 | 61 |
| GO Biological Processes | negative regulation of cell cycle G2/M phase transition | -2.5 | 2.1 | 3.2 | 61 |
| GO Biological Processes | killing of cells of other organism | -2.5 | 2.1 | 3.2 | 61 |
| GO Biological Processes | columnar/cuboidal epithelial cell differentiation | -2.5 | 1.8 | 3.1 | 110 |
| GO Biological Processes | regulation of synaptic vesicle transport | -2.5 | 1.9 | 3.1 | 85 |
| GO Biological Processes | cardiac muscle cell proliferation | -2.4 | 2.1 | 3.1 | 67 |
| GO Biological Processes | negative regulation of cytokine secretion | -2.4 | 2.1 | 3.1 | 67 |
| GO Biological Processes | protein heterooligomerization | -2.4 | 1.7 | 3 | 136 |
| GO Biological Processes | regulation of phosphatidylinositol 3-kinase signaling | -2.4 | 1.8 | 3 | 123 |
| GO Biological Processes | cell-cell junction assembly | -2.4 | 1.8 | 3 | 123 |
| GO Biological Processes | negative regulation of defense response | -2.4 | 1.5 | 2.9 | 217 |
| GO Biological Processes | regulation of cellular component size | -2.4 | 1.4 | 2.9 | 366 |
| GO Biological Processes | regulation of potassium ion transport | -2.4 | 1.8 | 3 | 104 |
| GO Biological Processes | alpha-amino acid metabolic process | -2.4 | 1.5 | 2.9 | 224 |
| GO Biological Processes | regulation of endothelial cell proliferation | -2.4 | 1.6 | 3 | 183 |
| GO Biological Processes | cellular response to fibroblast growth factor stimulus | -2.4 | 1.7 | 3 | 143 |
| GO Biological Processes | neuron projection extension | -2.4 | 1.6 | 3 | 163 |
| GO Biological Processes | positive regulation of ion transmembrane transporter activity | -2.4 | 1.8 | 3 | 98 |
| GO Biological Processes | dopamine metabolic process | -2.4 | 2.4 | 3.2 | 39 |
| GO Biological Processes | regulation of extracellular matrix organization | -2.4 | 2.4 | 3.2 | 39 |
| GO Biological Processes | positive regulation of cardiac muscle cell proliferation | -2.4 | 2.4 | 3.2 | 39 |
| GO Biological Processes | response to fibroblast growth factor | -2.4 | 1.7 | 3 | 150 |
| GO Biological Processes | lipid biosynthetic process | -2.4 | 1.3 | 2.8 | 718 |
| GO Biological Processes | negative regulation of myeloid cell differentiation | -2.4 | 1.9 | 3 | 92 |
| GO Biological Processes | regulation of interleukin-2 biosynthetic process | -2.4 | 3.2 | 3.4 | 19 |

| | | | | | |
|---|---|---|---|---|---|
| GO Biological Processes | camera-type eye photoreceptor cell differentiation | -2.4 | 3.2 | 3.4 | 19 |
| GO Biological Processes | negative regulation of tissue remodeling | -2.4 | 3.2 | 3.4 | 19 |
| GO Biological Processes | pulmonary valve development | -2.4 | 3.2 | 3.4 | 19 |
| GO Biological Processes | cellular response to biotic stimulus | -2.4 | 1.5 | 2.9 | 232 |
| GO Biological Processes | morphogenesis of an epithelial sheet | -2.4 | 2.2 | 3.1 | 56 |
| GO Biological Processes | embryonic epithelial tube formation | -2.4 | 1.7 | 3 | 124 |
| GO Biological Processes | dicarboxylic acid transport | -2.4 | 1.9 | 3 | 86 |
| GO Biological Processes | embryonic eye morphogenesis | -2.4 | 2.5 | 3.2 | 34 |
| GO Biological Processes | neural retina development | -2.4 | 2.1 | 3.1 | 62 |
| GO Biological Processes | regulation of nitric oxide biosynthetic process | -2.4 | 2.1 | 3.1 | 62 |
| GO Biological Processes | regulation of pathway-restricted SMAD protein phosphorylation | -2.4 | 2.1 | 3.1 | 62 |
| GO Biological Processes | positive regulation of lipid localization | -2.4 | 1.9 | 3 | 80 |
| GO Biological Processes | regulation of synaptic vesicle exocytosis | -2.4 | 1.9 | 3 | 80 |
| GO Biological Processes | regulation of release of sequestered calcium ion into cytosol | -2.4 | 1.9 | 3 | 80 |
| GO Biological Processes | phosphatidylcholine metabolic process | -2.4 | 1.9 | 3 | 80 |
| GO Biological Processes | negative regulation of cellular response to growth factor stimulus | -2.4 | 1.6 | 2.9 | 164 |
| GO Biological Processes | cardiac muscle cell action potential | -2.4 | 2 | 3.1 | 74 |
| GO Biological Processes | endoderm development | -2.4 | 2 | 3.1 | 74 |
| GO Biological Processes | cardiac muscle cell contraction | -2.4 | 2 | 3.1 | 68 |
| GO Biological Processes | positive regulation of response to wounding | -2.4 | 2 | 3.1 | 68 |
| GO Biological Processes | regulation of myeloid leukocyte differentiation | -2.4 | 1.8 | 3 | 118 |
| GO Biological Processes | epidermis morphogenesis | -2.4 | 2.7 | 3.3 | 29 |
| GO Biological Processes | epithelial tube branching involved in lung morphogenesis | -2.4 | 2.7 | 3.3 | 29 |
| GO Biological Processes | regulation of transcription involved in G1/S transition of mitotic cell cycle | -2.4 | 2.7 | 3.3 | 29 |
| GO Biological Processes | cellular response to angiotensin | -2.4 | 2.9 | 3.3 | 24 |
| GO Biological Processes | regulation of homotypic cell-cell adhesion | -2.4 | 2.9 | 3.3 | 24 |
| GO Biological Processes | positive regulation of endothelial cell differentiation | -2.4 | 2.9 | 3.3 | 24 |
| GO Biological Processes | plasminogen activation | -2.4 | 2.9 | 3.3 | 24 |
| GO Biological Processes | protein localization to plasma membrane | -2.4 | 1.5 | 2.9 | 254 |
| GO Biological Processes | male sex differentiation | -2.3 | 1.6 | 2.9 | 158 |
| GO Biological Processes | positive regulation of endothelial cell proliferation | -2.3 | 1.8 | 3 | 112 |
| GO Biological Processes | regulation of protein localization to plasma membrane | -2.3 | 1.9 | 3 | 93 |
| GO Biological Processes | regulation of anion transport | -2.3 | 1.9 | 3 | 93 |
| GO Biological Processes | regulation of neuronal synaptic plasticity | -2.3 | 2.2 | 3.1 | 51 |
| GO Biological Processes | positive regulation of cell division | -2.3 | 1.9 | 3 | 87 |
| GO Biological Processes | fatty acid metabolic process | -2.3 | 1.4 | 2.8 | 370 |
| GO Biological Processes | vitamin metabolic process | -2.3 | 1.7 | 2.9 | 132 |
| GO Biological Processes | cardiac muscle cell differentiation | -2.3 | 1.7 | 2.9 | 132 |
| GO Biological Processes | mitotic spindle midzone assembly | -2.3 | 4.9 | 3.8 | 7 |
| GO Biological Processes | fibroblast growth factor production | -2.3 | 4.9 | 3.8 | 7 |
| GO Biological Processes | positive regulation of extracellular matrix disassembly | -2.3 | 4.9 | 3.8 | 7 |
| GO Biological Processes | pericyte cell differentiation | -2.3 | 4.9 | 3.8 | 7 |
| GO Biological Processes | regulation of fibroblast growth factor production | -2.3 | 4.9 | 3.8 | 7 |

| | | | | | |
|---|---|---|---|---|---|
| GO Biological Processes | negative regulation of cytokinesis | -2.3 | 4.9 | 3.8 | 7 |
| GO Biological Processes | regulation of aldosterone metabolic process | -2.3 | 4.9 | 3.8 | 7 |
| GO Biological Processes | regulation of penile erection | -2.3 | 4.9 | 3.8 | 7 |
| GO Biological Processes | cellular response to hyperoxia | -2.3 | 4.9 | 3.8 | 7 |
| GO Biological Processes | negative regulation of gastrulation | -2.3 | 4.9 | 3.8 | 7 |
| GO Biological Processes | regulation of aldosterone biosynthetic process | -2.3 | 4.9 | 3.8 | 7 |
| GO Biological Processes | positive regulation of fever generation | -2.3 | 4.9 | 3.8 | 7 |
| GO Biological Processes | glycerol transport | -2.3 | 4.9 | 3.8 | 7 |
| GO Biological Processes | adenylate cyclase-inhibiting G protein-coupled acetylcholine receptor signaling pathway | -2.3 | 4.9 | 3.8 | 7 |
| GO Biological Processes | response to cGMP | -2.3 | 4.9 | 3.8 | 7 |
| GO Biological Processes | regulation of extracellular matrix constituent secretion | -2.3 | 4.9 | 3.8 | 7 |
| GO Biological Processes | equilibrioception | -2.3 | 4.9 | 3.8 | 7 |
| GO Biological Processes | luteolysis | -2.3 | 4.9 | 3.8 | 7 |
| GO Biological Processes | positive regulation of embryonic development | -2.3 | 2.4 | 3.1 | 40 |
| GO Biological Processes | regulation of sensory perception of pain | -2.3 | 2.4 | 3.1 | 40 |
| GO Biological Processes | dopamine secretion | -2.3 | 2.4 | 3.1 | 40 |
| GO Biological Processes | regulation of dopamine secretion | -2.3 | 2.4 | 3.1 | 40 |
| GO Biological Processes | negative regulation of actin filament polymerization | -2.3 | 2.1 | 3.1 | 57 |
| GO Biological Processes | cellular response to external stimulus | -2.3 | 1.4 | 2.8 | 334 |
| GO Biological Processes | heart trabecula formation | -2.3 | 3.4 | 3.4 | 15 |
| GO Biological Processes | regulation of protein kinase C signaling | -2.3 | 3.4 | 3.4 | 15 |
| GO Biological Processes | response to caffeine | -2.3 | 3.4 | 3.4 | 15 |
| GO Biological Processes | dopamine uptake | -2.3 | 3.4 | 3.4 | 15 |
| GO Biological Processes | hormone catabolic process | -2.3 | 3.4 | 3.4 | 15 |
| GO Biological Processes | response to diuretic | -2.3 | 3.4 | 3.4 | 15 |
| GO Biological Processes | regulation of cilium movement | -2.3 | 3.4 | 3.4 | 15 |
| GO Biological Processes | regulation of extracellular matrix disassembly | -2.3 | 3.4 | 3.4 | 15 |
| GO Biological Processes | venous blood vessel development | -2.3 | 3.4 | 3.4 | 15 |
| GO Biological Processes | negative regulation of catecholamine secretion | -2.3 | 3.4 | 3.4 | 15 |
| GO Biological Processes | phospholipid transport | -2.3 | 2 | 3 | 75 |
| GO Biological Processes | photoreceptor cell differentiation | -2.3 | 2.1 | 3 | 63 |
| GO Biological Processes | sister chromatid cohesion | -2.3 | 2.1 | 3 | 63 |
| GO Biological Processes | positive regulation of small GTPase mediated signal transduction | -2.3 | 2 | 3 | 69 |
| GO Biological Processes | ovulation cycle | -2.3 | 2 | 3 | 69 |
| GO Biological Processes | positive regulation of gliogenesis | -2.3 | 2 | 3 | 69 |
| GO Biological Processes | multi-organism behavior | -2.3 | 2 | 3 | 69 |
| GO Biological Processes | regulation of interleukin-1 beta production | -2.3 | 2 | 3 | 69 |
| GO Biological Processes | lymphocyte proliferation | -2.3 | 1.5 | 2.8 | 270 |
| GO Biological Processes | positive regulation of cytoskeleton organization | -2.3 | 1.5 | 2.8 | 221 |
| GO Biological Processes | phosphatidylinositol 3-kinase signaling | -2.3 | 1.7 | 2.9 | 146 |
| GO Biological Processes | epidermis development | -2.3 | 1.3 | 2.7 | 460 |
| GO Biological Processes | eye photoreceptor cell differentiation | -2.3 | 2.2 | 3.1 | 46 |
| GO Biological Processes | chromatin remodeling at centromere | -2.3 | 2.2 | 3.1 | 46 |
| GO Biological Processes | aorta morphogenesis | -2.3 | 2.5 | 3.1 | 35 |
| GO Biological Processes | intestinal absorption | -2.3 | 2.5 | 3.1 | 35 |

| | | | | | |
|---|---|---|---|---|---|
| GO Biological Processes | regulation of neuroinflammatory response | -2.3 | 2.5 | 3.1 | 35 |
| GO Biological Processes | negative regulation of angiogenesis | -2.3 | 1.6 | 2.8 | 180 |
| GO Biological Processes | calcium ion regulated exocytosis | -2.3 | 1.6 | 2.8 | 153 |
| GO Biological Processes | substrate adhesion-dependent cell spreading | -2.3 | 1.9 | 2.9 | 88 |
| GO Biological Processes | regulation of epithelial to mesenchymal transition | -2.3 | 1.9 | 2.9 | 88 |
| GO Biological Processes | regulation of type B pancreatic cell proliferation | -2.3 | 3.9 | 3.5 | 11 |
| GO Biological Processes | gap junction assembly | -2.3 | 3.9 | 3.5 | 11 |
| GO Biological Processes | regulation of cholesterol esterification | -2.3 | 3.9 | 3.5 | 11 |
| GO Biological Processes | regulation of cardiac epithelial to mesenchymal transition | -2.3 | 3.9 | 3.5 | 11 |
| GO Biological Processes | very-low-density lipoprotein particle remodeling | -2.3 | 3.9 | 3.5 | 11 |
| GO Biological Processes | tonic smooth muscle contraction | -2.3 | 3.9 | 3.5 | 11 |
| GO Biological Processes | retinal cone cell differentiation | -2.3 | 3.9 | 3.5 | 11 |
| GO Biological Processes | cardiac vascular smooth muscle cell differentiation | -2.3 | 3.9 | 3.5 | 11 |
| GO Biological Processes | negative regulation of cell fate commitment | -2.3 | 3.9 | 3.5 | 11 |
| GO Biological Processes | regulation of prostaglandin secretion | -2.3 | 3.9 | 3.5 | 11 |
| GO Biological Processes | retinal cone cell development | -2.3 | 3.9 | 3.5 | 11 |
| GO Biological Processes | bleb assembly | -2.3 | 3.9 | 3.5 | 11 |
| GO Biological Processes | cell proliferation in hindbrain | -2.3 | 3.9 | 3.5 | 11 |
| GO Biological Processes | establishment of blood-brain barrier | -2.3 | 3.9 | 3.5 | 11 |
| GO Biological Processes | mitral valve development | -2.3 | 3.9 | 3.5 | 11 |
| GO Biological Processes | histone-serine phosphorylation | -2.3 | 3.9 | 3.5 | 11 |
| GO Biological Processes | negative regulation of vasculature development | -2.3 | 1.6 | 2.8 | 194 |
| GO Biological Processes | regulation of skeletal muscle tissue development | -2.3 | 2.2 | 3 | 52 |
| GO Biological Processes | arachidonic acid metabolic process | -2.3 | 2.2 | 3 | 52 |
| GO Biological Processes | response to extracellular stimulus | -2.3 | 1.3 | 2.7 | 521 |
| GO Biological Processes | negative regulation of Wnt signaling pathway | -2.3 | 1.6 | 2.8 | 167 |
| GO Biological Processes | regulation of heart rate | -2.3 | 1.8 | 2.9 | 101 |
| GO Biological Processes | regulation of fatty acid oxidation | -2.3 | 2.6 | 3.1 | 30 |
| GO Biological Processes | positive regulation of protein maturation | -2.3 | 2.6 | 3.1 | 30 |
| GO Biological Processes | regulation of catecholamine metabolic process | -2.3 | 3 | 3.3 | 20 |
| GO Biological Processes | atrial cardiac muscle cell to AV node cell signaling | -2.3 | 3 | 3.3 | 20 |
| GO Biological Processes | maintenance of gastrointestinal epithelium | -2.3 | 3 | 3.3 | 20 |
| GO Biological Processes | copulation | -2.3 | 3 | 3.3 | 20 |
| GO Biological Processes | positive regulation of extracellular matrix organization | -2.3 | 3 | 3.3 | 20 |
| GO Biological Processes | regulation of dopamine metabolic process | -2.3 | 3 | 3.3 | 20 |
| GO Biological Processes | intestinal epithelial cell differentiation | -2.3 | 3 | 3.3 | 20 |
| GO Biological Processes | atrial cardiac muscle cell to AV node cell communication | -2.3 | 3 | 3.3 | 20 |
| GO Biological Processes | atrial cardiac muscle cell action potential | -2.3 | 3 | 3.3 | 20 |
| GO Biological Processes | mesoderm morphogenesis | -2.3 | 1.9 | 2.9 | 76 |
| GO Biological Processes | negative regulation of T cell proliferation | -2.2 | 2.1 | 3 | 58 |
| GO Biological Processes | positive regulation of blood vessel diameter | -2.2 | 2.1 | 3 | 58 |
| GO Biological Processes | positive regulation of organ growth | -2.2 | 2 | 3 | 64 |
| GO Biological Processes | endothelial cell development | -2.2 | 2 | 3 | 64 |
| GO Biological Processes | catagen | -2.2 | 6.5 | 4 | 4 |
| GO Biological Processes | positive regulation of interleukin-1 biosynthetic process | -2.2 | 6.5 | 4 | 4 |

| | | | | | |
|---|---|---|---|---|---|
| GO Biological Processes | response to bile acid | -2.2 | 6.5 | 4 | 4 |
| GO Biological Processes | endocardial cushion fusion | -2.2 | 6.5 | 4 | 4 |
| GO Biological Processes | regulation of antibacterial peptide production | -2.2 | 6.5 | 4 | 4 |
| GO Biological Processes | positive regulation of interleukin-1 beta biosynthetic process | -2.2 | 6.5 | 4 | 4 |
| GO Biological Processes | negative regulation of cell proliferation involved in contact inhibition | -2.2 | 6.5 | 4 | 4 |
| GO Biological Processes | positive regulation of transcription from RNA polymerase II promoter involved in myocardial precurso | -2.2 | 6.5 | 4 | 4 |
| GO Biological Processes | regulation of Rho-dependent protein serine/threonine kinase activity | -2.2 | 6.5 | 4 | 4 |
| GO Biological Processes | regulation of interleukin-1 beta biosynthetic process | -2.2 | 6.5 | 4 | 4 |
| GO Biological Processes | lung goblet cell differentiation | -2.2 | 6.5 | 4 | 4 |
| GO Biological Processes | adrenomedullin receptor signaling pathway | -2.2 | 6.5 | 4 | 4 |
| GO Biological Processes | regulation of interleukin-1 biosynthetic process | -2.2 | 6.5 | 4 | 4 |
| GO Biological Processes | urinary bladder development | -2.2 | 6.5 | 4 | 4 |
| GO Biological Processes | regulation of Notch signaling pathway involved in heart induction | -2.2 | 6.5 | 4 | 4 |
| GO Biological Processes | regulation of timing of catagen | -2.2 | 6.5 | 4 | 4 |
| GO Biological Processes | positive regulation of antimicrobial humoral response | -2.2 | 6.5 | 4 | 4 |
| GO Biological Processes | positive regulation of Notch signaling pathway involved in heart induction | -2.2 | 6.5 | 4 | 4 |
| GO Biological Processes | renin-angiotensin regulation of aldosterone production | -2.2 | 6.5 | 4 | 4 |
| GO Biological Processes | regulation of integrin biosynthetic process | -2.2 | 6.5 | 4 | 4 |
| GO Biological Processes | negative regulation of Wnt signaling pathway involved in heart development | -2.2 | 6.5 | 4 | 4 |
| GO Biological Processes | positive regulation of cell adhesion molecule production | -2.2 | 6.5 | 4 | 4 |
| GO Biological Processes | Notch signaling pathway involved in heart induction | -2.2 | 6.5 | 4 | 4 |
| GO Biological Processes | regulation of antimicrobial peptide production | -2.2 | 6.5 | 4 | 4 |
| GO Biological Processes | glomerular mesangial cell development | -2.2 | 6.5 | 4 | 4 |
| GO Biological Processes | regulation of cytolysis in other organism | -2.2 | 6.5 | 4 | 4 |
| GO Biological Processes | myotome development | -2.2 | 6.5 | 4 | 4 |
| GO Biological Processes | response to cortisol | -2.2 | 6.5 | 4 | 4 |
| GO Biological Processes | negative regulation of hepatocyte proliferation | -2.2 | 6.5 | 4 | 4 |
| GO Biological Processes | response to ozone | -2.2 | 6.5 | 4 | 4 |
| GO Biological Processes | regulation of lipopolysaccharide-mediated signaling pathway | -2.2 | 2.8 | 3.2 | 25 |
| GO Biological Processes | hair follicle morphogenesis | -2.2 | 2.8 | 3.2 | 25 |
| GO Biological Processes | meiotic chromosome separation | -2.2 | 2.8 | 3.2 | 25 |
| GO Biological Processes | eosinophil migration | -2.2 | 2.8 | 3.2 | 25 |
| GO Biological Processes | bile acid and bile salt transport | -2.2 | 2.8 | 3.2 | 25 |
| GO Biological Processes | regulation of reactive oxygen species biosynthetic process | -2.2 | 1.8 | 2.9 | 95 |
| GO Biological Processes | positive regulation of cardiocyte differentiation | -2.2 | 2.3 | 3 | 41 |
| GO Biological Processes | oligodendrocyte development | -2.2 | 2.3 | 3 | 41 |
| GO Biological Processes | regulation of sensory perception | -2.2 | 2.3 | 3 | 41 |
| GO Biological Processes | cellular response to molecule of bacterial origin | -2.2 | 1.5 | 2.8 | 209 |
| GO Biological Processes | regulation of biomineral tissue development | -2.2 | 1.8 | 2.9 | 89 |
| GO Biological Processes | negative regulation of transporter activity | -2.2 | 1.8 | 2.9 | 89 |

| | | | | | |
|---|---|---|---|---|---|
| GO Biological Processes | regulation of interleukin-6 production | -2.2 | 1.7 | 2.8 | 128 |
| GO Biological Processes | detoxification | -2.2 | 1.7 | 2.8 | 128 |
| GO Biological Processes | peptidyl-tyrosine dephosphorylation | -2.2 | 1.8 | 2.8 | 102 |
| GO Biological Processes | negative regulation of epithelial cell apoptotic process | -2.2 | 2.2 | 3 | 47 |
| GO Biological Processes | cochlea development | -2.2 | 2.2 | 3 | 47 |
| GO Biological Processes | glandular epithelial cell differentiation | -2.2 | 2.2 | 3 | 47 |
| GO Biological Processes | prostate gland development | -2.2 | 2.2 | 3 | 47 |
| GO Biological Processes | intermediate filament cytoskeleton organization | -2.2 | 2.2 | 3 | 47 |
| GO Biological Processes | ovulation cycle process | -2.2 | 2.2 | 3 | 47 |
| GO Biological Processes | defense response to bacterium | -2.2 | 1.4 | 2.7 | 324 |
| GO Biological Processes | myeloid leukocyte differentiation | -2.2 | 1.5 | 2.7 | 203 |
| GO Biological Processes | cellular response to mechanical stimulus | -2.2 | 1.9 | 2.9 | 77 |
| GO Biological Processes | associative learning | -2.2 | 1.9 | 2.9 | 77 |
| GO Biological Processes | cellular carbohydrate metabolic process | -2.2 | 1.4 | 2.7 | 281 |
| GO Biological Processes | macrophage derived foam cell differentiation | -2.2 | 2.4 | 3 | 36 |
| GO Biological Processes | regulation of cell fate commitment | -2.2 | 2.4 | 3 | 36 |
| GO Biological Processes | negative regulation of endothelial cell apoptotic process | -2.2 | 2.4 | 3 | 36 |
| GO Biological Processes | semi-lunar valve development | -2.2 | 2.4 | 3 | 36 |
| GO Biological Processes | membrane biogenesis | -2.2 | 2.4 | 3 | 36 |
| GO Biological Processes | foam cell differentiation | -2.2 | 2.4 | 3 | 36 |
| GO Biological Processes | regulation of interferon-gamma production | -2.2 | 1.8 | 2.8 | 96 |
| GO Biological Processes | body fluid secretion | -2.2 | 1.8 | 2.8 | 96 |
| GO Biological Processes | positive regulation of leukocyte migration | -2.2 | 1.7 | 2.8 | 122 |
| GO Biological Processes | negative regulation of cation transmembrane transport | -2.2 | 1.8 | 2.8 | 90 |
| GO Biological Processes | cellular modified amino acid metabolic process | -2.2 | 1.5 | 2.7 | 197 |
| GO Biological Processes | neuron cell-cell adhesion | -2.2 | 3.2 | 3.2 | 16 |
| GO Biological Processes | thyroid hormone generation | -2.2 | 3.2 | 3.2 | 16 |
| GO Biological Processes | complement activation, alternative pathway | -2.2 | 3.2 | 3.2 | 16 |
| GO Biological Processes | killing of cells in other organism involved in symbiotic interaction | -2.2 | 3.2 | 3.2 | 16 |
| GO Biological Processes | hyaluronan catabolic process | -2.2 | 3.2 | 3.2 | 16 |
| GO Biological Processes | metanephric glomerulus development | -2.2 | 3.2 | 3.2 | 16 |
| GO Biological Processes | dendrite self-avoidance | -2.2 | 3.2 | 3.2 | 16 |
| GO Biological Processes | regulation of glutamate secretion | -2.2 | 3.2 | 3.2 | 16 |
| GO Biological Processes | negative regulation of meiotic cell cycle | -2.2 | 3.2 | 3.2 | 16 |
| GO Biological Processes | regulation of norepinephrine secretion | -2.2 | 3.2 | 3.2 | 16 |
| GO Biological Processes | regulation of chemokine secretion | -2.2 | 3.2 | 3.2 | 16 |
| GO Biological Processes | catecholamine uptake | -2.2 | 3.2 | 3.2 | 16 |
| GO Biological Processes | sodium-independent organic anion transport | -2.2 | 3.2 | 3.2 | 16 |
| GO Biological Processes | cerebral cortex development | -2.2 | 1.7 | 2.8 | 116 |
| GO Biological Processes | positive regulation of cytosolic calcium ion concentration involved in phospholipase C-activating G | -2.2 | 2.5 | 3 | 31 |
| GO Biological Processes | positive regulation of smoothened signaling pathway | -2.2 | 2.5 | 3 | 31 |
| GO Biological Processes | axis elongation | -2.2 | 2.5 | 3 | 31 |
| GO Biological Processes | mitotic G2/M transition checkpoint | -2.2 | 2.5 | 3 | 31 |
| GO Biological Processes | arachidonic acid secretion | -2.2 | 2.5 | 3 | 31 |
| GO Biological Processes | arachidonate transport | -2.2 | 2.5 | 3 | 31 |

| | | | | | |
|---|---|---|---|---|---|
| GO Biological Processes | alcohol metabolic process | -2.2 | 1.4 | 2.6 | 355 |
| GO Biological Processes | interleukin-6 production | -2.2 | 1.6 | 2.7 | 136 |
| GO Biological Processes | fat-soluble vitamin metabolic process | -2.2 | 2.3 | 3 | 42 |
| GO Biological Processes | cell-cell adhesion mediated by cadherin | -2.2 | 2.3 | 3 | 42 |
| GO Biological Processes | drug transmembrane transport | -2.1 | 1.8 | 2.8 | 84 |
| GO Biological Processes | response to nutrient levels | -2.1 | 1.3 | 2.6 | 489 |
| GO Biological Processes | negative regulation of protein transport | -2.1 | 1.5 | 2.7 | 184 |
| GO Biological Processes | negative regulation of ion transmembrane transporter activity | -2.1 | 1.9 | 2.8 | 78 |
| GO Biological Processes | regulation of actin filament bundle assembly | -2.1 | 1.8 | 2.8 | 97 |
| GO Biological Processes | protein O-linked glycosylation | -2.1 | 1.7 | 2.8 | 110 |
| GO Biological Processes | regulation of leukocyte chemotaxis | -2.1 | 1.7 | 2.8 | 110 |
| GO Biological Processes | positive regulation of excitatory postsynaptic potential | -2.1 | 2.7 | 3.1 | 26 |
| GO Biological Processes | cell proliferation in forebrain | -2.1 | 2.7 | 3.1 | 26 |
| GO Biological Processes | regulation of hormone biosynthetic process | -2.1 | 2.7 | 3.1 | 26 |
| GO Biological Processes | positive regulation of mesenchymal cell proliferation | -2.1 | 2.7 | 3.1 | 26 |
| GO Biological Processes | regulation of microtubule-based movement | -2.1 | 2.7 | 3.1 | 26 |
| GO Biological Processes | positive regulation of blood coagulation | -2.1 | 2.7 | 3.1 | 26 |
| GO Biological Processes | regulation of long-term neuronal synaptic plasticity | -2.1 | 2.7 | 3.1 | 26 |
| GO Biological Processes | retinoic acid metabolic process | -2.1 | 2.7 | 3.1 | 26 |
| GO Biological Processes | positive regulation of hemostasis | -2.1 | 2.7 | 3.1 | 26 |
| GO Biological Processes | cell differentiation involved in metanephros development | -2.1 | 2.7 | 3.1 | 26 |
| GO Biological Processes | neuron death | -2.1 | 1.4 | 2.6 | 334 |
| GO Biological Processes | positive regulation of muscle contraction | -2.1 | 2.2 | 2.9 | 48 |
| GO Biological Processes | regulation of renal sodium excretion | -2.1 | 2.9 | 3.1 | 21 |
| GO Biological Processes | membrane raft organization | -2.1 | 2.9 | 3.1 | 21 |
| GO Biological Processes | norepinephrine transport | -2.1 | 2.9 | 3.1 | 21 |
| GO Biological Processes | peroxisome proliferator activated receptor signaling pathway | -2.1 | 2.9 | 3.1 | 21 |
| GO Biological Processes | kinetochore organization | -2.1 | 2.9 | 3.1 | 21 |
| GO Biological Processes | antibiotic biosynthetic process | -2.1 | 2.9 | 3.1 | 21 |
| GO Biological Processes | positive regulation of striated muscle cell differentiation | -2.1 | 1.9 | 2.8 | 72 |
| GO Biological Processes | regulation of interleukin-2 production | -2.1 | 2.1 | 2.9 | 54 |
| GO Biological Processes | dendritic spine morphogenesis | -2.1 | 2.1 | 2.9 | 54 |
| GO Biological Processes | negative regulation of small GTPase mediated signal transduction | -2.1 | 2.1 | 2.9 | 54 |
| GO Biological Processes | positive regulation of phagocytosis | -2.1 | 2 | 2.8 | 66 |
| GO Biological Processes | cellular response to ammonium ion | -2.1 | 2 | 2.8 | 66 |
| GO Biological Processes | chromatin assembly | -2.1 | 1.6 | 2.7 | 164 |
| GO Biological Processes | positive regulation of cell cycle G1/S phase transition | -2.1 | 2 | 2.8 | 60 |
| GO Biological Processes | metaphase plate congression | -2.1 | 2 | 2.8 | 60 |
| GO Biological Processes | positive regulation of lipid transport | -2.1 | 2 | 2.8 | 60 |
| GO Biological Processes | regulation of striated muscle cell differentiation | -2.1 | 1.7 | 2.7 | 117 |
| GO Biological Processes | unsaturated fatty acid metabolic process | -2.1 | 1.7 | 2.7 | 104 |
| GO Biological Processes | regulation of multi-organism process | -2.1 | 1.4 | 2.6 | 379 |
| GO Biological Processes | prostanoid metabolic process | -2.1 | 2.3 | 2.9 | 37 |
| GO Biological Processes | prostaglandin metabolic process | -2.1 | 2.3 | 2.9 | 37 |

| | | | | | |
|---|---|---|---|---|---|
| GO Biological Processes | regulation of lipid metabolic process | -2.1 | 1.4 | 2.6 | 394 |
| GO Biological Processes | glucose metabolic process | -2.1 | 1.5 | 2.6 | 206 |
| GO Biological Processes | hormone biosynthetic process | -2.1 | 1.8 | 2.8 | 85 |
| GO Biological Processes | regulation of DNA binding | -2.1 | 1.7 | 2.7 | 124 |
| GO Biological Processes | regulation of protein localization to cell periphery | -2.1 | 1.7 | 2.7 | 111 |
| GO Biological Processes | aminoglycan biosynthetic process | -2.1 | 1.7 | 2.7 | 111 |
| GO Biological Processes | ectodermal placode morphogenesis | -2.1 | 3.6 | 3.3 | 12 |
| GO Biological Processes | saliva secretion | -2.1 | 3.6 | 3.3 | 12 |
| GO Biological Processes | killing by host of symbiont cells | -2.1 | 3.6 | 3.3 | 12 |
| GO Biological Processes | positive regulation of killing of cells of other organism | -2.1 | 3.6 | 3.3 | 12 |
| GO Biological Processes | L-glutamate import across plasma membrane | -2.1 | 3.6 | 3.3 | 12 |
| GO Biological Processes | triglyceride-rich lipoprotein particle remodeling | -2.1 | 3.6 | 3.3 | 12 |
| GO Biological Processes | ectodermal placode formation | -2.1 | 3.6 | 3.3 | 12 |
| GO Biological Processes | negative regulation of vascular permeability | -2.1 | 3.6 | 3.3 | 12 |
| GO Biological Processes | regulation of postsynaptic density assembly | -2.1 | 3.6 | 3.3 | 12 |
| GO Biological Processes | substrate-independent telencephalic tangential interneuron migration | -2.1 | 3.6 | 3.3 | 12 |
| GO Biological Processes | MHC class II biosynthetic process | -2.1 | 3.6 | 3.3 | 12 |
| GO Biological Processes | epithelial cell differentiation involved in prostate gland development | -2.1 | 3.6 | 3.3 | 12 |
| GO Biological Processes | substrate-independent telencephalic tangential migration | -2.1 | 3.6 | 3.3 | 12 |
| GO Biological Processes | negative regulation of neuron migration | -2.1 | 3.6 | 3.3 | 12 |
| GO Biological Processes | regulation of guanylate cyclase activity | -2.1 | 3.6 | 3.3 | 12 |
| GO Biological Processes | regulation of phospholipase A2 activity | -2.1 | 3.6 | 3.3 | 12 |
| GO Biological Processes | locomotory exploration behavior | -2.1 | 3.6 | 3.3 | 12 |
| GO Biological Processes | DNA integrity checkpoint | -2.1 | 1.6 | 2.7 | 158 |
| GO Biological Processes | gamete generation | -2.1 | 1.3 | 2.5 | 690 |
| GO Biological Processes | regulation of glucose transmembrane transport | -2.1 | 1.9 | 2.8 | 79 |
| GO Biological Processes | regulation of gastrulation | -2.1 | 2.2 | 2.9 | 43 |
| GO Biological Processes | negative regulation of cell-matrix adhesion | -2.1 | 2.2 | 2.9 | 43 |
| GO Biological Processes | gonad development | -2.1 | 1.5 | 2.6 | 214 |
| GO Biological Processes | regulation of neuron death | -2.1 | 1.4 | 2.6 | 300 |
| GO Biological Processes | meiotic cell cycle phase transition | -2.1 | 4.3 | 3.4 | 8 |
| GO Biological Processes | epithelial fluid transport | -2.1 | 4.3 | 3.4 | 8 |
| GO Biological Processes | positive regulation of lipopolysaccharide-mediated signaling pathway | -2.1 | 4.3 | 3.4 | 8 |
| GO Biological Processes | transforming growth factor beta2 production | -2.1 | 4.3 | 3.4 | 8 |
| GO Biological Processes | blood vessel maturation | -2.1 | 4.3 | 3.4 | 8 |
| GO Biological Processes | ventricular compact myocardium morphogenesis | -2.1 | 4.3 | 3.4 | 8 |
| GO Biological Processes | negative regulation of protein activation cascade | -2.1 | 4.3 | 3.4 | 8 |
| GO Biological Processes | regulation of meiotic cell cycle phase transition | -2.1 | 4.3 | 3.4 | 8 |
| GO Biological Processes | positive regulation of integrin activation | -2.1 | 4.3 | 3.4 | 8 |
| GO Biological Processes | cellular response to caffeine | -2.1 | 4.3 | 3.4 | 8 |
| GO Biological Processes | regulation of blood volume by renin-angiotensin | -2.1 | 4.3 | 3.4 | 8 |
| GO Biological Processes | negative regulation of norepinephrine secretion | -2.1 | 4.3 | 3.4 | 8 |
| GO Biological Processes | regulation of transforming growth factor beta2 production | -2.1 | 4.3 | 3.4 | 8 |

| | | | | | |
|---|---|---|---|---|---|
| GO Biological Processes | endocardial cell differentiation | -2.1 | 4.3 | 3.4 | 8 |
| GO Biological Processes | cardiac neural crest cell migration involved in outflow tract morphogenesis | -2.1 | 4.3 | 3.4 | 8 |
| GO Biological Processes | negative regulation of mononuclear cell migration | -2.1 | 4.3 | 3.4 | 8 |
| GO Biological Processes | metanephric glomerulus vasculature development | -2.1 | 4.3 | 3.4 | 8 |
| GO Biological Processes | cardiac endothelial cell differentiation | -2.1 | 4.3 | 3.4 | 8 |
| GO Biological Processes | regulation of cell adhesion molecule production | -2.1 | 4.3 | 3.4 | 8 |
| GO Biological Processes | response to cisplatin | -2.1 | 4.3 | 3.4 | 8 |
| GO Biological Processes | ureteric bud elongation | -2.1 | 4.3 | 3.4 | 8 |
| GO Biological Processes | positive regulation of AMPA receptor activity | -2.1 | 4.3 | 3.4 | 8 |
| GO Biological Processes | negative regulation of complement activation | -2.1 | 4.3 | 3.4 | 8 |
| GO Biological Processes | neutrophil apoptotic process | -2.1 | 4.3 | 3.4 | 8 |
| GO Biological Processes | regulation of axon extension | -2.1 | 1.8 | 2.7 | 92 |
| GO Biological Processes | import across plasma membrane | -2.1 | 1.7 | 2.7 | 105 |
| GO Biological Processes | positive regulation of leukocyte differentiation | -2.1 | 1.6 | 2.6 | 145 |
| GO Biological Processes | G2 DNA damage checkpoint | -2.1 | 2.4 | 2.9 | 32 |
| GO Biological Processes | protein homotrimerization | -2.1 | 2.4 | 2.9 | 32 |
| GO Biological Processes | postsynapse assembly | -2.1 | 2.4 | 2.9 | 32 |
| GO Biological Processes | regulation of oligodendrocyte differentiation | -2.1 | 2.4 | 2.9 | 32 |
| GO Biological Processes | cell-cell signaling involved in cardiac conduction | -2.1 | 2.4 | 2.9 | 32 |
| GO Biological Processes | regulation of astrocyte differentiation | -2.1 | 2.4 | 2.9 | 32 |
| GO Biological Processes | purinergic receptor signaling pathway | -2.1 | 2.4 | 2.9 | 32 |
| GO Biological Processes | positive regulation of G protein-coupled receptor signaling pathway | -2.1 | 2.4 | 2.9 | 32 |
| GO Biological Processes | negative regulation of double-strand break repair | -2.1 | 2.4 | 2.9 | 32 |
| GO Biological Processes | positive regulation of smooth muscle contraction | -2.1 | 2.4 | 2.9 | 32 |
| GO Biological Processes | proximal/distal pattern formation | -2.1 | 2.4 | 2.9 | 32 |
| GO Biological Processes | muscle fiber development | -2.1 | 1.9 | 2.8 | 67 |
| GO Biological Processes | negative regulation of G2/M transition of mitotic cell cycle | -2.1 | 2.1 | 2.8 | 49 |
| GO Biological Processes | glial cell activation | -2.1 | 2.1 | 2.8 | 49 |
| GO Biological Processes | membrane repolarization | -2.1 | 2.1 | 2.8 | 49 |
| GO Biological Processes | embryonic pattern specification | -2 | 2 | 2.8 | 61 |
| GO Biological Processes | cellular defense response | -2 | 2 | 2.8 | 55 |
| GO Biological Processes | regulation of leukocyte apoptotic process | -2 | 1.8 | 2.7 | 86 |
| GO Biological Processes | positive regulation of phosphatidylinositol 3-kinase signaling | -2 | 1.8 | 2.7 | 86 |
| GO Biological Processes | regulation of calcium ion transmembrane transporter activity | -2 | 1.8 | 2.7 | 86 |
| GO Biological Processes | positive regulation of reactive oxygen species metabolic process | -2 | 1.7 | 2.7 | 99 |
| GO Biological Processes | negative regulation of cell projection organization | -2 | 1.5 | 2.6 | 180 |
| GO Biological Processes | regulation of lymphocyte proliferation | -2 | 1.5 | 2.6 | 208 |
| GO Biological Processes | negative regulation of establishment of protein localization | -2 | 1.5 | 2.6 | 187 |
| GO Biological Processes | response to peptide hormone | -2 | 1.3 | 2.5 | 427 |
| GO Biological Processes | response to immobilization stress | -2 | 2.6 | 2.9 | 27 |
| GO Biological Processes | positive regulation of vascular endothelial growth factor production | -2 | 2.6 | 2.9 | 27 |

| | | | | | |
|---|---|---|---|---|---|
| GO Biological Processes | negative regulation of amine transport | -2 | 2.6 | 2.9 | 27 |
| GO Biological Processes | cellular response to copper ion | -2 | 2.6 | 2.9 | 27 |
| GO Biological Processes | regulation of heterotypic cell-cell adhesion | -2 | 2.6 | 2.9 | 27 |
| GO Biological Processes | positive regulation of coagulation | -2 | 2.6 | 2.9 | 27 |
| GO Biological Processes | collateral sprouting | -2 | 2.6 | 2.9 | 27 |
| GO Biological Processes | cellular amino acid catabolic process | -2 | 1.7 | 2.6 | 119 |
| GO Biological Processes | regulation of transforming growth factor beta receptor signaling pathway | -2 | 1.7 | 2.6 | 119 |
| GO Biological Processes | rhythmic process | -2 | 1.4 | 2.5 | 280 |
| GO Biological Processes | response to monoamine | -2 | 2.3 | 2.8 | 38 |
| GO Biological Processes | apoptotic process involved in development | -2 | 2.3 | 2.8 | 38 |
| GO Biological Processes | response to catecholamine | -2 | 2.3 | 2.8 | 38 |
| GO Biological Processes | ventricular cardiac muscle cell action potential | -2 | 2.3 | 2.8 | 38 |
| GO Biological Processes | maintenance of cell polarity | -2 | 3 | 3 | 17 |
| GO Biological Processes | regulation of platelet aggregation | -2 | 3 | 3 | 17 |
| GO Biological Processes | negative regulation of interleukin-10 production | -2 | 3 | 3 | 17 |
| GO Biological Processes | DNA strand elongation involved in DNA replication | -2 | 3 | 3 | 17 |
| GO Biological Processes | norepinephrine secretion | -2 | 3 | 3 | 17 |
| GO Biological Processes | positive regulation of bone resorption | -2 | 3 | 3 | 17 |
| GO Biological Processes | female genitalia development | -2 | 3 | 3 | 17 |
| GO Biological Processes | wound healing, spreading of epidermal cells | -2 | 3 | 3 | 17 |
| GO Biological Processes | positive regulation of bone remodeling | -2 | 3 | 3 | 17 |
| GO Biological Processes | glycosaminoglycan biosynthetic process | -2 | 1.7 | 2.6 | 106 |
| GO Biological Processes | leukocyte apoptotic process | -2 | 1.7 | 2.6 | 106 |
| GO Biological Processes | synaptic vesicle transport | -2 | 1.6 | 2.6 | 153 |
| GO Biological Processes | establishment of synaptic vesicle localization | -2 | 1.6 | 2.6 | 153 |
| GO Biological Processes | regulation of cell killing | -2 | 1.8 | 2.7 | 93 |
| GO Biological Processes | regulation of supramolecular fiber organization | -2 | 1.4 | 2.5 | 346 |
| GO Biological Processes | activation of protein kinase activity | -2 | 1.4 | 2.5 | 324 |
| GO Biological Processes | mesoderm formation | -2 | 1.9 | 2.7 | 74 |
| GO Biological Processes | response to anesthetic | -2 | 1.9 | 2.7 | 74 |
| GO Biological Processes | interleukin-2 biosynthetic process | -2 | 2.7 | 3 | 22 |
| GO Biological Processes | regulation of interleukin-5 production | -2 | 2.7 | 3 | 22 |
| GO Biological Processes | lung cell differentiation | -2 | 2.7 | 3 | 22 |
| GO Biological Processes | steroid hormone secretion | -2 | 2.7 | 3 | 22 |
| GO Biological Processes | uterus development | -2 | 2.7 | 3 | 22 |
| GO Biological Processes | renal sodium excretion | -2 | 2.7 | 3 | 22 |
| GO Biological Processes | positive regulation of neurotransmitter secretion | -2 | 2.7 | 3 | 22 |
| GO Biological Processes | regulation of mononuclear cell proliferation | -2 | 1.5 | 2.5 | 209 |

| Category | Description | LogP | Enrichment | Z-score | GeneInGO |
|---|---|---|---|---|---|
| KEGG Pathway | PI3K-Akt signaling pathway | -35 | 2.1 | 3,8 | 200 |
| KEGG Pathway | cAMP signaling pathway | -35 | 4.1 | 2.1 | 102 |
| KEGG Pathway | Calcium signaling pathway | -25 | 1.8 | 10 | 99 |
| KEGG Pathway | BMP signaling pathway | -25 | 1.7 | 3.1 | 154 |
| KEGG Pathway | canonical Wnt signaling pathway | -25 | 1.8 | 4.8 | 285 |
| KEGG Pathway | IL-17 signaling pathway | -25 | 2.3 | 13 | 75 |
| KEGG Pathway | Wnt signaling pathway | -25 | 1.5 | 4.3 | 469 |
| KEGG Pathway | Neuroactive ligand-receptor interaction | -10 | 2.2 | 7.3 | 278 |
| KEGG Pathway | Protein digestion and absorption | -8 | 3 | 6.8 | 90 |
| KEGG Pathway | Complement and coagulation cascades | -7.6 | 3.1 | 6.6 | 79 |
| KEGG Pathway | Cell adhesion molecules (CAMs) | -6 | 2.3 | 5.5 | 145 |
| KEGG Pathway | Bile secretion | -5.5 | 2.8 | 5.5 | 71 |
| KEGG Pathway | ECM-receptor interaction | -5.4 | 2.6 | 5.3 | 82 |
| KEGG Pathway | ABC transporters | -5.3 | 3.3 | 5.5 | 45 |
| KEGG Pathway | Cell cycle | -5 | 2.2 | 4.9 | 124 |
| KEGG Pathway | Malaria | -4.7 | 3 | 5 | 49 |
| KEGG Pathway | Salivary secretion | -4.2 | 2.3 | 4.5 | 90 |
| KEGG Pathway | Pathways in cancer | -4.2 | 1.6 | 4.1 | 395 |
| KEGG Pathway | Tyrosine metabolism | -4.1 | 3.2 | 4.7 | 35 |
| KEGG Pathway | Cytokine-cytokine receptor interaction | -4.1 | 1.7 | 4.1 | 270 |
| KEGG Pathway | Vascular smooth muscle contraction | -4 | 2.1 | 4.3 | 121 |
| KEGG Pathway | Calcium signaling pathway | -4 | 1.8 | 4.2 | 182 |
| KEGG Pathway | Phenylalanine metabolism | -3.5 | 4.1 | 4.6 | 17 |
| KEGG Pathway | cAMP signaling pathway | -3.5 | 1.7 | 3.8 | 198 |
| KEGG Pathway | AGE-RAGE signaling pathway in diabetic complications | -3.5 | 2.1 | 3.9 | 99 |
| KEGG Pathway | Tryptophan metabolism | -3.4 | 2.8 | 4.1 | 40 |
| KEGG Pathway | Renin-angiotensin system | -3.2 | 3.4 | 4.1 | 23 |
| KEGG Pathway | Osteoclast differentiation | -3.1 | 1.9 | 3.5 | 130 |
| KEGG Pathway | PPAR signaling pathway | -2.9 | 2.2 | 3.6 | 72 |
| KEGG Pathway | Pancreatic secretion | -2.9 | 2 | 3.5 | 96 |
| KEGG Pathway | Focal adhesion | -2.9 | 1.6 | 3.3 | 199 |
| KEGG Pathway | Staphylococcus aureus infection | -2.9 | 2.3 | 3.5 | 56 |
| KEGG Pathway | Prion diseases | -2.8 | 2.7 | 3.7 | 35 |
| KEGG Pathway | Arachidonic acid metabolism | -2.8 | 2.2 | 3.5 | 62 |
| KEGG Pathway | O-glycan biosynthesis, mucin type core | -2.6 | 2.9 | 3.5 | 27 |
| KEGG Pathway | O-glycan biosynthesis, mucin type core | -2.6 | 2.9 | 3.5 | 27 |
| KEGG Pathway | Hypertrophic cardiomyopathy (HCM) | -2.6 | 2 | 3.2 | 83 |
| KEGG Pathway | Transcriptional misregulation in cancer | -2.5 | 1.6 | 3.1 | 180 |
| KEGG Pathway | Rheumatoid arthritis | -2.5 | 1.9 | 3.1 | 90 |
| KEGG Pathway | Dilated cardiomyopathy | -2.5 | 1.9 | 3.1 | 90 |
| KEGG Pathway | PI3K-Akt signaling pathway | -2.5 | 1.4 | 2.9 | 342 |

| | | | | | |
|---|---|---|---|---|---|
| KEGG Pathway | IL-17 signaling pathway | -2.3 | 1.9 | 3 | 93 |
| KEGG Pathway | Renin secretion | -2.2 | 2 | 2.9 | 65 |
| KEGG Pathway | Mucin type O-glycan biosynthesis | -2.2 | 2.5 | 3 | 31 |
| KEGG Pathway | Hematopoietic cell lineage | -2.1 | 1.8 | 2.8 | 97 |
| KEGG Pathway | Maturity onset diabetes of the young | -2.1 | 2.7 | 3.1 | 26 |
| KEGG Pathway | Glycosphingolipid biosynthesis - lacto and neolacto series | -2 | 2.6 | 2.9 | 27 |
| KEGG Pathway | Nitrogen metabolism | -2 | 3 | 3 | 17 |

Table Supplementary 9: Subgroup

| Type | Symbol | Type | Symbol | Type | Symbol | Type | Symbol |
|---|---|---|---|---|---|---|---|
| circRNA | hsa-circRNA15551-1 | lncRNA | CROCCP2 | miRNA | hsa-mir-145 | mRNA | ZEB1 |
| | hsa-circRNA15551-2 | | CTC-459F4.3 | | hsa-mir-205 | | NLN |
| | hsa-circRNA11554-1 | | DCP1A | | | | NFAM1 |
| | hsa-circRNA11554-2 | | FGD5-AS1 | | | | NEDD9 |
| | hsa-circRNA11554-3 | | HCG18 | | | | THRB |
| | hsa-circRNA11554-5 | | HOXA11-AS | | | | PRKCE |
| | hsa-circRNA11554-6 | | MAP3K14 | | | | GPC3 |
| | hsa-circRNA11554-7 | | PPP1R9B | | | | LAMA3 |
| | hsa-circRNA11554-27 | | RP11-1055B8.4 | | | | FGFR2 |
| | hsa-circRNA11554-28 | | RP11-156E6.1 | | | | COL1A1 |
| | hsa-circRNA11554-29 | | RP11-15H20.6 | | | | CCNE2 |
| | hsa-circRNA11554-30 | | RP11-834C11.4 | | | | EZH2 |
| | hsa-circRNA11554-31 | | RP1-283E3.8 | | | | |
| | hsa-circRNA11554-32 | | SLC38A3 | | | | |
| | hsa-circRNA11554-35 | | | | | | |
| | hsa-circRNA11554-36 | | | | | | |
| | hsa-circRNA11554-37 | | | | | | |
| | hsa-circRNA11554-38 | | | | | | |
| | hsa-circRNA11554-39 | | | | | | |
| | hsa-circRNA11554-40 | | | | | | |
| | hsa-circRNA11554-41 | | | | | | |
| | hsa-circRNA11554-42 | | | | | | |
| | hsa-circRNA11554-43 | | | | | | |
| | hsa-circRNA11554-44 | | | | | | |
| | hsa-circRNA11554-45 | | | | | | |
| | hsa-circRNA11554-46 | | | | | | |
| | hsa-circRNA11554-47 | | | | | | |
| | hsa-circRNA11554-48 | | | | | | |
| | hsa-circRNA11554-49 | | | | | | |
| | hsa-circRNA11554-58 | | | | | | |
| | hsa-circRNA11554-72 | | | | | | |
| | hsa-circRNA11554-73 | | | | | | |
| | hsa-circRNA11554-74 | | | | | | |
| | hsa-circRNA11554-75 | | | | | | |
| | hsa-circRNA11554-77 | | | | | | |
| | hsa-circRNA11554-78 | | | | | | |
| | hsa-circRNA11554-79 | | | | | | |
| | hsa-circRNA11554-80 | | | | | | |
| | hsa-circRNA11554-20 | | | | | | |
| | hsa-circRNA11554-12 | | | | | | |
| | hsa-circRNA11554-66 | | | | | | |
| | hsa-circRNA11554-33 | | | | | | |
| | hsa-circRNA11554-4 | | | | | | |
| | hsa-circRNA11554-34 | | | | | | |
| | hsa-circRNA11554-76 | | | | | | |
| | hsa-circRNA15355-2 | | | | | | |
| | hsa-circRNA15355-3 | | | | | | |
| | hsa-circRNA15355-4 | | | | | | |
| | hsa-circRNA15355-5 | | | | | | |
| | hsa-circRNA15355-31 | | | | | | |
| | hsa-circRNA15355-6 | | | | | | |
| | hsa-circRNA15355-10 | | | | | | |
| | hsa-circRNA15355-11 | | | | | | |
| | hsa-circRNA15355-13 | | | | | | |
| | hsa-circRNA15355-14 | | | | | | |
| | hsa-circRNA15355-18 | | | | | | |
| | hsa-circRNA15355-19 | | | | | | |

| |
|---|
| hsa-circRNA15355-24 |
| hsa-circRNA15355-25 |
| hsa-circRNA15355-32 |
| hsa-circRNA15355-1 |
| hsa-circRNA9316-2 |
| hsa-circRNA9316-1 |
| hsa-circRNA9316-5 |
| hsa-circRNA9316-8 |
| hsa-circRNA9316-9 |
| hsa-circRNA9316-10 |
| hsa-circRNA9316-4 |
| hsa-circRNA9316-6 |
| hsa-circRNA9316-7 |
| hsa-circRNA16245-3 |
| hsa-circRNA16245-5 |
| hsa-circRNA16245-1 |
| hsa-circRNA16245-2 |
| hsa-circRNA16245-4 |
| hsa-circRNA16245-6 |
| hsa-circRNA16245-7 |
| hsa-circRNA3631-1 |
| hsa-circRNA3631-2 |
| hsa-circRNA3631-3 |
| hsa-circRNA3631-4 |
| hsa-circRNA3631-6 |
| hsa-circRNA3631-84 |
| hsa-circRNA3631-85 |
| hsa-circRNA3631-86 |
| hsa-circRNA3631-87 |
| hsa-circRNA3631-88 |
| hsa-circRNA3631-92 |
| hsa-circRNA3631-93 |
| hsa-circRNA3631-94 |
| hsa-circRNA3631-95 |
| hsa-circRNA3631-5 |
| hsa-circRNA3631-9 |
| hsa-circRNA3631-69 |
| hsa-circRNA3631-70 |
| hsa-circRNA3631-82 |
| hsa-circRNA3631-66 |
| hsa-circRNA14596-1 |
| hsa-circRNA14596-2 |
| hsa-circRNA14596-3 |
| hsa-circRNA14596-4 |
| hsa-circRNA14596-5 |
| hsa-circRNA14596-6 |
| hsa-circRNA14597 |
| hsa-circRNA14599-1 |
| hsa-circRNA14599-2 |
| hsa-circRNA4427-1 |
| hsa-circRNA4427-2 |
| hsa-circRNA4427-3 |
| hsa-circRNA4427-4 |
| hsa-circRNA4427-5 |
| hsa-circRNA4427-6 |
| hsa-circRNA4427-7 |
| hsa-circRNA4427-10 |
| hsa-circRNA4426-8 |
| hsa-circRNA4426-9 |
| hsa-circRNA4426-10 |

| |
|---|
| hsa-circRNA4426-11 |
| hsa-circRNA4426-12 |
| hsa-circRNA4426-13 |
| hsa-circRNA4426-14 |
| hsa-circRNA4427-8 |
| hsa-circRNA4426-2 |
| hsa-circRNA4426-6 |
| hsa-circRNA4427-9 |
| hsa-circRNA13468-1 |
| hsa-circRNA13468-3 |
| hsa-circRNA13468-4 |
| hsa-circRNA13468-5 |
| hsa-circRNA13468-6 |
| hsa-circRNA13468-7 |
| hsa-circRNA13468-10 |
| hsa-circRNA13468-12 |
| hsa-circRNA13468-2 |
| hsa-circRNA13468-8 |
| hsa-circRNA13468-9 |
| hsa-circRNA13468-11 |
| hsa-circRNA897-2 |
| hsa-circRNA897-4 |
| hsa-circRNA897-5 |
| hsa-circRNA897-6 |
| hsa-circRNA897-10 |
| hsa-circRNA897-13 |
| hsa-circRNA897-14 |
| hsa-circRNA897-19 |
| hsa-circRNA897-20 |
| hsa-circRNA897-21 |
| hsa-circRNA897-22 |
| hsa-circRNA897-23 |
| hsa-circRNA897-24 |
| hsa-circRNA897-27 |
| hsa-circRNA897-29 |
| hsa-circRNA897-30 |
| hsa-circRNA897-31 |
| hsa-circRNA897-32 |
| hsa-circRNA897-9 |
| hsa-circRNA897-12 |
| hsa-circRNA897-26 |
| hsa-circRNA897-28 |
| hsa-circRNA897-16 |
| hsa-circRNA897-18 |
| hsa-circRNA897-3 |
| hsa-circRNA897-7 |
| hsa-circRNA897-11 |
| hsa-circRNA897-8 |
| hsa-circRNA897-15 |
| hsa-circRNA897-17 |
| hsa-circRNA897-25 |
| hsa-circRNA897-1 |

Table Supplementary 10: KEGG and GO analysis of key gene set

| Description | pvalue | Enrichment | Z-score | %InGO | STDV %InGO |
|---|---|---|---|---|---|
| drug catabolic process | 1.00E-25 | 2.6 | 12 | 4.5 | 0.39 |
| developmental cell growth | 1.00E-23 | 2.7 | 12 | 4 | 0.37 |
| Wnt signaling pathway | 1.00E-21 | 2.1 | 11 | 5.8 | 0.44 |
| PI3K-Akt signaling pathway | 1.00E-20 | 2.2 | 11 | 5.5 | 0.43 |
| drug metabolic process | 1.00E-20 | 2 | 10 | 6.3 | 0.46 |
| cell-cell signaling by wnt | 1.00E-19 | 2.1 | 10 | 5.7 | 0.44 |
| drug transport | 1.00E-19 | 2 | 9.9 | 6.5 | 0.46 |
| drug transmembrane transport | 1.00E-18 | 2 | 9.8 | 5.7 | 0.44 |
| transcriptional misregulation in cancer | 1.00E-17 | 2.5 | 9.9 | 3.4 | 0.34 |
| fatty acid derivative metabolic process | 1.00E-16 | 2 | 9.3 | 5.2 | 0.42 |
| histone phosphorylation | 1.00E-16 | 2.1 | 9.3 | 4.8 | 0.4 |
| epithelial cell migration | 1.00E-16 | 2.6 | 9.7 | 3.1 | 0.33 |
| lymphocyte proliferation | 1.00E-16 | 2 | 9.2 | 5.2 | 0.42 |
| mesonephric epithelium development | 1.00E-15 | 2 | 8.9 | 4.8 | 0.4 |
| BMP sinagling pathway | 1.00E-15 | 2.5 | 9.3 | 2.9 | 0.32 |
| canonical Wnt signaling pathway | 1.00E-15 | 2 | 8.9 | 4.9 | 0.41 |
| regulation of MAPK cascade | 1.00E-15 | 2 | 8.9 | 4.9 | 0.41 |
| calcitonin family receptor signaling pathway | 1.00E-15 | 2.5 | 9.3 | 2.9 | 0.32 |
| pathways in cancer | 1.00E-15 | 2 | 8.8 | 4.6 | 0.39 |
| cell surface receptor signaling pathway involved in cell-cell signaling | 1.00E-15 | 2 | 8.7 | 4.6 | 0.4 |

Table Supplementary 11: KEGG and GO analysis of key target genes

| Category | GO | Description | LogP | Enrichment | Z-score | GeneInGO |
|---|---|---|---|---|---|---|
| GO Biological Processes | GO:0009636 | response to toxic substance | -9 | 22 | 13 | 525 |
| GO Biological Processes | GO:0046677 | response to antibiotic | -8.9 | 30 | 14 | 328 |
| GO Biological Processes | GO:0071229 | cellular response to acid chemical | -6.6 | 34 | 13 | 209 |
| GO Biological Processes | GO:0097237 | cellular response to toxic substance | -6.2 | 29 | 12 | 247 |
| GO Biological Processes | GO:0045471 | response to ethanol | -5.8 | 45 | 13 | 126 |
| GO Biological Processes | GO:0071236 | cellular response to antibiotic | -5.5 | 39 | 12 | 147 |
| GO Biological Processes | GO:0090263 | positive regulation of canonical Wnt signaling pathway | -5.5 | 39 | 12 | 147 |
| GO Biological Processes | GO:0001101 | response to acid chemical | -5.5 | 21 | 9.7 | 344 |
| GO Biological Processes | GO:0010463 | mesenchymal cell proliferation | -5.4 | 97 | 17 | 44 |
| GO Biological Processes | GO:0040008 | regulation of growth | -5.3 | 13 | 8.1 | 682 |
| GO Biological Processes | GO:0030177 | positive regulation of Wnt signaling pathway | -5.2 | 32 | 11 | 179 |
| GO Biological Processes | GO:0051147 | regulation of muscle cell differentiation | -5.2 | 31 | 11 | 183 |
| GO Biological Processes | GO:0071300 | cellular response to retinoic acid | -4.8 | 62 | 13 | 69 |
| GO Biological Processes | GO:0000302 | response to reactive oxygen species | -4.7 | 24 | 9.5 | 233 |
| GO Biological Processes | GO:0097305 | response to alcohol | -4.7 | 24 | 9.5 | 234 |
| GO Biological Processes | GO:0043410 | positive regulation of MAPK cascade | -4.5 | 13 | 7.5 | 548 |
| GO Biological Processes | GO:0060828 | regulation of canonical Wnt signaling pathway | -4.4 | 20 | 8.5 | 286 |
| KEGG Pathway | hsa05206 | MicroRNAs in cancer | -4.3 | 19 | 8.3 | 299 |
| GO Biological Processes | GO:0071396 | cellular response to lipid | -4.3 | 12 | 7.1 | 610 |
| GO Biological Processes | GO:0032526 | response to retinoic acid | -4.2 | 39 | 11 | 108 |
| GO Biological Processes | GO:0045664 | regulation of neuron differentiation | -4.2 | 11 | 6.8 | 656 |
| GO Biological Processes | GO:0060070 | canonical Wnt signaling pathway | -4.1 | 17 | 7.8 | 335 |
| KEGG Pathway | hsa04151 | PI3K-Akt signaling pathway | -4.1 | 17 | 7.7 | 342 |
| GO Biological Processes | GO:0009611 | response to wounding | -4.1 | 10 | 6.6 | 682 |
| GO Biological Processes | GO:0048706 | embryonic skeletal system development | -4 | 34 | 9.8 | 126 |
| GO Biological Processes | GO:0031589 | cell-substrate adhesion | -4 | 16 | 7.5 | 356 |
| GO Biological Processes | GO:0071363 | cellular response to growth factor stimulus | -4 | 10 | 6.5 | 709 |
| GO Biological Processes | GO:0018108 | peptidyl-tyrosine phosphorylation | -4 | 16 | 7.5 | 364 |
| GO Biological Processes | GO:0030111 | regulation of Wnt signaling pathway | -4 | 16 | 7.4 | 366 |
| GO Biological Processes | GO:0018212 | peptidyl-tyrosine modification | -4 | 15 | 7.4 | 367 |
| GO Biological Processes | GO:0035690 | cellular response to drug | -4 | 15 | 7.4 | 369 |
| GO Biological Processes | GO:0070848 | response to growth factor | -3.9 | 9.6 | 6.3 | 739 |
| GO Biological Processes | GO:0001837 | epithelial to mesenchymal transition | -3.9 | 30 | 9.2 | 142 |
| GO Biological Processes | GO:0042692 | muscle cell differentiation | -3.9 | 15 | 7.2 | 387 |
| GO Biological Processes | GO:0045596 | negative regulation of cell differentiation | -3.9 | 9.4 | 6.2 | 757 |
| GO Biological Processes | GO:0042542 | response to hydrogen peroxide | -3.9 | 29 | 9.1 | 146 |
| KEGG Pathway | hsa05200 | Pathways in cancer | -3.8 | 14 | 7.1 | 395 |
| GO Biological Processes | GO:0051345 | positive regulation of hydrolase activity | -3.8 | 9.2 | 6.2 | 771 |
| GO Biological Processes | GO:0043408 | regulation of MAPK cascade | -3.8 | 9.2 | 6.1 | 774 |
| GO Biological Processes | GO:0043547 | positive regulation of GTPase activity | -3.8 | 14 | 7 | 405 |
| GO Biological Processes | GO:0030855 | epithelial cell differentiation | -3.8 | 9.1 | 6.1 | 781 |
| GO Biological Processes | GO:0045859 | regulation of protein kinase activity | -3.8 | 8.9 | 6 | 796 |
| GO Biological Processes | GO:0031214 | biomineral tissue development | -3.7 | 26 | 8.6 | 163 |

| | | | | | | |
|---|---|---|---|---|---|---|
| GO Biological Processes | GO:0034614 | cellular response to reactive oxygen species | -3.7 | 25 | 8.4 | 168 |
| GO Biological Processes | GO:0030324 | lung development | -3.6 | 25 | 8.3 | 172 |
| GO Biological Processes | GO:0030323 | respiratory tube development | -3.6 | 24 | 8.2 | 176 |
| GO Biological Processes | GO:0006979 | response to oxidative stress | -3.6 | 13 | 6.6 | 454 |
| GO Biological Processes | GO:0007369 | gastrulation | -3.6 | 23 | 8 | 185 |
| GO Biological Processes | GO:0043087 | regulation of GTPase activity | -3.5 | 12 | 6.4 | 479 |
| GO Biological Processes | GO:0022604 | regulation of cell morphogenesis | -3.5 | 12 | 6.3 | 484 |
| GO Biological Processes | GO:0048839 | inner ear development | -3.5 | 22 | 7.8 | 192 |
| GO Biological Processes | GO:0001763 | morphogenesis of a branching structure | -3.5 | 22 | 7.7 | 196 |
| GO Biological Processes | GO:0060541 | respiratory system development | -3.5 | 22 | 7.7 | 198 |
| GO Biological Processes | GO:0002521 | leukocyte differentiation | -3.4 | 11 | 6.1 | 516 |
| GO Biological Processes | GO:0071900 | regulation of protein serine/threonine kinase activity | -3.4 | 11 | 6.1 | 519 |
| GO Biological Processes | GO:0016055 | Wnt signaling pathway | -3.4 | 11 | 6.1 | 522 |
| GO Biological Processes | GO:0198738 | cell-cell signaling by wnt | -3.4 | 11 | 6 | 524 |
| GO Biological Processes | GO:1903827 | regulation of cellular protein localization | -3.4 | 11 | 6 | 524 |
| GO Biological Processes | GO:0010810 | regulation of cell-substrate adhesion | -3.4 | 20 | 7.4 | 215 |
| GO Biological Processes | GO:0045860 | positive regulation of protein kinase activity | -3.3 | 11 | 6 | 534 |
| GO Biological Processes | GO:0043583 | ear development | -3.3 | 19 | 7.3 | 219 |
| GO Biological Processes | GO:0048762 | mesenchymal cell differentiation | -3.3 | 19 | 7.2 | 221 |
| GO Biological Processes | GO:0071407 | cellular response to organic cyclic compound | -3.3 | 10 | 5.9 | 542 |
| GO Biological Processes | GO:0030335 | positive regulation of cell migration | -3.3 | 10 | 5.9 | 544 |
| GO Biological Processes | GO:0007423 | sensory organ development | -3.3 | 10 | 5.9 | 548 |
| GO Biological Processes | GO:0010720 | positive regulation of cell development | -3.3 | 10 | 5.9 | 553 |
| GO Biological Processes | GO:0010035 | response to inorganic substance | -3.3 | 10 | 5.8 | 562 |
| GO Biological Processes | GO:0042060 | wound healing | -3.3 | 10 | 5.8 | 567 |
| GO Biological Processes | GO:2000147 | positive regulation of cell motility | -3.2 | 10 | 5.8 | 568 |
| GO Biological Processes | GO:0048705 | skeletal system morphogenesis | -3.2 | 18 | 6.9 | 239 |
| GO Biological Processes | GO:0033674 | positive regulation of kinase activity | -3.2 | 9.9 | 5.7 | 575 |
| GO Biological Processes | GO:0051272 | positive regulation of cellular component movement | -3.2 | 9.7 | 5.7 | 585 |
| GO Biological Processes | GO:0048598 | embryonic morphogenesis | -3.2 | 9.7 | 5.7 | 586 |
| GO Biological Processes | GO:0071417 | cellular response to organonitrogen compound | -3.2 | 9.6 | 5.6 | 589 |
| GO Biological Processes | GO:0040017 | positive regulation of locomotion | -3.2 | 9.5 | 5.6 | 598 |
| GO Biological Processes | GO:0051301 | cell division | -3.2 | 9.5 | 5.6 | 598 |
| GO Biological Processes | GO:0071560 | cellular response to transforming growth factor beta stimulus | -3.2 | 17 | 6.7 | 252 |
| GO Biological Processes | GO:2000027 | regulation of animal organ morphogenesis | -3.2 | 17 | 6.7 | 254 |
| GO Biological Processes | GO:0090596 | sensory organ morphogenesis | -3.1 | 17 | 6.7 | 256 |
| GO Biological Processes | GO:0071559 | response to transforming growth factor beta | -3.1 | 17 | 6.7 | 258 |
| GO Biological Processes | GO:0043406 | positive regulation of MAP kinase activity | -3.1 | 16 | 6.6 | 260 |
| GO Biological Processes | GO:0031400 | negative regulation of protein modification process | -3.1 | 9.2 | 5.5 | 618 |
| GO Biological Processes | GO:1905114 | cell surface receptor signaling pathway involved in cell-cell signaling | -3.1 | 9.1 | 5.4 | 625 |
| GO Biological Processes | GO:0060485 | mesenchyme development | -3 | 15 | 6.4 | 279 |
| GO Biological Processes | GO:0001822 | kidney development | -3 | 15 | 6.4 | 279 |
| GO Biological Processes | GO:0048812 | neuron projection morphogenesis | -3 | 8.8 | 5.3 | 648 |
| GO Biological Processes | GO:0051347 | positive regulation of transferase activity | -3 | 8.7 | 5.3 | 651 |
| GO Biological Processes | GO:1901699 | cellular response to nitrogen compound | -3 | 8.7 | 5.3 | 652 |

| | | | | | | |
|---|---|---|---|---|---|---|
| GO Biological Processes | GO:0002274 | myeloid leukocyte activation | -3 | 8.7 | 5.3 | 653 |
| GO Biological Processes | GO:0120039 | plasma membrane bounded cell projection morphogenesis | -3 | 8.6 | 5.3 | 662 |
| GO Biological Processes | GO:0048858 | cell projection morphogenesis | -3 | 8.5 | 5.2 | 666 |
| GO Biological Processes | GO:0030036 | actin cytoskeleton organization | -3 | 8.5 | 5.2 | 667 |
| GO Biological Processes | GO:0090287 | regulation of cellular response to growth factor stimulus | -3 | 15 | 6.2 | 293 |
| GO Biological Processes | GO:0072001 | renal system development | -3 | 14 | 6.2 | 294 |
| GO Biological Processes | GO:0097435 | supramolecular fiber organization | -3 | 8.4 | 5.2 | 674 |
| GO Biological Processes | GO:0061061 | muscle structure development | -3 | 8.4 | 5.2 | 674 |
| GO Biological Processes | GO:0050768 | negative regulation of neurogenesis | -3 | 14 | 6.2 | 295 |
| GO Biological Processes | GO:0018105 | peptidyl-serine phosphorylation | -2.9 | 14 | 6.1 | 299 |
| GO Biological Processes | GO:0032990 | cell part morphogenesis | -2.9 | 8.3 | 5.1 | 685 |
| GO Biological Processes | GO:0010769 | regulation of cell morphogenesis involved in differentiation | -2.9 | 14 | 6.1 | 301 |
| GO Biological Processes | GO:0034599 | cellular response to oxidative stress | -2.9 | 14 | 6.1 | 304 |
| GO Biological Processes | GO:0030155 | regulation of cell adhesion | -2.9 | 8.2 | 5.1 | 693 |
| GO Biological Processes | GO:0043010 | camera-type eye development | -2.9 | 14 | 6 | 314 |
| GO Biological Processes | GO:0051961 | negative regulation of nervous system development | -2.9 | 14 | 5.9 | 315 |
| GO Biological Processes | GO:0018209 | peptidyl-serine modification | -2.9 | 13 | 5.9 | 322 |
| GO Biological Processes | GO:0001655 | urogenital system development | -2.8 | 13 | 5.8 | 331 |
| GO Biological Processes | GO:0000904 | cell morphogenesis involved in differentiation | -2.8 | 7.6 | 4.9 | 745 |
| GO Biological Processes | GO:0071902 | positive regulation of protein serine/threonine kinase activity | -2.8 | 13 | 5.7 | 336 |
| GO Biological Processes | GO:0043405 | regulation of MAP kinase activity | -2.8 | 13 | 5.7 | 341 |
| GO Biological Processes | GO:0030029 | actin filament-based process | -2.8 | 7.5 | 4.8 | 760 |
| GO Biological Processes | GO:0010721 | negative regulation of cell development | -2.8 | 12 | 5.6 | 344 |
| GO Biological Processes | GO:0001568 | blood vessel development | -2.8 | 7.5 | 4.8 | 762 |
| GO Biological Processes | GO:0030098 | lymphocyte differentiation | -2.7 | 12 | 5.6 | 353 |
| GO Biological Processes | GO:0060322 | head development | -2.7 | 7.3 | 4.7 | 777 |
| GO Biological Processes | GO:0001654 | eye development | -2.7 | 12 | 5.5 | 362 |
| GO Biological Processes | GO:0001944 | vasculature development | -2.7 | 7.2 | 4.7 | 793 |
| GO Biological Processes | GO:0150063 | visual system development | -2.7 | 12 | 5.4 | 366 |
| GO Biological Processes | GO:0030198 | extracellular matrix organization | -2.7 | 12 | 5.4 | 370 |
| GO Biological Processes | GO:0045666 | positive regulation of neuron differentiation | -2.7 | 11 | 5.4 | 371 |
| GO Biological Processes | GO:0048880 | sensory system development | -2.7 | 11 | 5.4 | 371 |
| GO Biological Processes | GO:0030900 | forebrain development | -2.6 | 11 | 5.3 | 382 |
| GO Biological Processes | GO:0048545 | response to steroid hormone | -2.6 | 11 | 5.3 | 385 |
| GO Biological Processes | GO:0045787 | positive regulation of cell cycle | -2.6 | 11 | 5.3 | 389 |
| GO Biological Processes | GO:0001503 | ossification | -2.6 | 11 | 5.2 | 399 |
| GO Biological Processes | GO:0001558 | regulation of cell growth | -2.5 | 10 | 5 | 419 |
| GO Biological Processes | GO:0009896 | positive regulation of catabolic process | -2.5 | 10 | 5 | 424 |
| GO Biological Processes | GO:0043062 | extracellular structure organization | -2.5 | 10 | 5 | 425 |
| GO Biological Processes | GO:0048732 | gland development | -2.5 | 9.8 | 4.9 | 435 |
| GO Biological Processes | GO:0007389 | pattern specification process | -2.5 | 9.6 | 4.8 | 446 |
| GO Biological Processes | GO:0016570 | histone modification | -2.4 | 9.3 | 4.8 | 457 |
| GO Biological Processes | GO:0008544 | epidermis development | -2.4 | 9.2 | 4.7 | 464 |
| GO Biological Processes | GO:0050769 | positive regulation of neurogenesis | -2.4 | 9 | 4.7 | 474 |
| GO Biological Processes | GO:1903706 | regulation of hemopoiesis | -2.4 | 8.9 | 4.6 | 477 |

| | | | | | | |
|---|---|---|---|---|---|---|
| GO Biological Processes | GO:0016569 | covalent chromatin modification | -2.4 | 8.9 | 4.6 | 477 |
| GO Biological Processes | GO:0016049 | cell growth | -2.3 | 8.8 | 4.6 | 487 |
| GO Biological Processes | GO:0010975 | regulation of neuron projection development | -2.3 | 8.5 | 4.5 | 499 |
| GO Biological Processes | GO:0090066 | regulation of anatomical structure size | -2.3 | 8.4 | 4.5 | 507 |
| GO Biological Processes | GO:0001501 | skeletal system development | -2.3 | 8.2 | 4.4 | 518 |
| GO Biological Processes | GO:0002009 | morphogenesis of an epithelium | -2.2 | 7.9 | 4.3 | 540 |
| GO Biological Processes | GO:0051962 | positive regulation of nervous system development | -2.2 | 7.9 | 4.3 | 541 |
| GO Biological Processes | GO:0002694 | regulation of leukocyte activation | -2.1 | 7.4 | 4.1 | 574 |
| GO Biological Processes | GO:0048667 | cell morphogenesis involved in neuron differentiation | -2.1 | 7.3 | 4.1 | 583 |
| GO Biological Processes | GO:0007507 | heart development | -2.1 | 7.2 | 4.1 | 588 |
| GO Biological Processes | GO:0007610 | behavior | -2.1 | 7.2 | 4 | 596 |
| GO Biological Processes | GO:0050865 | regulation of cell activation | -2.1 | 6.9 | 3.9 | 617 |
| GO Biological Processes | GO:0072657 | protein localization to membrane | -2.1 | 6.8 | 3.9 | 623 |
| GO Biological Processes | GO:0010638 | positive regulation of organelle organization | -2.1 | 6.8 | 3.9 | 624 |
| GO Biological Processes | GO:0043009 | chordate embryonic development | -2 | 6.8 | 3.9 | 629 |
| GO Biological Processes | GO:0009792 | embryo development ending in birth or egg hatching | -2 | 6.6 | 3.8 | 646 |
| GO Biological Processes | GO:0048589 | developmental growth | -2 | 6.3 | 3.7 | 674 |
| GO Biological Processes | GO:0048729 | tissue morphogenesis | -2 | 6.3 | 3.7 | 674 |
| GO Biological Processes | GO:0120035 | regulation of plasma membrane bounded cell projection organization | -1.9 | 6.1 | 3.6 | 698 |
| GO Biological Processes | GO:0009617 | response to bacterium | -1.9 | 6.1 | 3.6 | 702 |
| GO Biological Processes | GO:0031344 | regulation of cell projection organization | -1.9 | 6 | 3.6 | 708 |
| GO Biological Processes | GO:0007169 | transmembrane receptor protein tyrosine kinase signaling pathway | -1.9 | 5.9 | 3.6 | 722 |
| GO Biological Processes | GO:0007420 | brain development | -1.9 | 5.8 | 3.5 | 734 |
| GO Biological Processes | GO:0046649 | lymphocyte activation | -1.9 | 5.8 | 3.5 | 736 |
| GO Biological Processes | GO:0080135 | regulation of cellular response to stress | -1.8 | 5.7 | 3.5 | 749 |
| GO Biological Processes | GO:0008285 | negative regulation of cell proliferation | -1.8 | 5.6 | 3.4 | 762 |
